# Global DNA Methylation Analysis Identifies Two Discrete clusters of Pheochromocytoma with Distinct Genomic and Genetic Alterations

**DOI:** 10.1038/srep44943

**Published:** 2017-03-22

**Authors:** Samuel Backman, Rajani Maharjan, Alberto Falk-Delgado, Joakim Crona, Kenko Cupisti, Peter Stålberg, Per Hellman, Peyman Björklund

**Affiliations:** 1 Department of Surgical Sciences, Uppsala University, Uppsala, Sweden; 2 Department of Surgery, Marien-Hospital, Euskirchen, Germany

## Abstract

Pheochromocytomas and paragangliomas (PPGLs) are rare and frequently heritable neural-crest derived tumours arising from the adrenal medulla or extra-adrenal chromaffin cells respectively. The majority of PPGL tumours are benign and do not recur with distant metastases. However, a sizeable fraction of these tumours secrete vasoactive catecholamines into the circulation causing a variety of symptoms including hypertension, palpitations and diaphoresis. The genetic landscape of PPGL has been well characterized and more than a dozen genes have been described as recurrently mutated. Recent studies of DNA-methylation have revealed distinct clusters of PPGL that share DNA methylation patterns and driver mutations, as well as identified potential biomarkers for malignancy. However, these findings have not been adequately validated in independent cohorts. In this study we use an array-based genome-wide approach to study the methylome of 39 PPGL and 4 normal adrenal medullae. We identified two distinct clusters of tumours characterized by different methylation patterns and different driver mutations. Moreover, we identify genes that are differentially methylated between tumour subcategories, and between tumours and normal tissue.

Pheochromocytoma and paraganglioma (PPGL) are neural crest derived chromaffin cell tumours of the adrenal medulla and paraganglia, respectively. The tumours may secrete catecholamines such as epinephrine, norepinephrine and dopamine into the systemic circulation, leading to symptoms of sympathetic hyperactivity. The incidence is 2–8/1,000,000 per year^1^,^2^, and up to 40% of all PPGL are known to be part of inherited syndromes, including Neurofibromatosis type 1, Multiple Endocrine Neoplasia type 2A and 2B (MEN2A/MEN2B) as well as von Hippel-Lindau syndrome^3^. To date, more than a dozen genes have been identified as conferring susceptibility to PPGL: *SDHA, SDHB, SDHC, SDHD, SDHAF2* (from here on abbreviated as *SDHx*), *FH, VHL, RET, EPAS1, TMEM127, MAX, NF1*^3^,^4^,^5^,^6^,^7^,^8^,^9^,^10^,^11^,^12^,^13^ and *MDH2*^14^. In addition, recurrent somatic mutations have been found in the *HRAS*^15^ in sporadic PPGLs. In total these genetic aberrations are the cause of more than 60% of PPGL cases. The histone methyl transferase *KMT2D* has also been reported recurrently mutated^16^, as has the chromatin modulator *ATRX*^17^. However, the functional implications of these mutations remain to be elucidated. PPGL tumours can be sorted into two main clusters, based on driver mutation and gene expression patterns. Cluster 1 contains tumours with mutations in *VHL, EPAS1* and *SDHx*/*FH*/*MDH2*, and is characterized by a pseudohypoxic gene expression signature^18^ caused by the stabilization of EPAS-1 which drives angiogenesis and tumour development^19^,^20^. Cluster 2 contains tumours with mutations in *RET, TMEM127, MAX, NF1* and *HRAS* and is characterized by aberrant activation of kinase signalling pathways^21^. The vast majority of cases are benign and do not cause distant metastases. Approximately ten percent relapse with distant metastases, and metastatic pheochromocytoma is currently lacking an effective treatment. There are no reliable predictors of metastatic recurrence, but extra-adrenal location and *SDHB* mutations have been associated with increased risk of metastasis and poor outcome^22^. However, adrenal tumours without SDHx mutations can also give rise to metastatic disease. Patients with hereditary forms of the disease are at risk of developing multiple primary tumors.

Aberrant DNA methylation has been identified as an important feature of human cancers. In normal tissue, intergenic CpG sites are typically methylated, while CpG-islands in promoter regions exhibit tissue-dependent methylation levels. In tumour tissue, intergenic regions are often hypomethylated, while certain promoter regions are hypermethylated, causing transcriptional changes^23^ by interfering with transcription factor binding and affecting the chromatin structure. Molecular subgroups characterized by different DNA methylation patterns have been described in several tumour types, and have been reported to be associated with differences in survival^24^,^25^,^26^. Moreover, DNA-methylation of specific genes may be used as biomarkers for response to chemotherapy, such as methylation of *MGMT* and response to alkylating agents^27^.

DNA methylation has been investigated in PPGLs. Early studies used sequencing- and PCR-based methods to investigate the methylation status of carefully selected candidate genes^28^,^29^, while more recent studies have used array-based technologies to investigate genome-wide methylation^30^,^31^. *SDHx*-mutated tumours have been found to exhibit a hypermethylator phenotype, due to allosteric inhibition of the demethylating TET family of enzymes by the build-up of succinate. Another subset of tumours has been found to exhibit global hypomethylation^31^. Recently, the methylation level of a number of CpG-sites was proposed as markers of malignancy in PPGL, independent of genetic background^30^, with high levels of methylation of a specific CpG-residue in the promoter of the *RDBP* gene pinpointed as a strong candidate biomarker for metastatic disease. In this paper we report the results from a global methylation analysis of 39 tumours from 35 patients and evaluate the set of proposed malignancy markers in this cohort.

## Results

### Hierarchical clustering

**Figure 1:**
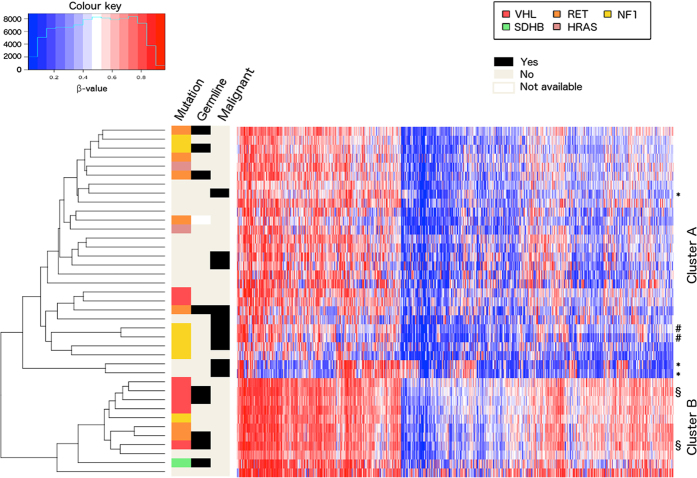
Heatmap and hierarchical clustering of tumour samples based on DNA methylation. Two distinct clusters are observed. The symbols *, #, and § denote samples originating from the same cases.

**Figure 2:**
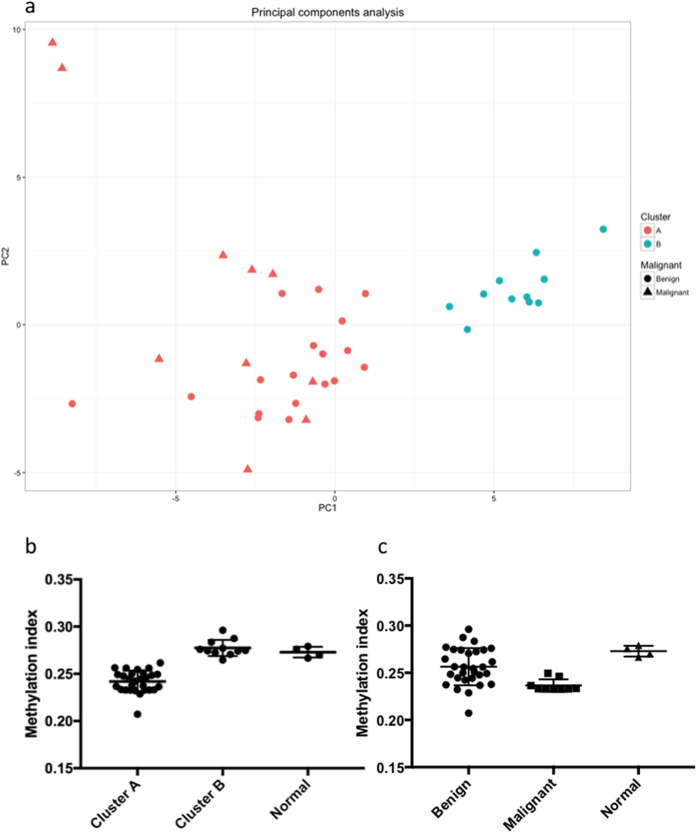
(**a**) Principal components analysis of the tumour samples shows the two clusters separated along the first principal component. (**b**) Methylation index of tumours in the two clusters, and of normal adrenal medulla. Tumours in cluster A have a lower global methylation level than tumours in cluster B and normal tissue. (**c**) Methylation index of benign tumours, malignant tumours and normal tissue.

Unsupervised hierarchical clustering with respect to the 10% most differentially methylated probes, as determined by standard deviation, revealed two clusters (Fig. 1, extended heatmap including normal samples in Supplemental Figure 1), from here on referred to as clusters A and B. Cluster A (n = 28) contained the majority of tumours with mutations in the *RET, NF1* and *HRAS* genes and most of those without known mutations, whereas cluster B (n = 11) contained the majority of the tumours with mutations in the *VHL* gene. Cluster A contained all malignant tumours in the cohort (n = 10), however, no difference in survival could be demonstrated (Supplemental Fig. 2). Principal Components Analysis (PCA) (Fig. 2a) was consistent with the results from the cluster analysis and separated the two clusters along the first principal component. Tumours in cluster A had an average methylation index (MI) of 0.242 while tumours in Cluster B had a higher average MI of 0.277 (p < 0.0001, Mann-Whitney test). Normal adrenal medulla had an average MI of 0.273, which was not statistically different from that of cluster B (p = 0.4, Kolmogorov-Smirnov), but significantly higher than that of Cluster A (p < 0.0001, Kolmogorov-Smirnov test, Fig. 2b). Malignant tumours were found to have a lower methylation index than benign tumours (0.237 vs 0.256, p = 0.0012 by the Mann-Whitney test, Fig. 2c).

### Differential methylation

**Figure 3:**
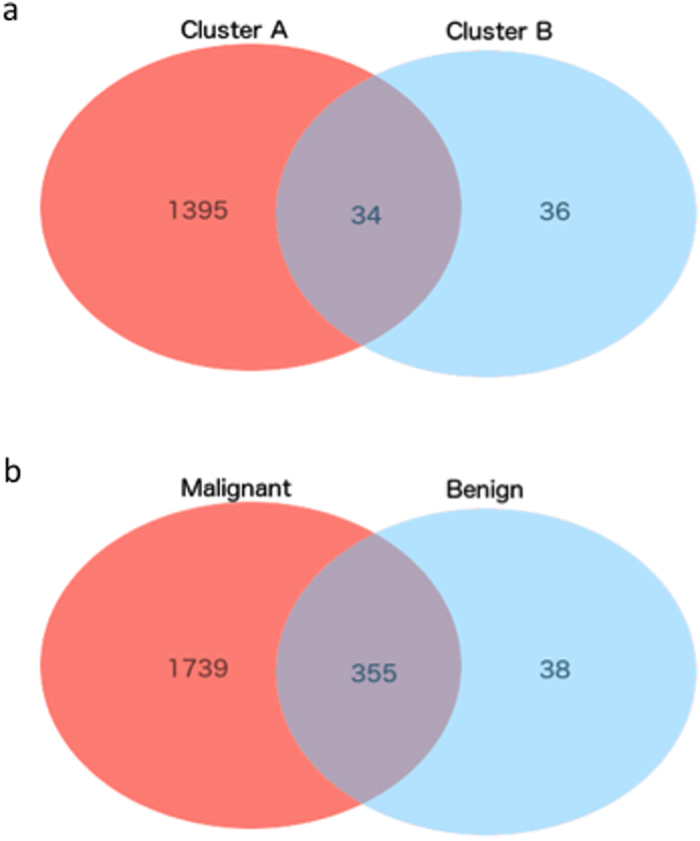
(**a**) Venn diagram of probes aberrantly methylated in the two clusters (compared to normal tissue). (**b**) Venn diagram of probes aberrantly methylated in benign and malignant tumours, compared to normal tissue.

Comparing methylation in pheochromocytomas to methylation in normal adrenal medulla, 642 probes were differentially methylated. Of these, 64 were more methylated and 578 were less methylated in tumours (Supplementary Table 1). Comparing the individual clusters to normal adrenal we found 1429 probes in Cluster A and 70 probes in Cluster B that were differentially methylated (Supplementary Tables 2 and 3), supporting the notion that tumours in cluster B are more similar to normal adrenal medulla in methylation. In total 34 of these probes were differentially methylated in both clusters (Fig. 3a). When the benign and the malignant tumours in cluster A were separately compared to normal medulla, 1254 probes were found differentially methylated in the benign tumours and 2904 were found differentially methylated in the malignant tumours (Supplementary Tables 4 and 5). Of these, 1006 were differentially methylated in both the benign and the malignant tumours.

In malignant tumours, 2094 probes were differentially methylated, while 393 probes were differentially methylated in benign tumours. In total 355 of these probes were found differentially methylated in both benign and malignant tumours compared to normal adrenal medulla (Fig. 3b). Notable genes found to be differentially methylated between tumour subgroups and normal tissue include *CTSZ*, a lysosomal enzyme which has found to be overexpressed in hepatocellular carcinoma^32^ and which was hypomethylated in tumours in Cluster A, as well as *SPRR3* which encodes the protein Esophagin which has been implicated in tumour cell proliferation and invasion in glioblastoma multiforme^33^, and has been shown to be upregulated in colorectal cancer^34^. Several cancer-testis antigens, including *CTAG2* and *CT45-2* were also found to be less methylated in tumour tissue than in normal tissue. Analysis of the expression of *CTAG2* and *CT45-*2 in the TCGA Pheochromocytoma and Paraganglioma RNA-Seq datasest revealed that *CTAG2* was expressed in 47% (89/187) of the samples. However, *CT45-*2 expression was not detected in any of the samples. Gene Ontology-enrichment analysis revealed genes involved in different immune response and cell differentiation pathways to be overrepresented in the list of genes differentially methylated in Cluster A (Supplementary Table 6). Whether or not this is related to a higher level of infiltrating immune cells in this subgroup should be further studied. No category was overrepresented among the genes differentially methylated in Cluster B.

A total of 275 probes were differentially methylated between benign and malignant tumours in our cohort (Supplementary Table 8). Of these, 14 were more methylated in malignant tumours and 261 were more methylated in benign tumours. Gene-ontology analysis of all the probes differentially methylated between benign and malignant tumours revealed overrepresentation of genes involved in immune response (Supplementary Table 9). A total of 119 probes were differentially methylated between benign and malignant tumours within Cluster A (Supplementary Table 10). Of these the majority (98 probes) had lower methylation levels in the malignant samples.

In the cases were sufficient samples were available tumours sharing a mutated gene were compared with normal medulla (Supplementary Tables 11–14). The highest number of differentially methylated probes were detected in tumours with *NF1*-mutations (1651 probes) followed by those without known mutations (1184 probes), those with *RET*-mutations (432 probes) and those with *VHL*-mutations (339 probes). The overlap of differentially methylated probes in these groups are shown as a Venn Diagram in Supplementary Figure 3. 100 probes were differentially methylated in all four groups and are listed in Supplementary Table 15.

### Correlation with Somatic Copy Number Aberrations

**Figure 4:**
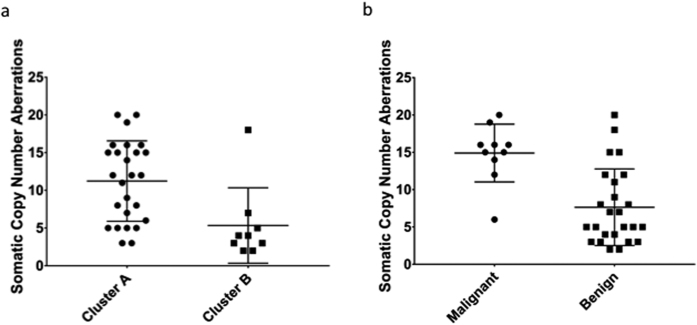
(**a**) Number of Somatic Copy Number Aberrations (SCNAs) in the two clusters. Tumours in cluster B carry more SCNAs than tumours in cluster A. (**b**) Number of SCNAs in malignant and benign tumours. Malignant tumours carry more SCNAs.

DNA hypomethylation is known to be associated with chromosomal instability. We compared the number of Somatic Copy-Number Aberrations (SCNAs) as determined by manual inspection of plotted SNP array data between cluster A (hypomethylated) and cluster B (non-hypomethylated) and found that tumours in cluster A had a greater number of chromosomal aberrations than tumours in cluster B (9.5 vs. 5.3, p = 0.0027 by the Mann-Whitney test, Fig. 4a). Similarly, malignant tumours carried a greater number of SCNAs than benign tumours (Fig. 4b 14.9 vs., 7.6, p = 0.0004 by the Mann-Whitney test). This remained true when only tumours in Cluster A were considered (14.9 vs. 8.9, p = 0.0198 by Kolmogorov-Smirnov). Linear regression of SCNA on methylation index revealed a significant negative correlation (R^2^ = 0.21, p = 0.0056, Supplementary Figure 4).

### Epigenetic heterogeneity

Three patients had multiple tumours included in the study. In two cases there was a high degree of concordance between different tumours from the same patient with 325 and 500 probes showing a difference in methylation of more than 0.2, respectively. However, in the third case, which included a primary tumour, a local recurrence, and a distant metastasis, a great discordance was found between the primary tumours and the two recurrences. The metastasis and the local recurrence had 2567 and 2677 probes with a methylation level differing more than 0.2 from the primary tumour. Moreover, they exhibited a methylation profile radically different from all other tumours in the cohort, clustering together in the outskirts of cluster A. The difference in MI between the primary tumour and the recurrences was negligible. Heatmaps for these three cases are given in Supplemental Figures 5–7. Tumour purity as estimated by ASCAT (19% contamination in the primary compared to 17% and 25% in the recurrent tumours) suggests that it is unlikely that these differences are explained by infiltrating non-tumoural cells.

### *PNMT* expression

**Figure 5:**
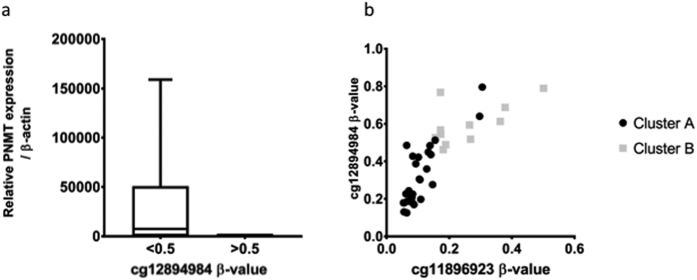
(**a**) PNMT mRNA expression is decreased in tumours with high levels of *PNMT* promoter methylation. (**b**) Tumours in cluster B tend to have higher levels of *PNMT* promoter methylation than tumours in cluster A.

Expression of PNMT, Phenylethanolamine-N-methyltransferase, the enzyme which converts norepinephrine to epinephrine is known to be regulated by promotor methylation^31^. In order to validate methylation analysis, *PNMT* expression was correlated with the observed methylation. A good concordance between the two was observed, with virtually abolished expression in tumours with high methylation level of the promoter. (Fig. 5). Linear regression revealed that the expression was significantly correlated to the probe cg12894984 (R^2^ = 0.14, p = 0.025) but not to the probe cg11896923. There was no significant difference in *PNMT* expression between the two clusters in our cohort; however, ANOVA revealed that the expression differed significantly between tumours with different mutation (p = 0.0024, Supplementary Figures 8 and 9).

### Evaluation of biomarkers

**Figure 6:**
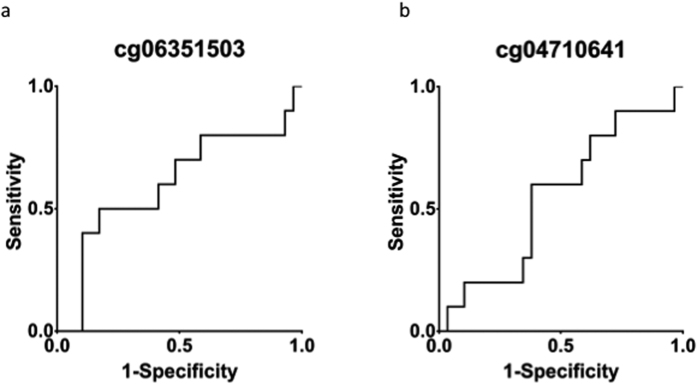
(**a**) Receiver Operating Characteristic (ROC) curve for a CpG probe associated with *RDBP* previously suggested as a biomarker of metastatic disease. (**b**) ROC curve for the other probes associated with *RDBP* on array.

A recent study proposed 52 probes as methylation-based markers of malignancy. We calculated Δβ- values for all these probes and were not able to replicate these findings (a |Δβ|- value of more than 0.2) in any of the probes. The probes associated with *RDBP* (cg04710641 and cg06351503) had Δβ- values of 0.007 and −0.098 between benign and malignant tumours, respectively. No significant differences in beta-values were found between benign and malignant tumours (p > 0.1 for both probes). In order to further assess the potential of the probes as predictors of metastasis Receiver Operating Characteristic (ROC) curves were generated (Fig. 6) and the Area Under the Receiver Operating Characteristic curve (AUROC) were calculated. For the two *RDBP* probes the AUROCs were 0.55 and 0.6, respectively.

Next we assessed the viability of the other 51 probes as predictors of metastasis. The AUROC was calculated for each of the probes. The mean AUROC of the probes was 0.60 (range 0.43–0.81). The best performance was by cg00626119 (*NTRK1*), which exhibited a Δβ of −0.15 in our cohort. A total of nine probes exhibited AUROCs > 0.7, which could indicate viability as biomarkers (Supplementary Table 16). All of these were less methylated in malignant tumours than in benign tumours.

## Discussion

We report here a global methylation analysis of thirty-nine PPGLs. We were able to reproduce many of the results previously published regarding methylation in PPGL. Namely, we found that tumours cluster according to mutated driver gene, and that tumours with mutations activating kinase pathways have global hypomethylation. The reason for this hypomethylation remains to be elucidated. Letouzé *et al*. identified three distinct clusters based on methylation data: M1–3. M1 contains tumours with *SDHx* mutations and hypermethylation, M2 *VHL*-mutated tumours, and M3 tumours with *NF1* and *RET* mutations and hypomethylation^31^. Roughly, our cluster A corresponds to the M3 cluster and cluster B to the M2 cluster. Unlike Letouzé *et al*., we were not able to identify a hypermethylator phenotype, possibly due to a paucity of *SDHx*-mutated tumours.

Within the hypomethylated cluster A we observe a greater number of chromosomal instability, as measured by number of chromosomal aberrations, in malignant tumours compared to benign tumours. This represents a possible mechanism through which aberrant DNA methylation might affect diverse disease progression course in pheochromocytoma in addition to altering gene expression.

Genomic hypomethylation has been found to be causally linked to chromosomal instability. In the hypomethylated cluster A we observe increased chromosomal instability as measured by number of chromosomal aberrations. This represents a possible mechanism through which aberrant DNA methylation can affect disease progression in pheochromocytoma in addition to altering gene expression. Indeed, malignant tumours were found to harbour a greater number of copy number aberrations than benign tumours, even within Cluster A, supporting the idea that hypomethylation can contribute to malignant progression.

In three cases we were able to study DNA methylation in multiple (2–3) tumours from the same individual. While this number is too small to draw any general conclusions, we observe a wide range in the number of differentially methylated probes between tumours from the same patient. The extent and impact of epigenetic heterogeneity in pheochromocytoma is an interesting topic for future studies. The importance of genetic heterogeneity in cancer is well established and has recently been studied in pheochromocytoma. Epigenetic heterogeneity, on the contrary, remains largely uninvestigated. Pioneering studies have reported that epigenetic heterogeneity is abundant and potentially plays an important role^35^.

The malignancy biomarkers proposed by other studies could not be reproduced in our cohort. In particular, *RDBP*, which has been suggested to be hypermethylated in metastasizing PPGL regardless of mutational status, was not hypermethylated in the malignant tumours of our cohort. A likely explanation for this is the differences in composition that exist between our cohort and the cohorts investigated in other studies. Notably, other studies investigating DNA methylation in PPGL have had an abundance of *SDHx*-mutated tumours, while only one such tumour is part of the cohort studied here. *SDHx*- and especially *SDHB*-mutated tumours have high metastatic potential, while also exhibiting a hypermethylator phenotype. This could explain why hypermethylation of *RDBP* is found to be associated with malignancy in other cohorts, but not in our cohort, where the probes for *RDBP* performed marginally better than chance as predictors of metastatic disease. The other probes that were proposed were also evaluated. While a number of them showed promise as markers of malignancy, closer examination revealed that they showed different methylation pattern in our cohort than in the cohorts in which they were discovered; in the present study they were less methylated in malignant tumours than in benign tumours, while the opposite relationship has previously been reported. This highlights the difficulties inherent in designing malignancy markers for a tumour type as diverse as PPGL. Since the different groups of PPGL exhibit striking differences in genetic background and tumorigenic pathways, reliable malignancy markers valid for all PPGL remain undiscovered. Future studies of large cohorts, incorporating multiple types of omics data may enable more reliable risk stratification of cases.

### Limitations of this study

A potential pitfall of this study is the presence of normal cell contamination (e.g. vascular cells, immune cells and fibroblasts) in the tumour samples. We have previously shown that tumour cell purity differs between mutational subgroups^36^. This may contribute to the differences noted between different subgroups by the present study and others. Tumour purity estimated by ASCAT (as reported previously) did not differ significantly between the methylation clusters in this study (Supplementary Table 17). However, an influence of infiltrating stromal cells on the results reported in this study cannot be excluded.

PPGLs are rare. This study included 39 tumours and 4 normal adrenal medullas. This is the only independent large cohort to date investigating and corroborating the findings by de Cubas *et al*.^30^. Even though we present statistically significant results and have employed several statistical methods to ensure solidity, much larger cohort or a meta analysis based on several reports are needed to confirm clinical significance of these findings.

In conclusion, previously discovered molecular subgroups of pheochromocytoma defined by DNA methylation were validated while proposed markers of malignancy could not be validated in our cohort.

## Materials and Methods

### Cohort

Thirty-nine histologically verified PPGLs (38 pheochromocytomas and 1 paraganglioma) from 35 individuals, as well as 4 normal adrenal medullae were included in this study. The cohort characteristics are outlined in Table 1. The mean age at diagnosis was 50.5 years (range 17–75 years) and the gender ratio (female:male) was 1.69. Two patients had MEN2A, two had MEN2B, an additional three patients had von Hippel-Lindau syndrome, and one had Neurofibromatosis type 1 while one patient had paraganglioma syndrome type 4. One patient with von Hippel-Lindau disease had bilateral disease with both tumours included in the cohort. The tumours had been previously studied for mutations in known driver genes and using high-density SNP array (data available for 35 of the tumours)^36^,^37^. Tumour purity was estimated from SNP array data using ASCAT v. 2.1^38^ as implemented in Nexus Copy-number Variation 7.5 (Biodiscovery Inc., CA).

### DNA extraction and methylation array analysis

All tissues were snap-frozen in liquid nitrogen perioperatively and were stored at −70 °C. The tumours were cryosectioned, and DNA was extracted using Qiagen DNA Blood and Tissue kit (QIAGEN, Redwood city) in accordance with the manufacturer’s instructions. The extracted DNA was treated using the QIAGEN EpiTect Bisulfite kit and fragmented. The final product was analyzed using the Illumina Infinium HumanMethylation27 (Illumina Inc. San Diego) microarray at the Bioinformatics and Expression Analysis Core Facility at the Karolinska Institute. The array has probes for 27,578 CpG-sites, located in the promoters of 14,497 protein-coding genes spread across the genome.

### RNA extraction and RT-PCR

Similarly to DNA, RNA was extracted from cryosections of fresh frozen tumour tissue using the Qiagen RNEasy Mini- and Midi-kits (QIAGEN, Redwood city). Extracted RNA was converted to cDNA using RevertAid First strand cDNA Synthesis kit (ThermoFisher scientific, MA,USA). RT-PCR was performed using SYBR Green on the Bio-Rad CFX96 Real-Time System (Bio-Rad, Hercules, California). Beta-actin was used as a reference gene, and the 2^−ΔΔCT^ method was used for calculations. Primer sequences are available upon request.

### Analysis of TCGA PCPG Expression data

Upper Quantile normalized FPKM (FPKM-UQ) gene expression data for the TCGA Pheochromocytoma&Paraganglioma cohort were obtained from the Genomics Data Commons portal (https://gdc-portal.nci.nih.gov). Expression values for genes of interest were extracted and summarized using custom python scripts. Genes with an FPKM-value > 1 in a given sample were considered to be expressed.

### Further data processing and statistics

Generated microarray images were imported into Illumina GenomeStudio (Illumina Inc., San Diego, US) where beta-values were calculated and exported. Differential Methylation analysis was performed in GenomeStudio using the Illumina Custom Error Model. Probes with |DiffScores| > 30 together with |Δβ| > 0.2 were considered differentially methylated. Further analysis was performed using the R statistical environment and GraphPad Prism 7 (GraphPad Software Inc, CA). Hierarchical clustering of the samples was performed after removal of probes on the sex chromosomes and of probes with missing values using the 10% most variably methylated probes, as determined by standard deviation, with the Euclidean distance as the distance metric. Principal Components Analysis (PCA) was performed using the R function *prcomp*. The Mann-Whitney U test and Fisher’s exact test were used. When sample-sizes were small, the two-sample Kolmogorov-Smirnov test was used in place of Mann-Whitney U. The Benjamini-Hochberg^39^ method was used to correct for multiple hypothesis testing when required. The Benjamini-Hochberg procedure is an alternative approach to monitor false discovery rates. Briefly, individual variables p-values are sorted in ascending order and are designated a numerical rank. Numerical rank divided by total number of variables (p-values) multiplied by the desired false discovery rate, gives the Benjamini-Hochberg critical value. The largest p-value that is numerical larger than its Benjamini-Hochberg critical value as well as all p-values smaller than it are then considered as significant, even those larger than their Benjamini-Hochberg critical value^40^,^41^. This procedure is fully implemented in Illumina GenomeStudio software. Two-tailed *p*-values of less than 0.05 were considered to be statistically significant. The CRAN package “pROC” was used to generate Receiver Operating Characteristics (ROCs) for the purpose of evaluating specific probes as predictors of metastatic disease. The Venn Diagram in Supplemental Figure 3 was generated using http://bioinformatics.psb.ugent.be/cgi-bin/liste/Venn/calculate_venn.htpl.

### Gene ontology analysis

Gene ontology analysis was performed using the PANTHER Overrepresentation test (http://www.pantherdb.org, release 20160715) with the PANTHER GO Ontology dataset. Bonferroni-correction was used to correct for multiple testing and only results with corrected p-values < 0.05 were considered significant.

### Ethics

All patients had provided written informed consent for the preservation and subsequent analysis of tissues. Ethical approval was obtained from the regional ethics committee in Uppsala. Experiments were conducted in accordance with relevant guidelines and regulations.

## Additional Information

**How to cite this article:** Backman, S. *et al*. Global DNA Methylation Analysis Identifies Two Discrete clusters of Pheochromocytoma with Distinct Genomic and Genetic Alterations. *Sci. Rep.* **7**, 44943; doi: 10.1038/srep44943 (2017).

**Publisher's note:** Springer Nature remains neutral with regard to jurisdictional claims in published maps and institutional affiliations.

## Supplementary Material

Supplementary Information

## Figures and Tables

**Table 1:** Cohort characteristics.

Tumour characteristics	Complete cohort	Cluster A	Cluster B
PCC/PGL	38/1	28/0	10/1
Benign/Malignant	29/10	18/10	11/0
Mutation: NF1/RET/HRAS/VHL/SDHB/-	7/7/2/7/1/15	6/5/2/2/0/13	1/2/0/5/1/2
**Patient characteristics**			
Male/Female	13/22	12/13	1/9
Age at diagnosis	50.5 (17–75)	53.2 (17–75)	43.3 (27–69)
Metastatic disease	7	7	0
Syndrome: VHL/NF1/MEN2/PGL4	3/1/4/1	0/1/3/0	3/0/1/1
