# Supplementary material for: Global DNA Methylation Analysis Identifies Two Discrete clusters of Pheochromocytoma with Distinct Genomic and Genetic Alterations
Source: Sci Rep. 2017 Mar 22;7:44943. doi: 10.1038/srep44943 (PMC5361146; doi:10.1038/srep44943)

Supplementary materials

Global DNA Methylation Analysis Identifies Two Discrete clusters of Pheochromocytoma with Distinct Genomic and Genetic Alterations

Samuel Backman[1], Rajani Maharjan[1], Alberto Falk-Delgado, Joakim Crona[1], Kenko Cupisti[2], Peter Stålberg[1], Per Hellman[1], Peyman Björklund[1]

[1] Department of Surgical Sciences, Uppsala University, Uppsala, Sweden
[2] Department of Surgery, Marien-Hospital, Euskirchen, Germany

Supplementary table 1 – Probes differentially methylated between tumours and normal tissue

TargetID	Normal.AVG_Beta	Pheos.AVG_Beta	Pheos.DiffScore	Pheos.Delta Beta	SYMBOL
cg13021192	0.6005161	0.1794109	-349.1551	-0.4211051	CTSZ
cg16179125	0.6432001	0.2326134	-349.1551	-0.4105867	CTSZ
cg25856811	0.7179568	0.3252473	-349.1551	-0.3927095	SPRR3
cg26164184	0.8149152	0.43574	-349.1551	-0.3791752	FCN2
cg14204735	0.4661456	0.08934068	-349.1551	-0.3768049	CYB561
cg27619475	0.5564861	0.1947175	-349.1551	-0.3617686	SLC16A5
cg04144768	0.6791029	0.3180636	-349.1551	-0.3610393	DDC
cg01335367	0.5233509	0.164579	-349.1551	-0.3587719	C12orf34
cg13726507	0.8284182	0.470094	-349.1551	-0.3583242	CTAG2
cg06536578	0.6501456	0.2957369	-349.1551	-0.3544087	JPH4
cg26927807	0.6936526	0.3424899	-349.1551	-0.3511627	BTBD2
cg22374142	0.4976765	0.1471041	-349.1551	-0.3505723	HSF4
cg14992108	0.7202581	0.3699769	-349.1551	-0.3502811	SNTB1
cg26672426	0.626225	0.2933851	-349.1551	-0.3328399	PTGES
cg20154346	0.6379861	0.3053585	-349.1551	-0.3326276	RAI2
cg20334738	0.6488818	0.317371	-349.1551	-0.3315108	MAB21L2
cg08450982	0.5887374	0.2600167	-349.1551	-0.3287207	NUMBL
cg18149207	0.7333511	0.4051361	-349.1551	-0.328215	RORC
cg06123346	0.6199951	0.2936695	-349.1551	-0.3263255	ATP4A
cg25221254	0.7264441	0.4001243	-349.1551	-0.3263199	ASAH3
cg08573687	0.6807172	0.3556303	-349.1551	-0.3250869	TH
cg25119415	0.687941	0.363129	-349.1551	-0.3248119	MNDA
cg01103730	0.6973703	0.3741394	-349.1551	-0.3232309	IL20
cg21457804	0.7505146	0.4276943	-349.1551	-0.3228202	CT45-2
cg24024214	0.8215494	0.4989479	-349.1551	-0.3226015	BTNL8
cg22575540	0.7558594	0.4382493	-349.1551	-0.31761	TRIM54
cg23815000	0.7271953	0.4103397	-349.1551	-0.3168556	LCN1
cg15503752	0.7297292	0.4138848	-349.1551	-0.3158444	ST6GALNAC1
cg06639544	0.7385153	0.422695	-349.1551	-0.3158202	OR7A5
cg12489960	0.6688474	0.3554831	-349.1551	-0.3133644	SGCB
cg21948655	0.7988736	0.4869205	-349.1551	-0.3119531	SMCP
cg14706739	0.6925761	0.380715	-349.1551	-0.3118611	EPB49
cg15652212	0.6933754	0.3865155	-349.1551	-0.3068599	FLJ90586
cg18780284	0.6813936	0.3752761	-349.1551	-0.3061175	SPRR1B
cg05444024	0.8518489	0.549286	-349.1551	-0.3025629	FUT6
cg07824742	0.7590712	0.458432	-349.1551	-0.3006391	DBH
cg03602500	0.7306974	0.432349	-349.1551	-0.2983484	FLJ00060
cg00698688	0.8040674	0.5080183	-349.1551	-0.2960491	SULT2B1
cg05348870	0.7693964	0.4755233	-349.1551	-0.2938731	TNFSF14
cg14256699	0.8603887	0.5675716	-349.1551	-0.2928171	SOST

cg08684473	0.7483833	0.4572159	-349.1551	-0.2911673	LILRB5
cg15711744	0.7625234	0.4735308	-349.1551	-0.2889926	ANP32D
cg10758292	0.7698587	0.4819526	-349.1551	-0.2879061	DEFA1
cg04138756	0.7806306	0.4970625	-349.1551	-0.2835681	SPRR3
cg17357062	0.8257549	0.542263	-349.1551	-0.283492	FCN1
cg05766474	0.818482	0.5420715	-349.1551	-0.2764105	CCL16
cg26149550	0.7885463	0.5170676	-349.1551	-0.2714787	KLK15
cg14333565	0.7914666	0.5216023	-349.1551	-0.2698643	NRTN
cg21686987	0.7764781	0.5087843	-349.1551	-0.2676938	CTRB1
cg25813714	0.8435937	0.583806	-349.1551	-0.2597876	CYP4F12
cg24992780	0.7986712	0.5393614	-349.1551	-0.2593099	OR7C1
cg14182690	0.7908964	0.5353193	-349.1551	-0.2555771	RUNX3
cg24407065	0.8032658	0.5506953	-349.1551	-0.2525705	BLZF1
cg08453096	0.8258924	0.5820401	-349.1551	-0.2438523	ABCG5
cg08475088	0.8484987	0.6055449	-349.1551	-0.2429538	NALP9
cg18138484	0.8258247	0.5832363	-349.1551	-0.2425884	CABP2
cg19561774	0.8592458	0.6169297	-349.1551	-0.2423161	SLC22A2
cg00644033	0.9175664	0.6757333	-349.1551	-0.2418332	MUC3B
cg21450627	0.8156587	0.5749301	-349.1551	-0.2407286	PSD4
cg04132607	0.8591244	0.6208734	-349.1551	-0.238251	GATA5
cg04816348	0.8662676	0.6303735	-349.1551	-0.235894	CLEC4G
cg03534410	0.8616629	0.6293499	-349.1551	-0.232313	TMEM40
cg23514672	0.8726774	0.6411138	-349.1551	-0.2315637	FLJ32871
cg08314660	0.8477555	0.6172999	-349.1551	-0.2304556	PKP3
cg17264470	0.8543522	0.6245466	-349.1551	-0.2298056	FGF21
cg10213812	0.8630112	0.6346682	-349.1551	-0.2283431	FOXN1
cg00520135	0.8555187	0.6322072	-349.1551	-0.2233115	TPM1
cg27341860	0.891391	0.6704241	-349.1551	-0.2209669	OR2L13
cg26473272	0.867389	0.6469713	-349.1551	-0.2204176	SYT8
cg11113534	0.8559733	0.6373187	-349.1551	-0.2186546	C20orf70
cg23753610	0.9301904	0.716409	-349.1551	-0.2137814	DNAHL1
cg07595943	0.9167925	0.7049012	-349.1551	-0.2118913	LOC161931
cg12582965	0.8588696	0.6477143	-349.1551	-0.2111553	ATP10A
cg11830061	0.9157187	0.7105842	-349.1551	-0.2051345	INSL6
cg17741572	0.7970396	0.546249	-348.1271	-0.2507906	CFB
cg08970446	0.8225887	0.5878221	-347.7729	-0.2347666	SLC1A7
cg24694549	0.6403007	0.3240729	-346.1951	-0.3162278	GRIP1
cg13608094	0.8641294	0.6591082	-346.0796	-0.2050212	CCND1
cg12815142	0.6761258	0.3708805	-345.4565	-0.3052454	SPAG7
cg01515887	0.7287439	0.446232	-339.4385	-0.2825119	SAA2
cg26292028	0.6899872	0.393079	-337.0683	-0.2969083	FLJ37587
cg21372914	0.7140282	0.4267384	-336.0137	-0.2872898	CLEC4M
cg27377450	0.8068079	0.5663286	-335.8326	-0.2404794	ARHGEF18
cg10805676	0.7133251	0.4260627	-335.2991	-0.2872624	MRPL28
cg12513481	0.5602626	0.2342089	-335.2858	-0.3260536	SCAP1

cg15516226	0.7981389	0.5528566	-334.8934	-0.2452822	BTNL9
cg21277505	0.8291306	0.6038567	-332.6728	-0.225274	LOC284361
cg18129786	0.8098671	0.5725743	-332.2004	-0.2372928	ZNF445
cg06244417	0.774786	0.5175284	-332.0867	-0.2572576	FCN1
cg06832950	0.7760125	0.5197286	-331.2612	-0.2562839	SPG3A
cg17907567	0.6217936	0.308522	-329.6122	-0.3132716	HAMP
cg10062065	0.6583511	0.3547563	-328.9843	-0.3035948	APEG1
cg20373326	0.6809996	0.3848795	-328.1474	-0.2961201	HSD17B2
cg07792737	0.8123163	0.5794969	-323.9589	-0.2328194	NPIP
cg19728223	0.5383787	0.2158357	-323.65	-0.322543	KCNQ1
cg19728577	0.7729563	0.5182743	-322.977	-0.2546819	GUCA2B
cg02442161	0.7531271	0.4888845	-322.0854	-0.2642426	PI3
cg20544605	0.6577012	0.3581229	-319.5542	-0.2995783	SORBS2
cg08626653	0.7109996	0.4301368	-318.0329	-0.2808628	FLJ37538
cg25203980	0.8098795	0.5778862	-317.6818	-0.2319933	CENTB5
cg08424423	0.8008775	0.564241	-315.9596	-0.2366365	CDSN
cg06101324	0.4954236	0.1754946	-315.5491	-0.3199289	SPRR1A
cg24670715	0.4996431	0.180294	-314.2922	-0.319349	ANGPT2
cg12456510	0.6795629	0.3895861	-313.1729	-0.2899767	TFF2
cg10677144	0.7112477	0.4331146	-311.9918	-0.2781331	MYOM1
cg14696820	0.6009493	0.2929877	-309.1959	-0.3079616	LCE1A
cg06236276	0.7581028	0.5040116	-303.0293	-0.2540912	SLC22A2
cg13656062	0.7520977	0.4953682	-302.581	-0.2567295	CYP4F2
cg03221619	0.5532917	0.2424317	-301.7823	-0.31086	FCER2
cg14150666	0.8354299	0.6254576	-300.6789	-0.2099723	IL8RB
cg10990993	0.5985331	0.2942526	-300.5915	-0.3042805	MLH1
cg16990174	0.547488	0.2368036	-300.4214	-0.3106844	RYBP
cg22013966	0.8409851	0.6347732	-300.2852	-0.2062119	SERPINA13
cg07997737	0.5722926	0.2644956	-299.8167	-0.307797	NRTN
cg22784047	0.6865979	0.405918	-297.7694	-0.2806799	MVP
cg18508525	0.8117525	0.5885997	-297.0598	-0.2231528	CD36
cg08471713	0.8352556	0.627315	-294.773	-0.2079406	MEOX1
cg12619162	0.7686013	0.523487	-293.77	-0.2451143	FXYD4
cg09701102	0.7531219	0.5009155	-293.0842	-0.2522064	NDUFV1
cg16772207	0.620232	0.3250083	-290.4832	-0.2952237	MYT1
cg19954000	0.5019863	0.1944584	-290.2011	-0.3075279	FGF1
cg15928132	0.607567	0.3105168	-289.1083	-0.2970501	CCKAR
cg04505023	0.6997429	0.4278599	-288.4169	-0.271883	SPRR1A
cg00334507	0.6243029	0.3310087	-288.328	-0.2932942	MVP
cg13521229	0.5531197	0.250439	-285.2996	-0.3026807	JOSD2
cg16122592	0.6405548	0.3536003	-282.9391	-0.2869545	MAGEB6
cg16462075	0.7248219	0.464963	-282.7489	-0.259859	MUC3B
cg22294577	0.8043756	0.5826795	-282.3722	-0.2216961	SLC26A3
cg14162076	0.8297534	0.6237301	-280.1606	-0.2060233	CLEC4D
cg14894144	0.479454	0.1783413	-278.2083	-0.3011126	LAMA3

cg27285599	0.7615805	0.5201346	-277.0762	-0.2414458	FLJ13841
cg22264436	0.7468928	0.498904	-276.8443	-0.2479888	SOST
cg26422060	0.7860416	0.5569527	-276.2981	-0.229089	TBX10
cg19949550	0.7388231	0.4879559	-275.5382	-0.2508672	ASB2
cg16175263	0.5629296	0.2663313	-275.2727	-0.2965983	TNFRSF10C
cg10569414	0.6894798	0.4214381	-272.7224	-0.2680417	C21orf121
cg04323365	0.744164	0.4970019	-272.3641	-0.2471622	GJB1
cg17820828	0.7245404	0.4699518	-270.9458	-0.2545886	KCNQ1
cg01827098	0.7951075	0.5730559	-270.7866	-0.2220516	GIMAP7
cg07126559	0.7593099	0.5200485	-269.7197	-0.2392614	SGCG
cg14845091	0.624664	0.3409131	-269.2831	-0.283751	ADPRHL1
cg05485062	0.7768545	0.5462084	-269.0728	-0.2306461	SERPINA12
cg19807685	0.6756111	0.4050766	-268.9254	-0.2705345	HSD17B2
cg19917856	0.5128558	0.2166341	-268.4885	-0.2962217	LOC342897
cg23776892	0.7766726	0.5462099	-268.4885	-0.2304627	MAGEA1
cg10275770	0.4400549	0.1471992	-267.3671	-0.2928557	ICAM2
cg06220755	0.618116	0.3352206	-265.0085	-0.2828954	RAI2
cg04719766	0.7839289	0.55888	-264.2481	-0.2250489	KCNQ1
cg02067021	0.6885798	0.424656	-263.694	-0.2639238	DNAJC5B
cg05671018	0.679382	0.4127809	-263.2805	-0.2666011	LYSMD2
cg22190114	0.7153814	0.4610602	-263.0235	-0.2543212	NALP8
cg10604646	0.5371482	0.2448906	-262.9251	-0.2922576	RGS5
cg21065959	0.6587805	0.3865619	-262.7348	-0.2722186	LCE1E
cg06436504	0.6696407	0.4006012	-262.4195	-0.2690395	DOC1
cg24363955	0.7855654	0.5621564	-262.3993	-0.223409	FLJ14054
cg24027679	0.7759639	0.5478842	-262.2426	-0.2280797	SLC2A7
cg09027725	0.6293691	0.3505451	-261.7391	-0.2788239	COX4I2
cg14238120	0.7126997	0.4580835	-261.649	-0.2546162	ELA3A
cg27394486	0.8008213	0.5857739	-261.3552	-0.2150474	C15orf2
cg25214346	0.654166	0.3814659	-261.3472	-0.2727002	NR1I3
cg11158374	0.7050698	0.4481361	-260.823	-0.2569337	TFF2
cg19368582	0.6470323	0.3729939	-260.4474	-0.2740384	MMRN2
cg23412777	0.6425221	0.3680225	-259.1906	-0.2744996	PYGO1
cg01718139	0.7657939	0.5342838	-259.1583	-0.2315102	UNQ3033
cg17240454	0.7999614	0.5855871	-258.6842	-0.2143743	SPDEF
cg05440289	0.7547014	0.5186027	-258.2331	-0.2360988	IVL
cg13019092	0.7327785	0.4881585	-256.752	-0.24462	PDZK1
cg25477904	0.8078945	0.598575	-256.7494	-0.2093195	PSG1
cg02351381	0.4641945	0.1754267	-256.2549	-0.2887678	C12orf34
cg05556202	0.7580193	0.5242994	-256.2549	-0.2337198	TM4SF19
cg13204181	0.6311664	0.3564085	-254.6987	-0.2747579	GH1
cg27235662	0.7571304	0.5237268	-254.6812	-0.2334036	CLDN16
cg13578652	0.6351296	0.3616584	-253.9177	-0.2734712	UBASH3A
cg09299388	0.6970874	0.4409614	-253.6384	-0.256126	PGK2
cg24824840	0.790109	0.5729057	-253.3816	-0.2172034	SHANK1

cg16626670	0.6462061	0.3759372	-252.741	-0.2702689	CLEC4G
cg26946769	0.7810324	0.5601134	-251.5734	-0.220919	MAPK4
cg02844051	0.7362346	0.4955091	-251.469	-0.2407255	ZD52F10
cg25781162	0.7540183	0.5211482	-250.5571	-0.2328701	ABCG5
cg01015871	0.6167418	0.341368	-250.2877	-0.2753739	MT4
cg11739626	0.7003604	0.4473656	-249.5756	-0.2529947	AKT1S1
cg15905124	0.7711913	0.5466432	-249.2842	-0.2245481	MGC13034
cg15780361	0.5513908	0.2680345	-248.4785	-0.2833562	ALS2CR11
cg01643624	0.726625	0.484568	-246.4133	-0.2420569	C11orf16
cg11884243	0.7931302	0.5804961	-246.3671	-0.212634	FCN2
cg18242139	0.7134107	0.4666366	-246.1152	-0.2467741	ELAVL4
cg16986846	0.5924717	0.3168318	-243.0499	-0.2756399	SCGB2A1
cg24490338	0.7198963	0.4770663	-242.8263	-0.24283	TPM3
cg08341924	0.7583263	0.5310573	-242.5137	-0.227269	TGM1
cg00321478	0.6564969	0.3942619	-242.2571	-0.262235	CRB1
cg24735489	0.6244857	0.3550967	-241.9299	-0.2693891	CDSN
cg08728865	0.7916111	0.5801899	-241.8438	-0.2114213	NALP7
cg14287742	0.7497964	0.5192221	-241.8186	-0.2305743	BLZF1
cg19233472	0.7223547	0.4814174	-240.8641	-0.2409373	FOXI1
cg26063872	0.6424041	0.3777391	-240.3909	-0.264665	DEFB123
cg17829936	0.6854419	0.4323488	-240.3209	-0.2530931	TAAR5
cg04431776	0.802137	0.5972912	-239.0161	-0.2048457	GAGE2
cg03364781	0.6811622	0.4278127	-238.3162	-0.2533495	ALPK1
cg11719283	0.6585968	0.3991438	-238.0435	-0.2594531	ZNF574
cg09414535	0.6036983	0.3330359	-237.275	-0.2706623	GRIP1
cg19042947	0.6387034	0.3754127	-236.2824	-0.2632907	SERPINA4
cg25915982	0.7758661	0.5597733	-235.451	-0.2160928	GRB10
cg09467501	0.3721131	0.1064787	-235.0825	-0.2656344	PYY
cg24901474	0.5516204	0.2758371	-234.8556	-0.2757833	RGS5
cg19859270	0.6829456	0.4320296	-234.7059	-0.250916	GPR15
cg06501790	0.6680466	0.4130271	-234.4604	-0.2550195	SLC34A1
cg08292050	0.7579495	0.534547	-234.0612	-0.2234026	SOCS4
cg24750391	0.4890362	0.2116702	-234.012	-0.277366	PON3
cg04953015	0.7136792	0.4731544	-233.8791	-0.2405248	CHRNA2
cg12334759	0.6979702	0.4526268	-232.9682	-0.2453434	C19orf19
cg05215575	0.5650438	0.2916764	-232.8409	-0.2733674	FLJ25410
cg04711324	0.5650914	0.292681	-231.1205	-0.2724104	RIT2
cg25033144	0.7326362	0.5007813	-230.3741	-0.2318549	FLJ00060
cg24697329	0.7395797	0.511025	-229.1472	-0.2285548	ARHGEF4
cg02601403	0.7693781	0.5535097	-228.7024	-0.2158684	TBC1D3C
cg06351503	0.6866283	0.4401737	-228.3482	-0.2464547	RDBP
cg07525077	0.5739071	0.3042197	-228.0558	-0.2696874	RNASE3
cg18729973	0.7458268	0.5205106	-227.6294	-0.2253162	TFF1
cg19712821	0.5667985	0.2967039	-227.3898	-0.2700946	KSP37
cg22189286	0.6909527	0.4466038	-226.854	-0.2443489	HSPB8

cg25762706	0.5692218	0.2998619	-226.5592	-0.2693599	STMN4
cg07651914	0.5732188	0.3048552	-225.5858	-0.2683637	CLDN15
cg10127415	0.5262495	0.2543201	-225.3772	-0.2719294	MAGEB6
cg10575735	0.6007839	0.3363411	-225.3472	-0.2644428	SSX4
cg01657380	0.6698722	0.4204321	-225.0013	-0.2494401	NPFF
cg02130905	0.6067913	0.3437373	-224.6438	-0.263054	STMN4
cg18204685	0.686089	0.4416396	-224.3099	-0.2444493	BTD
cg03458191	0.7495673	0.5274154	-224.3099	-0.2221519	SAA1
cg09736922	0.7911158	0.5875998	-223.8314	-0.203516	THPO
cg25778166	0.7248582	0.4938717	-222.991	-0.2309865	FMO3
cg02833725	0.6523967	0.3996254	-222.941	-0.2527713	ISG20L2
cg19845843	0.7100663	0.4739568	-222.9208	-0.2361095	CXorf20
cg15210427	0.5148324	0.2442842	-222.3961	-0.2705482	CST9L
cg01484156	0.5501247	0.2814487	-222.3258	-0.268676	NCALD
cg24607398	0.5218193	0.2516253	-222.1731	-0.270194	MLH1
cg19982860	0.6294757	0.3721965	-221.9413	-0.2572792	IFNA21
cg00594952	0.564952	0.2980545	-221.5884	-0.2668974	RIMS3
cg24355048	0.6374406	0.3823592	-221.0214	-0.2550814	CTSG
cg08448751	0.6614423	0.4120989	-220.8683	-0.2493433	SEMA3G
cg03609102	0.5712318	0.3061907	-219.5488	-0.2650411	MUC5B
cg07441143	0.6896355	0.4493204	-218.635	-0.2403151	SLURP1
cg23829949	0.5814235	0.3183523	-218.2275	-0.2630712	ZNF238
cg06437862	0.7601305	0.5455602	-217.8354	-0.2145703	TUBA2
cg05670596	0.4878161	0.2199822	-217.6406	-0.2678339	CCRL2
cg15743985	0.704594	0.4694955	-217.6406	-0.2350985	CD22
cg01568736	0.7396729	0.5170903	-217.4513	-0.2225826	SERPINB7
cg27442349	0.6332046	0.3792835	-217.4011	-0.2539211	NFKBIB
cg27090216	0.3580904	0.1053671	-216.8201	-0.2527233	TNFRSF10C
cg21495715	0.7150798	0.4840035	-216.6504	-0.2310763	SLC5A10
cg06811800	0.6753246	0.4320871	-216.4894	-0.2432374	ATP4B
cg04450876	0.7845926	0.5816689	-216.0721	-0.2029237	FAM112B
cg23749046	0.7882785	0.5871783	-215.8398	-0.2011002	GPR61
cg26583078	0.6611041	0.4145779	-215.6904	-0.2465262	SORBS2
cg13180098	0.641979	0.3911961	-215.2183	-0.2507829	RHO
cg17687962	0.7372782	0.5153332	-214.4634	-0.221945	KLK3
cg26264314	0.7494335	0.5322686	-214.3382	-0.2171649	NALP5
cg23881725	0.4296838	0.1673783	-214.1737	-0.2623055	DLEC1
cg10052840	0.4951251	0.229511	-213.8145	-0.265614	SEMA6B
cg15626350	0.467462	0.2027914	-213.4234	-0.2646706	ESR1
cg18920397	0.7027093	0.4693266	-213.3722	-0.2333827	LY9
cg22021786	0.7114305	0.48091	-213.3426	-0.2305205	WFDC8
cg23873703	0.7797037	0.5760549	-212.968	-0.2036488	KCNAB1
cg00941549	0.6680831	0.4252146	-212.3897	-0.2428685	AKAP4
cg25372195	0.6580823	0.4128451	-212.0971	-0.2452372	DCD
cg22510822	0.6442158	0.3958468	-211.8973	-0.2483689	OR1E2

cg11599505	0.7650282	0.5557978	-211.3154	-0.2092304	C20orf102
cg18979223	0.6606013	0.4164916	-211.2176	-0.2441097	CDKN2B
cg23444894	0.7427903	0.5251839	-210.1671	-0.2176065	UNQ5810
cg20551517	0.7594296	0.5484797	-210.1052	-0.2109499	GIP
cg27020690	0.3531938	0.1053756	-210.011	-0.2478183	TERC
cg08093398	0.6519684	0.4065903	-209.805	-0.2453781	PSF1
cg04810997	0.6714357	0.4314234	-208.9112	-0.2400123	TAS2R60
cg19717150	0.6672953	0.426462	-208.452	-0.2408334	HNF4A
cg10322876	0.6234811	0.3730989	-208.0348	-0.2503822	CYP2B6
cg07339138	0.5733532	0.3157791	-207.441	-0.2575741	CCDC13
cg06277657	0.4795639	0.2188082	-206.2004	-0.2607557	DGKI
cg07597976	0.5852943	0.330036	-205.9719	-0.2552584	CD19
cg23260026	0.506326	0.2455377	-205.9154	-0.2607883	FSTL3
cg22194129	0.5891984	0.3345531	-205.8387	-0.2546452	CLEC4C
cg09542291	0.7694307	0.5652779	-204.897	-0.2041528	SMCP
cg05912121	0.6600023	0.4197485	-204.2111	-0.2402538	TH
cg12022621	0.5507392	0.2929759	-204.206	-0.2577634	LAX1
cg02423618	0.5915165	0.3385462	-203.5699	-0.2529702	SPATA8
cg10779183	0.7421809	0.5277917	-203.5699	-0.2143893	ELA3A
cg18063149	0.6902608	0.4585783	-203.4422	-0.2316824	FMO3
cg15996947	0.6176445	0.3691847	-202.9583	-0.2484598	L2HGDH
cg15589427	0.7345403	0.5176773	-202.8995	-0.216863	MUC4
cg04574507	0.6489264	0.4068885	-202.8293	-0.2420379	CD1B
cg18533225	0.6892558	0.4576339	-202.8293	-0.2316219	KLHDC7B
cg02882813	0.7654443	0.5610455	-202.0898	-0.2043988	CST5
cg00756887	0.685461	0.4532082	-202.0223	-0.2322529	PVRL4
cg00689010	0.6543227	0.4142365	-201.6145	-0.2400862	NCSTN
cg15983538	0.745829	0.5341644	-201.0405	-0.2116647	SEMA4A
cg13694749	0.6845574	0.4531068	-200.1647	-0.2314506	SCN4A
cg16051685	0.7352245	0.5200791	-200.1647	-0.2151453	TRIM63
cg25072962	0.6253342	0.3800659	-200.0122	-0.2452683	MGC35295
cg03468463	0.6458012	0.404833	-199.8296	-0.2409682	SERPINB12
cg24851490	0.5042468	0.2473683	-199.5597	-0.2568786	RNASE2
cg17778867	0.6763697	0.4433345	-199.0074	-0.2330353	KRTAP10-8
cg01663968	0.3435176	0.1041754	-198.8416	-0.2393422	CTSZ
cg15422147	0.6884536	0.4594133	-197.874	-0.2290402	SERPINB5
cg03782453	0.3843829	0.1379444	-197.6023	-0.2464385	FLJ90575
cg00622552	0.379416	0.1344813	-196.4488	-0.2449348	ODF3L1
cg13960126	0.6412276	0.4013687	-196.2791	-0.2398589	CRB3
cg23580945	0.7498689	0.5423081	-196.1974	-0.2075608	FLJ43826
cg08555657	0.7449159	0.5355642	-196.0305	-0.2093517	SPRR2E
cg16242770	0.7622209	0.5597631	-195.7514	-0.2024577	KRTAP17-1
cg00042156	0.5952918	0.3476501	-195.7369	-0.2476417	MGC16291
cg12069042	0.6789856	0.4486949	-195.494	-0.2302907	PLXNB1
cg02324920	0.5251739	0.271412	-195.3787	-0.2537619	NEURL

cg00392257	0.6029352	0.3568412	-195.0708	-0.246094	ISG20L2
cg27418851	0.6592104	0.4242415	-194.9129	-0.234969	MBL2
cg01774645	0.682514	0.4536377	-194.73	-0.2288762	ARHGAP30
cg14366490	0.6375582	0.3979723	-194.6147	-0.2395859	TXNL6
cg10894512	0.6339787	0.3942764	-193.5892	-0.2397023	ACTA2
cg14179628	0.6389529	0.4002855	-193.5588	-0.2386673	TCEAL7
cg12951282	0.6104448	0.3665026	-193.5359	-0.2439422	ASGR2
cg25013053	0.6971928	0.4733745	-193.2639	-0.2238184	UNC45B
cg01530101	0.6279835	0.3874658	-193.039	-0.2405177	KCNQ1DN
cg15531099	0.5362542	0.2846212	-192.8764	-0.251633	LCE1D
cg19111262	0.5239811	0.2718823	-192.7185	-0.2520988	IGSF9
cg15542496	0.7282554	0.5147417	-192.7144	-0.2135137	PIP
cg18056600	0.3649402	0.125059	-192.3996	-0.2398812	ZMYND15
cg27257987	0.6470209	0.4111471	-191.8184	-0.2358738	PSG4
cg01982597	0.682607	0.4554784	-191.8184	-0.2271286	PGBD3
cg09426307	0.647449	0.4117493	-191.6891	-0.2356997	SEC14L3
cg24852661	0.3907833	0.1472487	-191.2217	-0.2435345	GOLPH2
cg20189782	0.7097399	0.491111	-190.9209	-0.2186289	MGC27121
cg19717326	0.4473534	0.1980299	-190.4245	-0.2493235	MYADM
cg04048249	0.6578084	0.4258133	-189.4161	-0.2319951	APOC3
cg04457051	0.6049128	0.3626765	-189.3793	-0.2422363	SCOC
cg18121684	0.6801006	0.4539918	-188.9359	-0.2261088	SERPINB13
cg20011352	0.3544226	0.1189879	-188.3634	-0.2354347	GPR124
cg21970438	0.7576416	0.5573325	-188.3066	-0.2003091	TTLL2
cg10883352	0.6726032	0.4449122	-188.2974	-0.2276911	
cg26353877	0.7535778	0.5524481	-186.9155	-0.2011297	APCS
cg08603768	0.6560817	0.4252316	-186.877	-0.23085	WNT8A
cg24884084	0.4305283	0.1852645	-186.1853	-0.2452638	SPRR1B
cg20311730	0.6280743	0.3918157	-186.1501	-0.2362586	NALP10
cg07484827	0.5007538	0.2525204	-185.9071	-0.2482334	CHRNA10
cg10071275	0.5805405	0.3373369	-185.643	-0.2432036	MYT1
cg14662756	0.6447378	0.4124581	-185.0998	-0.2322798	NPFF
cg06233503	0.5640055	0.3194705	-184.9828	-0.244535	KCNQ1
cg25957124	0.5272189	0.2802694	-184.862	-0.2469495	DNAH3
cg20649991	0.7353776	0.5287659	-184.7594	-0.2066116	LILRB5
cg12339029	0.7264883	0.5169507	-184.5264	-0.2095376	MYL1
cg07408456	0.6066627	0.3677737	-184.4929	-0.2388889	PGLYRP2
cg04962134	0.600041	0.360336	-184.21	-0.2397051	TRIM51
cg24825722	0.5957175	0.355834	-183.519	-0.2398835	ACADVL
cg26581729	0.4526273	0.2075006	-183.2974	-0.2451267	NPDC1
cg19384697	0.5005838	0.2541056	-183.2231	-0.2464781	UPK3B
cg01074640	0.6469871	0.4165729	-182.894	-0.2304142	IFNA17
cg07967308	0.6294427	0.3955495	-182.8102	-0.2338932	ACP5
cg25710140	0.6906511	0.4712271	-182.5639	-0.219424	MID1
cg02717866	0.6514395	0.4222467	-182.4948	-0.2291928	FLJ32771

cg17192247	0.3573814	0.1251042	-182.2415	-0.2322772	MAPRE3
cg15060813	0.5094273	0.2639813	-181.7884	-0.245446	LRFN3
cg19047670	0.6686655	0.4443225	-181.1409	-0.224343	CCND1
cg02187357	0.6349444	0.403676	-180.3298	-0.2312684	TBC1D22B
cg25400358	0.5628049	0.3212322	-180.2983	-0.2415727	GPR137
cg18530716	0.3409466	0.1133489	-180.0597	-0.2275977	SLC16A11
cg25101056	0.6888835	0.4706154	-179.8416	-0.2182682	KCNG4
cg01772980	0.6239709	0.391134	-179.5767	-0.2328368	SCGB1D1
cg12113132	0.5475886	0.3053133	-179.5094	-0.2422753	CCNDBP1
cg07412254	0.631498	0.4003092	-179.1762	-0.2311888	FLJ14816
cg01726767	0.6185221	0.3856026	-178.1981	-0.2329195	LALBA
cg25882366	0.3844072	0.1500984	-178.0105	-0.2343088	HOXB2
cg06539449	0.7392992	0.5380074	-177.8252	-0.2012919	CCND1
cg23131950	0.7224598	0.5155614	-177.6731	-0.2068984	AP2S1
cg12970084	0.677856	0.4579777	-177.6676	-0.2198782	ELF3
cg12850636	0.7149763	0.5057625	-177.5564	-0.2092139	TJP3
cg11070419	0.5480133	0.3075119	-176.8403	-0.2405014	C4BPA
cg09448880	0.6389734	0.4108276	-176.6613	-0.2281458	PGLYRP3
cg13271951	0.5252445	0.2836718	-176.5957	-0.2415726	FAM57B
cg08244028	0.6530562	0.4279732	-176.5101	-0.225083	MSH3
cg08972170	0.5567054	0.3177987	-175.4283	-0.2389067	Ells1
cg05093686	0.4766409	0.2359651	-174.8665	-0.2406758	MAB21L1
cg00626119	0.5367865	0.2970187	-174.672	-0.2397678	NTRK1
cg09300114	0.466732	0.2268877	-174.13	-0.2398443	SLC16A5
cg22341310	0.4293335	0.1925653	-173.2506	-0.2367682	ZNF541
cg24331162	0.7144635	0.5079948	-172.5983	-0.2064686	SYT8
cg03973663	0.4318539	0.1954147	-172.3968	-0.2364392	LYN
cg21038703	0.6577138	0.4362686	-172.3451	-0.2214452	ASB16
cg12061127	0.6687647	0.449944	-172.2692	-0.2188207	WFDC9
cg06531741	0.5358245	0.2979478	-171.7657	-0.2378768	HTR3B
cg01305625	0.5286632	0.290565	-171.5856	-0.2380982	PDLIM4
cg19910382	0.5956088	0.3636262	-171.3633	-0.2319826	FABP1
cg03752885	0.5251481	0.287275	-171.0334	-0.2378731	DAPK3
cg01993576	0.3897566	0.1591268	-171.0068	-0.2306298	SLC29A1
cg24429836	0.459862	0.2224433	-170.9769	-0.2374187	LDHD
cg00714377	0.3978373	0.1661512	-170.9214	-0.2316861	SLA2
cg15842430	0.627858	0.4014112	-170.7227	-0.2264468	FAM12B
cg04488758	0.6615677	0.4427364	-169.6075	-0.2188313	USP44
cg09546307	0.6743965	0.4586313	-169.5993	-0.2157652	CLEC4D
cg08260891	0.2960796	0.08571782	-169.3248	-0.2103618	PPGB
cg18473117	0.6979352	0.4888738	-168.8327	-0.2090614	CCDC22
cg20488657	0.6912975	0.4805511	-168.5854	-0.2107464	TFF3
cg22970435	0.3588514	0.1351392	-168.1033	-0.2237123	SPATS1
cg23278885	0.7050881	0.4985411	-168.0739	-0.2065469	TGM6
cg18396533	0.4189331	0.1866291	-168.0517	-0.232304	DYDC1

cg11161873	0.535502	0.300372	-167.6762	-0.23513	FLJ39575
cg03379131	0.2859704	0.07941443	-167.5633	-0.206556	ADAM15
cg06385087	0.2887798	0.08159076	-167.3418	-0.207189	CTSZ
cg16272420	0.7207115	0.5194365	-167.2311	-0.2012749	PNLIPRP2
cg19055231	0.2775417	0.07359323	-167.2229	-0.2039484	STAC
cg24489034	0.6910307	0.4811361	-167.1587	-0.2098947	LW-1
cg25982743	0.437015	0.2038067	-166.9709	-0.2332083	TIMP4
cg03003745	0.590077	0.3606759	-166.4518	-0.2294011	UNQ473
cg17788013	0.6975147	0.4902155	-165.831	-0.2072992	SPINK5
cg12775613	0.6292081	0.4063885	-165.5925	-0.2228196	HTR1F
cg26796190	0.2745275	0.07254623	-165.3597	-0.2019813	PYY
cg15741706	0.6950621	0.4875338	-165.1276	-0.2075283	CXorf48
cg19903229	0.5354032	0.302058	-165.1186	-0.2333452	C14orf105
cg05569220	0.5804387	0.3508244	-165.0798	-0.2296143	FLJ44861
cg00750606	0.5394855	0.3064509	-164.9978	-0.2330346	CDA
cg06303238	0.6522335	0.4347643	-164.3755	-0.2174692	SALL4
cg02812142	0.6484491	0.4305395	-163.8193	-0.2179096	ACMSD
cg04655481	0.7019932	0.4973483	-163.6068	-0.2046449	GPR21
cg00895324	0.7060616	0.5027089	-163.4087	-0.2033527	PCP4
cg04337944	0.4786991	0.245835	-163.3021	-0.2328641	FBLN1
cg04273431	0.3890684	0.1641052	-162.5736	-0.2249632	PRR3
cg21639401	0.6639407	0.4506229	-161.9144	-0.2133178	FLJ31222
cg06818777	0.3566888	0.13799	-160.9751	-0.2186988	CHAD
cg26420196	0.4721264	0.2411671	-160.7584	-0.2309592	GAS6
cg05636175	0.4301497	0.2018363	-160.56	-0.2283134	TNFRSF10C
cg05600717	0.3065997	0.0989812	-160.405	-0.2076185	FLJ13639
cg01550148	0.5474311	0.3180721	-160.3305	-0.2293591	H2AFY
cg04567009	0.5921081	0.3671353	-160.2795	-0.2249728	FCGR3B
cg14911395	0.4709089	0.2404331	-160.1345	-0.2304758	SEMA3B
cg26884581	0.3973826	0.1729121	-160.1345	-0.2244705	PYGM
cg10414946	0.6309593	0.4120933	-160.1164	-0.218866	MS4A2
cg03343942	0.5368052	0.3077216	-159.0457	-0.2290835	SLC39A5
cg24477636	0.5621782	0.3351369	-158.6557	-0.2270413	OR10H1
cg07922606	0.4949563	0.2652697	-158.3927	-0.2296866	HIST1H3E
cg08402568	0.6543597	0.441252	-158.3927	-0.2131077	MGC34647
cg19000186	0.668666	0.4588057	-158.2968	-0.2098603	CNGA1
cg13126790	0.6797944	0.4726199	-158.2845	-0.2071745	FLJ27255
cg22228134	0.5810493	0.3563382	-158.0062	-0.2247111	GZMH
cg20576002	0.6086753	0.3876974	-157.8225	-0.2209779	FAM112B
cg24628744	0.4040718	0.1804474	-157.6956	-0.2236244	H2AFY
cg07643942	0.5621251	0.3360526	-157.2767	-0.2260725	LACRT
cg21402035	0.6022036	0.3807133	-157.2247	-0.2214903	GALR3
cg07977490	0.6888297	0.484895	-156.8355	-0.2039347	C16orf45
cg18881269	0.6443889	0.4305101	-156.4996	-0.2138788	LEPREL2
cg01837574	0.535157	0.3078603	-156.3964	-0.2272967	TRAPPC1

cg27566805	0.4902384	0.2621932	-156.1122	-0.2280452	USH2A
cg00350478	0.6046786	0.3843304	-156.0766	-0.2203482	FRMD1
cg05654163	0.6304811	0.4144872	-155.7726	-0.215994	SLC39A2
cg06275635	0.5588427	0.3336254	-155.6891	-0.2252172	PGLYRP3
cg16545105	0.6570615	0.44682	-155.0011	-0.2102415	CRHBP
cg14740251	0.6919519	0.4909695	-153.5319	-0.2009824	SIGLEC5
cg21432842	0.5382873	0.3132383	-153.4712	-0.225049	CSF3
cg10125195	0.6904398	0.4892444	-153.2602	-0.2011954	LACRT
cg10586756	0.6810052	0.4776083	-152.9786	-0.2033969	NUP93
cg24541550	0.5734241	0.3516374	-152.6987	-0.2217867	MRVI1
cg08996986	0.6781263	0.4745287	-152.2007	-0.2035976	EPS8L1
cg03291145	0.6444508	0.4335856	-152.0665	-0.2108652	ARSF
cg24693053	0.4704029	0.2462509	-151.2278	-0.224152	MFSD7
cg21755709	0.477071	0.2527786	-151.1574	-0.2242924	C21orf124
cg00518911	0.5139667	0.2898824	-150.8174	-0.2240843	HOXA10
cg10837843	0.351329	0.140456	-150.6941	-0.210873	DUSP1
cg26415633	0.4676159	0.2440923	-150.4931	-0.2235236	KLK1
cg22083047	0.483888	0.2600347	-150.3575	-0.2238533	PRICKLE2
cg01861509	0.6773669	0.4748544	-150.3121	-0.2025125	SPOCK2
cg27337148	0.4365545	0.2152393	-149.8521	-0.2213152	CAMK1G
cg15903395	0.6549752	0.4478197	-149.7648	-0.2071555	FLJ25369
cg13552869	0.3978889	0.1807334	-149.4324	-0.2171555	SEZ6L2
cg18988110	0.4603425	0.237967	-149.3174	-0.2223755	ATAD4
cg11939496	0.6582251	0.452121	-149.2336	-0.206104	CD244
cg12781568	0.556967	0.3363344	-149.0491	-0.2206326	WT1
cg25020204	0.5781268	0.3598342	-148.5029	-0.2182926	DBH
cg14107638	0.6763214	0.4749922	-148.187	-0.2013293	MAGEA5
cg15303841	0.6011094	0.3858882	-148.076	-0.2152212	RFPL1
cg10787197	0.5716727	0.3530416	-148.0645	-0.2186311	C6orf105
cg04968473	0.5577998	0.3380138	-147.9757	-0.2197859	CYP1A2
cg04891836	0.644649	0.4369641	-147.5338	-0.2076849	TNFSF14
cg17217677	0.6465526	0.4392577	-147.5098	-0.2072949	SMPD3
cg15590780	0.4973402	0.2755506	-147.4011	-0.2217896	USH2A
cg19841506	0.3476608	0.1397175	-147.3861	-0.2079433	ZMYND15
cg24816455	0.4781379	0.2572576	-146.4137	-0.2208803	SEMA3B
cg03544379	0.4923198	0.2712615	-146.3941	-0.2210583	OR7C2
cg02218324	0.6499814	0.4441688	-146.3364	-0.2058127	RSHL1
cg03548857	0.4611185	0.2410386	-146.0997	-0.2200799	FFAR2
cg03453449	0.6149181	0.4031622	-145.9963	-0.2117559	USP44
cg03104936	0.6382935	0.4305624	-145.8713	-0.2077311	GRB10
cg08403419	0.647118	0.4411996	-145.6701	-0.2059183	RLN3R2
cg13053608	0.5109901	0.2912985	-144.6977	-0.2196916	LGP1
cg00436603	0.5485989	0.3305493	-144.6597	-0.2180497	CYP2E1
cg24910675	0.4141111	0.198589	-144.5577	-0.2155221	ENG
cg05112299	0.4981154	0.2784458	-144.5004	-0.2196697	OR7A17

cg19006008	0.6371928	0.4303488	-144.3388	-0.206844	F2RL3
cg05822532	0.5405232	0.3229848	-143.281	-0.2175383	ELN
cg20713492	0.3306163	0.1291897	-142.6242	-0.2014266	AQP10
cg11435943	0.5192146	0.3014214	-142.3919	-0.2177933	SERPINB12
cg23413307	0.6441457	0.4399536	-142.3819	-0.204192	LCE1F
cg13320683	0.6621972	0.4616855	-142.3288	-0.2005117	RHOBTB1
cg17095731	0.6429132	0.4386792	-142.1121	-0.2042341	LRP8
cg07115820	0.5770056	0.3632423	-142.0806	-0.2137633	EPX
cg06720660	0.6033224	0.392796	-141.9514	-0.2105263	RNASE6
cg24919884	0.657968	0.4569189	-141.8341	-0.2010491	ARHGEF16
cg16673198	0.6375206	0.4326599	-141.6117	-0.2048607	CPNE4
cg17910564	0.57946	0.3665345	-141.2933	-0.2129254	VDAC3
cg13125510	0.4704025	0.2536251	-141.1607	-0.2167774	C11orf44
cg11584690	0.3612086	0.1551923	-141.1607	-0.2060163	ZNF574
cg01970325	0.5379469	0.322032	-140.9426	-0.2159149	NELF
cg23458892	0.6534544	0.4522171	-140.8156	-0.2012373	SIGLEC7
cg08878744	0.5153375	0.2986611	-140.8053	-0.2166763	LCE1B
cg10370591	0.6195087	0.4126323	-140.2206	-0.2068765	TPO
cg08859675	0.6306909	0.4256767	-140.1799	-0.2050142	PDE4A
cg12351042	0.6480422	0.446734	-139.4052	-0.2013082	OR2B2
cg27622610	0.5085348	0.2932111	-138.8131	-0.2153237	OR1G1
cg23047271	0.3377871	0.1376363	-138.7522	-0.2001508	PRICKLE2
cg03956628	0.3994637	0.1900484	-138.3916	-0.2094153	MLH1
cg11750883	0.5592771	0.3468403	-138.2003	-0.2124367	C1orf42
cg15005385	0.56929	0.3578518	-138.0088	-0.2114382	CCL3L1
cg21453309	0.4336165	0.2214619	-137.6471	-0.2121546	FAM101A
cg14034870	0.5837391	0.3740469	-137.6212	-0.2096922	SFTPG
cg26189983	0.3507454	0.1493924	-137.1079	-0.201353	TNFRSF1B
cg07676849	0.5856572	0.3768139	-136.7584	-0.2088433	FOLR3
cg11052143	0.4073675	0.1983978	-136.5977	-0.2089697	ALS2CR11
cg09492887	0.3482586	0.1481997	-135.9145	-0.2000589	SLC26A5
cg01637734	0.6175476	0.4139752	-135.3356	-0.2035724	CD5L
cg17582777	0.6193945	0.416173	-135.2439	-0.2032215	EFNA3
cg19033555	0.5863889	0.3790877	-134.8257	-0.2073013	DEFB1
cg26661623	0.6339096	0.4334872	-134.663	-0.2004225	ASGR2
cg09971646	0.4091354	0.2020427	-133.8322	-0.2070927	DLK1
cg12970081	0.5756982	0.3680547	-133.8075	-0.2076435	GPR32
cg02157083	0.6308148	0.4305044	-133.8075	-0.2003103	APOA5
cg08815403	0.5746841	0.3671961	-133.4756	-0.2074881	HSD17B13
cg19421752	0.3997418	0.1941053	-133.2664	-0.2056364	SLC6A18
cg15782391	0.5685586	0.3607983	-133.0811	-0.2077602	ACPT
cg07220939	0.6247531	0.423998	-133.0695	-0.2007551	SLC22A12
cg06489008	0.55329	0.3443721	-133.0051	-0.2089179	CST11
cg22971191	0.516776	0.3062426	-132.8187	-0.2105333	SLC10A2
cg14036856	0.5625836	0.3550135	-132.169	-0.2075702	MGC52423

cg26218269	0.4887733	0.2787361	-131.8541	-0.2100372	MAB21L2
cg08157292	0.5814134	0.3758841	-131.8033	-0.2055293	PPP1R7
cg18223379	0.5193044	0.3100426	-131.265	-0.2092618	BPIL3
cg02202484	0.6153247	0.4151899	-130.2959	-0.2001348	SPRR4
cg23065097	0.4400008	0.2330687	-130.1714	-0.2069321	FKBP1B
cg01053621	0.5679817	0.3624098	-130.1639	-0.2055719	APOA2
cg12992720	0.4648356	0.2566777	-130.1187	-0.2081579	EDG4
cg00795812	0.392195	0.1902388	-129.543	-0.2019562	PDCD1
cg25659818	0.5754424	0.3711866	-129.3511	-0.2042558	CCL4
cg17091851	0.5275174	0.3200401	-129.3262	-0.2074774	LOC348174
cg06226384	0.4042277	0.2011019	-129.2648	-0.2031258	CACNG5
cg04143809	0.5718522	0.3674147	-129.1566	-0.2044376	FLJ39822
cg16377880	0.5974121	0.395909	-128.9289	-0.2015031	CYP4F3
cg19226099	0.5533149	0.3476415	-128.8071	-0.2056733	MC3R
cg13705284	0.5130768	0.3056751	-128.687	-0.2074017	ACOX2
cg01785568	0.5506277	0.3448997	-128.6539	-0.205728	MSX1
cg07950803	0.5072255	0.2999371	-128.4169	-0.2072884	CD1A
cg24888049	0.4579185	0.2515098	-128.2544	-0.2064087	FES
cg19787037	0.5487555	0.3437946	-127.5376	-0.2049609	SPAG11
cg24840099	0.5609211	0.3569342	-127.4014	-0.2039869	MSX1
cg12639234	0.5579125	0.3544624	-126.4211	-0.2034501	NAT2
cg16219122	0.482095	0.2767116	-126.0358	-0.2053834	ABCB1
cg26504906	0.4827824	0.2774218	-126.0011	-0.2053607	PRSS16
cg14141399	0.5067493	0.3017888	-125.4872	-0.2049604	HAS1
cg18490846	0.5259629	0.3215033	-125.4712	-0.2044596	C17orf73
cg24516901	0.4647579	0.2603575	-125.3859	-0.2044004	FLJ22746
cg09837803	0.5264704	0.3221414	-125.3464	-0.204329	IL16
cg00466436	0.5134117	0.3087902	-125.2321	-0.2046215	DEFB126
cg13859324	0.500455	0.2958379	-124.9985	-0.2046171	UNC45B
cg05696092	0.4847794	0.280595	-124.5179	-0.2041844	NOSIP
cg19464944	0.5453683	0.34275	-124.3512	-0.2026183	FCGR1A
cg26705561	0.5424584	0.3396781	-124.3477	-0.2027803	SEC31L2
cg22805308	0.5238139	0.3207603	-123.6418	-0.2030536	PLEKHG5
cg18239253	0.559132	0.3585832	-122.9183	-0.2005488	DEFB32
cg03918304	0.4470581	0.245693	-122.5869	-0.2013652	HOXD10
cg14826683	0.5406874	0.339337	-122.4321	-0.2013504	SPRR2D
cg07947016	0.4818926	0.279759	-122.004	-0.2021337	KLK2
cg23571857	0.5467227	0.346406	-121.5714	-0.2003167	BIRC4BP
cg10210238	0.4546087	0.2538639	-121.3372	-0.2007448	CDKN2B
cg18085517	0.5434423	0.3433844	-121.0168	-0.2000579	TRPM1
cg27069753	0.5239905	0.323727	-120.1736	-0.2002635	ELA3B
cg14511156	0.4926409	0.2920107	-120.025	-0.2006302	OSCAR
cg04245402	0.4984854	0.2982791	-119.5167	-0.2002063	C19orf21
cg17790333	0.4926285	0.6933803	120.9061	0.2007518	CYP11A1
cg04802221	0.418497	0.6214202	124.1755	0.2029232	LOC283849

cg24427660	0.5065683	0.7121113	127.7437	0.205543	PNPLA2
cg05253327	0.5510435	0.7536106	128.4231	0.2025671	B3GNT1
cg07251788	0.4873433	0.6970063	131.706	0.2096631	CLTCL1
cg17686260	0.4989893	0.715821	141.2148	0.2168317	MGMT
cg15926585	0.5148575	0.7312739	141.2148	0.2164164	COMT
cg05342835	0.3724449	0.585965	141.2148	0.2135201	SYNC1
cg16363586	0.3116095	0.6420277	349.1551	0.3304182	BST2
cg24101578	0.3397138	0.6606755	349.1551	0.3209617	CDH22
cg01346152	0.2509769	0.5709909	349.1551	0.3200139	DHRS3
cg14409083	0.2385762	0.5489431	349.1551	0.3103669	EMP1
cg15958424	0.2685107	0.5684447	349.1551	0.299934	ACPP
cg03852144	0.2662518	0.5514572	349.1551	0.2852054	GLRX
cg16517394	0.2047307	0.4885386	349.1551	0.2838078	TNFSF4
cg26509022	0.2033753	0.4830421	349.1551	0.2796668	ALDH1A3
cg08624249	0.4790302	0.7561793	349.1551	0.2771491	KIAA0889
cg18702197	0.3662047	0.6433522	349.1551	0.2771474	HOXD3
cg10861599	0.2820067	0.5572135	349.1551	0.2752068	TNFSF4
cg09871315	0.305374	0.5778506	349.1551	0.2724766	HOXA2
cg19224278	0.3148687	0.5863332	349.1551	0.2714646	ALDH1A3
cg06204948	0.226315	0.4946114	349.1551	0.2682964	MARK2
cg06507244	0.4464805	0.7133806	349.1551	0.2669001	DHX32
cg18172186	0.5204926	0.7851661	349.1551	0.2646735	KIAA1913
cg01103836	0.6749582	0.9357197	349.1551	0.2607614	MYO9B
cg14371590	0.3117769	0.5718333	349.1551	0.2600564	SLC26A10
cg01126560	0.3978014	0.6578573	349.1551	0.2600559	C9orf142
cg07285276	0.5220789	0.7782456	349.1551	0.2561668	RAPGEF1
cg25101936	0.5005782	0.7544968	349.1551	0.2539186	ZBTB16
cg02506908	0.4224889	0.6762525	349.1551	0.2537636	HPD
cg17105014	0.1782841	0.4286318	349.1551	0.2503477	GYPC
cg13547644	0.1747356	0.424314	349.1551	0.2495784	ACTA1
cg03605761	0.357101	0.6054595	349.1551	0.2483585	RNF126
cg17890764	0.4058547	0.6488432	349.1551	0.2429885	ITIH4
cg18392482	0.545368	0.7868214	349.1551	0.2414534	AMDHD1
cg12564453	0.5336493	0.7743607	349.1551	0.2407115	CETP
cg04106785	0.4860941	0.7256647	349.1551	0.2395707	CDK5R1
cg07236190	0.4856061	0.7251654	349.1551	0.2395594	AMDHD1
cg22628873	0.4528286	0.691975	349.1551	0.2391464	GGT6
cg02490034	0.3971752	0.6356252	349.1551	0.23845	MEST
cg13906813	0.3991899	0.6372017	349.1551	0.2380118	HLA-DPA1
cg24459563	0.4840423	0.7202178	349.1551	0.2361754	CACNG1
cg12177001	0.5207384	0.7556784	349.1551	0.2349401	IFI27
cg13351161	0.2395838	0.4730456	349.1551	0.2334618	SCARA3
cg11397854	0.1366182	0.3691158	349.1551	0.2324976	IQSEC1
cg03562120	0.3543206	0.5841243	349.1551	0.2298037	WISP2
cg03271907	0.5886568	0.8162938	349.1551	0.227637	MGMT

cg24012708	0.4222224	0.6492432	349.1551	0.2270208	HDHD3
cg02000005	0.1389588	0.365793	349.1551	0.2268341	CRIP1
cg02674804	0.6295489	0.8560374	349.1551	0.2264885	REEP6
cg13763232	0.4830189	0.7087107	349.1551	0.2256918	SLC6A6
cg26668713	0.1475352	0.372605	349.1551	0.2250698	SIPA1
cg11554605	0.5178393	0.7423853	349.1551	0.2245461	ASB4
cg27114026	0.5307989	0.7550393	349.1551	0.2242405	ELA1
cg03096975	0.1735029	0.3939396	349.1551	0.2204366	EML2
cg24122922	0.4226836	0.6421795	349.1551	0.219496	C20orf39
cg16616769	0.5399963	0.7581047	349.1551	0.2181085	MGC35048
cg22740835	0.3141955	0.5322756	349.1551	0.2180801	DDR2
cg09595479	0.1804643	0.3960773	349.1551	0.2156131	PRPH
cg22759185	0.5592517	0.7734538	349.1551	0.2142021	REEP6
cg23418591	0.1130913	0.3204938	349.1551	0.2074025	FLJ90166
cg17791651	0.2977996	0.50512	349.1551	0.2073204	POU3F1
cg24739326	0.1848891	0.3881737	349.1551	0.2032847	CHST8
cg17339202	0.2780684	0.4805886	349.1551	0.2025203	SYNC1

Supplementary table 2 – Probes differentially methylated between cluster A and normal tissue

TargetID	Cluster A.AVG_Beta	Cluster A.DiffScore	Cluster A.Delta Beta	Normal.AVG_Beta	SYMBOL
cg24101578	0.7332597	352.7258	0.3935458	0.3397138	CDH22
cg14409083	0.6145285	352.7258	0.3759523	0.2385762	EMP1
cg01346152	0.626482	352.7258	0.3755051	0.2509769	DHRS3
cg16363586	0.6836122	352.7258	0.3720027	0.3116095	BST2
cg15958424	0.6179878	352.7258	0.349477	0.2685107	ACPP
cg03852144	0.6107026	352.7258	0.3444507	0.2662518	GLRX
cg18702197	0.6866837	352.7258	0.320479	0.3662047	HOXD3
cg17105014	0.4973903	352.7258	0.3191062	0.1782841	GYPC
cg10861599	0.5965094	352.7258	0.3145027	0.2820067	TNFSF4
cg16517394	0.5172255	352.7258	0.3124948	0.2047307	TNFSF4
cg09871315	0.6114195	352.7258	0.3060455	0.305374	HOXA2
cg17890764	0.7031412	352.7258	0.2972865	0.4058547	ITIH4
cg02506908	0.71776	352.7258	0.2952711	0.4224889	HPD
cg08624249	0.7714651	352.7258	0.2924349	0.4790302	KIAA0889
cg06204948	0.5180413	352.7258	0.2917262	0.226315	MARK2
cg18172186	0.810114	352.7258	0.2896214	0.5204926	KIAA1913
cg01126560	0.6849098	352.7258	0.2871084	0.3978014	C9orf142
cg04106785	0.7731463	352.7258	0.2870523	0.4860941	CDK5R1
cg01103836	0.9594297	352.7258	0.2844715	0.6749582	MYO9B
cg19224278	0.5934695	352.7258	0.2786008	0.3148687	ALDH1A3
cg12564453	0.8119962	352.7258	0.2783469	0.5336493	CETP
cg13547644	0.4525869	352.7258	0.2778513	0.1747356	ACTA1

cg14371590	0.5890544	352.7258	0.2772775	0.3117769	SLC26A10
cg26509022	0.4798759	352.7258	0.2765006	0.2033753	ALDH1A3
cg13763232	0.7564856	352.7258	0.2734667	0.4830189	SLC6A6
cg25101936	0.7733716	352.7258	0.2727934	0.5005782	ZBTB16
cg11397854	0.4062031	352.7258	0.269585	0.1366182	IQSEC1
cg03096975	0.4415262	352.7258	0.2680233	0.1735029	EML2
cg07285276	0.7890551	352.7258	0.2669762	0.5220789	RAPGEF1
cg06507244	0.7111591	352.7258	0.2646786	0.4464805	DHX32
cg05342835	0.6354279	352.7258	0.262983	0.3724449	SYNC1
cg24459563	0.7467324	352.7258	0.2626901	0.4840423	CACNG1
cg13351161	0.5008287	352.7258	0.2612449	0.2395838	SCARA3
cg17791651	0.5555752	352.7258	0.2577756	0.2977996	POU3F1
cg18392482	0.799858	352.7258	0.25449	0.545368	AMDHD1
cg16616769	0.793669	352.7258	0.2536728	0.5399963	MGC3504 8
cg03605761	0.6107413	352.7258	0.2536402	0.357101	RNF126
cg11554605	0.7712382	352.7258	0.253399	0.5178393	ASB4
cg04700814	0.5465503	352.7258	0.252449	0.2941013	HEXIM1
cg07236190	0.7362686	352.7258	0.2506626	0.4856061	AMDHD1
cg24739326	0.4351847	352.7258	0.2502957	0.1848891	CHST8
cg02490034	0.6436431	352.7258	0.2464679	0.3971752	MEST
cg02674804	0.875141	352.7258	0.2455921	0.6295489	REEP6
cg03562120	0.5988758	352.7258	0.2445552	0.3543206	WISP2
cg00431549	0.5772381	352.7258	0.2437458	0.3334922	MGP
cg24012708	0.6654038	352.7258	0.2431815	0.4222224	HDHD3
cg05485060	0.6547932	352.7258	0.2428985	0.4118947	CTNNAL1
cg12177001	0.7634715	352.7258	0.2427331	0.5207384	IFI27
cg17339202	0.5202612	352.7258	0.2421929	0.2780684	SYNC1
cg08831348	0.5175472	352.7258	0.2405247	0.2770225	EML2
cg22740835	0.5542331	352.7258	0.2400376	0.3141955	DDR2
cg09595479	0.4201087	352.7258	0.2396445	0.1804643	PRPH
cg15926585	0.7544509	352.7258	0.2395933	0.5148575	COMT
cg24427660	0.7459579	352.7258	0.2393897	0.5065683	PNPLA2
cg24122922	0.661855	352.7258	0.2391714	0.4226836	C20orf39
cg22628873	0.6912075	352.7258	0.2383789	0.4528286	GGT6
cg26668713	0.3849941	352.7258	0.2374589	0.1475352	SIPA1
cg13906813	0.6349856	352.7258	0.2357956	0.3991899	HLA-DPA1
cg04640913	0.6280844	352.7258	0.2332859	0.3947985	CDH22
cg27114026	0.7597534	352.7258	0.2289546	0.5307989	ELA1
cg03271907	0.8175515	352.7258	0.2288947	0.5886568	MGMT
cg02000005	0.3650815	352.7258	0.2261227	0.1389588	CRIP1
cg22759185	0.7847828	352.7258	0.2255311	0.5592517	REEP6
cg24315815	0.7485856	352.7258	0.2243492	0.5242364	PLSCR4
cg24134767	0.6538556	352.7258	0.2232977	0.4305578	HTR3A
cg12232463	0.6534845	352.7258	0.2229266	0.4305579	LONRF2

cg06627364	0.4051811	352.7258	0.2221046	0.1830765	MGC4677
cg17518962	0.4466075	352.7258	0.2217132	0.2248944	GAL3ST4
cg23418591	0.3278128	352.7258	0.2147214	0.1130913	FLJ90166
cg22601917	0.4682869	352.7258	0.2139677	0.2543193	H6PD
cg26511075	0.8041921	352.7258	0.2107728	0.5934194	FLJ25422
cg03752628	0.4513408	352.7258	0.2099021	0.2414387	PTGFRN
cg27301343	0.3521307	352.7258	0.206864	0.1452667	EML2
cg23547429	0.4433117	352.7258	0.2063925	0.2369192	SLC43A3
cg15940569	0.4653472	352.7258	0.2003441	0.2650031	GABRB3
cg17998964	0.3640886	352.7258	0.2002974	0.1637913	MARK2
cg26394940	0.3185764	352.7258	0.2002554	0.118321	FLJ10945
cg17686260	0.7179564	144.0333	0.2189671	0.4989893	MGMT
cg20748065	0.8217276	144.0333	0.2027962	0.6189314	POR
cg13185308	0.6091214	141.1364	0.216127	0.3929945	ABCC8
cg05253327	0.7634494	141.1364	0.212406	0.5510435	B3GNT1
cg07251788	0.6994576	134.0077	0.2121144	0.4873433	CLTCL1
cg21685427	0.7496092	127.6712	0.2026033	0.5470058	SGK2
cg12866859	0.564211	126.7013	0.2018786	0.3623323	HEXIM1
cg04802221	0.6242384	126.1966	0.2057414	0.418497	LOC28384 9
cg16077929	0.7059836	122.454	0.202078	0.5039055	CDKL1
cg04902405	0.6831874	120.2602	0.2012576	0.4819297	ZC3H11A
cg20322977	0.305178	-118.4144	-0.2001438	0.5053217	CYP26C1
cg23855121	0.2799986	-119.1795	-0.2005431	0.4805417	TLR10
cg09712066	0.3317988	-119.6763	-0.2006135	0.5324123	PART1
cg00690280	0.3057972	-119.8623	-0.2013216	0.5071188	WFDC10B
cg18994063	0.3437956	-119.9316	-0.2003259	0.5441215	TIMD4
cg06190732	0.3028412	-120.0363	-0.2014804	0.5043216	SERPINA3
cg01899253	0.2514867	-120.1436	-0.200185	0.4516717	FLT1
cg15518950	0.2711562	-120.2079	-0.2011585	0.4723147	PRP2
cg24546463	0.2616771	-120.3635	-0.2009171	0.4625942	MGC3971 5
cg04780454	0.3553798	-120.4698	-0.2001289	0.5555087	SERPINF2
cg09551916	0.3277289	-120.9396	-0.2017671	0.529496	CFHR2
cg01193293	0.3164706	-121.0064	-0.2021138	0.5185844	SIGLEC7
cg19242268	0.3404177	-121.0124	-0.2013383	0.541756	TCEA2
cg09748960	0.2931482	-121.122	-0.20237	0.4955182	BTNL2
cg19618706	0.2499615	-121.9452	-0.2016473	0.4516088	BGN
cg06821120	0.2980053	-122.198	-0.2032683	0.5012736	RASSF1
cg11291009	0.225016	-123.5925	-0.2011363	0.4261523	ARHGEF9
cg07905963	0.2427093	-123.7094	-0.2026787	0.445388	CYP2A13
cg10078829	0.2649967	-123.9057	-0.2040406	0.4690373	KLK4
cg17589341	0.2931259	-124.1937	-0.2048698	0.4979957	SLC14A1
cg03641225	0.230982	-124.4566	-0.2024149	0.4333968	DIRAS3
cg08390254	0.278048	-124.7521	-0.2051388	0.4831868	ATP1A2

cg04991214	0.3881027	-124.811	-0.2008432	0.5889459	PFDN2
cg09573795	0.3831814	-125.2384	-0.2016078	0.5847892	MSX1
cg01375871	0.2882161	-125.8227	-0.2061679	0.4943841	TPSD1
cg09229960	0.381303	-126.0885	-0.2023905	0.5836935	EMD
cg15585987	0.2554448	-126.4039	-0.2057061	0.461151	SNTG1
cg21274025	0.2914673	-126.4936	-0.2067241	0.4981915	PLA2G3
cg02964385	0.2918202	-126.7198	-0.2069061	0.4987263	STK38
cg13530946	0.3950854	-126.7866	-0.2015653	0.5966507	IARS2
cg16567044	0.3598641	-126.8894	-0.2046869	0.564551	MEG3
cg22960952	0.2821444	-126.9434	-0.2070005	0.4891449	FLJ23657
cg02847500	0.2053086	-127.24	-0.2021651	0.4074737	SOX3
cg17034109	0.2978995	-127.4115	-0.2074535	0.505353	CYB561D1
cg05379350	0.1999034	-128.0079	-0.2021398	0.4020432	GIT1
cg05078019	0.3292986	-128.6128	-0.2076704	0.536969	PDE6B
cg13088755	0.3718534	-128.6896	-0.2050821	0.5769354	P2RY10
cg24625388	0.3042749	-128.7373	-0.2084416	0.5127165	NEBL
cg11885098	0.1913416	-128.9562	-0.2017857	0.3931274	EFNA2
cg01138020	0.3516943	-128.974	-0.2067776	0.5584719	MGC2967 1
cg15095327	0.3596787	-129.0713	-0.2063104	0.5659891	IL17RE
cg12493906	0.3520238	-129.4122	-0.2070758	0.5590996	MMP26
cg12188416	0.2908005	-129.4216	-0.2090559	0.4998563	TP73L
cg15952487	0.282603	-129.5231	-0.2090795	0.4916825	CD1B
cg16899036	0.4121099	-129.6008	-0.2016239	0.6137337	HOMER3
cg20822579	0.2196767	-129.6012	-0.205779	0.4254557	RIPK3
cg25427638	0.4107473	-129.7497	-0.2018911	0.6126384	CYP2A7
cg08695223	0.3229546	-129.7582	-0.2087946	0.5317491	SLC9A6
cg24516901	0.2558637	-130.2406	-0.2088943	0.4647579	FLJ22746
cg25391023	0.3174582	-130.2804	-0.2093625	0.5268207	BTNL2
cg22927134	0.3313952	-130.3938	-0.2089404	0.5403357	CHRM5
cg13370916	0.2228892	-130.5849	-0.206916	0.4298052	STARD8
cg06494770	0.334155	-130.6557	-0.2090115	0.5431665	KLHL13
cg22396129	0.2738834	-130.6557	-0.2098444	0.4837278	ZCRB1
cg05341878	0.3312151	-130.8823	-0.2093156	0.5405307	RIMS2
cg18946226	0.2123801	-131.0083	-0.2062153	0.4185955	MYR8
cg19937039	0.2143259	-131.0266	-0.2064409	0.4207667	SERPINA1 0
cg09871043	0.2681919	-131.0416	-0.2100105	0.4782025	PKHD1
cg15778232	0.317656	-131.0884	-0.20998	0.5276359	PHB2
cg21132577	0.2030788	-131.1667	-0.2052771	0.4083559	MYOM2
cg08831522	0.4055932	-131.2883	-0.2035489	0.6091421	ATP10A
cg03112433	0.2472252	-131.325	-0.2093503	0.4565755	PFTK1
cg26128441	0.3620113	-131.3663	-0.2078136	0.5698249	P2RX3
cg20070090	0.2993773	-131.6576	-0.2107921	0.5101693	S100A8
cg06785429	0.2677357	-131.7551	-0.210578	0.4783137	DCUN1D1

cg10479672	0.2233045	-131.905	-0.2080699	0.4313744	IL1F8
cg24252809	0.3921725	-132.2431	-0.2056834	0.5978559	MRGPRX1
cg22025233	0.4331007	-132.318	-0.2007014	0.6338021	CYP2W1
cg10329418	0.2559967	-132.5723	-0.2107975	0.4667942	PON3
cg27244482	0.3012956	-132.5942	-0.2114826	0.5127783	CAMK2A
cg17412560	0.275489	-132.6036	-0.2114277	0.4869167	CSEN
cg22534509	0.2509681	-132.8587	-0.2107947	0.4617628	GPR81
cg17141902	0.4249209	-132.9019	-0.2022038	0.6271248	NINJ1
cg23110514	0.2455575	-133.1624	-0.210747	0.4563045	LCE3E
cg12089698	0.3090793	-133.5026	-0.2120383	0.5211176	SPATC1
cg02149446	0.181548	-133.72	-0.2044099	0.3859579	C5AR1
cg24765079	0.2340671	-133.8411	-0.2105478	0.4446149	CDH1
cg05248470	0.2730139	-133.8799	-0.212388	0.4854019	LILRB2
cg14003512	0.3142816	-134.054	-0.212326	0.5266076	PLGLB2
cg24870391	0.2836571	-134.0543	-0.2126748	0.4963319	CCL11
cg23152755	0.3484918	-134.0714	-0.2107196	0.5592114	DEFB105A
cg24054653	0.1739829	-134.3727	-0.2037273	0.3777102	C1GALT1C1
cg04037732	0.2119074	-134.4534	-0.2090723	0.4209796	NLGN3
cg00689340	0.3576903	-134.5314	-0.2104098	0.5681001	RTKN
cg25771201	0.3807566	-134.6053	-0.2084787	0.5892353	SCDR10
cg07434382	0.4189802	-134.614	-0.2041244	0.6231046	LOC348174
cg02797569	0.3661812	-135.0655	-0.2101178	0.5762991	PCOLCE
cg26531804	0.2638909	-135.4019	-0.2133679	0.4772589	SPINT1
cg11873854	0.1882231	-135.5486	-0.2070014	0.3952245	LCN6
cg01346718	0.1908237	-135.7369	-0.2075422	0.3983659	CSNK1E
cg06469542	0.4203733	-135.7935	-0.2047104	0.6250837	THSD3
cg03294491	0.2164522	-135.8837	-0.2107423	0.4271945	SMAD2
cg05019001	0.2472808	-135.9794	-0.2131457	0.4604265	AR
cg11765205	0.1818747	-136.126	-0.2065444	0.3884191	ARL11
cg15869022	0.3471672	-136.126	-0.2123	0.5594673	GPR17
cg20017147	0.2959923	-136.1786	-0.2143196	0.5103118	TEX101
cg20189937	0.278811	-136.5787	-0.2146159	0.4934269	L2HGDH
cg14117297	0.3105381	-136.9484	-0.2146208	0.5251589	MGC23244
cg20104776	0.3539625	-137.149	-0.2125531	0.5665156	LDOC1
cg11630242	0.1496658	-137.3244	-0.2015264	0.3511922	AKAP10
cg08711674	0.2864629	-137.4816	-0.2153485	0.5018114	AKT1S1
cg13283751	0.145064	-137.5771	-0.2007078	0.3457718	GPX5
cg26644395	0.2473038	-137.9517	-0.2147191	0.4620229	UCN3
cg11959435	0.4498086	-138.0517	-0.2018388	0.6516473	IFNA1
cg03918304	0.2330286	-138.1843	-0.2140296	0.4470581	HOXD10
cg11787522	0.3221724	-138.2974	-0.2152137	0.5373861	STRA6
cg03014957	0.3529748	-138.6226	-0.2136601	0.5666349	DEFB118

cg14345281	0.1653643	-138.6542	-0.2058529	0.3712171	NHS
cg02554564	0.4470816	-138.8802	-0.2027748	0.6498564	NTF3
cg12878228	0.3427253	-138.8854	-0.2145511	0.5572764	PRSS1
cg16008138	0.2450157	-139.1944	-0.2155798	0.4605955	RNF190
cg13030582	0.1650685	-139.6483	-0.2066343	0.3717028	MFAP4
cg22986999	0.1567595	-139.6994	-0.2050559	0.3618154	MRGPRF
cg10919204	0.2066081	-139.9559	-0.213046	0.4196541	CDH6
cg25082710	0.2297456	-140.0118	-0.2152484	0.4449939	IVL
cg00443307	0.373426	-140.0306	-0.212898	0.586324	KLRG1
cg14076161	0.3039969	-140.3604	-0.2173117	0.5213086	PRB4
cg06132342	0.3706629	-140.4285	-0.2134314	0.5840943	KRTHB5
cg00400028	0.1650919	-140.6146	-0.2074653	0.3725572	ACPL2
cg11472424	0.46507	-140.6441	-0.200864	0.6659341	PFKFB1
cg04201347	0.3134785	-140.7607	-0.2173402	0.5308187	PRRG2
cg12530021	0.1670439	-140.8469	-0.2080245	0.3750684	SIGLEC12
cg02989940	0.3596947	-140.8469	-0.2146835	0.5743782	ERAF
cg13703437	0.4243395	-140.8877	-0.2074287	0.6317682	FYB
cg18414950	0.2376453	-140.9846	-0.2165813	0.4542266	PDK3
cg20312687	0.4111603	-141.2237	-0.2094319	0.6205922	DEFB118
cg17706173	0.2226909	-141.2915	-0.2157291	0.43842	C16orf30
cg17051440	0.4563577	-141.5576	-0.2028964	0.6592541	CLDN2
cg09584711	0.2366992	-141.5733	-0.2169933	0.4536925	HPR
cg21846488	0.1930393	-141.7934	-0.2129134	0.4059526	LCE4A
cg03886110	0.4701139	-141.8275	-0.20068	0.6707939	PECAM1
cg03960217	0.1693143	-142.0158	-0.2094267	0.3787409	LCE2C
cg01868128	0.4053701	-142.7082	-0.2111286	0.6164987	LCE5A
cg21283680	0.4450153	-142.7692	-0.2054803	0.6504956	SH3BP5
cg25697314	0.4050295	-142.8032	-0.2112346	0.6162641	IL26
cg09258965	0.4485958	-142.8194	-0.2049373	0.6535331	TBC1D7
cg26757722	0.2471447	-143.1702	-0.2188267	0.4659714	CACNG2
cg07022477	0.3212568	-143.1928	-0.2188037	0.5400606	HIF3A
cg18098286	0.4164928	-143.2794	-0.2100291	0.6265219	UGT1A10
cg03330516	0.4116905	-143.3961	-0.2107508	0.6224413	SRMS
cg10379687	0.2115823	-143.6322	-0.2165875	0.4281698	SPINLW1
cg11653864	0.2514844	-143.9112	-0.2195982	0.4710826	ELK1
cg11721194	0.358223	-144.1003	-0.2170462	0.5752692	SLAMF7
cg10345936	0.4589359	-144.1806	-0.2040199	0.6629558	SLC36A2
cg02164442	0.3741359	-144.1914	-0.2156478	0.5897837	ITGAD
cg10710439	0.2263446	-144.1914	-0.2183523	0.4446968	FLJ37549
cg26692016	0.4414303	-144.2926	-0.2069836	0.6484139	APOBEC1
cg12391921	0.4017639	-144.3346	-0.2126414	0.6144053	ITGB1BP2
cg13311440	0.2941735	-144.5328	-0.2206053	0.5147789	CD48
cg03599338	0.3792056	-145.1558	-0.2157747	0.5949804	SUSD2
cg17040807	0.16549	-145.1856	-0.2114108	0.3769008	CYGB
cg12435792	0.2837303	-145.3017	-0.2212702	0.5050005	PDE6B

cg27413508	0.228167	-145.3263	-0.2193979	0.447565	COX4I2
cg14724265	0.3385094	-145.4979	-0.2194948	0.5580043	PPEF2
cg00687674	0.2523653	-145.6092	-0.2209581	0.4733233	TMEM84
cg13396068	0.4492672	-145.662	-0.2065355	0.6558028	DCD
cg10818781	0.4087511	-145.6989	-0.2126103	0.6213614	PHB2
cg20790540	0.4506408	-146.1804	-0.2066086	0.6572493	PTCRA
cg25839227	0.3954162	-146.2385	-0.2146399	0.610056	ABI3
cg16536450	0.1970449	-146.3997	-0.2172039	0.4142489	UNQ2541
cg22879289	0.3132991	-146.4757	-0.2214967	0.5347958	NID1
cg15792367	0.2997589	-146.4855	-0.2219304	0.5216893	KLK11
cg02595219	0.2420373	-146.5807	-0.2212449	0.4632823	KCNE3
cg11328541	0.196887	-146.7819	-0.2175023	0.4143894	OR7C1
cg23216015	0.1672887	-146.971	-0.2132244	0.3805131	C7orf16
cg05982504	0.4795794	-147.4093	-0.2021373	0.6817167	IGFALS
cg23571857	0.3250641	-147.4612	-0.2216586	0.5467227	BIRC4BP
cg04515986	0.2463548	-147.5236	-0.2221797	0.4685345	FTHL17
cg16514843	0.381165	-147.5513	-0.2171338	0.5982988	PAX4
cg25650811	0.46213	-147.6704	-0.2054992	0.6676292	LOC223075
cg12354377	0.3049158	-147.6877	-0.2226599	0.5275756	ANK3
cg02988947	0.3266466	-147.7999	-0.2218112	0.5484578	LIMD2
cg18356799	0.3019392	-147.8987	-0.2228991	0.5248383	DSC1
cg27329371	0.3304183	-147.9071	-0.2216765	0.5520948	ALDH3A1
cg18806980	0.4388489	-147.9369	-0.2095889	0.6484378	KIAA0703
cg22202141	0.3842517	-147.9807	-0.2170756	0.6013273	FCGR3A
cg22549408	0.2736993	-148.7233	-0.2237812	0.4974805	PMAIP1
cg26745032	0.2319348	-148.7697	-0.2223457	0.4542806	REPS2
cg12616487	0.4818087	-148.8613	-0.2025152	0.684324	EML3
cg12397274	0.246868	-149.0077	-0.2233362	0.4702042	TINAG
cg16601385	0.4114724	-149.1962	-0.2144194	0.6258917	CFD
cg21529807	0.1239057	-149.3882	-0.2055418	0.3294475	CEACAM4
cg08834018	0.2937329	-149.6384	-0.2243297	0.5180627	PRODH
cg05973262	0.2888535	-149.6653	-0.2244275	0.513281	NOTCH4
cg20437604	0.4813178	-149.9854	-0.2032314	0.6845492	ANXA9
cg04739485	0.4025286	-150.0947	-0.2161793	0.618708	MLXIP
cg23324787	0.4008134	-150.0947	-0.2164026	0.617216	RAG2
cg18303397	0.2397192	-150.2395	-0.2239334	0.4636526	MBD4
cg02966851	0.2625979	-150.5136	-0.2249602	0.4875581	C6orf149
cg16219122	0.2572388	-150.5426	-0.2248562	0.482095	ABCB1
cg10478221	0.2903058	-150.5465	-0.2250453	0.5153511	WFIKKN1
cg17405586	0.4263836	-150.9531	-0.213324	0.6397075	KRT1
cg23075286	0.4092997	-151.0334	-0.2158496	0.6251493	GALP
cg10432859	0.4270325	-151.1385	-0.2133386	0.6403711	UGT1A7
cg22909609	0.2763251	-151.3004	-0.2256913	0.5020164	ITGBL1
cg00673191	0.2233127	-151.3388	-0.2237286	0.4470413	DOPEY2

cg24304714	0.2145681	-151.3756	-0.2230319	0.4376	LCE1C
cg01693350	0.2668423	-151.4644	-0.2257425	0.4925848	WT1
cg02436686	0.2404351	-151.5272	-0.2249544	0.4653894	GMFG
cg06323290	0.4013438	-151.6876	-0.217326	0.6186698	HK1
cg05492113	0.4573896	-151.7381	-0.2086656	0.6660552	TUB
cg04057858	0.3856391	-151.8873	-0.2194065	0.6050456	UNQ9391
cg26777475	0.3773532	-152.1175	-0.2204934	0.5978466	PCOLCE
cg22772878	0.2362997	-152.1621	-0.2252199	0.4615197	DIRAS1
cg14178895	0.2285026	-152.2419	-0.2248005	0.4533031	C6orf105
cg19006008	0.4221993	-152.6947	-0.2149934	0.6371928	F2RL3
cg14186992	0.4473409	-152.9292	-0.2110871	0.6584281	HKR3
cg01762581	0.204545	-152.9583	-0.2232794	0.4278245	DKK4
cg19258973	0.3955472	-153.6843	-0.2192921	0.6148393	KRTHB3
cg11656547	0.1086341	-153.7138	-0.2047213	0.3133553	MAMDC2
cg09076584	0.4620097	-153.7842	-0.2089669	0.6709765	FLJ25006
cg12682367	0.3934429	-153.8131	-0.2196435	0.6130863	FLJ46358
cg21624359	0.2508735	-153.8843	-0.2271428	0.4780163	FFAR3
cg02579133	0.4642254	-154.1701	-0.2087704	0.6729958	KRTAP10-8
cg24147596	0.4405248	-154.5361	-0.2131495	0.6536743	ARL14
cg26222045	0.3680768	-154.9147	-0.2232403	0.5913171	UNQ5810
cg01353448	0.158517	-154.9217	-0.2180867	0.3766038	C7orf16
cg18766847	0.4180777	-154.9822	-0.2169532	0.6350309	ACMSD
cg22937804	0.3106337	-155.2697	-0.2277431	0.5383769	MGC4450 5
cg11811840	0.4031739	-155.3922	-0.2193204	0.6224942	UGT1A1
cg04365980	0.4123907	-155.4745	-0.2180764	0.6304671	CLEC3A
cg19554294	0.3922128	-155.5953	-0.2208846	0.6130974	VN1R2
cg09001777	0.4307757	-155.621	-0.2153644	0.6461401	FUT3
cg10848367	0.440317	-155.7612	-0.2138831	0.6542001	SCGB1D2
cg15916061	0.3222231	-155.7636	-0.2275024	0.5497255	SLC17A4
cg01185080	0.3223415	-155.8091	-0.2275274	0.5498688	ZNF710
cg07197059	0.1707434	-156.0413	-0.2211574	0.3919009	EFS
cg24309555	0.407032	-156.2309	-0.2192938	0.6263258	APOB
cg15484375	0.5091329	-156.6051	-0.2009427	0.7100756	SAA1
cg25645748	0.4271277	-157.0951	-0.2167916	0.6439193	AKAP14
cg06980053	0.2599823	-157.3777	-0.2299094	0.4898917	RASSF1
cg14324675	0.3868751	-157.5825	-0.2227609	0.609636	LST1
cg26185508	0.4705416	-157.7764	-0.2095241	0.6800656	CDCP2
cg04833845	0.3969057	-157.8961	-0.2216625	0.6185682	KCNN4
cg15746187	0.4348632	-158.5422	-0.2163526	0.6512157	FBXO44
cg24340657	0.4289711	-158.7118	-0.2174114	0.6463825	KRT24
cg02046017	0.2394491	-159.051	-0.2304628	0.469912	LOC22007 0
cg21808053	0.2943644	-159.5813	-0.2312542	0.5256186	DIRAS3
cg27168844	0.4139656	-159.7097	-0.220312	0.6342776	IL17

cg22445920	0.4291323	-159.8096	-0.2180046	0.647137	SLC36A3
cg17191715	0.4532608	-159.8428	-0.2138712	0.667132	CA1
cg12019109	0.3623379	-159.8586	-0.2269299	0.5892678	AZGP1
cg02656594	0.1788845	-159.8925	-0.225427	0.4043115	IL21R
cg04189838	0.5201645	-160.0506	-0.2001466	0.7203111	CYP2C19
cg02633817	0.4756064	-160.2205	-0.2098199	0.6854264	FXYD3
cg06885782	0.2561986	-160.4227	-0.2320093	0.4882079	KCNQ4
cg15669228	0.3592457	-160.4373	-0.227605	0.5868507	IFNA8
cg20516209	0.4120677	-160.508	-0.2210638	0.6331316	EMILIN1
cg04740359	0.2572383	-160.5453	-0.2321214	0.4893597	NTF3
cg12937434	0.1239207	-160.6077	-0.2148091	0.3387298	BZRAP1
cg07745725	0.4106768	-160.7582	-0.2214178	0.6320946	PSG3
cg00134787	0.3632242	-160.9906	-0.2275463	0.5907705	MYH1
cg19998328	0.3342838	-161.0465	-0.2301728	0.5644566	LOC90580
cg23282949	0.4382015	-161.5398	-0.2174641	0.6556656	RENBP
cg16713808	0.4343469	-161.7284	-0.2182237	0.6525706	CRNN
cg16791508	0.5221641	-161.9435	-0.2005942	0.7227583	KRTHB3
cg14898892	0.3075231	-161.9442	-0.2323716	0.5398947	SHRM
cg24552358	0.4808476	-162.1369	-0.2097592	0.6906068	ORM1
cg01479232	0.3674792	-162.1369	-0.2278015	0.5952808	C20orf54
cg26917999	0.4056487	-162.1874	-0.2229825	0.6286312	LZTS1
cg21754343	0.3664255	-162.226	-0.2279745	0.5944	LCE2B
cg23029519	0.4901026	-162.5256	-0.2080323	0.6981349	IFNA4
cg09626634	0.3063965	-162.5256	-0.2328155	0.539212	EBI2
cg16463460	0.2481602	-162.5452	-0.233334	0.4814942	WT1
cg24654350	0.5075833	-162.6712	-0.2042977	0.711881	KIR3DL1
cg20795863	0.4858132	-162.678	-0.2090192	0.6948323	NEU2
cg03386373	0.3384578	-162.7298	-0.2309425	0.5694003	SPATA3
cg15329483	0.3690506	-162.7867	-0.2280393	0.5970898	SSX7
cg22563697	0.5068834	-162.8045	-0.2045243	0.7114077	PPP1R16A
cg04713352	0.3458334	-162.8282	-0.2303871	0.5762205	ATP4A
cg21109025	0.5168666	-163.0001	-0.2023481	0.7192147	CCL2
cg27655855	0.4963335	-163.2626	-0.2070765	0.70341	CST9L
cg01031251	0.3859918	-163.2712	-0.2263087	0.6123005	RPS6KA1
cg16175792	0.3128198	-163.3323	-0.2330513	0.5458711	HSD3B1
cg22607339	0.5102783	-163.4568	-0.2040805	0.7143588	MPL
cg21148892	0.499262	-163.6696	-0.206645	0.705907	CLEC4F
cg07979357	0.08702693	-163.6714	-0.2056606	0.2926875	IL27RA
cg22340747	0.478885	-163.7891	-0.2110323	0.6899173	GATM
cg11271605	0.0798566	-163.8154	-0.2030405	0.2828971	STEAP4
cg00684178	0.5271242	-163.8239	-0.2003419	0.7274661	NEU4
cg08474603	0.4495813	-163.8376	-0.2167383	0.6663196	CRP
cg20342105	0.2645445	-164.0333	-0.2347074	0.4992518	BSCL2
cg05190718	0.4868271	-164.1129	-0.2095499	0.696377	CASQ2
cg25193494	0.3795384	-164.1908	-0.2276791	0.6072175	FLJ20186

cg09686308	0.2520704	-164.2334	-0.2346686	0.486739	CIB3
cg00463848	0.5202302	-164.5338	-0.2023103	0.7225405	KRT2A
cg00962799	0.4972267	-164.6764	-0.2076032	0.70483	SSX8
cg12228229	0.5259702	-164.6785	-0.2010276	0.7269978	DLG4
cg04761824	0.4860111	-164.6785	-0.2100153	0.6960264	DEC1
cg14696870	0.4018438	-164.7306	-0.2250406	0.6268843	FCER1A
cg12891678	0.1800195	-164.7804	-0.2294143	0.4094338	SPRR2D
cg24959428	0.3664176	-164.8178	-0.229603	0.5960206	GBP6
cg11405695	0.2887962	-164.89	-0.2350741	0.5238702	ATAD3C
cg19096475	0.3954901	-165.3203	-0.2262816	0.6217718	ASAM
cg25994725	0.3284299	-165.6335	-0.2335966	0.5620265	C6orf81
cg13813391	0.5268831	-165.643	-0.2012666	0.7281497	CMTM2
cg13745346	0.4608822	-165.6597	-0.2156015	0.6764838	CBFA2T3
cg14550066	0.2985745	-165.8755	-0.2354167	0.5339913	NCR1
cg05696092	0.2486613	-166.3861	-0.2361182	0.4847794	NOSIP
cg17915429	0.4373794	-166.5669	-0.2204044	0.6577839	PGLYRP2
cg17288121	0.4200761	-166.5718	-0.2233145	0.6433907	DEFB103A
cg04893119	0.202651	-166.5737	-0.233454	0.436105	PI15
cg27337148	0.2028704	-166.8607	-0.2336841	0.4365545	CAMK1G
cg20305726	0.3052894	-167.1226	-0.235947	0.5412364	DEFB126
cg11505080	0.1753354	-167.1772	-0.2305676	0.405903	GPR173
cg13482233	0.5004858	-167.228	-0.2081434	0.7086292	HEPH
cg26895595	0.267673	-167.2844	-0.2369873	0.5046603	MAGEB3
cg13760253	0.4250703	-167.5545	-0.2230454	0.6481158	DNAJC5B
cg01248426	0.1986101	-167.58	-0.2337939	0.432404	ATP6V0D2
cg00476577	0.1026622	-167.6949	-0.2143964	0.3170587	ZNF217
cg02311163	0.3871416	-167.8128	-0.2288678	0.6160095	SEMG2
cg06415153	0.3160853	-167.8619	-0.2358528	0.5519381	PITPNM2
cg02787991	0.06907104	-167.934	-0.2020282	0.2710993	SECTM1
cg00415993	0.2159729	-168.1362	-0.2357567	0.4517296	F2RL2
cg02786019	0.3490456	-168.3644	-0.2335734	0.5826191	TRPV6
cg25545210	0.4722593	-168.4197	-0.2147719	0.6870312	KRTHA4
cg20176989	0.3674747	-168.501	-0.2317132	0.5991879	KIR3DL2
cg19067730	0.08652589	-168.5388	-0.2095201	0.296046	PPGB
cg07072643	0.324684	-168.6833	-0.2358161	0.5605	EMR3
cg21094154	0.5118819	-168.9326	-0.2063652	0.7182471	TNFSF11
cg05755354	0.3227683	-168.9326	-0.2361074	0.5588757	FRMD4A
cg08023751	0.5233502	-169.0672	-0.2037136	0.7270638	MERTK
cg26200585	0.3510107	-169.3282	-0.2339717	0.5849823	PRX
cg21122774	0.5288978	-169.3793	-0.2025092	0.7314069	SARDH
cg01135626	0.3508408	-169.5291	-0.2341154	0.5849562	CDX4
cg21789545	0.2376439	-169.7408	-0.2381341	0.475778	COL9A1
cg04995095	0.2861661	-169.8692	-0.2384805	0.5246466	CD300E
cg21745164	0.2970756	-169.9121	-0.2381449	0.5352205	LOC63928
cg09256683	0.3318933	-169.9559	-0.2360753	0.5679686	CCL14

cg23812886	0.293957	-170.0014	-0.2383255	0.5322825	SSX5
cg19642007	0.4515478	-170.0487	-0.2196898	0.6712376	TNNT3
cg24862483	0.08827329	-170.1185	-0.2114568	0.2997301	CD300LG
cg00057593	0.3380913	-170.1261	-0.2356733	0.5737647	GML
cg15423764	0.433304	-170.1617	-0.2230781	0.6563821	GLYAT
cg08952029	0.2331421	-170.1858	-0.2382709	0.471413	CHRDL2
cg09837803	0.2877089	-170.3389	-0.2387615	0.5264704	IL16
cg26062856	0.38914	-170.392	-0.2301055	0.6192455	ATP10A
cg24623694	0.219832	-170.4415	-0.2377102	0.4575422	PRX
cg03552103	0.3344351	-170.6925	-0.2363383	0.5707734	SEPT10
cg08356693	0.3005941	-170.853	-0.2386201	0.5392143	ITLN1
cg24816455	0.2388616	-171.2845	-0.2392763	0.4781379	SEMA3B
cg16222568	0.4992177	-171.5036	-0.2105034	0.709721	APEG1
cg23022999	0.410051	-171.5639	-0.2276976	0.6377487	FLJ45909
cg19464252	0.2670209	-171.5659	-0.2399244	0.5069454	FBS1
cg06836849	0.1550003	-171.6429	-0.2306228	0.3856231	SLC17A8
cg11554507	0.5153831	-171.7493	-0.2068728	0.7222558	NEUROD6
cg26189983	0.1262497	-171.8481	-0.2244957	0.3507454	TNFRSF1B
cg21519900	0.2898094	-171.9381	-0.2397571	0.5295665	C20orf186
cg04484789	0.5432475	-171.9461	-0.200081	0.7433285	KRT25B
cg08403419	0.4206955	-172.3644	-0.2264225	0.647118	RLN3R2
cg16381688	0.3098056	-172.6944	-0.2393318	0.5491374	THEM2
cg07173760	0.2707386	-172.7973	-0.2407282	0.5114667	CLC
cg18192417	0.1511996	-172.8857	-0.2308531	0.3820527	NEBL
cg13705284	0.272122	-173.1626	-0.2409548	0.5130768	ACOX2
cg21209356	0.4697278	-173.177	-0.217699	0.6874267	CSF2RB
cg25141995	0.2794072	-173.4225	-0.241009	0.5204162	VDAC1
cg12992720	0.224644	-173.5123	-0.2401917	0.4648356	EDG4
cg05559445	0.2381468	-173.6704	-0.2409189	0.4790657	CDKN1C
cg00556408	0.5173299	-173.8068	-0.2073564	0.7246863	TMPRSS6
cg11052143	0.1721704	-173.8951	-0.2351971	0.4073675	ALS2CR11
cg11432797	0.4781888	-173.9263	-0.2163012	0.6944901	SPN
cg05301852	0.3587352	-173.9263	-0.2359447	0.59468	FABP1
cg24698533	0.463597	-174.1518	-0.2194428	0.6830398	LYZL2
cg11061975	0.3062831	-174.2061	-0.2404965	0.5467796	SIRPB2
cg09863066	0.1916284	-174.6378	-0.2382662	0.4298946	PVALB
cg07892051	0.4135725	-174.9304	-0.2289844	0.6425568	AKAP3
cg05556717	0.362697	-175.2288	-0.2362531	0.5989501	CCL26
cg27132814	0.3591621	-175.3665	-0.236737	0.5958991	C20orf79
cg13407883	0.3527032	-175.4345	-0.2374839	0.5901871	SIGLEC9
cg08321330	0.4932618	-175.5022	-0.2137533	0.7070151	TPSB2
cg07846167	0.1084243	-175.5422	-0.2224821	0.3309064	FBLIM1
cg03956628	0.164266	-175.5446	-0.2351976	0.3994637	MLH1
cg04245402	0.2555839	-176.0273	-0.2429014	0.4984854	C19orf21
cg25712380	0.4921199	-176.069	-0.2142794	0.7063993	GRAP2

cg03752087	0.4659134	-176.227	-0.2200024	0.6859157	CASP14
cg14620221	0.544175	-176.3909	-0.2017685	0.7459434	OR8B8
cg24866437	0.5196635	-176.3911	-0.2079632	0.7276267	ALPK1
cg03741352	0.5294456	-176.3968	-0.2055436	0.7349892	LCE3C
cg09971646	0.1720424	-176.4669	-0.237093	0.4091354	DLK1
cg22545356	0.5298432	-176.5656	-0.2055191	0.7353623	MMRN2
cg20401945	0.1450611	-176.6759	-0.2325305	0.3775916	ASPHD1
cg05330360	0.2983609	-177.3285	-0.2428762	0.5412371	ZPBP2
cg16792160	0.3171225	-177.413	-0.241823	0.5589455	ASAH2
cg09799714	0.492415	-177.467	-0.2148734	0.7072884	PDZD3
cg18294257	0.1512761	-177.8764	-0.2346494	0.3859255	SEC14L3
cg21207418	0.524076	-177.9279	-0.2075572	0.7316332	ACP5
cg14722162	0.4227136	-177.9988	-0.2290908	0.6518043	C5orf20
cg01507173	0.4016342	-178.0601	-0.2325752	0.6342095	IL1F5
cg18462653	0.1670824	-178.2321	-0.23764	0.4047224	DEFB119
cg14321743	0.41698	-178.2968	-0.2302265	0.6472065	PLA2G2D
cg15905634	0.4952438	-178.5877	-0.2147511	0.7099949	TAS2R60
cg24898863	0.1523627	-178.5877	-0.2353886	0.3877513	S100A8
cg23047271	0.1118618	-178.6452	-0.2259253	0.3377871	PRICKLE2
cg18221897	0.4747705	-179.0287	-0.2195053	0.6942758	KIR2DL1
cg15798153	0.1848606	-179.0462	-0.2406797	0.4255403	PFTK1
cg20781967	0.1944294	-179.3583	-0.2419721	0.4364015	NINJ2
cg04623837	0.08405039	-179.3812	-0.2173758	0.3014262	HCG9
cg22088368	0.2889704	-179.5967	-0.2447318	0.5337022	MGC35206
cg21301440	0.1142713	-179.599	-0.2273628	0.3416341	CYGB
cg19464944	0.3009501	-179.976	-0.2444182	0.5453683	FCGR1A
cg04143809	0.3292805	-180.1863	-0.2425717	0.5718522	FLJ39822
cg24489015	0.3546581	-180.331	-0.2401475	0.5948056	LPO
cg09418321	0.2335984	-180.7991	-0.2456488	0.4792472	DYRK4
cg15417244	0.5006391	-180.8404	-0.2145427	0.7151818	PDZRN4
cg00174500	0.2385725	-180.8847	-0.2458715	0.4844441	CMTM5
cg22780475	0.3386708	-180.9333	-0.242165	0.5808358	CBLC
cg05135288	0.2294926	-181.1831	-0.2457512	0.4752438	RHOT2
cg15590780	0.2510184	-181.1935	-0.2463218	0.4973402	USH2A
cg07426848	0.4966059	-181.4606	-0.2157696	0.7123755	S100A3
cg20692181	0.3773851	-181.53	-0.23801	0.6153951	RETN
cg21312148	0.2843646	-181.5494	-0.2461383	0.5305029	LCE2D
cg07073964	0.4334992	-181.6257	-0.2290538	0.662553	PRSSL1
cg03366382	0.521148	-182.1225	-0.2101396	0.7312876	INS
cg12610070	0.0760156	-182.1621	-0.216488	0.2925036	TSPAN15
cg27349244	0.4199599	-182.2549	-0.2318037	0.6517636	MLXIP
cg25778479	0.1499293	-182.4929	-0.2378628	0.3877921	ANKMY2
cg03329572	0.4679669	-182.5371	-0.2226577	0.6906246	FCRL5
cg11819637	0.4160801	-182.5697	-0.2326434	0.6487235	THPO

cg07864297	0.1722869	-182.6507	-0.2416677	0.4139546	ESRRB
cg20261167	0.1710888	-182.9232	-0.2416915	0.4127803	SPP1
cg19756068	0.3971438	-183.0575	-0.2359844	0.6331282	CYP2B6
cg17446142	0.5528998	-183.542	-0.2024447	0.7553446	GDF9
cg25107791	0.3337537	-183.6117	-0.2442094	0.5779631	CLPS
cg19592945	0.1788472	-183.6962	-0.2432949	0.4221421	P2RXL1
cg08886154	0.2985988	-184.0688	-0.2470834	0.5456822	PAX4
cg01204985	0.5532609	-184.3357	-0.2026684	0.7559292	LILRA4
cg06906435	0.2883072	-184.4396	-0.247806	0.5361133	FLJ25773
cg14652095	0.2623776	-184.8415	-0.2487125	0.5110902	HIST1H1A
cg19587887	0.4716525	-185.213	-0.2231277	0.6947802	PSKH2
cg07017706	0.5008922	-185.329	-0.2165082	0.7174003	K6IRS3
cg02097420	0.422437	-185.378	-0.2329727	0.6554097	HRG
cg11161417	0.4324627	-185.4333	-0.2311696	0.6636322	SPACA3
cg11297236	0.2600591	-185.4928	-0.2491516	0.5092107	PDILT
cg17910564	0.3341281	-185.5929	-0.2453319	0.57946	VDAC3
cg17542495	0.3332354	-186.4919	-0.2459278	0.5791631	GJB1
cg01227741	0.5304788	-186.58	-0.2096636	0.7401425	GIMAP7
cg08157292	0.3355957	-186.6925	-0.2458177	0.5814134	PPP1R7
cg02688643	0.4987094	-186.7775	-0.2176711	0.7163805	MGST2
cg02064402	0.5156044	-186.9411	-0.2136138	0.7292182	SLC6A18
cg27420123	0.495501	-187.0249	-0.2185436	0.7140445	FSHB
cg11263296	0.08507877	-187.0776	-0.2238081	0.3088868	CCDC64
cg13530039	0.2840117	-187.0941	-0.2496217	0.5336334	CHRM1
cg10853416	0.4857021	-187.2482	-0.2209289	0.7066309	MS4A7
cg11898695	0.5226111	-187.3344	-0.2120146	0.7346257	PTCRA
cg13859324	0.250051	-187.4358	-0.250404	0.500455	UNC45B
cg01359534	0.548995	-187.5836	-0.2051503	0.7541453	AQP10
cg01375994	0.3060479	-187.6272	-0.2488029	0.5548508	MXRA5
cg22805308	0.2734386	-187.7448	-0.2503753	0.5238139	PLEKHG5
cg01678091	0.5450177	-187.7661	-0.2063077	0.7513254	MAGEL2
cg07443748	0.3290897	-188.3692	-0.2474139	0.5765036	CESK1
cg18849169	0.4742111	-188.4939	-0.2240961	0.6983072	GPX3
cg23704362	0.2981484	-188.4939	-0.2498056	0.547954	C8orf46
cg08763351	0.540401	-188.5065	-0.2078512	0.7482522	SPRR4
cg23350580	0.5591456	-188.8586	-0.202849	0.7619945	TBC1D3C
cg00319692	0.2488716	-188.8727	-0.2513249	0.5001965	ATP6V0D2
cg23244913	0.1293613	-188.9393	-0.2383253	0.3676866	HCG9
cg20657421	0.5100769	-189.0188	-0.2159013	0.7259783	CCL4
cg24621042	0.2923678	-189.1393	-0.2505191	0.5428869	SERPINA1
cg25462291	0.127876	-189.1721	-0.2381538	0.3660297	HEYL
cg23065097	0.1913309	-189.3079	-0.24867	0.4400008	FKBP1B
cg05955301	0.259077	-189.4541	-0.2517078	0.5107849	PRELP
cg22341310	0.1815527	-189.5454	-0.2477808	0.4293335	ZNF541
cg13397379	0.4021219	-189.8379	-0.2387664	0.6408883	OR2C3

cg03860768	0.405689	-189.9223	-0.2382175	0.6439065	BLK
cg24353217	0.5626703	-189.9816	-0.2023073	0.7649776	MYL2
cg11237817	0.3595492	-190.326	-0.2452187	0.6047679	KIR3DL3
cg10129493	0.5466718	-190.3601	-0.2069147	0.7535865	CD33
cg19447966	0.544962	-190.5143	-0.2074435	0.7524056	TEAD1
cg07220939	0.3824115	-190.7815	-0.2423416	0.6247531	SLC22A12
cg26385286	0.2207925	-190.837	-0.251871	0.4726635	GCNT2
cg18239253	0.3085514	-190.8682	-0.2505806	0.559132	DEFB32
cg17699374	0.2096387	-191.0643	-0.2513902	0.4610289	MGC35206
cg19372178	0.2190659	-191.2542	-0.2520638	0.4711297	TMEM16G
cg22456522	0.1882962	-191.2784	-0.2497362	0.4380323	LILRB3
cg23114594	0.4865209	-191.2899	-0.2225825	0.7091034	C10orf30
cg20856834	0.391454	-191.3356	-0.2412655	0.6327196	OR12D3
cg06226384	0.1584049	-191.3372	-0.2458228	0.4042277	CACNG5
cg23464269	0.2908892	-191.6624	-0.2521423	0.5430315	UGT1A3
cg01637734	0.3734074	-191.707	-0.2441403	0.6175476	CD5L
cg00466436	0.2602707	-191.7123	-0.253141	0.5134117	DEFB126
cg26390526	0.5707473	-191.7836	-0.2006925	0.7714398	FLG
cg16794682	0.5456471	-191.8445	-0.2077985	0.7534457	CCND1
cg26391080	0.5057938	-192.1013	-0.2183087	0.7241026	SH2D4B
cg09492887	0.1118676	-192.2669	-0.236391	0.3482586	SLC26A5
cg11584690	0.1220118	-192.4518	-0.2391968	0.3612086	ZNF574
cg26420196	0.2191599	-192.5803	-0.2529665	0.4721264	GAS6
cg19623751	0.3721772	-192.581	-0.2447893	0.6169665	CEACAM7
cg03872376	0.3943676	-192.9887	-0.241671	0.6360386	ZP4
cg08816023	0.282591	-193.201	-0.2534659	0.5360569	FGF1
cg12547930	0.3695998	-193.2702	-0.2455121	0.6151119	WFDC6
cg19421752	0.1532586	-193.4595	-0.2464832	0.3997418	SLC6A18
cg00501366	0.3396928	-193.4658	-0.2492948	0.5889876	ALOX12B
cg23765993	0.2265384	-193.724	-0.2540309	0.4805694	SPINLW1
cg13125510	0.2167867	-193.7425	-0.2536159	0.4704025	C11orf44
cg15895197	0.1532639	-193.7812	-0.2467191	0.399983	EMILIN1
cg07879977	0.2707552	-193.864	-0.254287	0.5250422	OR1F1
cg16899306	0.3768471	-194.0129	-0.2448877	0.6217347	HLA-DQB2
cg23807646	0.3482956	-194.145	-0.2487082	0.5970038	SLC26A8
cg19987219	0.423977	-194.1604	-0.2371205	0.6610975	FLJ32011
cg04901273	0.5331739	-194.2573	-0.2121556	0.7453295	TBC1D3
cg08260891	0.07170174	-194.3537	-0.2243779	0.2960796	PPGB
cg01987509	0.3350521	-194.3646	-0.2502983	0.5853504	PGR
cg11456838	0.2913683	-194.5312	-0.253859	0.5452273	LOC202459
cg04311964	0.5595344	-194.7117	-0.2050436	0.764578	LYPD2
cg16176600	0.5180808	-195.0281	-0.2164437	0.7345245	FRK
cg04675937	0.3883995	-195.1147	-0.243724	0.6321235	CDKN2B

cg05600717	0.07868465	-195.3764	-0.2279151	0.3065997	FLJ13639
cg03916421	0.1749437	-195.3972	-0.2510818	0.4260255	LOC132321
cg08784110	0.2847883	-195.9412	-0.2550288	0.5398171	MAS1
cg26457013	0.3860292	-195.9487	-0.2445239	0.6305531	TMEM86B
cg12029639	0.187334	-196.0352	-0.252923	0.440257	MAB21L1
cg24840099	0.306994	-196.3502	-0.2539271	0.5609211	MSX1
cg03473518	0.07966833	-196.3669	-0.2290527	0.308721	GJB6
cg02124291	0.4835402	-196.7791	-0.2257245	0.7092648	OR7A5
cg01993576	0.1425563	-197.1921	-0.2472002	0.3897566	SLC29A1
cg27069753	0.2675513	-197.2271	-0.2564392	0.5239905	ELA3B
cg07947016	0.2255829	-197.267	-0.2563097	0.4818926	KLK2
cg07790638	0.4195839	-197.2679	-0.2394649	0.6590487	LOC91431
cg20423977	0.5002202	-197.5256	-0.2220181	0.7222383	PLAC4
cg27383362	0.4146546	-197.5769	-0.2405222	0.6551768	ATAD3C
cg10300154	0.3792581	-197.588	-0.2464147	0.6256728	MGC5297
cg12069309	0.05796274	-197.6072	-0.2209702	0.278933	SEMA3B
cg09995854	0.4346041	-197.6072	-0.2367494	0.6713535	IL1F8
cg18988110	0.2048679	-197.6072	-0.2554746	0.4603425	ATAD4
cg19903229	0.2790282	-197.7318	-0.256375	0.5354032	C14orf105
cg14532417	0.5495685	-197.7729	-0.2090564	0.758625	TBC1D3
cg24214470	0.4805924	-198.7048	-0.2272708	0.7078633	SERPINF1
cg06825166	0.2774212	-199.1299	-0.257271	0.5346922	TMEM10
cg18807515	0.3739043	-199.1416	-0.2480186	0.621923	PRAMEF2
cg10986043	0.269507	-199.1659	-0.2575749	0.527082	TCAP
cg12954718	0.2331019	-199.3808	-0.2578864	0.4909883	USP6
cg17398613	0.5431722	-199.4206	-0.2114824	0.7546546	SLC37A1
cg01917648	0.2670681	-199.7622	-0.2580062	0.5250742	SPIC
cg26349773	0.5298046	-199.9836	-0.2153676	0.7451722	ATP6V0A4
cg19841506	0.1068064	-200.1482	-0.2408544	0.3476608	ZMYND15
cg12949760	0.4624073	-200.2698	-0.2321237	0.694531	KCNQ1
cg12067287	0.5452892	-200.3916	-0.2112646	0.7565538	MYOM1
cg14942312	0.446717	-200.3966	-0.2355795	0.6822965	GPR119
cg04520391	0.5324119	-200.4698	-0.2148566	0.7472685	PRB2
cg16739580	0.3859892	-200.4979	-0.2468791	0.6328683	POP2
cg07950803	0.2484412	-200.5692	-0.2587844	0.5072255	CD1A
cg26218269	0.2301659	-200.6104	-0.2586074	0.4887733	MAB21L2
cg06259570	0.3749972	-200.6784	-0.2486657	0.6236629	MMP27
cg08878744	0.2563497	-200.9959	-0.2589878	0.5153375	LCE1B
cg14141399	0.2476458	-201.0746	-0.2591035	0.5067493	HAS1
cg22971191	0.2577263	-201.1252	-0.2590497	0.516776	SLC10A2
cg21723486	0.3344979	-201.3726	-0.254266	0.5887639	TP73L
cg13281868	0.4199992	-201.9008	-0.2416415	0.6616407	C6orf142
cg17091851	0.2682325	-201.9155	-0.2592849	0.5275174	LOC348174

cg14102807	0.4628877	-202.5518	-0.2330446	0.6959323	CD19
cg10368842	0.3649952	-202.6897	-0.251159	0.6161542	C10orf81
cg27160701	0.3247343	-203.3379	-0.2563577	0.5810921	SBEM
cg14511156	0.2322271	-203.3996	-0.2604138	0.4926409	OSCAR
cg00896220	0.4733896	-203.6741	-0.2311351	0.7045248	CCL4L2
cg26523005	0.2885751	-203.9507	-0.2595774	0.5481524	ZNF662
cg18982568	0.3369105	-204.1849	-0.2555232	0.5924338	KRT1B
cg25168545	0.2251458	-204.5314	-0.2609497	0.4860955	GIMAP1
cg26796190	0.05121354	-204.7078	-0.223314	0.2745275	PYY
cg06142324	0.3849565	-204.7078	-0.2491695	0.634126	FLJ25530
cg05112299	0.2367335	-204.8406	-0.261382	0.4981154	OR7A17
cg10837843	0.1068444	-205.0626	-0.2444846	0.351329	DUSP1
cg16466334	0.4114963	-205.2865	-0.2448592	0.6563554	MMP3
cg22983092	0.3398155	-205.3493	-0.2558181	0.5956336	KRT25A
cg25691167	0.04296111	-205.4303	-0.2197262	0.2626873	FERD3L
cg17001430	0.5184201	-205.5039	-0.220631	0.7390511	KIF25
cg05921699	0.3834912	-205.7957	-0.249957	0.6334482	CD79A
cg26628847	0.2424536	-206.1199	-0.2622117	0.5046653	PIP
cg21985470	0.27349	-206.162	-0.2616197	0.5351098	PKLR
cg02694395	0.2835898	-206.201	-0.2611654	0.5447552	FMO4
cg03309967	0.3628917	-206.5321	-0.2534499	0.6163416	PSENEN
cg18971671	0.4581695	-206.8191	-0.2359996	0.6941691	TULP2
cg16377880	0.3408805	-206.909	-0.2565317	0.5974121	CYP4F3
cg08887581	0.4607326	-207.0609	-0.2355307	0.6962634	C1orf64
cg20932053	0.4846151	-207.1424	-0.2299456	0.7145607	CPM
cg01785568	0.2892318	-207.1489	-0.2613959	0.5506277	MSX1
cg02202484	0.361303	-207.1892	-0.2540217	0.6153247	SPRR4
cg13916742	0.5826701	-207.2329	-0.2027664	0.7854365	SCGB1D1
cg07026910	0.4975863	-207.4368	-0.2268423	0.7244287	INPP5D
cg19824441	0.3790114	-207.617	-0.251599	0.6306103	ADMR
cg22478614	0.2971772	-207.8334	-0.2612617	0.5584389	DEFB4
cg21643191	0.3713018	-207.9306	-0.2529476	0.6242494	ABCB5
cg07895149	0.04032382	-207.9989	-0.2203582	0.260682	FAM26B
cg21964481	0.4127044	-208.2769	-0.2460754	0.6587797	SLC34A3
cg18434152	0.4502668	-208.5515	-0.238529	0.6887958	PROK1
cg24812523	0.3824757	-208.66	-0.2515632	0.6340389	AKAP6
cg03387497	0.5722589	-208.7511	-0.2064905	0.7787495	C20orf179
cg08390209	0.3264298	-208.9224	-0.2592561	0.5856858	CDKN2B
cg05985767	0.4258343	-209.0487	-0.2438865	0.6697208	ANPEP
cg21458041	0.3722045	-209.218	-0.2534621	0.6256666	TNP2
cg01072821	0.3752697	-209.4346	-0.2530968	0.6283666	UNQ9391
cg15060813	0.2450855	-209.5837	-0.2643418	0.5094273	LRFN3
cg21407055	0.5834165	-209.5904	-0.2033554	0.7867719	ART1
cg21256656	0.4801772	-209.7368	-0.2321276	0.7123048	KLK6
cg25141490	0.2387892	-209.788	-0.2644728	0.503262	IL17B

cg18490846	0.2616155	-210.0518	-0.2643474	0.5259629	C17orf73
cg22247240	0.351932	-210.3811	-0.2569668	0.6088988	C14orf115
cg12732155	0.1977144	-210.7071	-0.2635661	0.4612805	LAPTM5
cg09748975	0.5205587	-210.8541	-0.222174	0.7427327	MSX1
cg26504906	0.2178204	-211.2484	-0.264962	0.4827824	PRSS16
cg14587868	0.3956705	-211.3521	-0.2506548	0.6463253	TGM1
cg12639234	0.294306	-211.6249	-0.2636066	0.5579125	NAT2
cg14120879	0.5368395	-211.8699	-0.2180349	0.7548744	DEFB105A
cg19055231	0.04923646	-212.3945	-0.2283052	0.2775417	STAC
cg00795812	0.1355233	-212.5402	-0.2566716	0.392195	PDCD1
cg10210238	0.1903151	-212.7219	-0.2642936	0.4546087	CDKN2B
cg01837574	0.2695171	-212.7394	-0.2656398	0.535157	TRAPPC1
cg09923671	0.4396296	-213.0279	-0.2428285	0.682458	GATA5
cg04557383	0.08205026	-213.9969	-0.2432006	0.3252509	MT1H
cg18530716	0.09381244	-214.0065	-0.2471341	0.3409466	SLC16A11
cg00518911	0.2465138	-214.8405	-0.2674529	0.5139667	HOXA10
cg26111757	0.4456798	-215.2077	-0.242453	0.6881328	C20orf185
cg08815403	0.3099729	-216.0585	-0.2647113	0.5746841	HSD17B13
cg06385087	0.05499241	-216.0808	-0.2337874	0.2887798	CTSZ
cg01530101	0.3708211	-216.2955	-0.2571624	0.6279835	KCNQ1DN
cg26822241	0.457723	-216.349	-0.2402183	0.6979414	CYP2C9
cg01367992	0.3264466	-216.349	-0.2631792	0.5896258	LY9
cg05654163	0.3737469	-216.3546	-0.2567343	0.6304811	SLC39A2
cg14826683	0.2730325	-216.5878	-0.2676549	0.5406874	SPRR2D
cg02280309	0.4073631	-216.5989	-0.2509618	0.658325	PKLR
cg23213217	0.2914748	-216.5989	-0.2665497	0.5580245	DEGS1
cg07676849	0.3217069	-216.8164	-0.2639503	0.5856572	FOLR3
cg09142399	0.4043372	-216.8766	-0.2516655	0.6560028	CRYZ
cg25043279	0.4414744	-216.9049	-0.244122	0.6855964	C7orf33
cg23988567	0.549861	-216.9933	-0.2161251	0.7659861	
cg26884581	0.137308	-217.0237	-0.2600746	0.3973826	PYGM
cg25677709	0.5155256	-217.0289	-0.2259115	0.7414371	NDST1
cg25993152	0.4645914	-217.1454	-0.2389628	0.7035542	XAGE5
cg26309498	0.5930557	-217.4859	-0.2029366	0.7959924	EDAR
cg15075718	0.3729053	-217.6643	-0.2575175	0.6304227	MFRP
cg21453309	0.168307	-217.7208	-0.2653095	0.4336165	FAM101A
cg04273431	0.1297887	-218.0439	-0.2592797	0.3890684	PRR3
cg03379131	0.05194796	-218.2336	-0.2340225	0.2859704	ADAM15
cg15005385	0.3026752	-218.3275	-0.2666148	0.56929	CCL3L1
cg15670863	0.4150099	-218.7974	-0.250489	0.6654989	SPACA4
cg14740251	0.4483435	-219.2486	-0.2436084	0.6919519	SIGLEC5
cg20713492	0.08303054	-219.5504	-0.2475858	0.3306163	AQP10
cg24429836	0.1911291	-219.6298	-0.2687329	0.459862	LDHD
cg04655481	0.4611241	-219.7526	-0.2408691	0.7019932	GPR21
cg08459368	0.5915831	-219.8641	-0.2041849	0.7957681	SCGB2A1

cg22083047	0.2136135	-220.1452	-0.2702745	0.483888	PRICKLE2
cg04637372	0.3392813	-220.4774	-0.2637782	0.6030594	FLJ32784
cg27117399	0.3298076	-220.515	-0.2649723	0.5947799	CNDP1
cg23458892	0.3990675	-220.6093	-0.2543869	0.6534544	SIGLEC7
cg10787197	0.3039027	-220.6765	-0.2677701	0.5716727	C6orf105
cg05131835	0.3990857	-220.7514	-0.2544518	0.6535376	GH2
cg23131950	0.4875756	-221.0504	-0.2348842	0.7224598	AP2S1
cg06946880	0.4768234	-221.2125	-0.2376667	0.7144901	ATP6V1B1
cg01474260	0.3340552	-221.4586	-0.2649415	0.5989967	CESK1
cg19382175	0.4938801	-221.5968	-0.2334663	0.7273464	PDE6A
cg24888049	0.1877903	-222.2455	-0.2701282	0.4579185	FES
cg21958034	0.5595706	-222.5708	-0.215165	0.7747356	MST1
cg19787037	0.2779873	-222.5987	-0.2707682	0.5487555	SPAG11
cg21038703	0.4031397	-222.6806	-0.2545741	0.6577138	ASB16
cg23173910	0.5481748	-222.9668	-0.2187489	0.7669237	ACTG2
cg19154438	0.5581197	-223.1932	-0.2158246	0.7739443	CKM
cg09528351	0.445501	-223.3235	-0.2459976	0.6914986	PIK3R5
cg15447486	0.4591606	-223.4468	-0.2428809	0.7020414	GPR109B
cg24506604	0.4648921	-223.6649	-0.2415971	0.7064892	LOC14450 1
cg12593411	0.3676518	-223.7839	-0.2613273	0.6289791	ANGPTL6
cg22937320	0.466188	-223.7983	-0.2413401	0.7075281	C9orf138
cg20485165	0.3904828	-224.3002	-0.2576758	0.6481587	WFDC12
cg10414946	0.3696597	-224.4028	-0.2612997	0.6309593	MS4A2
cg05500074	0.4653886	-224.4392	-0.2417941	0.7071827	TSKS
cg10586756	0.4313199	-224.5983	-0.2496853	0.6810052	NUP93
cg21168884	0.5250574	-224.9319	-0.226209	0.7512664	C6orf122
cg13320683	0.4072012	-225.3524	-0.254996	0.6621972	RHOBTB1
cg25214366	0.5405068	-225.4592	-0.221909	0.7624158	DEFB103A
cg26093687	0.5537579	-225.705	-0.2180185	0.7717764	EIF3S2
cg14036856	0.2908895	-225.9438	-0.2716942	0.5625836	MGC5242 3
cg27496506	0.5411245	-226.3328	-0.2220322	0.7631567	TGM5
cg22189286	0.4430644	-226.4991	-0.2478884	0.6909527	HSPB8
cg27291231	0.4476483	-227.2517	-0.2471504	0.6947987	SSNA1
cg05873268	0.4942114	-227.2649	-0.2355766	0.7297881	TPSAB1
cg22585988	0.4367001	-227.7524	-0.2498461	0.6865462	PVRL4
cg13552869	0.1316639	-227.7524	-0.266225	0.3978889	SEZ6L2
cg24910675	0.1454349	-227.7589	-0.2686762	0.4141111	ENG
cg03087937	0.436597	-228.0901	-0.2500168	0.6866138	MUC15
cg11161873	0.2607329	-228.2174	-0.274769	0.535502	FLJ39575
cg10464775	0.4168305	-228.638	-0.2545056	0.6713361	LAMP1
cg12943082	0.5133103	-229.1105	-0.2310544	0.7443646	CCL26
cg05822532	0.2654341	-229.1902	-0.2750891	0.5405232	ELN
cg21755709	0.2017805	-229.1981	-0.2752905	0.477071	C21orf124

cg27214365	0.5310996	-229.3187	-0.2260408	0.7571404	GYPB
cg24628744	0.1358298	-229.5988	-0.268242	0.4040718	H2AFY
cg19717326	0.173824	-229.616	-0.2735294	0.4473534	MYADM
cg03044435	0.5426577	-229.775	-0.2227785	0.7654362	FLJ35816
cg06489008	0.2785572	-229.9212	-0.2747328	0.55329	CST11
cg25177139	0.583678	-230.1203	-0.2100533	0.7937313	SLC10A6
cg02658251	0.387164	-230.6828	-0.2612046	0.6483686	DEFB4
cg03548857	0.1855687	-231.2609	-0.2755499	0.4611185	FFAR2
cg19292712	0.4926519	-231.4671	-0.2375874	0.7302393	SPAM1
cg19226099	0.2776383	-231.5848	-0.2756765	0.5533149	MC3R
cg05922591	0.6044611	-231.6551	-0.2036161	0.8080772	LILRB4
cg21402035	0.3317835	-231.7938	-0.2704201	0.6022036	GALR3
cg27566805	0.2123811	-232.8712	-0.2778573	0.4902384	USH2A
cg00689010	0.3931876	-233.0197	-0.2611351	0.6543227	NCSTN
cg23413307	0.3805593	-233.2938	-0.2635864	0.6441457	LCE1F
cg09841009	0.5244843	-233.4728	-0.2294388	0.7539231	GYPA
cg01668126	0.434213	-233.4852	-0.2528153	0.6870282	MSR1
cg14015044	0.04666576	-233.7196	-0.2430692	0.289735	TNFRSF10 C
cg26415633	0.1901024	-233.9493	-0.2775136	0.4676159	KLK1
cg04337944	0.2005767	-234.0101	-0.2781224	0.4786991	FBLN1
cg23471482	0.5722874	-234.1302	-0.2150213	0.7873088	CCL22
cg22988566	0.6061218	-235.1902	-0.2041106	0.8102324	WFDC10B
cg26705561	0.2639273	-235.364	-0.2785311	0.5424584	SEC31L2
cg24693053	0.191743	-235.7045	-0.2786599	0.4704029	MFSD7
cg25101056	0.4352384	-236.0701	-0.2536451	0.6888835	KCNG4
cg19257200	0.5134272	-236.3159	-0.2336299	0.7470571	SOX10
cg26264314	0.5165959	-236.6322	-0.2328376	0.7494335	NALP5
cg13726463	0.5006417	-236.8723	-0.2374265	0.7380682	COX6A2
cg07922606	0.2145593	-237.1462	-0.280397	0.4949563	HIST1H3E
cg00714377	0.1264112	-237.1757	-0.2714261	0.3978373	SLA2
cg09207718	0.2888857	-237.67	-0.2780257	0.5669113	CYP1A2
cg26661623	0.3657293	-237.7172	-0.2681803	0.6339096	ASGR2
cg22970435	0.09461148	-237.9651	-0.26424	0.3588514	SPATS1
cg10190509	0.551337	-238.2648	-0.2229867	0.7743237	CCL16
cg11939496	0.3946501	-239.2211	-0.2635749	0.6582251	CD244
cg06277277	0.2039344	-239.7655	-0.2816131	0.4855476	NR1I3
cg25882366	0.1138174	-240.0411	-0.2705898	0.3844072	HOXB2
cg12781568	0.2766865	-240.184	-0.2802806	0.556967	WT1
cg24489034	0.4357178	-240.4487	-0.2553129	0.6910307	LW-1
cg01550148	0.2660386	-240.9611	-0.2813925	0.5474311	H2AFY
cg16155702	0.5427942	-241.1593	-0.2265812	0.7693753	FGF21
cg04744379	0.5062443	-241.5128	-0.2375172	0.7437615	KLK15
cg27622610	0.225441	-241.7547	-0.2830938	0.5085348	OR1G1
cg03343942	0.2543135	-241.8897	-0.2824917	0.5368052	SLC39A5

cg24884084	0.1516542	-242.3381	-0.2788741	0.4305283	SPRR1B
cg06022562	0.6065037	-242.3666	-0.2060715	0.8125752	FLJ13841
cg03608577	0.5516407	-242.3666	-0.2242244	0.7758651	OR12D3
cg07531356	0.3956968	-242.5069	-0.2647918	0.6604886	INSL6
cg24949488	0.4116188	-242.5586	-0.2615371	0.6731559	DNTT
cg00563932	0.4554485	-242.7578	-0.2515072	0.7069557	PTGDS
cg03752885	0.2416102	-242.9764	-0.2835379	0.5251481	DAPK3
cg17192247	0.09081265	-243.0304	-0.2665687	0.3573814	MAPRE3
cg17582777	0.3454308	-243.204	-0.2739638	0.6193945	EFNA3
cg06818777	0.09008704	-243.3966	-0.2666018	0.3566888	CHAD
cg02192965	0.4333533	-243.5449	-0.2571197	0.690473	SLC3A1
cg06625767	0.5460433	-243.5562	-0.2263721	0.7724154	F12
cg24477636	0.2803108	-243.752	-0.2818674	0.5621782	OR10H1
cg03712237	0.5628262	-243.7593	-0.221123	0.7839491	SSX2
cg17217677	0.3777225	-243.8471	-0.2688301	0.6465526	SMPD3
cg03169180	0.3517169	-243.8959	-0.2733151	0.625032	NLGN2
cg05254747	0.4994555	-243.9204	-0.240308	0.7397634	SLC39A14
cg17386181	0.4899489	-244.0838	-0.2430054	0.7329543	MT1B
cg18085517	0.2596492	-244.7931	-0.2837931	0.5434423	TRPM1
cg01861509	0.4154456	-245.4577	-0.2619213	0.6773669	SPOCK2
cg12351042	0.3784694	-245.8754	-0.2695728	0.6480422	OR2B2
cg13758677	0.511733	-246.3703	-0.2376461	0.7493791	GAGE4
cg03973663	0.1505944	-246.3981	-0.2812595	0.4318539	LYN
cg03291145	0.3737101	-246.5437	-0.2707407	0.6444508	ARSF
cg06720660	0.3247464	-246.7385	-0.278576	0.6033224	RNASE6
cg00436603	0.2634565	-247.826	-0.2851425	0.5485989	CYP2E1
cg02218324	0.3797856	-247.8882	-0.2701958	0.6499814	RSHL1
cg13053608	0.2243562	-248.1226	-0.286634	0.5109901	LGP1
cg05569220	0.2977181	-248.6749	-0.2827206	0.5804387	FLJ44861
cg12970084	0.4142967	-248.8654	-0.2635593	0.677856	ELF3
cg00350478	0.3251449	-248.8892	-0.2795337	0.6046786	FRMD1
cg04511534	0.5155445	-249.2471	-0.2375124	0.7530569	GGT6
cg11435943	0.2316725	-249.9605	-0.2875422	0.5192146	SERPINB12
cg01970325	0.2507749	-250.428	-0.287172	0.5379469	NELF
cg00895324	0.4502617	-250.5968	-0.2557999	0.7060616	PCP4
cg00367281	0.3588663	-250.6364	-0.2751542	0.6340206	CHRNB3
cg02812142	0.3764105	-250.695	-0.2720386	0.6484491	ACMSD
cg18056600	0.09267846	-250.735	-0.2722618	0.3649402	ZMYND15
cg24541550	0.2887823	-250.7565	-0.2846418	0.5734241	MRVI1
cg17298704	0.5826992	-250.9348	-0.2167361	0.7994353	CLDN18
cg11750883	0.2731339	-251.0671	-0.2861431	0.5592771	C1orf42
cg09931793	0.4302919	-251.2943	-0.2609071	0.6911989	OR2K2
cg14034870	0.2999207	-251.3929	-0.2838184	0.5837391	SFTPG
cg18881269	0.3709347	-251.6059	-0.2734542	0.6443889	LEPREL2
cg14107638	0.4108543	-251.6894	-0.2654672	0.6763214	MAGEA5

cg00226923	0.5292994	-251.7381	-0.2342147	0.7635141	FGD2
cg08859675	0.3541074	-252.0137	-0.2765835	0.6306909	PDE4A
cg27513764	0.3943535	-252.1948	-0.2691676	0.6635211	EFCAB3
cg06958211	0.3147447	-252.5269	-0.2826168	0.5973614	PAK6
cg23873703	0.5523642	-252.8643	-0.2273394	0.7797037	KCNAB1
cg19033555	0.3020942	-252.8643	-0.2842948	0.5863889	DEFB1
cg18396533	0.1357731	-252.9798	-0.28316	0.4189331	DYDC1
cg09038914	0.5134486	-253.0403	-0.239427	0.7528756	GFAP
cg02157083	0.3535977	-253.225	-0.2772171	0.6308148	APOA5
cg01144251	0.4859082	-253.2493	-0.2474034	0.7333116	KLK9
cg03782453	0.1065735	-253.6487	-0.2778094	0.3843829	FLJ90575
cg20649991	0.4883307	-254.164	-0.2470469	0.7353776	LILRB5
cg06531741	0.246447	-254.2172	-0.2893775	0.5358245	HTR3B
cg06275635	0.2708662	-254.3235	-0.2879764	0.5588427	PGLYRP3
cg20891917	0.470459	-254.3933	-0.2520191	0.7224781	IFRD1
cg04705866	0.4319283	-254.7893	-0.2619016	0.6938299	GZMK
cg03352153	0.4334731	-255.5006	-0.261803	0.6952761	GLULD1
cg03544379	0.2017983	-255.5011	-0.2905215	0.4923198	OR7C2
cg03104936	0.3613836	-255.5724	-0.2769099	0.6382935	GRB10
cg18223379	0.2285603	-255.7561	-0.2907441	0.5193044	BPIL3
cg18473117	0.436765	-255.9038	-0.2611702	0.6979352	CCDC22
cg09343150	0.5436325	-256.6601	-0.2313237	0.7749562	MEN1
cg10894512	0.3553211	-257.1682	-0.2786576	0.6339787	ACTA2
cg08766149	0.4203504	-257.5329	-0.265674	0.6860244	GZMB
cg00819362	0.5363369	-257.9166	-0.2340246	0.7703615	CLIPR-59
cg06233503	0.2744303	-258.1121	-0.2895752	0.5640055	KCNQ1
cg00134539	0.4507644	-259.6862	-0.2590842	0.7098486	UBASH3A
cg00622552	0.09947708	-259.7033	-0.279939	0.379416	ODF3L1
cg04891836	0.3669757	-259.7423	-0.2776734	0.644649	TNFSF14
cg07115820	0.2878047	-259.8951	-0.2892009	0.5770056	EPX
cg00392257	0.3170984	-260.1018	-0.2858368	0.6029352	ISG20L2
cg10370591	0.3364127	-260.2356	-0.2830961	0.6195087	TPO
cg15782391	0.2778208	-261.107	-0.2907378	0.5685586	ACPT
cg00750606	0.2463965	-261.4402	-0.293089	0.5394855	CDA
cg15903395	0.3787432	-261.6642	-0.2762319	0.6549752	FLJ25369
cg10746737	0.4434244	-261.8705	-0.2617585	0.7051829	HLA-DRB5
cg19000186	0.3956558	-262.2639	-0.2730102	0.668666	CNGA1
cg10125195	0.4236531	-262.4191	-0.2667868	0.6904398	LACRT
cg19384697	0.2061875	-262.4456	-0.2943963	0.5005838	UPK3B
cg00344709	0.5416344	-262.6129	-0.2338045	0.7754389	ANKRD21
cg18790143	0.437057	-263.2229	-0.2638491	0.7009061	OTOS
cg21432842	0.243921	-263.7147	-0.2943663	0.5382873	CSF3
cg00209066	0.5819864	-263.9484	-0.2207322	0.8027186	BRD1
cg25957124	0.2320623	-264.3664	-0.2951566	0.5272189	DNAH3
cg22022041	0.5029412	-264.4533	-0.2463304	0.7492716	CCR9

cg16998872	0.5117315	-264.8137	-0.2438152	0.7555467	GYPE
cg15626350	0.1730309	-264.9078	-0.2944311	0.467462	ESR1
cg03003745	0.2995116	-265.5219	-0.2905655	0.590077	UNQ473
cg11070419	0.2531605	-265.6757	-0.2948528	0.5480133	C4BPA
cg03453449	0.328102	-265.7572	-0.286816	0.6149181	USP44
cg18967533	0.2436493	-266.2716	-0.2956727	0.539322	KLK6
cg19910382	0.3052712	-266.5583	-0.2903375	0.5956088	FABP1
cg16673198	0.3544933	-267.0427	-0.2830273	0.6375206	CPNE4
cg21505334	0.47451	-267.5179	-0.2555291	0.7300391	CEACAM5
cg12970081	0.2819273	-268.2698	-0.2937709	0.5756982	GPR32
cg01731341	0.5653226	-269.2832	-0.2279496	0.7932723	FGF6
cg25710140	0.4205144	-269.3308	-0.2701367	0.6906511	MID1
cg16504670	0.4400075	-269.3445	-0.2653506	0.7053581	FLJ20186
cg25982743	0.1429408	-269.4642	-0.2940742	0.437015	TIMP4
cg09864990	0.56778	-270.0331	-0.2273336	0.7951136	GATA5
cg10417559	0.5431135	-270.0331	-0.2355651	0.7786786	LMO6
cg13181284	0.6409424	-270.4356	-0.2008958	0.8418382	KRT6B
cg22228134	0.2865813	-270.7859	-0.294468	0.5810493	GZMH
cg25020204	0.2831326	-271.1342	-0.2949942	0.5781268	DBH
cg19481686	0.3429203	-271.5204	-0.2869291	0.6298494	CDKN2B
cg21639401	0.3849457	-271.6308	-0.278995	0.6639407	FLJ31222
cg04968473	0.2601665	-272.3537	-0.2976333	0.5577998	CYP1A2
cg07711097	0.3830023	-273.1255	-0.2799846	0.6629869	GML
cg04457051	0.3125393	-273.253	-0.2923735	0.6049128	SCOC
cg12339029	0.4668424	-273.3449	-0.2596459	0.7264883	MYL1
cg25659818	0.2789412	-273.4499	-0.2965012	0.5754424	CCL4
cg24825722	0.3018067	-273.4777	-0.2939108	0.5957175	ACADVL
cg11984608	0.5695336	-273.5178	-0.2277158	0.7972494	CLDN16
cg08555657	0.4925148	-273.588	-0.2524011	0.7449159	SPRR2E
cg19875656	0.5610448	-273.9405	-0.2307398	0.7917845	TSP50
cg24691255	0.6091955	-274.5809	-0.2138605	0.823056	SERPINB2
cg05446471	0.6350932	-275.2408	-0.2042929	0.8393861	HDAC11
cg02723372	0.5623582	-275.6206	-0.2307638	0.793122	RUNX3
cg18533225	0.4154668	-275.8857	-0.273789	0.6892558	KLHDC7B
cg25400358	0.2636187	-276.168	-0.2991863	0.5628049	GPR137
cg09426307	0.3617564	-276.7559	-0.2856926	0.647449	SEC14L3
cg17095731	0.3561249	-276.8296	-0.2867883	0.6429132	LRP8
cg01305625	0.2270165	-276.8296	-0.3016466	0.5286632	PDLIM4
cg07967308	0.3392382	-277.8171	-0.2902045	0.6294427	ACP5
cg01053621	0.2684119	-277.8654	-0.2995697	0.5679817	APOA2
cg27212977	0.6266514	-278.1718	-0.2082235	0.8348749	DEFA6
cg27257987	0.3603279	-278.5452	-0.286693	0.6470209	PSG4
cg01663968	0.06246698	-278.9345	-0.2810506	0.3435176	CTSZ
cg04048249	0.3734956	-279.1021	-0.2843128	0.6578084	APOC3
cg13126790	0.4015483	-279.1077	-0.2782461	0.6797944	FLJ27255

cg01309153	0.6482783	-279.6017	-0.2002066	0.8484849	SURF1
cg21541083	0.5813487	-279.6017	-0.2252475	0.8065962	STXBP2
cg02719634	0.5463156	-279.8842	-0.2374004	0.783716	SLC22A18 AS
cg07643942	0.2607775	-280.2285	-0.3013476	0.5621251	LACRT
cg03977657	0.5538131	-280.2803	-0.2349929	0.788806	LAMB3
cg15648315	0.4513979	-280.9472	-0.2664712	0.717869	FLJ26443
cg16016036	0.6208293	-280.9578	-0.2111141	0.8319434	TPO
cg16507522	0.5136249	-281.3313	-0.2484202	0.7620451	SERPINA3
cg18783781	0.5263722	-281.4824	-0.2444136	0.7707858	MGC4399
cg12022621	0.2476728	-281.7932	-0.3030664	0.5507392	LAX1
cg10490064	0.4478829	-282.0498	-0.2677978	0.7156807	CRYBB2
cg15741706	0.419955	-282.4341	-0.2751071	0.6950621	CXorf48
cg04567009	0.2929352	-282.7332	-0.2991729	0.5921081	FCGR3B
cg01255591	0.5978374	-282.9227	-0.2201717	0.8180091	R3HDML
cg02537838	0.5663452	-283.2268	-0.2315088	0.7978539	C20orf151
cg15531099	0.2315382	-283.3527	-0.304716	0.5362542	LCE1D
cg12113132	0.2434373	-283.4819	-0.3041513	0.5475886	CCNDBP1
cg22253945	0.5448371	-283.6391	-0.2389772	0.7838143	GPR45
cg27087809	0.4980678	-284.0695	-0.2540833	0.7521511	ACSBG1
cg08402568	0.3664	-284.6953	-0.2879597	0.6543597	MGC3464 7
cg07977490	0.4105672	-284.8373	-0.2782625	0.6888297	C16orf45
cg01484156	0.2451172	-285.5057	-0.3050075	0.5501247	NCALD
cg07408456	0.3082819	-285.5898	-0.2983808	0.6066627	PGLYRP2
cg24851490	0.1977015	-285.6934	-0.3065453	0.5042468	RNASE2
cg25531166	0.5178339	-286.3084	-0.2486001	0.766434	CTAG1B
cg15303841	0.3011009	-287.0264	-0.3000085	0.6011094	RFPL1
cg15842430	0.3326167	-287.1727	-0.2952414	0.627858	FAM12B
cg20095587	0.4439802	-287.5256	-0.2707281	0.7147083	TREM2
cg00042156	0.2935182	-288.7946	-0.3017735	0.5952918	MGC1629 1
cg22194129	0.2862951	-289.3222	-0.3029032	0.5891984	CLEC4C
cg01772980	0.3267902	-289.53	-0.2971807	0.6239709	SCGB1D1
cg16358738	0.4564345	-289.6003	-0.2680157	0.7244502	AGXT
cg15096123	0.6544133	-289.768	-0.2000601	0.8544734	KLK4
cg12069042	0.3952163	-290.0172	-0.2837692	0.6789856	PLXNB1
cg24901474	0.2441151	-290.6381	-0.3075053	0.5516204	RGS5
cg09458237	0.4147226	-291.2664	-0.2795594	0.694282	HSPA12B
cg10408410	0.4372455	-291.4086	-0.2738394	0.711085	RLBP1
cg23881725	0.1253204	-291.5779	-0.3043634	0.4296838	DLEC1
cg14934821	0.5965958	-291.6654	-0.2228435	0.8194393	GPSM1
cg09300114	0.1581555	-292.5821	-0.3085765	0.466732	SLC16A5
cg24852661	0.09223668	-292.6131	-0.2985466	0.3907833	GOLPH2
cg12850636	0.4419065	-292.801	-0.2730698	0.7149763	TJP3

cg01469547	0.5813481	-292.8955	-0.2287482	0.8100963	OR5P3
cg12775613	0.331373	-292.9869	-0.2978351	0.6292081	HTR1F
cg14662756	0.3503369	-293.0247	-0.294401	0.6447378	NPFF
cg16545105	0.3656366	-293.184	-0.2914249	0.6570615	CRHBP
cg19111262	0.2137915	-293.2231	-0.3101895	0.5239811	IGSF9
cg12332316	0.6302835	-293.3694	-0.2104363	0.8407198	F12
cg13960126	0.3457187	-293.5995	-0.2955089	0.6412276	CRB3
cg25889160	0.6341406	-293.9659	-0.2090622	0.8432028	SERPINA5
cg15140807	0.4784398	-294.286	-0.2632067	0.7416465	FLJ31222
cg24743310	0.6419602	-294.4726	-0.2060869	0.8480471	FLJ31196
cg27020690	0.06216067	-294.5948	-0.2910332	0.3531938	TERC
cg02324920	0.2142664	-294.6975	-0.3109075	0.5251739	NEURL
cg04962134	0.295958	-294.8112	-0.304083	0.600041	TRIM51
cg16051685	0.4687419	-295.6075	-0.2664826	0.7352245	TRIM63
cg00626119	0.2256173	-296.0958	-0.3111691	0.5367865	NTRK1
cg01325515	0.550091	-296.317	-0.2407348	0.7908258	CTAG2
cg05670596	0.1762175	-296.3468	-0.3115986	0.4878161	CCRL2
cg22039846	0.6537306	-296.3829	-0.2017903	0.8555208	KIR2DL1
cg10052840	0.1832747	-296.4127	-0.3118504	0.4951251	SEMA6B
cg17926869	0.5206151	-296.4702	-0.2507026	0.7713177	LOC11509<br>8
cg22220722	0.5238435	-296.5362	-0.2496639	0.7735075	PLA2G2A
cg24331162	0.4392722	-296.9139	-0.2751912	0.7144635	SYT8
cg06001166	0.6002716	-297.0007	-0.2228105	0.8230821	RPL3L
cg20011352	0.06177698	-297.3228	-0.2926457	0.3544226	GPR124
cg01982597	0.3963978	-297.3707	-0.2862092	0.682607	PGBD3
cg10779183	0.4775974	-297.8295	-0.2645836	0.7421809	ELA3A
cg09467501	0.07486033	-298.0313	-0.2972528	0.3721131	PYY
cg25552889	0.5505059	-298.372	-0.2411602	0.7916662	C3orf40
cg26353877	0.4935323	-298.8527	-0.2600455	0.7535778	APCS
cg26581729	0.141939	-299.0525	-0.3106883	0.4526273	NPDC1
cg17827767	0.587439	-300.142	-0.2283686	0.8158076	LRRC21
cg24908058	0.6278279	-300.2759	-0.2129888	0.8408167	CGB5
cg02833725	0.3561583	-300.6501	-0.2962384	0.6523967	ISG20L2
cg06303238	0.3556225	-301.3529	-0.296611	0.6522335	SALL4
cg19465374	0.5237271	-301.6609	-0.2511573	0.7748845	AZGP1
cg05636175	0.1206589	-301.8205	-0.3094908	0.4301497	TNFRSF10<br>C
cg02423618	0.2826235	-301.945	-0.308893	0.5915165	SPATA8
cg11346450	0.4539747	-302.0488	-0.2728043	0.726779	UGT1A3
cg24607398	0.2069951	-302.3382	-0.3148242	0.5218193	MLH1
cg12951282	0.3042613	-302.5775	-0.3061835	0.6104448	ASGR2
cg10409560	0.5097134	-302.6913	-0.2560535	0.7657669	FLJ23657
cg27394486	0.5625953	-303.3608	-0.238226	0.8008213	C15orf2
cg15480475	0.4463923	-303.4216	-0.2753881	0.7217804	TUB

cg24697329	0.4712322	-303.8142	-0.2683475	0.7395797	ARHGEF4
cg04488758	0.3661189	-304.0098	-0.2954489	0.6615677	USP44
cg08603768	0.3590076	-304.3621	-0.2970741	0.6560817	WNT8A
cg00075967	0.4368866	-304.5222	-0.2783756	0.7152622	STRA6
cg24816866	0.4815656	-306.1877	-0.2659447	0.7475104	PARK2
cg15542496	0.4541747	-306.2224	-0.2740807	0.7282554	PIP
cg18204685	0.396766	-306.3748	-0.289323	0.686089	BTD
cg02717866	0.3518708	-307.1404	-0.2995688	0.6514395	FLJ32771
cg03993463	0.6286557	-307.5837	-0.2143007	0.8429564	KCNJ15
cg00594952	0.2500748	-307.653	-0.3148771	0.564952	RIMS3
cg05093686	0.1596323	-308.3028	-0.3170086	0.4766409	MAB21L1
cg07484827	0.1828693	-308.3852	-0.3178844	0.5007538	CHRNA10
cg09037813	0.360417	-309.1925	-0.2985704	0.6589874	LRRFIP1
cg03609102	0.2561865	-309.3185	-0.3150454	0.5712318	MUC5B
cg17788013	0.4105215	-309.5193	-0.2869932	0.6975147	SPINK5
cg10071275	0.2659039	-310.7282	-0.3146366	0.5805405	MYT1
cg09448880	0.3346491	-310.8279	-0.3043243	0.6389734	PGLYRP3
cg02187357	0.3296021	-311.0429	-0.3053423	0.6349444	TBC1D22B
cg14911395	0.152816	-311.249	-0.3180929	0.4709089	SEMA3B
cg09546307	0.3786727	-312.4618	-0.2957239	0.6743965	CLEC4D
cg07297178	0.4961194	-312.68	-0.263298	0.7594174	CEACAM7
cg01459162	0.5414894	-313.1819	-0.2482433	0.7897327	PADI3
cg16272420	0.4405603	-313.1932	-0.2801511	0.7207115	PNLIPRP2
cg15210427	0.1945725	-313.2207	-0.3202599	0.5148324	CST9L
cg01074640	0.3432064	-313.7365	-0.3037807	0.6469871	IFNA17
cg25072962	0.3162956	-314.5058	-0.3090386	0.6253342	MGC3529 5
cg19368582	0.3428667	-314.5862	-0.3041656	0.6470323	MMRN2
cg19812619	0.6207693	-314.9917	-0.2190678	0.8398371	ITGB7
cg22510822	0.3391191	-315.1014	-0.3050966	0.6442158	OR1E2
cg13271951	0.2041817	-315.2852	-0.3210627	0.5252445	FAM57B
cg23278885	0.4181079	-315.3753	-0.2869802	0.7050881	TGM6
cg20576002	0.2959459	-315.5447	-0.3127294	0.6086753	FAM112B
cg04810997	0.3733408	-315.6746	-0.2980949	0.6714357	TAS2R60
cg02882813	0.5031943	-317.0327	-0.26225	0.7654443	CST5
cg24919884	0.3553892	-317.2199	-0.3025788	0.657968	ARHGEF16
cg00463202	0.5095125	-317.9836	-0.260433	0.7699456	ADPRHL1
cg16480209	0.6486808	-318.3035	-0.2084654	0.8571462	CNGB1
cg06277657	0.1572346	-319.036	-0.3223293	0.4795639	DGKI
cg13180098	0.3342773	-319.556	-0.3077018	0.641979	RHO
cg08996986	0.3802059	-319.7308	-0.2979205	0.6781263	EPS8L1
cg01726767	0.3055167	-320.0232	-0.3130054	0.6185221	LALBA
cg07597976	0.266815	-320.0261	-0.3184793	0.5852943	CD19
cg07412254	0.3210226	-320.3366	-0.3104755	0.631498	FLJ14816
cg07525077	0.2536817	-320.7127	-0.3202254	0.5739071	RNASE3

cg10127415	0.2024967	-320.9348	-0.3237528	0.5262495	MAGEB6
cg07446846	0.5388784	-320.9484	-0.251195	0.7900734	SLC6A8
cg26946769	0.5249795	-321.043	-0.256053	0.7810324	MAPK4
cg21495715	0.4291016	-321.4447	-0.2859782	0.7150798	SLC5A10
cg02981703	0.5698641	-321.7208	-0.2401904	0.8100545	CA6
cg01894895	0.5199026	-323.3341	-0.2584007	0.7783033	ANXA1
cg19917856	0.1876958	-323.3508	-0.3251599	0.5128558	LOC342897
cg24355048	0.3267258	-323.701	-0.3107148	0.6374406	CTSG
cg14366490	0.3267543	-323.9493	-0.3108039	0.6375582	TXNL6
cg02351381	0.1403574	-324.2376	-0.3238371	0.4641945	C12orf34
cg08972170	0.232967	-324.4896	-0.3237384	0.5567054	Ells1
cg16175725	0.5548052	-324.6436	-0.2464404	0.8012456	TCF1
cg06539449	0.4615954	-325.1357	-0.2777038	0.7392992	CCND1
cg07339138	0.2508708	-325.2944	-0.3224824	0.5733532	CCDC13
cg15422147	0.3910492	-325.8373	-0.2974044	0.6884536	SERPINB5
cg09508556	0.6528663	-326.4757	-0.2083774	0.8612437	PSORS1C2
cg17687962	0.4578489	-327.1526	-0.2794293	0.7372782	KLK3
cg05912121	0.3532359	-327.5354	-0.3067664	0.6600023	TH
cg23829949	0.2587149	-327.8643	-0.3227086	0.5814235	ZNF238
cg09542291	0.5045001	-328.2713	-0.2649306	0.7694307	SMCP
cg27157038	0.4754021	-329.0511	-0.2745782	0.7499803	DNTT
cg26918728	0.6052164	-329.3563	-0.2283987	0.8336151	SEMA3B
cg25013053	0.401154	-329.4645	-0.2960388	0.6971928	UNC45B
cg10248727	0.5675938	-329.9757	-0.2430153	0.810609	LCN1
cg13694749	0.3838293	-330.463	-0.3007281	0.6845574	SCN4A
cg20189782	0.4177759	-330.5232	-0.291964	0.7097399	MGC27121
cg14179628	0.3252831	-330.8939	-0.3136697	0.6389529	TCEAL7
cg04574507	0.3375845	-331.1469	-0.311342	0.6489264	CD1B
cg16986846	0.2694562	-331.8031	-0.3230155	0.5924717	SCGB2A1
cg00941549	0.3613079	-332.6506	-0.3067752	0.6680831	AKAP4
cg04431776	0.5529564	-333.0763	-0.2491806	0.802137	GAGE2
cg10604646	0.2080375	-333.1383	-0.3291107	0.5371482	RGS5
cg08093398	0.3401287	-334.0027	-0.3118396	0.6519684	PSF1
cg27090216	0.04763377	-334.4768	-0.3104566	0.3580904	TNFRSF10C
cg23260026	0.175757	-334.8237	-0.330569	0.506326	FSTL3
cg10883352	0.3656414	-336.0956	-0.3069619	0.6726032	
cg27418851	0.348381	-336.0956	-0.3108295	0.6592104	MBL2
cg19717150	0.358658	-336.3095	-0.3086373	0.6672953	HNF4A
cg20551517	0.4861671	-336.6554	-0.2732625	0.7594296	GIP
cg12061127	0.3603942	-336.6672	-0.3083705	0.6687647	WFDC9
cg10275770	0.1127888	-336.7608	-0.327266	0.4400549	ICAM2
cg25372195	0.346233	-337.6609	-0.3118493	0.6580823	DCD

cg21970438	0.4830888	-337.694	-0.2745528	0.7576416	TTLL2
cg06437862	0.4866819	-337.9285	-0.2734486	0.7601305	TUBA2
cg21624282	0.5447901	-338.3134	-0.2534465	0.7982366	LOC122258
cg08420900	0.6433162	-338.5591	-0.2147718	0.858088	LW-1
cg15983538	0.4651572	-339.1519	-0.2806718	0.745829	SEMA4A
cg18920397	0.4040957	-339.8966	-0.2986136	0.7027093	LY9
cg03458191	0.4701371	-340.3138	-0.2794302	0.7495673	SAA1
cg03468463	0.3293321	-340.7763	-0.3164691	0.6458012	SERPINB12
cg20488657	0.3881912	-340.98	-0.3031063	0.6912975	TFF3
cg20311730	0.3072675	-341.1833	-0.3208069	0.6280743	NALP10
cg07651914	0.2432898	-341.2409	-0.329929	0.5732188	CLDN15
cg19304352	0.5345432	-341.8284	-0.258023	0.7925661	DEFA4
cg14284171	0.5854444	-341.9161	-0.2389464	0.8243908	SSX4
cg07441143	0.3854205	-342.202	-0.304215	0.6896355	SLURP1
cg25477904	0.5583174	-343.0284	-0.249577	0.8078945	PSG1
cg06090864	0.6599521	-343.0347	-0.2085464	0.8684985	FFAR1
cg27442349	0.3126822	-343.0479	-0.3205225	0.6332046	NFKBIB
cg02130905	0.2806203	-343.7266	-0.326171	0.6067913	STMN4
cg01568736	0.4541648	-344.1411	-0.2855081	0.7396729	SERPINB7
cg22218909	0.6514704	-344.2061	-0.2124104	0.8638808	DEFA3
cg05547500	0.592259	-344.668	-0.2368767	0.8291358	TXNDC2
cg15996947	0.2924846	-346.0182	-0.32516	0.6176445	L2HGDH
cg15627025	0.6368562	-347.1357	-0.2191649	0.8560211	KIR3DL1
cg24750391	0.1530921	-347.203	-0.3359441	0.4890362	PON3
cg18063149	0.3839908	-347.4694	-0.30627	0.6902608	FMO3
cg05215575	0.2312278	-347.802	-0.3338161	0.5650438	FLJ25410
cg26813458	0.6523358	-347.8446	-0.2127216	0.8650575	CEACAM6
cg08448751	0.3454146	-349.2083	-0.3160277	0.6614423	SEMA3G
cg17894008	0.5739967	-349.2411	-0.2450243	0.819021	NACAL
cg17778867	0.3647156	-349.389	-0.3116541	0.6763697	KRTAP10-8
cg03931808	0.6866927	-352.7258	-0.2003426	0.8870353	RLN3
cg00269932	0.6904089	-352.7258	-0.2062206	0.8966295	LAIR2
cg02910574	0.689382	-352.7258	-0.2064093	0.8957913	PCOLN3
cg01119135	0.6717091	-352.7258	-0.2069625	0.8786716	C1orf116
cg20822628	0.7206532	-352.7258	-0.2089576	0.9296108	GATA5
cg21399079	0.6634654	-352.7258	-0.2100514	0.8735169	GPR45
cg08742106	0.673506	-352.7258	-0.2109548	0.8844607	USP6
cg25087423	0.6960046	-352.7258	-0.2135956	0.9096003	BLR1
cg21825364	0.6962662	-352.7258	-0.2137297	0.9099959	VCY
cg13696012	0.6619815	-352.7258	-0.2142497	0.8762311	BPIL1
cg25514503	0.6716544	-352.7258	-0.2181028	0.8897572	PER3
cg20676303	0.6611347	-352.7258	-0.2181656	0.8793003	GAGE7B
cg18565510	0.6920105	-352.7258	-0.2190725	0.911083	CENTB5
cg21003606	0.6634859	-352.7258	-0.2265315	0.8900174	CALN1

cg14519000	0.6486569	-352.7258	-0.2280897	0.8767466	GATA5
cg18231267	0.6558737	-352.7258	-0.2281056	0.8839793	RUNX3
cg03557698	0.6397599	-352.7258	-0.2294526	0.8692125	C1orf177
cg09837648	0.6361181	-352.7258	-0.2304895	0.8666076	PLXNB1
cg04254916	0.6310069	-352.7258	-0.2310483	0.8620552	KRT5
cg15379858	0.657989	-352.7258	-0.2312474	0.8892364	ChGn
cg07339327	0.6726423	-352.7258	-0.2312915	0.9039338	CCND1
cg06325687	0.6706373	-352.7258	-0.2318333	0.9024706	OPN1MW
cg05248781	0.6090333	-352.7258	-0.2322478	0.8412812	LCE5A
cg06985415	0.6139731	-352.7258	-0.2330909	0.847064	C10orf39
cg08124722	0.6074584	-352.7258	-0.2335812	0.8410395	CCL7
cg09283007	0.6699133	-352.7258	-0.2344526	0.9043659	FAM47B
cg18809535	0.6102067	-352.7258	-0.2347228	0.8449295	LDHAL6B
cg10938286	0.6095831	-352.7258	-0.2353019	0.844885	CST2
cg20543571	0.6037781	-352.7258	-0.2371295	0.8409076	C15orf43
cg12040555	0.6535989	-352.7258	-0.238906	0.8925049	MGMT
cg18389810	0.6020694	-352.7258	-0.2393957	0.8414651	C14orf8
cg12266049	0.5806788	-352.7258	-0.2432015	0.8238803	CCND1
cg13439299	0.5792388	-352.7258	-0.2455125	0.8247514	DNAJC5G
cg03782727	0.6093279	-352.7258	-0.2458876	0.8552155	FFAR1
cg00152644	0.5714646	-352.7258	-0.2497616	0.8212262	SPRR2E
cg19971655	0.6447574	-352.7258	-0.2501604	0.8949178	BSND
cg00601486	0.6407053	-352.7258	-0.2520304	0.8927357	H1T2
cg21277505	0.576471	-352.7258	-0.2526597	0.8291306	LOC28436 1
cg08634024	0.5632554	-352.7258	-0.2552119	0.8184674	OR2F1
cg16964535	0.6523905	-352.7258	-0.2571236	0.9095141	DNAJC5G
cg05564657	0.6216125	-352.7258	-0.2585815	0.880194	AADAC
cg14297029	0.5826994	-352.7258	-0.2590175	0.8417169	SSTR3
cg07123548	0.5566222	-352.7258	-0.260441	0.8170632	HIPK4
cg22013966	0.5803393	-352.7258	-0.2606457	0.8409851	SERPINA1 3
cg22627427	0.5840246	-352.7258	-0.2611043	0.8451289	C11orf9
cg14162076	0.5685815	-352.7258	-0.2611719	0.8297534	CLEC4D
cg08495878	0.6031184	-352.7258	-0.2614333	0.8645517	SERPINA4
cg08471713	0.5734982	-352.7258	-0.2617574	0.8352556	MEOX1
cg11830061	0.6532003	-352.7258	-0.2625183	0.9157187	INSL6
cg00727947	0.6085969	-352.7258	-0.2644539	0.8730508	LILRA5
cg18521925	0.6290919	-352.7258	-0.2653196	0.8944114	SLC22A16
cg11015241	0.5952066	-352.7258	-0.2658082	0.8610147	ATP10A
cg15538820	0.6493569	-352.7258	-0.2671272	0.9164841	OBP2B
cg07595943	0.6490885	-352.7258	-0.267704	0.9167925	LOC16193 1
cg09948350	0.5271527	-352.7258	-0.2679555	0.7951082	FLJ25084
cg20416179	0.5195642	-352.7258	-0.271061	0.7906252	C6orf71

cg23749046	0.5160941	-352.7258	-0.2721844	0.7882785	GPR61
cg04450876	0.511929	-352.7258	-0.2726637	0.7845926	FAM112B
cg08728865	0.5176678	-352.7258	-0.2739434	0.7916111	NALP7
cg09736922	0.516545	-352.7258	-0.2745708	0.7911158	THPO
cg25915982	0.5001695	-352.7258	-0.2756966	0.7758661	GRB10
cg11884243	0.5169803	-352.7258	-0.2761499	0.7931302	FCN2
cg05700681	0.514067	-352.7258	-0.2766355	0.7907025	CCL22
cg03534410	0.5837216	-352.7258	-0.2779413	0.8616629	TMEM40
cg13608094	0.5830495	-352.7258	-0.2810799	0.8641294	CCND1
cg17240454	0.5186989	-352.7258	-0.2812625	0.7999614	SPDEF
cg02601403	0.4871154	-352.7258	-0.2822627	0.7693781	TBC1D3C
cg00520135	0.5723745	-352.7258	-0.2831442	0.8555187	TPM1
cg21450627	0.5313782	-352.7258	-0.2842805	0.8156587	PSD4
cg27341860	0.6071019	-352.7258	-0.2842892	0.891391	OR2L13
cg25203980	0.5247045	-352.7258	-0.285175	0.8098795	CENTB5
cg14150666	0.5499311	-352.7258	-0.2854988	0.8354299	IL8RB
cg11113534	0.5701099	-352.7258	-0.2858634	0.8559733	C20orf70
cg11599505	0.4785994	-352.7258	-0.2864289	0.7650282	C20orf102
cg04719766	0.4963696	-352.7258	-0.2875593	0.7839289	KCNQ1
cg07126559	0.4705005	-352.7258	-0.2888094	0.7593099	SGCG
cg22294577	0.5155568	-352.7258	-0.2888189	0.8043756	SLC26A3
cg23753610	0.6408595	-352.7258	-0.2893309	0.9301904	DNAHL1
cg18129786	0.5201986	-352.7258	-0.2896685	0.8098671	ZNF445
cg00644033	0.6276304	-352.7258	-0.289936	0.9175664	MUC3B
cg24824840	0.4996942	-352.7258	-0.2904148	0.790109	SHANK1
cg14287742	0.45927	-352.7258	-0.2905264	0.7497964	BLZF1
cg18729973	0.4551771	-352.7258	-0.2906497	0.7458268	TFF1
cg01718139	0.4749766	-352.7258	-0.2908173	0.7657939	UNQ3033
cg08292050	0.4659346	-352.7258	-0.292015	0.7579495	SOCS4
cg08314660	0.5556598	-352.7258	-0.2920957	0.8477555	PKP3
cg23444894	0.4506426	-352.7258	-0.2921478	0.7427903	UNQ5810
cg12582965	0.5664625	-352.7258	-0.2924071	0.8588696	ATP10A
cg15589427	0.441911	-352.7258	-0.2926293	0.7345403	MUC4
cg23580945	0.4572249	-352.7258	-0.292644	0.7498689	FLJ43826
cg26473272	0.5734994	-352.7258	-0.2938896	0.867389	SYT8
cg04132607	0.5651802	-352.7258	-0.2939441	0.8591244	GATA5
cg16242770	0.4682765	-352.7258	-0.2939444	0.7622209	KRTAP17-1
cg05485062	0.4822264	-352.7258	-0.2946281	0.7768545	SERPINA1 2
cg17264470	0.5595945	-352.7258	-0.2947577	0.8543522	FGF21
cg27285599	0.4648957	-352.7258	-0.2966847	0.7615805	FLJ13841
cg08453096	0.5290136	-352.7258	-0.2968788	0.8258924	ABCG5
cg23514672	0.5750664	-352.7258	-0.2976111	0.8726774	FLJ32871
cg24363955	0.4870755	-352.7258	-0.29849	0.7855654	FLJ14054
cg27235662	0.4582622	-352.7258	-0.2988682	0.7571304	CLDN16

cg17820828	0.4241687	-352.7258	-0.3003717	0.7245404	KCNQ1
cg26422060	0.4854029	-352.7258	-0.3006387	0.7860416	TBX10
cg25781162	0.4525527	-352.7258	-0.3014656	0.7540183	ABCG5
cg18508525	0.5102794	-352.7258	-0.3014731	0.8117525	CD36
cg10213812	0.5602913	-352.7258	-0.3027199	0.8630112	FOXN1
cg08341924	0.454426	-352.7258	-0.3039003	0.7583263	TGM1
cg05440289	0.4500387	-352.7258	-0.3046627	0.7547014	IVL
cg04953015	0.4071358	-352.7258	-0.3065434	0.7136792	CHRNA2
cg04816348	0.5596771	-352.7258	-0.3065904	0.8662676	CLEC4G
cg08475088	0.5412613	-352.7258	-0.3072374	0.8484987	NALP9
cg25778166	0.417122	-352.7258	-0.3077362	0.7248582	FMO3
cg24407065	0.4939486	-352.7258	-0.3093171	0.8032658	BLZF1
cg27377450	0.497348	-352.7258	-0.3094599	0.8068079	ARHGEF18
cg19845843	0.4005892	-352.7258	-0.3094771	0.7100663	CXorf20
cg08424423	0.4908563	-352.7258	-0.3100212	0.8008775	CDSN
cg07792737	0.5020545	-352.7258	-0.3102618	0.8123163	NPIP
cg01643624	0.4163384	-352.7258	-0.3102866	0.726625	C11orf16
cg17741572	0.4864202	-352.7258	-0.3106194	0.7970396	CFB
cg19561774	0.5484324	-352.7258	-0.3108134	0.8592458	SLC22A2
cg05556202	0.4470188	-352.7258	-0.3110004	0.7580193	TM4SF19
cg01827098	0.4837575	-352.7258	-0.31135	0.7951075	GIMAP7
cg18121684	0.367482	-352.7258	-0.3126186	0.6801006	SERPINB13
cg15516226	0.483029	-352.7258	-0.3151099	0.7981389	BTNL9
cg11739626	0.3849224	-352.7258	-0.3154379	0.7003604	AKT1S1
cg15905124	0.4554797	-352.7258	-0.3157116	0.7711913	MGC1303<br>4
cg14333565	0.4755068	-352.7258	-0.3159598	0.7914666	NRTN
cg06236276	0.4412875	-352.7258	-0.3168153	0.7581028	SLC22A2
cg17829936	0.3679025	-352.7258	-0.3175393	0.6854419	TAAR5
cg01657380	0.3522651	-352.7258	-0.3176072	0.6698722	NPFF
cg25033144	0.4147533	-352.7258	-0.3178829	0.7326362	FLJ00060
cg19233472	0.4040452	-352.7258	-0.3183095	0.7223547	FOXI1
cg22190114	0.397056	-352.7258	-0.3183255	0.7153814	NALP8
cg23776892	0.4579759	-352.7258	-0.3186967	0.7766726	MAGEA1
cg02067021	0.3696525	-352.7258	-0.3189273	0.6885798	DNAJC5B
cg02442161	0.4338206	-352.7258	-0.3193065	0.7531271	PI3
cg21065959	0.3393786	-352.7258	-0.3194018	0.6587805	LCE1E
cg18138484	0.5063573	-352.7258	-0.3194674	0.8258247	CABP2
cg13656062	0.4324334	-352.7258	-0.3196643	0.7520977	CYP4F2
cg01774645	0.3627444	-352.7258	-0.3197696	0.682514	ARHGAP30
cg13928961	0.381842	-352.7258	-0.3199427	0.7017848	K6IRS3
cg04323365	0.4239616	-352.7258	-0.3202024	0.744164	GJB1
cg19047670	0.3474835	-352.7258	-0.321182	0.6686655	CCND1
cg24027679	0.4544323	-352.7258	-0.3215316	0.7759639	SLC2A7
cg22784047	0.3632978	-352.7258	-0.3233001	0.6865979	MVP

cg22021786	0.387661	-352.7258	-0.3237695	0.7114305	WFDC8
cg15743985	0.3801671	-352.7258	-0.3244269	0.704594	CD22
cg25813714	0.5184745	-352.7258	-0.3251191	0.8435937	CYP4F12
cg18979223	0.3353037	-352.7258	-0.3252977	0.6606013	CDKN2B
cg11158374	0.3795393	-352.7258	-0.3255305	0.7050698	TFF2
cg26583078	0.3343017	-352.7258	-0.3268023	0.6611041	SORBS2
cg24735489	0.2974933	-352.7258	-0.3269925	0.6244857	CDSN
cg18242139	0.3858212	-352.7258	-0.3275895	0.7134107	ELAVL4
cg19042947	0.3109535	-352.7258	-0.3277499	0.6387034	SERPINA4
cg00756887	0.3576337	-352.7258	-0.3278273	0.685461	PVRL4
cg12619162	0.4404047	-352.7258	-0.3281966	0.7686013	FXYD4
cg08244028	0.3248578	-352.7258	-0.3281984	0.6530562	MSH3
cg19982860	0.3011699	-352.7258	-0.3283057	0.6294757	IFNA21
cg10677144	0.3828415	-352.7258	-0.3284062	0.7112477	MYOM1
cg10322876	0.2947809	-352.7258	-0.3287002	0.6234811	CYP2B6
cg11719283	0.3286717	-352.7258	-0.3299251	0.6585968	ZNF574
cg04505023	0.3695696	-352.7258	-0.3301733	0.6997429	SPRR1A
cg06351503	0.3559565	-352.7258	-0.3306718	0.6866283	RDBP
cg06244417	0.4433347	-352.7258	-0.3314513	0.774786	FCN1
cg24490338	0.3882649	-352.7258	-0.3316314	0.7198963	TPM3
cg09299388	0.3654354	-352.7258	-0.331652	0.6970874	PGK2
cg00321478	0.3246931	-352.7258	-0.3318038	0.6564969	CRB1
cg06811800	0.34313	-352.7258	-0.3321946	0.6753246	ATP4B
cg19859270	0.3507019	-352.7258	-0.3322437	0.6829456	GPR15
cg13019092	0.4002278	-352.7258	-0.3325507	0.7327785	PDZK1
cg06832950	0.4426368	-352.7258	-0.3333757	0.7760125	SPG3A
cg02844051	0.402856	-352.7258	-0.3333786	0.7362346	ZD52F10
cg19728577	0.4393969	-352.7258	-0.3335593	0.7729563	GUCA2B
cg24992780	0.4628873	-352.7258	-0.335784	0.7986712	OR7C1
cg06436504	0.3334668	-352.7258	-0.336174	0.6696407	DOC1
cg10575735	0.264506	-352.7258	-0.3362779	0.6007839	SSX4
cg00334507	0.2876406	-352.7258	-0.3366624	0.6243029	MVP
cg14238120	0.3758136	-352.7258	-0.3368861	0.7126997	ELA3A
cg14182690	0.4535824	-352.7258	-0.337314	0.7908964	RUNX3
cg16462075	0.3874975	-352.7258	-0.3373244	0.7248219	MUC3B
cg10569414	0.3507375	-352.7258	-0.3387423	0.6894798	C21orf121
cg26063872	0.3036302	-352.7258	-0.3387739	0.6424041	DEFB123
cg16626670	0.3067236	-352.7258	-0.3394825	0.6462061	CLEC4G
cg25762706	0.2296134	-352.7258	-0.3396084	0.5692218	STMN4
cg09701102	0.4130652	-352.7258	-0.3400567	0.7531219	NDUFV1
cg09414535	0.2630463	-352.7258	-0.3406519	0.6036983	GRIP1
cg08684473	0.4068898	-352.7258	-0.3414935	0.7483833	LILRB5
cg13578652	0.293515	-352.7258	-0.3416146	0.6351296	UBASH3A
cg03364781	0.3393791	-352.7258	-0.3417831	0.6811622	ALPK1
cg19949550	0.3963192	-352.7258	-0.3425038	0.7388231	ASB2

cg08970446	0.4800559	-352.7258	-0.3425328	0.8225887	SLC1A7
cg16122592	0.2980184	-352.7258	-0.3425364	0.6405548	MAGEB6
cg09027725	0.2859382	-352.7258	-0.3434309	0.6293691	COX4I2
cg01515887	0.3850819	-352.7258	-0.3436621	0.7287439	SAA2
cg21372914	0.3703009	-352.7258	-0.3437272	0.7140282	CLEC4M
cg13204181	0.2871337	-352.7258	-0.3440327	0.6311664	GH1
cg15780361	0.2069186	-352.7258	-0.3444721	0.5513908	ALS2CR11
cg01015871	0.271356	-352.7258	-0.3453859	0.6167418	MT4
cg00698688	0.4582555	-352.7258	-0.345812	0.8040674	SULT2B1
cg22264436	0.400654	-352.7258	-0.3462388	0.7468928	SOST
cg14894144	0.1326733	-352.7258	-0.3467807	0.479454	LAMA3
cg06501790	0.3211504	-352.7258	-0.3468962	0.6680466	SLC34A1
cg19807685	0.3276089	-352.7258	-0.3480022	0.6756111	HSD17B2
cg04138756	0.432207	-352.7258	-0.3484236	0.7806306	SPRR3
cg12456510	0.3302532	-352.7258	-0.3493097	0.6795629	TFF2
cg12334759	0.3484569	-352.7258	-0.3495133	0.6979702	C19orf19
cg15711744	0.4125352	-352.7258	-0.3499882	0.7625234	ANP32D
cg04711324	0.2146701	-352.7258	-0.3504212	0.5650914	RIT2
cg06220755	0.2665477	-352.7258	-0.3515683	0.618116	RAI2
cg13521229	0.2011699	-352.7258	-0.3519498	0.5531197	JOSD2
cg18780284	0.3286064	-352.7258	-0.3527872	0.6813936	SPRR1B
cg08626653	0.3579938	-352.7258	-0.3530059	0.7109996	FLJ37538
cg14845091	0.2703709	-352.7258	-0.3542931	0.624664	ADPRHL1
cg21686987	0.421128	-352.7258	-0.3553501	0.7764781	CTRB1
cg26149550	0.4329793	-352.7258	-0.3555669	0.7885463	KLK15
cg19712821	0.2107486	-352.7258	-0.35605	0.5667985	KSP37
cg05671018	0.3217638	-352.7258	-0.3576182	0.679382	LYSMD2
cg19954000	0.1437485	-352.7258	-0.3582378	0.5019863	FGF1
cg12513481	0.2019454	-352.7258	-0.3583172	0.5602626	SCAP1
cg16990174	0.1886213	-352.7258	-0.3588667	0.547488	RYBP
cg05348870	0.4103637	-352.7258	-0.3590327	0.7693964	TNFSF14
cg25214346	0.2945686	-352.7258	-0.3595974	0.654166	NR1I3
cg17357062	0.4647044	-352.7258	-0.3610505	0.8257549	FCN1
cg14696820	0.2394022	-352.7258	-0.3615471	0.6009493	LCE1A
cg10990993	0.2366186	-352.7258	-0.3619145	0.5985331	MLH1
cg07997737	0.2083139	-352.7258	-0.3639787	0.5722926	NRTN
cg24670715	0.1353427	-352.7258	-0.3643004	0.4996431	ANGPT2
cg10758292	0.4024503	-352.7258	-0.3674083	0.7698587	DEFA1
cg10805676	0.3448183	-352.7258	-0.3685068	0.7133251	MRPL28
cg15503752	0.360881	-352.7258	-0.3688481	0.7297292	ST6GALNA C1
cg20373326	0.3116225	-352.7258	-0.3693771	0.6809996	HSD17B2
cg06101324	0.125056	-352.7258	-0.3703675	0.4954236	SPRR1A
cg23412777	0.269934	-352.7258	-0.3725881	0.6425221	PYGO1
cg03221619	0.1797764	-352.7258	-0.3735154	0.5532917	FCER2

cg07824742	0.3852971	-352.7258	-0.3737741	0.7590712	DBH
cg12489960	0.2942555	-352.7258	-0.3745919	0.6688474	SGCB
cg15928132	0.2321469	-352.7258	-0.3754201	0.607567	CCKAR
cg03602500	0.35479	-352.7258	-0.3759074	0.7306974	FLJ00060
cg12815142	0.299954	-352.7258	-0.3761719	0.6761258	SPAG7
cg19728223	0.1609787	-352.7258	-0.3773999	0.5383787	KCNQ1
cg16175263	0.1847211	-352.7258	-0.3782085	0.5629296	TNFRSF10C
cg16772207	0.2412168	-352.7258	-0.3790152	0.620232	MYT1
cg21948655	0.4169995	-352.7258	-0.3818741	0.7988736	SMCP
cg14706739	0.3101248	-352.7258	-0.3824512	0.6925761	EPB49
cg10062065	0.2730348	-352.7258	-0.3853163	0.6583511	APEG1
cg15652212	0.3076275	-352.7258	-0.3857479	0.6933754	FLJ90586
cg06639544	0.3525002	-352.7258	-0.3860151	0.7385153	OR7A5
cg20544605	0.2716411	-352.7258	-0.3860601	0.6577012	SORBS2
cg05444024	0.4657555	-352.7258	-0.3860934	0.8518489	FUT6
cg05766474	0.4313662	-352.7258	-0.3871159	0.818482	CCL16
cg06123346	0.2325662	-352.7258	-0.3874289	0.6199951	ATP4A
cg08450982	0.2010452	-352.7258	-0.3876922	0.5887374	NUMBL
cg17907567	0.2331067	-352.7258	-0.3886868	0.6217936	HAMP
cg22575540	0.3654611	-352.7258	-0.3903983	0.7558594	TRIM54
cg24024214	0.4307597	-352.7258	-0.3907897	0.8215494	BTNL8
cg25119415	0.2958256	-352.7258	-0.3921153	0.687941	MNDA
cg26292028	0.2968556	-352.7258	-0.3931316	0.6899872	FLJ37587
cg14256699	0.4664212	-352.7258	-0.3939674	0.8603887	SOST
cg26672426	0.231858	-352.7258	-0.3943669	0.626225	PTGES
cg18149207	0.3363262	-352.7258	-0.397025	0.7333511	RORC
cg24694549	0.2414251	-352.7258	-0.3988756	0.6403007	GRIP1
cg23815000	0.3258845	-352.7258	-0.4013108	0.7271953	LCN1
cg08573687	0.2775849	-352.7258	-0.4031323	0.6807172	TH
cg01335367	0.1201917	-352.7258	-0.4031591	0.5233509	C12orf34
cg06536578	0.2461314	-352.7258	-0.4040142	0.6501456	JPH4
cg21457804	0.3457561	-352.7258	-0.4047585	0.7505146	CT45-2
cg20154346	0.2303925	-352.7258	-0.4075936	0.6379861	RAI2
cg25221254	0.3149043	-352.7258	-0.4115398	0.7264441	ASAH3
cg14204735	0.05277788	-352.7258	-0.4133677	0.4661456	CYB561
cg22374142	0.0816729	-352.7258	-0.4160036	0.4976765	HSF4
cg01103730	0.2788593	-352.7258	-0.418511	0.6973703	IL20
cg20334738	0.2266694	-352.7258	-0.4222124	0.6488818	MAB21L2
cg26927807	0.2692144	-352.7258	-0.4244381	0.6936526	BTBD2
cg04144768	0.2401921	-352.7258	-0.4389108	0.6791029	DDC
cg14992108	0.2813344	-352.7258	-0.4389236	0.7202581	SNTB1
cg27619475	0.1166617	-352.7258	-0.4398244	0.5564861	SLC16A5
cg13726507	0.3676315	-352.7258	-0.4607867	0.8284182	CTAG2
cg25856811	0.2489789	-352.7258	-0.4689779	0.7179568	SPRR3

cg26164184	0.3354425	-352.7258	-0.4794727	0.8149152	FCN2
cg13021192	0.09783199	-352.7258	-0.5026841	0.6005161	CTSZ
cg16179125	0.1330246	-352.7258	-0.5101755	0.6432001	CTSZ

Supplementary table 3 – probes differentially methylated between cluster B and normal tissue

TargetID	Cluster B.AVG_Beta	Cluster B.DiffScore	Cluster B.Delta Beta	Normal.AVG_Beta	SYMBOL
cg13099330	0.4842643	346.7507	0.3202974	0.163967	RBP1
cg26509022	0.4911016	346.7507	0.2877263	0.2033753	ALDH1A3
cg07360692	0.5004826	346.7507	0.2766723	0.2238103	FLJ20032
cg15310873	0.65658	346.7507	0.273802	0.382778	C20orf85
cg06507244	0.7190354	346.7507	0.2725549	0.4464805	DHX32
cg23855989	0.4822389	346.7507	0.2627599	0.2194789	AQP5
cg27652350	0.3916925	346.7507	0.2601677	0.1315248	ALDH1A3
cg22392666	0.4241241	346.7507	0.2574953	0.1666288	FXYD7
cg16046376	0.6111804	346.7507	0.2557272	0.3554532	PC
cg19224278	0.5681682	346.7507	0.2532995	0.3148687	ALDH1A3
cg18818531	0.630201	346.7507	0.2471147	0.3830863	FOSL1
cg25936385	0.8055148	346.7507	0.2457882	0.5597265	FAIM2
cg13906813	0.6428428	346.7507	0.2436529	0.3991899	HLA-DPA1
cg22628873	0.6939286	346.7507	0.2411	0.4528286	GGT6
cg08624249	0.71727	346.7507	0.2382398	0.4790302	KIAA0889
cg06392241	0.3769304	346.7507	0.2358713	0.1410591	NUDT4
cg02676865	0.3579776	346.7507	0.2331076	0.12487	UBTD1
cg01333011	0.5348968	346.7507	0.2316751	0.3032217	PTHLH
cg02000005	0.367604	346.7507	0.2286451	0.1389588	CRIP1
cg00446235	0.3615425	346.7507	0.2285267	0.1330158	F11R
cg03271907	0.8130926	346.7507	0.2244358	0.5886568	MGMT
cg10521852	0.4628236	346.7507	0.2212826	0.241541	EDG4
cg01683883	0.317448	346.7507	0.2164093	0.1010387	CMTM2
cg01561916	0.2709986	346.7507	0.2155201	0.05547852	HAAO
cg17067528	0.2945692	346.7507	0.2128365	0.08173271	IER3
cg16517394	0.4155173	346.7507	0.2107866	0.2047307	TNFSF4
cg04369341	0.3909286	346.7507	0.2085526	0.182376	C20orf100
cg03001305	0.3226499	346.7507	0.2028558	0.1197941	STAT5A
cg01103836	0.8753666	346.7507	0.2004084	0.6749582	MYO9B
cg21604615	0.3572092	346.7507	0.2001896	0.1570196	SYTL1
cg03605761	0.592015	129.8385	0.234914	0.357101	RNF126
cg07285276	0.7507304	127.2488	0.2286515	0.5220789	RAPGEF1
cg06204948	0.4349718	127.2488	0.2086568	0.226315	MARK2
cg10150813	0.5270693	125.6838	0.2242339	0.3028354	KIAA0746

cg23696949	0.4522658	125.6838	0.2120227	0.2402431	LAMC2
cg25447894	0.4053745	125.6838	0.2018072	0.2035673	CSDC2
cg16363586	0.5361763	124.5603	0.2245668	0.3116095	BST2
cg26822175	0.8224487	124.5603	0.2094749	0.6129738	CRYBA4
cg20289949	0.4468058	122.1916	0.2081956	0.2386102	HAAO
cg14371590	0.5279977	114.4628	0.2162207	0.3117769	SLC26A1 0
cg20439022	0.5890222	112.8358	0.2205583	0.3684638	SLC16A8
cg17790333	0.7113694	112.13	0.2187409	0.4926285	CYP11A1
cg12177001	0.7358415	111.1609	0.2151031	0.5207384	IFI27
cg23668631	0.5825233	110.6471	0.2182415	0.3642818	CAMKK1
cg21716693	0.4875242	109.6252	0.2067116	0.2808126	CPNE5
cg27114026	0.7430398	109.4343	0.212241	0.5307989	ELA1
cg06285340	0.7981589	108.8752	0.2011554	0.5970035	CYP11A1
cg02490034	0.6152161	108.8564	0.2180409	0.3971752	MEST
cg18392482	0.7536374	107.2477	0.2082694	0.545368	AMDHD1
cg17686260	0.7103856	104.9564	0.2113963	0.4989893	MGMT
cg15544721	0.6912748	104.877	0.2128296	0.4784451	PPP1R9A
cg07236190	0.6969027	103.7169	0.2112966	0.4856061	AMDHD1
cg14897096	0.7658682	101.2339	0.2000257	0.5658425	PC
cg25580076	0.6805679	99.48838	0.208156	0.4724118	MRPL43
cg12125117	0.5952502	99.34595	0.2084572	0.386793	GPR97
cg25101936	0.7064518	99.16131	0.2058735	0.5005782	ZBTB16
cg24928687	0.7324516	97.37164	0.2012012	0.5312504	EPHX1
cg18172186	0.7216625	96.12093	0.2011699	0.5204926	KIAA1913
cg07251788	0.6907666	95.4754	0.2034234	0.4873433	CLTCL1
cg24670715	0.2947156	-95.41345	-0.2049274	0.4996431	ANGPT2
cg10275770	0.2347893	-99.98315	-0.2052656	0.4400549	ICAM2
cg22341310	0.2205973	-105.0623	-0.2087363	0.4293335	ZNF541
cg13021192	0.3870664	-106.6246	-0.2134497	0.6005161	CTSZ
cg19917856	0.2902949	-113.4073	-0.2225608	0.5128558	LOC3428 97
cg06536578	0.4220054	-129.8385	-0.2281402	0.6501456	JPH4
cg12513481	0.3163344	-139.9844	-0.2439282	0.5602626	SCAP1
cg01335367	0.2775647	-142.2894	-0.2457862	0.5233509	C12orf34
cg22189286	0.4556133	-149.7886	-0.2353394	0.6909527	HSPB8
cg14204735	0.1824096	-208.1352	-0.283736	0.4661456	CYB561
cg21949305	0.4451759	-238.1708	-0.2801127	0.7252886	ADORA2 A

Supplementary table 4 – probes differentially methylated between Malignant tumours and normal tissue

TargetID	Malign.AVG_Beta	Malign.DiffScore	Malign.Delta Beta	Normal medulla.AVG_Beta	SYMBOL
cg25856811	0.1918496	-353.1397	-0.5261072	0.7179568	SPRR3
cg16179125	0.121508	-353.1397	-0.5216921	0.6432001	CTSZ
cg13021192	0.08940586	-353.1397	-0.5111102	0.6005161	CTSZ
cg21457804	0.2417219	-353.1397	-0.5087927	0.7505146	CT45-2
cg26164184	0.3138765	-353.1397	-0.5010387	0.8149152	FCN2
cg13726507	0.3325259	-353.1397	-0.4958923	0.8284182	CTAG2
cg05444024	0.3666006	-353.1397	-0.4852483	0.8518489	FUT6
cg14992108	0.2360716	-353.1397	-0.4841865	0.7202581	SNTB1
cg05766474	0.337676	-353.1397	-0.480806	0.818482	CCL16
cg04144768	0.1990229	-353.1397	-0.48008	0.6791029	DDC
cg25221254	0.2509786	-353.1397	-0.4754656	0.7264441	ASAH3
cg24024214	0.3541081	-353.1397	-0.4674414	0.8215494	BTNL8
cg15538820	0.4530806	-353.1397	-0.4634036	0.9164841	OBP2B
cg17907567	0.1588891	-353.1397	-0.4629045	0.6217936	HAMP
cg25119415	0.2266518	-353.1397	-0.4612892	0.687941	MNDA
cg15711744	0.3049476	-353.1397	-0.4575758	0.7625234	ANP32D
cg26927807	0.2383257	-353.1397	-0.4553268	0.6936526	BTBD2
cg08424423	0.3511186	-353.1397	-0.4497589	0.8008775	CDSN
cg00698688	0.3552629	-353.1397	-0.4488046	0.8040674	SULT2B1
cg05348870	0.3242536	-353.1397	-0.4451429	0.7693964	TNFSF14
cg24694549	0.1952028	-353.1397	-0.4450979	0.6403007	GRIP1
cg08453096	0.381714	-353.1397	-0.4441783	0.8258924	ABCG5
cg12456510	0.2358742	-353.1397	-0.4436887	0.6795629	TFF2
cg21948655	0.356401	-353.1397	-0.4424726	0.7988736	SMCP
cg06123346	0.1808753	-353.1397	-0.4391198	0.6199951	ATP4A
cg16462075	0.2858849	-353.1397	-0.438937	0.7248219	MUC3B
cg15652212	0.2563163	-353.1397	-0.4370591	0.6933754	FLJ90586
cg20334738	0.2134055	-353.1397	-0.4354763	0.6488818	MAB21L2
cg14333565	0.357435	-353.1397	-0.4340316	0.7914666	NRTN
cg01103730	0.2664935	-353.1397	-0.4308768	0.6973703	IL20
cg04719766	0.3542481	-353.1397	-0.4296809	0.7839289	KCNQ1
cg17264470	0.4252936	-353.1397	-0.4290587	0.8543522	FGF21
cg22374142	0.06878583	-353.1397	-0.4288906	0.4976765	HSF4
cg01015871	0.1881558	-353.1397	-0.428586	0.6167418	MT4
cg16626670	0.2194524	-353.1397	-0.4267537	0.6462061	CLEC4G
cg26149550	0.3630838	-353.1397	-0.4254625	0.7885463	KLK15
cg26292028	0.2646607	-353.1397	-0.4253265	0.6899872	FLJ37587
cg12513481	0.1361822	-353.1397	-0.4240803	0.5602626	SCAP1
cg12815142	0.2526424	-353.1397	-0.4234834	0.6761258	SPAG7

cg06536578	0.2287474	-353.1397	-0.4213982	0.6501456	JPH4
cg08970446	0.4016043	-353.1397	-0.4209844	0.8225887	SLC1A7
cg27619475	0.136802	-353.1397	-0.4196841	0.5564861	SLC16A5
cg14256699	0.4409448	-353.1397	-0.4194439	0.8603887	SOST
cg15589427	0.3151931	-353.1397	-0.4193472	0.7345403	MUC4
cg08573687	0.2624885	-353.1397	-0.4182287	0.6807172	TH
cg21686987	0.3583098	-353.1397	-0.4181683	0.7764781	CTRB1
cg17829936	0.2673488	-353.1397	-0.4180931	0.6854419	TAAR5
cg18149207	0.3156386	-353.1397	-0.4177125	0.7333511	RORC
cg13578652	0.2178856	-353.1397	-0.4172439	0.6351296	UBASH3A
cg13928961	0.2845886	-353.1397	-0.4171962	0.7017848	K6IRS3
cg06639544	0.3216326	-353.1397	-0.4168827	0.7385153	OR7A5
cg03602500	0.3148318	-353.1397	-0.4158656	0.7306974	FLJ00060
cg20154346	0.2241253	-353.1397	-0.4138608	0.6379861	RAI2
cg03364781	0.2681359	-353.1397	-0.4130263	0.6811622	ALPK1
cg14204735	0.0544948	-353.1397	-0.4116508	0.4661456	CYB561
cg01774645	0.2713733	-353.1397	-0.4111406	0.682514	ARHGAP30
cg09542291	0.3586163	-353.1397	-0.4108144	0.7694307	SMCP
cg16772207	0.2098051	-353.1397	-0.4104269	0.620232	MYT1
cg20544605	0.247885	-353.1397	-0.4098162	0.6577012	SORBS2
cg20373326	0.271272	-353.1397	-0.4097276	0.6809996	HSD17B2
cg12489960	0.2596922	-353.1397	-0.4091552	0.6688474	SGCB
cg08450982	0.1796286	-353.1397	-0.4091088	0.5887374	NUMBL
cg01144251	0.324304	-353.1397	-0.4090076	0.7333116	KLK9
cg19859270	0.2742115	-353.1397	-0.4087341	0.6829456	GPR15
cg06501790	0.2594283	-353.1397	-0.4086183	0.6680466	SLC34A1
cg19561774	0.450701	-353.1397	-0.4085449	0.8592458	SLC22A2
cg23776892	0.3681794	-353.1397	-0.4084932	0.7766726	MAGEA1
cg10758292	0.3616349	-353.1397	-0.4082238	0.7698587	DEFA1
cg21065959	0.2506559	-353.1397	-0.4081246	0.6587805	LCE1E
cg19845843	0.302144	-353.1397	-0.4079224	0.7100663	CXorf20
cg19728577	0.3651664	-353.1397	-0.4077898	0.7729563	GUCA2B
cg07126559	0.3519461	-353.1397	-0.4073638	0.7593099	SGCG
cg22575540	0.3486045	-353.1397	-0.4072548	0.7558594	TRIM54
cg07997737	0.1653667	-353.1397	-0.4069259	0.5722926	NRTN
cg04816348	0.4607313	-353.1397	-0.4055363	0.8662676	CLEC4G
cg08475088	0.4432616	-353.1397	-0.4052371	0.8484987	NALP9
cg17741572	0.3925749	-353.1397	-0.4044648	0.7970396	CFB
cg01718139	0.3616614	-353.1397	-0.4041325	0.7657939	UNQ3033
cg18780284	0.2774399	-353.1397	-0.4039537	0.6813936	SPRR1B
cg01335367	0.1194096	-353.1397	-0.4039413	0.5233509	C12orf34
cg03221619	0.1505491	-353.1397	-0.4027427	0.5532917	FCER2
cg18920397	0.3002461	-353.1397	-0.4024632	0.7027093	LY9
cg26672426	0.2244904	-353.1397	-0.4017346	0.626225	PTGES
cg23815000	0.3258466	-353.1397	-0.4013486	0.7271953	LCN1

cg00727947	0.4717107	-353.1397	-0.4013401	0.8730508	LILRA5
cg18790143	0.2997488	-353.1397	-0.4011573	0.7009061	OTOS
cg17357062	0.4248715	-353.1397	-0.4008835	0.8257549	FCN1
cg15928132	0.20705	-353.1397	-0.400517	0.607567	CCKAR
cg14942312	0.2825689	-353.1397	-0.3997276	0.6822965	GPR119
cg20095587	0.3152015	-353.1397	-0.3995068	0.7147083	TREM2
cg16122592	0.2417291	-353.1397	-0.3988257	0.6405548	MAGEB6
cg19807685	0.2770931	-353.1397	-0.398518	0.6756111	HSD17B2
cg16175263	0.1646563	-353.1397	-0.3982733	0.5629296	TNFRSF10C
cg08684473	0.3505404	-353.1397	-0.3978429	0.7483833	LILRB5
cg14696820	0.2034238	-353.1397	-0.3975255	0.6009493	LCE1A
cg18121684	0.2844862	-353.1397	-0.3956144	0.6801006	SERPINB13
cg06220755	0.2228054	-353.1397	-0.3953106	0.618116	RAI2
cg23412777	0.2478148	-353.1397	-0.3947073	0.6425221	PYGO1
cg20649991	0.3407245	-353.1397	-0.3946531	0.7353776	LILRB5
cg14150666	0.4415726	-353.1397	-0.3938573	0.8354299	IL8RB
cg10569414	0.2957648	-353.1397	-0.393715	0.6894798	C21orf121
cg10062065	0.2646952	-353.1397	-0.393656	0.6583511	APEG1
cg14182690	0.3976696	-353.1397	-0.3932269	0.7908964	RUNX3
cg10575735	0.2076187	-353.1397	-0.3931652	0.6007839	SSX4
cg12061127	0.2759923	-353.1397	-0.3927724	0.6687647	WFDC9
cg07824742	0.3666477	-353.1397	-0.3924235	0.7590712	DBH
cg22264436	0.3548968	-353.1397	-0.3919961	0.7468928	SOST
cg14238120	0.3209153	-353.1397	-0.3917844	0.7126997	ELA3A
cg02844051	0.3449644	-353.1397	-0.3912702	0.7362346	ZD52F10
cg19368582	0.2562717	-353.1397	-0.3907607	0.6470323	MMRN2
cg18204685	0.2956881	-353.1397	-0.3904009	0.686089	BTD
cg11739626	0.309971	-353.1397	-0.3903894	0.7003604	AKT1S1
cg20822628	0.5395295	-353.1397	-0.3900813	0.9296108	GATA5
cg10990993	0.2086779	-353.1397	-0.3898552	0.5985331	MLH1
cg07297178	0.3714801	-353.1397	-0.3879373	0.7594174	CEACAM7
cg15743985	0.3192534	-353.1397	-0.3853406	0.704594	CD22
cg21495715	0.3301479	-353.1397	-0.384932	0.7150798	SLC5A10
cg24670715	0.1148898	-353.1397	-0.3847533	0.4996431	ANGPT2
cg25033144	0.3479971	-353.1397	-0.3846391	0.7326362	FLJ00060
cg18521925	0.510487	-353.1397	-0.3839245	0.8944114	SLC22A16
cg22021786	0.3278267	-353.1397	-0.3836038	0.7114305	WFDC8
cg25214346	0.2710718	-353.1397	-0.3830943	0.654166	NR1I3
cg14366490	0.2547488	-353.1397	-0.3828094	0.6375582	TXNL6
cg07441143	0.3081239	-353.1397	-0.3815116	0.6896355	SLURP1
cg16990174	0.1660908	-353.1397	-0.3813972	0.547488	RYBP
cg13521229	0.1717496	-353.1397	-0.3813701	0.5531197	JOSD2
cg00689010	0.273333	-353.1397	-0.3809897	0.6543227	NCSTN
cg10213812	0.4825747	-353.1397	-0.3804365	0.8630112	FOXN1
cg24992780	0.4185619	-353.1397	-0.3801093	0.7986712	OR7C1

cg06236276	0.3780407	-353.1397	-0.3800621	0.7581028	SLC22A2
cg09299388	0.3170849	-353.1397	-0.3800025	0.6970874	PGK2
cg21372914	0.3343245	-353.1397	-0.3797037	0.7140282	CLEC4M
cg11158374	0.3256954	-353.1397	-0.3793744	0.7050698	TFF2
cg08244028	0.2741447	-353.1397	-0.3789115	0.6530562	MSH3
cg06325687	0.5240554	-353.1397	-0.3784153	0.9024706	OPN1MW
cg05215575	0.1869235	-353.1397	-0.3781203	0.5650438	FLJ25410
cg08093398	0.274041	-353.1397	-0.3779274	0.6519684	PSF1
cg24027679	0.3988175	-353.1397	-0.3771464	0.7759639	SLC2A7
cg10322876	0.2463628	-353.1397	-0.3771183	0.6234811	CYP2B6
cg16545105	0.2804936	-353.1397	-0.3765678	0.6570615	CRHBP
cg04138756	0.4050808	-353.1397	-0.3755498	0.7806306	SPRR3
cg06101324	0.1201795	-353.1397	-0.3752441	0.4954236	SPRR1A
cg08471713	0.4603995	-353.1397	-0.3748561	0.8352556	MEOX1
cg21505334	0.3557376	-353.1397	-0.3743015	0.7300391	CEACAM5
cg25778166	0.3514925	-353.1397	-0.3733657	0.7248582	FMO3
cg15481539	0.4360782	-353.1397	-0.3732135	0.8092917	DEFA5
cg23131950	0.3497453	-353.1397	-0.3727145	0.7224598	AP2S1
cg19304352	0.4199971	-353.1397	-0.372569	0.7925661	DEFA4
cg10805676	0.3411797	-353.1397	-0.3721454	0.7133251	MRPL28
cg03609102	0.1991042	-353.1397	-0.3721277	0.5712318	MUC5B
cg00941549	0.2960889	-353.1397	-0.3719942	0.6680831	AKAP4
cg15379858	0.5172948	-353.1397	-0.3719416	0.8892364	ChGn
cg24355048	0.2658559	-353.1397	-0.3715847	0.6374406	CTSG
cg26264314	0.377984	-353.1397	-0.3714495	0.7494335	NALP5
cg16155702	0.3981431	-353.1397	-0.3712322	0.7693753	FGF21
cg11346450	0.3565485	-353.1397	-0.3702305	0.726779	UGT1A3
cg10883352	0.3023795	-353.1397	-0.3702238	0.6726032	
cg04891836	0.2747343	-353.1397	-0.3699147	0.644649	TNFSF14
cg19047670	0.2991976	-353.1397	-0.3694679	0.6686655	CCND1
cg12582965	0.4902818	-353.1397	-0.3685878	0.8588696	ATP10A
cg15516226	0.4298694	-353.1397	-0.3682695	0.7981389	BTNL9
cg01568736	0.3715624	-353.1397	-0.3681105	0.7396729	SERPINB7
cg19717150	0.2994847	-353.1397	-0.3678106	0.6672953	HNF4A
cg09414535	0.2360598	-353.1397	-0.3676385	0.6036983	GRIP1
cg11884243	0.4263784	-353.1397	-0.3667518	0.7931302	FCN2
cg08996986	0.3119959	-353.1397	-0.3661304	0.6781263	EPS8L1
cg23580945	0.3838131	-353.1397	-0.3660558	0.7498689	FLJ43826
cg15140807	0.3757119	-353.1397	-0.3659346	0.7416465	FLJ31222
cg20485165	0.2828671	-353.1397	-0.3652916	0.6481587	WFDC12
cg19954000	0.1371843	-353.1397	-0.3648021	0.5019863	FGF1
cg16986846	0.2279526	-353.1397	-0.3645191	0.5924717	SCGB2A1
cg17778867	0.3122911	-353.1397	-0.3640786	0.6763697	KRTAP10-8
cg10746737	0.3413668	-353.1397	-0.3638161	0.7051829	HLA-DRB5
cg04711324	0.2016328	-353.1397	-0.3634586	0.5650914	RIT2

cg05922591	0.4448559	-353.1397	-0.3632213	0.8080772	LILRB4
cg27157038	0.3870067	-353.1397	-0.3629737	0.7499803	DNTT
cg24735489	0.2616997	-353.1397	-0.362786	0.6244857	CDSN
cg09027725	0.2666847	-353.1397	-0.3626844	0.6293691	COX4I2
cg10370591	0.2568521	-353.1397	-0.3626566	0.6195087	TPO
cg01827098	0.4326444	-353.1397	-0.3624631	0.7951075	GIMAP7
cg09701102	0.3910062	-353.1397	-0.3621156	0.7531219	NDUFV1
cg13204181	0.2691233	-353.1397	-0.3620431	0.6311664	GH1
cg18138484	0.4637827	-353.1397	-0.362042	0.8258247	CABP2
cg15780361	0.1895959	-353.1397	-0.3617949	0.5513908	ALS2CR11
cg26063872	0.2806902	-353.1397	-0.3617139	0.6424041	DEFB123
cg25762706	0.2077741	-353.1397	-0.3614477	0.5692218	STMN4
cg15422147	0.3275714	-353.1397	-0.3608822	0.6884536	SERPINB5
cg26422060	0.4251677	-353.1397	-0.3608739	0.7860416	TBX10
cg14706739	0.3321406	-353.1397	-0.3604355	0.6925761	EPB49
cg10677144	0.3509827	-353.1397	-0.360265	0.7112477	MYOM1
cg04505023	0.3394846	-353.1397	-0.3602583	0.6997429	SPRR1A
cg00042156	0.2350968	-353.1397	-0.360195	0.5952918	MGC16291
cg08459368	0.435635	-353.1397	-0.3601331	0.7957681	SCGB2A1
cg14284171	0.4649712	-353.1397	-0.3594196	0.8243908	SSX4
cg27513764	0.3042786	-353.1397	-0.3592424	0.6635211	EFCAB3
cg24825722	0.2371101	-353.1397	-0.3586074	0.5957175	ACADVL
cg13053608	0.1524696	-353.1397	-0.3585205	0.5109901	LGP1
cg01515887	0.3704707	-353.1397	-0.3582732	0.7287439	SAA2
cg04431776	0.4440412	-353.1397	-0.3580958	0.802137	GAGE2
cg24750391	0.1312689	-353.1397	-0.3577673	0.4890362	PON3
cg27291231	0.3371188	-353.1397	-0.3576799	0.6947987	SSNA1
cg25072962	0.2680399	-353.1397	-0.3572944	0.6253342	MGC35295
cg24919884	0.3008333	-353.1397	-0.3571347	0.657968	ARHGEF16
cg10409560	0.4086342	-353.1397	-0.3571327	0.7657669	FLJ23657
cg12334759	0.3408964	-353.1397	-0.3570737	0.6979702	C19orf19
cg11113534	0.4990594	-353.1397	-0.3569139	0.8559733	C20orf70
cg06437862	0.40385	-353.1397	-0.3562804	0.7601305	TUBA2
cg13656062	0.3960565	-353.1397	-0.3560413	0.7520977	CYP4F2
cg20488657	0.3359535	-353.1397	-0.355344	0.6912975	TFF3
cg03104936	0.2834188	-353.1397	-0.3548747	0.6382935	GRB10
cg15503752	0.3760384	-353.1397	-0.3536908	0.7297292	ST6GALNAC1
cg13916742	0.4317627	-353.1397	-0.3536738	0.7854365	SCGB1D1
cg18242139	0.3597679	-353.1397	-0.3536429	0.7134107	ELAVL4
cg13126790	0.3261902	-353.1397	-0.3536042	0.6797944	FLJ27255
cg08314660	0.4942361	-353.1397	-0.3535194	0.8477555	PKP3
cg05485062	0.4250809	-353.1397	-0.3517736	0.7768545	SERPINA12
cg00075967	0.3638342	-353.1397	-0.351428	0.7152622	STRA6
cg00644033	0.5665978	-353.1397	-0.3509686	0.9175664	MUC3B
cg17788013	0.3470318	-353.1397	-0.3504829	0.6975147	SPINK5

cg15741706	0.3452654	-353.1397	-0.3497967	0.6950621	CXorf48
cg05556202	0.4084814	-353.1397	-0.3495379	0.7580193	TM4SF19
cg27377450	0.4576887	-353.1397	-0.3491192	0.8068079	ARHGEF18
cg00520135	0.5070312	-353.1397	-0.3484875	0.8555187	TPM1
cg06244417	0.4265561	-353.1397	-0.3482299	0.774786	FCN1
cg21168884	0.4030654	-353.1397	-0.348201	0.7512664	C6orf122
cg17298704	0.4519594	-353.1397	-0.3474759	0.7994353	CLDN18
cg22784047	0.3397102	-353.1397	-0.3468877	0.6865979	MVP
cg25781162	0.4075105	-353.1397	-0.3465078	0.7540183	ABCG5
cg25903122	0.4034972	-353.1397	-0.3456905	0.7491877	MGC2747
cg06832950	0.4306082	-353.1397	-0.3454043	0.7760125	SPG3A
cg11830061	0.5703696	-353.1397	-0.3453491	0.9157187	INSL6
cg25013053	0.3518655	-353.1397	-0.3453273	0.6971928	UNC45B
cg21003606	0.5454174	-353.1397	-0.3446	0.8900174	CALN1
cg13019092	0.3896125	-353.1397	-0.343166	0.7327785	PDZK1
cg11154879	0.4521337	-353.1397	-0.3430554	0.7951891	C20orf151
cg03712237	0.4411383	-353.1397	-0.3428108	0.7839491	SSX2
cg08341924	0.4160888	-353.1397	-0.3422374	0.7583263	TGM1
cg22190114	0.3733314	-353.1397	-0.3420501	0.7153814	NALP8
cg04520391	0.4054843	-353.1397	-0.3417842	0.7472685	PRB2
cg21624282	0.45813	-353.1397	-0.3401066	0.7982366	LOC122258
cg26473272	0.527793	-353.1397	-0.339596	0.867389	SYT8
cg26093687	0.43224	-353.1397	-0.3395364	0.7717764	EIF3S2
cg24824840	0.4507549	-353.1397	-0.3393541	0.790109	SHANK1
cg08728865	0.4523108	-353.1397	-0.3393003	0.7916111	NALP7
cg12619162	0.4295629	-353.1397	-0.3390384	0.7686013	FXYD4
cg04132607	0.5202248	-353.1397	-0.3388996	0.8591244	GATA5
cg23514672	0.534259	-353.1397	-0.3384185	0.8726774	FLJ32871
cg16242770	0.4246746	-353.1397	-0.3375463	0.7622209	KRTAP17-1
cg02601403	0.4323477	-353.1397	-0.3370304	0.7693781	TBC1D3C
cg03534410	0.5252028	-353.1397	-0.3364601	0.8616629	TMEM40
cg11015241	0.5250095	-353.1397	-0.3360053	0.8610147	ATP10A
cg13453139	0.4159389	-353.1397	-0.3359694	0.7519083	PIK3R5
cg06270401	0.4288664	-353.1397	-0.3359692	0.7648356	DYRK4
cg01309153	0.5129861	-353.1397	-0.3354988	0.8484849	SURF1
cg23753610	0.5948404	-353.1397	-0.33535	0.9301904	DNAHL1
cg05440289	0.4204902	-353.1397	-0.3342112	0.7547014	IVL
cg25177139	0.4601569	-353.1397	-0.3335743	0.7937313	SLC10A6
cg13608094	0.5319676	-353.1397	-0.3321618	0.8641294	CCND1
cg18809535	0.5157196	-353.1397	-0.3292099	0.8449295	LDHAL6B
cg07595943	0.5892391	-353.1397	-0.3275534	0.9167925	LOC161931
cg02442161	0.4258407	-353.1397	-0.3272864	0.7531271	PI3
cg10129493	0.4263587	-353.1397	-0.3272278	0.7535865	CD33
cg16607065	0.4440067	-353.1397	-0.3259121	0.7699188	TP73
cg07792737	0.4879497	-353.1397	-0.3243666	0.8123163	NPIP

cg21399079	0.5492972	-353.1397	-0.3242197	0.8735169	GPR45
cg21407055	0.4678544	-353.1397	-0.3189175	0.7867719	ART1
cg04450876	0.4658791	-353.1397	-0.3187135	0.7845926	FAM112B
cg26813458	0.5464146	-353.1397	-0.3186429	0.8650575	CEACAM6
cg07506795	0.5561472	-353.1397	-0.3170552	0.8732024	ZNF19
cg24908058	0.5238456	-353.1397	-0.3169711	0.8408167	CGB5
cg15627025	0.5392129	-353.1397	-0.3168082	0.8560211	KIR3DL1
cg24407065	0.4867082	-353.1397	-0.3165575	0.8032658	BLZF1
cg00601486	0.5768236	-353.1397	-0.3159121	0.8927357	H1T2
cg09691574	0.4869934	-353.1397	-0.3140952	0.8010886	MRGPRX4
cg18508525	0.4977302	-353.1397	-0.3140223	0.8117525	CD36
cg10334928	0.5186656	-353.1397	-0.3123267	0.8309923	STON2
cg22013966	0.5295085	-353.1397	-0.3114766	0.8409851	SERPINA13
cg18231267	0.5728946	-353.1397	-0.3110847	0.8839793	RUNX3
cg17894008	0.5097622	-353.1397	-0.3092588	0.819021	NACAL
cg27212977	0.52693	-353.1397	-0.307945	0.8348749	DEFA6
cg08124722	0.5340626	-353.1397	-0.3069769	0.8410395	CCL7
cg13181284	0.5355783	-353.1397	-0.3062599	0.8418382	KRT6B
cg12040555	0.5880759	-353.1397	-0.3044289	0.8925049	MGMT
cg22218909	0.5605991	-353.1397	-0.3032817	0.8638808	DEFA3
cg09508556	0.560279	-353.1397	-0.3009647	0.8612437	PSORS1C2
cg16016036	0.5311612	-353.1397	-0.3007822	0.8319434	TPO
cg00152644	0.520776	-353.1397	-0.3004503	0.8212262	SPRR2E
cg20543571	0.5406709	-353.1397	-0.3002366	0.8409076	C15orf43
cg00269932	0.5978572	-353.1397	-0.2987723	0.8966295	LAIR2
cg22039846	0.5580025	-353.1397	-0.2975183	0.8555208	KIR2DL1
cg22899145	0.6054332	-353.1397	-0.2968296	0.9022628	OPN1LW
cg13696012	0.5802678	-353.1397	-0.2959633	0.8762311	BPIL1
cg15096123	0.558532	-353.1397	-0.2959414	0.8544734	KLK4
cg06001166	0.5272968	-353.1397	-0.2957852	0.8230821	RPL3L
cg10938286	0.5493305	-353.1397	-0.2955545	0.844885	CST2
cg14297029	0.5492567	-353.1397	-0.2924601	0.8417169	SSTR3
cg25813714	0.5518429	-353.1397	-0.2917507	0.8435937	CYP4F12
cg09750183	0.5904374	-353.1397	-0.2885401	0.8789775	PGA5
cg20676303	0.5913581	-353.1397	-0.2879422	0.8793003	GAGE7B
cg27341860	0.6057094	-353.1397	-0.2856816	0.891391	OR2L13
cg25087423	0.6271483	-353.1397	-0.282452	0.9096003	BLR1
cg02910574	0.6158151	-353.1397	-0.2799762	0.8957913	PCOLN3
cg25514503	0.6119118	-353.1397	-0.2778454	0.8897572	PER3
cg05564657	0.6042902	-353.1397	-0.2759038	0.880194	AADAC
cg03846767	0.6211254	-353.1397	-0.2753859	0.8965113	TP73
cg14893129	0.5989105	-353.1397	-0.2753411	0.8742516	CARD14
cg09283007	0.6349209	-353.1397	-0.269445	0.9043659	FAM47B
cg08742106	0.6171443	-353.1397	-0.2673165	0.8844607	USP6
cg05384917	0.6365483	-353.1397	-0.2669822	0.9035305	GPR109B

cg25651984	0.6182711	-353.1397	-0.2657937	0.8840648	MAGEC1
cg07339327	0.6387529	-353.1397	-0.2651809	0.9039338	CCND1
cg11360149	0.6490521	-353.1397	-0.2615538	0.9106058	OR2V2
cg19971655	0.6335066	-353.1397	-0.2614112	0.8949178	BSND
cg27360282	0.6452697	-353.1397	-0.2583929	0.9036626	RUNX3
cg18604842	0.6423548	-353.1397	-0.2542655	0.8966204	FLJ36046
cg21643045	0.6408277	-353.1397	-0.2514121	0.8922398	CCL20
cg10719920	0.6436859	-353.1397	-0.251214	0.8948999	C21orf93
cg14365123	0.663397	-353.1397	-0.245508	0.9089049	ARPM2
cg15772361	0.6733456	-353.1397	-0.243422	0.9167676	SERPINB3
cg20059312	0.6725587	-353.1397	-0.2416248	0.9141835	NGEF
cg14645650	0.7042676	-353.1397	-0.2349712	0.9392388	RUNX3
cg26750319	0.7266508	-353.1397	-0.2326446	0.9592954	KCNQ1
cg07432969	0.6949706	-353.1397	-0.2265293	0.9214999	GPR35
cg26208930	0.7213792	-353.1397	-0.2241257	0.9455049	TP73
cg16825643	0.7154665	-353.1397	-0.2219012	0.9373677	FAM38A
cg20483763	0.7375296	-353.1397	-0.2145904	0.9521199	CABP4
cg17240454	0.4926659	-353.0258	-0.3072956	0.7999614	SPDEF
cg05800321	0.5403467	-352.7386	-0.290791	0.8311377	LY6D
cg14894144	0.1265585	-352.5416	-0.3528954	0.479454	LAMA3
cg02130905	0.2502714	-350.8302	-0.3565199	0.6067913	STMN4
cg06233503	0.2064612	-349.7787	-0.3575443	0.5640055	KCNQ1
cg01731341	0.4842712	-349.7325	-0.3090011	0.7932723	FGF6
cg18729973	0.4181969	-348.782	-0.32763	0.7458268	TFF1
cg03544379	0.1396938	-348.148	-0.3526261	0.4923198	OR7C2
cg22627427	0.5648825	-347.5702	-0.2802464	0.8451289	C11orf9
cg02423618	0.2356898	-347.2813	-0.3558266	0.5915165	SPATA8
cg20311730	0.2751103	-347.1521	-0.352964	0.6280743	NALP10
cg18986165	0.4912712	-346.9534	-0.3059312	0.7972023	SIGLEC12
cg11750883	0.203096	-346.7798	-0.3561811	0.5592771	C1orf42
cg09448880	0.2874843	-346.764	-0.3514891	0.6389734	PGLYRP3
cg02723372	0.4855635	-346.3501	-0.3075585	0.793122	RUNX3
cg03264414	0.5583675	-346.3462	-0.2823683	0.8407358	PAEP
cg26583078	0.3131535	-346.224	-0.3479506	0.6611041	SORBS2
cg27050793	0.5784721	-345.3047	-0.2743818	0.8528539	SBSN
cg27090216	0.03628109	-344.4484	-0.3218093	0.3580904	TNFRSF10C
cg05248781	0.5602388	-344.0608	-0.2810424	0.8412812	LCE5A
cg19787037	0.1942855	-343.351	-0.35447	0.5487555	SPAG11
cg01469547	0.5122277	-343.0413	-0.2978686	0.8100963	OR5P3
cg03931808	0.6384987	-341.1724	-0.2485366	0.8870353	RLN3
cg15484375	0.3750719	-341.0395	-0.3350037	0.7100756	SAA1
cg26306976	0.5653939	-340.7834	-0.2782051	0.843599	ITGB1BP1
cg03468463	0.2980134	-340.6226	-0.3477878	0.6458012	SERPINB12
cg04574507	0.301855	-340.0353	-0.3470714	0.6489264	CD1B
cg10052840	0.1459826	-339.5224	-0.3491424	0.4951251	SEMA6B

cg09546307	0.3321069	-339.0651	-0.3422896	0.6743965	CLEC4D
cg07339138	0.2221162	-335.8798	-0.3512369	0.5733532	CCDC13
cg27496506	0.4476597	-335.8798	-0.315497	0.7631567	TGM5
cg10127415	0.1761027	-335.8208	-0.3501468	0.5262495	MAGEB6
cg08477744	0.5888508	-335.6369	-0.2677497	0.8566005	MFAP2
cg19728223	0.1877859	-335.4621	-0.3505927	0.5383787	KCNQ1
cg00626119	0.1862851	-335.4266	-0.3505014	0.5367865	NTRK1
cg27065979	0.5099916	-335.4266	-0.2963477	0.8063393	NEK3
cg22194129	0.2387454	-335.2111	-0.350453	0.5891984	CLEC4C
cg20751395	0.5783061	-335.0291	-0.2716953	0.8500014	KCNQ1
cg10071275	0.2299695	-334.8189	-0.350571	0.5805405	MYT1
cg21148892	0.3727474	-334.674	-0.3331596	0.705907	CLEC4F
cg23260026	0.158566	-333.917	-0.34776	0.506326	FSTL3
cg06403553	0.4874102	-333.5167	-0.3030075	0.7904177	PGK2
cg03752885	0.1761474	-333.4886	-0.3490007	0.5251481	DAPK3
cg08495878	0.6032762	-333.0081	-0.2612755	0.8645517	SERPINA4
cg15823100	0.5861913	-332.3417	-0.2678993	0.8540906	PISD
cg04953015	0.3836903	-332.2814	-0.3299889	0.7136792	CHRNA2
cg19623751	0.269946	-331.7565	-0.3470205	0.6169665	CEACAM7
cg14162076	0.5474698	-331.7491	-0.2822836	0.8297534	CLEC4D
cg00321478	0.314736	-331.0704	-0.3417609	0.6564969	CRB1
cg14740251	0.3571407	-330.949	-0.3348112	0.6919519	SIGLEC5
cg19233472	0.3954623	-330.9107	-0.3268924	0.7223547	FOXI1
cg12339029	0.4008183	-330.9107	-0.32567	0.7264883	MYL1
cg13397379	0.2970143	-330.729	-0.3438739	0.6408883	OR2C3
cg12970084	0.340134	-330.7284	-0.337722	0.677856	ELF3
cg27442349	0.288689	-330.0425	-0.3445156	0.6332046	NFKBIB
cg23114594	0.3792172	-329.4581	-0.3298862	0.7091034	C10orf30
cg05921699	0.2894349	-329.0247	-0.3440132	0.6334482	CD79A
cg17142134	0.5822421	-328.7969	-0.2684582	0.8507003	SLC2A2
cg19712821	0.2188378	-328.7054	-0.3479607	0.5667985	KSP37
cg17981339	0.4541289	-328.4109	-0.3111713	0.7653002	SBEM
cg14519000	0.6265342	-327.3362	-0.2502124	0.8767466	GATA5
cg23829949	0.2344383	-327.1583	-0.3469852	0.5814235	ZNF238
cg00334507	0.2804125	-326.5976	-0.3438905	0.6243029	MVP
cg14902389	0.3972699	-326.1091	-0.3247434	0.7220133	MGAT4A
cg19584957	0.6829803	-325.7143	-0.2249513	0.9079316	TTLL10
cg13180098	0.3009604	-324.9408	-0.3410186	0.641979	RHO
cg05055150	0.415839	-324.722	-0.3199046	0.7357436	MAG
cg09837648	0.6105359	-324.4493	-0.2560717	0.8666076	PLXNB1
cg18223379	0.1749816	-324.1272	-0.3443228	0.5193044	BPIL3
cg24363955	0.4848307	-323.955	-0.3007347	0.7855654	FLJ14054
cg14826683	0.1957523	-323.0161	-0.3449351	0.5406874	SPRR2D
cg05700681	0.4927325	-322.9175	-0.29797	0.7907025	CCL22
cg24691255	0.5412006	-322.7146	-0.2818553	0.823056	SERPINB2

cg00463202	0.4638954	-321.3355	-0.3060501	0.7699456	ADPRHL1
cg18979223	0.3244635	-320.5774	-0.3361379	0.6606013	CDKN2B
cg05423257	0.6857328	-320.4113	-0.2224702	0.908203	ALPI
cg15210427	0.1727947	-319.8676	-0.3420376	0.5148324	CST9L
cg18129786	0.5226234	-319.4687	-0.2872437	0.8098671	ZNF445
cg10275770	0.1081539	-319.4328	-0.331901	0.4400549	ICAM2
cg02064402	0.4098586	-319.2558	-0.3193596	0.7292182	SLC6A18
cg00895324	0.3805142	-318.5893	-0.3255474	0.7060616	PCP4
cg24607398	0.1800867	-318.1659	-0.3417326	0.5218193	MLH1
cg08878744	0.1742053	-317.8564	-0.3411322	0.5153375	LCE1B
cg11719283	0.3234318	-317.8564	-0.335165	0.6585968	ZNF574
cg05912121	0.3252504	-317.4857	-0.3347519	0.6600023	TH
cg15905124	0.4675464	-317.3112	-0.3036449	0.7711913	MGC13034
cg15903395	0.3195446	-317.2043	-0.3354305	0.6549752	FLJ25369
cg03557698	0.6188056	-315.7769	-0.2504069	0.8692125	C1orf177
cg13758677	0.4383389	-315.6474	-0.3110402	0.7493791	GAGE4
cg15439078	0.6035519	-315.6261	-0.2565032	0.8600551	MYL3
cg22988566	0.5251462	-315.2609	-0.2850862	0.8102324	WFDC10B
cg21522797	0.5678004	-314.9108	-0.2699828	0.8377832	PLCG2
cg13960126	0.3049195	-314.8607	-0.3363082	0.6412276	CRB3
cg00392257	0.2628223	-314.8342	-0.3401129	0.6029352	ISG20L2
cg04488758	0.3283681	-314.8342	-0.3331997	0.6615677	USP44
cg08420900	0.6008282	-314.4862	-0.2572598	0.858088	LW-1
cg16964535	0.6908345	-314.2189	-0.2186796	0.9095141	DNAJC5G
cg03309967	0.2776801	-314.0094	-0.3386615	0.6163416	PSENEN
cg04567009	0.2518233	-313.9023	-0.3402848	0.5921081	FCGR3B
cg20283107	0.242698	-313.8516	-0.3406222	0.5833201	FAM91A1
cg03291145	0.3091391	-313.7477	-0.3353117	0.6444508	ARSF
cg05275605	0.6694773	-313.4337	-0.2281652	0.8976426	C21orf123
cg16175725	0.5128466	-312.9989	-0.288399	0.8012456	TCF1
cg07123548	0.5365896	-312.9365	-0.2804736	0.8170632	HIPK4
cg06811800	0.3456815	-312.7293	-0.329643	0.6753246	ATP4B
cg14120879	0.4472553	-312.6633	-0.3076191	0.7548744	DEFB105A
cg09207718	0.22655	-312.6202	-0.3403614	0.5669113	CYP1A2
cg01255591	0.5382189	-312.5575	-0.2797902	0.8180091	R3HDML
cg02351381	0.1315371	-312.1647	-0.3326574	0.4641945	C12orf34
cg18389810	0.5750351	-312.0031	-0.26643	0.8414651	C14orf8
cg01442426	0.466824	-311.8608	-0.3019623	0.7687863	XCR1
cg15531099	0.1968501	-311.7158	-0.339404	0.5362542	LCE1D
cg25915982	0.4769036	-311.5663	-0.2989625	0.7758661	GRB10
cg05615150	0.3974414	-311.5421	-0.3192534	0.7166948	ARPP-21
cg15975283	0.4293278	-311.4532	-0.3117801	0.7411079	SLC9A11
cg19006008	0.3025199	-310.4956	-0.3346729	0.6371928	F2RL3
cg04810997	0.3421519	-310.4956	-0.3292838	0.6714357	TAS2R60
cg18241647	0.5061952	-309.6785	-0.2894352	0.7956304	WFDC12

cg12188860	0.6006603	-309.5129	-0.2559221	0.8565824	TOP1MT
cg27418851	0.3284767	-309.0108	-0.3307337	0.6592104	MBL2
cg24678320	0.6430327	-308.8274	-0.2384765	0.8815092	FLJ38451
cg26581729	0.1236362	-308.2489	-0.3289911	0.4526273	NPDC1
cg24949488	0.3454688	-307.887	-0.3276871	0.6731559	DNTT
cg02903525	0.4677341	-307.7752	-0.300289	0.7680231	C5AR1
cg09458237	0.3717023	-306.6064	-0.3225797	0.694282	HSPA12B
cg24506604	0.3870794	-306.239	-0.3194098	0.7064892	LOC144501
cg00350478	0.2690348	-305.9206	-0.3356438	0.6046786	FRMD1
cg09044738	0.5255961	-305.7608	-0.2819049	0.807501	FAM12A
cg25710140	0.3681616	-304.72	-0.3224895	0.6906511	MID1
cg04705866	0.3723494	-304.1432	-0.3214805	0.6938299	GZMK
cg22506059	0.5863695	-304.0774	-0.2598734	0.8462428	CARD10
cg16673198	0.3060345	-304.0511	-0.3314861	0.6375206	CPNE4
cg12022621	0.2150075	-303.1851	-0.3357317	0.5507392	LAX1
cg03782727	0.601393	-303.0601	-0.2538226	0.8552155	FFAR1
cg21450627	0.5392098	-302.9344	-0.2764489	0.8156587	PSD4
cg06094150	0.5004902	-302.5623	-0.2888629	0.7893532	MT1B
cg08887581	0.3761463	-302.5563	-0.3201171	0.6962634	C1orf64
cg05822532	0.2057033	-301.8447	-0.3348199	0.5405232	ELN
cg14934821	0.5455812	-301.6287	-0.2738581	0.8194393	GPSM1
cg01072821	0.2970113	-301.5843	-0.3313552	0.6283666	UNQ9391
cg06958211	0.2635019	-301.3344	-0.3338595	0.5973614	PAK6
cg10248727	0.5323788	-301.3258	-0.2782302	0.810609	LCN1
cg08603768	0.328837	-300.8905	-0.3272447	0.6560817	WNT8A
cg21256656	0.3975263	-299.9553	-0.3147785	0.7123048	KLK6
cg19917856	0.1806572	-299.5529	-0.3321985	0.5128558	LOC342897
cg23278885	0.3886291	-299.5529	-0.316459	0.7050881	TGM6
cg24841244	0.4134916	-299.4522	-0.3110696	0.7245612	CD3D
cg10190509	0.4806731	-299.3223	-0.2936506	0.7743237	CCL16
cg00057593	0.2399878	-299.318	-0.3337769	0.5737647	GML
cg04484789	0.4384841	-298.7927	-0.3048444	0.7433285	KRT25B
cg24443136	0.6907479	-298.752	-0.2150425	0.9057904	ATP2B3
cg11070419	0.2146902	-298.417	-0.3333231	0.5480133	C4BPA
cg09864990	0.5109174	-298.3633	-0.2841962	0.7951136	GATA5
cg14264994	0.6314825	-298.3633	-0.2404914	0.8719739	CTCFL
cg26136776	0.4742827	-298.1844	-0.2950645	0.7693472	KLF1
cg15113803	0.6400504	-298.1844	-0.236908	0.8769584	RHO
cg05671018	0.3579564	-297.7798	-0.3214256	0.679382	LYSMD2
cg18967533	0.2067425	-297.4105	-0.3325795	0.539322	KLK6
cg03860768	0.3169232	-296.7597	-0.3269832	0.6439065	BLK
cg02882813	0.4696093	-296.6651	-0.295835	0.7654443	CST5
cg20312687	0.2909363	-296.5975	-0.3296559	0.6205922	DEFB118
cg06145357	0.3597003	-296.3667	-0.3205391	0.6802394	MAGEA8
cg00594952	0.2325984	-296.2392	-0.3323536	0.564952	RIMS3

cg27020690	0.05353852	-296.0718	-0.2996553	0.3531938	TERC
cg06277277	0.1576904	-296.0501	-0.3278572	0.4855476	NR1I3
cg04254119	0.5455532	-295.8837	-0.2720215	0.8175747	AKAP3
cg22407504	0.5680591	-295.383	-0.2639861	0.8320452	MUC5B
cg24857545	0.3572976	-295.2897	-0.3204865	0.6777841	GDI1
cg12385643	0.5618991	-294.8	-0.266	0.8278991	UGT1A6
cg19042947	0.3120283	-294.7924	-0.3266751	0.6387034	SERPINA4
cg24331162	0.4030116	-294.5627	-0.3114519	0.7144635	SYT8
cg25659818	0.2445192	-293.69	-0.3309232	0.5754424	CCL4
cg19982860	0.3023203	-293.5112	-0.3271554	0.6294757	IFNA21
cg17667972	0.5696692	-292.9973	-0.2626728	0.832342	KRT4
cg09237521	0.6273773	-292.7566	-0.2406039	0.8679812	IFITM2
cg07484827	0.1729667	-292.6528	-0.3277871	0.5007538	CHRNA10
cg21130124	0.5627694	-292.5722	-0.2649939	0.8277633	CALML5
cg07967308	0.3028381	-292.4249	-0.3266046	0.6294427	ACP5
cg10503138	0.4134004	-292.3277	-0.3082967	0.7216971	CNTN4
cg24477636	0.2319348	-292.0056	-0.3302434	0.5621782	OR10H1
cg18434152	0.3723929	-291.8256	-0.3164029	0.6887958	PROK1
cg08292050	0.4618224	-291.6512	-0.2961271	0.7579495	SOCS4
cg02192965	0.3745389	-291.6498	-0.3159341	0.690473	SLC3A1
cg00367281	0.3088431	-290.6748	-0.3251774	0.6340206	CHRNB3
cg26353877	0.4563722	-290.653	-0.2972056	0.7535778	APCS
cg14107638	0.3579342	-290.5877	-0.3183873	0.6763214	MAGEA5
cg21038703	0.3360829	-290.3551	-0.3216308	0.6577138	ASB16
cg15983538	0.4461034	-290.3238	-0.2997256	0.745829	SEMA4A
cg16739580	0.3078104	-290.19	-0.3250579	0.6328683	POP2
cg14911395	0.1477637	-290.181	-0.3231452	0.4709089	SEMA3B
cg03886110	0.3517158	-289.8704	-0.3190781	0.6707939	PECAM1
cg13726463	0.4360323	-289.8394	-0.3020359	0.7380682	COX6A2
cg26809968	0.5556958	-289.7321	-0.2665536	0.8222495	OPN1LW
cg27394486	0.5239003	-289.0362	-0.276921	0.8008213	C15orf2
cg19949550	0.4376332	-288.6436	-0.3011898	0.7388231	ASB2
cg00896220	0.3936315	-288.3469	-0.3108932	0.7045248	CCL4L2
cg16101800	0.42624	-287.8733	-0.3036138	0.7298539	PCK1
cg01204985	0.4609945	-287.8498	-0.2949347	0.7559292	LILRA4
cg19226099	0.2253562	-287.5638	-0.3279587	0.5533149	MC3R
cg14338548	0.5921156	-287.1561	-0.2526509	0.8447666	LALBA
cg15996947	0.292749	-286.6937	-0.3248956	0.6176445	L2HGDH
cg09467501	0.07122152	-286.5387	-0.3008916	0.3721131	PYY
cg22456522	0.1219951	-286.3629	-0.3160372	0.4380323	LILRB3
cg01413516	0.5240042	-286.2322	-0.2759372	0.7999414	INSL3
cg01643624	0.4229245	-286.156	-0.3037005	0.726625	C11orf16
cg04962134	0.2741401	-285.9575	-0.3259009	0.600041	TRIM51
cg01484156	0.2232	-285.6108	-0.3269247	0.5501247	NCALD
cg25193494	0.2821836	-285.2482	-0.3250339	0.6072175	FLJ20186

cg14179628	0.3172815	-285.0804	-0.3216713	0.6389529	TCEAL7
cg19111262	0.1985641	-284.4666	-0.325417	0.5239811	IGSF9
cg07597976	0.2594008	-284.4331	-0.3258935	0.5852943	CD19
cg13601079	0.4812854	-283.4394	-0.2877685	0.7690538	SSX3
cg00756887	0.3727656	-283.1886	-0.3126954	0.685461	PVRL4
cg15303841	0.2768767	-282.8339	-0.3242328	0.6011094	RFPL1
cg05093686	0.1563824	-282.7733	-0.3202585	0.4766409	MAB21L1
cg12951282	0.2872549	-282.1524	-0.3231899	0.6104448	ASGR2
cg06090864	0.63332	-282.0502	-0.2351785	0.8684985	FFAR1
cg01119135	0.6506495	-281.7461	-0.228022	0.8786716	C1orf116
cg26822241	0.3888538	-281.7045	-0.3090876	0.6979414	CYP2C9
cg01726767	0.2963167	-281.6501	-0.3222054	0.6185221	LALBA
cg00412805	0.6614091	-281.5157	-0.2234551	0.8848642	KBTBD5
cg15485859	0.4345184	-281.4644	-0.2991429	0.7336613	C1orf116
cg25552889	0.5143903	-281.4022	-0.2772759	0.7916662	C3orf40
cg24818418	0.6178559	-280.986	-0.2409822	0.8588381	EGF
cg18063149	0.3798459	-280.8559	-0.3104149	0.6902608	FMO3
cg12067261	0.4939349	-280.783	-0.2831749	0.7771097	INSL4
cg23873703	0.4977478	-280.4895	-0.2819558	0.7797037	KCNAB1
cg10848367	0.3371355	-280.4496	-0.3170646	0.6542001	SCGB1D2
cg07525077	0.249778	-280.3332	-0.3241291	0.5739071	RNASE3
cg22247240	0.2866	-280.2397	-0.3222989	0.6088988	C14orf115
cg21094154	0.4152481	-280.1492	-0.302999	0.7182471	TNFSF11
cg13733733	0.2635436	-280.0541	-0.3235411	0.5870848	LILRA3
cg20551517	0.4698443	-279.9203	-0.2895853	0.7594296	GIP
cg00603172	0.452023	-279.8592	-0.2942094	0.7462324	BOK
cg20657421	0.4253808	-279.8186	-0.3005974	0.7259783	CCL4
cg15447486	0.3951218	-279.5107	-0.3069197	0.7020414	GPR109B
cg23444894	0.447749	-279.2525	-0.2950413	0.7427903	UNQ5810
cg27132814	0.2732683	-279.1901	-0.3226308	0.5958991	C20orf79
cg17446142	0.464728	-279.0681	-0.2906166	0.7553446	GDF9
cg02537838	0.5247844	-278.6814	-0.2730695	0.7978539	C20orf151
cg05254747	0.4448709	-277.0305	-0.2948926	0.7397634	SLC39A14
cg05636175	0.120219	-276.8831	-0.3099307	0.4301497	TNFRSF10C
cg12683641	0.6052139	-276.8566	-0.2445847	0.8497986	KLK14
cg10853416	0.4022907	-276.7757	-0.3043402	0.7066309	MS4A7
cg06539449	0.4444254	-276.7246	-0.2948739	0.7392992	CCND1
cg23471482	0.5107865	-276.1992	-0.2765223	0.7873088	CCL22
cg00534856	0.6739477	-275.8404	-0.2165903	0.890538	CGB
cg23988567	0.4811639	-275.452	-0.2848222	0.7659861	
cg02737335	0.4061463	-275.3507	-0.3029554	0.7091017	ARHGEF16
cg06303238	0.337617	-275.2273	-0.3146165	0.6522335	SALL4
cg19875656	0.5177253	-275.2273	-0.2740592	0.7917845	TSP50
cg11435943	0.1990275	-274.7951	-0.3201871	0.5192146	SERPINB12
cg22220722	0.4920937	-274.554	-0.2814138	0.7735075	PLA2G2A

cg16379513	0.6262316	-274.3281	-0.2357324	0.861964	TSSK3
cg08555657	0.4531764	-274.2628	-0.2917395	0.7449159	SPRR2E
cg10705251	0.6221677	-274.1646	-0.2372684	0.8594362	ZNF683
cg16358738	0.4265771	-273.7511	-0.2978731	0.7244502	AGXT
cg12858514	0.6637642	-273.5866	-0.2202896	0.8840538	PADI3
cg00501366	0.2690464	-273.3433	-0.3199412	0.5889876	ALOX12B
cg11845202	0.5768731	-273.3233	-0.2538339	0.830707	K5B
cg19764418	0.2432153	-273.2296	-0.3205677	0.563783	RYR2
cg01772980	0.3067942	-273.168	-0.3171766	0.6239709	SCGB1D1
cg14845091	0.3082397	-271.8687	-0.3164243	0.624664	ADPRHL1
cg12113132	0.2278408	-271.8079	-0.3197479	0.5475886	CCNDBP1
cg15060813	0.1915449	-271.6338	-0.3178824	0.5094273	LRFN3
cg07728874	0.5631549	-271.5239	-0.2580142	0.8211691	CD3D
cg12850636	0.4158088	-271.1644	-0.2991675	0.7149763	TJP3
cg20584011	0.5579782	-270.9345	-0.2595768	0.817555	ZDHHC11
cg10490064	0.416858	-270.882	-0.2988228	0.7156807	CRYBB2
cg13694749	0.3781838	-270.8791	-0.3063736	0.6845574	SCN4A
cg04740359	0.1738479	-270.541	-0.3155118	0.4893597	NTF3
cg17687962	0.4452374	-270.1197	-0.2920408	0.7372782	KLK3
cg22228134	0.2625904	-269.9321	-0.3184589	0.5810493	GZMH
cg06946880	0.4159466	-269.7504	-0.2985435	0.7144901	ATP6V1B1
cg06142324	0.3199995	-269.6158	-0.3141266	0.634126	FLJ25530
cg20011352	0.06655951	-269.5324	-0.2878631	0.3544226	GPR124
cg16899306	0.3063021	-269.4678	-0.3154326	0.6217347	HLA-DQB2
cg04254916	0.6289424	-269.1213	-0.2331128	0.8620552	KRT5
cg03458191	0.462213	-268.9593	-0.2873544	0.7495673	SAA1
cg27235662	0.4724743	-268.8993	-0.2846561	0.7571304	CLDN16
cg25548825	0.4999415	-268.7503	-0.2769896	0.7769311	TBX10
cg06356454	0.6943426	-268.7236	-0.2058588	0.9002013	AQP12A
cg03782453	0.08867389	-268.715	-0.295709	0.3843829	FLJ90575
cg26918728	0.583741	-268.6897	-0.2498741	0.8336151	SEMA3B
cg22253945	0.5099686	-268.2018	-0.2738457	0.7838143	GPR45
cg03044435	0.4843026	-268.0616	-0.2811336	0.7654362	FLJ35816
cg25372195	0.3483286	-267.9003	-0.3097537	0.6580823	DCD
cg06351503	0.3824882	-267.564	-0.3041402	0.6866283	RDBP
cg18239253	0.2416315	-267.4139	-0.3175006	0.559132	DEFB32
cg09911342	0.551919	-267.0493	-0.2602633	0.8121823	ASZ1
cg23881725	0.1247693	-267.0098	-0.3049144	0.4296838	DLEC1
cg25400358	0.2456114	-266.8678	-0.3171935	0.5628049	GPR137
cg00344709	0.4988802	-266.8052	-0.2765587	0.7754389	ANKRD21
cg05670596	0.1744398	-266.7596	-0.3133763	0.4878161	CCRL2
cg18807515	0.3080736	-266.5872	-0.3138494	0.621923	PRAMEF2
cg17237881	0.6826226	-266.3007	-0.2102904	0.8929129	BIRC7
cg00750606	0.2229054	-266.2115	-0.31658	0.5394855	CDA
cg24541550	0.2567583	-266.1995	-0.3166658	0.5734241	MRVI1

cg23081213	0.3837045	-265.2551	-0.3028976	0.6866021	PRKAG3
cg19000186	0.3622676	-265.1003	-0.3063984	0.668666	CNGA1
cg07651914	0.2572291	-264.9127	-0.3159898	0.5732188	CLDN15
cg21519900	0.214151	-264.682	-0.3154155	0.5295665	C20orf186
cg18783781	0.4935446	-264.6097	-0.2772412	0.7707858	MGC4399
cg21958034	0.4991082	-264.5146	-0.2756274	0.7747356	MST1
cg22478614	0.2425748	-264.3591	-0.3158641	0.5584389	DEFB4
cg08972170	0.2408826	-264.2888	-0.3158228	0.5567054	Ells1
cg03608577	0.5009508	-264.0291	-0.2749143	0.7758651	OR12D3
cg20281815	0.689484	-263.7707	-0.206655	0.896139	PSCA
cg15626350	0.158094	-263.7547	-0.309368	0.467462	ESR1
cg01897036	0.5470238	-263.1692	-0.2605069	0.8075308	SCTR
cg01459162	0.5211238	-263.088	-0.2686089	0.7897327	PADI3
cg17405586	0.330025	-262.7445	-0.3096825	0.6397075	KRT1
cg00714377	0.1018046	-262.7059	-0.2960327	0.3978373	SLA2
cg02324920	0.2111299	-262.4981	-0.3140439	0.5251739	NEURL
cg25993152	0.4062282	-262.2642	-0.297326	0.7035542	XAGE5
cg03741352	0.4465556	-262.0864	-0.2884336	0.7349892	LCE3C
cg26862286	0.6194292	-261.9938	-0.2345892	0.8540184	NCL
cg01227741	0.4537117	-261.3979	-0.2864307	0.7401425	GIMAP7
cg26189983	0.06812957	-260.7947	-0.2826158	0.3507454	TNFRSF1B
cg24353217	0.4876325	-260.6299	-0.2773451	0.7649776	MYL2
cg11819637	0.3416641	-260.2551	-0.3070594	0.6487235	THPO
cg07440877	0.6462179	-260.2551	-0.2236346	0.8698525	FLJ46358
cg06985415	0.609262	-260.0679	-0.237802	0.847064	C10orf39
cg01663968	0.06344497	-260.0528	-0.2800726	0.3435176	CTSZ
cg00078867	0.3143547	-259.818	-0.3099217	0.6242764	WAS
cg02274362	0.5831261	-259.818	-0.2471537	0.8302798	PNMA6A
cg12547930	0.3043896	-259.6326	-0.3107223	0.6151119	WFDC6
cg22510822	0.3369053	-259.5505	-0.3073104	0.6442158	OR1E2
cg06275635	0.2456896	-259.3447	-0.313153	0.5588427	PGLYRP3
cg09995854	0.3686127	-259.3441	-0.3027408	0.6713535	IL1F8
cg08766149	0.3862954	-259.2494	-0.299729	0.6860244	GZMB
cg17191715	0.363722	-259.1514	-0.30341	0.667132	CA1
cg02833725	0.3470209	-258.2256	-0.3053758	0.6523967	ISG20L2
cg14287742	0.4683073	-258.2256	-0.2814891	0.7497964	BLZF1
cg06980460	0.4585793	-257.7181	-0.2837236	0.7423029	FOLH1
cg17501569	0.556118	-257.3205	-0.2554678	0.8115858	MSLN
cg24743310	0.6123194	-257.1492	-0.2357277	0.8480471	FLJ31196
cg26385286	0.1663054	-256.6999	-0.306358	0.4726635	GCNT2
cg27160701	0.2699981	-256.3446	-0.3110939	0.5810921	SBEM
cg02717866	0.3469588	-256.3327	-0.3044807	0.6514395	FLJ32771
cg20576002	0.2992653	-256.238	-0.30941	0.6086753	FAM112B
cg04048249	0.3545572	-255.9354	-0.3032512	0.6578084	APOC3
cg09300114	0.1616524	-255.7314	-0.3050796	0.466732	SLC16A5

cg00622552	0.09155175	-255.3118	-0.2878643	0.379416	ODF3L1
cg19384697	0.1926235	-254.1908	-0.3079602	0.5005838	UPK3B
cg19717326	0.1459543	-254.1178	-0.3013991	0.4473534	MYADM
cg07026910	0.4372698	-253.933	-0.2871588	0.7244287	INPP5D
cg04645174	0.4373072	-253.5016	-0.2869679	0.7242752	OR7A17
cg02989940	0.2647014	-253.3669	-0.3096768	0.5743782	ERAF
cg06531741	0.2264769	-252.9651	-0.3093477	0.5358245	HTR3B
cg12949760	0.4001812	-252.911	-0.2943497	0.694531	KCNQ1
cg16356516	0.5887648	-252.8408	-0.2428256	0.8315904	EXOSC6
cg12514506	0.591264	-252.6185	-0.2418705	0.8331345	OSBPL5
cg25020204	0.2692328	-252.1488	-0.308894	0.5781268	DBH
cg16483466	0.4053316	-252.1177	-0.2930122	0.6983438	C20orf186
cg24490338	0.4324582	-252.1177	-0.2874381	0.7198963	TPM3
cg25101056	0.393713	-252.1122	-0.2951706	0.6888835	KCNG4
cg08634024	0.5692983	-251.8622	-0.2491691	0.8184674	OR2F1
cg09931793	0.3967506	-251.7413	-0.2944483	0.6911989	OR2K2
cg24910675	0.1201222	-251.5923	-0.2939888	0.4141111	ENG
cg10784090	0.4992883	-251.5819	-0.2705746	0.7698629	CLDN18
cg26298099	0.5111067	-251.5078	-0.2671969	0.7783036	ESRRB
cg21312148	0.2221678	-251.4957	-0.3083351	0.5305029	LCE2D
cg10417559	0.5116758	-251.4448	-0.2670028	0.7786786	LMO6
cg09447105	0.6003067	-251.2108	-0.238167	0.8384738	PDE6H
cg07220939	0.3199084	-250.7599	-0.3048447	0.6247531	SLC22A12
cg02719634	0.5191706	-250.7361	-0.2645454	0.783716	SLC22A18AS
cg23244913	0.08521163	-250.6808	-0.282475	0.3676866	HCG9
cg21130374	0.258088	-249.8567	-0.3078908	0.5659788	MX2
cg23663653	0.4840793	-249.7078	-0.2739464	0.7580256	
cg11237817	0.2989385	-249.2472	-0.3058294	0.6047679	KIR3DL3
cg06489008	0.2457473	-249.124	-0.3075427	0.55329	CST11
cg18473117	0.4065917	-248.9801	-0.2913435	0.6979352	CCDC22
cg12775613	0.3258654	-248.9661	-0.3033426	0.6292081	HTR1F
cg18396533	0.1255145	-248.6165	-0.2934186	0.4189331	DYDC1
cg02981703	0.5585432	-248.4913	-0.2515113	0.8100545	CA6
cg17386181	0.4514601	-248.3221	-0.2814942	0.7329543	MT1B
cg04968473	0.2507459	-248.241	-0.3070539	0.5577998	CYP1A2
cg03386373	0.2626052	-248.0002	-0.3067951	0.5694003	SPATA3
cg24851490	0.1993787	-247.9807	-0.3048681	0.5042468	RNASE2
cg15782391	0.2619191	-247.696	-0.3066395	0.5685586	ACPT
cg24884084	0.1353997	-247.6876	-0.2951286	0.4305283	SPRR1B
cg18849169	0.4078099	-247.6513	-0.2904974	0.6983072	GPX3
cg23765993	0.178489	-246.9597	-0.3020803	0.4805694	SPINLW1
cg12907644	0.4747465	-246.6922	-0.2751217	0.7498682	SAA2
cg23587532	0.3188322	-246.6133	-0.3028324	0.6216645	BM88
cg03343942	0.23106	-246.4151	-0.3057452	0.5368052	SLC39A5
cg02706881	0.6504258	-246.4015	-0.2177653	0.8681911	C21orf123

cg07711097	0.3659909	-246.3535	-0.2969961	0.6629869	GML
cg00962799	0.4166714	-246.3286	-0.2881586	0.70483	SSX8
cg17699374	0.1617654	-246.289	-0.2992635	0.4610289	MGC35206
cg22022041	0.4743024	-246.0835	-0.2749692	0.7492716	CCR9
cg25058957	0.6308077	-246.0835	-0.2252288	0.8560365	RAXL1
cg17348429	0.5265357	-245.8989	-0.2604946	0.7870303	ENPP7
cg17095731	0.3431648	-245.7195	-0.2997484	0.6429132	LRP8
cg07017706	0.4329423	-245.6187	-0.284458	0.7174003	K6IRS3
cg15005385	0.2639212	-245.4926	-0.3053688	0.56929	CCL3L1
cg22088368	0.2286066	-245.4609	-0.3050956	0.5337022	MGC35206
cg03548857	0.1623071	-245.4506	-0.2988114	0.4611185	FFAR2
cg11939496	0.3611575	-245.0935	-0.2970676	0.6582251	CD244
cg18219418	0.455785	-244.4012	-0.2787793	0.7345642	PARP6
cg17582777	0.3179039	-243.8695	-0.3014906	0.6193945	EFNA3
cg13410437	0.4333952	-243.7642	-0.2835398	0.716935	OR5P2
cg20781967	0.1424013	-243.6178	-0.2940002	0.4364015	NINJ2
cg00466436	0.2103763	-243.5166	-0.3030354	0.5134117	DEFB126
cg10300154	0.3250486	-243.511	-0.3006242	0.6256728	MGC5297
cg25982743	0.1433324	-242.8203	-0.2936826	0.437015	TIMP4
cg18655584	0.5755368	-242.6942	-0.2437999	0.8193368	SLC22A18
cg05187322	0.659137	-242.5638	-0.2131367	0.8722737	CARD14
cg04086012	0.5549906	-242.4878	-0.2504338	0.8054244	FLJ36180
cg22484793	0.3971853	-241.9597	-0.289816	0.6870012	TLR9
cg19537511	0.260428	-241.6443	-0.3032894	0.5637174	ARHGEF15
cg26504906	0.1836447	-241.1563	-0.2991378	0.4827824	PRSS16
cg27069753	0.2217257	-241.0796	-0.3022648	0.5239905	ELA3B
cg06192619	0.685944	-240.3052	-0.2015894	0.8875334	SLC1A6
cg17229197	0.5231801	-240.2547	-0.2592903	0.7824704	KCNQ1
cg07173760	0.2103869	-240.2139	-0.3010799	0.5114667	CLC
cg17001430	0.4640868	-240.117	-0.2749643	0.7390511	KIF25
cg09736922	0.5355965	-240.0316	-0.2555193	0.7911158	THPO
cg01993576	0.107245	-239.8182	-0.2825115	0.3897566	SLC29A1
cg11161873	0.2336758	-239.5082	-0.3018261	0.535502	FLJ39575
cg21643191	0.3259125	-239.2328	-0.2983369	0.6242494	ABCB5
cg26111757	0.4001288	-239.2227	-0.288004	0.6881328	C20orf185
cg14550066	0.2324029	-239.1774	-0.3015884	0.5339913	NCR1
cg16998872	0.4866264	-239.1726	-0.2689203	0.7555467	GYPE
cg16792160	0.2570689	-239.0951	-0.3018766	0.5589455	ASAH2
cg07022477	0.2384506	-238.9283	-0.30161	0.5400606	HIF3A
cg08402568	0.3602382	-238.8625	-0.2941215	0.6543597	MGC34647
cg22294577	0.5554684	-238.8155	-0.2489072	0.8043756	SLC26A3
cg04457794	0.5242891	-238.8007	-0.2583795	0.7826686	CTSE
cg07073964	0.3699756	-238.537	-0.2925774	0.662553	PRSSL1
cg23458892	0.3593995	-238.5122	-0.2940549	0.6534544	SIGLEC7
cg01074640	0.3519725	-238.4889	-0.2950146	0.6469871	IFNA17

cg15423764	0.3629659	-238.215	-0.2934162	0.6563821	GLYAT
cg26062856	0.3213396	-237.615	-0.2979059	0.6192455	ATP10A
cg24388263	0.6520064	-237.5085	-0.2143621	0.8663685	FLJ10374
cg24198840	0.6861659	-237.216	-0.2005849	0.8867508	NEU2
cg04901273	0.4741273	-237.0913	-0.2712022	0.7453295	TBC1D3
cg11037787	0.5776351	-236.7724	-0.2409548	0.8185899	PLA2G2A
cg27383362	0.3626752	-236.2872	-0.2925015	0.6551768	ATAD3C
cg09142399	0.3637027	-236.1701	-0.2923	0.6560028	CRYZ
cg07446846	0.5363578	-236.0011	-0.2537156	0.7900734	SLC6A8
cg24552358	0.4051743	-235.7294	-0.2854326	0.6906068	ORM1
cg24030627	0.5293371	-235.5033	-0.2556016	0.7849387	FCGBP
cg17192247	0.08538153	-235.4816	-0.2719998	0.3573814	MAPRE3
cg06415153	0.2521867	-235.3919	-0.2997513	0.5519381	PITPNM2
cg19465374	0.5152912	-235.3919	-0.2595933	0.7748845	AZGP1
cg20573420	0.6456842	-234.9929	-0.2159968	0.861681	FLJ38451
cg02280309	0.3672827	-234.6622	-0.2910422	0.658325	PKLR
cg01375994	0.2555768	-234.543	-0.299274	0.5548508	MXRA5
cg03055440	0.3544769	-234.3852	-0.2926202	0.6470971	MS4A6A
cg24697329	0.4682893	-234.0034	-0.2712904	0.7395797	ARHGEF4
cg25645748	0.3511271	-233.9005	-0.2927921	0.6439193	AKAP14
cg21643361	0.2636186	-233.8223	-0.298816	0.5624346	ZNF135
cg20090497	0.2631778	-233.7645	-0.2987829	0.5619607	TAS2R9
cg24654350	0.4327385	-233.7645	-0.2791425	0.711881	KIR3DL1
cg03003745	0.2925084	-233.2111	-0.2975686	0.590077	UNQ473
cg05982504	0.3958939	-233.1518	-0.2858228	0.6817167	IGFALS
cg01053621	0.2697169	-233.0276	-0.2982647	0.5679817	APOA2
cg16507522	0.4991981	-232.592	-0.262847	0.7620451	SERPINA3
cg08886154	0.2479101	-232.0489	-0.297772	0.5456822	PAX4
cg00795812	0.113223	-232.0262	-0.278972	0.392195	PDCD1
cg11505080	0.1241696	-231.5812	-0.2817335	0.405903	GPR173
cg05973262	0.2171488	-231.2906	-0.2961322	0.513281	NOTCH4
cg20713492	0.06865302	-231.1362	-0.2619633	0.3306163	AQP10
cg24852661	0.1126102	-231.1268	-0.2781731	0.3907833	GOLPH2
cg08157292	0.2846843	-231.0796	-0.2967291	0.5814134	PPP1R7
cg19154438	0.5165108	-230.9505	-0.2574335	0.7739443	CKM
cg25214366	0.500765	-230.7452	-0.2616509	0.7624158	DEFB103A
cg03973663	0.1456271	-230.7166	-0.2862268	0.4318539	LYN
cg03021690	0.614049	-230.6108	-0.2262065	0.8402554	TNFAIP2
cg01632517	0.475194	-230.5793	-0.268108	0.743302	SSX8
cg20416179	0.540418	-230.2079	-0.2502071	0.7906252	C6orf71
cg25531166	0.5066288	-230.1177	-0.2598052	0.766434	CTAG1B
cg07408456	0.3120572	-230.0586	-0.2946055	0.6066627	PGLYRP2
cg11465372	0.5688382	-229.9571	-0.2412885	0.8101268	KRT2B
cg19421752	0.1202252	-229.9277	-0.2795165	0.3997418	SLC6A18
cg07115820	0.2809049	-229.7531	-0.2961007	0.5770056	EPX

cg05112299	0.2040084	-229.6712	-0.2941071	0.4981154	OR7A17
cg21970438	0.4949566	-229.6016	-0.262685	0.7576416	TTLL2
cg05019001	0.1708249	-228.8885	-0.2896016	0.4604265	AR
cg00556408	0.4519677	-228.783	-0.2727186	0.7246863	TMPRSS6
cg25168545	0.1936406	-228.6655	-0.2924549	0.4860955	GIMAP1
cg01637734	0.3248165	-228.5211	-0.2927312	0.6175476	CD5L
cg20305726	0.2457294	-228.3122	-0.295507	0.5412364	DEFB126
cg12682367	0.3200733	-228.3006	-0.293013	0.6130863	FLJ46358
cg09418321	0.1878713	-228.0159	-0.2913759	0.4792472	DYRK4
cg23173910	0.5086028	-227.8738	-0.2583209	0.7669237	ACTG2
cg22083047	0.1921601	-227.7874	-0.2917279	0.483888	PRICKLE2
cg14173523	0.3741659	-227.6771	-0.2865091	0.660675	FUT5
cg21961766	0.3948354	-227.4842	-0.2832057	0.6780411	PRRG2
cg24901474	0.2565571	-227.3565	-0.2950633	0.5516204	RGS5
cg15842430	0.3369415	-227.3142	-0.2909166	0.627858	FAM12B
cg02854090	0.5761593	-227.1555	-0.2378535	0.8140128	HIST1H2AA
cg25882366	0.1098603	-227.1525	-0.2745469	0.3844072	HOXB2
cg09426307	0.3594036	-226.7683	-0.2880454	0.647449	SEC14L3
cg13552869	0.1205277	-226.6941	-0.2773612	0.3978889	SEZ6L2
cg03684977	0.4428008	-226.3056	-0.2735574	0.7163582	GRB7
cg22971191	0.2235393	-225.972	-0.2932366	0.516776	SLC10A2
cg14102807	0.4175147	-225.8965	-0.2784176	0.6959323	CD19
cg23413307	0.3561753	-225.85	-0.2879703	0.6441457	LCE1F
cg15815843	0.5226995	-225.747	-0.2535286	0.7762281	MFAP5
cg06938878	0.5592317	-225.6973	-0.2426764	0.8019081	CALCB
cg22762309	0.4719102	-225.5553	-0.266656	0.7385662	OTUD6A
cg01031251	0.320868	-225.5465	-0.2914325	0.6123005	RPS6KA1
cg07879977	0.2321323	-224.7381	-0.29291	0.5250422	OR1F1
cg08952029	0.1827976	-224.7381	-0.2886154	0.471413	CHRDL2
cg07643942	0.2687108	-224.6467	-0.2934143	0.5621251	LACRT
cg03169180	0.3357258	-224.0921	-0.2893062	0.625032	NLGN2
cg15532088	0.6038756	-223.8051	-0.2273347	0.8312103	C17orf66
cg11554507	0.4519849	-223.4912	-0.2702709	0.7222558	NEUROD6
cg23680518	0.5839068	-223.4828	-0.2339332	0.81784	SBSN
cg06436504	0.3872287	-223.4754	-0.282412	0.6696407	DOC1
cg14308452	0.4445131	-223.3982	-0.2718302	0.7163433	MGC24975
cg15862544	0.6197882	-223.3266	-0.2215918	0.84138	EGFL4
cg12332316	0.6188246	-223.2189	-0.2218952	0.8407198	F12
cg14036856	0.2701641	-222.9837	-0.2924196	0.5625836	MGC52423
cg08411049	0.5168497	-222.8241	-0.2539288	0.7707785	SERPINB5
cg08859675	0.3428393	-222.7592	-0.2878516	0.6306909	PDE4A
cg26523005	0.2559232	-222.6097	-0.2922292	0.5481524	ZNF662
cg14578030	0.5162574	-222.4007	-0.2539105	0.7701679	FGF4
cg04245402	0.2086149	-222.31	-0.2898705	0.4984854	C19orf21
cg03330516	0.3340566	-222.114	-0.2883847	0.6224413	SRMS

cg10968815	0.4329671	-221.9495	-0.2735136	0.7064807	BPIL1
cg00548060	0.410025	-221.7446	-0.2777433	0.6877683	NPL
cg15210999	0.35205	-221.6914	-0.2862535	0.6383035	DTNBP1
cg06818777	0.09212146	-221.5824	-0.2645674	0.3566888	CHAD
cg18533225	0.41195	-221.563	-0.2773058	0.6892558	KLHDC7B
cg19370451	0.5452097	-221.4324	-0.2452322	0.7904419	CRNN
cg07072643	0.2689972	-221.428	-0.2915028	0.5605	EMR3
cg09229960	0.2928557	-221.3871	-0.2908379	0.5836935	EMD
cg10779183	0.4792335	-221.2399	-0.2629474	0.7421809	ELA3A
cg24489015	0.3046856	-221.1859	-0.29012	0.5948056	LPO
cg22789545	0.5006495	-221.1691	-0.2575383	0.7581878	C20orf114
cg02028524	0.6634381	-221.1617	-0.2046595	0.8680977	ATXN3
cg07412254	0.3447646	-221.1029	-0.2867334	0.631498	FLJ14816
cg13271951	0.2345003	-221.0961	-0.2907441	0.5252445	FAM57B
cg24489034	0.4144485	-221.0416	-0.2765822	0.6910307	LW-1
cg16084788	0.6254402	-220.6908	-0.2186283	0.8440685	SLC2A7
cg09038914	0.4937997	-220.6755	-0.2590759	0.7528756	GFAP
cg03752087	0.4085552	-220.4943	-0.2773606	0.6859157	CASP14
cg23065097	0.1582245	-220.4062	-0.2817763	0.4400008	FKBP1B
cg00684178	0.4605868	-220.3606	-0.2668793	0.7274661	NEU4
cg13439299	0.5961191	-220.2391	-0.2286323	0.8247514	DNAJC5G
cg04491443	0.6675867	-220.0877	-0.2026995	0.8702862	PDILT
cg13771579	0.6061524	-219.8698	-0.2250896	0.831242	TCL1B
cg16272420	0.4522213	-219.8671	-0.2684902	0.7207115	PNLIPRP2
cg01883966	0.5294439	-219.7025	-0.2490749	0.7785187	STK19
cg22189286	0.4152595	-219.5568	-0.2756932	0.6909527	HSPB8
cg08626653	0.4401063	-219.5568	-0.2708933	0.7109996	FLJ37538
cg11068096	0.5390049	-219.4667	-0.2462299	0.7852348	KRT14
cg09841009	0.4959419	-219.4555	-0.2579812	0.7539231	GYPA
cg21754343	0.3054993	-219.1672	-0.2889008	0.5944	LCE2B
cg00436603	0.2584782	-219.1381	-0.2901208	0.5485989	CYP2E1
cg02164442	0.3007594	-218.9318	-0.2890243	0.5897837	ITGAD
cg20256783	0.6598619	-218.8015	-0.2052434	0.8651053	K6HF
cg08834018	0.2290725	-218.7237	-0.2889901	0.5180627	PRODH
cg14141399	0.2186015	-218.4182	-0.2881478	0.5067493	HAS1
cg14015044	0.04777583	-218.3614	-0.2419591	0.289735	TNFRSF10C
cg03364504	0.636477	-218.2646	-0.2137796	0.8502566	FLJ36116
cg21207418	0.4672542	-218.2579	-0.264379	0.7316332	ACP5
cg21277505	0.6038913	-218.2136	-0.2252394	0.8291306	LOC284361
cg05131835	0.3717189	-218.0681	-0.2818187	0.6535376	GH2
cg09863066	0.1511486	-218.0445	-0.2787459	0.4298946	PVALB
cg05446471	0.6196816	-218.0289	-0.2197045	0.8393861	HDAC11
cg14321743	0.3644699	-218.0176	-0.2827367	0.6472065	PLA2G2D
cg27214365	0.5011694	-217.972	-0.255971	0.7571404	GYPB
cg13435189	0.6182107	-217.8285	-0.2201458	0.8383565	CWF19L2

cg19464944	0.256107	-217.7886	-0.2892613	0.5453683	FCGR1A
cg04323365	0.4839736	-217.7648	-0.2601905	0.744164	GJB1
cg08256781	0.4104481	-217.7555	-0.2756259	0.686074	ACSBG2
cg22545356	0.4725065	-217.6207	-0.2628558	0.7353623	MMRN2
cg18429742	0.3007054	-217.479	-0.2881654	0.5888708	ZDHHC11
cg14871138	0.3383225	-217.3247	-0.2852862	0.6236088	NTSR1
cg15337006	0.3983825	-217.3104	-0.2774345	0.675817	ITGAM
cg13407883	0.3022049	-217.3042	-0.2879822	0.5901871	SIGLEC9
cg06821120	0.2142455	-217.2285	-0.2870281	0.5012736	RASSF1
cg06720660	0.3163566	-217.0491	-0.2869658	0.6033224	RNASE6
cg19618706	0.1702103	-216.7712	-0.2813985	0.4516088	BGN
cg07977490	0.4144174	-216.7497	-0.2744123	0.6888297	C16orf45
cg13859324	0.2137955	-216.714	-0.2866595	0.500455	UNC45B
cg12417466	0.4518083	-216.6933	-0.2670405	0.7188488	ARPP-21
cg21639401	0.3847884	-216.5639	-0.2791523	0.6639407	FLJ31222
cg06385087	0.04814024	-216.43	-0.2406396	0.2887798	CTSZ
cg03993463	0.6262782	-216.1393	-0.2166783	0.8429564	KCNJ15
cg10894512	0.3506856	-216.0513	-0.2832931	0.6339787	ACTA2
cg01325515	0.5492007	-215.6299	-0.2416252	0.7908258	CTAG2
cg17542495	0.2917974	-215.454	-0.2873658	0.5791631	GJB1
cg07093661	0.5329809	-215.1396	-0.2461187	0.7790996	DHDH
cg03818682	0.3468862	-215.1209	-0.2831933	0.6300795	MUC5AC
cg02658251	0.3676327	-215.0732	-0.2807359	0.6483686	DEFB4
cg12069042	0.403605	-214.9972	-0.2753806	0.6789856	PLXNB1
cg12266049	0.5978611	-214.9397	-0.2260193	0.8238803	CCND1
cg17910564	0.2926601	-214.5518	-0.2867998	0.57946	VDAC3
cg13882988	0.5152222	-214.5494	-0.2507509	0.7659731	MBL2
cg24628744	0.1322151	-214.487	-0.2718567	0.4040718	H2AFY
cg05547500	0.6062328	-214.1445	-0.222903	0.8291358	TXNDC2
cg02097420	0.3764686	-213.9208	-0.2789411	0.6554097	HRG
cg25712380	0.437848	-213.8713	-0.2685513	0.7063993	GRAP2
cg18490846	0.2395928	-213.8395	-0.2863701	0.5259629	C17orf73
cg02554564	0.3701921	-213.7088	-0.2796643	0.6498564	NTF3
cg21570818	0.4426547	-213.3624	-0.2673353	0.70999	FUT5
cg14034870	0.2979006	-213.296	-0.2858385	0.5837391	SFTPG
cg11297236	0.2240314	-213.2573	-0.2851793	0.5092107	PDILT
cg26014197	0.4038215	-213.1771	-0.2744016	0.6782231	ZNF206
cg20189782	0.4424737	-213.1575	-0.2672662	0.7097399	MGC27121
cg09948350	0.5567961	-213.1165	-0.2383121	0.7951082	FLJ25084
cg26420196	0.1904603	-212.799	-0.281666	0.4721264	GAS6
cg12435792	0.2204922	-212.5984	-0.2845083	0.5050005	PDE6B
cg20047055	0.3907171	-212.3933	-0.2760676	0.6667847	KLRC1
cg22340747	0.4184859	-212.3152	-0.2714315	0.6899173	GATM
cg01581111	0.429196	-212.245	-0.2694191	0.6986151	RBM18
cg22534509	0.1817819	-212.0446	-0.2799808	0.4617628	GPR81

cg25677709	0.4840421	-211.7502	-0.2573949	0.7414371	NDST1
cg14898892	0.254415	-211.7415	-0.2854797	0.5398947	SHRM
cg18806980	0.3698857	-211.5862	-0.2785521	0.6484378	KIAA0703
cg04273431	0.1223607	-211.0216	-0.2667077	0.3890684	PRR3
cg04645843	0.3893785	-210.6833	-0.2753671	0.6647456	DPCR1
cg23807646	0.3135351	-210.678	-0.2834687	0.5970038	SLC26A8
cg06132342	0.2999262	-210.6302	-0.2841682	0.5840943	KRTHB5
cg03473518	0.06432478	-210.4514	-0.2443962	0.308721	GJB6
cg25477904	0.5768809	-210.3504	-0.2310135	0.8078945	PSG1
cg18884741	0.5939192	-209.9977	-0.2254128	0.819332	RABGEF1
cg18294257	0.1205186	-209.9592	-0.2654069	0.3859255	SEC14L3
cg03352153	0.4265451	-209.8949	-0.268731	0.6952761	GLULD1
cg21541083	0.5754427	-209.5801	-0.2311534	0.8065962	STXBP2
cg15602735	0.4175596	-209.5078	-0.2701603	0.6877199	MAGEC2
cg02813121	0.6049834	-209.4615	-0.2215398	0.8265232	S100A12
cg13770446	0.6061788	-209.3844	-0.2211087	0.8272875	CLCNKA
cg08763351	0.4945419	-209.3152	-0.2537103	0.7482522	SPRR4
cg20423977	0.4606914	-209.2823	-0.2615469	0.7222383	PLAC4
cg21432842	0.2543805	-209.2441	-0.2839068	0.5382873	CSF3
cg03453449	0.333849	-209.1591	-0.2810691	0.6149181	USP44
cg14587868	0.3690902	-209.0028	-0.2772351	0.6463253	TGM1
cg20692181	0.3346261	-208.7502	-0.280769	0.6153951	RETN
cg25827666	0.4787128	-208.6175	-0.2571739	0.7358867	NTRK1
cg27285599	0.5128479	-208.6175	-0.2487326	0.7615805	FLJ13841
cg04189838	0.4586864	-208.5619	-0.2616247	0.7203111	CYP2C19
cg24777950	0.3926003	-208.4012	-0.2736537	0.666254	CTSG
cg23749046	0.5500888	-208.088	-0.2381896	0.7882785	GPR61
cg16601385	0.3467226	-207.93	-0.2791691	0.6258917	CFD
cg19055231	0.04524419	-207.7798	-0.2322975	0.2775417	STAC
cg23812886	0.249594	-207.5028	-0.2826886	0.5322825	SSX5
cg15542496	0.4696707	-207.2472	-0.2585847	0.7282554	PIP
cg24352688	0.3382676	-207.0901	-0.2794965	0.6177641	OFD1
cg22202141	0.3206251	-206.7655	-0.2807022	0.6013273	FCGR3A
cg10368842	0.3367075	-206.7655	-0.2794467	0.6161542	C10orf81
cg26796190	0.04386351	-206.7058	-0.230664	0.2745275	PYY
cg01970325	0.2556352	-206.6841	-0.2823118	0.5379469	NELF
cg17217677	0.370855	-206.6214	-0.2756976	0.6465526	SMPD3
cg24429836	0.18339	-206.5732	-0.276472	0.459862	LDHD
cg22805308	0.2420615	-206.4644	-0.2817524	0.5238139	PLEKHG5
cg05810550	0.4476985	-206.3721	-0.2628351	0.7105336	DEFB106A
cg18982568	0.3114197	-206.337	-0.2810141	0.5924338	KRT1B
cg25391023	0.2451082	-206.2235	-0.2817125	0.5268207	BTNL2
cg02187357	0.3581567	-205.8701	-0.2767877	0.6349444	TBC1D22B
cg09258965	0.3796247	-205.4494	-0.2739084	0.6535331	TBC1D7
cg09914304	0.5579316	-205.4428	-0.23476	0.7926916	PRF1

cg24888049	0.1824806	-205.2662	-0.2754379	0.4579185	FES
cg04713352	0.2953579	-204.8568	-0.2808626	0.5762205	ATP4A
cg04637372	0.3237093	-204.8417	-0.2793501	0.6030594	FLJ32784
cg12970081	0.2949416	-204.6622	-0.2807566	0.5756982	GPR32
cg09528351	0.4252292	-204.6495	-0.2662694	0.6914986	PIK3R5
cg06625767	0.5301371	-204.5399	-0.2422783	0.7724154	F12
cg23350580	0.5160019	-204.3969	-0.2459926	0.7619945	TBC1D3C
cg15905634	0.448384	-204.2064	-0.261611	0.7099949	TAS2R60
cg17091851	0.2470384	-204.1998	-0.280479	0.5275174	LOC348174
cg08784110	0.2590638	-204.1487	-0.2807533	0.5398171	MAS1
cg06906435	0.2554299	-204.1487	-0.2806833	0.5361133	FLJ25773
cg19553721	0.5304711	-204.012	-0.2419479	0.772419	FAM106A
cg05569220	0.3003169	-203.9659	-0.2801218	0.5804387	FLJ44861
cg24765446	0.3430396	-203.9572	-0.2772365	0.6202761	WFDC6
cg21453309	0.1626897	-203.6203	-0.2709268	0.4336165	FAM101A
cg00415993	0.1781762	-203.5125	-0.2735534	0.4517296	F2RL2
cg19784470	0.555299	-203.2334	-0.2345872	0.7898862	OR7C2
cg07895149	0.03691868	-203.2321	-0.2237634	0.260682	FAM26B
cg16377880	0.3187779	-203.0977	-0.2786342	0.5974121	CYP4F3
cg23240895	0.5595254	-203.0596	-0.233266	0.7927914	PRR5
cg07371530	0.5949175	-202.9678	-0.222285	0.8172026	RPUSD1
cg01987509	0.3062662	-202.6938	-0.2790841	0.5853504	PGR
cg11291009	0.1570269	-202.6732	-0.2691254	0.4261523	ARHGEF9
cg04337944	0.2024918	-202.6304	-0.2762073	0.4786991	FBLN1
cg24866437	0.4717939	-202.603	-0.2558327	0.7276267	ALPK1
cg14532417	0.5127263	-202.3617	-0.2458987	0.758625	TBC1D3
cg03891191	0.4572923	-202.2486	-0.2587836	0.716076	PRAMEF2
cg18524091	0.6356575	-202.2486	-0.2082567	0.8439142	TAS1R2
cg04623837	0.06373513	-202.1993	-0.2376911	0.3014262	HCG9
cg18530716	0.0912517	-202.1879	-0.2496949	0.3409466	SLC16A11
cg05241571	0.6263514	-202.1838	-0.2114735	0.8378248	UNQ467
cg00518911	0.2355487	-201.8637	-0.278418	0.5139667	HOXA10
cg16791508	0.4662484	-201.5187	-0.2565099	0.7227583	KRTHB3
cg18534730	0.3674215	-201.4317	-0.2731915	0.6406131	CABP5
cg18271969	0.6437663	-201.1057	-0.2049577	0.848724	HTR3C
cg04515986	0.1944599	-200.9325	-0.2740746	0.4685345	FTHL17
cg26884581	0.134738	-200.8703	-0.2626445	0.3973826	PYGM
cg02067021	0.424185	-200.7444	-0.2643948	0.6885798	DNAJC5B
cg13424446	0.5996746	-200.6559	-0.2198125	0.8194871	GGTLA4
cg19353006	0.3388029	-200.6248	-0.2756587	0.6144616	TUSC3
cg05873268	0.4759046	-200.5136	-0.2538834	0.7297881	TPSAB1
cg01305625	0.250491	-200.5054	-0.2781722	0.5286632	PDLIM4
cg20994561	0.5842282	-200.2785	-0.2245769	0.808805	BAI1
cg21518208	0.2945707	-200.0726	-0.2779149	0.5724856	KRTHB5
cg02173484	0.2551284	-199.9543	-0.2779503	0.5330787	GJA5

cg09626634	0.2612333	-199.7977	-0.2779787	0.539212	EBI2
cg20862119	0.5630982	-199.7112	-0.2307667	0.7938649	KRT2B
cg05955301	0.2341515	-199.2929	-0.2766333	0.5107849	PRELP
cg07531356	0.3917746	-199.1343	-0.268714	0.6604886	INSL6
cg27285056	0.4339686	-199.096	-0.2617579	0.6957265	NAPSA
cg21723486	0.3122382	-199.0016	-0.2765257	0.5887639	TP73L
cg21985470	0.2577315	-198.9783	-0.2773783	0.5351098	PKLR
cg05246522	0.331798	-198.8572	-0.275177	0.606975	KSR1
cg21707816	0.2288848	-198.7213	-0.275911	0.5047958	ENDOGL1
cg04311964	0.5230588	-198.7147	-0.2415192	0.764578	LYPD2
cg09923671	0.4183588	-198.2975	-0.2640992	0.682458	GATA5
cg01078434	0.5075714	-198.2016	-0.2452562	0.7528276	MAS1L
cg02847500	0.1444089	-198.1743	-0.2630647	0.4074737	SOX3
cg14324675	0.3351567	-198.1306	-0.2744793	0.609636	LST1
cg08474603	0.3992715	-198.1224	-0.2670481	0.6663196	CRP
cg21402035	0.3271441	-198.1035	-0.2750595	0.6022036	GALR3
cg18414950	0.1837281	-197.9738	-0.2704985	0.4542266	PDK3
cg10210238	0.184134	-197.8539	-0.2704746	0.4546087	CDKN2B
cg25778479	0.129091	-197.604	-0.2587011	0.3877921	ANKMY2
cg04037732	0.1559189	-197.3795	-0.2650607	0.4209796	NLGN3
cg11885098	0.1335142	-197.2147	-0.2596132	0.3931274	EFNA2
cg09037813	0.3913808	-197.0551	-0.2676066	0.6589874	LRRFIP1
cg24499411	0.3939739	-196.9846	-0.2671967	0.6611706	TNS3
cg26349773	0.4983152	-196.8056	-0.246857	0.7451722	ATP6V0A4
cg05358404	0.1333051	-196.7548	-0.2592109	0.392516	RTEL1
cg16542081	0.5290354	-196.6716	-0.2389917	0.7680271	GATA5
cg21484834	0.6139711	-196.5832	-0.2134865	0.8274575	PTK6
cg24898863	0.1296834	-196.5449	-0.2580678	0.3877513	S100A8
cg10612997	0.4086127	-196.438	-0.2646763	0.673289	GREB1
cg01135626	0.3099557	-196.3586	-0.2750005	0.5849562	CDX4
cg00819362	0.5324635	-196.2975	-0.2378981	0.7703615	CLIPR-59
cg01917648	0.2497685	-196.2021	-0.2753057	0.5250742	SPIC
cg25691167	0.04195161	-195.6657	-0.2207357	0.2626873	FERD3L
cg16446783	0.3879758	-195.5927	-0.2672534	0.6552292	MRGPRX4
cg02812142	0.3801998	-195.5337	-0.2682492	0.6484491	ACMSD
cg19841506	0.09996095	-195.3418	-0.2476998	0.3476608	ZMYND15
cg18098286	0.3555917	-195.2787	-0.2709302	0.6265219	UGT1A10
cg08430598	0.6080027	-195.2718	-0.2149247	0.8229274	CST1
cg02981853	0.5137917	-195.2575	-0.2422867	0.7560784	ARHGEF18
cg01657380	0.4054251	-195.1218	-0.2644471	0.6698722	NPFF
cg10345936	0.3973171	-195.0792	-0.2656386	0.6629558	SLC36A2
cg22193702	0.5528733	-194.9725	-0.2316486	0.7845219	PADI1
cg04365980	0.3602373	-194.9189	-0.2702298	0.6304671	CLEC3A
cg05606799	0.4380791	-194.8968	-0.2587646	0.6968437	KISS1
cg15670863	0.4004276	-194.8936	-0.2650712	0.6654989	SPACA4

cg23683201	0.395941	-194.8388	-0.2656973	0.6616384	SLC22A9
cg03379131	0.0574137	-194.7118	-0.2285568	0.2859704	ADAM15
cg02124291	0.4536272	-194.6631	-0.2556376	0.7092648	OR7A5
cg02311163	0.3444277	-194.6208	-0.2715818	0.6160095	SEMG2
cg13833831	0.5451831	-194.5322	-0.2336056	0.7787886	MATK
cg24816866	0.5029756	-194.3671	-0.2445348	0.7475104	PARK2
cg02797569	0.3025372	-193.8923	-0.2737619	0.5762991	PCOLCE
cg18462653	0.1448469	-193.746	-0.2598755	0.4047224	DEFB119
cg22937804	0.2643264	-193.7308	-0.2740504	0.5383769	MGC44505
cg18303397	0.1951233	-192.9213	-0.2685293	0.4636526	MBD4
cg05078019	0.2636152	-192.6768	-0.2733538	0.536969	PDE6B
cg19756068	0.3646923	-192.6763	-0.2684359	0.6331282	CYP2B6
cg20932053	0.4617846	-192.3153	-0.2527761	0.7145607	CPM
cg19592945	0.1599911	-192.2575	-0.262151	0.4221421	P2RXL1
cg01000094	0.5907457	-192.2531	-0.2191833	0.809929	
cg19096475	0.3523556	-192.1987	-0.2694162	0.6217718	ASAM
cg25957124	0.2545372	-192.0163	-0.2726817	0.5272189	DNAH3
cg01353448	0.1237319	-191.9194	-0.2528718	0.3766038	C7orf16
cg04833845	0.3494225	-191.2783	-0.2691457	0.6185682	KCNN4
cg20822579	0.163535	-190.9607	-0.2619207	0.4254557	RIPK3
cg21697779	0.1458386	-190.9407	-0.2580374	0.4038761	FUNDC2
cg07892051	0.3765464	-190.8579	-0.2660105	0.6425568	AKAP3
cg09712066	0.2605272	-190.5541	-0.2718851	0.5324123	PART1
cg14721213	0.2996592	-190.5179	-0.2717293	0.5713885	FMO2
cg16466334	0.3926902	-190.4196	-0.2636652	0.6563554	MMP3
cg21129531	0.6086334	-190.3557	-0.2127182	0.8213516	LRRC4
cg20104776	0.2947943	-190.3118	-0.2717213	0.5665156	LDOC1
cg18056600	0.1157624	-190.285	-0.2491779	0.3649402	ZMYND15
cg09425228	0.4092717	-190.2506	-0.2611404	0.6704121	CCL20
cg04893119	0.1729592	-190.23	-0.2631458	0.436105	PI15
cg06825166	0.2630236	-190.166	-0.2716686	0.5346922	TMEM10
cg26390526	0.5379673	-189.9315	-0.2334725	0.7714398	FLG
cg10379687	0.1664592	-189.9249	-0.2617106	0.4281698	SPINLW1
cg15916061	0.2780756	-189.9115	-0.2716499	0.5497255	SLC17A4
cg00918005	0.6001456	-189.8363	-0.2152097	0.8153554	REG3G
cg26954174	0.1695098	-189.8307	-0.2622135	0.4317232	CARD15
cg10837843	0.1058447	-189.8042	-0.2454843	0.351329	DUSP1
cg19372178	0.203829	-189.6805	-0.2673007	0.4711297	TMEM16G
cg13283751	0.1018698	-189.582	-0.243902	0.3457718	GPX5
cg26309498	0.57252	-189.3937	-0.2234724	0.7959924	EDAR
cg02988947	0.2772728	-189.2157	-0.271185	0.5484578	LIMD2
cg15648315	0.4680343	-189.1788	-0.2498348	0.717869	FLJ26443
cg24517042	0.5950937	-189.1063	-0.2164841	0.8115779	CAV3
cg03387497	0.5486198	-189.0081	-0.2301297	0.7787495	C20orf179
cg24340657	0.3821978	-188.9686	-0.2641847	0.6463825	KRT24

cg13792279	0.6216349	-188.8702	-0.2078385	0.8294734	CLDN17
cg27329371	0.2811586	-188.86	-0.2709361	0.5520948	ALDH3A1
cg15329483	0.3278261	-188.7763	-0.2692637	0.5970898	SSX7
cg19033555	0.3168219	-188.247	-0.269567	0.5863889	DEFB1
cg15895197	0.1444672	-188.0706	-0.2555158	0.399983	EMILIN1
cg20401945	0.1268258	-188.0104	-0.2507658	0.3775916	ASPHD1
cg22970435	0.1126291	-187.8062	-0.2462223	0.3588514	SPATS1
cg20795863	0.4403925	-187.651	-0.2544398	0.6948323	NEU2
cg10125195	0.435105	-187.5501	-0.2553348	0.6904398	LACRT
cg13603551	0.4619253	-187.5501	-0.2502385	0.7121638	ABP1
cg25383242	0.636734	-187.5257	-0.2022196	0.8389536	CNTNAP4
cg21846488	0.1496335	-187.5162	-0.2563191	0.4059526	LCE4A
cg03574571	0.5234925	-187.4537	-0.2360731	0.7595656	CD22
cg26391080	0.4771285	-187.4062	-0.246974	0.7241026	SH2D4B
cg15495837	0.5019926	-187.4062	-0.2413417	0.7433342	TRIM43
cg20514061	0.2042657	-187.1856	-0.2655533	0.469819	MID2
cg09001777	0.3832434	-187.0038	-0.2628967	0.6461401	FUT3
cg13281868	0.401174	-186.9807	-0.2604667	0.6616407	C6orf142
cg12536534	0.118301	-186.8185	-0.247263	0.365564	G6PD
cg12992720	0.2000777	-186.8018	-0.2647579	0.4648356	EDG4
cg23213217	0.2886654	-186.5647	-0.2693592	0.5580245	DEGS1
cg05546044	0.289497	-186.5007	-0.2693052	0.5588021	MAPK1
cg00476577	0.08318712	-186.1728	-0.2338716	0.3170587	ZNF217
cg09256683	0.299118	-186.0936	-0.2688506	0.5679686	CCL14
cg25839227	0.3436241	-186.0725	-0.2664319	0.610056	ABI3
cg21209356	0.4324562	-186.062	-0.2549706	0.6874267	CSF2RB
cg01367992	0.3218325	-185.8857	-0.2677934	0.5896258	LY9
cg04405541	0.5993639	-185.8311	-0.213748	0.8131119	C20orf114
cg21365235	0.1810999	-185.8203	-0.2612693	0.4423692	OCRL
cg12943082	0.5045133	-185.627	-0.2398513	0.7443646	CCL26
cg07443748	0.3082674	-185.6058	-0.2682362	0.5765036	CESK1
cg06226384	0.1494388	-185.5711	-0.2547889	0.4042277	CACNG5
cg17170504	0.3303871	-185.4319	-0.2669897	0.5973768	HSPC065
cg05600717	0.07640045	-185.3054	-0.2301993	0.3065997	FLJ13639
cg26628847	0.2374603	-185.2634	-0.267205	0.5046653	PIP
cg26218269	0.2228687	-184.9866	-0.2659045	0.4887733	MAB21L2
cg24311282	0.6030508	-184.9164	-0.2121986	0.8152493	SIGLEC11
cg20782689	0.5919561	-184.8594	-0.2156209	0.807577	OXA1L
cg04349727	0.3605692	-184.7503	-0.2640857	0.6246549	KLK5
cg01837574	0.2671176	-184.7066	-0.2680393	0.535157	TRAPPC1
cg21624359	0.2133052	-184.6153	-0.2647111	0.4780163	FFAR3
cg08642068	0.4696039	-184.5652	-0.2470703	0.7166742	SPAG4L
cg11599505	0.5328135	-184.4523	-0.2322147	0.7650282	C20orf102
cg19486673	0.1917373	-184.3527	-0.2618292	0.4535665	LILRA2
cg23047271	0.09914307	-184.2144	-0.238644	0.3377871	PRICKLE2

cg24310246	0.4841552	-184.0491	-0.2436975	0.7278527	RAXL1
cg07608333	0.2425347	-183.77	-0.2664674	0.5090021	CD209
cg27622610	0.2421099	-183.7466	-0.2664249	0.5085348	OR1G1
cg11834681	0.5249583	-183.568	-0.2338051	0.7587633	CYP2A13
cg15952487	0.2266112	-183.3671	-0.2650713	0.4916825	CD1B
cg20261167	0.1578572	-183.2531	-0.254923	0.4127803	SPP1
cg23338993	0.4015157	-183.1375	-0.2581852	0.6597009	UGT1A6
cg18881269	0.3840072	-182.9353	-0.2603816	0.6443889	LEPREL2
cg25141490	0.2377208	-182.8718	-0.2655412	0.503262	IL17B
cg08695223	0.2650516	-182.8064	-0.2666976	0.5317491	SLC9A6
cg26065841	0.533332	-182.642	-0.2312067	0.7645386	CHAC1
cg12552392	0.3585198	-182.5908	-0.2629563	0.6214761	NFS1
cg02825709	0.4881676	-182.4422	-0.2419778	0.7301454	ZNF75A
cg13530039	0.2671522	-182.4259	-0.2664812	0.5336334	CHRM1
cg14511156	0.2281513	-182.3826	-0.2644895	0.4926409	OSCAR
cg03916421	0.1694483	-182.3285	-0.2565772	0.4260255	LOC132321
cg05654163	0.3689068	-182.047	-0.2615744	0.6304811	SLC39A2
cg07922606	0.2305563	-181.9886	-0.2644	0.4949563	HIST1H3E
cg22563697	0.4648918	-181.7333	-0.2465158	0.7114077	PPP1R16A
cg19447966	0.5177283	-181.6885	-0.2346773	0.7524056	TEAD1
cg11263296	0.08003377	-181.6528	-0.228853	0.3088868	CCDC64
cg07745725	0.3710933	-181.4975	-0.2610013	0.6320946	PSG3
cg24235633	0.5908841	-181.3684	-0.2144129	0.805297	CDIPT
cg08109646	0.3651298	-181.3225	-0.2615187	0.6266485	ZNF683
cg21122774	0.4907382	-181.0324	-0.2406688	0.7314069	SARDH
cg00319692	0.2361266	-180.9559	-0.2640699	0.5001965	ATP6V0D2
cg18221897	0.4442658	-180.9332	-0.25001	0.6942758	KIR2DL1
cg02579133	0.418952	-180.4924	-0.2540438	0.6729958	KRTAP10-8
cg19403023	0.286268	-180.3768	-0.2652347	0.5515027	TESSP1
cg08191854	0.1046144	-180.2178	-0.2374512	0.3420657	TRPM2
cg26415633	0.2069324	-180.0598	-0.2606835	0.4676159	KLK1
cg18113270	0.6263433	-180.0358	-0.2026521	0.8289954	MMP3
cg11733245	0.4270356	-179.9895	-0.2524563	0.6794919	IL2RA
cg11151665	0.5748645	-179.9521	-0.2185227	0.7933872	PSG6
cg04806409	0.495049	-179.9385	-0.2391297	0.7341787	TFF3
cg10432859	0.3816145	-179.7769	-0.2587565	0.6403711	UGT1A7
cg03329572	0.44064	-179.7315	-0.2499846	0.6906246	FCRL5
cg10408410	0.4658926	-179.6743	-0.2451923	0.711085	RLBP1
cg21434954	0.3879972	-179.6503	-0.2579005	0.6458977	LCE1B
cg21458041	0.3653717	-179.3911	-0.2602949	0.6256666	TNP2
cg09492887	0.1097757	-179.2647	-0.2384829	0.3482586	SLC26A5
cg18356799	0.2607601	-179.2167	-0.2640782	0.5248383	DSC1
cg07207789	0.3321264	-179.2084	-0.2628865	0.5950129	CRISPLD2
cg25195673	0.2316896	-179.2084	-0.2624797	0.4941693	GFPT1
cg01868128	0.3555326	-178.9833	-0.2609662	0.6164987	LCE5A

cg18085517	0.2791873	-178.9717	-0.264255	0.5434423	TRPM1
cg08260891	0.07290064	-178.6632	-0.223179	0.2960796	PPGB
cg00280894	0.1762456	-178.5701	-0.254976	0.4312215	NXF2
cg11219178	0.5053056	-178.5439	-0.2360446	0.7413502	OSBPL5
cg05248470	0.224186	-178.339	-0.2612159	0.4854019	LILRB2
cg26946769	0.5588534	-178.0973	-0.2221791	0.7810324	MAPK4
cg06259570	0.3641248	-177.9901	-0.2595381	0.6236629	MMP27
cg02992596	0.2966986	-177.8412	-0.2633901	0.5600887	MGC27016
cg09871043	0.2179843	-177.7904	-0.2602181	0.4782025	PKHD1
cg11456838	0.2818794	-177.6334	-0.263348	0.5452273	LOC202459
cg01550148	0.2840911	-177.6185	-0.26334	0.5474311	H2AFY
cg25098401	0.2575034	-177.5782	-0.262802	0.5203053	LCE2B
cg27420123	0.4711864	-177.3668	-0.2428581	0.7140445	FSHB
cg10137010	0.1537592	-177.254	-0.2493611	0.4031202	TRPV3
cg01668126	0.4380164	-177.2337	-0.2490118	0.6870282	MSR1
cg23696712	0.5416806	-177.2336	-0.2263722	0.7680528	KRT6L
cg04744379	0.509477	-177.0384	-0.2342845	0.7437615	KLK15
cg26015133	0.5657801	-177.0116	-0.2197306	0.7855107	FCRLM2
cg16219122	0.2220757	-176.9846	-0.2600193	0.482095	ABCB1
cg23571857	0.2838598	-176.9396	-0.2628628	0.5467227	BIRC4BP
cg26718420	0.3749227	-176.8828	-0.2577172	0.6326399	C12orf59
cg13861644	0.4133269	-176.8541	-0.2527479	0.6660748	PIWIL1
cg12954718	0.2304437	-176.6959	-0.2605446	0.4909883	USP6
cg07676849	0.3240337	-176.6029	-0.2616235	0.5856572	FOLR3
cg21682902	0.5573161	-176.6003	-0.2218749	0.779191	HAL
cg12029639	0.1853153	-176.5101	-0.2549417	0.440257	MAB21L1
cg11584690	0.1212908	-176.3928	-0.2399178	0.3612086	ZNF574
cg05301852	0.3338457	-176.2472	-0.2608342	0.59468	FABP1
cg01910481	0.4853411	-176.2406	-0.2392843	0.7246255	PLUNC
cg02431964	0.3931876	-175.9629	-0.2549895	0.6481771	MARCO
cg11653864	0.2128587	-175.8571	-0.258224	0.4710826	ELK1
cg26928682	0.599416	-175.761	-0.2093052	0.8087211	TREML2
cg12133004	0.4039559	-175.5912	-0.2533339	0.6572897	C12orf54
cg27566805	0.230506	-175.5842	-0.2597324	0.4902384	USH2A
cg15055101	0.1267877	-175.434	-0.2408232	0.3676109	SH2D3A
cg08448751	0.4089026	-175.4304	-0.2525397	0.6614423	SEMA3G
cg27022827	0.2105317	-175.3082	-0.2575473	0.468079	TJP3
cg11472424	0.4142789	-175.2602	-0.2516552	0.6659341	PFKFB1
cg02148642	0.2276822	-175.2147	-0.2592191	0.4869013	RGPD5
cg12428416	0.5498995	-174.904	-0.2230526	0.772952	GJA3
cg19587887	0.449131	-174.8019	-0.2456491	0.6947802	PSKH2
cg27348440	0.5667077	-174.6699	-0.218354	0.7850618	
cg23029519	0.4534079	-174.5536	-0.244727	0.6981349	IFNA4
cg25043279	0.4382145	-174.4784	-0.2473819	0.6855964	C7orf33
cg15914863	0.579603	-174.3973	-0.2145621	0.7941651	CYP2W1

cg13605579	0.5143222	-174.1636	-0.2316424	0.7459645	ALPP
cg05500074	0.4649838	-173.9608	-0.2421989	0.7071827	TSKS
cg21355508	0.333866	-173.8726	-0.2592716	0.5931376	RBM10
cg17474651	0.4935828	-173.8436	-0.2362084	0.7297912	MAGEC3
cg14722162	0.3988535	-173.8328	-0.2529508	0.6518043	C5orf20
cg20789620	0.1895484	-173.8014	-0.2535463	0.4430947	FSD1
cg21491308	0.1385498	-173.7746	-0.2428128	0.3813626	TEX101
cg15798153	0.1746617	-173.6502	-0.2508786	0.4255403	PFTK1
cg13705284	0.2533025	-173.6214	-0.2597742	0.5130768	ACOX2
cg14625604	0.169727	-173.5719	-0.2498694	0.4195963	UBE2A
cg04514469	0.5718295	-173.1792	-0.2162055	0.788035	C14orf49
cg11052143	0.1598897	-173.1209	-0.2474778	0.4073675	ALS2CR11
cg19831369	0.3097072	-173.1167	-0.259852	0.5695592	HPS4
cg26980692	0.1976468	-172.8776	-0.2540477	0.4516945	SLC15A3
cg21964481	0.4075997	-172.8776	-0.25118	0.6587797	SLC34A3
cg07790638	0.408012	-172.7354	-0.2510368	0.6590487	LOC91431
cg11405695	0.2643769	-172.6174	-0.2594933	0.5238702	ATAD3C
cg12069309	0.06473751	-172.6174	-0.2141955	0.278933	SEMA3B
cg25605408	0.6201158	-172.5825	-0.2014577	0.8215735	FLJ40142
cg22445920	0.3944833	-172.4411	-0.2526537	0.647137	SLC36A3
cg06825142	0.03978399	-172.2629	-0.2000927	0.2398766	DRD4
cg05047411	0.2754039	-172.0942	-0.2593867	0.5347906	MAGEA8
cg03723845	0.5652186	-171.9025	-0.2174366	0.7826551	BTNL8
cg06736444	0.2827881	-171.8833	-0.2593223	0.5421104	SRRM2
cg26751195	0.5781908	-171.6088	-0.2136538	0.7918446	SPN
cg22165685	0.07430244	-171.5096	-0.2178714	0.2921739	VENTX
cg11201288	0.4703562	-171.4768	-0.2397445	0.7101007	SFRS11
cg07196761	0.582841	-171.1973	-0.2121249	0.7949659	WDR71
cg23887102	0.2767461	-171.1857	-0.2587608	0.5355069	TAAR6
cg17820828	0.4887591	-171.1445	-0.2357813	0.7245404	KCNQ1
cg19903229	0.2768302	-170.9178	-0.258573	0.5354032	C14orf105
cg14178895	0.200471	-170.7741	-0.2528321	0.4533031	C6orf105
cg19881895	0.5399979	-170.6494	-0.2235115	0.7635094	SLC43A3
cg08007665	0.5834132	-170.6005	-0.2116787	0.7950919	KCNQ1
cg04457051	0.348849	-170.5621	-0.2560638	0.6049128	SCOC
cg13446199	0.3121101	-170.5087	-0.257993	0.570103	PSCA
cg01762581	0.1787363	-170.3979	-0.2490881	0.4278245	DKK4
cg07864297	0.1671382	-170.3475	-0.2468163	0.4139546	ESRRB
cg17733331	0.07605612	-170.2297	-0.2176009	0.293657	CDH3
cg25661884	0.1583112	-170.2272	-0.2448232	0.4031344	RDH8
cg03960217	0.1387403	-170.2272	-0.2400006	0.3787409	LCE2C
cg24816455	0.2230873	-170.2078	-0.2550505	0.4781379	SEMA3B
cg19421044	0.1767576	-170.1655	-0.2485305	0.4252881	DNASE1L1
cg07473550	0.09584954	-170.122	-0.2258714	0.3217209	TSC22D3
cg13018903	0.412598	-170.0639	-0.2487461	0.6613441	FLJ25530

cg01474260	0.342871	-170.0346	-0.2561257	0.5989967	CESK1
cg24621042	0.2849946	-169.8795	-0.2578923	0.5428869	SERPINA1
cg09686308	0.2312016	-169.8795	-0.2555374	0.486739	CIB3
cg08321330	0.4678562	-169.6009	-0.2391589	0.7070151	TPSB2
cg18043195	0.5147299	-169.5377	-0.2290941	0.7438241	C9orf138
cg26644395	0.2091072	-169.445	-0.2529157	0.4620229	UCN3
cg01739167	0.3155938	-169.4367	-0.2571198	0.5727136	CHRNE
cg24862483	0.08084463	-169.2192	-0.2188854	0.2997301	CD300LG
cg14535518	0.2431425	-169.0738	-0.2558439	0.4989864	MAG
cg25082710	0.1945236	-168.8533	-0.2504703	0.4449939	IVL
cg12530021	0.1368251	-168.712	-0.2382433	0.3750684	SIGLEC12
cg14876043	0.587453	-168.6886	-0.2095941	0.7970471	NALP7
cg15206445	0.3720189	-168.5301	-0.2526954	0.6247143	SPG7
cg06980053	0.235104	-168.4447	-0.2547877	0.4898917	RASSF1
cg01714932	0.4055956	-168.3946	-0.2486808	0.6542764	PZP
cg20070090	0.2541893	-168.3889	-0.25598	0.5101693	S100A8
cg01982597	0.4390179	-168.2904	-0.2435891	0.682607	PGBD3
cg03087937	0.4438688	-168.2477	-0.242745	0.6866138	MUC15
cg20349625	0.5746139	-168.1859	-0.2130078	0.7876217	PLCG2
cg11959435	0.4028459	-168.0057	-0.2488014	0.6516473	IFNA1
cg21087701	0.3230407	-167.894	-0.2557722	0.5788128	GPR119
cg00968475	0.113787	-167.8902	-0.2305215	0.3443085	BHLHB9
cg04557383	0.09990897	-167.6202	-0.2253419	0.3252509	MT1H
cg06793062	0.541353	-167.6175	-0.2216008	0.7629538	CNTNAP4
cg12205591	0.336417	-167.2944	-0.254692	0.5911089	CRYAA
cg21109025	0.484821	-167.1731	-0.2343937	0.7192147	CCL2
cg05812599	0.4641512	-167.034	-0.2383963	0.7025475	CLPB
cg03956628	0.1574326	-167.0002	-0.2420311	0.3994637	MLH1
cg14076161	0.2659205	-166.9076	-0.2553881	0.5213086	PRB4
cg18674980	0.06738764	-166.643	-0.2104566	0.2778443	CA3
cg17034109	0.250932	-166.5113	-0.254421	0.505353	CYB561D1
cg20176989	0.3457672	-166.2876	-0.2534207	0.5991879	KIR3DL2
cg11128808	0.4956025	-166.1061	-0.2315262	0.7271286	CACNG6
cg05135288	0.2233478	-166.0227	-0.251896	0.4752438	RHOT2
cg00563932	0.4704962	-165.7676	-0.2364595	0.7069557	PTGDS
cg14186992	0.4123538	-165.6765	-0.2460742	0.6584281	HKR3
cg15075718	0.3804548	-165.6535	-0.2499679	0.6304227	MFRP
cg26757722	0.2152422	-165.642	-0.2507292	0.4659714	CACNG2
cg06800962	0.1876673	-165.1494	-0.2464921	0.4341594	TM4SF1
cg19539004	0.3130974	-165.0487	-0.2541615	0.5672589	LIF
cg24389359	0.3469499	-164.8503	-0.2523772	0.599327	CCDC63
cg10604646	0.2829477	-164.7336	-0.2542005	0.5371482	RGS5
cg15669228	0.3338743	-164.5576	-0.2529764	0.5868507	IFNA8
cg19258973	0.3640353	-164.4928	-0.250804	0.6148393	KRTHB3
cg26222045	0.3387242	-164.395	-0.2525929	0.5913171	UNQ5810

cg10318258	0.4874021	-164.1465	-0.232164	0.719566	RIPK3
cg10986043	0.2734883	-164.1398	-0.2535937	0.527082	TCAP
cg14662756	0.3977151	-164.1398	-0.2470227	0.6447378	NPFF
cg17749384	0.5901205	-164.121	-0.2066543	0.7967747	MPP7
cg19257200	0.5227445	-164.0954	-0.2243125	0.7470571	SOX10
cg03918304	0.1995784	-164.067	-0.2474797	0.4470581	HOXD10
cg05755354	0.3055223	-163.6578	-0.2533534	0.5588757	FRMD4A
cg16514843	0.3467244	-163.6481	-0.2515744	0.5982988	PAX4
cg15677294	0.3925256	-163.6262	-0.2473127	0.6398383	TUBB4
cg12391921	0.3642659	-163.5326	-0.2501394	0.6144053	ITGB1BP2
cg22954265	0.5752703	-163.3958	-0.2104909	0.7857612	FLJ45832
cg06323290	0.369143	-163.2662	-0.2495268	0.6186698	HK1
cg00418150	0.3644933	-163.1277	-0.2498499	0.6143432	ALX4
cg12610070	0.07939307	-163.1277	-0.2131105	0.2925036	TSPAN15
cg00833777	0.4235698	-163.0731	-0.2428501	0.6664199	ITGAM
cg04345908	0.4918993	-162.9841	-0.2305656	0.7224649	HLA-DQB2
cg05330360	0.2885023	-162.6511	-0.2527348	0.5412371	ZPBP2
cg20993403	0.5755242	-162.6356	-0.210046	0.7855701	EPB41L1
cg26705561	0.2897427	-162.6215	-0.2527157	0.5424584	SEC31L2
cg21514871	0.1343887	-162.5237	-0.2324864	0.3668751	MEFV
cg17412560	0.2365186	-162.4882	-0.250398	0.4869167	CSEN
cg12937434	0.1130428	-162.3762	-0.225687	0.3387298	BZRAP1
cg11825652	0.330113	-162.0661	-0.2514355	0.5815486	CAV2
cg26745032	0.2073389	-162.0204	-0.2469416	0.4542806	REPS2
cg11721194	0.3236445	-161.945	-0.2516246	0.5752692	SLAMF7
cg15792367	0.2699205	-161.812	-0.2517688	0.5216893	KLK11
cg18254848	0.4922141	-161.5843	-0.2297071	0.7219212	CLC
cg01143454	0.09420494	-161.5804	-0.2180513	0.3122562	C20orf141
cg00134539	0.4771644	-161.4818	-0.2326842	0.7098486	UBASH3A
cg19987219	0.4185446	-161.4396	-0.2425529	0.6610975	FLJ32011
cg19481686	0.3828966	-161.4345	-0.2469528	0.6298494	CDKN2B
cg13320683	0.4200022	-161.2009	-0.242195	0.6621972	RHOBTB1
cg02694395	0.2933137	-160.8614	-0.2514415	0.5447552	FMO4
cg12653105	0.2562944	-160.8447	-0.2504689	0.5067633	PLP2
cg23216015	0.1462874	-160.7387	-0.2342258	0.3805131	C7orf16
cg22772878	0.2146796	-160.7279	-0.24684	0.4615197	DIRAS1
cg25771201	0.3392887	-160.6178	-0.2499466	0.5892353	SCDR10
cg13125510	0.222744	-160.6178	-0.2476585	0.4704025	C11orf44
cg00044729	0.3752487	-160.5118	-0.2471111	0.6223598	FLJ25421
cg13286902	0.2110615	-160.4123	-0.2461446	0.4572061	CXorf34
cg18988110	0.2139248	-160.3132	-0.2464176	0.4603425	ATAD4
cg12732155	0.2149399	-160.0559	-0.2463406	0.4612805	LAPTM5
cg04387658	0.4463339	-160.0376	-0.2373991	0.6837329	CD86
cg24959428	0.3469687	-160.0038	-0.2490519	0.5960206	GBP6
cg23756219	0.1781865	-159.9318	-0.2406223	0.4188087	DRP2

cg26259865	0.5222568	-159.694	-0.2220325	0.7442893	LOC124220
cg22585988	0.4500228	-159.6196	-0.2365234	0.6865462	PVRL4
cg13370916	0.1877522	-159.5882	-0.2420529	0.4298052	STARD8
cg07947016	0.2339733	-159.5658	-0.2479194	0.4818926	KLK2
cg19910382	0.3468637	-159.5562	-0.2487451	0.5956088	FABP1
cg22010317	0.1996903	-159.4826	-0.243848	0.4435383	CXorf36
cg11061975	0.2964078	-159.4216	-0.2503718	0.5467796	SIRPB2
cg24101873	0.5719876	-159.3596	-0.2093578	0.7813454	C20orf70
cg02202484	0.3684264	-159.2696	-0.2468983	0.6153247	SPRR4
cg01185080	0.2997031	-159.161	-0.2501658	0.5498688	ZNF710
cg26916607	0.5551162	-159.1416	-0.2136946	0.7688109	CETP
cg10586756	0.4437551	-159.1181	-0.2372501	0.6810052	NUP93
cg00093177	0.5491419	-159.0571	-0.2151712	0.7643131	FLJ43826
cg23464269	0.2931219	-158.7968	-0.2499096	0.5430315	UGT1A3
cg14437986	0.3688639	-158.7565	-0.2465079	0.6153718	C6orf25
cg18719571	0.4406497	-158.7557	-0.237525	0.6781747	DTNBP1
cg11520395	0.3037192	-158.6633	-0.2497574	0.5534766	JMJD4
cg26229648	0.5927014	-158.6633	-0.2032524	0.7959538	CNTF
cg02721374	0.4536108	-158.2399	-0.2350616	0.6886724	OR1D2
cg19998328	0.3152356	-158.2113	-0.249221	0.5644566	LOC90580
cg21301440	0.1178738	-158.2	-0.2237603	0.3416341	CYGB
cg08390209	0.3374497	-158.0502	-0.2482362	0.5856858	CDKN2B
cg21307628	0.4280135	-158.0025	-0.2390004	0.6670139	URB
cg10919204	0.1802972	-157.9088	-0.2393569	0.4196541	CDH6
cg20579480	0.3285437	-157.8104	-0.2484841	0.5770279	APOBEC4
cg01248426	0.191251	-157.7741	-0.2411531	0.432404	ATP6V0D2
cg24110063	0.1685046	-157.7277	-0.2368787	0.4053833	COX6A2
cg13118849	0.5656291	-157.7023	-0.2102007	0.7758297	CSPG3
cg02876062	0.6013232	-157.5644	-0.2002339	0.8015572	FAM107B
cg01861509	0.4406856	-157.4112	-0.2366813	0.6773669	SPOCK2
cg04655481	0.4704945	-157.3528	-0.2314987	0.7019932	GPR21
cg00852964	0.2622812	-157.157	-0.2479387	0.5102199	VNN1
cg05786601	0.2662866	-156.8856	-0.2479097	0.5141963	AR
cg25142416	0.4640324	-156.7907	-0.232347	0.6963794	CA1
cg02149446	0.153374	-156.66	-0.2325839	0.3859579	C5AR1
cg21529807	0.1098102	-156.5929	-0.2196372	0.3294475	CEACAM4
cg14652095	0.2635574	-156.5452	-0.2475328	0.5110902	HIST1H1A
cg08816023	0.2878585	-156.5387	-0.2481984	0.5360569	FGF1
cg14117297	0.2771807	-156.5058	-0.2479782	0.5251589	MGC23244
cg22289837	0.1245713	-156.3666	-0.2243538	0.3489251	CA3
cg02184413	0.4313079	-156.2167	-0.2373777	0.6686856	VNN1
cg10414946	0.3880524	-156.1927	-0.242907	0.6309593	MS4A2
cg06852652	0.5372979	-156.0451	-0.2164688	0.7537667	CYP2C18
cg24484296	0.373176	-156.0449	-0.2442686	0.6174446	ZFPL
cg21789545	0.2309278	-156.0313	-0.2448502	0.475778	COL9A1

cg05696092	0.2392498	-155.9581	-0.2455296	0.4847794	NOSIP
cg19585882	0.5477854	-155.8742	-0.2138082	0.7615936	SSX1
cg18466173	0.5606778	-155.8508	-0.2105253	0.771203	C21orf93
cg22025233	0.3915119	-155.8463	-0.2422902	0.6338021	CYP2W1
cg19824441	0.3880091	-155.7487	-0.2426013	0.6306103	ADMR
cg25650811	0.4304392	-155.7413	-0.23719	0.6676292	LOC223075
cg02298612	0.582894	-155.5929	-0.2044606	0.7873546	CHST6
cg17031773	0.2836988	-155.5818	-0.2474036	0.5311024	KLK9
cg04558553	0.2417903	-155.4845	-0.2453544	0.4871447	UGT2B7
cg12554476	0.4526368	-155.4326	-0.2334814	0.6861182	GP2
cg09748960	0.2496582	-155.4064	-0.24586	0.4955182	BTNL2
cg15746187	0.4116555	-155.3761	-0.2395603	0.6512157	FBXO44
cg13088755	0.3303733	-155.2614	-0.2465622	0.5769354	P2RY10
cg14003512	0.2795732	-155.2083	-0.2470344	0.5266076	PLGLB2
cg17463527	0.5745652	-155.0355	-0.2064356	0.7810008	SGK2
cg23152755	0.3123273	-154.9441	-0.246884	0.5592114	DEFB105A
cg02657438	0.3584451	-154.7914	-0.2446018	0.603047	STON2
cg15830940	0.3192418	-154.776	-0.2465966	0.5658384	SMARCAL1
cg22960952	0.2441957	-154.7417	-0.2449492	0.4891449	FLJ23657
cg08831522	0.3651517	-154.6616	-0.2439904	0.6091421	ATP10A
cg21755709	0.2334197	-154.2336	-0.2436513	0.477071	C21orf124
cg20342105	0.254082	-154.1629	-0.2451699	0.4992518	BSCL2
cg11854877	0.2813659	-154.0562	-0.2462004	0.5275664	F8A1
cg12891678	0.1745599	-153.8348	-0.2348739	0.4094338	SPRR2D
cg24269657	0.5601491	-153.6248	-0.2094823	0.7696313	F7
cg04200192	0.5470331	-153.2411	-0.212572	0.7596051	HTR3D
cg17067005	0.5455707	-153.2106	-0.2129129	0.7584836	IL10
cg18059223	0.3742292	-153.1723	-0.2421978	0.616427	NALP2
cg05379350	0.1690051	-152.9878	-0.2330381	0.4020432	GIT1
cg08815403	0.3298166	-152.8707	-0.2448675	0.5746841	HSD17B13
cg00983899	0.3976081	-152.7928	-0.2395652	0.6371733	CABP2
cg02688643	0.491717	-152.7793	-0.2246635	0.7163805	MGST2
cg12639234	0.3127617	-152.5878	-0.2451508	0.5579125	NAT2
cg06417962	0.3780621	-152.4418	-0.2413458	0.619408	RNH1
cg09548084	0.3335975	-152.3799	-0.2443539	0.5779514	SLC35B3
cg01138020	0.3135263	-152.3304	-0.2449456	0.5584719	MGC29671
cg00406188	0.3811324	-152.3195	-0.2409683	0.6221007	LCE2C
cg10173723	0.5901434	-152.2726	-0.200776	0.7909194	KRT25C
cg18675600	0.3258607	-152.2129	-0.244528	0.5703887	PTP4A3
cg16547341	0.2238897	-152.0791	-0.2409723	0.4648619	USP29
cg03900284	0.5003391	-151.9483	-0.2224136	0.7227527	P518
cg12397274	0.2288363	-151.9259	-0.2413679	0.4702042	TINAG
cg12781568	0.3123246	-151.9005	-0.2446424	0.556967	WT1
cg25462291	0.1408266	-151.7955	-0.2252031	0.3660297	HEYL
cg14132995	0.09991955	-151.7598	-0.2117944	0.311714	SLC35A2

cg10710439	0.2062771	-151.6464	-0.2384197	0.4446968	FLJ37549
cg12493906	0.3147737	-151.5382	-0.2443259	0.5590996	MMP26
cg21132577	0.1752801	-151.5128	-0.2330758	0.4083559	MYOM2
cg15703357	0.3543327	-151.4916	-0.2425636	0.5968962	LINCR
cg12019109	0.346241	-151.4402	-0.2430269	0.5892678	AZGP1
cg02046017	0.2290008	-151.3488	-0.2409112	0.469912	LOC220070
cg13396068	0.4200818	-151.2254	-0.235721	0.6558028	DCD
cg24870391	0.2535776	-151.1246	-0.2427543	0.4963319	CCL11
cg21652958	0.09934401	-151.0413	-0.2109358	0.3102798	THBS2
cg20436912	0.3185941	-150.8766	-0.2437438	0.5623379	SEC14L4
cg22496683	0.3516303	-150.8161	-0.2422517	0.593882	MGC27016
cg01507173	0.395797	-150.8147	-0.2384124	0.6342095	IL1F5
cg04872051	0.3018103	-150.8038	-0.2439152	0.5457255	CHST7
cg07426848	0.488302	-150.6892	-0.2240735	0.7123755	S100A3
cg11432797	0.4663437	-150.6655	-0.2281464	0.6944901	SPN
cg17078393	0.5870589	-150.6126	-0.2007642	0.7878231	LCK
cg06255227	0.5738192	-150.532	-0.2042795	0.7780986	NPBWR2
cg22396129	0.2422813	-150.5201	-0.2414466	0.4837278	ZCRB1
cg09551916	0.2859957	-150.419	-0.2435002	0.529496	CFHR2
cg03221914	0.1550327	-150.3719	-0.2276335	0.3826661	HIST1H2AJ
cg06619299	0.572544	-150.2821	-0.2044802	0.7770241	GMCL1L
cg01356829	0.4691005	-150.2611	-0.2274001	0.6965006	IL12RB2
cg01227519	0.5798523	-150.2129	-0.2025039	0.7823561	SPANXD
cg01192952	0.26468	-150.1873	-0.2426155	0.5072955	LGALS14
cg07846167	0.1150428	-150.1779	-0.2158636	0.3309064	FBLIM1
cg07193504	0.3765666	-150.1313	-0.2398472	0.6164138	SRP9
cg09923855	0.2951431	-150.0941	-0.2433517	0.5384948	HTATSF1
cg25410053	0.3210398	-150.0709	-0.2430636	0.5641034	ZIC3
cg09120035	0.3541225	-150.0597	-0.2415299	0.5956524	CYP11B1
cg25545210	0.4578915	-149.9428	-0.2291397	0.6870312	KRTHA4
cg19241311	0.3514672	-149.5591	-0.2413432	0.5928104	DEFB123
cg19067730	0.09023859	-149.3628	-0.2058074	0.296046	PPGB
cg14841098	0.1361317	-149.3206	-0.2217272	0.357859	SLC9A7
cg20856834	0.395326	-149.2694	-0.2373936	0.6327196	OR12D3
cg26895595	0.2630136	-149.0614	-0.2416467	0.5046603	MAGEB3
cg06210526	0.5002136	-148.9653	-0.2206494	0.7208629	CYP2F1
cg05564251	0.538348	-148.8062	-0.2121804	0.7505285	SP140
cg10099900	0.4776057	-148.797	-0.2249455	0.7025512	PSMF1
cg20857253	0.199378	-148.7162	-0.2349595	0.4343375	TCP10L
cg22182945	0.4113809	-148.7003	-0.2351435	0.6465244	NYD-SP26
cg13633026	0.3162775	-148.6491	-0.2421137	0.5583913	MS4A8B
cg13745346	0.4462832	-148.6446	-0.2302006	0.6764838	CBFA2T3
cg21885995	0.3815599	-148.4947	-0.2382455	0.6198054	SRP68
cg14533138	0.4927489	-148.2397	-0.2217116	0.7144605	KRTHA3B
cg25890048	0.4317595	-148.1793	-0.2320765	0.6638361	OR5I1

cg22986999	0.1400547	-148.0791	-0.2217606	0.3618154	MRGPRF
cg26777475	0.3579997	-148.0717	-0.2398469	0.5978466	PCOLCE
cg11811840	0.3850173	-147.8648	-0.2374769	0.6224942	UGT1A1
cg19229991	0.4800247	-147.7727	-0.2238618	0.7038866	FLJ22655
cg12268344	0.5530353	-147.7569	-0.2080628	0.7610981	PRM1
cg01359534	0.5439578	-147.6295	-0.2101874	0.7541453	AQP10
cg08711674	0.2613946	-147.6231	-0.2404167	0.5018114	AKT1S1
cg22580353	0.1478067	-147.5805	-0.2234087	0.3712154	PVR
cg07434382	0.3860631	-147.3886	-0.2370416	0.6231046	LOC348174
cg10517312	0.253154	-147.2693	-0.239637	0.492791	RNF36
cg07378350	0.216902	-147.0818	-0.2360395	0.4529415	GDF5
cg20176648	0.2351333	-147.0034	-0.2379679	0.4731012	AQP1
cg08159444	0.134385	-146.8138	-0.2190439	0.353429	PNMA5
cg14726637	0.1006268	-146.7947	-0.2076808	0.3083076	CR1
cg11328541	0.1837315	-146.7612	-0.2306578	0.4143894	OR7C1
cg07581973	0.2048223	-146.7089	-0.2340926	0.4389149	PRDX4
cg17040807	0.1529974	-146.6282	-0.2239034	0.3769008	CYGB
cg08088390	0.1795399	-146.3545	-0.2295212	0.409061	DEFB125
cg25107791	0.3383541	-146.2435	-0.239609	0.5779631	CLPS
cg08965337	0.1472754	-146.1714	-0.2220494	0.3693248	RAP2C
cg07197059	0.1654985	-146.1145	-0.2264024	0.3919009	EFS
cg27244482	0.2731416	-145.9511	-0.2396367	0.5127783	CAMK2A
cg26232558	0.4663299	-145.9511	-0.2251709	0.6915008	OR3A1
cg02388150	0.1279383	-145.469	-0.2159293	0.3438676	SFRP1
cg05788638	0.4773603	-145.1095	-0.2226937	0.7000539	SERPINA10
cg06812844	0.2995237	-145.0517	-0.2395009	0.5390247	TRPM2
cg06026755	0.5625256	-145.0284	-0.2042062	0.7667317	CEP27
cg03941108	0.3538001	-144.9751	-0.237866	0.5916662	C1QB
cg11233153	0.1728939	-144.9419	-0.2269838	0.3998777	SLC10A3
cg11267879	0.3235747	-144.7515	-0.2390064	0.5625811	CYP2F1
cg03872376	0.4027525	-144.5061	-0.2332861	0.6360386	ZP4
cg17461214	0.5364702	-144.4934	-0.2101498	0.74662	CHML
cg11710560	0.5564171	-144.4346	-0.2053825	0.7617996	KRTAP9-3
cg15383120	0.1313437	-144.333	-0.2159635	0.3473071	DUSP22
cg25949363	0.1910587	-144.2989	-0.2298709	0.4209296	TMEM92
cg19554294	0.3775205	-144.2245	-0.2355769	0.6130974	VN1R2
cg27345534	0.3158865	-144.1038	-0.238664	0.5545505	PRB2
cg03870261	0.1396333	-144.0941	-0.2181566	0.3577899	TIMM13
cg23540651	0.3486396	-144.0392	-0.2374625	0.5861021	GBP3
cg25866075	0.2677559	-143.9838	-0.2378218	0.5055777	NALP12
cg06277657	0.2433434	-143.9838	-0.2362205	0.4795639	DGKI
cg01309152	0.3242406	-143.9451	-0.2383627	0.5626033	PCP4
cg27168844	0.4012647	-143.8968	-0.233013	0.6342776	IL17
cg15417244	0.4970275	-143.8216	-0.2181543	0.7151818	PDZRN4
cg06545504	0.4347506	-143.7397	-0.2286847	0.6634353	HTN1

cg00174500	0.2480535	-143.7363	-0.2363905	0.4844441	CMTM5
cg04180953	0.170699	-143.6626	-0.2254007	0.3960997	DSC1
cg16381688	0.3108299	-143.574	-0.2383075	0.5491374	THEM2
cg07897701	0.5251635	-143.516	-0.2121056	0.7372691	ABP1
cg24147596	0.4236301	-143.509	-0.2300442	0.6536743	ARL14
cg18374517	0.4764771	-143.3914	-0.2217467	0.6982238	ALPPL2
cg21550442	0.2531966	-143.2005	-0.2363298	0.4895264	FLJ36445
cg16269097	0.1567384	-143.1929	-0.2218208	0.3785592	CASK
cg00308665	0.2288575	-143.1111	-0.2341073	0.4629648	HTR2A
cg24304714	0.2063323	-143.1111	-0.2312677	0.4376	LCE1C
cg14345281	0.1509641	-143.0439	-0.2202531	0.3712171	NHS
cg24546463	0.2286995	-142.876	-0.2338947	0.4625942	MGC39715
cg00953256	0.4207458	-142.8701	-0.2299805	0.6507263	CCND1
cg22082462	0.5472254	-142.7444	-0.2066253	0.7538508	CPNE6
cg05730092	0.536335	-142.6583	-0.2091018	0.7454368	KIAA1446
cg13461622	0.4177557	-142.6077	-0.2301781	0.6479338	RUNX3
cg00371195	0.5703713	-142.2528	-0.2006831	0.7710544	F2
cg02436686	0.2318091	-142.1012	-0.2335803	0.4653894	GMFG
cg24054653	0.1568248	-142.0983	-0.2208854	0.3777102	C1GALT1C1
cg24779040	0.2859702	-142.0776	-0.2369465	0.5229167	TSPYL2
cg12067287	0.5512779	-142.0683	-0.2052759	0.7565538	MYOM1
cg26158194	0.4923005	-142.0671	-0.217978	0.7102786	LSP1
cg02656594	0.1786712	-142.0636	-0.2256403	0.4043115	IL21R
cg18123948	0.1030478	-141.9098	-0.2042271	0.3072748	GATA4
cg24387818	0.2457106	-141.7364	-0.2345493	0.48026	POF1B
cg16051685	0.5240119	-141.6119	-0.2112126	0.7352245	TRIM63
cg18133957	0.1387909	-141.4952	-0.2155962	0.3543871	APC2
cg23207527	0.5157523	-141.4287	-0.2128648	0.7286171	RBM24
cg14457691	0.1511281	-141.0403	-0.2185173	0.3696454	ARMCX4
cg08798116	0.1639538	-140.7594	-0.2213716	0.3853254	GPC4
cg01975392	0.1622689	-140.7142	-0.2209441	0.383213	LANCL3
cg03014957	0.3311475	-140.4576	-0.2354874	0.5666349	DEFB118
cg25450806	0.5510874	-140.4444	-0.2043828	0.7554702	PRDM2
cg12032049	0.4084527	-140.3757	-0.2297236	0.6381763	LPO
cg06256735	0.2155986	-140.3082	-0.2301532	0.4457518	MFAP5
cg16150435	0.4979269	-140.2662	-0.2157556	0.7136825	C6orf15
cg11806565	0.1634731	-139.864	-0.2204735	0.3839466	PHKA1
cg02148711	0.1946161	-139.7905	-0.2265769	0.421193	ATRX
cg08575950	0.4632433	-139.7288	-0.2216373	0.6848806	FLJ14503
cg25994725	0.3269959	-139.7201	-0.2350306	0.5620265	C6orf81
cg10478221	0.2805313	-139.6441	-0.2348198	0.5153511	WFIKKN1
cg13322449	0.2199444	-139.5563	-0.2300656	0.45001	CEACAM3
cg25933726	0.2388191	-139.4866	-0.2320615	0.4708806	RPGR
cg23282949	0.4291357	-139.4665	-0.22653	0.6556656	RENBP
cg16148454	0.5382012	-139.0245	-0.2065277	0.7447289	TMED6

cg27583102	0.4610571	-138.7727	-0.2213565	0.6824136	KLK5
cg02017155	0.2559213	-138.7529	-0.2328345	0.4887558	FFAR3
cg08403419	0.4199518	-138.6893	-0.2271661	0.647118	RLN3R2
cg15977272	0.2823179	-138.4606	-0.2339163	0.5162343	EFNB1
cg26128441	0.336075	-138.4265	-0.2337498	0.5698249	P2RX3
cg18799866	0.2224857	-138.4265	-0.2294109	0.4518966	SLC9A7
cg16609872	0.2419634	-138.358	-0.231399	0.4733625	KCNQ1
cg26457013	0.4015389	-138.3243	-0.2290142	0.6305531	TMEM86B
cg12593411	0.3998239	-138.2721	-0.2291552	0.6289791	ANGPTL6
cg20922422	0.1560498	-137.8902	-0.2169528	0.3730026	PCYT1B
cg23704362	0.3141529	-137.8764	-0.2338011	0.547954	C8orf46
cg19521927	0.4239737	-137.8672	-0.2260818	0.6500555	PLA2G5
cg04184278	0.4110281	-137.6795	-0.2275051	0.6385332	GYS2
cg13473336	0.4570079	-137.6662	-0.2212512	0.6782591	SLC5A2
cg23324787	0.3874058	-137.5253	-0.2298102	0.617216	RAG2
cg00436282	0.5379537	-137.3753	-0.2055952	0.7435489	STATH
cg04057858	0.3742607	-137.3616	-0.2307849	0.6050456	UNQ9391
cg19382175	0.5173048	-137.3616	-0.2100416	0.7273464	PDE6A
cg07478122	0.3898567	-137.2379	-0.2293746	0.6192313	HBII-13
cg10051054	0.5367434	-136.9331	-0.2056021	0.7423455	CCDC27
cg24926780	0.2355314	-136.8605	-0.2295133	0.4650446	OR10H2
cg07374632	0.2234748	-136.8316	-0.2281569	0.4516317	PCSK1N
cg13131015	0.2821915	-136.7722	-0.2325222	0.5147137	GUCY2C
cg04761824	0.4791474	-136.6164	-0.216879	0.6960264	30-nov
cg14620221	0.5417415	-136.4685	-0.2042019	0.7459434	OR8B8
cg10787197	0.3396055	-136.4306	-0.2320672	0.5716727	C6orf105
cg27554782	0.1146156	-136.4306	-0.2034435	0.3180591	CHRNB4
cg20169062	0.2124573	-136.4239	-0.2263744	0.4388317	HSPB3
cg01080862	0.2081636	-136.3773	-0.2257219	0.4338855	IL13RA1
cg16175792	0.3133605	-136.2589	-0.2325105	0.5458711	HSD3B1
cg00071250	0.3765863	-136.2344	-0.2297604	0.6063467	FASLG
cg07974891	0.29629	-136.0594	-0.2322772	0.5285671	ITGB1BP1
cg26379475	0.2263527	-136.0255	-0.2277956	0.4541482	SH2D1B
cg11656547	0.1114426	-135.9732	-0.2019127	0.3133553	MAMDC2
cg22780475	0.3497267	-135.7655	-0.231109	0.5808358	CBLC
cg16343842	0.156277	-135.6637	-0.2149995	0.3712764	CD99L2
cg06469542	0.3976184	-135.6561	-0.2274652	0.6250837	THSD3
cg05740244	0.2508841	-135.5476	-0.2297587	0.4806428	LDHC
cg18920846	0.5029545	-135.5434	-0.2117744	0.714729	ASCL3
cg03573747	0.4630949	-135.5071	-0.2188224	0.6819173	ADIPOQ
cg13311440	0.2832767	-135.5057	-0.2315021	0.5147789	CD48
cg24623694	0.229826	-135.4901	-0.2277163	0.4575422	PRX
cg18312429	0.3192517	-135.487	-0.2318246	0.5510763	SLC10A2
cg22833175	0.2684715	-135.4635	-0.2308292	0.4993008	WBP11
cg12687463	0.2895101	-135.4299	-0.2316125	0.5211226	CFHR1

cg08130265	0.32945	-135.3191	-0.2314907	0.5609407	C15orf5
cg18942631	0.2290509	-135.3018	-0.2274571	0.456508	CASQ2
cg01346718	0.1787889	-135.1946	-0.219577	0.3983659	CSNK1E
cg12629515	0.2503232	-135.1894	-0.2293876	0.4797108	HIST1H2BO
cg21474838	0.4514299	-134.8477	-0.220169	0.6715989	KLRD1
cg14176836	0.2308126	-134.7145	-0.2271467	0.4579593	ITGAL
cg26738010	0.5336511	-134.6456	-0.2048735	0.7385246	CETN1
cg06817269	0.4518326	-134.6427	-0.2199657	0.6717983	GCNT3
cg22879289	0.3036336	-134.6172	-0.2311622	0.5347958	NID1
cg19464252	0.2765341	-134.5136	-0.2304112	0.5069454	FBS1
cg15552238	0.2533045	-134.4429	-0.2289838	0.4822883	EMR3
cg16855440	0.3767777	-134.4402	-0.2283728	0.6051506	KLC3
cg26661623	0.4085289	-134.3867	-0.2253808	0.6339096	ASGR2
cg24642820	0.504107	-134.2769	-0.210721	0.714828	NUP210
cg13588835	0.4650913	-134.2737	-0.2176464	0.6827377	LGALS7
cg11380128	0.5278329	-134.0006	-0.2057216	0.7335544	PRLH
cg19937039	0.1987301	-133.8854	-0.2220366	0.4207667	SERPINA10
cg01375871	0.2651354	-133.8268	-0.2292486	0.4943841	TPSD1
cg02332537	0.4675421	-133.7292	-0.2168812	0.6844233	RTP3
cg02633817	0.4687548	-133.7133	-0.2166715	0.6854264	FXYD3
cg26233253	0.4016889	-133.685	-0.2255813	0.6272702	SLC35E4
cg04317399	0.1376502	-133.3707	-0.2079014	0.3455516	HOXA4
cg16003238	0.1754233	-133.3035	-0.2171787	0.392602	PUNC
cg16504670	0.4933669	-133.1172	-0.2119913	0.7053581	FLJ20186
cg19963797	0.1194283	-133.1128	-0.201997	0.3214253	GLT28D1
cg07258507	0.3063383	-133.0748	-0.2299021	0.5362405	IL17E
cg08942800	0.1774896	-133.0248	-0.2173513	0.3948409	CRISP2
cg06885782	0.2599989	-132.9938	-0.228209	0.4882079	KCNQ4
cg27337148	0.213447	-132.5818	-0.2231075	0.4365545	CAMK1G
cg22332306	0.3398844	-132.5634	-0.2289784	0.5688628	C3orf63
cg09971646	0.1898645	-132.5089	-0.2192709	0.4091354	DLK1
cg07876586	0.1853281	-132.26	-0.2182121	0.4035402	PGRMC1
cg25410279	0.1288418	-132.2316	-0.20422	0.3330618	DXS9879E
cg21533271	0.1477914	-132.2175	-0.2096597	0.3574512	PLXNA3
cg23855121	0.2535597	-132.0945	-0.226982	0.4805417	TLR10
cg25427638	0.3869058	-132.0485	-0.2257326	0.6126384	CYP2A7
cg17169998	0.1265117	-131.9835	-0.2032551	0.3297668	MLC1
cg02096520	0.5053274	-131.9178	-0.2089517	0.7142791	FGF16
cg26135325	0.4745199	-131.7008	-0.214345	0.6888649	LCE3A
cg16567044	0.336175	-131.6666	-0.228376	0.564551	MEG3
cg20322977	0.2775392	-131.3523	-0.2277825	0.5053217	CYP26C1
cg03641225	0.2118124	-131.1253	-0.2215844	0.4333968	DIRAS3
cg15480475	0.515356	-131.0477	-0.2064244	0.7217804	TUB
cg13482233	0.4990617	-131.0376	-0.2095675	0.7086292	HEPH
cg17839611	0.5039102	-130.8395	-0.208524	0.7124342	GNGT2

cg12200412	0.1642312	-130.7021	-0.212361	0.3765922	CD1E
cg23661676	0.3143349	-130.6752	-0.2279432	0.5422781	GPR42
cg11511443	0.2400878	-130.6749	-0.2245747	0.4646626	LMO3
cg11812202	0.4370045	-130.4453	-0.2190843	0.6560888	PNLIP
cg02964385	0.2720962	-130.2993	-0.2266302	0.4987263	STK38
cg26414720	0.5045059	-130.2496	-0.2080227	0.7125286	MAGEA1
cg26091981	0.5047624	-130.2383	-0.2079652	0.7127276	LCT
cg26185508	0.4653139	-130.1152	-0.2147517	0.6800656	CDCP2
cg24459209	0.4149812	-130.0366	-0.2214689	0.6364501	PRG3
cg24010336	0.2068648	-130.0339	-0.2198771	0.4267419	FBXO17
cg04344997	0.2665379	-130.0226	-0.2260804	0.4926183	EMD
cg24252809	0.3726349	-129.9543	-0.225221	0.5978559	MRGPRX1
cg26561254	0.1664289	-129.9173	-0.2121393	0.3785682	ISG20L1
cg05376954	0.248924	-129.9118	-0.2247094	0.4736334	FPR1
cg21045917	0.3965858	-129.4864	-0.2229322	0.619518	ALAS2
cg05206587	0.2966186	-129.4157	-0.2267514	0.5233699	GLRA2
cg08356693	0.3123693	-129.3429	-0.2268449	0.5392143	ITLN1
cg12351042	0.4287351	-129.2955	-0.2193071	0.6480422	OR2B2
cg07428182	0.209702	-129.2818	-0.2196244	0.4293264	PLXNA3
cg20664247	0.2426293	-129.1006	-0.2234397	0.466069	PEX10
cg01214847	0.4192866	-128.8996	-0.2201433	0.6394299	TMPRSS3
cg22341310	0.2100207	-128.8771	-0.2193128	0.4293335	ZNF541
cg05102817	0.4603514	-128.7823	-0.2145863	0.6749377	RBM18
cg17571782	0.1894136	-128.7755	-0.2158132	0.4052268	CXorf40A
cg27655855	0.4946328	-128.6358	-0.2087772	0.70341	CST9L
cg12237269	0.503112	-128.56	-0.2071634	0.7102754	SLN
cg21808053	0.2996776	-128.3954	-0.2259409	0.5256186	DIRAS3
cg27413508	0.2266098	-128.2539	-0.2209552	0.447565	COX4I2
cg07373172	0.4173869	-128.197	-0.219822	0.6372089	IFNA21
cg16512727	0.1761041	-128.1452	-0.2126106	0.3887147	C12orf4
cg18123677	0.2146274	-128.0961	-0.2192509	0.4338783	SERPINI1
cg04828792	0.4334655	-127.8595	-0.2176521	0.6511175	MX2
cg06277838	0.1775126	-127.8142	-0.2125966	0.3901092	SLC16A2
cg02947354	0.3997765	-127.7092	-0.2212618	0.6210383	LOC129530
cg18971671	0.4843258	-127.5509	-0.2098433	0.6941691	TULP2
cg10795646	0.1780808	-127.4704	-0.2123952	0.390476	S100A10
cg06154597	0.374494	-127.3565	-0.2230467	0.5975407	MGC4618
cg25298754	0.271777	-127.2698	-0.2239827	0.4957597	ZBED2
cg21932814	0.2223656	-127.2632	-0.2195311	0.4418967	CSTA
cg22909609	0.2777573	-127.2459	-0.2242591	0.5020164	ITGBL1
cg20017147	0.2858282	-127.1249	-0.2244836	0.5103118	TEX101
cg15992730	0.3115287	-127.0617	-0.2249171	0.5364458	GDF3
cg26240939	0.4589888	-126.8753	-0.2134159	0.6724048	LOC57149
cg02982734	0.5182874	-126.8415	-0.2030843	0.7213718	MAGEL2
cg25670900	0.3457307	-126.7828	-0.2240958	0.5698264	ZCCHC5

cg24660086	0.4551592	-126.7734	-0.2139035	0.6690627	RGR
cg00958560	0.4325894	-126.667	-0.2168723	0.6494617	FLJ46481
cg02504280	0.4773206	-126.3064	-0.2101503	0.6874709	MAGEA12
cg00673191	0.2277265	-126.2915	-0.2193147	0.4470413	DOPEY2
cg26143719	0.1786359	-126.2	-0.2113262	0.3899621	C1QTNF6
cg14654731	0.4364353	-126.1371	-0.2159973	0.6524326	FLJ30834
cg10401803	0.1978368	-125.9592	-0.2146983	0.4125351	TAF1
cg23075286	0.4058289	-125.9435	-0.2193204	0.6251493	GALP
cg21991396	0.5214104	-125.9272	-0.2018608	0.7232713	CIAS1
cg02611282	0.2312441	-125.396	-0.2189279	0.450172	CREB5
cg19526600	0.4394251	-125.1987	-0.2149294	0.6543545	DIO1
cg21578906	0.5216716	-125.1222	-0.2012871	0.7229587	SLC5A4
cg13217373	0.2016527	-125.0585	-0.2145151	0.4161678	RPS6KA2
cg16268563	0.4983018	-125.0326	-0.2056611	0.703963	ATP6V1G2
cg11762346	0.4969354	-125.0036	-0.2058847	0.7028201	HKDC1
cg05559445	0.2580214	-124.9377	-0.2210443	0.4790657	CDKN1C
cg03404502	0.453627	-124.8709	-0.2127555	0.6663825	GPR18
cg22975712	0.4591902	-124.7923	-0.2118937	0.6710839	GALR3
cg03742272	0.5254032	-124.6093	-0.2001985	0.7256017	ALOX12B
cg11787522	0.3148147	-124.2692	-0.2225713	0.5373861	STRA6
cg20050113	0.1920189	-124.2387	-0.2120957	0.4041146	SLC9A2
cg03879730	0.3545677	-124.1855	-0.2216243	0.576192	PSMD4
cg05190718	0.4899089	-124.0583	-0.2064681	0.696377	CASQ2
cg09998591	0.4617698	-124.0461	-0.2109731	0.672743	KCNQ1DN
cg08496601	0.201571	-123.9886	-0.2135064	0.4150774	EBP
cg09801065	0.5209592	-123.9182	-0.2006248	0.721584	FAT2
cg09584711	0.235677	-123.8523	-0.2180156	0.4536925	HPR
cg13760253	0.4335091	-123.7761	-0.2146067	0.6481158	DNAJC5B
cg00134787	0.3704179	-123.6572	-0.2203526	0.5907705	MYH1
cg06872381	0.5168689	-123.57	-0.2011901	0.718059	DPPA3
cg20189937	0.2726843	-123.5296	-0.2207426	0.4934269	L2HGDH
cg05624196	0.3642512	-123.4439	-0.2205318	0.584783	APOD
cg21631409	0.27547	-123.4117	-0.220788	0.496258	ALDH3B2
cg03803009	0.3702327	-123.3714	-0.2201274	0.5903602	GPR142
cg21066636	0.2546939	-123.3714	-0.219374	0.474068	TM4SF5
cg12654845	0.4283964	-123.283	-0.2148421	0.6432385	CLDN2
cg09076077	0.3754302	-123.2799	-0.2197216	0.5951518	FLJ33860
cg26709720	0.3257397	-123.2748	-0.2216512	0.5473909	B3GALT5
cg17542385	0.3196481	-123.2471	-0.2216721	0.5413202	LCE4A
cg19062189	0.2707928	-123.2296	-0.2203582	0.491151	TMSL8
cg26984805	0.3186894	-123.1554	-0.2215963	0.5402857	TACR2
cg17974185	0.339642	-123.1525	-0.2212804	0.5609224	CTNNBL1
cg16907488	0.4441139	-123.1484	-0.2127719	0.6568857	CCDC17
cg10124201	0.2348276	-122.9905	-0.2171168	0.4519444	BOLL
cg03793778	0.4088506	-122.9051	-0.2166571	0.6255078	LGALS7

cg22983092	0.3763705	-122.8022	-0.219263	0.5956336	KRT25A
cg11059341	0.3968341	-122.7089	-0.2176201	0.6144542	MYL1
cg10885338	0.3934495	-122.6466	-0.217859	0.6113085	ECRG4
cg08946332	0.4483714	-122.5591	-0.211759	0.6601303	ALOX12
cg14528319	0.2120551	-122.4428	-0.2136758	0.4257309	GIPC1
cg07326586	0.1497158	-122.3193	-0.2008745	0.3505903	UBD
cg27349244	0.4389757	-122.2964	-0.2127879	0.6517636	MLXIP
cg16536450	0.2022378	-122.2964	-0.212011	0.4142489	UNQ2541
cg20816612	0.2064867	-122.2308	-0.2126298	0.4191165	PHKA1
cg02284188	0.4791444	-122.2258	-0.2069422	0.6860866	TRIM49
cg26922202	0.3751629	-122.2138	-0.2188563	0.5940192	OR2S2
cg04780454	0.3350646	-122.0504	-0.2204441	0.5555087	SERPINF2
cg12188416	0.2801453	-121.9725	-0.219711	0.4998563	TP73L
cg27562023	0.4232537	-121.9725	-0.2144068	0.6376605	RPH3AL
cg24433189	0.1700291	-121.9319	-0.2054436	0.3754727	SSTR5
cg02966851	0.2686114	-121.8192	-0.2189468	0.4875581	C6orf149
cg07950803	0.2873658	-121.798	-0.2198597	0.5072255	CD1A
cg22325572	0.211688	-121.7855	-0.2129982	0.4246861	CD53
cg01722450	0.4862616	-121.6519	-0.2053597	0.6916213	C18orf20
cg23349242	0.3042371	-121.531	-0.220075	0.524312	SUSD2
cg03014680	0.4238408	-121.4482	-0.2139345	0.6377753	CLEC12A
cg06836849	0.178789	-121.4178	-0.2068341	0.3856231	SLC17A8
cg21860846	0.2692033	-121.376	-0.21858	0.4877833	ELK1
cg09784259	0.3099914	-121.3	-0.2199438	0.5299352	SELE
cg17896229	0.492314	-120.8481	-0.203766	0.6960801	PROKR2
cg16713808	0.4411968	-120.8059	-0.2113738	0.6525706	CRNN
cg03599338	0.3775191	-120.6911	-0.2174613	0.5949804	SUSD2
cg27257987	0.4350423	-120.5974	-0.2119787	0.6470209	PSG4
cg04721098	0.378996	-120.5503	-0.2172459	0.5962419	CACNG3
cg24655310	0.3935857	-120.5024	-0.2161196	0.6097053	CYP4F11
cg18085435	0.3135966	-120.4985	-0.2192721	0.5328687	ATP8B1
cg18192417	0.1766402	-120.396	-0.2054125	0.3820527	NEBL
cg19642007	0.4634043	-120.1337	-0.2078333	0.6712376	TNNT3
cg04726200	0.3370087	-120.089	-0.2187323	0.555741	SLC22A18
cg17915429	0.4478273	-120.0752	-0.2099565	0.6577839	PGLYRP2
cg00466249	0.3795245	-120.073	-0.2168166	0.5963411	MGC15523
cg06363801	0.1733946	-120.0699	-0.2044042	0.3777988	MOSPD1
cg10464775	0.4636993	-119.9311	-0.2076368	0.6713361	LAMP1
cg15014034	0.1870425	-119.8836	-0.2070179	0.3940603	MAOA
cg14001035	0.4003913	-119.8249	-0.2149965	0.6153878	ATP10A
cg07842062	0.2867754	-119.6322	-0.2179001	0.5046755	MEFV
cg13030582	0.1689577	-119.3674	-0.2027451	0.3717028	MFAP4
cg00625425	0.1842059	-119.1416	-0.205759	0.3899649	ANKRD38
cg22584138	0.2582695	-119.0432	-0.2156928	0.4739623	SLC6A4
cg00191052	0.2353936	-119.0345	-0.213507	0.4489007	CHST7

cg00474004	0.3591543	-119.0051	-0.2170713	0.5762256	IFNA14
cg07297906	0.2352689	-118.8603	-0.2133276	0.4485966	GPC3
cg21745164	0.3174188	-118.8052	-0.2178017	0.5352205	LOC63928
cg05767404	0.2678013	-118.7918	-0.2161402	0.4839415	C1orf150
cg13614083	0.457292	-118.7853	-0.2076874	0.6649794	KCNAB2
cg19206010	0.2105585	-118.6713	-0.2098905	0.420449	UXT
cg17753124	0.4878209	-118.6324	-0.2029223	0.6907432	IER2
cg20790540	0.4485011	-118.6133	-0.2087482	0.6572493	PTCRA
cg17231524	0.2473641	-118.4129	-0.2141616	0.4615257	MGC39606
cg01757745	0.1624424	-118.2313	-0.2001302	0.3625726	C10orf93
cg24693053	0.255695	-118.207	-0.2147079	0.4704029	MFSD7
cg12876594	0.2095671	-117.6645	-0.2087885	0.4183556	NPR2
cg04881903	0.4403141	-117.6578	-0.2090614	0.6493754	CAPG
cg12687215	0.2432518	-117.6248	-0.2130255	0.4562773	FAM50A
cg00532335	0.1707928	-117.485	-0.2013389	0.3721317	LGI1
cg18963171	0.180401	-117.48	-0.2033888	0.3837898	C21orf121
cg01980222	0.3366316	-117.4435	-0.2164514	0.5530831	TREM2
cg02786019	0.36738	-117.3146	-0.215239	0.5826191	TRPV6
cg19278780	0.4362242	-117.3073	-0.2092771	0.6455013	SIT1
cg00962459	0.3886895	-117.1411	-0.2137523	0.6024418	PROKR1
cg17099569	0.3002848	-117.1185	-0.2160727	0.5163575	GLI2
cg15590780	0.2819683	-117.0747	-0.2153719	0.4973402	USH2A
cg16324018	0.4672239	-116.9069	-0.2048556	0.6720795	RBED1
cg03112433	0.2441818	-116.8554	-0.2123937	0.4565755	PFTK1
cg01656853	0.2297923	-116.7687	-0.2107142	0.4405065	FUT2
cg09018824	0.294469	-116.7538	-0.2155787	0.5100477	DKFZP686A01247
cg22436411	0.4001249	-116.6778	-0.2124637	0.6125886	FCGR2B
cg16742703	0.4199172	-116.6057	-0.2105422	0.6304594	KLK3
cg18438300	0.4371387	-116.5059	-0.2085438	0.6456825	FZD9
cg24262376	0.3247426	-116.4895	-0.2157514	0.540494	SCNM1
cg18431127	0.3228939	-116.4114	-0.2156879	0.5385817	EPB42
cg27378216	0.4446658	-116.3879	-0.2075265	0.6521923	SETBP1
cg13613682	0.2796692	-116.3723	-0.2146121	0.4942813	ZIC3
cg07905963	0.2345111	-116.357	-0.2108769	0.445388	CYP2A13
cg09272256	0.4036466	-116.1605	-0.2117366	0.6153831	AKR1C4
cg23181133	0.3092856	-116.1036	-0.2153336	0.5246192	CEACAM3
cg17267907	0.2626185	-115.9668	-0.2131735	0.4757919	DEFA1
cg26227465	0.3429482	-115.9469	-0.2150102	0.5579584	IFNG
cg06850526	0.3746495	-115.8998	-0.2136448	0.5882943	MGC15523
cg01361446	0.4134355	-115.8042	-0.2105463	0.6239817	IL2RG
cg23022999	0.4288354	-115.7629	-0.2089132	0.6377487	FLJ45909
cg17589341	0.2840144	-115.4474	-0.2139812	0.4979957	SLC14A1
cg24217877	0.41863	-115.4177	-0.2097197	0.6283497	FLJ44674
cg18787783	0.476922	-115.1661	-0.2020949	0.6790169	MMP19

cg12616487	0.4832084	-115.1636	-0.2011156	0.684324	EML3
cg05209917	0.1903726	-115.078	-0.2030104	0.393383	CNTN6
cg20392764	0.4430154	-114.8254	-0.2065156	0.649531	ASCL2
cg12971694	0.3941404	-114.7729	-0.2113786	0.6055189	CD72
cg22038738	0.3545648	-114.7126	-0.2135827	0.5681474	PLAT
cg24870273	0.4054174	-114.5702	-0.210282	0.6156994	STK19
cg05064181	0.24684	-114.4726	-0.2104155	0.4572555	ABLIM1
cg07456201	0.3168493	-114.3746	-0.2138615	0.5307108	UMOD
cg00754617	0.3329361	-114.3332	-0.2137726	0.5467087	SPACA4
cg12089698	0.3075349	-114.1928	-0.2135828	0.5211176	SPATC1
cg06488505	0.413513	-114.0884	-0.2091411	0.6226541	DKFZp667M2411
cg22802439	0.3676484	-114.0832	-0.212473	0.5801215	UPK1A
cg15585987	0.250796	-114.0222	-0.210355	0.461151	SNTG1
cg27159719	0.2206025	-113.9762	-0.2068462	0.4274487	TMEM71
cg26081812	0.4121295	-113.847	-0.2090694	0.6211989	IL5
cg23937047	0.4051587	-113.8096	-0.2096676	0.6148263	PAGE1
cg22158923	0.3037506	-113.4575	-0.2128331	0.5165837	FLJ39237
cg19103704	0.287647	-113.4575	-0.2123043	0.4999513	FCGBP
cg22518733	0.226928	-113.3358	-0.2070733	0.4340013	CCL3
cg19906550	0.4063135	-113.2619	-0.2091183	0.6154318	SLC22A18
cg21880903	0.2077263	-113.1209	-0.2041112	0.4118375	KLB
cg21414251	0.19662	-113.1209	-0.2022416	0.3988616	OR12D2
cg20611872	0.36682	-113.0043	-0.2115834	0.5784035	XAGE3
cg02990033	0.2299698	-112.9202	-0.2070567	0.4370264	GFRA4
cg21621114	0.2755835	-112.7272	-0.2110127	0.4865962	CRX
cg25322008	0.3080801	-112.6716	-0.2122085	0.5202886	OR51E2
cg04201347	0.3184956	-112.6616	-0.2123231	0.5308187	PRRG2
cg06731599	0.2451831	-112.4524	-0.208321	0.4535041	ATP6AP2
cg04578090	0.3681703	-112.4388	-0.2110311	0.5792013	PROCA1
cg10159529	0.4587007	-112.4235	-0.2026083	0.661309	IL5RA
cg09637363	0.3914176	-112.1768	-0.2093973	0.6008149	CCND1
cg25341726	0.423559	-112.15	-0.206559	0.630118	IL27
cg14719352	0.351285	-112.1347	-0.2114304	0.5627154	FUT1
cg08390254	0.2731029	-111.9043	-0.2100839	0.4831868	ATP1A2
cg03160508	0.3035048	-111.7152	-0.2112271	0.5147318	RHOD
cg06190732	0.2935867	-111.5064	-0.2107349	0.5043216	SERPINA3
cg20322862	0.2588601	-111.5032	-0.2086696	0.4675298	TGIF
cg07042144	0.4110395	-111.4052	-0.2071592	0.6181987	NALP12
cg18780401	0.2246849	-111.3483	-0.2048577	0.4295426	OTUD5
cg21518947	0.2705692	-111.3416	-0.2093886	0.4799577	CABP4
cg09936839	0.3503204	-111.2692	-0.2106991	0.5610195	SIRT6
cg13897627	0.4303222	-111.1436	-0.2050301	0.6353524	FLJ44674
cg14345676	0.3869375	-111.1389	-0.2088261	0.5957636	HRH2
cg02150910	0.262748	-111.0421	-0.2085384	0.4712864	GZMH

cg27117399	0.3861232	-110.8832	-0.2086567	0.5947799	CNDP1
cg15775914	0.4565816	-110.6033	-0.2014571	0.6580387	CHML
cg10832945	0.3580008	-110.5485	-0.2098077	0.5678085	FLJ45964
cg09970593	0.2644162	-110.4949	-0.2081425	0.4725587	OPRD1
cg12878228	0.3471887	-110.4938	-0.2100876	0.5572764	PRSS1
cg06882926	0.4067715	-110.2931	-0.2065978	0.6133693	OR1G1
cg18390025	0.3877318	-110.0319	-0.2078254	0.5955572	ELOVL3
cg26692016	0.4461018	-110.0191	-0.2023121	0.6484139	APOBEC1
cg03936963	0.2323141	-109.8807	-0.2044002	0.4367142	TMEM29
cg11846968	0.4272417	-109.8115	-0.2042671	0.6315088	PLUNC
cg11009736	0.2109412	-109.6896	-0.2012562	0.4121974	MARCO
cg00443307	0.3782898	-109.6038	-0.2080342	0.586324	KLRG1
cg25384595	0.2629524	-109.5314	-0.2071114	0.4700637	LILRA1
cg01182697	0.4247849	-109.3134	-0.2041028	0.6288877	TMEM59
cg27401095	0.2105136	-109.1989	-0.2006992	0.4112128	ZNF324
cg10818781	0.4167301	-109.02	-0.2046312	0.6213614	PHB2
cg14724265	0.3494235	-108.8837	-0.2085808	0.5580043	PPEF2
cg18256128	0.2619355	-108.8251	-0.2063367	0.4682722	PDK3
cg14696870	0.4230393	-108.8085	-0.203845	0.6268843	FCER1A
cg02818322	0.3541399	-108.7157	-0.2082938	0.5624337	MAGEC3
cg19282782	0.4057254	-108.1754	-0.2048806	0.6106061	IL27RA
cg01999333	0.3696276	-108.0131	-0.2070633	0.5766909	CASP14
cg23110514	0.2517954	-107.8664	-0.2045091	0.4563045	LCE3E
cg18994063	0.3365185	-107.5578	-0.207603	0.5441215	TIMD4
cg05823029	0.3516824	-107.4324	-0.2072014	0.5588838	ATP4B
cg20430101	0.3442026	-107.3817	-0.2073276	0.5515302	RBM10
cg03840259	0.3858268	-107.0972	-0.205377	0.5912039	GRAP2
cg17504145	0.4119817	-106.8199	-0.2031883	0.61517	CRYBB2
cg18669381	0.2614284	-106.5034	-0.2040349	0.4654633	ARHGEF19
cg11004890	0.2414766	-106.4978	-0.2021128	0.4435894	SLC4A11
cg03391568	0.4366085	-106.3529	-0.200381	0.6369895	PTHR1
cg13739417	0.2994902	-105.7342	-0.2054863	0.5049765	IL8RB
cg06384491	0.3142538	-105.5762	-0.2057064	0.5199602	SPIN2
cg13828758	0.2928718	-105.4059	-0.2049251	0.4977968	NDN
cg11096993	0.3143871	-104.9393	-0.2051059	0.5194929	ACY3
cg00687674	0.270415	-104.6424	-0.2029083	0.4733233	TMEM84
cg23936476	0.2590616	-104.4824	-0.2018541	0.4609157	BEX1
cg13943564	0.3514064	-104.4269	-0.2044679	0.5558743	SERPINB2
cg06353345	0.3180651	-104.3446	-0.2045899	0.522655	OR51B4
cg16463460	0.2783713	-104.329	-0.2031229	0.4814942	WT1
cg00689340	0.3644822	-103.9496	-0.2036179	0.5681001	RTKN
cg25229305	0.3095926	-103.8654	-0.2039752	0.5135677	KCNK18
cg10500909	0.4139886	-103.8084	-0.2004172	0.6144058	CYP11B2
cg19635695	0.3247553	-103.8031	-0.204129	0.5288842	PDE6C
cg20625138	0.4020716	-103.7557	-0.2013245	0.6033961	UTS2

cg03604278	0.2950823	-103.5598	-0.2032133	0.4982956	CIDEC
cg05254049	0.2991396	-103.4168	-0.2032268	0.5023664	SYN1
cg06494770	0.339486	-103.3749	-0.2036805	0.5431665	KLHL13
cg15320474	0.3831082	-103.3643	-0.2022066	0.5853148	UBD
cg07300408	0.2913184	-103.196	-0.2026886	0.494007	RNASE11
cg04019407	0.3609298	-102.9551	-0.2028156	0.5637454	GIP
cg04991214	0.3874567	-102.8525	-0.2014892	0.5889459	PFDN2
cg04995095	0.321623	-102.6804	-0.2030236	0.5246466	CD300E
cg04545516	0.2746108	-102.6372	-0.2011963	0.4758072	SEMG1
cg12753858	0.3694146	-102.1707	-0.2017805	0.5711951	NUT
cg25553916	0.3203452	-102.0772	-0.2024332	0.5227784	FLJ22318
cg18680834	0.3144322	-101.9048	-0.2021711	0.5166033	ZNF536
cg18960218	0.3793598	-101.6437	-0.2008338	0.5801935	SLC7A7
cg07265310	0.3464832	-101.3166	-0.2016335	0.5481167	K5B
cg05134796	0.2820622	-101.0486	-0.2000785	0.4821408	HNRPDL
cg09837803	0.3251485	-100.8887	-0.2013219	0.5264704	IL16
cg09778422	0.3006437	-100.741	-0.2006476	0.5012913	MSN
cg17971003	0.3074713	-100.7056	-0.2008305	0.5083018	SLN
cg12499211	0.350787	-100.4815	-0.2007632	0.5515503	SH2D2A
cg09424896	0.3432841	-100.4205	-0.200826	0.5441101	CIB3
cg15778232	0.3275256	-99.61754	-0.2001103	0.5276359	PHB2
cg04902405	0.6851214	104.9132	0.2031916	0.4819297	ZC3H11A
cg24134767	0.6365968	105.863	0.206039	0.4305578	HTR3A
cg15233681	0.6786079	106.9994	0.2056396	0.4729684	IL1R1
cg04802221	0.6273745	108.8802	0.2088775	0.418497	LOC283849
cg03642518	0.6045561	116.9631	0.2156438	0.3889123	PNOC
cg12556134	0.5137188	119.4144	0.2101969	0.3035219	TGIF2
cg12177001	0.7357199	122.6466	0.2149816	0.5207384	IFI27
cg02490034	0.6186633	123.323	0.2214881	0.3971752	MEST
cg18829411	0.5263433	123.5321	0.2149111	0.3114323	HMGN1
cg10559803	0.6209886	123.8437	0.2219911	0.3989975	RALGPS2
cg04640913	0.6167002	123.8703	0.2219017	0.3947985	CDH22
cg09191626	0.5599448	124.8351	0.2193908	0.340554	SPON1
cg21279601	0.4864121	126.471	0.2115493	0.2748629	DRD5
cg10150813	0.5208944	128.6514	0.218059	0.3028354	KIAA0746
cg16616769	0.7574651	128.8359	0.2174688	0.5399963	MGC35048
cg02506908	0.6525798	133.2761	0.2300909	0.4224889	HPD
cg12232463	0.662165	135.4024	0.2316071	0.4305579	LONRF2
cg13906813	0.6313448	135.8797	0.2321548	0.3991899	HLA-DPA1
cg04376312	0.5367144	135.8797	0.2253469	0.3113675	MYLK
cg00431549	0.5617676	136.4321	0.2282754	0.3334922	MGP
cg13828047	0.4396805	138.7834	0.211516	0.2281644	MPI
cg05253327	0.7760009	141.7108	0.2249575	0.5510435	B3GNT1
cg20748065	0.8302547	141.7108	0.2113233	0.6189314	POR
cg15958424	0.7081825	353.1397	0.4396717	0.2685107	ACPP

cg24101578	0.7719321	353.1397	0.4322182	0.3397138	CDH22
cg10861599	0.666178	353.1397	0.3841713	0.2820067	TNFSF4
cg22740835	0.6921056	353.1397	0.3779101	0.3141955	DDR2
cg03096975	0.5436484	353.1397	0.3701454	0.1735029	EML2
cg16517394	0.5742987	353.1397	0.369568	0.2047307	TNFSF4
cg17105014	0.542862	353.1397	0.3645779	0.1782841	GYPC
cg08831348	0.6368999	353.1397	0.3598774	0.2770225	EML2
cg24739326	0.5351676	353.1397	0.3502785	0.1848891	CHST8
cg14409083	0.5821207	353.1397	0.3435445	0.2385762	EMP1
cg01346152	0.5881367	353.1397	0.3371598	0.2509769	DHRS3
cg27301343	0.4755825	353.1397	0.3303157	0.1452667	EML2
cg24122922	0.7523098	353.1397	0.3296262	0.4226836	C20orf39
cg18342279	0.4330666	353.1397	0.3265792	0.1064874	ZAR1
cg02806777	0.6353467	353.1397	0.3221392	0.3132075	PGLYRP1
cg18172186	0.8356723	353.1397	0.3151796	0.5204926	KIAA1913
cg06204948	0.5396023	353.1397	0.3132873	0.226315	MARK2
cg02000005	0.448947	353.1397	0.3099881	0.1389588	CRIP1
cg09595479	0.4899674	353.1397	0.3095031	0.1804643	PRPH
cg08624249	0.7860634	353.1397	0.3070333	0.4790302	KIAA0889
cg13959523	0.3949041	353.1397	0.3061634	0.0887407	CHST8
cg11397854	0.4415281	353.1397	0.30491	0.1366182	IQSEC1
cg13547644	0.4793745	353.1397	0.3046389	0.1747356	ACTA1
cg23418591	0.414988	353.1397	0.3018967	0.1130913	FLJ90166
cg13351161	0.5371094	353.1397	0.2975256	0.2395838	SCARA3
cg14371590	0.6090864	353.1397	0.2973095	0.3117769	SLC26A10
cg18702197	0.6629874	353.1397	0.2967826	0.3662047	HOXD3
cg20427879	0.4925303	353.1397	0.2911081	0.2014222	EML2
cg03562120	0.6447425	353.1397	0.290422	0.3543206	WISP2
cg17339202	0.5675157	353.1397	0.2894474	0.2780684	SYNC1
cg12564453	0.8201361	353.1397	0.2864868	0.5336493	CETP
cg05485060	0.6979289	353.1397	0.2860342	0.4118947	CTNNAL1
cg07285276	0.8080006	353.1397	0.2859217	0.5220789	RAPGEF1
cg16363586	0.5966949	353.1397	0.2850854	0.3116095	BST2
cg01103836	0.9568336	353.1397	0.2818754	0.6749582	MYO9B
cg03852144	0.5468324	353.1397	0.2805806	0.2662518	GLRX
cg11554605	0.7958225	353.1397	0.2779832	0.5178393	ASB4
cg05342835	0.6499318	353.1397	0.2774869	0.3724449	SYNC1
cg20654468	0.7621069	353.1397	0.2758488	0.4862581	LPXN
cg17890764	0.6811544	353.1397	0.2752997	0.4058547	ITIH4
cg23283495	0.386691	353.1397	0.2744951	0.1121959	IRF6
cg06507244	0.720916	353.1397	0.2744354	0.4464805	DHX32
cg03752628	0.5105612	353.1397	0.2691225	0.2414387	PTGFRN
cg13763232	0.7476215	353.1397	0.2646026	0.4830189	SLC6A6
cg24459563	0.7465445	353.1397	0.2625021	0.4840423	CACNG1
cg12052765	0.3939792	353.1397	0.2621455	0.1318336	CHAT

cg25947945	0.5703273	353.1397	0.2597199	0.3106074	LAD1
cg13185308	0.6526365	353.1397	0.2596421	0.3929945	ABCC8
cg19224278	0.5722235	353.1397	0.2573549	0.3148687	ALDH1A3
cg27114026	0.7864946	353.1397	0.2556957	0.5307989	ELA1
cg04099420	0.5972992	353.1397	0.2554477	0.3418514	RIPK1
cg07236190	0.7394531	353.1397	0.2538471	0.4856061	AMDHD1
cg23855989	0.4713063	353.1397	0.2518273	0.2194789	AQP5
cg24315815	0.7748262	353.1397	0.2505897	0.5242364	PLSCR4
cg00240880	0.8343865	353.1397	0.2481524	0.5862341	WISP2
cg16612562	0.6838341	353.1397	0.2480577	0.4357764	RRP22
cg15127733	0.6851814	353.1397	0.2478255	0.4373559	HSPA12B
cg02498063	0.6026551	353.1397	0.2466288	0.3560262	CCDC60
cg13140267	0.3546688	353.1397	0.244432	0.1102368	ASCC3L1
cg17791651	0.5404724	353.1397	0.2426728	0.2977996	POU3F1
cg18392482	0.7868043	353.1397	0.2414362	0.545368	AMDHD1
cg22980351	0.5754073	353.1397	0.237946	0.3374613	WDR40B
cg04700814	0.5303264	353.1397	0.2362251	0.2941013	HEXIM1
cg13431205	0.85387	353.1397	0.2338204	0.6200497	RB1
cg19428417	0.4679749	353.1397	0.2321961	0.2357788	RRAD
cg26509022	0.4331208	353.1397	0.2297455	0.2033753	ALDH1A3
cg08858521	0.4449888	353.1397	0.2258717	0.219117	WFIKKN1
cg04123507	0.8262188	353.1397	0.2251241	0.6010947	KRTHB6
cg17067528	0.3034729	353.1397	0.2217402	0.08173271	IER3
cg25277950	0.3635853	353.1397	0.2166563	0.146929	EML2
cg02674804	0.8450751	353.1397	0.2155262	0.6295489	REEP6
cg26764244	0.3664221	353.1397	0.2154571	0.150965	GNG12
cg22215728	0.4044863	353.1397	0.2147191	0.1897672	FHIT
cg09963123	0.3581379	353.1397	0.2135014	0.1446365	KLF3
cg12024292	0.3496956	353.1397	0.2071196	0.142576	ASTN2
cg18691434	0.2879029	353.1397	0.2027121	0.08519084	GPC2
cg24330042	0.2597553	353.1397	0.202468	0.05728735	GSTT1
cg04502814	0.331757	353.1397	0.2014676	0.1302894	SEPP1

Supplementary table 5 – Probes differentially methylated between benign tumours and normal tissue

TargetID	Benign.AVG_Beta	Benign.DiffScore	Benign.Delta Beta	Normal medulla.AVG_Beta	SYMBOL
cg13021192	0.2104471	-346.9279	-0.3900689	0.6005161	CTSZ
cg16179125	0.2709256	-346.9279	-0.3722745	0.6432001	CTSZ
cg14204735	0.1013565	-346.9279	-0.3647891	0.4661456	CYB561
cg25856811	0.3712465	-346.9279	-0.3467103	0.7179568	SPRR3
cg01335367	0.1801547	-346.9279	-0.3431962	0.5233509	C12orf34
cg27619475	0.2146884	-346.9279	-0.3417978	0.5564861	SLC16A5
cg26164184	0.4777619	-346.9279	-0.3371533	0.8149152	FCN2
cg06536578	0.3188367	-346.9279	-0.3313089	0.6501456	JPH4

cg04144768	0.3591121	-346.9279	-0.3199908	0.6791029	DDC
cg26927807	0.3784085	-346.9279	-0.315244	0.6936526	BTBD2
cg13726507	0.5175312	-346.9279	-0.310887	0.8284182	CTAG2
cg14992108	0.4161512	-346.9279	-0.3041069	0.7202581	SNTB1
cg15503752	0.4269353	-346.9279	-0.3027939	0.7297292	ST6GALN AC1
cg18149207	0.4359973	-346.9279	-0.2973538	0.7333511	RORC
cg22575540	0.4691614	-346.9279	-0.286698	0.7558594	TRIM54
cg24024214	0.5488926	-346.9279	-0.2726568	0.8215494	BTNL8
cg21948655	0.5319272	-346.9279	-0.2669464	0.7988736	SMCP
cg14256699	0.611236	-346.9279	-0.2491527	0.8603887	SOST
cg25813714	0.5948278	-346.9279	-0.2487658	0.8435937	CYP4F12
cg17357062	0.5827428	-346.9279	-0.2430122	0.8257549	FCN1
cg05444024	0.6122809	-346.9279	-0.239568	0.8518489	FUT6
cg00644033	0.7133662	-346.9279	-0.2042003	0.9175664	MUC3B
cg25203980	0.5628686	-336.1616	-0.2470109	0.8098795	CENTB5
cg23815000	0.4394752	-332.711	-0.28772	0.7271953	LCN1
cg06639544	0.4575441	-328.085	-0.2809711	0.7385153	OR7A5
cg07824742	0.4900818	-321.8413	-0.2689893	0.7590712	DBH
cg14706739	0.3974647	-319.3188	-0.2951113	0.6925761	EPB49
cg00698688	0.5606926	-317.2351	-0.2433748	0.8040674	SULT2B1
cg22374142	0.1741104	-314.7481	-0.323566	0.4976765	HSF4
cg26672426	0.3171419	-309.9415	-0.3090831	0.626225	PTGES
cg08626653	0.426699	-309.6932	-0.2843006	0.7109996	FLJ37538
cg21277505	0.6038447	-309.5274	-0.2252859	0.8291306	LOC28436 1
cg04138756	0.5287803	-306.2257	-0.2518503	0.7806306	SPRR3
cg20154346	0.3333699	-306.0706	-0.3046162	0.6379861	RAI2
cg08573687	0.3877482	-306.0109	-0.292969	0.6807172	TH
cg04132607	0.6555799	-304.8479	-0.2035445	0.8591244	GATA5
cg01103730	0.4112587	-302.9466	-0.2861116	0.6973703	IL20
cg25221254	0.4515538	-302.0631	-0.2748903	0.7264441	ASAH3
cg21450627	0.5872474	-296.1111	-0.2284113	0.8156587	PSD4
cg19728223	0.225508	-294.2858	-0.3128706	0.5383787	KCNQ1
cg20334738	0.3532212	-292.761	-0.2956606	0.6488818	MAB21L2
cg21457804	0.4918227	-288.3329	-0.2586918	0.7505146	CT45-2
cg24407065	0.5727598	-283.6278	-0.2305059	0.8032658	BLZF1
cg10758292	0.5234414	-280.5266	-0.2464172	0.7698587	DEFA1
cg08450982	0.2877368	-280.0028	-0.3010006	0.5887374	NUMBL
cg10604646	0.2317675	-279.139	-0.3053807	0.5371482	RGS5
cg25119415	0.4101902	-278.2538	-0.2777508	0.687941	MNDA
cg08684473	0.4940006	-276.4854	-0.2543827	0.7483833	LILRB5
cg12489960	0.3885144	-271.8225	-0.280333	0.6688474	SGCB
cg17820828	0.4634666	-269.8796	-0.2610738	0.7245404	KCNQ1
cg05348870	0.5276853	-269.2685	-0.2417111	0.7693964	TNFSF14

cg06101324	0.1945688	-269.0948	-0.3008547	0.4954236	SPRR1A
cg03602500	0.4728722	-268.082	-0.2578252	0.7306974	FLJ00060
cg18129786	0.5897987	-267.1189	-0.2200684	0.8098671	ZNF445
cg01515887	0.4723566	-263.3627	-0.2563873	0.7287439	SAA2
cg06123346	0.3325641	-263.3597	-0.287431	0.6199951	ATP4A
cg18780284	0.4090126	-263.0738	-0.2723809	0.6813936	SPRR1B
cg24670715	0.2028472	-261.2144	-0.2967958	0.4996431	ANGPT2
cg12513481	0.2680113	-256.874	-0.2922513	0.5602626	SCAP1
cg27285599	0.5226473	-255.1927	-0.2389331	0.7615805	FLJ13841
cg02442161	0.5106237	-254.9574	-0.2425034	0.7531271	PI3
cg10805676	0.4553327	-254.9155	-0.2579924	0.7133251	MRPL28
cg10062065	0.3858119	-250.8833	-0.2725392	0.6583511	APEG1
cg02067021	0.4248185	-250.2057	-0.2637613	0.6885798	DNAJC5B
cg21372914	0.4586053	-250.2057	-0.2554229	0.7140282	CLEC4M
cg15652212	0.4314117	-249.5957	-0.2619637	0.6933754	FLJ90586
cg24992780	0.5810164	-247.5042	-0.2176549	0.7986712	OR7C1
cg04323365	0.5014944	-247.4849	-0.2426696	0.744164	GJB1
cg05766474	0.6125528	-244.8388	-0.2059293	0.818482	CCL16
cg12815142	0.4116522	-244.5072	-0.2644736	0.6761258	SPAG7
cg19954000	0.2142081	-244.4774	-0.2877782	0.5019863	FGF1
cg16990174	0.2611873	-244.1961	-0.2863007	0.547488	RYBP
cg18138484	0.6244271	-243.9305	-0.2013975	0.8258247	CABP2
cg00334507	0.3484557	-243.0216	-0.2758473	0.6243029	MVP
cg22294577	0.5920626	-242.2158	-0.2123131	0.8043756	SLC26A3
cg24694549	0.3685108	-241.5891	-0.2717899	0.6403007	GRIP1
cg06436504	0.4052124	-241.1902	-0.2644283	0.6696407	DOC1
cg06832950	0.5504597	-240.7483	-0.2255528	0.7760125	SPG3A
cg06244417	0.5488982	-240.2652	-0.2258878	0.774786	FCN1
cg15711744	0.5316629	-239.1629	-0.2308604	0.7625234	ANP32D
cg14696820	0.3238718	-238.0965	-0.2770775	0.6009493	LCE1A
cg26149550	0.5701655	-238.0965	-0.2183807	0.7885463	KLK15
cg22784047	0.4287483	-237.8129	-0.2578496	0.6865979	MVP
cg19917856	0.2290399	-237.5689	-0.2838159	0.5128558	LOC34289<br>7
cg14894144	0.1961975	-237.5399	-0.2832565	0.479454	LAMA3
cg10677144	0.461436	-237.4671	-0.2498117	0.7112477	MYOM1
cg14845091	0.3521797	-237.1861	-0.2724843	0.624664	ADPRHL1
cg10275770	0.1606631	-235.7296	-0.2793918	0.4400549	ICAM2
cg26946769	0.5605479	-235.0579	-0.2204846	0.7810324	MAPK4
cg10990993	0.323761	-233.392	-0.274772	0.5985331	MLH1
cg20373326	0.4240545	-233.1244	-0.2569451	0.6809996	HSD17B2
cg03221619	0.2741154	-232.4173	-0.2791763	0.5532917	FCER2
cg20544605	0.396136	-230.3184	-0.2615652	0.6577012	SORBS2
cg14333565	0.5782117	-230.2386	-0.2132549	0.7914666	NRTN
cg26292028	0.4373611	-229.9639	-0.2526261	0.6899872	FLJ37587

cg07792737	0.611065	-226.9027	-0.2012513	0.8123163	NPIP
cg13521229	0.2775732	-225.9915	-0.2755464	0.5531197	JOSD2
cg07997737	0.298678	-225.5685	-0.2736146	0.5722926	NRTN
cg19949550	0.5053086	-225.0674	-0.2335145	0.7388231	ASB2
cg27377450	0.6037906	-224.5415	-0.2030174	0.8068079	ARHGEF1 8
cg25477904	0.6060557	-223.2005	-0.2018387	0.8078945	PSG1
cg02351381	0.1905611	-222.021	-0.2736334	0.4641945	C12orf34
cg21686987	0.560672	-220.8819	-0.2158061	0.7764781	CTRB1
cg14182690	0.5827848	-218.6583	-0.2081116	0.7908964	RUNX3
cg17907567	0.3601196	-216.887	-0.261674	0.6217936	HAMP
cg05671018	0.4316859	-215.1837	-0.2476962	0.679382	LYSMD2
cg01530101	0.3684948	-215.079	-0.2594886	0.6279835	KCNQ1DN
cg15516226	0.595266	-214.9299	-0.2028729	0.7981389	BTNL9
cg24901474	0.2824853	-214.7753	-0.2691351	0.5516204	RGS5
cg04505023	0.4583341	-214.552	-0.2414088	0.6997429	SPRR1A
cg13656062	0.5296136	-213.608	-0.2224841	0.7520977	CYP4F2
cg06277657	0.2103478	-213.0913	-0.2692161	0.4795639	DGKI
cg15928132	0.3461951	-212.3166	-0.2613719	0.607567	CCKAR
cg08448751	0.4132011	-207.9783	-0.2482412	0.6614423	SEMA3G
cg12619162	0.5558746	-207.7145	-0.2127267	0.7686013	FXYD4
cg09467501	0.1186364	-207.4925	-0.2534768	0.3721131	PYY
cg16772207	0.3647335	-205.7846	-0.2554984	0.620232	MYT1
cg01894895	0.5709264	-205.4082	-0.2073769	0.7783033	ANXA1
cg01657380	0.4256069	-204.6896	-0.2442653	0.6698722	NPFF
cg27235662	0.5414001	-204.4026	-0.2157302	0.7571304	CLDN16
cg16175263	0.3013916	-203.4989	-0.261538	0.5629296	TNFRSF10 C
cg27087809	0.5357909	-201.862	-0.2163602	0.7521511	ACSBG1
cg24490338	0.4924484	-200.8472	-0.2274479	0.7198963	TPM3
cg16122592	0.3921765	-200.2936	-0.2483783	0.6405548	MAGEB6
cg09027725	0.3794625	-199.1981	-0.2499065	0.6293691	COX4I2
cg09701102	0.5388152	-198.7181	-0.2143067	0.7531219	NDUFV1
cg12456510	0.4425902	-196.4217	-0.2369726	0.6795629	TFF2
cg22189286	0.4574122	-195.873	-0.2335406	0.6909527	HSPB8
cg06236276	0.5474498	-195.5097	-0.210653	0.7581028	SLC22A2
cg27257987	0.4029075	-195.4094	-0.2441135	0.6470209	PSG4
cg14287742	0.5367789	-193.9374	-0.2130175	0.7497964	BLZF1
cg15780361	0.2950824	-193.582	-0.2563084	0.5513908	ALS2CR11
cg01643624	0.5058244	-192.975	-0.2208005	0.726625	C11orf16
cg22190114	0.4913115	-192.2138	-0.2240699	0.7153814	NALP8
cg13204181	0.3865069	-191.1132	-0.2446595	0.6311664	GH1
cg16051685	0.518723	-190.6624	-0.2165015	0.7352245	TRIM63
cg19728577	0.5710701	-190.4075	-0.2018861	0.7729563	GUCA2B
cg07651914	0.3212779	-189.5981	-0.2519409	0.5732188	CLDN15

cg24697329	0.5257614	-188.6223	-0.2138184	0.7395797	ARHGEF4
cg19042947	0.3972694	-188.2504	-0.241434	0.6387034	SERPINA4
cg07525077	0.3229927	-188.0964	-0.2509144	0.5739071	RNASE3
cg06220755	0.3739845	-186.5881	-0.2441315	0.618116	RAI2
cg05670596	0.2356864	-184.8539	-0.2521297	0.4878161	CCRL2
cg23881725	0.182071	-183.5698	-0.2476128	0.4296838	DLEC1
cg00321478	0.4216847	-183.5528	-0.2348123	0.6564969	CRB1
cg11599505	0.5637234	-183.3434	-0.2013048	0.7650282	C20orf102
cg25214346	0.4195328	-182.4745	-0.2346332	0.654166	NR1I3
cg06351503	0.4600652	-182.1908	-0.2265631	0.6866283	RDBP
cg16986846	0.3474799	-182.14	-0.2449919	0.5924717	SCGB2A1
cg11719283	0.4252513	-182.0244	-0.2333455	0.6585968	ZNF574
cg02833725	0.4177649	-181.9124	-0.2346318	0.6523967	ISG20L2
cg15626350	0.2182042	-181.6879	-0.2492577	0.467462	ESR1
cg01484156	0.3015344	-181.5028	-0.2485903	0.5501247	NCALD
cg18056600	0.1282647	-181.4715	-0.2366755	0.3649402	ZMYND15
cg24750391	0.2393948	-181.1006	-0.2496414	0.4890362	PON3
cg10569414	0.4647737	-180.4836	-0.2247061	0.6894798	C21orf121
cg13019092	0.5221399	-179.0064	-0.2106386	0.7327785	PDZK1
cg19368582	0.413243	-178.8186	-0.2337894	0.6470323	MMRN2
cg05440289	0.5524345	-178.0706	-0.2022669	0.7547014	IVL
cg24735489	0.3873025	-177.4804	-0.2371832	0.6244857	CDSN
cg19807685	0.4492089	-177.3938	-0.2264022	0.6756111	HSD17B2
cg00594952	0.3206256	-176.7426	-0.2443263	0.564952	RIMS3
cg22341310	0.1865462	-176.2602	-0.2427874	0.4293335	ZNF541
cg23412777	0.4094735	-176.2506	-0.2330486	0.6425221	PYGO1
cg19712821	0.3235543	-175.3622	-0.2432442	0.5667985	KSP37
cg15210427	0.2689357	-175.3558	-0.2458967	0.5148324	CST9L
cg24607398	0.2762938	-175.0069	-0.2455255	0.5218193	MLH1
cg19233472	0.5110571	-174.3844	-0.2112976	0.7223547	FOXI1
cg27020690	0.1232504	-174.3705	-0.2299435	0.3531938	TERC
cg10127415	0.2812916	-174.3701	-0.2449579	0.5262495	MAGEB6
cg26063872	0.4112043	-173.3734	-0.2311999	0.6424041	DEFB123
cg04457051	0.3674446	-173.3128	-0.2374682	0.6049128	SCOC
cg19982860	0.3962917	-172.7653	-0.233184	0.6294757	IFNA21
cg09414535	0.366476	-172.7047	-0.2372223	0.6036983	GRIP1
cg01982597	0.4611544	-172.411	-0.2214526	0.682607	PGBD3
cg04711324	0.324077	-171.8969	-0.2410144	0.5650914	RIT2
cg11158374	0.490357	-171.4287	-0.2147127	0.7050698	TFF2
cg27090216	0.1291898	-171.2134	-0.2289005	0.3580904	TNFRSF10C
cg01663968	0.1182204	-170.2253	-0.2252972	0.3435176	CTSZ
cg21065959	0.433426	-169.7018	-0.2253544	0.6587805	LCE1E
cg16176600	0.5301658	-169.5615	-0.2043587	0.7345245	FRK
cg22510822	0.4161715	-169.1814	-0.2280443	0.6442158	OR1E2

cg14662756	0.4175419	-168.0199	-0.227196	0.6447378	NPFF
cg18242139	0.5034879	-167.6739	-0.2099229	0.7134107	ELAVL4
cg25762706	0.3316163	-167.4118	-0.2376055	0.5692218	STMN4
cg04953015	0.5040041	-167.4118	-0.2096751	0.7136792	CHRNA2
cg00622552	0.1492846	-167.1848	-0.2301315	0.379416	ODF3L1
cg24851490	0.2639164	-167.1055	-0.2403304	0.5042468	RNASE2
cg24852661	0.1591931	-166.703	-0.2315902	0.3907833	GOLPH2
cg05215575	0.3277981	-166.3965	-0.2372457	0.5650438	FLJ25410
cg18533225	0.473387	-166.3965	-0.2158688	0.6892558	KLHDC7B
cg25372195	0.4350921	-165.9087	-0.2229902	0.6580823	DCD
cg09299388	0.4836774	-165.8692	-0.21341	0.6970874	PGK2
cg03782453	0.1549342	-164.9917	-0.2294487	0.3843829	FLJ90575
cg25957124	0.2891426	-164.4351	-0.2380763	0.5272189	DNAH3
cg23829949	0.3472882	-164.1505	-0.2341354	0.5814235	ZNF238
cg02130905	0.3759669	-163.9215	-0.2308244	0.6067913	STMN4
cg14238120	0.5053829	-163.2239	-0.2073168	0.7126997	ELA3A
cg18530716	0.1209686	-162.7433	-0.2199779	0.3409466	SLC16A11
cg10052840	0.258314	-162.1236	-0.2368111	0.4951251	SEMA6B
cg10894512	0.4093078	-161.2564	-0.2246709	0.6339787	ACTA2
cg12069042	0.4642432	-160.6127	-0.2147424	0.6789856	PLXNB1
cg13578652	0.4112352	-160.4119	-0.2238944	0.6351296	UBASH3A
cg07597976	0.3543929	-160.0781	-0.2309014	0.5852943	CD19
cg27442349	0.410523	-158.0892	-0.2226816	0.6332046	NFKBIB
cg08260891	0.09013753	-157.3538	-0.2059421	0.2960796	PPGB
cg24516901	0.2321847	-157.3266	-0.2325732	0.4647579	FLJ22746
cg06811800	0.4618821	-157.2983	-0.2134424	0.6753246	ATP4B
cg19717326	0.215987	-157.138	-0.2313664	0.4473534	MYADM
cg02324920	0.292199	-157.0221	-0.2329749	0.5251739	NEURL
cg13180098	0.4223118	-156.0206	-0.2196672	0.641979	RHO
cg12334759	0.4911545	-155.929	-0.2068156	0.6979702	C19orf19
cg12022621	0.3198615	-155.7543	-0.2308777	0.5507392	LAX1
cg17192247	0.1388017	-155.5325	-0.2185797	0.3573814	MAPRE3
cg11739626	0.4947431	-155.1174	-0.2056172	0.7003604	AKT1S1
cg19910382	0.3694064	-155.0941	-0.2262024	0.5956088	FABP1
cg09426307	0.4297995	-154.6875	-0.2176495	0.647449	SEC14L3
cg20011352	0.1370667	-154.5691	-0.217356	0.3544226	GPR124
cg24884084	0.2024592	-154.4923	-0.2280691	0.4305283	SPRR1B
cg03609102	0.3431171	-154.1399	-0.2281147	0.5712318	MUC5B
cg01015871	0.3941997	-154.0733	-0.2225421	0.6167418	MT4
cg15996947	0.3955418	-153.6333	-0.2221027	0.6176445	L2HGDH
cg23260026	0.2755279	-153.6015	-0.230798	0.506326	FSTL3
cg20189782	0.5078824	-153.3955	-0.2018574	0.7097399	MGC2712 1
cg16626670	0.4298975	-152.4013	-0.2163087	0.6462061	CLEC4G
cg25882366	0.1639736	-151.5724	-0.2204336	0.3844072	HOXB2

cg22970435	0.1429012	-151.2213	-0.2159502	0.3588514	SPATS1
cg18979223	0.4482254	-150.8914	-0.2123759	0.6606013	CDKN2B
cg07339138	0.3480766	-150.4436	-0.2252765	0.5733532	CCDC13
cg26583078	0.4495518	-149.8749	-0.2115523	0.6611041	SORBS2
cg24693053	0.2429943	-149.8075	-0.2274085	0.4704029	MFSD7
cg04810997	0.4622066	-149.8075	-0.2092291	0.6714357	TAS2R60
cg18063149	0.4857274	-149.5567	-0.2045333	0.6902608	FMO3
cg13694749	0.4789423	-149.0542	-0.205615	0.6845574	SCN4A
cg19111262	0.2971645	-148.5049	-0.2268166	0.5239811	IGSF9
cg02187357	0.4193723	-148.4157	-0.2155721	0.6349444	TBC1D22B
cg24355048	0.4225328	-148.1152	-0.2149078	0.6374406	CTSG
cg07408456	0.3869863	-148.0136	-0.2196763	0.6066627	PGLYRP2
cg00756887	0.480947	-147.7886	-0.204514	0.685461	PVRL4
cg22194129	0.3675903	-147.6835	-0.2216081	0.5891984	CLEC4C
cg10575735	0.3807281	-147.4866	-0.2200558	0.6007839	SSX4
cg19903229	0.3107573	-146.1467	-0.2246459	0.5354032	C14orf105
cg19384697	0.2753063	-146.0615	-0.2252774	0.5005838	UPK3B
cg24429836	0.2359099	-145.815	-0.2239521	0.459862	LDHD
cg13271951	0.3006275	-145.6367	-0.2246169	0.5252445	FAM57B
cg01305625	0.3043836	-145.34	-0.2242796	0.5286632	PDLIM4
cg04143809	0.3502563	-145.3174	-0.2215959	0.5718522	FLJ39822
cg12951282	0.3938294	-144.5875	-0.2166154	0.6104448	ASGR2
cg15590780	0.2733376	-144.4064	-0.2240026	0.4973402	USH2A
cg02218324	0.4402226	-144.2418	-0.2097588	0.6499814	RSHL1
cg05912121	0.4523341	-144.1295	-0.2076682	0.6600023	TH
cg27337148	0.2158573	-143.577	-0.2206972	0.4365545	CAMK1G
cg04048249	0.4503844	-143.1266	-0.207424	0.6578084	APOC3
cg07412254	0.4194626	-142.742	-0.2120355	0.631498	FLJ14816
cg02423618	0.374014	-142.4934	-0.2175025	0.5915165	SPATA8
cg03973663	0.2125828	-142.1493	-0.2192711	0.4318539	LYN
cg15531099	0.3148871	-141.8257	-0.2213671	0.5362542	LCE1D
cg14179628	0.4289075	-141.7681	-0.2100453	0.6389529	TCEAL7
cg01074640	0.438849	-141.1566	-0.2081381	0.6469871	IFNA17
cg10414946	0.4203833	-140.6043	-0.210576	0.6309593	MS4A2
cg07484827	0.2799526	-140.1045	-0.2208011	0.5007538	CHRNA10
cg24840099	0.3424799	-139.8774	-0.2184411	0.5609211	MSX1
cg15060813	0.2889594	-139.7246	-0.2204679	0.5094273	LRFN3
cg01993576	0.177017	-139.7246	-0.2127395	0.3897566	SLC29A1
cg00392257	0.3892615	-139.2405	-0.2136737	0.6029352	ISG20L2
cg06501790	0.4659923	-138.6373	-0.2020543	0.6680466	SLC34A1
cg04574507	0.443107	-138.5115	-0.2058195	0.6489264	CD1B
cg01550148	0.3297896	-137.6938	-0.2176415	0.5474311	H2AFY
cg13960126	0.4346271	-137.6512	-0.2066005	0.6412276	CRB3
cg02157083	0.4228431	-137.0862	-0.2079717	0.6308148	APOA5
cg04273431	0.1784998	-136.8969	-0.2105686	0.3890684	PRR3

cg04337944	0.260781	-136.7859	-0.2179181	0.4786991	FBLN1
cg09300114	0.2493826	-136.6161	-0.2173494	0.466732	SLC16A5
cg21755709	0.259454	-136.4706	-0.217617	0.477071	C21orf124
cg26884581	0.1860756	-136.44	-0.211307	0.3973826	PYGM
cg25400358	0.3473084	-136.2634	-0.2154966	0.5628049	GPR137
cg07922606	0.2772399	-136.166	-0.2177164	0.4949563	HIST1H3E
cg26581729	0.2364194	-136.1101	-0.2162079	0.4526273	NPDC1
cg27418851	0.4572638	-136.0246	-0.2019466	0.6592104	MBL2
cg02717866	0.4482081	-135.7065	-0.2032315	0.6514395	FLJ32771
cg27566805	0.2731198	-135.5106	-0.2171186	0.4902384	USH2A
cg03468463	0.4416673	-135.5106	-0.2041338	0.6458012	SERPINB12
cg10787197	0.3576747	-135.3105	-0.213998	0.5716727	C6orf105
cg12113132	0.332028	-135.076	-0.2155607	0.5475886	CCNDBP1
cg25072962	0.4186956	-134.2033	-0.2066386	0.6253342	MGC35295
cg05569220	0.3682408	-134.0151	-0.2121979	0.5804387	FLJ44861
cg04962134	0.3900587	-133.9851	-0.2099823	0.600041	TRIM51
cg00714377	0.1883396	-133.9851	-0.2094976	0.3978373	SLA2
cg10322876	0.416801	-133.9176	-0.2066801	0.6234811	CYP2B6
cg06818777	0.1538068	-133.3707	-0.2028821	0.3566888	CHAD
cg18396533	0.2077031	-133.1579	-0.21123	0.4189331	DYDC1
cg18988110	0.2462574	-132.8333	-0.2140851	0.4603425	ATAD4
cg25982743	0.2246599	-132.5112	-0.2123551	0.437015	TIMP4
cg08972170	0.3443215	-131.7344	-0.2123839	0.5567054	Ells1
cg00042156	0.3864616	-131.7344	-0.2088302	0.5952918	MGC16291
cg12781568	0.3446136	-131.7251	-0.2123534	0.556967	WT1
cg15842430	0.4236422	-131.57	-0.2042159	0.627858	FAM12B
cg26420196	0.2586522	-131.43	-0.2134741	0.4721264	GAS6
cg02812142	0.447898	-131.4102	-0.2005511	0.6484491	ACMSD
cg06531741	0.3225929	-131.4055	-0.2132317	0.5358245	HTR3B
cg01837574	0.3219095	-131.397	-0.2132474	0.535157	TRAPPC1
cg05093686	0.2634074	-130.9833	-0.2132335	0.4766409	MAB21L1
cg01785568	0.3387767	-130.6305	-0.211851	0.5506277	MSX1
cg01772980	0.4202167	-130.2258	-0.2037542	0.6239709	SCGB1D1
cg11161873	0.3233706	-130.0176	-0.2121313	0.535502	FLJ39575
cg24628744	0.1970792	-129.7599	-0.2069926	0.4040718	H2AFY
cg07967308	0.427519	-128.9561	-0.2019237	0.6294427	ACP5
cg26415633	0.2569061	-128.1509	-0.2107098	0.4676159	KLK1
cg03003745	0.384182	-127.2908	-0.2058951	0.590077	UNQ473
cg01726767	0.4163908	-127.0831	-0.2021313	0.6185221	LALBA
cg05654163	0.4302045	-127.0426	-0.2002766	0.6304811	SLC39A2
cg10071275	0.3743601	-126.4315	-0.2061804	0.5805405	MYT1
cg11070419	0.3395194	-126.2065	-0.2084939	0.5480133	C4BPA

cg24816455	0.2690404	-125.6907	-0.2090975	0.4781379	SEMA3B
cg21402035	0.3991855	-125.4356	-0.2030181	0.6022036	GALR3
cg06233503	0.3584393	-123.8817	-0.2055662	0.5640055	KCNQ1
cg09971646	0.2062421	-123.7286	-0.2028933	0.4091354	DLK1
cg13125510	0.2642738	-122.2718	-0.2061288	0.4704025	C11orf44
cg09837803	0.3211045	-121.1479	-0.2053659	0.5264704	IL16
cg21432842	0.3335341	-120.9564	-0.2047532	0.5382873	CSF3
cg00518911	0.3086182	-120.8084	-0.2053485	0.5139667	HOXA10
cg00750606	0.3352596	-120.4056	-0.2042258	0.5394855	CDA
cg07643942	0.3592739	-120.37	-0.2028512	0.5621251	LACRT
cg15095327	0.3635907	-120.1664	-0.2023984	0.5659891	IL17RE
cg03343942	0.3341567	-118.3441	-0.2026484	0.5368052	SLC39A5
cg02595219	0.2608487	-118.1288	-0.2024336	0.4632823	KCNE3
cg05636175	0.2299802	-117.8688	-0.2001695	0.4301497	TNFRSF10C
cg07950803	0.3042721	-117.8434	-0.2029534	0.5072255	CD1A
cg00626119	0.3352027	-117.0787	-0.2015838	0.5367865	NTRK1
cg22083047	0.2834398	-114.9885	-0.2004482	0.483888	PRICKLE2
cg04802221	0.6193671	116.2671	0.2008701	0.418497	LOC283849
cg11554605	0.7239587	124.5568	0.2061195	0.5178393	ASB4
cg13587552	0.748615	124.7839	0.203934	0.544681	SCNN1D
cg07047653	0.7612386	125.6195	0.2030588	0.5581798	AQP2
cg16077929	0.7129662	127.0657	0.2090607	0.5039055	CDKL1
cg24427660	0.7163236	128.0183	0.2097554	0.5065683	PNPLA2
cg13763232	0.6952932	130.1387	0.2122743	0.4830189	SLC6A6
cg03562120	0.5632215	132.4722	0.2089009	0.3543206	WISP2
cg27114026	0.7441927	134.5603	0.2133939	0.5307989	ELA1
cg21685427	0.7584045	134.5603	0.2113987	0.5470058	SGK2
cg16363586	0.6576596	348.5116	0.3460501	0.3116095	BST2
cg01346152	0.5650785	348.5116	0.3141015	0.2509769	DHRS3
cg09871315	0.6141825	348.5116	0.3088086	0.305374	HOXA2
cg14409083	0.5375026	348.5116	0.2989264	0.2385762	EMP1
cg26509022	0.5002564	348.5116	0.2968811	0.2033753	ALDH1A3
cg03852144	0.553052	348.5116	0.2868002	0.2662518	GLRX
cg01126560	0.6840987	348.5116	0.2862973	0.3978014	C9orf142
cg24101578	0.6223112	348.5116	0.2825973	0.3397138	CDH22
cg22628873	0.7328217	348.5116	0.2799931	0.4528286	GGT6
cg19224278	0.5911987	348.5116	0.27633	0.3148687	ALDH1A3
cg03605761	0.6316794	348.5116	0.2745783	0.357101	RNF126
cg25101936	0.7741333	348.5116	0.273555	0.5005782	ZBTB16
cg18702197	0.6365814	348.5116	0.2703767	0.3662047	HOXD3
cg08624249	0.7458744	348.5116	0.2668442	0.4790302	KIAA0889
cg06507244	0.7107822	348.5116	0.2643017	0.4464805	DHX32
cg02506908	0.6844155	348.5116	0.2619266	0.4224889	HPD

cg16517394	0.4589661	348.5116	0.2542354	0.2047307	TNFSF4
cg01103836	0.928439	348.5116	0.2534807	0.6749582	MYO9B
cg04106785	0.7395539	348.5116	0.2534598	0.4860941	CDK5R1
cg06204948	0.4790973	348.5116	0.2527823	0.226315	MARK2
cg03271907	0.8411444	348.5116	0.2524876	0.5886568	MGMT
cg15958424	0.5202593	348.5116	0.2517485	0.2685107	ACPP
cg07251788	0.7383964	348.5116	0.2510532	0.4873433	CLTCL1
cg18172186	0.7677502	348.5116	0.2472576	0.5204926	KIAA1913
cg14371590	0.5589874	348.5116	0.2472104	0.3117769	SLC26A10
cg07285276	0.7679853	348.5116	0.2459064	0.5220789	RAPGEF1
cg02490034	0.6414741	348.5116	0.2442989	0.3971752	MEST
cg12177001	0.7625607	348.5116	0.2418223	0.5207384	IFI27
cg18392482	0.7868274	348.5116	0.2414594	0.545368	AMDHD1
cg24012708	0.6627925	348.5116	0.2405701	0.4222224	HDHD3
cg26668713	0.387832	348.5116	0.2402968	0.1475352	SIPA1
cg13906813	0.6392214	348.5116	0.2400314	0.3991899	HLA-DPA1
cg17790333	0.730447	348.5116	0.2378185	0.4926285	CYP11A1
cg10861599	0.5196396	348.5116	0.2376329	0.2820067	TNFSF4
cg17686260	0.7338976	348.5116	0.2349083	0.4989893	MGMT
cg07236190	0.7202386	348.5116	0.2346325	0.4856061	AMDHD1
cg17890764	0.6377015	348.5116	0.2318467	0.4058547	ITIH4
cg13547644	0.4053276	348.5116	0.230592	0.1747356	ACTA1
cg15926585	0.7453433	348.5116	0.2304858	0.5148575	COMT
cg02674804	0.8598175	348.5116	0.2302686	0.6295489	REEP6
cg24459563	0.7111396	348.5116	0.2270972	0.4840423	CACNG1
cg12564453	0.7585761	348.5116	0.2249268	0.5336493	CETP
cg22759185	0.7784568	348.5116	0.2192051	0.5592517	REEP6
cg26394940	0.3375127	348.5116	0.2191917	0.118321	FLJ10945
cg16616769	0.7583253	348.5116	0.2183291	0.5399963	MGC35048
cg14444710	0.8050643	348.5116	0.2175655	0.5874987	PDPK1
cg12866859	0.5797057	348.5116	0.2173734	0.3623323	HEXIM1
cg13351161	0.4509546	348.5116	0.2113708	0.2395838	SCARA3
cg17105014	0.3892421	348.5116	0.210958	0.1782841	GYPC
cg22601917	0.464293	348.5116	0.2099738	0.2543193	H6PD
cg07284558	0.3458493	348.5116	0.2093686	0.1364807	STK32C
cg17296078	0.326533	348.5116	0.2079994	0.1185335	UBTD1
cg11397854	0.344146	348.5116	0.2075279	0.1366182	IQSEC1

Supplementary table 6 – Gene Ontology enrichment analysis of genes differentially methylated between Cluster A and normal tissue

GO biological process complete	Homo sapiens - REFLIST (20972)	upload_1 (1133)	upload_1 (expected)	upload_1 (over/under)	upload_1 (fold Enrichment)	upload_1 (P-value)
epoxygenase P450 pathway (GO:0019373)	18	9	0.97	+	9.26	7.13E-03
peptide cross-linking (GO:0018149)	56	26	3.03	+	8.59	2.79E-12
keratinization (GO:0031424)	50	23	2.7	+	8.51	1.69E-10
drug metabolic process (GO:0017144)	40	13	2.16	+	6.02	3.80E-03
defense response to fungus (GO:0050832)	37	12	2	+	6	1.06E-02
acute-phase response (GO:0006953)	45	14	2.43	+	5.76	2.33E-03
monocyte chemotaxis (GO:0002548)	40	12	2.16	+	5.55	2.34E-02
keratinocyte differentiation (GO:0030216)	101	30	5.46	+	5.5	1.57E-09
acute inflammatory response (GO:0002526)	76	22	4.11	+	5.36	3.96E-06
defense response to Gram-positive bacterium (GO:0050830)	66	18	3.57	+	5.05	3.52E-04
xenobiotic metabolic process (GO:0006805)	97	23	5.24	+	4.39	6.54E-05
cellular response to xenobiotic stimulus (GO:0071466)	101	23	5.46	+	4.22	1.35E-04
epidermal cell differentiation (GO:0009913)	145	32	7.83	+	4.08	5.04E-07
chemokine-mediated signaling pathway (GO:0070098)	73	16	3.94	+	4.06	3.15E-02
response to xenobiotic stimulus (GO:0009410)	110	23	5.94	+	3.87	6.13E-04
defense response to bacterium (GO:0042742)	229	45	12.37	+	3.64	3.55E-09
epidermis development (GO:0008544)	257	42	13.88	+	3.03	5.44E-06
skin development (GO:0043588)	212	34	11.45	+	2.97	3.49E-04

negative regulation of peptidase activity (GO:0010466)	250	40	13.51	+	2.96	2.47E-05
negative regulation of endopeptidase activity (GO:0010951)	239	37	12.91	+	2.87	2.18E-04
humoral immune response (GO:0006959)	170	26	9.18	+	2.83	3.02E-02
positive regulation of ERK1 and ERK2 cascade (GO:0070374)	177	27	9.56	+	2.82	2.08E-02
defense response to other organism (GO:0098542)	491	68	26.53	+	2.56	4.75E-08
leukocyte migration (GO:0050900)	265	36	14.32	+	2.51	7.10E-03
negative regulation of proteolysis (GO:0045861)	316	42	17.07	+	2.46	1.59E-03
defense response (GO:0006952)	1236	164	66.77	+	2.46	7.52E-22
response to bacterium (GO:0009617)	521	68	28.15	+	2.42	5.83E-07
extracellular matrix organization (GO:0030198)	309	40	16.69	+	2.4	5.88E-03
extracellular structure organization (GO:0043062)	310	40	16.75	+	2.39	6.36E-03
regulation of inflammatory response (GO:0050727)	301	38	16.26	+	2.34	1.96E-02
regulation of lipid metabolic process (GO:0019216)	287	36	15.51	+	2.32	4.11E-02
immune response (GO:0006955)	1105	138	59.7	+	2.31	1.59E-15
innate immune response (GO:0045087)	618	76	33.39	+	2.28	6.47E-07
inflammatory response (GO:0006954)	470	56	25.39	+	2.21	5.79E-04
negative regulation of hydrolase activity (GO:0051346)	405	48	21.88	+	2.19	5.70E-03
regulation of peptidase activity (GO:0052547)	399	47	21.56	+	2.18	8.75E-03
response to other organism (GO:0051707)	844	94	45.6	+	2.06	6.59E-07
response to external biotic stimulus (GO:0043207)	844	94	45.6	+	2.06	6.59E-07
response to biotic stimulus (GO:0009607)	876	95	47.33	+	2.01	2.04E-06
regulation of response to external stimulus (GO:0032101)	797	81	43.06	+	1.88	6.82E-04
regulation of immune response	862	87	46.57	+	1.87	3.00E-

(GO:0050776)						04
immune system process (GO:0002376)	2013	198	108.75	+	1.82	1.58E-12
response to external stimulus (GO:0009605)	1835	177	99.13	+	1.79	4.86E-10
cellular homeostasis (GO:0019725)	719	69	38.84	+	1.78	4.28E-02
chemical homeostasis (GO:0048878)	907	87	49	+	1.78	2.55E-03
regulation of immune system process (GO:0002682)	1397	134	75.47	+	1.78	1.23E-06
ion transmembrane transport (GO:0034220)	787	74	42.52	+	1.74	3.93E-02
negative regulation of catalytic activity (GO:0043086)	843	79	45.54	+	1.73	2.12E-02
response to nitrogen compound (GO:1901698)	881	82	47.6	+	1.72	1.76E-02
response to organic cyclic compound (GO:0014070)	902	83	48.73	+	1.7	2.33E-02
negative regulation of molecular function (GO:0044092)	1094	98	59.1	+	1.66	9.13E-03
homeostatic process (GO:0042592)	1374	121	74.23	+	1.63	1.11E-03
transmembrane transport (GO:0055085)	1131	99	61.1	+	1.62	2.13E-02
cellular response to chemical stimulus (GO:0070887)	2310	200	124.8	+	1.6	1.64E-07
response to oxygen-containing compound (GO:1901700)	1418	122	76.61	+	1.59	3.22E-03
cell surface receptor signaling pathway (GO:0007166)	2158	184	116.58	+	1.58	4.16E-06
tissue development (GO:0009888)	1513	126	81.74	+	1.54	1.06E-02
response to organic substance (GO:0010033)	2584	209	139.6	+	1.5	1.74E-05
response to stress (GO:0006950)	3301	266	178.33	+	1.49	6.86E-08
cellular response to organic substance (GO:0071310)	1887	150	101.94	+	1.47	1.29E-02
regulation of biological quality (GO:0065008)	3460	273	186.92	+	1.46	3.08E-07
system process (GO:0003008)	1804	142	97.46	+	1.46	4.19E-02
animal organ development (GO:0048513)	2811	220	151.86	+	1.45	8.90E-05
response to chemical	3866	299	208.86	+	1.43	2.23E-

(GO:0042221)						07
regulation of response to stimulus (GO:0048583)	3640	281	196.65	+	1.43	1.42E-06
response to stimulus (GO:0050896)	7565	580	408.69	+	1.42	1.78E-21
regulation of localization (GO:0032879)	2447	187	132.2	+	1.41	7.09E-03
multi-organism process (GO:0051704)	2251	170	121.61	+	1.4	4.83E-02
regulation of catalytic activity (GO:0050790)	2352	177	127.07	+	1.39	3.69E-02
single-multicellular organism process (GO:0044707)	5301	398	286.38	+	1.39	9.24E-10
system development (GO:0048731)	3938	291	212.75	+	1.37	5.33E-05
multicellular organismal process (GO:0032501)	6356	466	343.38	+	1.36	6.30E-11
signal transduction (GO:0007165)	4765	349	257.43	+	1.36	1.85E-06
regulation of molecular function (GO:0065009)	2843	207	153.59	+	1.35	4.25E-02
cell differentiation (GO:0030154)	3210	233	173.42	+	1.34	1.13E-02
single organism signaling (GO:0044700)	5107	368	275.9	+	1.33	3.30E-06
signaling (GO:0023052)	5110	368	276.06	+	1.33	3.55E-06
cell communication (GO:0007154)	5197	371	280.76	+	1.32	8.48E-06
multicellular organism development (GO:0007275)	4532	323	244.84	+	1.32	2.35E-04
cellular response to stimulus (GO:0051716)	6129	436	331.12	+	1.32	1.22E-07
anatomical structure development (GO:0048856)	4966	350	268.29	+	1.3	1.46E-04
developmental process (GO:0032502)	5297	366	286.17	+	1.28	5.12E-04
negative regulation of biological process (GO:0048519)	4490	310	242.57	+	1.28	1.01E-02
single-organism developmental process (GO:0044767)	5210	355	281.47	+	1.26	3.93E-03
single-organism cellular process (GO:0044763)	10980	734	593.19	+	1.24	1.50E-13
single-organism process (GO:0044699)	12314	820	665.26	+	1.23	6.34E-18
regulation of biological process	10826	695	584.87	+	1.19	2.32E-

(GO:0050789)						07
biological regulation (GO:0065007)	11443	734	618.2	+	1.19	1.52E-08
regulation of cellular process (GO:0050794)	10301	634	556.51	+	1.14	1.91E-02
biological_process (GO:0008150)	16903	1022	913.17	+	1.12	5.44E-15
macromolecule metabolic process (GO:0043170)	7412	326	400.43	-	0.81	1.26E-02
regulation of cellular biosynthetic process (GO:0031326)	4246	169	229.39	-	0.74	1.85E-02
cellular macromolecule metabolic process (GO:0044260)	6729	265	363.53	-	0.73	5.81E-07
regulation of transcription, DNA-templated (GO:0006355)	3565	138	192.6	-	0.72	3.45E-02
regulation of RNA biosynthetic process (GO:2001141)	3606	139	194.81	-	0.71	2.36E-02
regulation of nucleic acid-templated transcription (GO:1903506)	3589	138	193.89	-	0.71	2.17E-02
regulation of nucleobase-containing compound metabolic process (GO:0019219)	4054	155	219.01	-	0.71	2.67E-03
nitrogen compound metabolic process (GO:0006807)	5609	214	303.02	-	0.71	3.07E-06
regulation of macromolecule biosynthetic process (GO:0010556)	4065	155	219.61	-	0.71	2.14E-03
regulation of RNA metabolic process (GO:0051252)	3717	139	200.81	-	0.69	2.54E-03
organic cyclic compound metabolic process (GO:1901360)	4882	181	263.75	-	0.69	6.92E-06
regulation of nitrogen compound metabolic process (GO:0051171)	4349	161	234.95	-	0.69	7.80E-05
regulation of cellular macromolecule biosynthetic process (GO:2000112)	3949	144	213.34	-	0.67	1.74E-04
organic substance biosynthetic process (GO:1901576)	4681	168	252.89	-	0.66	1.21E-06
biosynthetic process (GO:0009058)	4768	171	257.59	-	0.66	7.02E-07
organelle organization (GO:0006996)	3210	112	173.42	-	0.65	4.59E-04
heterocycle metabolic process (GO:0046483)	4646	161	251	-	0.64	7.03E-08
cellular biosynthetic process	4579	158	247.38	-	0.64	7.36E-

(GO:0044249)						08
cellular aromatic compound metabolic process (GO:0006725)	4695	161	253.64	-	0.63	2.01E-08
cellular nitrogen compound metabolic process (GO:0034641)	5131	175	277.2	-	0.63	6.37E-10
transcription, DNA-templated (GO:0006351)	2560	87	138.3	-	0.63	4.10E-03
nucleic acid-templated transcription (GO:0097659)	2561	87	138.36	-	0.63	4.00E-03
organic cyclic compound biosynthetic process (GO:1901362)	3198	107	172.77	-	0.62	4.28E-05
RNA biosynthetic process (GO:0032774)	2684	89	145	-	0.61	6.18E-04
aromatic compound biosynthetic process (GO:0019438)	3090	101	166.94	-	0.61	2.16E-05
cellular macromolecule biosynthetic process (GO:0034645)	3633	118	196.27	-	0.6	3.51E-07
macromolecule biosynthetic process (GO:0009059)	3714	120	200.65	-	0.6	1.35E-07
heterocycle biosynthetic process (GO:0018130)	3078	99	166.29	-	0.6	9.12E-06
nucleobase-containing compound biosynthetic process (GO:0034654)	3020	97	163.15	-	0.59	1.26E-05
nucleobase-containing compound metabolic process (GO:0006139)	4509	141	243.6	-	0.58	1.69E-11
cellular nitrogen compound biosynthetic process (GO:0044271)	3508	109	189.52	-	0.58	4.00E-08
gene expression (GO:0010467)	3770	112	203.67	-	0.55	1.51E-10
RNA metabolic process (GO:0016070)	3446	102	186.17	-	0.55	2.23E-09
nucleic acid metabolic process (GO:0090304)	3961	112	213.99	-	0.52	4.06E-13
Unclassified (UNCLASSIFIED)	4069	111	219.83	-	0.5	0.00E+00
chromosome organization (GO:0051276)	992	21	53.59	-	0.39	1.71E-03
DNA metabolic process (GO:0006259)	777	15	41.98	-	0.36	8.86E-03
cellular macromolecular complex assembly (GO:0034622)	727	14	39.28	-	0.36	1.93E-02

modification-dependent macromolecule catabolic process (GO:0043632)	495	7	26.74	-	0.26	4.29E-02
mRNA metabolic process (GO:0016071)	621	8	33.55	-	0.24	8.42E-04
RNA processing (GO:0006396)	860	10	46.46	-	0.22	4.81E-07
mRNA processing (GO:0006397)	439	5	23.72	-	0.21	2.73E-02
ncRNA processing (GO:0034470)	404	4	21.83	-	< 0.2	2.68E-02
ncRNA metabolic process (GO:0034660)	554	5	29.93	-	< 0.2	1.47E-04
RNA splicing (GO:0008380)	374	3	20.21	-	< 0.2	1.92E-02
ribonucleoprotein complex biogenesis (GO:0022613)	459	3	24.8	-	< 0.2	3.22E-04

Supplementary table 7 – probes differentially methylated between benign tumours in cluster A and normal tissue

TargetID	A_benign.AVG_Beta	A_benign.DiffScore	A_benign.DeltaBeta	normal.AVG_Beta	SYMBOL
cg16363586	0.7318995	350.9073	0.42029	0.3116095	BST2
cg01346152	0.6477849	350.9073	0.396808	0.2509769	DHRS3
cg14409083	0.6325329	350.9073	0.3939567	0.2385762	EMP1
cg09871315	0.6886036	350.9073	0.3832297	0.305374	HOXA2
cg03852144	0.646186	350.9073	0.3799342	0.2662518	GLRX
cg24101578	0.7117751	350.9073	0.3720612	0.3397138	CDH22
cg01126560	0.7422166	350.9073	0.3444153	0.3978014	C9orf142
cg04106785	0.8219019	350.9073	0.3358079	0.4860941	CDK5R1
cg18702197	0.6998484	350.9073	0.3336436	0.3662047	HOXD3
cg02506908	0.7539712	350.9073	0.3314823	0.4224889	HPD
cg25101936	0.8154942	350.9073	0.314916	0.5005782	ZBTB16
cg17890764	0.7153561	350.9073	0.3095013	0.4058547	ITIH4
cg22628873	0.7565897	350.9073	0.3037612	0.4528286	GGT6
cg26509022	0.505851	350.9073	0.3024757	0.2033753	ALDH1A3
cg15958424	0.5678796	350.9073	0.2993689	0.2685107	ACPP
cg03605761	0.6559187	350.9073	0.2988177	0.357101	RNF126
cg17105014	0.4721282	350.9073	0.2938441	0.1782841	GYPC
cg19224278	0.6052728	350.9073	0.2904042	0.3148687	ALDH1A3
cg01103836	0.9608721	350.9073	0.2859139	0.6749582	MYO9B
cg08624249	0.7633548	350.9073	0.2843247	0.4790302	KIAA0889
cg16517394	0.4855182	350.9073	0.2807874	0.2047307	TNFSF4
cg07251788	0.7675034	350.9073	0.2801602	0.4873433	CLTCL1
cg06204948	0.5060629	350.9073	0.2797478	0.226315	MARK2

cg13763232	0.7614101	350.9073	0.2783912	0.4830189	SLC6A6
cg10861599	0.5578045	350.9073	0.2757978	0.2820067	TNFSF4
cg18172186	0.7959149	350.9073	0.2754223	0.5204926	KIAA1913
cg15926585	0.7899944	350.9073	0.2751369	0.5148575	COMT
cg24012708	0.6962115	350.9073	0.2739891	0.4222224	HDHD3
cg12564453	0.807474	350.9073	0.2738247	0.5336493	CETP
cg16616769	0.8137823	350.9073	0.2737861	0.5399963	MGC3504 8
cg03271907	0.8582872	350.9073	0.2696304	0.5886568	MGMT
cg26668713	0.4164093	350.9073	0.2688741	0.1475352	SIPA1
cg17791651	0.5639657	350.9073	0.2661661	0.2977996	POU3F1
cg14371590	0.5779256	350.9073	0.2661486	0.3117769	SLC26A10
cg12866859	0.6282226	350.9073	0.2658903	0.3623323	HEXIM1
cg24427660	0.7715482	350.9073	0.2649799	0.5065683	PNPLA2
cg13547644	0.437705	350.9073	0.2629693	0.1747356	ACTA1
cg24459563	0.7468368	350.9073	0.2627944	0.4840423	CACNG1
cg02674804	0.8918444	350.9073	0.2622955	0.6295489	REEP6
cg18392482	0.8071101	350.9073	0.2617421	0.545368	AMDHD1
cg04700814	0.5555636	350.9073	0.2614623	0.2941013	HEXIM1
cg02490034	0.6575207	350.9073	0.2603455	0.3971752	MEST
cg06507244	0.7057386	350.9073	0.2592581	0.4464805	DHX32
cg12177001	0.7788891	350.9073	0.2581507	0.5207384	IFI27
cg07285276	0.7785299	350.9073	0.256451	0.5220789	RAPGEF1
cg05342835	0.6273702	350.9073	0.2549253	0.3724449	SYNC1
cg00431549	0.5858328	350.9073	0.2523405	0.3334922	MGP
cg06627364	0.433069	350.9073	0.2499924	0.1830765	MGC4677
cg11397854	0.3865781	350.9073	0.2499599	0.1366182	IQSEC1
cg17790333	0.7421055	350.9073	0.2494771	0.4926285	CYP11A1
cg17686260	0.748266	350.9073	0.2492768	0.4989893	MGMT
cg07236190	0.7344995	350.9073	0.2488934	0.4856061	AMDHD1
cg22601917	0.502579	350.9073	0.2482598	0.2543193	H6PD
cg23910243	0.7139539	350.9073	0.2475247	0.4664292	TGFB1I1
cg26394940	0.3649756	350.9073	0.2466546	0.118321	FLJ10945
cg23547429	0.4816236	350.9073	0.2447044	0.2369192	SLC43A3
cg13351161	0.4806728	350.9073	0.241089	0.2395838	SCARA3
cg09835085	0.5777772	350.9073	0.241033	0.3367442	KCNE4
cg15940569	0.5055822	350.9073	0.2405791	0.2650031	GABRB3
cg22759185	0.7991371	350.9073	0.2398854	0.5592517	REEP6
cg11554605	0.7575803	350.9073	0.2397411	0.5178393	ASB4
cg04640913	0.6344089	350.9073	0.2396104	0.3947985	CDH22
cg17518962	0.4631739	350.9073	0.2382795	0.2248944	GAL3ST4
cg13906813	0.6370082	350.9073	0.2378183	0.3991899	HLA-DPA1
cg14444710	0.8219597	350.9073	0.234461	0.5874987	PDPK1
cg07047653	0.7924626	350.9073	0.2342829	0.5581798	AQP2
cg26511075	0.8276694	350.9073	0.2342501	0.5934194	FLJ25422

cg24134767	0.6634437	350.9073	0.2328859	0.4305578	HTR3A
cg06456031	0.438114	350.9073	0.2316598	0.2064543	FLJ11000
cg07284558	0.3648087	350.9073	0.228328	0.1364807	STK32C
cg16077929	0.7314463	350.9073	0.2275407	0.5039055	CDKL1
cg21685427	0.7724158	350.9073	0.2254099	0.5470058	SGK2
cg13587552	0.7644741	142.947	0.2197931	0.544681	SCNN1D
cg03562120	0.5733943	140.0818	0.2190737	0.3543206	WISP2
cg05485060	0.6308289	134.2784	0.2189342	0.4118947	CTNNAL1
cg15236866	0.4824858	350.9073	0.2184439	0.2640419	DLX1
cg12232463	0.648662	132.5832	0.2181041	0.4305579	LONRF2
cg17339202	0.4940087	350.9073	0.2159404	0.2780684	SYNC1
cg27114026	0.7448972	133.339	0.2140984	0.5307989	ELA1
cg17296078	0.3314849	350.9073	0.2129513	0.1185335	UBTD1
cg14611112	0.6013207	127.7779	0.2126407	0.38868	LCN6
cg03096975	0.3847917	350.9073	0.2112888	0.1735029	EML2
cg22467567	0.4894396	142.947	0.2110077	0.2784319	IGFBP5
cg10055471	0.9262407	350.9073	0.2098029	0.7164378	NR0B2
cg24315815	0.7340075	126.9884	0.2097711	0.5242364	PLSCR4
cg03359508	0.3371783	350.9073	0.2077055	0.1294728	ANXA11
cg02085507	0.4674757	142.947	0.2069165	0.2605592	TRIP10
cg21747271	0.6526744	117.8083	0.2060854	0.446589	AIP
cg05253327	0.7564764	125.313	0.205433	0.5510435	B3GNT1
cg08493463	0.653909	116.786	0.205217	0.448692	ENPP6
cg04802221	0.6224962	115.7478	0.2039992	0.418497	LOC283849
cg26069745	0.515412	125.7503	0.2025176	0.3128945	HOXA2
cg17998964	0.3660597	350.9073	0.2022685	0.1637913	MARK2
cg04498511	0.8033556	130.8741	0.2018071	0.6015485	ZC3H11A
cg09595479	0.3812983	350.9073	0.2008341	0.1804643	PRPH
cg04902405	0.6821129	111.9026	0.2001831	0.4819297	ZC3H11A
cg07572435	0.7740341	122.4825	0.200073	0.5739611	LY6D
cg18356799	0.3248164	-110.8892	-0.2000218	0.5248383	DSC1
cg20423977	0.5221806	-145.6849	-0.2000577	0.7222383	PLAC4
cg09995854	0.471266	-129.1504	-0.2000875	0.6713535	IL1F8
cg15779716	0.3466449	-111.6417	-0.2000905	0.5467355	CDCP1
cg15822411	0.4983643	-137.3375	-0.2002677	0.6986321	MGC34732
cg06415153	0.3515844	-112.2031	-0.2003536	0.5519381	PITPNM2
cg14620221	0.5455269	-156.9226	-0.2004165	0.7459434	OR8B8
cg06495803	0.3175568	-111.2913	-0.2004517	0.5180085	TNFRSF7
cg04991214	0.3884616	-115.2014	-0.2004843	0.5889459	PFDN2
cg05040360	0.4023282	-116.9318	-0.2005837	0.6029119	MS4A6E
cg04720330	0.09568101	-148.5375	-0.2007309	0.296412	PHLDA2
cg08191915	0.225937	-116.4886	-0.2008085	0.4267455	PANX3
cg01346718	0.1975097	-120.5512	-0.2008562	0.3983659	CSNK1E

cg22825487	0.4943737	-137.0931	-0.2008941	0.6952678	VNN3
cg14321743	0.4461523	-124.7908	-0.2010542	0.6472065	PLA2G2D
cg26757722	0.2648683	-113.3994	-0.201103	0.4659714	CACNG2
cg08711674	0.3003897	-112.459	-0.2014217	0.5018114	AKT1S1
cg13726463	0.5365358	-154.7618	-0.2015324	0.7380682	COX6A2
cg03014957	0.3651012	-114.4624	-0.2015337	0.5666349	DEFB118
cg23216015	0.1789561	-124.6484	-0.201557	0.3805131	C7orf16
cg23824713	0.5273647	-151.0276	-0.2017291	0.7290938	SERPINB5
cg19258973	0.4130538	-120.0178	-0.2017855	0.6148393	KRTHB3
cg10938286	0.6430569	-242.4591	-0.2018281	0.844885	CST2
cg12445208	0.3525514	-114.0172	-0.2018304	0.5543818	ZNF583
cg26822241	0.4959837	-139.4404	-0.2019576	0.6979414	CYP2C9
cg22937804	0.33636	-113.501	-0.2020168	0.5383769	MGC44505
cg20543571	0.6388376	-237.8687	-0.20207	0.8409076	C15orf43
cg11505080	0.2037609	-120.9703	-0.2021421	0.405903	GPR173
cg19096475	0.4194538	-121.7182	-0.202318	0.6217718	ASAM
cg04713352	0.3738753	-116.1589	-0.2023452	0.5762205	ATP4A
cg01359534	0.5517934	-164.2864	-0.2023519	0.7541453	AQP10
cg26391080	0.521719	-149.8381	-0.2023836	0.7241026	SH2D4B
cg19478478	0.4811571	-135.5152	-0.20239	0.6835471	TRDN
cg08390254	0.2807952	-114.0433	-0.2023915	0.4831868	ATP1A2
cg09715672	0.2641212	-114.9502	-0.2024781	0.4665993	C10orf116
cg12040555	0.6900006	-328.7926	-0.2025043	0.8925049	MGMT
cg18221897	0.4917175	-139.0512	-0.2025582	0.6942758	KIR2DL1
cg20305726	0.3383783	-114.5754	-0.2028581	0.5412364	DEFB126
cg14898892	0.3370276	-114.539	-0.2028671	0.5398947	SHRM
cg15916061	0.3467495	-115.0813	-0.202976	0.5497255	SLC17A4
cg15585987	0.2580275	-116.0694	-0.2031235	0.461151	SNTG1
cg12188416	0.29672	-114.456	-0.2031363	0.4998563	TP73L
cg06747888	0.1935352	-123.7731	-0.2032001	0.3967353	TBR1
cg03440846	0.2137814	-120.7376	-0.2032454	0.4170268	ACSS2
cg12616487	0.4810311	-136.9558	-0.2032928	0.684324	EML3
cg10431340	0.3294729	-114.8056	-0.2033034	0.5327762	MPZ
cg16739580	0.4294218	-124.9851	-0.2034464	0.6328683	POP2
cg15746187	0.4477564	-128.6656	-0.2034594	0.6512157	FBXO44
cg00476577	0.1134818	-144.8521	-0.2035769	0.3170587	ZNF217
cg00364814	0.4532914	-130.1745	-0.2036331	0.6569245	R3HDM2
cg24147596	0.4499107	-129.5975	-0.2037636	0.6536743	ARL14
cg06946880	0.5106438	-148.0482	-0.2038462	0.7144901	ATP6V1B1
cg20832020	0.5041015	-145.5876	-0.2038678	0.7079693	VSIG9
cg11241627	0.09744513	-151.476	-0.2039041	0.3013493	FERD3L
cg20769842	0.3380242	-115.8398	-0.203931	0.5419552	MKRN3
cg00499822	0.2705599	-116.3307	-0.2040223	0.4745822	CXCL12
cg12332316	0.6366496	-241.9673	-0.2040702	0.8407198	F12

cg00463848	0.5184652	-151.657	-0.2040753	0.7225405	KRT2A
cg20904010	0.4917455	-142.0883	-0.2043427	0.6960882	SYN3
cg17977362	0.553925	-169.8673	-0.204374	0.7582989	FLJ45964
cg25598083	0.3860212	-120.0657	-0.2044413	0.5904626	ACOT2
cg17040807	0.1724303	-129.2211	-0.2044705	0.3769008	CYGB
cg14672680	0.6798923	-316.4981	-0.2045506	0.8844429	TNNI2
cg01274324	0.6376901	-245.2935	-0.2046285	0.8423186	C21orf77
cg10726357	0.3663226	-118.4381	-0.2046499	0.5709725	TSCOT
cg14401454	0.6324998	-238.908	-0.2047051	0.8372048	C14orf8
cg01598642	0.5166456	-152.2852	-0.204833	0.7214786	HBG1
cg07072643	0.3556211	-117.9402	-0.204879	0.5605	EMR3
cg15446391	0.204311	-124.0334	-0.2049993	0.4093103	WT1
cg27420123	0.5090091	-149.5896	-0.2050354	0.7140445	FSHB
cg10746737	0.500123	-146.2639	-0.2050599	0.7051829	HLA-DRB5
cg15329483	0.391953	-121.6641	-0.2051368	0.5970898	SSX7
cg02311163	0.4108716	-124.243	-0.2051378	0.6160095	SEMG2
cg05248781	0.6361415	-245.062	-0.2051397	0.8412812	LCE5A
cg21624282	0.5929345	-199.6467	-0.2053021	0.7982366	LOC12225 8
cg16219122	0.2767739	-117.5249	-0.2053211	0.482095	ABCB1
cg15792367	0.3163358	-116.9807	-0.2053535	0.5216893	KLK11
cg20790540	0.4518295	-132.5551	-0.2054199	0.6572493	PTCRA
cg07914866	0.1321058	-140.7331	-0.2054397	0.3375455	IRAK3
cg13650156	0.4936715	-144.9825	-0.2056342	0.6993057	PILRA
cg16899306	0.4160388	-125.8406	-0.205696	0.6217347	HLA-DQB2
cg09382492	0.509633	-151.2859	-0.2058172	0.7154502	AANAT
cg02633817	0.4794129	-140.9685	-0.2060135	0.6854264	FXYD3
cg21023001	0.2995441	-117.8035	-0.2060603	0.5056044	C1QR1
cg04623837	0.09533664	-154.8626	-0.2060896	0.3014262	HCG9
cg03309967	0.4102314	-125.4964	-0.2061101	0.6163416	PSENEN
cg27655855	0.4972784	-147.1358	-0.2061316	0.70341	CST9L
cg15140807	0.5355108	-163.6267	-0.2061357	0.7416465	FLJ31222
cg04761824	0.4898244	-144.6527	-0.2062021	0.6960264	30-nov
cg01109219	0.3261268	-118.2361	-0.2062386	0.5323654	RASGRP3
cg21624359	0.2717448	-118.8507	-0.2062716	0.4780163	FFAR3
cg11656547	0.1070737	-150.3813	-0.2062816	0.3133553	MAMDC2
cg22607339	0.5079649	-151.6838	-0.2063939	0.7143588	MPL
cg26159905	0.3700302	-121.0263	-0.2064155	0.5764457	ASB10
cg26134665	0.3789326	-122.006	-0.2065235	0.5854561	STX1B2
cg25993152	0.4970154	-147.7721	-0.2065388	0.7035542	XAGE5
cg26185508	0.4734458	-140.1851	-0.2066198	0.6800656	CDCP2
cg19875656	0.5851111	-196.8409	-0.2066734	0.7917845	TSP50
cg25545210	0.4802414	-142.5378	-0.2067898	0.6870312	KRTHA4
cg01407244	0.1975558	-127.1324	-0.2068196	0.4043754	TNFRSF10 C

cg14127336	0.6631874	-294.0492	-0.2068793	0.8700668	TCL1A
cg26222045	0.3843838	-123.1545	-0.2069333	0.5913171	UNQ5810
cg05113558	0.5780452	-192.0948	-0.2069672	0.7850124	FOXH1
cg07173760	0.3042673	-119.1234	-0.2071994	0.5114667	CLC
cg10710439	0.2374931	-122.3807	-0.2072037	0.4446968	FLJ37549
cg15447486	0.4947377	-148.3136	-0.2073037	0.7020414	GPR109B
cg24862483	0.09240032	-157.6244	-0.2073298	0.2997301	CD300LG
cg13482233	0.501277	-150.8364	-0.2073522	0.7086292	HEPH
cg02097420	0.4479751	-134.7449	-0.2074346	0.6554097	HRG
cg03329572	0.4831486	-144.632	-0.207476	0.6906246	FCRL5
cg13745346	0.4689928	-140.3103	-0.207491	0.6764838	CBFA2T3
cg07220939	0.4171354	-128.7402	-0.2076177	0.6247531	SLC22A12
cg03112433	0.248916	-121.8903	-0.2076595	0.4565755	PFTK1
cg09626634	0.3314871	-120.2309	-0.2077249	0.539212	EBI2
cg14102807	0.488095	-146.9102	-0.2078373	0.6959323	CD19
cg13678049	0.2077195	-126.71	-0.2078492	0.4155688	PARC
cg10146929	0.1558321	-137.0231	-0.2079849	0.363817	HIST1H1A
cg19242268	0.3337625	-120.6527	-0.2079935	0.541756	TCEA2
cg11693019	0.6146575	-229.8722	-0.2083415	0.8229991	AKR1B10
cg07892051	0.4341426	-133.148	-0.2084143	0.6425568	AKAP3
cg02537838	0.5894345	-205.1722	-0.2084194	0.7978539	C20orf151
cg25677709	0.5330164	-167.0906	-0.2084207	0.7414371	NDST1
cg16792160	0.3504857	-122.0893	-0.2084598	0.5589455	ASAH2
cg01193293	0.3100429	-120.7511	-0.2085415	0.5185844	SIGLEC7
cg04719766	0.575326	-194.1925	-0.208603	0.7839289	KCNQ1
cg07823492	0.6416223	-265.6131	-0.2086276	0.8502499	HOXB1
cg13530946	0.3879893	-125.8789	-0.2086614	0.5966507	IARS2
cg20017147	0.301639	-120.8961	-0.2086729	0.5103118	TEX101
cg26745032	0.2455992	-123.3356	-0.2086813	0.4542806	REPS2
cg08804892	0.1440583	-141.0269	-0.2087607	0.352819	TRAK1
cg12937434	0.129964	-145.2287	-0.2087658	0.3387298	BZRAP1
cg13030582	0.1629079	-136.2462	-0.2087949	0.3717028	MFAP4
cg01762581	0.2188832	-126.4398	-0.2089413	0.4278245	DKK4
cg23324787	0.4082621	-129.1687	-0.2089539	0.617216	RAG2
cg17568996	0.272131	-122.0031	-0.2090184	0.4811494	NFAM1
cg02124291	0.5001586	-153.5775	-0.2091061	0.7092648	OR7A5
cg02765820	0.3675907	-124.3548	-0.2091753	0.576766	C3orf22
cg14178895	0.2440757	-124.0881	-0.2092274	0.4533031	C6orf105
cg11811840	0.4132608	-130.3706	-0.2092334	0.6224942	UGT1A1
cg03387723	0.5541958	-181.1377	-0.2092772	0.763473	SCMH1
cg12547930	0.4058277	-129.2424	-0.2092842	0.6151119	WFDC6
cg17894008	0.6096826	-227.5632	-0.2093384	0.819021	NACAL
cg13447818	0.5605526	-185.6161	-0.2094048	0.7699574	FLG
cg27214365	0.5477276	-177.4491	-0.2094128	0.7571404	GYPB
cg13407883	0.3807579	-126.0526	-0.2094293	0.5901871	SIGLEC9

cg07979357	0.08325797	-164.3348	-0.2094295	0.2926875	IL27RA
cg17803430	0.4566161	-140.2096	-0.2095297	0.6661457	C4BPA
cg26692016	0.438835	-135.9398	-0.2095789	0.6484139	APOBEC1
cg01072821	0.4187466	-131.9116	-0.20962	0.6283666	UNQ9391
cg11432797	0.4847695	-149.0682	-0.2097206	0.6944901	SPN
cg26777475	0.3881051	-127.3537	-0.2097415	0.5978466	PCOLCE
cg05254747	0.5297803	-168.7045	-0.2099832	0.7397634	SLC39A14
cg00501366	0.3789408	-126.6685	-0.2100468	0.5889876	ALOX12B
cg00344709	0.5653868	-190.5183	-0.2100521	0.7754389	ANKRD21
cg19138060	0.4083929	-130.7233	-0.2100553	0.6184482	WDR13
cg11328541	0.2041956	-129.9154	-0.2101937	0.4143894	OR7C1
cg16879596	0.5096647	-159.7903	-0.2102965	0.7199612	CYP3A5
cg08952029	0.2611113	-124.085	-0.2103017	0.471413	CHRDL2
cg17819635	0.1774774	-135.0537	-0.2105428	0.3880202	TCTEX1D1
cg14154330	0.2854715	-123.3613	-0.2105668	0.4960383	ARHGAP27
cg19587887	0.4841644	-150.4559	-0.2106158	0.6947802	PSKH2
cg01560871	0.6458548	-280.4762	-0.21073	0.8565848	C10orf27
cg14462830	0.3983284	-130.1693	-0.2107552	0.6090836	TRDN
cg12330929	0.5253197	-168.1408	-0.2108059	0.7361256	USP9X
cg11787522	0.32626	-124.2319	-0.2111261	0.5373861	STRA6
cg20277416	0.3780341	-128.0512	-0.2111554	0.5891895	TM7SF2
cg07426848	0.5012192	-157.8612	-0.2111564	0.7123755	S100A3
cg12089698	0.3099373	-123.9365	-0.2111803	0.5211176	SPATC1
cg22088368	0.3225059	-124.2046	-0.2111963	0.5337022	MGC35206
cg20189937	0.2822147	-124.2158	-0.2112122	0.4934269	L2HGDH
cg05190718	0.485115	-151.9169	-0.211262	0.696377	CASQ2
cg01135626	0.3735547	-127.8925	-0.2114015	0.5849562	CDX4
cg18807515	0.410477	-133.1059	-0.211446	0.621923	PRAMEF2
cg11237817	0.3932219	-130.5159	-0.2115461	0.6047679	KIR3DL3
cg19067730	0.08446328	-166.3454	-0.2115828	0.296046	PPGB
cg21312148	0.3189183	-124.5857	-0.2115845	0.5305029	LCE2D
cg09133026	0.5182403	-166.3128	-0.2116103	0.7298506	RPS6KL1
cg27383362	0.443532	-140.2423	-0.2116448	0.6551768	ATAD3C
cg24192663	0.5162734	-165.5499	-0.2117022	0.7279755	HSPA6
cg24642468	0.3065259	-124.6755	-0.2118193	0.5183452	MGC33367
cg06494770	0.3311934	-125.4916	-0.2119732	0.5431665	KLHL13
cg06074920	0.5657875	-196.2327	-0.2122312	0.7780187	KRTHA4
cg24489015	0.3824205	-130.2576	-0.2123851	0.5948056	LPO
cg23282949	0.443238	-141.4095	-0.2124276	0.6556656	RENBP
cg07339327	0.6914697	-350.9073	-0.2124642	0.9039338	CCND1
cg18750960	0.2840813	-125.7067	-0.2125005	0.4965817	HOXD4
cg15417244	0.5026455	-161.1121	-0.2125363	0.7151818	PDZRN4

cg14449575	0.2491046	-127.5278	-0.212547	0.4616516	HAPLN4
cg19554294	0.4003752	-133.2658	-0.2127222	0.6130974	VN1R2
cg11630242	0.1384494	-147.2588	-0.2127428	0.3511922	AKAP10
cg22456522	0.2251301	-130.242	-0.2129022	0.4380323	LILRB3
cg03993463	0.6299766	-265.1775	-0.2129799	0.8429564	KCNJ15
cg25645462	0.6392916	-279.1084	-0.2129903	0.8522819	GPR56
cg20781967	0.2233339	-130.6301	-0.2130677	0.4364015	NINJ2
cg06142324	0.4210437	-137.4712	-0.2130823	0.634126	FLJ25530
cg04057858	0.3919604	-132.5224	-0.2130853	0.6050456	UNQ9391
cg22772878	0.2483109	-128.3638	-0.2132087	0.4615197	DIRAS1
cg27168844	0.4210217	-137.7253	-0.2132559	0.6342776	IL17
cg12397274	0.2568856	-127.8839	-0.2133186	0.4702042	TINAG
cg08878744	0.3019855	-126.5396	-0.213352	0.5153375	LCE1B
cg18239253	0.3457292	-128.1005	-0.2134028	0.559132	DEFB32
cg15669228	0.373341	-130.7074	-0.2135097	0.5868507	IFNA8
cg09841009	0.5403412	-182.4693	-0.2135819	0.7539231	GYPA
cg23812886	0.3186032	-127.1848	-0.2136793	0.5322825	SSX5
cg02688643	0.5025941	-163.5222	-0.2137864	0.7163805	MGST2
cg23244913	0.153889	-144.2315	-0.2137977	0.3676866	HCG9
cg23075286	0.4112279	-136.8663	-0.2139214	0.6251493	GALP
cg00689340	0.353917	-129.7504	-0.2141831	0.5681001	RTKN
cg23110514	0.242092	-130.0684	-0.2142125	0.4563045	LCE3E
cg20692181	0.4011401	-135.6025	-0.214255	0.6153951	RETN
cg10479672	0.2168849	-133.1109	-0.2144896	0.4313744	IL1F8
cg13883681	0.2926494	-128.0197	-0.2145407	0.5071902	GOT2
cg13311440	0.3002273	-128.0023	-0.2145515	0.5147789	CD48
cg12067287	0.5419622	-185.7683	-0.2145916	0.7565538	MYOM1
cg07471052	0.6912712	-350.9073	-0.2146562	0.9059274	CDK3
cg00415993	0.236971	-131.1608	-0.2147586	0.4517296	F2RL2
cg03599338	0.3801426	-133.3675	-0.2148378	0.5949804	SUSD2
cg01185080	0.3349183	-129.4686	-0.2149505	0.5498688	ZNF710
cg20357806	0.4891322	-160.2303	-0.2149719	0.7041041	PPBP
cg09283007	0.6893535	-350.9073	-0.2150124	0.9043659	FAM47B
cg08453096	0.6108466	-247.3811	-0.2150458	0.8258924	ABCG5
cg18565355	0.2454918	-130.8841	-0.2151626	0.4606544	RBM35A
cg16142218	0.2581041	-130.0146	-0.2151653	0.4732695	CHMP7
cg20437604	0.4693387	-153.4933	-0.2152104	0.6845492	ANXA9
cg00400028	0.1572538	-145.1556	-0.2153033	0.3725572	ACPL2
cg21296676	0.3152098	-129.2044	-0.2153476	0.5305574	EYA4
cg15778232	0.3121729	-129.2758	-0.215463	0.5276359	PHB2
cg00443307	0.3707239	-133.2959	-0.2156	0.586324	KLRG1
cg09061733	0.3675256	-133.1059	-0.2157111	0.5832367	SERPING1
cg09863066	0.2141171	-134.9926	-0.2157774	0.4298946	PVALB
cg14519000	0.6609473	-331.0442	-0.2157993	0.8767466	GATA5
cg14722162	0.4359692	-145.0562	-0.2158352	0.6518043	C5orf20

cg22927134	0.3244067	-130.2336	-0.2159289	0.5403357	CHRM5
cg10078829	0.2530355	-131.3308	-0.2160018	0.4690373	KLK4
cg06980053	0.2738036	-130.3505	-0.2160881	0.4898917	RASSF1
cg22879289	0.3186688	-130.277	-0.216127	0.5347958	NID1
cg09837648	0.6503305	-312.0899	-0.2162771	0.8666076	PLXNB1
cg10300154	0.4093744	-140.0818	-0.2162983	0.6256728	MGC5297
cg21274025	0.281818	-130.4451	-0.2163735	0.4981915	PLA2G3
cg09584711	0.2372671	-133.1312	-0.2164255	0.4536925	HPR
cg11873854	0.1787685	-141.6731	-0.216456	0.3952245	LCN6
cg26918728	0.6171473	-260.3022	-0.2164679	0.8336151	SEMA3B
cg11830061	0.6992174	-350.9073	-0.2165012	0.9157187	INSL6
cg00601486	0.6761953	-350.9073	-0.2165405	0.8927357	H1T2
cg27394486	0.5840925	-224.0106	-0.2167288	0.8008213	C15orf2
cg04245402	0.2816779	-130.9797	-0.2168075	0.4984854	C19orf21
cg04893119	0.2191464	-135.7415	-0.2169585	0.436105	PI15
cg13813391	0.511143	-173.9554	-0.2170066	0.7281497	CMTM2
cg12878228	0.3402456	-132.5141	-0.2170307	0.5572764	PRSS1
cg10818781	0.4043183	-140.245	-0.217043	0.6213614	PHB2
cg00895324	0.4890103	-164.1299	-0.2170513	0.7060616	PCP4
cg01637734	0.4004023	-139.7323	-0.2171453	0.6175476	CD5L
cg26111757	0.4709859	-157.5133	-0.2171469	0.6881328	C20orf185
cg20932053	0.4972988	-168.0407	-0.217262	0.7145607	CPM
cg21038703	0.4403934	-148.5839	-0.2173203	0.6577138	ASB16
cg08157292	0.3638799	-135.213	-0.2175336	0.5814134	PPP1R7
cg06625767	0.5548801	-201.6053	-0.2175353	0.7724154	F12
cg18294257	0.1683637	-145.1941	-0.2175619	0.3859255	SEC14L3
cg18665384	0.4396086	-148.9671	-0.2176832	0.6572918	FLJ43505
cg03557698	0.6514012	-321.1516	-0.2178113	0.8692125	C1orf177
cg09256683	0.3501018	-134.357	-0.2178668	0.5679686	CCL14
cg19756068	0.4151723	-143.7057	-0.2179558	0.6331282	CYP2B6
cg12019109	0.3712807	-136.6958	-0.2179871	0.5892678	AZGP1
cg01869233	0.1947005	-140.6589	-0.218089	0.4127895	C20orf75
cg10417559	0.5605789	-207.4243	-0.2180997	0.7786786	LMO6
cg07197059	0.1736573	-144.8529	-0.2182436	0.3919009	EFS
cg12610070	0.07413922	-179.7906	-0.2183644	0.2925036	TSPAN15
cg24304714	0.2191436	-137.5163	-0.2184564	0.4376	LCE1C
cg07895149	0.04221556	-199.1546	-0.2184665	0.260682	FAM26B
cg27413508	0.2290322	-136.4914	-0.2185328	0.447565	COX4I2
cg11346450	0.5081005	-175.9323	-0.2186785	0.726779	UGT1A3
cg24959428	0.3772226	-138.5896	-0.2187981	0.5960206	GBP6
cg11947493	0.06876281	-183.2344	-0.2188021	0.2875649	TNFRSF10 D
cg18946226	0.1997719	-140.7005	-0.2188236	0.4185955	MYR8
cg23047271	0.1189278	-160.9094	-0.2188593	0.3377871	PRICKLE2
cg02787991	0.0521865	-193.1426	-0.2189128	0.2710993	SECTM1

cg08886154	0.3267592	-134.2325	-0.218923	0.5456822	PAX4
cg14435807	0.1964029	-141.4095	-0.2189553	0.4153582	LOXL1
cg15234096	0.4806633	-164.4338	-0.2189617	0.699625	LIX1
cg17051440	0.4401723	-151.4448	-0.2190818	0.6592541	CLDN2
cg27513764	0.4443951	-152.6489	-0.219126	0.6635211	EFCAB3
cg25691167	0.04352194	-199.1413	-0.2191653	0.2626873	FERD3L
cg26796190	0.05529689	-191.5952	-0.2192307	0.2745275	PYY
cg11765205	0.1691265	-147.0919	-0.2192926	0.3884191	ARL11
cg11271605	0.06359672	-186.7324	-0.2193003	0.2828971	STEAP4
cg19464944	0.3258629	-134.9559	-0.2195054	0.5453683	FCGR1A
cg19998328	0.3448662	-136.273	-0.2195904	0.5644566	LOC90580
cg22253945	0.5642086	-214.4371	-0.2196057	0.7838143	GPR45
cg10478221	0.295736	-134.3514	-0.219615	0.5153511	WFIKKN1
cg20176989	0.3795344	-140.1414	-0.2196535	0.5991879	KIR3DL2
cg01678091	0.5313656	-191.6507	-0.2199598	0.7513254	MAGEL2
cg16536450	0.1941599	-143.1292	-0.2200889	0.4142489	UNQ2541
cg04201347	0.3106911	-135.2078	-0.2201276	0.5308187	PRRG2
cg17264470	0.6342062	-300.0241	-0.2201461	0.8543522	FGF21
cg02436686	0.2452273	-136.9648	-0.2201622	0.4653894	GMFG
cg09418321	0.2590023	-136.1525	-0.2202449	0.4792472	DYRK4
cg25697314	0.3959218	-143.6953	-0.2203423	0.6162641	IL26
cg21283680	0.4299857	-151.1536	-0.2205099	0.6504956	SH3BP5
cg03473518	0.08819252	-175.5075	-0.2205285	0.308721	GJB6
cg22247240	0.3882276	-142.9196	-0.2206712	0.6088988	C14orf115
cg01375994	0.3340873	-137.0772	-0.2207635	0.5548508	MXRA5
cg18538812	0.2209456	-140.2407	-0.2208844	0.44183	GIF
cg15869022	0.3385827	-137.5363	-0.2208846	0.5594673	GPR17
cg19832721	0.4032701	-145.9696	-0.220953	0.6242231	KIAA1267
cg11263296	0.08788155	-176.235	-0.2210053	0.3088868	CCDC64
cg25552889	0.5705702	-224.0816	-0.2210959	0.7916662	C3orf40
cg11405695	0.3023624	-136.8433	-0.2215078	0.5238702	ATAD3C
cg06615154	0.4653252	-163.6456	-0.221595	0.6869202	S100A3
cg26385286	0.2510631	-138.3061	-0.2216004	0.4726635	GCNT2
cg00152644	0.5996249	-256.0463	-0.2216013	0.8212262	SPRR2E
cg17386181	0.5113316	-183.8912	-0.2216226	0.7329543	MT1B
cg01899253	0.2300261	-140.1652	-0.2216457	0.4516717	FLT1
cg24625388	0.2909261	-137.1249	-0.2217905	0.5127165	NEBL
cg21541083	0.5846298	-240.3475	-0.2219664	0.8065962	STXBP2
cg16713808	0.4305414	-153.749	-0.2220292	0.6525706	CRNN
cg11695684	0.55246	-211.6694	-0.2221133	0.7745733	CHRND
cg05301852	0.3725627	-142.7497	-0.2221172	0.59468	FABP1
cg15589427	0.5123097	-185.7342	-0.2222306	0.7345403	MUC4
cg20802392	0.2954502	-137.7523	-0.2222802	0.5177305	CTSK
cg17910564	0.3571658	-141.1437	-0.2222942	0.57946	VDAC3
cg02719634	0.5613961	-219.5149	-0.2223199	0.783716	SLC22A18

					AS
cg12970084	0.4554982	-161.6629	-0.2223577	0.677856	ELF3
cg20401945	0.1551918	-154.1231	-0.2223998	0.3775916	ASPHD1
cg24898863	0.1649623	-152.2313	-0.222789	0.3877513	S100A8
cg17542495	0.3562565	-141.9153	-0.2229066	0.5791631	GJB1
cg07126559	0.536364	-202.0106	-0.2229459	0.7593099	SGCG
cg12339029	0.5035224	-182.7094	-0.2229659	0.7264883	MYL1
cg00690280	0.284081	-138.7964	-0.2230377	0.5071188	WFDC10B
cg09686308	0.2636642	-139.4449	-0.2230748	0.486739	CIB3
cg16175725	0.5781155	-237.2738	-0.22313	0.8012456	TCF1
cg10248727	0.5871576	-247.732	-0.2234514	0.810609	LCN1
cg17199658	0.1948837	-147.1178	-0.223471	0.4183547	MGC3971 5
cg19787037	0.3244883	-141.2455	-0.2242672	0.5487555	SPAG11
cg13735697	0.5592067	-223.2655	-0.2243484	0.7835552	MYOG
cg18389810	0.6170886	-289.843	-0.2243766	0.8414651	C14orf8
cg02192965	0.466028	-169.2007	-0.224445	0.690473	SLC3A1
cg02046017	0.2452537	-142.5378	-0.2246582	0.469912	LOC22007 0
cg14826683	0.3159659	-141.4468	-0.2247215	0.5406874	SPRR2D
cg12069309	0.05419898	-199.1859	-0.224734	0.278933	SEMA3B
cg24949488	0.4483688	-163.7039	-0.2247871	0.6731559	DNTT
cg24724583	0.2042889	-147.2377	-0.2247881	0.429077	PLXDC1
cg17699374	0.236235	-143.4334	-0.2247939	0.4610289	MGC3520 6
cg25889160	0.6182504	-293.8655	-0.2249525	0.8432028	SERPINA5
cg16899036	0.3887607	-149.4908	-0.224973	0.6137337	HOMER3
cg08260891	0.07103567	-189.7874	-0.2250439	0.2960796	PPGB
cg23382741	0.4441042	-162.9185	-0.2251059	0.6692101	PPP1R14D
cg18462653	0.1794356	-152.1623	-0.2252869	0.4047224	DEFB119
cg14150666	0.6101303	-283.0993	-0.2252996	0.8354299	IL8RB
cg02656594	0.179003	-152.2682	-0.2253085	0.4043115	IL21R
cg05873268	0.5043819	-188.4291	-0.2254062	0.7297881	TPSAB1
cg00466436	0.2879899	-141.8464	-0.2254218	0.5134117	DEFB126
cg27157038	0.5245107	-200.3029	-0.2254696	0.7499803	DNTT
cg14724265	0.3324461	-143.5034	-0.2255582	0.5580043	PPEF2
cg11884243	0.5673147	-234.791	-0.2258155	0.7931302	FCN2
cg13269964	0.539158	-211.3623	-0.2258623	0.7650203	CD300LG
cg27160701	0.3551433	-146.1363	-0.2259488	0.5810921	SBEM
cg08403419	0.4211087	-157.9952	-0.2260093	0.647118	RLN3R2
cg19055231	0.05145439	-202.6662	-0.2260873	0.2775417	STAC
cg07846167	0.1047473	-175.3048	-0.2261591	0.3309064	FBLIM1
cg12943082	0.5181975	-198.0099	-0.2261671	0.7443646	CCL26
cg18783781	0.5446098	-216.3206	-0.226176	0.7707858	MGC4399
cg00673191	0.2208605	-146.7696	-0.2261807	0.4470413	DOPEY2

cg17915429	0.431575	-161.1473	-0.2262089	0.6577839	PGLYRP2
cg19642007	0.4449609	-165.2708	-0.2262767	0.6712376	TNNT3
cg25778479	0.1615061	-157.3392	-0.226286	0.3877921	ANKMY2
cg10037005	0.1763295	-154.0781	-0.226345	0.4026746	CD37
cg12891678	0.1830525	-152.7966	-0.2263812	0.4094338	SPRR2D
cg04891836	0.4182208	-157.9778	-0.2264282	0.644649	TNFSF14
cg22909609	0.2755294	-143.3806	-0.226487	0.5020164	ITGBL1
cg05755354	0.3323494	-144.8529	-0.2265263	0.5588757	FRMD4A
cg12992720	0.2382919	-145.4538	-0.2265437	0.4648356	EDG4
cg16794682	0.5268843	-204.4933	-0.2265614	0.7534457	CCND1
cg05600717	0.07995365	-187.1476	-0.2266461	0.3065997	FLJ13639
cg11015241	0.634205	-329.2807	-0.2268097	0.8610147	ATP10A
cg21825364	0.6830851	-350.9073	-0.2269108	0.9099959	VCY
cg24765079	0.2173192	-148.5839	-0.2272957	0.4446149	CDH1
cg23765993	0.2532326	-145.3878	-0.2273368	0.4805694	SPINLW1
cg06437862	0.5326996	-210.6842	-0.2274309	0.7601305	TUBA2
cg01993576	0.1621737	-158.7947	-0.2275828	0.3897566	SLC29A1
cg21643191	0.3965181	-155.214	-0.2277313	0.6242494	ABCB5
cg13760253	0.4203822	-160.7392	-0.2277336	0.6481158	DNAJC5B
cg01718139	0.5379295	-215.6454	-0.2278644	0.7657939	UNQ3033
cg16480209	0.6291769	-325.068	-0.2279693	0.8571462	CNGB1
cg19421752	0.1716104	-157.3165	-0.2281314	0.3997418	SLC6A18
cg02966851	0.2592571	-146.3076	-0.228301	0.4875581	C6orf149
cg11052143	0.1789931	-156.0509	-0.2283745	0.4073675	ALS2CR11
cg10329418	0.2383638	-147.8512	-0.2284304	0.4667942	PON3
cg09038914	0.5243647	-207.6023	-0.2285109	0.7528756	GFAP
cg26531804	0.2487185	-147.2117	-0.2285404	0.4772589	SPINT1
cg12228229	0.4984097	-192.2255	-0.2285881	0.7269978	DLG4
cg02280309	0.42963	-164.9054	-0.2286949	0.658325	PKLR
cg23355492	0.4103187	-159.8808	-0.2287142	0.6390329	SP6
cg04705866	0.4650277	-177.1943	-0.2288022	0.6938299	GZMK
cg20342105	0.270357	-146.6669	-0.2288948	0.4992518	BSCL2
cg09142399	0.426912	-164.8276	-0.2290908	0.6560028	CRYZ
cg11297236	0.2800744	-146.7874	-0.2291363	0.5092107	PDILT
cg23696886	0.1641714	-160.3256	-0.2291782	0.3933496	PDLIM2
cg01507173	0.4048771	-159.6427	-0.2293324	0.6342095	IL1F5
cg21301440	0.11227	-176.3406	-0.2293641	0.3416341	CYGB
cg23807646	0.367607	-152.808	-0.2293968	0.5970038	SLC26A8
cg06906435	0.3065723	-147.6392	-0.2295409	0.5361133	FLJ25773
cg01248426	0.2026985	-153.5775	-0.2297055	0.432404	ATP6V0D2
cg14168975	0.346471	-150.7108	-0.2297406	0.5762116	ALAS2
cg16998872	0.5256788	-211.8899	-0.2298679	0.7555467	GYPE
cg04254916	0.6321538	-339.4816	-0.2299014	0.8620552	KRT5
cg06385087	0.05879917	-203.0736	-0.2299806	0.2887798	CTSZ
cg13859324	0.270193	-148.4751	-0.230262	0.500455	UNC45B

cg23065097	0.2097233	-153.3051	-0.2302776	0.4400008	FKBP1B
cg22022041	0.5188516	-208.619	-0.2304199	0.7492716	CCR9
cg06985415	0.6165904	-313.8571	-0.2304736	0.847064	C10orf39
cg13705284	0.2825772	-148.5839	-0.2304995	0.5130768	ACOX2
cg03294491	0.1966875	-155.52	-0.2305071	0.4271945	SMAD2
cg24816455	0.2476251	-149.8381	-0.2305128	0.4781379	SEMA3B
cg08555657	0.5143694	-206.0144	-0.2305465	0.7449159	SPRR2E
cg25101056	0.4583081	-178.0106	-0.2305754	0.6888835	KCNG4
cg16008138	0.2298659	-151.5702	-0.2307297	0.4605955	RNF190
cg05696092	0.2538898	-149.9525	-0.2308896	0.4847794	NOSIP
cg22478614	0.3275118	-150.7156	-0.2309271	0.5584389	DEFB4
cg27069753	0.29301	-149.2766	-0.2309805	0.5239905	ELA3B
cg00687674	0.2423377	-150.833	-0.2309857	0.4733233	TMEM84
cg21495715	0.4840759	-190.134	-0.2310039	0.7150798	SLC5A10
cg09923671	0.4514466	-176.2452	-0.2310114	0.682458	GATA5
cg13281868	0.4304577	-169.5532	-0.231183	0.6616407	C6orf142
cg17141902	0.3959036	-160.7659	-0.2312211	0.6271248	NINJ1
cg03956628	0.1680624	-162.2299	-0.2314013	0.3994637	MLH1
cg00134787	0.3592277	-154.8626	-0.2315428	0.5907705	MYH1
cg00819362	0.5384888	-226.6992	-0.2318727	0.7703615	CLIPR-59
cg09448875	0.3222505	-151.7146	-0.2318981	0.5541486	ABCC2
cg22220722	0.5414824	-229.5885	-0.2320251	0.7735075	PLA2G2A
cg02397720	0.1140501	-179.1648	-0.2321042	0.3461542	RAB17
cg23458892	0.4211052	-168.9052	-0.2323492	0.6534544	SIGLEC7
cg15518950	0.2399518	-152.7701	-0.2323629	0.4723147	PRP2
cg08424423	0.5684884	-256.0872	-0.2323891	0.8008775	CDSN
cg22013966	0.6085786	-308.7188	-0.2324064	0.8409851	SERPINA13
cg22189286	0.4585115	-181.7559	-0.2324412	0.6909527	HSPB8
cg25994725	0.3292266	-153.4918	-0.2327999	0.5620265	C6orf81
cg19592945	0.1893228	-159.7636	-0.2328193	0.4221421	P2RXL1
cg07879977	0.2922124	-151.7842	-0.2328299	0.5250422	OR1F1
cg06785429	0.2454114	-153.0804	-0.2329023	0.4783137	DCUN1D1
cg22805308	0.2908704	-151.9169	-0.2329435	0.5238139	PLEKHG5
cg24698533	0.4500591	-179.5296	-0.2329807	0.6830398	LYZL2
cg07790638	0.4260127	-171.5296	-0.233036	0.6590487	LOC91431
cg18204685	0.4529203	-180.9862	-0.2331687	0.686089	BTD
cg16175792	0.3125194	-153.1801	-0.2333517	0.5458711	HSD3B1
cg03104936	0.4046973	-166.7381	-0.2335962	0.6382935	GRB10
cg15741706	0.4614491	-185.3063	-0.233613	0.6950621	CXorf48
cg02981703	0.5761535	-269.739	-0.2339011	0.8100545	CA6
cg19987219	0.4269951	-173.7807	-0.2341024	0.6610975	FLJ32011
cg06885782	0.2540874	-154.1517	-0.2341205	0.4882079	KCNQ4
cg21808053	0.2914125	-153.6368	-0.2342061	0.5256186	DIRAS3
cg01987509	0.3510443	-157.9987	-0.2343061	0.5853504	PGR

cg20261167	0.1784396	-163.7647	-0.2343406	0.4127803	SPP1
cg26895595	0.2702616	-153.9591	-0.2343987	0.5046603	MAGEB3
cg21789545	0.2413751	-155.3196	-0.2344029	0.475778	COL9A1
cg16466334	0.4219441	-172.8054	-0.2344114	0.6563554	MMP3
cg07595943	0.6823382	-350.9073	-0.2344543	0.9167925	LOC16193 1
cg22609784	0.2160303	-157.9952	-0.2345483	0.4505785	MSX1
cg15060813	0.2748302	-154.1424	-0.2345971	0.5094273	LRFN3
cg05341878	0.3058885	-154.675	-0.2346421	0.5405307	RIMS2
cg09528351	0.4567631	-185.6161	-0.2347355	0.6914986	PIK3R5
cg03291145	0.4095829	-170.16	-0.2348679	0.6444508	ARSF
cg11061975	0.3117694	-155.4776	-0.2350102	0.5467796	SIRPB2
cg15798153	0.1905266	-162.3667	-0.2350137	0.4255403	PFTK1
cg00463202	0.5348554	-232.5842	-0.2350902	0.7699456	ADPRHL1
cg09492887	0.1130298	-183.5744	-0.2352288	0.3482586	SLC26A5
cg10368842	0.3807105	-164.5547	-0.2354437	0.6161542	C10orf81
cg26333641	0.4712773	-193.9764	-0.2357056	0.7069829	IL22
cg13703437	0.3960448	-168.2897	-0.2357234	0.6317682	FYB
cg07443748	0.3406576	-159.1145	-0.235846	0.5765036	CESK1
cg14587868	0.4104374	-172.1756	-0.2358879	0.6463253	TGM1
cg14107638	0.4402544	-181.8721	-0.2360671	0.6763214	MAGEA5
cg13126790	0.4434139	-183.6455	-0.2363805	0.6797944	FLJ27255
cg17288121	0.4067739	-172.4792	-0.2366167	0.6433907	DEFB103A
cg14696870	0.3900684	-168.7868	-0.2368159	0.6268843	FCER1A
cg01459162	0.5528036	-254.0708	-0.2369291	0.7897327	PADI3
cg19985911	0.5192215	-225.563	-0.2370173	0.7562389	AVIL
cg26420196	0.2351041	-159.3287	-0.2370222	0.4721264	GAS6
cg19841506	0.1106095	-186.8293	-0.2370513	0.3476608	ZMYND15
cg03379131	0.04891143	-218.6526	-0.237059	0.2859704	ADAM15
cg09799714	0.4701945	-196.3864	-0.2370938	0.7072884	PDZD3
cg05330360	0.3038379	-158.5482	-0.2373992	0.5412371	ZPBP2
cg08728865	0.5539772	-257.4603	-0.2376339	0.7916111	NALP7
cg00075967	0.4774713	-201.5144	-0.237791	0.7152622	STRA6
cg05955301	0.2729245	-158.6876	-0.2378604	0.5107849	PRELP
cg18565510	0.6731143	-350.9073	-0.2379687	0.911083	CENTB5
cg23022999	0.3996152	-173.2894	-0.2381334	0.6377487	FLJ45909
cg18333690	0.4589587	-194.542	-0.2386647	0.6976234	PADI4
cg11584690	0.1224123	-184.757	-0.2387963	0.3612086	ZNF574
cg19257200	0.508251	-222.385	-0.2388061	0.7470571	SOX10
cg07864297	0.1751472	-170.2169	-0.2388073	0.4139546	ESRRB
cg10370591	0.3806129	-170.2233	-0.2388958	0.6195087	TPO
cg23328404	0.3664506	-167.9169	-0.2391214	0.605572	ChGn
cg08023751	0.4879117	-210.3662	-0.239152	0.7270638	MERTK
cg20516209	0.3938994	-173.791	-0.2392322	0.6331316	EMILIN1
cg05131835	0.4142895	-179.306	-0.2392481	0.6535376	GH2

cg04744379	0.5044484	-221.1051	-0.2393131	0.7437615	KLK15
cg16176600	0.4951427	-215.2786	-0.2393818	0.7345245	FRK
cg26353877	0.5141768	-228.1043	-0.239401	0.7535778	APCS
cg27337148	0.1969946	-167.2433	-0.23956	0.4365545	CAMK1G
cg20713492	0.09101804	-198.4492	-0.2395982	0.3306163	AQP10
cg01568736	0.5000551	-218.9509	-0.2396178	0.7396729	SERPINB7
cg16381688	0.3092366	-162.4767	-0.2399009	0.5491374	THEM2
cg22971191	0.276719	-161.7493	-0.2400569	0.516776	SLC10A2
cg00209066	0.5625432	-274.9658	-0.2401754	0.8027186	BRD1
cg01325515	0.5505856	-262.1735	-0.2402402	0.7908258	CTAG2
cg13530039	0.2933781	-162.2794	-0.2402553	0.5336334	CHRM1
cg16507522	0.5216398	-236.4668	-0.2404053	0.7620451	SERPINA3
cg14297029	0.6012787	-330.2034	-0.2404382	0.8417169	SSTR3
cg08784110	0.2990796	-163.1889	-0.2407375	0.5398171	MAS1
cg06226384	0.1633861	-175.5028	-0.2408417	0.4042277	CACNG5
cg18920397	0.46179	-200.584	-0.2409194	0.7027093	LY9
cg25710140	0.4495993	-195.4037	-0.2410518	0.6906511	MID1
cg16075940	0.5045261	-226.2	-0.2413285	0.7458546	FLJ37396
cg18982568	0.3510721	-169.0281	-0.2413616	0.5924338	KRT1B
cg26523005	0.306715	-164.5915	-0.2414375	0.5481524	ZNF662
cg03782727	0.6137362	-350.9073	-0.2414793	0.8552155	FFAR1
cg05500074	0.4656135	-203.8556	-0.2415693	0.7071827	TSKS
cg18766847	0.3932123	-178.1322	-0.2418186	0.6350309	ACMSD
cg15895197	0.158151	-178.0749	-0.241832	0.399983	EMILIN1
cg21723486	0.3468644	-169.2628	-0.2418995	0.5887639	TP73L
cg05822532	0.2986178	-164.8699	-0.2419054	0.5405232	ELN
cg22855405	0.1716661	-175.5144	-0.2422623	0.4139284	TINAGL1
cg03366382	0.4890176	-218.4566	-0.24227	0.7312876	INS
cg09931793	0.448926	-197.6521	-0.242273	0.6911989	OR2K2
cg25043279	0.4432854	-195.3554	-0.242311	0.6855964	C7orf33
cg06810461	0.4381541	-193.3352	-0.242316	0.6804701	AGRP
cg05135288	0.2329063	-166.7095	-0.2423375	0.4752438	RHOT2
cg27349244	0.4093955	-183.5885	-0.242368	0.6517636	MLXIP
cg25531166	0.524059	-243.8339	-0.242375	0.766434	CTAG1B
cg15670863	0.4231112	-187.985	-0.2423877	0.6654989	SPACA4
cg06022562	0.5699813	-292.3829	-0.2425939	0.8125752	FLJ13841
cg06259570	0.3810374	-176.6408	-0.2426255	0.6236629	MMP27
cg14141399	0.2637815	-166.0084	-0.2429678	0.5067493	HAS1
cg05112299	0.2549141	-166.6003	-0.2432013	0.4981154	OR7A17
cg21964481	0.4155403	-187.0949	-0.2432394	0.6587797	SLC34A3
cg24623694	0.2142798	-169.8216	-0.2432625	0.4575422	PRX
cg15903395	0.4116313	-186.0563	-0.2433438	0.6549752	FLJ25369
cg09207718	0.3235166	-168.8571	-0.2433947	0.5669113	CYP1A2
cg20856834	0.389303	-179.9812	-0.2434166	0.6327196	OR12D3
cg25168545	0.2426487	-167.5465	-0.2434468	0.4860955	GIMAP1

cg24489034	0.4475341	-199.5867	-0.2434967	0.6910307	LW-1
cg02882813	0.5218526	-245.4055	-0.2435917	0.7654443	CST5
cg19372178	0.2275309	-168.9052	-0.2435988	0.4711297	TMEM16G
cg14015044	0.04604905	-229.3737	-0.2436859	0.289735	TNFRSF10C
cg02786019	0.3388598	-171.1422	-0.2437592	0.5826191	TRPV6
cg06836849	0.1417844	-185.1839	-0.2438387	0.3856231	SLC17A8
cg11898695	0.4907263	-223.4912	-0.2438994	0.7346257	PTCRA
cg19971655	0.6510079	-350.9073	-0.24391	0.8949178	BSND
cg10837843	0.1073998	-197.1173	-0.2439292	0.351329	DUSP1
cg16545105	0.4129382	-187.9402	-0.2441233	0.6570615	CRHBP
cg00319692	0.2559521	-168.0407	-0.2442444	0.5001965	ATP6V0D2
cg16377880	0.3531597	-174.0202	-0.2442524	0.5974121	CYP4F3
cg00795812	0.1479124	-184.022	-0.2442826	0.392195	PDCD1
cg18473117	0.453528	-204.2233	-0.2444073	0.6979352	CCDC22
cg05547500	0.5844959	-320.561	-0.2446399	0.8291358	TXNDC2
cg11939496	0.4132572	-189.6451	-0.2449679	0.6582251	CD244
cg18192417	0.1370659	-188.0992	-0.2449868	0.3820527	NEBL
cg15005385	0.3242052	-171.5215	-0.2450848	0.56929	CCL3L1
cg08356693	0.2940523	-169.4868	-0.2451619	0.5392143	ITLN1
cg12354377	0.2823775	-169.1964	-0.2451981	0.5275756	ANK3
cg19464252	0.2617358	-169.2339	-0.2452095	0.5069454	FBS1
cg25462291	0.1206812	-193.9982	-0.2453485	0.3660297	HEYL
cg03534410	0.616232	-350.9073	-0.2454309	0.8616629	TMEM40
cg17706173	0.1927849	-176.0696	-0.2456352	0.43842	C16orf30
cg18530716	0.09523508	-204.657	-0.2457115	0.3409466	SLC16A11
cg01479232	0.349361	-176.1423	-0.2459197	0.5952808	C20orf54
cg26504906	0.2368069	-171.4516	-0.2459755	0.4827824	PRSS16
cg04655481	0.4559184	-209.0061	-0.2460748	0.7019932	GPR21
cg03872376	0.3897094	-185.3063	-0.2463292	0.6360386	ZP4
cg11113534	0.6095824	-350.9073	-0.2463909	0.8559733	C20orf70
cg24621042	0.296464	-171.4813	-0.2464228	0.5428869	SERPINA1
cg19465374	0.5284137	-259.5718	-0.2464707	0.7748845	AZGP1
cg19382175	0.4808663	-223.6324	-0.24648	0.7273464	PDE6A
cg19226099	0.306684	-172.4143	-0.2466308	0.5533149	MC3R
cg24214470	0.4612129	-212.9703	-0.2466503	0.7078633	SERPINF1
cg13053608	0.2642931	-171.3309	-0.246697	0.5109901	LGP1
cg08766149	0.4392699	-202.8599	-0.2467545	0.6860244	GZMB
cg25107791	0.3311979	-174.9779	-0.2467652	0.5779631	CLPS
cg00520135	0.6086764	-350.9073	-0.2468423	0.8555187	TPM1
cg09971646	0.1621413	-184.0239	-0.2469941	0.4091354	DLK1
cg04450876	0.5375122	-270.3395	-0.2470804	0.7845926	FAM112B
cg03752885	0.2779784	-172.0201	-0.2471697	0.5251481	DAPK3
cg11750883	0.3120439	-173.765	-0.2472331	0.5592771	C1orf42
cg00367281	0.386657	-186.3491	-0.2473636	0.6340206	CHRNB3

cg17091851	0.280007	-172.557	-0.2475104	0.5275174	LOC348174
cg09573795	0.3370109	-177.355	-0.2477783	0.5847892	MSX1
cg03916421	0.1779967	-182.0055	-0.2480288	0.4260255	LOC132321
cg22780475	0.3325285	-177.6143	-0.2483072	0.5808358	CBLC
cg00350478	0.3563172	-181.4296	-0.2483614	0.6046786	FRMD1
cg01917648	0.2766789	-173.8234	-0.2483953	0.5250742	SPIC
cg11456838	0.2966399	-174.7995	-0.2485874	0.5452273	LOC202459
cg05492113	0.4173601	-198.2513	-0.2486951	0.6660552	TUB
cg19812619	0.5910724	-348.563	-0.2487647	0.8398371	ITGB7
cg05564657	0.631236	-350.9073	-0.248958	0.880194	AADAC
cg06825166	0.2854198	-175.343	-0.2492723	0.5346922	TMEM10
cg07123548	0.5677515	-314.2797	-0.2493117	0.8170632	HIPK4
cg14652095	0.2617222	-175.2812	-0.249368	0.5110902	HIST1H1A
cg14162076	0.5803103	-333.5074	-0.2494431	0.8297534	CLEC4D
cg21745164	0.2857738	-175.5991	-0.2494467	0.5352205	LOC63928
cg21458041	0.3760006	-187.8548	-0.2496661	0.6256666	TNP2
cg07446846	0.5402787	-281.901	-0.2497947	0.7900734	SLC6A8
cg16222568	0.4599063	-219.5274	-0.2498147	0.709721	APEG1
cg23413307	0.3941059	-193.3295	-0.2500397	0.6441457	LCE1F
cg12582965	0.6087851	-350.9073	-0.2500845	0.8588696	ATP10A
cg16463460	0.2313762	-177.7201	-0.250118	0.4814942	WT1
cg02658251	0.3980147	-195.0707	-0.2503539	0.6483686	DEFB4
cg14333565	0.5411022	-284.6871	-0.2503644	0.7914666	NRTN
cg22627427	0.5946591	-350.9073	-0.2504698	0.8451289	C11orf9
cg18971671	0.4436382	-212.9485	-0.2505309	0.6941691	TULP2
cg10490064	0.465119	-224.1186	-0.2505617	0.7156807	CRYBB2
cg00174500	0.2333053	-179.0546	-0.2511387	0.4844441	CMTM5
cg16358738	0.473022	-230.8851	-0.2514282	0.7244502	AGXT
cg04816348	0.614647	-350.9073	-0.2516205	0.8662676	CLEC4G
cg13424229	0.5240545	-271.1755	-0.2516502	0.7757047	CPA3
cg17788013	0.4457936	-216.6452	-0.2517211	0.6975147	SPINK5
cg12029639	0.1884555	-185.3217	-0.2518015	0.440257	MAB21L1
cg06233503	0.3121909	-180.9862	-0.2518145	0.5640055	KCNQ1
cg02601403	0.5175419	-265.6017	-0.2518361	0.7693781	TBC1D3C
cg26917999	0.3767893	-191.9695	-0.251842	0.6286312	LZTS1
cg23580945	0.4980093	-249.1964	-0.2518596	0.7498689	FLJ43826
cg04739485	0.3668079	-189.7077	-0.2519	0.618708	MLXIP
cg05559445	0.2271053	-180.7139	-0.2519604	0.4790657	CDKN1C
cg18490846	0.2738503	-179.3574	-0.2521126	0.5259629	C17orf73
cg13608094	0.6114283	-350.9073	-0.252701	0.8641294	CCND1
cg12266049	0.5711331	-332.6861	-0.2527472	0.8238803	CCND1
cg08475088	0.5957056	-350.9073	-0.2527931	0.8484987	NALP9

cg21985470	0.2822447	-180.7139	-0.252865	0.5351098	PKLR
cg04557383	0.07212877	-226.2256	-0.2531221	0.3252509	MT1H
cg26457013	0.3774127	-194.4951	-0.2531404	0.6305531	TMEM86B
cg23464269	0.2896488	-181.8236	-0.2533827	0.5430315	UGT1A3
cg05654163	0.3764358	-195.8936	-0.2540453	0.6304811	SLC39A2
cg03087937	0.432557	-215.5817	-0.2540568	0.6866138	MUC15
cg06958211	0.3432128	-188.9651	-0.2541486	0.5973614	PAK6
cg19000186	0.4142048	-208.7541	-0.2544612	0.668666	CNGA1
cg26218269	0.2342199	-183.9594	-0.2545534	0.4887733	MAB21L2
cg24910675	0.1594974	-195.0963	-0.2546136	0.4141111	ENG
cg19845843	0.455281	-228.5964	-0.2547853	0.7100663	CXorf20
cg13439299	0.5698609	-339.3887	-0.2548905	0.8247514	DNAJC5G
cg01668126	0.4320999	-217.2703	-0.2549283	0.6870282	MSR1
cg24477636	0.3071865	-185.6198	-0.2549917	0.5621782	OR10H1
cg24331162	0.459417	-231.5745	-0.2550464	0.7144635	SYT8
cg04637372	0.3479323	-191.5028	-0.2551271	0.6030594	FLJ32784
cg04273431	0.1339154	-202.9057	-0.255153	0.3890684	PRR3
cg19903229	0.2802493	-184.1742	-0.2551539	0.5354032	C14orf105
cg09458237	0.4386228	-221.975	-0.2556592	0.694282	HSPA12B
cg00392257	0.3472518	-192.3436	-0.2556834	0.6029352	ISG20L2
cg06277277	0.2296256	-186.2598	-0.255922	0.4855476	NR1I3
cg03544379	0.2363008	-185.9764	-0.256019	0.4923198	OR7C2
cg00644033	0.6615375	-350.9073	-0.256029	0.9175664	MUC3B
cg19368582	0.3909751	-203.8685	-0.2560573	0.6470323	MMRN2
cg16673198	0.3814149	-201.0927	-0.2561057	0.6375206	CPNE4
cg08816023	0.2796646	-186.0659	-0.2563922	0.5360569	FGF1
cg12954718	0.2345788	-186.6541	-0.2564095	0.4909883	USP6
cg06489008	0.2967849	-187.172	-0.2565051	0.55329	CST11
cg19561774	0.6027277	-350.9073	-0.2565181	0.8592458	SLC22A2
cg10586756	0.4244114	-217.4762	-0.2565938	0.6810052	NUP93
cg19824441	0.3740127	-200.001	-0.2565977	0.6306103	ADMR
cg24516901	0.2079776	-189.5923	-0.2567803	0.4647579	FLJ22746
cg13125510	0.213477	-189.1798	-0.2569255	0.4704025	C11orf44
cg22585988	0.4292986	-221.1051	-0.2572476	0.6865462	PVRL4
cg00714377	0.1400816	-204.6783	-0.2577557	0.3978373	SLA2
cg03352153	0.4373219	-226.4778	-0.2579542	0.6952761	GLULD1
cg24825722	0.3377493	-194.5995	-0.2579682	0.5957175	ACADVL
cg08314660	0.589784	-350.9073	-0.2579715	0.8477555	PKP3
cg02202484	0.3573456	-198.4713	-0.2579791	0.6153247	SPRR4
cg19717326	0.1893071	-194.0196	-0.2580463	0.4473534	MYADM
cg14511156	0.2344914	-189.2549	-0.2581494	0.4926409	OSCAR
cg04995095	0.2664679	-188.5495	-0.2581788	0.5246466	CD300E
cg22083047	0.225532	-190.1659	-0.258356	0.483888	PRICKLE2
cg17741572	0.5385565	-309.6386	-0.2584832	0.7970396	CFB
cg08634024	0.5598983	-339.0388	-0.2585691	0.8184674	OR2F1

cg12850636	0.4564053	-238.3383	-0.2585711	0.7149763	TJP3
cg22549408	0.2388382	-189.7599	-0.2586423	0.4974805	PMAIP1
cg26884581	0.1387357	-206.353	-0.2586468	0.3973826	PYGM
cg17582777	0.3607234	-200.5261	-0.2586711	0.6193945	EFNA3
cg23704362	0.289257	-190.1842	-0.258697	0.547954	C8orf46
cg19292712	0.4715158	-248.3047	-0.2587235	0.7302393	SPAM1
cg17827767	0.556597	-336.6873	-0.2592106	0.8158076	LRRC21
cg26200585	0.3255991	-195.0885	-0.2593832	0.5849823	PRX
cg26628847	0.2452276	-190.7034	-0.2594376	0.5046653	PIP
cg10213812	0.6034673	-350.9073	-0.259544	0.8630112	FOXN1
cg09837803	0.2669092	-190.7034	-0.2595613	0.5264704	IL16
cg11161873	0.2757646	-191.1971	-0.2597373	0.535502	FLJ39575
cg10986043	0.2672952	-191.0641	-0.2597868	0.527082	TCAP
cg00563932	0.4470887	-236.1465	-0.259867	0.7069557	PTGDS
cg25477904	0.5480043	-327.1228	-0.2598901	0.8078945	PSG1
cg08996986	0.4181003	-222.1913	-0.2600261	0.6781263	EPS8L1
cg13552869	0.1378506	-208.6094	-0.2600383	0.3978889	SEZ6L2
cg14036856	0.3024036	-193.6352	-0.26018	0.5625836	MGC5242 3
cg01693350	0.2323849	-192.4514	-0.2601999	0.4925848	WT1
cg18988110	0.1998362	-196.0225	-0.2605063	0.4603425	ATAD4
cg01367992	0.32901	-197.7417	-0.2606158	0.5896258	LY9
cg10210238	0.193749	-197.4047	-0.2608596	0.4546087	CDKN2B
cg00226923	0.5026017	-279.2088	-0.2609124	0.7635141	FGD2
cg07947016	0.2209216	-194.4345	-0.260971	0.4818926	KLK2
cg18223379	0.2583263	-192.8563	-0.2609781	0.5193044	BPIL3
cg07441143	0.4283631	-229.6539	-0.2612724	0.6896355	SLURP1
cg00518911	0.2526055	-193.4879	-0.2613612	0.5139667	HOXA10
cg12061127	0.4072841	-220.8238	-0.2614806	0.6687647	WFDC9
cg08495878	0.6030308	-350.9073	-0.2615209	0.8645517	SERPINA4
cg05556717	0.3373571	-200.8929	-0.261593	0.5989501	CCL26
cg09343150	0.5133193	-291.9315	-0.2616369	0.7749562	MEN1
cg17829936	0.4237657	-228.3717	-0.2616762	0.6854419	TAAR5
cg15075718	0.3687111	-208.3191	-0.2617117	0.6304227	MFRP
cg13320683	0.4000895	-219.3373	-0.2621077	0.6621972	RHOBTB1
cg15422147	0.4263147	-230.6227	-0.2621389	0.6884536	SERPINB5
cg21453309	0.1714277	-203.2531	-0.2621888	0.4336165	FAM101A
cg07531356	0.3978758	-219.5499	-0.2626128	0.6604886	INSL6
cg03552103	0.3081545	-198.3076	-0.2626189	0.5707734	09-sep
cg03548857	0.1984917	-199.3213	-0.2626268	0.4611185	FFAR2
cg25915982	0.513095	-295.293	-0.2627711	0.7758661	GRB10
cg05485062	0.513974	-296.5872	-0.2628806	0.7768545	SERPINA1 2
cg24824840	0.5268826	-312.2926	-0.2632264	0.790109	SHANK1
cg20512303	0.3579061	-208.6279	-0.2633603	0.6212664	PDLIM4

cg15590780	0.233824	-197.4047	-0.2635162	0.4973402	USH2A
cg17192247	0.09382994	-230.0359	-0.2635514	0.3573814	MAPRE3
cg22341310	0.1657372	-206.521	-0.2635964	0.4293335	ZNF541
cg23753610	0.6664256	-350.9073	-0.2637647	0.9301904	DNAHL1
cg25141490	0.2393827	-197.71	-0.2638793	0.503262	IL17B
cg20551517	0.4952354	-282.3838	-0.2641943	0.7594296	GIP
cg01837574	0.2708502	-198.3088	-0.2643068	0.535157	TRAPPC1
cg03169180	0.3606009	-211.3846	-0.2644311	0.625032	NLGN2
cg24429836	0.1954286	-202.4436	-0.2644334	0.459862	LDHD
cg05700681	0.5259196	-316.638	-0.2647829	0.7907025	CCL22
cg23213217	0.2930357	-200.76	-0.2649889	0.5580245	DEGS1
cg17217677	0.3815378	-218.7594	-0.2650148	0.6465526	SMPD3
cg07676849	0.3204142	-204.5151	-0.265243	0.5856572	FOLR3
cg08390209	0.3203076	-204.7271	-0.2653783	0.5856858	CDKN2B
cg10779183	0.4766884	-270.5448	-0.2654925	0.7421809	ELA3A
cg13928961	0.4358717	-244.377	-0.2659131	0.7017848	K6IRS3
cg24628744	0.1378379	-217.5831	-0.2662339	0.4040718	H2AFY
cg18121684	0.4135907	-234.5535	-0.2665099	0.6801006	SERPINB1<br>3
cg02694395	0.2781877	-202.3479	-0.2665676	0.5447552	FMO4
cg24697329	0.4728671	-270.9779	-0.2667126	0.7395797	ARHGEF4
cg17240454	0.5331618	-333.1444	-0.2667997	0.7999614	SPDEF
cg24541550	0.3065735	-205.3957	-0.2668507	0.5734241	MRVI1
cg26422060	0.5188669	-316.644	-0.2671747	0.7860416	TBX10
cg24888049	0.1907402	-207.271	-0.2671783	0.4579185	FES
cg06818777	0.08895681	-238.2824	-0.267732	0.3566888	CHAD
cg21402035	0.334361	-211.7441	-0.2678427	0.6022036	GALR3
cg03782453	0.1165178	-226.9843	-0.2678651	0.3843829	FLJ90575
cg21277505	0.5612375	-350.9073	-0.2678931	0.8291306	LOC28436<br>1
cg17398613	0.4865465	-286.4203	-0.2681081	0.7546546	SLC37A1
cg06539449	0.4711343	-273.7114	-0.2681649	0.7392992	CCND1
cg25882366	0.1160158	-227.9531	-0.2683914	0.3844072	HOXB2
cg26473272	0.5988919	-350.9073	-0.2684971	0.867389	SYT8
cg25013053	0.4285365	-246.9893	-0.2686563	0.6971928	UNC45B
cg23776892	0.5078627	-309.7513	-0.2688099	0.7766726	MAGEA1
cg04132607	0.5901555	-350.9073	-0.2689689	0.8591244	GATA5
cg01774645	0.4135061	-240.1927	-0.2690079	0.682514	ARHGAP3<br>0
cg00042156	0.3259746	-212.8576	-0.2693172	0.5952918	MGC1629<br>1
cg11435943	0.2498085	-206.2636	-0.2694061	0.5192146	SERPINB1<br>2
cg03343942	0.2672321	-206.8345	-0.2695731	0.5368052	SLC39A5
cg15095327	0.2963475	-209.0736	-0.2696416	0.5659891	IL17RE

cg16242770	0.4924998	-296.8693	-0.2697211	0.7622209	KRTAP17-1
cg11161417	0.3938835	-233.2629	-0.2697487	0.6636322	SPACA3
cg01474260	0.3291576	-214.4444	-0.2698391	0.5989967	CESK1
cg24884084	0.1606845	-216.9496	-0.2698438	0.4305283	SPRR1B
cg09546307	0.4045425	-237.9865	-0.269854	0.6743965	CLEC4D
cg07967308	0.3594604	-221.975	-0.2699822	0.6294427	ACP5
cg15983538	0.4757426	-282.9326	-0.2700864	0.745829	SEMA4A
cg24309555	0.3562332	-221.2866	-0.2700927	0.6263258	APOB
cg18729973	0.4757217	-282.9602	-0.2701051	0.7458268	TFF1
cg21065959	0.3886691	-231.9739	-0.2701114	0.6587805	LCE1E
cg08859675	0.3603675	-222.9049	-0.2703235	0.6306909	PDE4A
cg07711097	0.3924531	-234.3866	-0.2705338	0.6629869	GML
cg00941549	0.3975407	-236.4832	-0.2705425	0.6680831	AKAP4
cg23278885	0.434485	-255.1497	-0.2706031	0.7050881	TGM6
cg14366490	0.3667573	-225.7551	-0.2708009	0.6375582	TXNL6
cg25778166	0.4535829	-269.3168	-0.2712753	0.7248582	FMO3
cg10414946	0.3594415	-224.9848	-0.2715178	0.6309593	MS4A2
cg04511534	0.4813157	-292.7527	-0.2717412	0.7530569	GGT6
cg10883352	0.4007869	-240.7054	-0.2718163	0.6726032	
cg18533225	0.4174205	-248.7168	-0.2718353	0.6892558	KLHDC7B
cg24919884	0.3856981	-235.4985	-0.27227	0.657968	ARHGEF16
cg17687962	0.4648553	-280.8503	-0.2724229	0.7372782	KLK3
cg13960126	0.368385	-230.5021	-0.2728426	0.6412276	CRB3
cg12732155	0.1881447	-216.8309	-0.2731358	0.4612805	LAPTM5
cg10125195	0.4172909	-251.7899	-0.2731489	0.6904398	LACRT
cg02595219	0.1900134	-216.7686	-0.2732689	0.4632823	KCNE3
cg11070419	0.2745329	-213.8654	-0.2734804	0.5480133	C4BPA
cg00134539	0.4360978	-264.2132	-0.2737508	0.7098486	UBASH3A
cg04048249	0.384017	-238.1394	-0.2737914	0.6578084	APOC3
cg11739626	0.4265621	-258.5622	-0.2737983	0.7003604	AKT1S1
cg12639234	0.2840528	-215.2786	-0.2738598	0.5579125	NAT2
cg06720660	0.3294073	-222.1824	-0.2739151	0.6033224	RNASE6
cg06275635	0.2848532	-215.5817	-0.2739894	0.5588427	PGLYRP3
cg20488657	0.4172121	-254.0258	-0.2740854	0.6912975	TFF3
cg02812142	0.3743053	-235.2887	-0.2741438	0.6484491	ACMSD
cg22970435	0.08460171	-249.8455	-0.2742497	0.3588514	SPATS1
cg04488758	0.3870915	-240.8304	-0.2744762	0.6615677	USP44
cg26946769	0.5061606	-328.7866	-0.2748719	0.7810324	MAPK4
cg23514672	0.5977372	-350.9073	-0.2749403	0.8726774	FLJ32871
cg03458191	0.4745395	-296.6616	-0.2750279	0.7495673	SAA1
cg08093398	0.3768441	-238.2908	-0.2751242	0.6519684	PSF1
cg18967533	0.2641532	-216.2074	-0.2751689	0.539322	KLK6
cg00622552	0.10388	-243.1485	-0.275536	0.379416	ODF3L1
cg11984608	0.5216067	-350.9073	-0.2756428	0.7972494	CLDN16

cg15648315	0.4421554	-273.3433	-0.2757137	0.717869	FLJ26443
cg08815403	0.2989485	-220.2943	-0.2757356	0.5746841	HSD17B13
cg19717150	0.3915321	-245.5676	-0.2757633	0.6672953	HNF4A
cg01861509	0.4014234	-250.4643	-0.2759435	0.6773669	SPOCK2
cg10894512	0.3578964	-233.8849	-0.2760824	0.6339787	ACTA2
cg22983092	0.3195071	-224.3412	-0.2761265	0.5956336	KRT25A
cg04567009	0.3157751	-224.0484	-0.276333	0.5921081	FCGR3B
cg25141995	0.2440492	-217.803	-0.276367	0.5204162	VDAC1
cg25781162	0.4775761	-303.8308	-0.2764422	0.7540183	ABCG5
cg22194129	0.3127117	-223.7945	-0.2764867	0.5891984	CLEC4C
cg24355048	0.3605424	-236.4261	-0.2768982	0.6374406	CTSG
cg22937320	0.4303398	-269.5462	-0.2771883	0.7075281	C9orf138
cg25659818	0.2980646	-223.1695	-0.2773778	0.5754424	CCL4
cg18396533	0.1414724	-233.3373	-0.2774608	0.4189331	DYDC1
cg24816866	0.4696712	-300.8889	-0.2778392	0.7475104	PARK2
cg09448880	0.3608518	-239.0637	-0.2781216	0.6389734	PGLYRP3
cg06531741	0.2575415	-221.2866	-0.278283	0.5358245	HTR3B
cg16964535	0.6310327	-350.9073	-0.2784814	0.9095141	DNAJC5G
cg05985767	0.3912248	-251.6893	-0.278496	0.6697208	ANPEP
cg03973663	0.153354	-231.8559	-0.2784999	0.4318539	LYN
cg21639401	0.385033	-249.9067	-0.2789076	0.6639407	FLJ31222
cg04337944	0.1995128	-224.8861	-0.2791863	0.4786991	FBLN1
cg12593411	0.3497784	-237.8651	-0.2792007	0.6289791	ANGPTL6
cg03977657	0.5092642	-349.6087	-0.2795417	0.788806	LAMB3
cg17095731	0.3633249	-243.0098	-0.2795883	0.6429132	LRP8
cg00750606	0.2594471	-224.3589	-0.2800384	0.5394855	CDA
cg08603768	0.375769	-249.2571	-0.2803127	0.6560817	WNT8A
cg07977490	0.4084282	-264.8641	-0.2804015	0.6888297	C16orf45
cg07950803	0.2268164	-224.8836	-0.2804092	0.5072255	CD1A
cg10464775	0.3907923	-256.2815	-0.2805438	0.6713361	LAMP1
cg18881269	0.3636721	-245.5676	-0.2807167	0.6443889	LEPREL2
cg04810997	0.3906679	-256.7471	-0.2807678	0.6714357	TAS2R60
cg25033144	0.4518402	-294.7061	-0.2807961	0.7326362	FLJ00060
cg16462075	0.443949	-288.8555	-0.2808729	0.7248219	MUC3B
cg22228134	0.2999097	-230.4126	-0.2811397	0.5810493	GZMH
cg21970438	0.4764955	-317.9115	-0.281146	0.7576416	TTLL2
cg01663968	0.06192365	-272.9997	-0.2815939	0.3435176	CTSZ
cg06236276	0.4764246	-319.6019	-0.2816781	0.7581028	SLC22A2
cg15782391	0.286655	-229.9919	-0.2819036	0.5685586	ACPT
cg25072962	0.3431043	-242.1157	-0.2822299	0.6253342	MGC35295
cg00436603	0.2662222	-228.901	-0.2823767	0.5485989	CYP2E1
cg17778867	0.3938404	-262.5024	-0.2825294	0.6763697	KRTAP10-8
cg08341924	0.4757243	-321.8601	-0.282602	0.7583263	TGM1

cg20416179	0.5079787	-350.9073	-0.2826465	0.7906252	C6orf71
cg15542496	0.4455658	-295.242	-0.2826895	0.7282554	PIP
cg14034870	0.3010431	-233.5574	-0.282696	0.5837391	SFTPG
cg02423618	0.3086978	-235.1442	-0.2828187	0.5915165	SPATA8
cg01827098	0.5121537	-350.9073	-0.2829538	0.7951075	GIMAP7
cg03609102	0.2878989	-232.8209	-0.283333	0.5712318	MUC5B
cg27341860	0.6078755	-350.9073	-0.2835156	0.891391	OR2L13
cg05569220	0.2962744	-235.5788	-0.2841644	0.5804387	FLJ44861
cg09426307	0.3630635	-253.4228	-0.2843855	0.647449	SEC14L3
cg09948350	0.5106841	-350.9073	-0.2844241	0.7951082	FLJ25084
cg08402568	0.3698232	-256.4083	-0.2845365	0.6543597	MGC3464 7
cg12022621	0.2658202	-233.4674	-0.2849191	0.5507392	LAX1
cg18056600	0.07985406	-268.5818	-0.2850862	0.3649402	ZMYND15
cg24812523	0.3489266	-250.0215	-0.2851123	0.6340389	AKAP6
cg09736922	0.5059608	-350.9073	-0.2851549	0.7911158	THPO
cg07115820	0.2916379	-237.154	-0.2853677	0.5770056	EPX
cg15531099	0.2508093	-233.5608	-0.2854448	0.5362542	LCE1D
cg15516226	0.5125621	-350.9073	-0.2855768	0.7981389	BTNL9
cg01772980	0.337899	-248.6564	-0.2860718	0.6239709	SCGB1D1
cg15626350	0.1813291	-238.5415	-0.2861328	0.467462	ESR1
cg27020690	0.06695075	-277.1617	-0.2862431	0.3531938	TERC
cg15303841	0.3145589	-243.6327	-0.2865506	0.6011094	RFPL1
cg06303238	0.3656256	-259.4737	-0.286608	0.6522335	SALL4
cg16272420	0.434082	-297.8051	-0.2866294	0.7207115	PNLIPRP2
cg03003745	0.3034022	-241.6061	-0.2866748	0.590077	UNQ473
cg19384697	0.213723	-236.4273	-0.2868608	0.5005838	UPK3B
cg26415633	0.1807523	-239.8395	-0.2868636	0.4676159	KLK1
cg25020204	0.2908547	-240.6405	-0.2872721	0.5781268	DBH
cg27377450	0.519381	-350.9073	-0.2874269	0.8068079	ARHGEF18
cg10787197	0.2840677	-240.321	-0.287605	0.5716727	C6orf105
cg27566805	0.2023117	-239.0446	-0.2879267	0.4902384	USH2A
cg05440289	0.4664546	-331.149	-0.2882468	0.7547014	IVL
cg12069042	0.390556	-275.2743	-0.2884296	0.6789856	PLXNB1
cg00698688	0.5154736	-350.9073	-0.2885938	0.8040674	SULT2B1
cg21450627	0.5270273	-350.9073	-0.2886314	0.8156587	PSD4
cg25400358	0.2736227	-241.99	-0.2891822	0.5628049	GPR137
cg13180098	0.3527866	-260.3751	-0.2891924	0.641979	RHO
cg07922606	0.2056721	-241.0495	-0.2892842	0.4949563	HIST1H3E
cg00626119	0.2474686	-240.3404	-0.2893179	0.5367865	NTRK1
cg05556202	0.4684285	-337.7115	-0.2895907	0.7580193	TM4SF19
cg08292050	0.4682191	-337.9574	-0.2897304	0.7579495	SOCS4
cg19859270	0.3931966	-280.0113	-0.289749	0.6829456	GPR15
cg10408410	0.4213305	-297.4216	-0.2897545	0.711085	RLBP1
cg01970325	0.2480747	-241.3465	-0.2898722	0.5379469	NELF

cg03453449	0.3249093	-253.28	-0.2900088	0.6149181	USP44
cg09076584	0.3808365	-274.5992	-0.29014	0.6709765	FLJ25006
cg15711744	0.4723061	-343.9573	-0.2902173	0.7625234	ANP32D
cg22021786	0.4209023	-299.2905	-0.2905283	0.7114305	WFDC8
cg23444894	0.4522501	-324.8721	-0.2905403	0.7427903	UNQ5810
cg15743985	0.4140081	-294.7374	-0.2905859	0.704594	CD22
cg24027679	0.4853294	-350.9073	-0.2906345	0.7759639	SLC2A7
cg16626670	0.3552076	-265.4209	-0.2909985	0.6462061	CLEC4G
cg18129786	0.5188515	-350.9073	-0.2910156	0.8098671	ZNF445
cg23749046	0.4972081	-350.9073	-0.2910703	0.7882785	GPR61
cg10052840	0.2039926	-244.3445	-0.2911325	0.4951251	SEMA6B
cg02833725	0.3612347	-268.2295	-0.291162	0.6523967	ISG20L2
cg05912121	0.3687834	-271.5685	-0.2912189	0.6600023	TH
cg04143809	0.2804507	-247.075	-0.2914015	0.5718522	FLJ39822
cg01550148	0.2560095	-244.6052	-0.2914217	0.5474311	H2AFY
cg04574507	0.3574342	-267.4485	-0.2914923	0.6489264	CD1B
cg26661623	0.3419518	-262.7482	-0.2919578	0.6339096	ASGR2
cg04962134	0.3080791	-253.178	-0.291962	0.600041	TRIM51
cg19728577	0.4806361	-350.9073	-0.2923201	0.7729563	GUCA2B
cg27622610	0.2161805	-245.7353	-0.2923542	0.5085348	OR1G1
cg04968473	0.2654002	-247.2142	-0.2923996	0.5577998	CYP1A2
cg19033555	0.2939121	-251.3253	-0.2924768	0.5863889	DEFB1
cg01484156	0.2572935	-247.3215	-0.2928312	0.5501247	NCALD
cg21755709	0.1842031	-249.3461	-0.2928679	0.477071	C21orf124
cg26705561	0.2495855	-246.9197	-0.2928729	0.5424584	SEC31L2
cg13259290	0.4327754	-315.3253	-0.2929085	0.7256839	CSF2
cg04953015	0.420161	-307.3509	-0.2935182	0.7136792	CHRNA2
cg17926869	0.4777485	-350.9073	-0.2935692	0.7713177	LOC115098
cg25982743	0.1427233	-259.6999	-0.2942917	0.437015	TIMP4
cg19047670	0.374309	-281.7563	-0.2943565	0.6686655	CCND1
cg18508525	0.5172511	-350.9073	-0.2945014	0.8117525	CD36
cg18085517	0.2487947	-250.1373	-0.2946476	0.5434423	TRPM1
cg10071275	0.2858675	-254.3104	-0.2946731	0.5805405	MYT1
cg12775613	0.3344328	-266.5016	-0.2947753	0.6292081	HTR1F
cg16504670	0.4103634	-304.5279	-0.2949947	0.7053581	FLJ20186
cg09467501	0.07688189	-285.8539	-0.2952313	0.3721131	PYY
cg20011352	0.05912001	-295.6634	-0.2953026	0.3544226	GPR124
cg12113132	0.252102	-251.9261	-0.2954866	0.5475886	CCNDBP1
cg14287742	0.4542493	-343.1444	-0.2955471	0.7497964	BLZF1
cg11158374	0.4094526	-305.6733	-0.2956172	0.7050698	TFF2
cg18138484	0.5300097	-350.9073	-0.2958149	0.8258247	CABP2
cg27117399	0.2985212	-259.9629	-0.2962587	0.5947799	CNDP1
cg12951282	0.3137093	-264.6352	-0.2967355	0.6104448	ASGR2
cg02717866	0.3545996	-278.813	-0.2968399	0.6514395	FLJ32771

cg12456510	0.382686	-292.5274	-0.2968769	0.6795629	TFF2
cg01530101	0.3310097	-270.3274	-0.2969737	0.6279835	KCNQ1DN
cg16051685	0.4380363	-333.0358	-0.2971882	0.7352245	TRIM63
cg24363955	0.4883226	-350.9073	-0.2972429	0.7855654	FLJ14054
cg12351042	0.3505441	-278.696	-0.2974981	0.6480422	OR2B2
cg13694749	0.3869657	-296.7716	-0.2975917	0.6845574	SCN4A
cg15842430	0.3302141	-271.5305	-0.297644	0.627858	FAM12B
cg08970446	0.5236402	-350.9073	-0.2989486	0.8225887	SLC1A7
cg03468463	0.3467314	-280.8425	-0.2990698	0.6458012	SERPINB1 2
cg01015871	0.3175783	-270.9826	-0.2991636	0.6167418	MT4
cg13656062	0.4526428	-350.9073	-0.2994549	0.7520977	CYP4F2
cg13578652	0.3355313	-277.8089	-0.2995983	0.6351296	UBASH3A
cg27418851	0.3594388	-288.0722	-0.2997716	0.6592104	MBL2
cg24607398	0.2219442	-259.2102	-0.2998751	0.5218193	MLH1
cg16986846	0.2925137	-266.4167	-0.299958	0.5924717	SCGB2A1
cg08244028	0.3530317	-285.7881	-0.3000245	0.6530562	MSH3
cg12781568	0.2568875	-261.1892	-0.3000795	0.556967	WT1
cg21432842	0.2381101	-260.0525	-0.3001772	0.5382873	CSF3
cg01053621	0.2676869	-262.8608	-0.3002947	0.5679817	APOA2
cg07408456	0.3061845	-270.7377	-0.3004782	0.6066627	PGLYRP2
cg26581729	0.1521072	-267.996	-0.3005201	0.4526273	NPDC1
cg12970081	0.2746971	-265.313	-0.3010011	0.5756982	GPR32
cg02844051	0.4350179	-343.1784	-0.3012167	0.7362346	ZD52F10
cg19111262	0.2222512	-262.6631	-0.3017299	0.5239811	IGSF9
cg10322876	0.3216799	-278.1111	-0.3018012	0.6234811	CYP2B6
cg09748975	0.4408863	-350.9073	-0.3018464	0.7427327	MSX1
cg01785568	0.2484402	-264.5965	-0.3021875	0.5506277	MSX1
cg03364781	0.3789587	-304.4191	-0.3022035	0.6811622	ALPK1
cg07792737	0.5098905	-350.9073	-0.3024258	0.8123163	NPIP
cg20311730	0.3251326	-281.828	-0.3029417	0.6280743	NALP10
cg22510822	0.340349	-289.7312	-0.3038667	0.6442158	OR1E2
cg18063149	0.3862935	-313.7604	-0.3039673	0.6902608	FMO3
cg23881725	0.1256265	-280.8073	-0.3040572	0.4296838	DLEC1
cg27090216	0.05394081	-312.9915	-0.3041496	0.3580904	TNFRSF10 C
cg10575735	0.29611	-277.2615	-0.3046739	0.6007839	SSX4
cg09299388	0.3922968	-320.0313	-0.3047906	0.6970874	PGK2
cg20891917	0.4176386	-339.3335	-0.3048395	0.7224781	IFRD1
cg24840099	0.2559622	-270.7978	-0.3049589	0.5609211	MSX1
cg22190114	0.4102363	-334.2178	-0.3051451	0.7153814	NALP8
cg00594952	0.259784	-271.632	-0.3051679	0.564952	RIMS3
cg24407065	0.4979711	-350.9073	-0.3052947	0.8032658	BLZF1
cg20189782	0.4040549	-331.0451	-0.305685	0.7097399	MGC2712 1

cg07643942	0.2563702	-272.4211	-0.3057549	0.5621251	LACRT
cg14182690	0.484645	-350.9073	-0.3062514	0.7908964	RUNX3
cg14238120	0.4063127	-334.9023	-0.306387	0.7126997	ELA3A
cg07339138	0.2668455	-275.3521	-0.3065077	0.5733532	CCDC13
cg27235662	0.4503666	-350.9073	-0.3067638	0.7571304	CLDN16
cg24735489	0.3173786	-288.8555	-0.3071072	0.6244857	CDSN
cg27442349	0.3260117	-292.1113	-0.307193	0.6332046	NFKBIB
cg24851490	0.1967697	-274.1461	-0.3074771	0.5042468	RNASE2
cg25957124	0.2195763	-273.8479	-0.3076426	0.5272189	DNAH3
cg01726767	0.3106277	-288.4838	-0.3078944	0.6185221	LALBA
cg15210427	0.2066712	-274.9658	-0.3081611	0.5148324	CST9L
cg10569414	0.3812778	-322.3452	-0.308202	0.6894798	C21orf121
cg01074640	0.3383363	-300.5196	-0.3086507	0.6469871	IFNA17
cg10127415	0.21716	-276.6231	-0.3090895	0.5262495	MAGEB6
cg19481686	0.3207112	-294.7374	-0.3091382	0.6298494	CDKN2B
cg02324920	0.2160088	-276.7488	-0.3091651	0.5251739	NEURL
cg05215575	0.2558413	-279.3724	-0.3092026	0.5650438	FLJ25410
cg23829949	0.2722019	-281.9062	-0.3092216	0.5814235	ZNF238
cg14179628	0.3297285	-298.3348	-0.3092244	0.6389529	TCEAL7
cg05636175	0.1209033	-291.1786	-0.3092464	0.4301497	TNFRSF10C
cg02130905	0.2974809	-287.8697	-0.3093105	0.6067913	STMN4
cg24852661	0.08091807	-307.811	-0.3098652	0.3907833	GOLPH2
cg01982597	0.37272	-321.6963	-0.309887	0.682607	PGBD3
cg08684473	0.4381951	-350.9073	-0.3101882	0.7483833	LILRB5
cg22784047	0.3764021	-324.8721	-0.3101959	0.6865979	MVP
cg09300114	0.1562128	-284.6454	-0.3105192	0.466732	SLC16A5
cg05670596	0.1772051	-281.6657	-0.310611	0.4878161	CCRL2
cg10677144	0.4005408	-343.5021	-0.3107069	0.7112477	MYOM1
cg22294577	0.4933836	-350.9073	-0.310992	0.8043756	SLC26A3
cg24992780	0.4875124	-350.9073	-0.3111588	0.7986712	OR7C1
cg05348870	0.4582027	-350.9073	-0.3111937	0.7693964	TNFSF14
cg16122592	0.3292902	-303.1908	-0.3112646	0.6405548	MAGEB6
cg02218324	0.3376594	-309.5093	-0.312322	0.6499814	RSHL1
cg07484827	0.1883708	-283.8648	-0.312383	0.5007538	CHRNA10
cg04457051	0.2923672	-293.7453	-0.3125455	0.6049128	SCOC
cg06501790	0.3554405	-319.175	-0.3126061	0.6680466	SLC34A1
cg25372195	0.3450688	-314.8414	-0.3130135	0.6580823	DCD
cg18242139	0.4002953	-350.8403	-0.3131154	0.7134107	ELAVL4
cg19910382	0.2821643	-293.1941	-0.3134445	0.5956088	FABP1
cg04505023	0.3862834	-341.026	-0.3134595	0.6997429	SPRR1A
cg27087809	0.4386388	-350.9073	-0.3135123	0.7521511	ACSBG1
cg19233472	0.4088135	-350.9073	-0.3135412	0.7223547	FOXI1
cg15480475	0.4080791	-350.9073	-0.3137013	0.7217804	TUB
cg01643624	0.4126795	-350.9073	-0.3139455	0.726625	C11orf16

cg24693053	0.1562141	-291.3462	-0.3141888	0.4704029	MFSD7
cg07597976	0.270934	-292.7651	-0.3143603	0.5852943	CD19
cg24901474	0.2372028	-288.1036	-0.3144175	0.5516204	RGS5
cg20576002	0.2941018	-298.8212	-0.3145735	0.6086753	FAM112B
cg01305625	0.2139752	-287.5299	-0.314688	0.5286632	PDLIM4
cg01894895	0.4634722	-350.9073	-0.3148311	0.7783033	ANXA1
cg02442161	0.4382539	-350.9073	-0.3148732	0.7531271	PI3
cg26583078	0.3460507	-320.6961	-0.3150533	0.6611041	SORBS2
cg04675937	0.3169513	-307.9467	-0.3151721	0.6321235	CDKN2B
cg05093686	0.1614378	-292.3197	-0.315203	0.4766409	MAB21L1
cg14911395	0.1556228	-293.4158	-0.3152861	0.4709089	SEMA3B
cg09037813	0.3432149	-321.1516	-0.3157725	0.6589874	LRRFIP1
cg11599505	0.4484804	-350.9073	-0.3165478	0.7650282	C20orf102
cg26149550	0.4718102	-350.9073	-0.3167361	0.7885463	KLK15
cg07525077	0.2558504	-298.0316	-0.3180567	0.5739071	RNASE3
cg02351381	0.1452575	-302.1096	-0.318937	0.4641945	C12orf34
cg18979223	0.341326	-329.5379	-0.3192753	0.6606013	CDKN2B
cg19807685	0.3556733	-339.429	-0.3199378	0.6756111	HSD17B2
cg21686987	0.456027	-350.9073	-0.3204511	0.7764781	CTRB1
cg14662756	0.3240156	-324.8897	-0.3207222	0.6447378	NPFF
cg22264436	0.4260747	-350.9073	-0.3208181	0.7468928	SOST
cg23260026	0.1853075	-300.6665	-0.3210185	0.506326	FSTL3
cg02187357	0.3137384	-321.7162	-0.321206	0.6349444	TBC1D22B
cg19917856	0.1916062	-300.7784	-0.3212496	0.5128558	LOC34289 7
cg12513481	0.2384804	-303.6669	-0.3217821	0.5602626	SCAP1
cg06244417	0.4526562	-350.9073	-0.3221298	0.774786	FCN1
cg12619162	0.446428	-350.9073	-0.3221733	0.7686013	FXYD4
cg15905124	0.4487759	-350.9073	-0.3224154	0.7711913	MGC1303 4
cg27285599	0.4382556	-350.9073	-0.3233248	0.7615805	FLJ13841
cg07412254	0.3078325	-325.5587	-0.3236655	0.631498	FLJ14816
cg21372914	0.3902878	-350.9073	-0.3237403	0.7140282	CLEC4M
cg24750391	0.1652161	-308.0969	-0.3238201	0.4890362	PON3
cg18780284	0.3570322	-350.9073	-0.3243613	0.6813936	SPRR1B
cg10275770	0.1153637	-320.4833	-0.3246911	0.4400549	ICAM2
cg15996947	0.2923377	-323.9796	-0.3253069	0.6176445	L2HGDH
cg09414535	0.2780388	-320.4035	-0.3256594	0.6036983	GRIP1
cg26063872	0.3163746	-335.3188	-0.3260295	0.6424041	DEFB123
cg00321478	0.3302248	-342.7138	-0.3262721	0.6564969	CRB1
cg13019092	0.4061252	-350.9073	-0.3266533	0.7327785	PDZK1
cg06832950	0.4493194	-350.9073	-0.3266932	0.7760125	SPG3A
cg11719283	0.3315827	-345.4343	-0.3270141	0.6585968	ZNF574
cg06220755	0.290849	-328.3398	-0.3272671	0.618116	RAI2
cg25762706	0.2417464	-316.6455	-0.3274754	0.5692218	STMN4

cg09701102	0.4253201	-350.9073	-0.3278017	0.7531219	NDUFV1
cg08972170	0.2285695	-316.4248	-0.3281359	0.5567054	Ells1
cg27257987	0.3188199	-342.2245	-0.328201	0.6470209	PSG4
cg19042947	0.3103563	-338.7437	-0.3283471	0.6387034	SERPINA4
cg19982860	0.3005309	-336.1518	-0.3289448	0.6294757	IFNA21
cg05444024	0.5208416	-350.9073	-0.3310073	0.8518489	FUT6
cg02157083	0.2985285	-343.9606	-0.3322863	0.6308148	APOA5
cg00334507	0.2916562	-342.2193	-0.3326467	0.6243029	MVP
cg09027725	0.2966346	-344.336	-0.3327345	0.6293691	COX4I2
cg04138756	0.4472772	-350.9073	-0.3333534	0.7806306	SPRR3
cg06811800	0.3417124	-350.9073	-0.3336121	0.6753246	ATP4B
cg13204181	0.2971395	-348.0213	-0.3340269	0.6311664	GH1
cg15780361	0.2165424	-329.998	-0.3348484	0.5513908	ALS2CR11
cg05766474	0.4834163	-350.9073	-0.3350658	0.818482	CCL16
cg01515887	0.3931991	-350.9073	-0.3355448	0.7287439	SAA2
cg13521229	0.2175145	-331.7588	-0.3356052	0.5531197	JOSD2
cg00756887	0.3492271	-350.9073	-0.3362339	0.685461	PVRL4
cg17820828	0.3882852	-350.9073	-0.3362552	0.7245404	KCNQ1
cg07651914	0.2355458	-338.9721	-0.3376731	0.5732188	CLDN15
cg13271951	0.1873381	-335.3835	-0.3379064	0.5252445	FAM57B
cg25203980	0.4709641	-350.9073	-0.3389154	0.8098795	CENTB5
cg17357062	0.4868338	-350.9073	-0.3389211	0.8257549	FCN1
cg07997737	0.2321734	-344.0519	-0.3401192	0.5722926	NRTN
cg14696820	0.2593903	-350.9073	-0.341559	0.6009493	LCE1A
cg04711324	0.2219131	-349.6812	-0.3431783	0.5650914	RIT2
cg14894144	0.1360703	-350.9073	-0.3433836	0.479454	LAMA3
cg25813714	0.4999365	-350.9073	-0.3436572	0.8435937	CYP4F12
cg10758292	0.4251256	-350.9073	-0.3447331	0.7698587	DEFA1
cg12334759	0.3526571	-350.9073	-0.3453131	0.6979702	C19orf19
cg06351503	0.3412167	-350.9073	-0.3454117	0.6866283	RDBP
cg16990174	0.2011383	-350.9073	-0.3463497	0.547488	RYBP
cg10990993	0.2521413	-350.9073	-0.3463918	0.5985331	MLH1
cg25214346	0.3076224	-350.9073	-0.3465436	0.654166	NR1I3
cg20373326	0.3340394	-350.9073	-0.3469601	0.6809996	HSD17B2
cg21457804	0.4035528	-350.9073	-0.3469617	0.7505146	CT45-2
cg01657380	0.3227317	-350.9073	-0.3471405	0.6698722	NPFF
cg17907567	0.2743388	-350.9073	-0.3474548	0.6217936	HAMP
cg24024214	0.4733439	-350.9073	-0.3482055	0.8215494	BTNL8
cg21948655	0.4506653	-350.9073	-0.3482083	0.7988736	SMCP
cg02067021	0.3393566	-350.9073	-0.3492232	0.6885798	DNAJC5B
cg12815142	0.3262381	-350.9073	-0.3498877	0.6761258	SPAG7
cg08448751	0.3101435	-350.9073	-0.3512988	0.6614423	SEMA3G
cg24670715	0.1467054	-350.9073	-0.3529376	0.4996431	ANGPT2
cg04323365	0.3906217	-350.9073	-0.3535424	0.744164	GJB1
cg25119415	0.3342555	-350.9073	-0.3536854	0.687941	MNDA

cg03602500	0.3769889	-350.9073	-0.3537084	0.7306974	FLJ00060
cg19954000	0.1473954	-350.9073	-0.354591	0.5019863	FGF1
cg12489960	0.3134574	-350.9073	-0.3553901	0.6688474	SGCB
cg24490338	0.363713	-350.9073	-0.3561833	0.7198963	TPM3
cg15652212	0.3361337	-350.9073	-0.3572417	0.6933754	FLJ90586
cg03221619	0.1960138	-350.9073	-0.357278	0.5532917	FCER2
cg06123346	0.2612833	-350.9073	-0.3587117	0.6199951	ATP4A
cg23412777	0.2822225	-350.9073	-0.3602996	0.6425221	PYGO1
cg19712821	0.2062546	-350.9073	-0.3605439	0.5667985	KSP37
cg15928132	0.2460896	-350.9073	-0.3614774	0.607567	CCKAR
cg16772207	0.2586677	-350.9073	-0.3615642	0.620232	MYT1
cg07824742	0.3956579	-350.9073	-0.3634132	0.7590712	DBH
cg19949550	0.373367	-350.9073	-0.3654561	0.7388231	ASB2
cg06436504	0.303599	-350.9073	-0.3660417	0.6696407	DOC1
cg10805676	0.3468397	-350.9073	-0.3664854	0.7133251	MRPL28
cg16175263	0.1958683	-350.9073	-0.3670614	0.5629296	TNFRSF10C
cg06101324	0.1277653	-350.9073	-0.3676583	0.4954236	SPRR1A
cg06639544	0.3696489	-350.9073	-0.3688664	0.7385153	OR7A5
cg06277657	0.1093963	-350.9073	-0.3701676	0.4795639	DGKI
cg10604646	0.1664207	-350.9073	-0.3707275	0.5371482	RGS5
cg20544605	0.2848389	-350.9073	-0.3728623	0.6577012	SORBS2
cg24694549	0.2671041	-350.9073	-0.3731966	0.6403007	GRIP1
cg26292028	0.3147417	-350.9073	-0.3752455	0.6899872	FLJ37587
cg14845091	0.2493326	-350.9073	-0.3753314	0.624664	ADPRHL1
cg08450982	0.2129434	-350.9073	-0.3757941	0.5887374	NUMBL
cg25221254	0.3504186	-350.9073	-0.3760255	0.7264441	ASAH3
cg15503752	0.3524603	-350.9073	-0.3772689	0.7297292	ST6GALNAC1
cg05671018	0.3016568	-350.9073	-0.3777252	0.679382	LYSMD2
cg14256699	0.4805748	-350.9073	-0.3798139	0.8603887	SOST
cg10062065	0.277668	-350.9073	-0.3806831	0.6583511	APEG1
cg22575540	0.3748259	-350.9073	-0.3810335	0.7558594	TRIM54
cg18149207	0.3478193	-350.9073	-0.3855318	0.7333511	RORC
cg26672426	0.2359512	-350.9073	-0.3902738	0.626225	PTGES
cg19728223	0.1460859	-350.9073	-0.3922928	0.5383787	KCNQ1
cg06536578	0.2557892	-350.9073	-0.3943564	0.6501456	JPH4
cg14706739	0.2978938	-350.9073	-0.3946822	0.6925761	EPB49
cg08573687	0.2859719	-350.9073	-0.3947454	0.6807172	TH
cg08626653	0.3123756	-350.9073	-0.398624	0.7109996	FLJ37538
cg23815000	0.3259055	-350.9073	-0.4012898	0.7271953	LCN1
cg01335367	0.1206263	-350.9073	-0.4027246	0.5233509	C12orf34
cg20154346	0.2338742	-350.9073	-0.4041119	0.6379861	RAI2
cg26927807	0.2863748	-350.9073	-0.4072778	0.6936526	BTBD2
cg22374142	0.08883238	-350.9073	-0.4088441	0.4976765	HSF4

cg01103730	0.2857291	-350.9073	-0.4116412	0.6973703	IL20
cg14992108	0.3064805	-350.9073	-0.4137776	0.7202581	SNTB1
cg14204735	0.05182404	-350.9073	-0.4143216	0.4661456	CYB561
cg20334738	0.2340382	-350.9073	-0.4148436	0.6488818	MAB21L2
cg04144768	0.2630639	-350.9073	-0.416039	0.6791029	DDC
cg25856811	0.2807174	-350.9073	-0.4372394	0.7179568	SPRR3
cg13726507	0.3871346	-350.9073	-0.4412836	0.8284182	CTAG2
cg27619475	0.1054726	-350.9073	-0.4510135	0.5564861	SLC16A5
cg26164184	0.3474236	-350.9073	-0.4674916	0.8149152	FCN2
cg13021192	0.1025132	-350.9073	-0.4980029	0.6005161	CTSZ
cg16179125	0.1394228	-350.9073	-0.5037773	0.6432001	CTSZ

Supplementary table 8 – Probes differentially methylated between benign and malignant tumours

TargetID	Malign.AVG_Beta	Malign.DiffScore	Malign.DeltaBeta	Benign.AVG_Beta	SYMBOL
cg15538820	0.4530806	-349.7383	-0.3560391	0.8091196	OBP2B
cg15481539	0.4360782	-349.7383	-0.3330745	0.7691528	DEFA5
cg20822628	0.5395295	-349.7383	-0.3137423	0.8532718	GATA5
cg05055150	0.415839	-349.7383	-0.2962189	0.7120579	MAG
cg05922591	0.4448559	-349.7383	-0.2940205	0.7388764	LILRB4
cg13928961	0.2845886	-349.7383	-0.2924294	0.5770181	K6IRS3
cg16155702	0.3981431	-349.7383	-0.2874181	0.6855612	FGF21
cg08424423	0.3511186	-349.7383	-0.2866129	0.6377315	CDSN
cg01144251	0.324304	-349.7383	-0.284581	0.608885	KLK9
cg20283107	0.242698	-349.7383	-0.2842894	0.5269874	FAM91A1
cg08459368	0.435635	-349.7383	-0.2836125	0.7192475	SCGB2A1
cg14942312	0.2825689	-349.7383	-0.2831784	0.5657473	GPR119
cg18790143	0.2997488	-349.7383	-0.2811847	0.5809335	OTOS
cg06270401	0.4288664	-349.7383	-0.2797524	0.7086188	DYRK4
cg06145357	0.3597003	-349.7383	-0.2782477	0.637948	MAGEA8
cg09542291	0.3586163	-349.7383	-0.2779242	0.6365405	SMCP
cg00727947	0.4717107	-349.7383	-0.2771084	0.7488191	LILRA5
cg04719766	0.3542481	-349.7383	-0.2751947	0.6294428	KCNQ1
cg05766474	0.337676	-349.7383	-0.2748767	0.6125528	CCL16
cg20095587	0.3152015	-349.7383	-0.2740147	0.5892162	TREM2
cg11154879	0.4521337	-349.7383	-0.2724403	0.724574	C20orf151
cg15589427	0.3151931	-349.7383	-0.2723064	0.5874994	MUC4
cg13916742	0.4317627	-349.7383	-0.2706729	0.7024356	SCGB1D1
cg08453096	0.381714	-349.7383	-0.2694039	0.651118	ABCG5
cg06325687	0.5240554	-349.7383	-0.2686477	0.7927031	OPN1MW
cg17264470	0.4252936	-349.7383	-0.2679611	0.6932546	FGF21

cg01442426	0.466824	-349.7383	-0.2674867	0.7343107	XCR1
cg04721098	0.378996	-349.7383	-0.2660427	0.6450387	CACNG3
cg25903122	0.4034972	-349.7383	-0.2651348	0.668632	MGC2747
cg07297178	0.3714801	-349.7383	-0.2646588	0.6361389	CEACAM7
cg04645843	0.3893785	-349.7383	-0.2640179	0.6533964	DPCR1
cg02737335	0.4061463	-349.7383	-0.2607353	0.6668816	ARHGEF1 6
cg05615150	0.3974414	-349.7383	-0.2607169	0.6581584	ARPP-21
cg06850526	0.3746495	-349.7383	-0.2598681	0.6345176	MGC1552 3
cg00078867	0.3143547	-349.7383	-0.2597524	0.5741071	WAS
cg21168884	0.4030654	-349.7383	-0.2590842	0.6621496	C6orf122
cg24841244	0.4134916	-349.7383	-0.2563933	0.6698849	CD3D
cg15210999	0.35205	-349.7383	-0.2548002	0.6068502	DTNBP1
cg18521925	0.510487	-349.7383	-0.2547985	0.7652855	SLC22A16
cg13603551	0.4619253	-349.7383	-0.2539879	0.7159132	ABP1
cg20050113	0.1920189	-349.7383	-0.2539599	0.4459788	SLC9A2
cg15379858	0.5172948	-349.7383	-0.2531638	0.7704585	ChGn
cg24777950	0.3926003	-349.7383	-0.2530188	0.6456191	CTSG
cg20649991	0.3407245	-349.7383	-0.2528833	0.5936078	LILRB5
cg04520391	0.4054843	-349.7383	-0.2516547	0.657139	PRB2
cg08970446	0.4016043	-349.7383	-0.2504309	0.6520352	SLC1A7
cg21457804	0.2417219	-349.7383	-0.2501009	0.4918227	CT45-2
cg05740244	0.2508841	-349.7383	-0.2477106	0.4985947	LDHC
cg14150666	0.4415726	-349.7383	-0.2472936	0.6888662	IL8RB
cg19304352	0.4199971	-349.7383	-0.2471404	0.6671375	DEFA4
cg10334928	0.5186656	-349.7383	-0.2469132	0.7655788	STON2
cg03886110	0.3517158	-349.7383	-0.2467308	0.5984465	PECAM1
cg05444024	0.3666006	-349.7383	-0.2456803	0.6122809	FUT6
cg00625425	0.1842059	-349.7383	-0.2456387	0.4298446	ANKRD38
cg01774645	0.2713733	-349.7383	-0.2451142	0.5164875	ARHGAP3 0
cg17298704	0.4519594	-349.7383	-0.2450196	0.696979	CLDN18
cg21505334	0.3557376	-349.7383	-0.244886	0.6006235	CEACAM5
cg00548060	0.410025	-349.7383	-0.2447128	0.6547378	NPL
cg27291231	0.3371188	-349.7383	-0.2445031	0.5816218	SSNA1
cg13453139	0.4159389	-349.7383	-0.2444932	0.6604321	PIK3R5
cg00603172	0.452023	-349.7383	-0.2434982	0.6955212	BOK
cg24310246	0.4841552	-349.7383	-0.2432622	0.7274174	RAXL1
cg17981339	0.4541289	-349.7383	-0.2430857	0.6972146	SBEM
cg16607065	0.4440067	-349.7383	-0.2429445	0.6869512	TP73
cg19764418	0.2432153	-349.7383	-0.2421881	0.4854034	RYR2
cg20485165	0.2828671	-349.7383	-0.2420703	0.5249375	WFDC12
cg22506059	0.5863695	-349.7383	-0.2420411	0.8284106	CARD10
cg16462075	0.2858849	-349.7383	-0.2408291	0.526714	MUC3B

cg10409560	0.4086342	-349.7383	-0.2408101	0.6494443	FLJ23657
cg15140807	0.3757119	-349.7383	-0.2402097	0.6159216	FLJ31222
cg09691574	0.4869934	-349.7383	-0.2395915	0.7265849	MRGPRX4
cg23776892	0.3681794	-349.7383	-0.2394203	0.6075997	MAGEA1
cg22484793	0.3971853	-349.7383	-0.2381729	0.6353582	TLR9
cg12205591	0.336417	-349.7383	-0.2381542	0.5745711	CRYAA
cg09044738	0.5255961	-349.7383	-0.2378273	0.7634234	FAM12A
cg14284171	0.4649712	-349.7383	-0.2373047	0.7022759	SSX4
cg09076077	0.3754302	-349.7383	-0.2371517	0.6125819	FLJ33860
cg27157038	0.3870067	-349.7383	-0.2367619	0.6237686	DNTT
cg22679120	0.2827845	-349.7383	-0.2365012	0.5192857	SNX8
cg05246522	0.331798	-349.7383	-0.2360892	0.5678872	KSR1
cg10746737	0.3413668	-349.7383	-0.2359594	0.5773262	HLA-DRB5
cg03712237	0.4411383	-349.7383	-0.2355894	0.6767277	SSX2
cg13861644	0.4133269	-349.7383	-0.2354456	0.6487724	PIWIL1
cg26306976	0.5653939	-349.7383	-0.235133	0.8005269	ITGB1BP1
cg15337006	0.3983825	-349.7383	-0.2348147	0.6331971	ITGAM
cg12061127	0.2759923	-349.7383	-0.233935	0.5099273	WFDC9
cg00466249	0.3795245	-349.7383	-0.2329377	0.6124622	MGC15523
cg11128808	0.4956025	-349.7383	-0.2327401	0.7283425	CACNG6
cg26862286	0.6194292	-349.7383	-0.2326031	0.8520324	NCL
cg26298099	0.5111067	-349.7383	-0.2325617	0.7436684	ESRRB
cg01718139	0.3616614	-349.7383	-0.2321473	0.5938087	UNQ3033
cg12650635	0.2440127	-349.7383	-0.2319266	0.4759393	AQP2
cg21399079	0.5492972	-349.7383	-0.2318422	0.7811394	GPR45
cg25866075	0.2677559	-349.7383	-0.2317846	0.4995404	NALP12
cg19537511	0.260428	-349.7383	-0.2316766	0.4921046	ARHGEF15
cg19845843	0.302144	-349.7383	-0.2310586	0.5332026	CXorf20
cg11346450	0.3565485	-349.7383	-0.2309327	0.5874811	UGT1A3
cg07506795	0.5561472	-349.7383	-0.2299617	0.7861089	ZNF19
cg26709720	0.3257397	-349.7383	-0.2294499	0.5551896	B3GALT5
cg18121684	0.2844862	-349.7383	-0.2279559	0.5124421	SERPINB13
cg18920397	0.3002461	-349.7383	-0.2273841	0.5276302	LY9
cg16512727	0.1761041	-349.7383	-0.2268255	0.4029295	C12orf4
cg15711744	0.3049476	-349.7383	-0.2267154	0.5316629	ANP32D
cg16545105	0.2804936	-349.7383	-0.2236802	0.5041739	CRHBP
cg03264414	0.5583675	-349.7383	-0.2230498	0.7814173	PAEP
cg17829936	0.2673488	-349.7383	-0.2218965	0.4892453	TAAR5
cg08477744	0.5888508	-349.7383	-0.2215217	0.8103725	MFAP2
cg05973262	0.2171488	-349.7383	-0.2203037	0.4374526	NOTCH4

cg20090497	0.2631778	-349.7383	-0.2195344	0.4827121	TAS2R9
cg24818418	0.6178559	-349.7383	-0.2141469	0.8320028	EGF
cg09207718	0.22655	-349.7383	-0.2124448	0.4389948	CYP1A2
cg18604842	0.6423548	-349.7383	-0.2106856	0.8530405	FLJ36046
cg16626670	0.2194524	-349.7383	-0.2104451	0.4298975	CLEC4G
cg21707816	0.2288848	-349.7383	-0.2087309	0.4376157	ENDOGL1
cg01015871	0.1881558	-349.7383	-0.2060439	0.3941997	MT4
cg22580353	0.1478067	-349.7383	-0.2012693	0.3490759	PVR
cg17907567	0.1588891	-349.7383	-0.2012304	0.3601196	HAMP
cg19787037	0.1942855	-349.7383	-0.201064	0.3953495	SPAG11
cg04740359	0.1738479	-349.7383	-0.2010541	0.374902	NTF3
cg24678320	0.6430327	-349.5606	-0.2038725	0.8469052	FLJ38451
cg21434954	0.3879972	-348.8308	-0.2321814	0.6201785	LCE1B
cg02989940	0.2647014	-348.2069	-0.2163252	0.4810266	ERAF
cg18986165	0.4912712	-347.9269	-0.2296647	0.7209359	SIGLEC12
cg04891836	0.2747343	-347.4762	-0.2181711	0.4929054	TNFSF14
cg03055440	0.3544769	-344.641	-0.2286599	0.5831368	MS4A6A
cg00057593	0.2399878	-344.3014	-0.2093579	0.4493457	GML
cg21518208	0.2945707	-343.7306	-0.2206734	0.515244	KRTHB5
cg14902389	0.3972699	-342.5865	-0.2305449	0.6278148	MGAT4A
cg22899145	0.6054332	-342.4624	-0.2108555	0.8162888	OPN1LW
cg15484375	0.3750719	-341.3942	-0.2291345	0.6042064	SAA1
cg19623751	0.269946	-341.3942	-0.2153059	0.4852519	CEACAM7
cg12133004	0.4039559	-341.3883	-0.2303247	0.6342806	C12orf54
cg19831369	0.3097072	-341.241	-0.2222748	0.5319819	HPS4
cg03364781	0.2681359	-340.8514	-0.2147378	0.4828737	ALPK1
cg24857545	0.3572976	-340.1646	-0.2274397	0.5847373	GDI1
cg12456510	0.2358742	-339.2136	-0.2067161	0.4425902	TFF2
cg07126559	0.3519461	-337.3503	-0.2260688	0.5780149	SGCG
cg04816348	0.4607313	-336.657	-0.2281395	0.6888708	CLEC4G
cg15975283	0.4293278	-335.5066	-0.2286265	0.6579543	SLC9A11
cg02274362	0.5831261	-333.8466	-0.212859	0.7959852	PNMA6A
cg03386373	0.2626052	-332.0456	-0.210797	0.4734021	SPATA3
cg17170504	0.3303871	-332.027	-0.2220818	0.5524689	HSPC065
cg10370591	0.2568521	-331.9665	-0.2094974	0.4663496	TPO
cg23081213	0.3837045	-330.4482	-0.2260368	0.6097413	PRKAG3
cg21148892	0.3727474	-330.3783	-0.2253898	0.5981373	CLEC4F
cg14607011	0.5765337	-329.7861	-0.2128528	0.7893865	C10orf12 5
cg10503138	0.4134004	-329.2079	-0.2265394	0.6399398	CNTN4
cg01581111	0.429196	-329.1739	-0.2265616	0.6557575	RBM18
cg19859270	0.2742115	-329.1666	-0.2122381	0.4864496	GPR15
cg08996986	0.3119959	-328.8183	-0.2185786	0.5305745	EPS8L1
cg23131950	0.3497453	-328.6273	-0.2229941	0.5727394	AP2S1
cg21522797	0.5678004	-327.1663	-0.2135746	0.7813751	PLCG2

cg27513764	0.3042786	-326.5733	-0.216692	0.5209706	EFCAB3
cg08471713	0.4603995	-325.2466	-0.2244726	0.684872	MEOX1
cg13181284	0.5355783	-324.9248	-0.2177263	0.7533046	KRT6B
cg20312687	0.2909363	-324.4077	-0.2137966	0.504733	DEFB118
cg10784090	0.4992883	-323.5112	-0.2213007	0.720589	CLDN18
cg13733733	0.2635436	-323.5112	-0.2082248	0.4717684	LILRA3
cg16483466	0.4053316	-323.2937	-0.2244387	0.6297703	C20orf18 6
cg23587532	0.3188322	-322.9373	-0.2175964	0.5364285	BM88
cg06177698	0.2434229	-322.8376	-0.203427	0.4468499	GABRQ
cg04484789	0.4384841	-321.3296	-0.2238625	0.6623466	KRT25B
cg19561774	0.450701	-321.3296	-0.223549	0.67425	SLC22A2
cg09120035	0.3541225	-321.3296	-0.2209692	0.5750917	CYP11B1
cg06501790	0.2594283	-321.1715	-0.206564	0.4659923	SLC34A1
cg21307628	0.4280135	-320.6594	-0.2237497	0.6517632	URB
cg14333565	0.357435	-319.9149	-0.2207766	0.5782117	NRTN
cg15485859	0.4345184	-319.4134	-0.2232827	0.6578011	C1orf116
cg21130124	0.5627694	-319.1251	-0.2119399	0.7747093	CALML5
cg06094150	0.5004902	-319.0854	-0.2197497	0.72024	MT1B
cg16046376	0.2592078	-317.2285	-0.2052414	0.4644492	PC
cg02981853	0.5137917	-316.7817	-0.2177206	0.7315123	ARHGEF1 8
cg10129493	0.4263587	-316.7741	-0.2224665	0.6488252	CD33
cg03684977	0.4428008	-316.5683	-0.2221886	0.6649894	GRB7
cg21130374	0.258088	-316.1543	-0.2046324	0.4627204	MX2
cg10742801	0.2622358	-315.1691	-0.2052117	0.4674475	PRAP1
cg12514506	0.591264	-313.3204	-0.2051764	0.7964404	OSBPL5
cg20751395	0.5783061	-313.1685	-0.2075026	0.7858087	KCNQ1
cg08244028	0.2741447	-312.8005	-0.2068727	0.4810175	MSH3
cg18884741	0.5939192	-311.8996	-0.2042289	0.7981482	RABGEF1
cg03309967	0.2776801	-311.5582	-0.2071355	0.4848156	PSENEN
cg00953256	0.4207458	-308.8552	-0.2198225	0.6405683	CCND1
cg25221254	0.2509786	-308.5319	-0.2005752	0.4515538	ASAH3
cg24919884	0.3008333	-307.7591	-0.2099082	0.5107415	ARHGEF1 6
cg27606341	0.5621977	-307.6039	-0.2084707	0.7706684	FYB
cg21003606	0.5454174	-307.2675	-0.2108361	0.7562535	CALN1
cg08458487	0.5076604	-306.413	-0.2149904	0.7226508	SFTPD
cg11201288	0.4703562	-306.0389	-0.2176539	0.6880101	SFRS11
cg08475088	0.4432616	-304.74	-0.2182431	0.6615047	NALP9
cg26976437	0.317456	-304.1469	-0.2111019	0.528558	LY6K
cg10318258	0.4874021	-300.7382	-0.2148304	0.7022325	RIPK3
cg04837071	0.361176	-300.2626	-0.2144368	0.5756128	NOXA1
cg11783497	0.5443475	-300.2606	-0.2087535	0.753101	IL1RN
cg00075967	0.3638342	-300.2089	-0.2146123	0.5784466	STRA6

cg12971694	0.3941404	-299.7361	-0.2161438	0.6102842	CD72
cg09750183	0.5904374	-299.6744	-0.2011615	0.7915989	PGA5
cg06952310	0.2969466	-299.6476	-0.2065254	0.5034719	CSPG3
cg04541607	0.5334575	-298.9429	-0.2097148	0.7431723	CRYBB1
cg04464446	0.573222	-298.9429	-0.2039574	0.7771794	GAL
cg03860768	0.3169232	-297.5752	-0.2087809	0.5257041	BLK
cg24765446	0.3430396	-297.2302	-0.2117408	0.5547804	WFDC6
cg15815843	0.5226995	-296.9675	-0.2103178	0.7330173	MFAP5
cg27050793	0.5784721	-296.8872	-0.2024092	0.7808813	SBSN
cg05564251	0.538348	-295.4459	-0.2079552	0.7463033	SP140
cg26014197	0.4038215	-294.8189	-0.2147328	0.6185544	ZNF206
cg15166089	0.4638107	-292.4911	-0.2133938	0.6772044	CD164L2
cg23580945	0.3838131	-292.4297	-0.2131484	0.5969615	FLJ43826
cg12582965	0.4902818	-292.1792	-0.2117196	0.7020013	ATP10A
cg17405586	0.330025	-292.0819	-0.2085265	0.5385516	KRT1
cg02818322	0.3541399	-291.4603	-0.2107684	0.5649083	MAGEC3
cg06403553	0.4874102	-291.3066	-0.2116207	0.6990309	PGK2
cg02022375	0.3740108	-288.9755	-0.2113789	0.5853897	KRTAP1-1
cg25177139	0.4601569	-288.7032	-0.2122241	0.672381	SLC10A6
cg05800321	0.5403467	-288.3283	-0.2053615	0.7457083	LY6D
cg13397379	0.2970143	-287.9737	-0.2025071	0.4995214	OR2C3
cg21495715	0.3301479	-287.4554	-0.2069093	0.5370572	SLC5A10
cg26813458	0.5464146	-287.0046	-0.2041164	0.750531	CEACAM6
cg13897627	0.4303222	-286.9776	-0.2122552	0.6425774	FLJ44674
cg22021786	0.3278267	-285.2997	-0.2058706	0.5336974	WFDC8
cg13601079	0.4812854	-284.8631	-0.209809	0.6910943	SSX3
cg22229142	0.4435449	-284.459	-0.2112188	0.6547636	KCTD17
cg27212977	0.52693	-282.7381	-0.2051004	0.7320303	DEFA6
cg06980460	0.4585793	-282.3979	-0.2100728	0.6686521	FOLH1
cg07093661	0.5329809	-281.9774	-0.2041374	0.7371182	DHDH
cg12417466	0.4518083	-280.1945	-0.2095094	0.6613178	ARPP-21
cg13446199	0.3121101	-280.1945	-0.2020141	0.5141242	PSCA
cg24908058	0.5238456	-280.0073	-0.2044961	0.7283417	CGB5
cg05348870	0.3242536	-279.6946	-0.2034317	0.5276853	TNFSF14
cg26149550	0.3630838	-279.0892	-0.2070817	0.5701655	KLK15
cg01309153	0.5129861	-278.8297	-0.2051974	0.7181835	SURF1
cg18809535	0.5157196	-278.5172	-0.2048227	0.7205423	LDHAL6B
cg25033144	0.3479971	-278.1779	-0.2054685	0.5534655	FLJ00060
cg27625732	0.5199867	-277.7074	-0.2041094	0.7240961	TBC1D13
cg15743985	0.3192534	-277.6357	-0.2020497	0.5213031	CD22
cg26093687	0.43224	-277.6282	-0.2089128	0.6411529	EIF3S2
cg26264314	0.377984	-277.519	-0.2074861	0.5854701	NALP5
cg06417962	0.3780621	-277.2513	-0.2073928	0.5854549	RNH1
cg08256781	0.4104481	-277.225	-0.2086169	0.619065	ACSBG2
cg00698688	0.3552629	-276.338	-0.2054297	0.5606926	SULT2B1

cg11819637	0.3416641	-275.642	-0.2039127	0.5455768	THPO
cg19728577	0.3651664	-275.483	-0.2059037	0.5710701	GUCA2B
cg17741572	0.3925749	-273.2783	-0.2066652	0.5992401	CFB
cg13002506	0.4011521	-273.1196	-0.2069077	0.6080598	GDPD4
cg10612997	0.4086127	-273.0855	-0.2070847	0.6156974	GREB1
cg11884243	0.4263784	-273.0281	-0.2072618	0.6336402	FCN2
cg12432709	0.4298342	-272.5995	-0.2070925	0.6369268	CCDC68
cg14120879	0.4472553	-271.1126	-0.2063375	0.6535928	DEFB105 A
cg02844051	0.3449644	-270.7769	-0.2024567	0.5474211	ZD52F10
cg11733245	0.4270356	-270.5407	-0.2063402	0.6333758	IL2RA
cg04431776	0.4440412	-270.3004	-0.2060949	0.6501361	GAGE2
cg10213812	0.4825747	-270.269	-0.2045394	0.6871142	FOXN1
cg01447817	0.4972509	-268.024	-0.2027074	0.6999583	AQP12A
cg21686987	0.3583098	-267.3481	-0.2023622	0.560672	CTRB1
cg08368934	0.3648643	-264.7707	-0.201911	0.5667753	GPR97
cg24579667	0.4706296	-263.4147	-0.2026892	0.6733188	GPD2
cg25548825	0.4999415	-262.9417	-0.200671	0.7006124	TBX10
cg01999333	0.3696276	-259.8452	-0.2004208	0.5700484	CASP14
cg21747271	0.3952332	-257.6616	-0.2009564	0.5961896	AIP
cg21094154	0.4152481	-257.5285	-0.2014309	0.616679	TNFSF11
cg01214847	0.4192866	-257.3025	-0.2013966	0.6206832	TMPRSS3
cg24027679	0.3988175	-256.064	-0.200469	0.5992865	SLC2A7
cg02806777	0.6353467	349.7383	0.2757326	0.3596141	PGLYRP1
cg18403361	0.6757336	349.7383	0.2671745	0.4085591	CLEC14A
cg18741908	0.5640532	349.7383	0.2612827	0.3027706	GPR160
cg22980351	0.5754073	349.7383	0.2394026	0.3360047	WDR40B
cg12556134	0.5137188	349.7383	0.2302909	0.2834279	TGIF2
cg20973210	0.4659333	349.7383	0.2237841	0.2421491	C19orf35
cg08831348	0.6368999	349.7383	0.2163504	0.4205496	EML2
cg22740835	0.6921056	349.7383	0.2149439	0.4771617	DDR2
cg18691434	0.2879029	349.7383	0.2135389	0.07436401	GPC2
cg15127733	0.6851814	349.7383	0.2134855	0.4716959	HSPA12B
cg18342279	0.4330666	349.7383	0.2104813	0.2225853	ZAR1
cg17200465	0.4617882	349.7383	0.2052948	0.2564934	ENTPD3
cg03096975	0.5436484	349.7383	0.2013325	0.3423159	EML2
cg25947945	0.5703273	349.7383	0.2006639	0.3696634	LAD1

Supplementary Table 9 – Gene ontology enrichment analysis of genes differentially methylated between benign and malignant tumours

GO biological	Homo sapiens	upload	upload_1	upload_1	upload_1	upload_

process complete	- REFLIST (20972)	_1 (257)	(expected)	(over/under)	(fold Enrichment)	1 (P-value)
immune response (GO:0006955)	1105	33	13.54	+	2.44	2.06E-02
defense response (GO:0006952)	1236	36	15.15	+	2.38	1.21E-02
immune system process (GO:0002376)	2013	55	24.67	+	2.23	1.05E-04
Unclassified (UNCLASSIFIED)	4069	29	49.86	-	0.58	0.00E+00

Suppelementary table 10. Probes differentially methylated between benign and malignant tumours within cluster A.

TargetID	A_malignant.AVG_Beta	A_malignant.DiffScore	A_malignant.Delta Beta	A_benign.AVG_Beta	SYMBOL
cg04675937	0.517006	350.6937	0.2000547	0.3169513	CDKN2B
cg01429391	0.6685527	350.6937	0.2116865	0.4568662	MYH7
cg07640473	0.4477181	350.6937	0.2127852	0.2349329	SEMA3F
cg22740835	0.6921056	350.6937	0.2144682	0.4776374	DDR2
cg10150813	0.5208944	350.6937	0.2155135	0.3053809	KIAA0746
cg04907257	0.5916469	350.6937	0.2173524	0.3742945	ADCY2
cg18691434	0.2879029	350.6937	0.2211446	0.06675828	GPC2
cg15679651	0.4293491	350.6937	0.2212728	0.2080763	MAP4K1
cg09748975	0.6639689	350.6937	0.2230826	0.4408863	MSX1
cg18342279	0.4330666	350.6937	0.2262887	0.2067779	ZAR1
cg15127733	0.6851814	350.6937	0.2267572	0.4584242	HSPA12B
cg09076584	0.6081214	350.6937	0.2272849	0.3808365	FLJ25006
cg13140267	0.3546688	350.6937	0.22782	0.1268488	ASCC3L1
cg25947945	0.5703273	350.6937	0.2324568	0.3378705	LAD1
cg22980351	0.5754073	350.6937	0.2376334	0.3377739	WDR40B
cg17200465	0.4617882	350.6937	0.2511543	0.2106339	ENTPD3
cg20973210	0.4659333	350.6937	0.2645214	0.2014118	C19orf35
cg18741908	0.5640532	350.6937	0.2756845	0.2883687	GPR160
cg02806777	0.6353467	350.6937	0.2779162	0.3574305	PGLYRP1
cg18403361	0.6757336	350.6937	0.3042796	0.371454	CLEC14A

cg12556134	0.5137188	350.6937	0.307425	0.2062938	TGIF2
cg24841244	0.4134916	-224.3254	-0.202677	0.6161686	CD3D
cg09076077	0.3754302	-227.381	-0.2034266	0.5788568	FLJ33860
cg21434954	0.3879972	-228.1575	-0.2041184	0.5921156	LCE1B
cg17298704	0.4519594	-228.5838	-0.2033729	0.6553323	CLDN18
cg20095587	0.3152015	-230.1237	-0.2003224	0.5155239	TREM2
cg16607065	0.4440067	-230.8924	-0.2046699	0.6486766	TP73
cg24579667	0.4706296	-231.7224	-0.2035277	0.6741573	GPD2
cg24777950	0.3926003	-234.515	-0.206831	0.5994313	CTSG
cg05246522	0.331798	-235.1777	-0.2040357	0.5358337	KSR1
cg27625732	0.5199867	-237.0952	-0.2008772	0.7208639	TBC1D13
cg05615150	0.3974414	-237.8385	-0.2082377	0.6056792	ARPP-21
cg06094150	0.5004902	-238.0364	-0.2034292	0.7039195	MT1B
cg09835085	0.3697833	-238.9034	-0.207994	0.5777772	KCNE4
cg15484375	0.3750719	-239.7562	-0.2085393	0.5836112	SAA1
cg17264470	0.4252936	-239.9491	-0.2089126	0.6342062	FGF21
cg11201288	0.4703562	-241.7993	-0.2075065	0.6778627	SFRS11
cg23081213	0.3837045	-252.0713	-0.2136798	0.5973843	PRKAG3
cg14902389	0.3972699	-253.4393	-0.2144156	0.6116855	MGAT4A
cg00603172	0.452023	-253.4978	-0.2131281	0.6651511	BOK
cg27063986	0.2507764	-253.8432	-0.200205	0.4509814	NDST4
cg00727947	0.4717107	-256.4438	-0.2129341	0.6846448	LILRA5
cg07388493	0.404849	-257.0906	-0.2158484	0.6206974	NDUFS5
cg26264314	0.377984	-257.3989	-0.2156184	0.5936025	NALP5
cg23131950	0.3497453	-257.6297	-0.2144027	0.564148	AP2S1
cg04837071	0.361176	-259.7419	-0.2158619	0.5770379	NOXA1
cg15975283	0.4293278	-260.0525	-0.2165903	0.6459181	SLC9A11
cg07506795	0.5561472	-260.4547	-0.2041111	0.7602583	ZNF19
cg00078867	0.3143547	-260.7348	-0.2125722	0.5269269	WAS
cg01309153	0.5129861	-260.7825	-0.2104546	0.7234406	SURF1
cg17981339	0.4541289	-260.9011	-0.215826	0.6699549	SBEM
cg22484793	0.3971853	-262.4735	-0.2179322	0.6151174	TLR9
cg08424423	0.3511186	-265.0195	-0.2173698	0.5684884	CDSN
cg10334928	0.5186656	-265.0857	-0.2112836	0.7299492	STON2
cg09871315	0.472488	-265.0876	-0.2161156	0.6886036	HOXA2
cg26306976	0.5653939	-265.5616	-0.2042983	0.7696922	ITGB1BP1
cg18790143	0.2997488	-267.7996	-0.2135905	0.5133393	OTOS
cg10742801	0.2622358	-268.4951	-0.2080587	0.4702945	PRAP1
cg06952310	0.2969466	-268.834	-0.2136292	0.5105758	CSPG3

cg10784090	0.4992883	-269.2437	-0.215082	0.7143703	CLDN18
cg14607011	0.5765337	-273.6078	-0.2049018	0.7814355	C10orf125
cg04719766	0.3542481	-274.264	-0.2210779	0.575326	KCNQ1
cg04464446	0.573222	-278.3966	-0.20709	0.780312	GAL
cg03684977	0.4428008	-278.3985	-0.2228622	0.665663	GRB7
cg18087477	0.2709468	-278.7548	-0.2134567	0.4844035	SYCP1
cg16155702	0.3981431	-281.3978	-0.2250127	0.6231559	FGF21
cg09044738	0.5255961	-286.2923	-0.2176099	0.743206	FAM12A
cg15379858	0.5172948	-286.5752	-0.2188576	0.7361524	ChGn
cg20782689	0.5919561	-286.8934	-0.2060337	0.7979898	OXA1L
cg02173484	0.2551284	-287.7839	-0.2139142	0.4690427	GJA5
cg09542291	0.3586163	-289.4568	-0.2269304	0.5855467	SMCP
cg22467567	0.2708923	-292.6509	-0.2185474	0.4894396	IGFBP5
cg08453096	0.381714	-293.2885	-0.2291326	0.6108466	ABCG5
cg07047653	0.5821059	-294.1888	-0.2103567	0.7924626	AQP2
cg22229142	0.4435449	-294.419	-0.228505	0.6720499	KCTD17
cg23587532	0.3188322	-299.0298	-0.2273598	0.5461919	BM88
cg20649991	0.3407245	-299.9613	-0.2296096	0.5703341	LILRB5
cg08477744	0.5888508	-300.1096	-0.2107619	0.7996127	MFAP2
cg23878206	0.6048216	-300.123	-0.207229	0.8120506	ATF5
cg26976437	0.317456	-301.7808	-0.2282211	0.5456772	LY6K
cg25903122	0.4034972	-303.6787	-0.2329513	0.6364485	MGC2747
cg03264414	0.5583675	-304.0731	-0.2180388	0.7764063	PAEP
cg06270401	0.4288664	-307.7151	-0.2337492	0.6626156	DYRK4
cg17536532	0.225608	-308.0916	-0.2147324	0.4403405	KIAA0649
cg02274362	0.5831261	-309.3057	-0.2147608	0.797887	PNMA6A
cg13916742	0.4317627	-310.9698	-0.2347448	0.6665075	SCGB1D1
cg04721098	0.378996	-313.6501	-0.2362232	0.6152191	CACNG3
cg26298099	0.5111067	-315.6582	-0.2291945	0.7403012	ESRRB
cg01442426	0.466824	-315.6813	-0.2340792	0.7009032	XCR1
cg19831369	0.3097072	-317.3199	-0.2328371	0.5425442	HPS4
cg06325687	0.5240554	-317.717	-0.2280164	0.7520718	OPN1MW
cg02981853	0.5137917	-327.3333	-0.2324887	0.7462804	ARHGEF18
cg24310246	0.4841552	-332.0498	-0.2376408	0.721796	RAXL1
cg04645843	0.3893785	-335.3449	-0.2436407	0.6330192	DPCR1
cg08459368	0.435635	-335.3547	-0.242586	0.678221	SCGB2A1

cg13603551	0.4619253	-344.2523	-0.2435766	0.7055019	ABP1
cg16512727	0.1761041	-345.6162	-0.2129028	0.3890068	C12orf4
cg03198372	0.1881589	-348.1959	-0.2176657	0.4058246	KREMEN1
cg20283107	0.242698	-348.3892	-0.3082423	0.5509402	FAM91A1
cg15538820	0.4530806	-348.3892	-0.3053188	0.7583994	OBP2B
cg15481539	0.4360782	-348.3892	-0.296026	0.7321042	DEFA5
cg06850526	0.3746495	-348.3892	-0.2896782	0.6643277	MGC15523
cg05055150	0.415839	-348.3892	-0.2823841	0.6982231	MAG
cg20822628	0.5395295	-348.3892	-0.281748	0.8212775	GATA5
cg11154879	0.4521337	-348.3892	-0.2672881	0.7194219	C20orf151
cg06145357	0.3597003	-348.3892	-0.2649736	0.624674	MAGEA8
cg00466249	0.3795245	-348.3892	-0.2647276	0.6442521	MGC15523
cg21747271	0.3952332	-348.3892	-0.2574412	0.6526744	AIP
cg14942312	0.2825689	-348.3892	-0.2553415	0.5379104	GPR119
cg11783497	0.5443475	-348.3892	-0.2546123	0.7989599	IL1RN
cg01144251	0.324304	-348.3892	-0.2513844	0.5756884	KLK9
cg13453139	0.4159389	-348.3892	-0.2506206	0.6665596	PIK3R5
cg20050113	0.1920189	-348.3892	-0.2492462	0.4412651	SLC9A2
cg22679120	0.2827845	-348.3892	-0.2485805	0.531365	SNX8
cg05922591	0.4448559	-348.3892	-0.2482746	0.6931306	LILRB4
cg05740244	0.2508841	-348.3892	-0.2473995	0.4982836	LDHC
cg19764418	0.2432153	-348.3892	-0.2367249	0.4799402	RYR2
cg22506059	0.5863695	-348.3892	-0.2309128	0.8172823	CARD10
cg26862286	0.6194292	-348.3892	-0.221701	0.8411303	NCL

Suppelementary table 11. Probes differentially methylated between *NF1*-mutated tumours and normal tissue

TargetID	NF1.AVG_Beta	NF1.DiffScore	NF1.Delta Beta	normal.AVG_Beta	SYMBOL
cg09871315	0.6965898	348.7749	0.3912158	0.305374	HOXA2
cg24101578	0.6988041	348.7749	0.3590902	0.3397138	CDH22
cg16517394	0.5235299	348.7749	0.3187992	0.2047307	TNFSF4
cg14371590	0.6236935	348.7749	0.3119166	0.3117769	SLC26A10
cg15958424	0.574763	348.7749	0.3062523	0.2685107	ACPP

cg17686260	0.8032176	348.7749	0.3042283	0.4989893	MGMT
cg07285276	0.821921	348.7749	0.2998421	0.5220789	RAPGEF1
cg17339202	0.5737547	348.7749	0.2956863	0.2780684	SYNC1
cg03752628	0.5343904	348.7749	0.2929518	0.2414387	PTGFRN
cg19125606	0.6374627	348.7749	0.2884381	0.3490245	AGT
cg02506908	0.7068832	348.7749	0.2843943	0.4224889	HPD
cg07047653	0.8414207	348.7749	0.2832409	0.5581798	AQP2
cg01103836	0.9511703	348.7749	0.2762121	0.6749582	MYO9B
cg04802221	0.6943331	348.7749	0.2758361	0.418497	LOC283849
cg14409083	0.5139847	348.7749	0.2754085	0.2385762	EMP1
cg17890764	0.6807331	348.7749	0.2748784	0.4058547	ITIH4
cg07236190	0.7600912	348.7749	0.2744851	0.4856061	AMDHD1
cg17791651	0.5707121	348.7749	0.2729125	0.2977996	POU3F1
cg17105014	0.4479879	348.7749	0.2697038	0.1782841	GYPC
cg18702197	0.6343318	348.7749	0.2681271	0.3662047	HOXD3
cg17790333	0.7589638	348.7749	0.2663354	0.4926285	CYP11A1
cg03271907	0.8519585	348.7749	0.2633016	0.5886568	MGMT
cg11554605	0.7766601	348.7749	0.2588209	0.5178393	ASB4
cg24134767	0.6884218	348.7749	0.257864	0.4305578	HTR3A
cg12564453	0.7913745	348.7749	0.2577252	0.5336493	CETP
cg22740835	0.5705751	348.7749	0.2563796	0.3141955	DDR2
cg26668713	0.4026574	348.7749	0.2551222	0.1475352	SIPA1
cg08624249	0.7327272	348.7749	0.2536971	0.4790302	KIAA0889

cg18172186	0.772864	348.7749	0.2523714	0.5204926	KIAA1913
cg24459563	0.7360434	348.7749	0.252001	0.4840423	CACNG1
cg02674804	0.8801907	348.7749	0.2506418	0.6295489	REEP6
cg10861599	0.5325695	348.7749	0.2505627	0.2820067	TNFSF4
cg04700814	0.5433615	348.7749	0.2492602	0.2941013	HEXIM1
cg13763232	0.7312835	348.7749	0.2482646	0.4830189	SLC6A6
cg06507244	0.693307	139.7493	0.2468265	0.4464805	DHX32
cg26069745	0.5589859	142.6299	0.2460915	0.3128945	HOXA2
cg11653858	0.7029867	135.9538	0.2425544	0.4604322	SLC13A2
cg15381313	0.6481272	131.1097	0.241303	0.4068242	NR1H4
cg12177001	0.7571451	136.8648	0.2364067	0.5207384	IFI27
cg27114026	0.7668031	139.7493	0.2360043	0.5307989	ELA1
cg26511075	0.8270028	348.7749	0.2335835	0.5934194	FLJ25422
cg05342835	0.6029424	119.0505	0.2304975	0.3724449	SYNC1
cg18392482	0.7740394	132.6677	0.2286714	0.545368	AMDHD1
cg22759185	0.7859135	133.0607	0.2266619	0.5592517	REEP6
cg16363586	0.5369419	118.0146	0.2253324	0.3116095	BST2
cg24928687	0.7552074	123.4717	0.2239569	0.5312504	EPHX1
cg17186803	0.4128042	135.1871	0.2174902	0.195314	SCN4B
cg23547429	0.4535924	121.8962	0.2166732	0.2369192	SLC43A3
cg16708623	0.5011004	105.1766	0.2103171	0.2907833	TRIM2
cg24315815	0.7337341	105.4593	0.2094976	0.5242364	PLSCR4
cg20289949	0.4467449	112.3488	0.2081347	0.2386102	HAAO

cg17129388	0.7558743	106.0773	0.206287	0.5495873	NGFR
cg05722906	0.3800062	129.9799	0.2062131	0.1737931	CYP4F12
cg19224278	0.5202059	97.37476	0.2053372	0.3148687	ALDH1A3
cg05253327	0.7560579	104.8994	0.2050145	0.5510435	B3GNT1
cg13185308	0.5971879	91.88846	0.2041935	0.3929945	ABCC8
cg10055471	0.9203848	348.7749	0.203947	0.7164378	NR0B2
cg07572435	0.7755153	105.514	0.2015542	0.5739611	LY6D
cg05520656	0.2682838	348.7749	0.2015286	0.06675524	ZNF681
cg07251788	0.6877667	91.65414	0.2004234	0.4873433	CLTCL1
cg05256043	0.5875705	88.35542	0.2003055	0.387265	DCT
cg08858521	0.419414	108.8849	0.2002969	0.219117	WFIKKN1
cg18462653	0.2046788	-99.3614	-0.2000436	0.4047224	DEFB119
cg04511534	0.5530116	-113.0998	-0.2000453	0.7530569	GGT6
cg26349773	0.5451245	-110.8726	-0.2000477	0.7451722	ATP6V0A4
cg26369642	0.5754656	-120.4023	-0.2000616	0.7755272	C8orf72
cg24898863	0.1876777	-102.5606	-0.2000736	0.3877513	S100A8
cg05484458	0.5150857	-103.7888	-0.2001956	0.7152812	GNB3
cg05206661	0.3242608	-88.45398	-0.200295	0.5245558	FLJ33534
cg15501381	0.3508636	-88.16765	-0.2003096	0.5511732	NGB
cg08256781	0.4856607	-98.5591	-0.2004132	0.686074	ACSBG2
cg01040850	0.331816	-88.42261	-0.2004334	0.5322494	MR1
cg21484834	0.6270109	-144.5686	-0.2004467	0.8274575	PTK6
cg06747888	0.1962691	-101.2652	-0.2004661	0.3967353	TBR1

cg11360149	0.7100705	-217.178	-0.2005354	0.9106058	OR2V2
cg20781967	0.2357472	-95.2805	-0.2006543	0.4364015	NINJ2
cg16907566	0.1751612	-105.8329	-0.2007073	0.3758685	COL14A1
cg07897701	0.5365517	-109.4775	-0.2007174	0.7372691	ABP1
cg01138020	0.3576904	-88.63487	-0.2007815	0.5584719	MGC29671
cg26224139	0.6117709	-137.1084	-0.2008127	0.8125836	C9orf116
cg05546044	0.3579872	-88.66652	-0.200815	0.5588021	MAPK1
cg21969640	0.3132668	-89.3176	-0.2008801	0.5141469	GPR84
cg16466334	0.4554137	-94.87328	-0.2009417	0.6563554	MMP3
cg26333641	0.5060081	-102.8653	-0.2009747	0.7069829	IL22
cg02787991	0.0700649	-144.7541	-0.2010344	0.2710993	SECTM1
cg26240939	0.4713672	-97.08132	-0.2010376	0.6724048	LOC57149
cg00168942	0.327907	-89.05566	-0.2010531	0.5289601	CX40.1
cg20234959	0.4359946	-93.18576	-0.2013246	0.6373192	NID1
cg26813458	0.6637015	-171.4334	-0.2013559	0.8650575	CEACAM6
cg11380128	0.532186	-109.2137	-0.2013685	0.7335544	PRLH
cg03684977	0.5149417	-105.2634	-0.2014165	0.7163582	GRB7
cg05989054	0.4889322	-100.2788	-0.2014321	0.6903643	GAMT
cg27547703	0.4964206	-101.7262	-0.2015464	0.6979669	TDRD1
cg14519000	0.6751508	-181.9228	-0.2015958	0.8767466	GATA5
cg24101873	0.5796713	-124.4089	-0.201674	0.7813454	C20orf70
cg06293195	0.5043284	-103.5185	-0.2017943	0.7061228	TXN2
cg20104776	0.3647183	-89.69584	-0.2017973	0.5665156	LDOC1

cg23413307	0.4422694	-94.39011	-0.2018763	0.6441457	LCE1F
cg24010336	0.2248199	-97.93326	-0.201922	0.4267419	FBXO17
cg17348429	0.5850792	-127.0143	-0.2019511	0.7870303	ENPP7
cg22807700	0.3630716	-89.83076	-0.2019726	0.5650442	DNTTIP2
cg19067730	0.09405605	-133.651	-0.20199	0.296046	PPGB
cg25771201	0.3872259	-90.55153	-0.2020094	0.5892353	SCDR10
cg18431127	0.3364751	-89.89673	-0.2021066	0.5385817	EPB42
cg08804892	0.1506557	-113.3582	-0.2021633	0.352819	TRAK1
cg16377872	0.428175	-93.34877	-0.2021759	0.6303509	SLC1A6
cg20856834	0.4305288	-93.57861	-0.2021908	0.6327196	OR12D3
cg21256656	0.510094	-105.2101	-0.2022108	0.7123048	KLK6
cg10225525	0.3658567	-90.11749	-0.202222	0.5680788	FUT8
cg11505080	0.2036663	-101.5755	-0.2022367	0.405903	GPR173
cg03221914	0.1804023	-106.1471	-0.2022639	0.3826661	HIST1H2AJ
cg10536916	0.3207767	-90.35233	-0.2022662	0.523043	HIST1H4L
cg03258472	0.2371795	-96.62807	-0.2023035	0.4394829	CRB3
cg21068030	0.5283213	-109.5737	-0.2023914	0.7307128	LSMD1
cg19206010	0.2180465	-99.36835	-0.2024025	0.420449	UXT
cg24290574	0.2627182	-94.07529	-0.2025731	0.4652913	CPA3
cg19486673	0.250992	-95.25378	-0.2025745	0.4535665	LILRA2
cg13644052	0.5956047	-132.5007	-0.2025945	0.7981992	PAQR4
cg22012981	0.3544276	-90.44487	-0.2027255	0.5571531	ACOX2
cg00958560	0.4466715	-95.84428	-0.2027903	0.6494617	FLJ46481

cg04592706	0.5167238	-107.4148	-0.2028316	0.7195554	ABCA4
cg17981339	0.5624215	-120.0301	-0.2028787	0.7653002	SBEM
cg15178040	0.4460706	-95.87499	-0.2028827	0.6489533	GSTA5
cg13861644	0.4630741	-98.10513	-0.2030007	0.6660748	PIWIL1
cg12330929	0.5330703	-111.6319	-0.2030553	0.7361256	USP9X
cg16150435	0.5105851	-106.3991	-0.2030974	0.7136825	C6orf15
cg12144803	0.5825332	-127.8833	-0.2031484	0.7856817	KALRN
cg09923671	0.4793096	-100.6474	-0.2031484	0.682458	GATA5
cg08390209	0.3825158	-91.50742	-0.20317	0.5856858	CDKN2B
cg23504246	0.211026	-101.2165	-0.2031862	0.4142121	FGF7
cg06885782	0.2850199	-92.86846	-0.2031879	0.4882079	KCNQ4
cg00615241	0.2390755	-97.20674	-0.2031935	0.442269	PRTN3
cg20994561	0.6056077	-138.1703	-0.2031974	0.808805	BAI1
cg11584690	0.1579662	-112.422	-0.2032423	0.3612086	ZNF574
cg06806080	0.6224108	-147.214	-0.2032901	0.8257009	CD47
cg16609872	0.2700533	-94.10276	-0.2033092	0.4733625	KCNQ1
cg04545516	0.2724838	-93.92446	-0.2033234	0.4758072	SEMG1
cg04533291	0.5891379	-130.9726	-0.203389	0.7925268	WDR39
cg01910481	0.5212162	-109.1741	-0.2034093	0.7246255	PLUNC
cg24125648	0.1103606	-128.29	-0.2034914	0.313852	HYPK
cg04833845	0.4149586	-93.75056	-0.2036096	0.6185682	KCNN4
cg15862544	0.6377286	-157.3248	-0.2036514	0.84138	EGFL4
cg14724265	0.3543485	-91.32681	-0.2036557	0.5580043	PPEF2

cg01353448	0.172928	-109.1741	-0.2036758	0.3766038	C7orf16
cg14154330	0.2923096	-92.911	-0.2037287	0.4960383	ARHGAP27
cg22081096	0.3007708	-92.47596	-0.2037386	0.5045093	ABCA6
cg13813391	0.5243897	-110.3768	-0.20376	0.7281497	CMTM2
cg03534410	0.6578997	-172.1436	-0.2037632	0.8616629	TMEM40
cg07974891	0.324768	-91.67728	-0.2037992	0.5285671	ITGB1BP1
cg10539808	0.5019423	-105.5043	-0.2038196	0.705762	KCTD1
cg14759043	0.1492355	-115.4731	-0.2038946	0.3531301	MGC4266
cg16480209	0.6531149	-168.9923	-0.2040313	0.8571462	CNGB1
cg13283751	0.1416964	-117.8727	-0.2040754	0.3457718	GPX5
cg03914452	0.3033435	-92.68279	-0.2040916	0.5074351	PRR8
cg23181133	0.320456	-92.12511	-0.2041632	0.5246192	CEACAM3
cg13994177	0.6392351	-159.6063	-0.2042915	0.8435266	PWCR1
cg16743289	0.222179	-100.5326	-0.2043273	0.4265063	CACYBP
cg17399166	0.5268653	-111.7177	-0.2043285	0.7311938	CD1D
cg27655855	0.4990722	-105.5628	-0.2043378	0.70341	CST9L
cg08108953	0.3221661	-92.27032	-0.2043659	0.526532	WFDC13
cg07892051	0.4380661	-96.73796	-0.2044908	0.6425568	AKAP3
cg08766149	0.4815065	-102.5847	-0.2045178	0.6860244	GZMB
cg19278780	0.440922	-97.12758	-0.2045793	0.6455013	SIT1
cg07123548	0.612431	-144.2108	-0.2046322	0.8170632	HIPK4
cg14437986	0.4107319	-94.48828	-0.20464	0.6153718	C6orf25
cg10045881	0.4901162	-104.2829	-0.2046772	0.6947934	CHI3L2

cg18611122	0.410191	-94.57658	-0.204762	0.614953	LASS2
cg14458731	0.3453392	-92.34988	-0.2047651	0.5501044	COQ4
cg19382175	0.5225701	-111.232	-0.2047762	0.7273464	PDE6A
cg04995095	0.3197032	-92.86891	-0.2049434	0.5246466	CD300E
cg22789545	0.5532233	-120.0301	-0.2049645	0.7581878	C20orf114
cg06653796	0.4979872	-106.1464	-0.2049977	0.7029849	LIME1
cg19033555	0.3813765	-93.26403	-0.2050125	0.5863889	DEFB1
cg04999691	0.3572243	-92.70312	-0.2050634	0.5622877	C7orf29
cg18913171	0.5250844	-112.3488	-0.2051658	0.7302502	SLAMF9
cg16742703	0.4252211	-96.27872	-0.2052383	0.6304594	KLK3
cg06154597	0.3922946	-93.98282	-0.2052461	0.5975407	MGC4618
cg22607339	0.509111	-108.7528	-0.2052478	0.7143588	MPL
cg04415689	0.660033	-177.3813	-0.2052831	0.8653162	TMEM16G
cg18656170	0.5167412	-110.8882	-0.2055894	0.7223306	LTB
cg04558553	0.2813531	-95.5366	-0.2057916	0.4871447	UGT2B7
cg25993152	0.4977417	-107.0962	-0.2058125	0.7035542	XAGE5
cg14606768	0.3735073	-93.79254	-0.2058214	0.5793287	C9
cg03608577	0.5699576	-127.2861	-0.2059075	0.7758651	OR12D3
cg07440877	0.6639384	-182.2	-0.2059141	0.8698525	FLJ46358
cg02436686	0.2594693	-97.49596	-0.2059201	0.4653894	GMFG
cg18128666	0.5517191	-120.9072	-0.2059284	0.7576476	NCOA1
cg15329483	0.3911413	-94.62595	-0.2059486	0.5970898	SSX7
cg08047457	0.2850781	-95.5366	-0.2060615	0.4911396	RASSF1

cg20074593	0.4209613	-97.0089	-0.2062898	0.627251	GPR17
cg20283107	0.3767894	-94.59647	-0.2065307	0.5833201	FAM91A1
cg03473518	0.1021711	-134.8809	-0.2065499	0.308721	GJB6
cg05873268	0.5232201	-113.715	-0.2065679	0.7297881	TPSAB1
cg20163033	0.5237079	-113.8709	-0.2065933	0.7303012	C4orf8
cg11161417	0.4569884	-101.3284	-0.2066438	0.6636322	SPACA3
cg06074920	0.5712246	-129.1121	-0.2067941	0.7780187	KRTHA4
cg21279865	0.5232372	-114.0988	-0.206854	0.7300912	FAM106A
cg13929970	0.5009649	-109.1462	-0.2069508	0.7079158	FGFBP1
cg01617750	0.4570633	-101.6863	-0.2069512	0.6640145	CMTM8
cg12354377	0.320552	-94.8182	-0.2070236	0.5275756	ANK3
cg26917999	0.42159	-97.85574	-0.2070412	0.6286312	LZTS1
cg25710140	0.4835494	-105.9831	-0.2071017	0.6906511	MID1
cg10345936	0.4556264	-101.9217	-0.2073293	0.6629558	SLC36A2
cg02096520	0.5068915	-110.9346	-0.2073876	0.7142791	FGF16
cg24661752	0.5761784	-132.0575	-0.2074211	0.7835994	AP2M1
cg04991214	0.3814266	-95.76215	-0.2075193	0.5889459	PFDN2
cg14654731	0.444903	-100.7761	-0.2075297	0.6524326	FLJ30834
cg26128441	0.3622904	-95.19065	-0.2075345	0.5698249	P2RX3
cg19521927	0.4424699	-100.5474	-0.2075856	0.6500555	PLA2G5
cg25712380	0.4986053	-109.6962	-0.2077941	0.7063993	GRAP2
cg06803253	0.4885941	-107.8182	-0.2078668	0.6964608	GNAT1
cg10523671	0.5690612	-129.9799	-0.2078826	0.7769439	SLC15A2

cg23458892	0.4455262	-101.2971	-0.2079281	0.6534544	SIGLEC7
cg15772361	0.7088255	-238.3363	-0.2079421	0.9167676	SERPINB3
cg07873488	0.6142798	-151.3683	-0.2080093	0.8222891	MUC17
cg10146929	0.1556749	-117.8753	-0.2081421	0.363817	HIST1H1A
cg06206628	0.5399513	-120.4429	-0.2081593	0.7481106	FRMPD2
cg18988110	0.2521665	-100.3571	-0.208176	0.4603425	ATAD4
cg04001668	0.7450367	-303.2588	-0.2082204	0.9532571	GPR56
cg03573747	0.4735396	-105.785	-0.2083777	0.6819173	ADIPOQ
cg10986043	0.3185608	-96.31112	-0.2085212	0.527082	TCAP
cg11487967	0.5059331	-112.2771	-0.2086185	0.7145516	FLJ41327
cg12647587	0.5864651	-138.4051	-0.2086249	0.79509	C10orf129
cg04081402	0.4779798	-106.9727	-0.2087455	0.6867254	TCN2
cg21958034	0.5659834	-130.1516	-0.2087522	0.7747356	MST1
cg24862483	0.09097334	-142.3459	-0.2087567	0.2997301	CD300LG
cg03533858	0.3147543	-96.68515	-0.2087883	0.5235426	MORN1
cg22833175	0.2904818	-97.80792	-0.208819	0.4993008	WBP11
cg15779716	0.3377067	-96.4904	-0.2090288	0.5467355	CDCP1
cg20092728	0.4127699	-99.20407	-0.2090317	0.6218016	SLC5A12
cg19910382	0.3865103	-97.59267	-0.2090985	0.5956088	FABP1
cg05131835	0.4444117	-102.4866	-0.2091258	0.6535376	GH2
cg02046017	0.2607742	-100.3951	-0.2091378	0.469912	LOC220070
cg20423977	0.5130836	-114.6067	-0.2091547	0.7222383	PLAC4
cg18356799	0.3156203	-97.06407	-0.2092179	0.5248383	DSC1

cg16192575	0.2152717	-106.3117	-0.2093026	0.4245744	PTPN20B
cg24660086	0.4597383	-104.7462	-0.2093243	0.6690627	RGR
cg07846167	0.1214991	-130.141	-0.2094073	0.3309064	FBLIM1
cg17141902	0.4176641	-100.0769	-0.2094607	0.6271248	NINJ1
cg26414720	0.503006	-112.7669	-0.2095227	0.7125286	MAGEA1
cg01820374	0.4362683	-102.1124	-0.2096418	0.6459101	LAG3
cg14308452	0.5066762	-113.7784	-0.2096671	0.7163433	MGC24975
cg08786003	0.3013696	-98.0743	-0.2096984	0.511068	FCRL3
cg27345534	0.344697	-97.27721	-0.2098535	0.5545505	PRB2
cg01868128	0.4066241	-99.62025	-0.2098746	0.6164987	LCE5A
cg02656594	0.1943968	-110.6025	-0.2099147	0.4043115	IL21R
cg22101098	0.4589886	-105.715	-0.2102534	0.669242	SLC17A1
cg21731286	0.642886	-174.9227	-0.2102978	0.8531839	TAS1R2
cg13018903	0.4509752	-104.7196	-0.2103689	0.6613441	FLJ25530
cg17542495	0.3687823	-98.19172	-0.2103808	0.5791631	GJB1
cg19618706	0.24111	-103.8164	-0.2104988	0.4516088	BGN
cg07974303	0.3420764	-97.97424	-0.2105599	0.5526363	DEFA6
cg18604842	0.686046	-216.6688	-0.2105744	0.8966204	FLJ36046
cg02142461	0.3646055	-98.30611	-0.2106111	0.5752166	LYAR
cg11241627	0.09071147	-144.494	-0.2106378	0.3013493	FERD3L
cg24777950	0.4556159	-105.6767	-0.2106381	0.666254	CTSG
cg09133026	0.5191917	-118.1254	-0.2106589	0.7298506	RPS6KL1
cg27349244	0.4410524	-103.8446	-0.2107112	0.6517636	MLXIP

cg06518271	0.5881565	-142.87	-0.2107872	0.7989437	ADH6
cg08191854	0.1311918	-128.2506	-0.2108738	0.3420657	TRPM2
cg07371530	0.6063206	-152.262	-0.2108819	0.8172026	RPUSD1
cg10379687	0.2172285	-107.5833	-0.2109413	0.4281698	SPINLW1
cg12891678	0.1983087	-111.0109	-0.2111251	0.4094338	SPRR2D
cg16628918	0.3186795	-98.82211	-0.2111301	0.5298096	GPR172A
cg26692016	0.437261	-103.8867	-0.2111529	0.6484139	APOBEC1
cg01684901	0.4643839	-107.8091	-0.2113512	0.6757351	C3orf40
cg09871043	0.2667862	-102.0188	-0.2114162	0.4782025	PKHD1
cg26757722	0.2545471	-103.1875	-0.2114243	0.4659714	CACNG2
cg18312429	0.3395851	-98.88618	-0.2114912	0.5510763	SLC10A2
cg09432154	0.3337244	-98.93099	-0.2115054	0.5452298	GPR87
cg22954265	0.5742286	-137.8811	-0.2115325	0.7857612	FLJ45832
cg23338993	0.4481618	-105.6851	-0.2115392	0.6597009	UGT1A6
cg27117399	0.3832223	-99.9567	-0.2115577	0.5947799	CNDP1
cg02825709	0.5185591	-119.207	-0.2115863	0.7301454	ZNF75A
cg26745032	0.2426844	-104.6822	-0.2115962	0.4542806	REPS2
cg17687282	0.5458591	-127.4234	-0.2116487	0.7575078	A4GNT
cg20090497	0.3503036	-99.09348	-0.211657	0.5619607	TAS2R9
cg21277505	0.6174102	-160.3748	-0.2117205	0.8291306	LOC284361
cg16155702	0.5576288	-131.6853	-0.2117465	0.7693753	FGF21
cg05556717	0.3871081	-100.4401	-0.211842	0.5989501	CCL26
cg04014889	0.5442906	-127.2031	-0.2118485	0.7561391	MAGEL2

cg11845202	0.6188525	-161.541	-0.2118545	0.830707	K5B
cg14898892	0.3280098	-99.36835	-0.2118849	0.5398947	SHRM
cg10919204	0.2076791	-110.1624	-0.211975	0.4196541	CDH6
cg20822579	0.2134541	-109.2081	-0.2120016	0.4254557	RIPK3
cg03960217	0.1666907	-118.8819	-0.2120502	0.3787409	LCE2C
cg23264413	0.3918863	-101.0242	-0.2121456	0.6040319	PSG4
cg04645843	0.4524934	-107.097	-0.2122522	0.6647456	DPCR1
cg22980079	0.5662569	-135.9554	-0.2123564	0.7786133	C4orf8
cg23756219	0.2063733	-110.8323	-0.2124355	0.4188087	DRP2
cg16585619	0.4303517	-104.5928	-0.2124753	0.642827	KRT19
cg15698196	0.3419482	-99.9989	-0.2126061	0.5545543	CAPN12
cg13181284	0.6292015	-169.9436	-0.2126367	0.8418382	KRT6B
cg02797569	0.3636386	-100.3571	-0.2126605	0.5762991	PCOLCE
cg07378350	0.2402599	-106.0273	-0.2126817	0.4529415	GDF5
cg21732383	0.2011671	-112.1237	-0.2127965	0.4139636	THBS2
cg17067005	0.5455956	-129.1573	-0.2128879	0.7584836	IL10
cg12875426	0.46171	-109.3814	-0.2130457	0.6747558	DYM
cg12397274	0.2570097	-104.6487	-0.2131944	0.4702042	TINAG
cg18960218	0.3669408	-101.0468	-0.2132527	0.5801935	SLC7A7
cg19055231	0.06406307	-162.1195	-0.2134786	0.2775417	STAC
cg20342105	0.2857499	-102.5847	-0.2135019	0.4992518	BSCL2
cg15812957	0.457736	-109.4376	-0.2136144	0.6713504	VCY
cg24110063	0.1917673	-114.7277	-0.213616	0.4053833	COX6A2

cg20017147	0.2966903	-102.0913	-0.2136215	0.5103118	TEX101
cg04345908	0.5086855	-119.6643	-0.2137794	0.7224649	HLA-DQB2
cg17920197	0.4465494	-108.176	-0.2138993	0.6604487	LGI4
cg25691167	0.04877738	-172.5624	-0.2139099	0.2626873	FERD3L
cg18279742	0.2684724	-104.3346	-0.2139669	0.4824393	RPS2
cg09044738	0.5934936	-151.1153	-0.2140073	0.807501	FAM12A
cg21707816	0.2907229	-102.8458	-0.2140729	0.5047958	ENDOGL1
cg00443307	0.3722323	-102.0955	-0.2140917	0.586324	KLRG1
cg26200585	0.370776	-102.1546	-0.2142063	0.5849823	PRX
cg19831369	0.3553012	-101.7865	-0.214258	0.5695592	HPS4
cg24841244	0.510265	-120.7167	-0.2142962	0.7245612	CD3D
cg25141490	0.2888876	-103.2437	-0.2143744	0.503262	IL17B
cg05700681	0.5762813	-143.607	-0.2144212	0.7907025	CCL22
cg25514503	0.6753203	-215.1894	-0.2144369	0.8897572	PER3
cg01119135	0.6642192	-203.4983	-0.2144524	0.8786716	C1orf116
cg09799714	0.4928102	-116.9043	-0.2144782	0.7072884	PDZD3
cg08158289	0.1613435	-122.749	-0.2145063	0.3758498	KIAA0141
cg03977657	0.5742772	-142.8856	-0.2145288	0.788806	LAMB3
cg22563697	0.496832	-117.9027	-0.2145756	0.7114077	PPP1R16A
cg26571739	0.2011989	-113.8792	-0.2146088	0.4158077	VAV1
cg05507459	0.1988361	-114.358	-0.2146431	0.4134792	C9orf121
cg26626042	0.5770443	-144.5079	-0.2147651	0.7918094	AGER
cg12530021	0.1601247	-123.5305	-0.2149438	0.3750684	SIGLEC12

cg03835296	0.3697836	-102.9444	-0.2150141	0.5847977	SLC17A1
cg15590780	0.2823191	-104.3058	-0.2150211	0.4973402	USH2A
cg20791593	0.4322418	-107.6606	-0.2150237	0.6472656	NEU4
cg09616556	0.5338601	-128.5252	-0.215032	0.7488921	AMN
cg22927134	0.3252684	-102.5606	-0.2150672	0.5403357	CHRM5
cg20436912	0.3472666	-102.4948	-0.2150714	0.5623379	SEC14L4
cg00057593	0.3584762	-102.8929	-0.2152885	0.5737647	GML
cg12473775	0.4581185	-111.5254	-0.2153302	0.6734487	RHOD
cg18303397	0.2483139	-107.6606	-0.2153387	0.4636526	MBD4
cg26457013	0.4151965	-106.2424	-0.2153566	0.6305531	TMEM86B
cg18967533	0.3239593	-102.8713	-0.2153628	0.539322	KLK6
cg21019522	0.3054777	-103.4748	-0.2154424	0.5209201	SLC22A18
cg06850526	0.3728408	-103.52	-0.2154535	0.5882943	MGC15523
cg17199658	0.2028702	-114.4359	-0.2154845	0.4183547	MGC39715
cg17298704	0.5839178	-149.0085	-0.2155175	0.7994353	CLDN18
cg23900225	0.3595401	-103.1981	-0.2155555	0.5750955	CX40.1
cg26159905	0.3607982	-103.3238	-0.2156475	0.5764457	ASB10
cg25548825	0.5612555	-139.2858	-0.2156757	0.7769311	TBX10
cg12435792	0.2892978	-104.5337	-0.2157026	0.5050005	PDE6B
cg20322862	0.2518222	-107.6373	-0.2157076	0.4675298	TGIF
cg06812844	0.3232847	-103.2628	-0.2157399	0.5390247	TRPM2
cg13739417	0.2891634	-104.6487	-0.215813	0.5049765	IL8RB
cg24388263	0.6505524	-194.0012	-0.2158161	0.8663685	FLJ10374

cg14267151	0.4411982	-109.7556	-0.2158905	0.6570887	MUTED
cg06303238	0.4362492	-109.23	-0.2159844	0.6522335	SALL4
cg23801057	0.4279366	-108.2769	-0.2160044	0.643941	P2RX7
cg12992720	0.2488205	-108.2699	-0.2160151	0.4648356	EDG4
cg26316946	0.1265374	-135.3045	-0.2160451	0.3425826	GRIK2
cg13370916	0.213745	-113.11	-0.2160602	0.4298052	STARD8
cg13446199	0.3540049	-103.6343	-0.2160982	0.570103	PSCA
cg20368904	0.3934814	-105.4568	-0.216225	0.6097064	TNFAIP2
cg25889160	0.6269284	-176.0668	-0.2162744	0.8432028	SERPINA5
cg13424229	0.559344	-139.6343	-0.2163607	0.7757047	CPA3
cg09686308	0.2702664	-106.6297	-0.2164726	0.486739	CIB3
cg18951427	0.3210172	-104.1326	-0.2165556	0.5375728	ACYP2
cg18202456	0.7469674	-344.0233	-0.2166305	0.9635979	KLF17
cg19515446	0.6120256	-166.8266	-0.2166907	0.8287163	HIST1H1T
cg22932819	0.5971285	-158.1129	-0.2167338	0.8138623	GPNMB
cg07220939	0.4079964	-107.1379	-0.2167567	0.6247531	SLC22A12
cg19250907	0.4355898	-110.1278	-0.2168275	0.6524173	CPA6
cg03552103	0.353833	-104.5151	-0.2169404	0.5707734	09-sep
cg24926276	0.2781644	-106.5736	-0.2170292	0.4951937	LRG1
cg26531804	0.2600551	-108.2699	-0.2172038	0.4772589	SPINT1
cg14003512	0.3092874	-105.2168	-0.2173202	0.5266076	PLGLB2
cg06325687	0.6850085	-235.524	-0.2174622	0.9024706	OPN1MW
cg11291009	0.20865	-115.4267	-0.2175023	0.4261523	ARHGEF9

cg21578906	0.5054407	-123.8965	-0.217518	0.7229587	SLC5A4
cg01437411	0.09706955	-149.0012	-0.2175183	0.3145879	ATN1
cg11263296	0.09125715	-151.8851	-0.2176297	0.3088868	CCDC64
cg18294257	0.1682727	-124.1618	-0.2176528	0.3859255	SEC14L3
cg00983899	0.4194969	-109.2451	-0.2176764	0.6371733	CABP2
cg12029639	0.2225614	-113.3343	-0.2176956	0.440257	MAB21L1
cg00684178	0.5097264	-125.3479	-0.2177397	0.7274661	NEU4
cg23765993	0.2627072	-108.6699	-0.2178621	0.4805694	SPINLW1
cg14462830	0.391186	-107.1026	-0.2178976	0.6090836	TRDN
cg00474004	0.3583052	-105.6284	-0.2179204	0.5762256	IFNA14
cg02982734	0.5034292	-123.9675	-0.2179426	0.7213718	MAGEL2
cg12610070	0.07408342	-161.8515	-0.2184202	0.2925036	TSPAN15
cg13181019	0.4863178	-120.633	-0.2184632	0.7047811	MPP7
cg00319692	0.2816611	-107.8182	-0.2185354	0.5001965	ATP6V0D2
cg04484789	0.5247437	-130.872	-0.2185848	0.7433285	KRT25B
cg01432087	0.3771518	-107.0981	-0.2186455	0.5957973	FLJ21749
cg11898695	0.5159232	-128.4493	-0.2187025	0.7346257	PTCRA
cg10832945	0.3490719	-106.2862	-0.2187366	0.5678085	FLJ45964
cg03112433	0.2377306	-112.3613	-0.2188448	0.4565755	PFTK1
cg03330516	0.4034827	-109.1848	-0.2189586	0.6224413	SRMS
cg22193702	0.565311	-146.8115	-0.2192109	0.7845219	PADI1
cg21453309	0.2142942	-116.2902	-0.2193223	0.4336165	FAM101A
cg22039846	0.636198	-190.0201	-0.2193229	0.8555208	KIR2DL1

cg11656547	0.09402031	-152.4557	-0.219335	0.3133553	MAMDC2
cg04311964	0.5451729	-139.023	-0.2194051	0.764578	LYPD2
cg20305726	0.3218206	-107.0227	-0.2194158	0.5412364	DEFB126
cg15648315	0.498427	-124.7801	-0.2194421	0.717869	FLJ26443
cg17267907	0.2563442	-110.8517	-0.2194478	0.4757919	DEFA1
cg01135626	0.3654258	-107.5444	-0.2195304	0.5849562	CDX4
cg14141399	0.2872022	-108.4826	-0.2195471	0.5067493	HAS1
cg12242338	0.5017115	-125.9647	-0.219686	0.7213975	TRIM42
cg03386373	0.3496687	-107.3353	-0.2197315	0.5694003	SPATA3
cg09212058	0.4603921	-117.2545	-0.219732	0.6801241	PRKD3
cg03453449	0.3951812	-109.3836	-0.2197368	0.6149181	USP44
cg02173484	0.3133023	-107.5786	-0.2197764	0.5330787	GJA5
cg12949760	0.4747545	-119.9494	-0.2197765	0.694531	KCNQ1
cg11819637	0.4289439	-112.6936	-0.2197796	0.6487235	THPO
cg03387497	0.5588648	-145.1778	-0.2198847	0.7787495	C20orf179
cg08260891	0.07607399	-162.5331	-0.2200056	0.2960796	PPGB
cg19258973	0.394797	-109.6962	-0.2200423	0.6148393	KRTHB3
cg05241571	0.6176115	-177.8521	-0.2202133	0.8378248	UNQ467
cg13614083	0.444732	-115.3816	-0.2202474	0.6649794	KCNAB2
cg03840259	0.3709323	-108.5494	-0.2202716	0.5912039	GRAP2
cg12237269	0.4900029	-123.88	-0.2202724	0.7102754	SLN
cg03270204	0.5919597	-161.9077	-0.2202804	0.8122401	DDR1
cg10417559	0.5583347	-145.7199	-0.2203439	0.7786786	LMO6

cg11504646	0.4053442	-110.9225	-0.2203844	0.6257287	RTP1
cg17827767	0.5953508	-164.1582	-0.2204568	0.8158076	LRRC21
cg17539235	0.546217	-141.1202	-0.2204766	0.7666936	KISS1
cg11432797	0.4739419	-120.7567	-0.2205482	0.6944901	SPN
cg22825487	0.4747171	-120.9139	-0.2205507	0.6952678	VNN3
cg07895149	0.04009487	-186.8419	-0.2205872	0.260682	FAM26B
cg13086467	0.4355769	-114.5347	-0.2206309	0.6562078	MORG1
cg26306976	0.6228632	-182.7849	-0.2207357	0.843599	ITGB1BP1
cg22463915	0.3665643	-108.8694	-0.22074	0.5873044	EEF1A2
cg09863066	0.2091056	-118.6541	-0.220789	0.4298946	PVALB
cg04365980	0.4096697	-111.7954	-0.2207974	0.6304671	CLEC3A
cg20789620	0.2222428	-116.5719	-0.2208519	0.4430947	FSD1
cg19464252	0.2857238	-110.2575	-0.2212215	0.5069454	FBS1
cg06196379	0.2177104	-117.7615	-0.2213322	0.4390426	TREM1
cg00962799	0.4832915	-124.0511	-0.2215385	0.70483	SSX8
cg25787984	0.2197335	-117.7188	-0.2216065	0.44134	DKK3
cg20176989	0.3775493	-110.3434	-0.2216386	0.5991879	KIR3DL2
cg15798153	0.2036628	-120.7167	-0.2218775	0.4255403	PFTK1
cg04454050	0.3908007	-111.5593	-0.2219973	0.6127979	TREML1
cg19832721	0.4019616	-112.7238	-0.2222615	0.6242231	KIAA1267
cg22767466	0.4976611	-128.6247	-0.2223322	0.7199932	TRIM49
cg00626119	0.3144006	-110.2202	-0.2223859	0.5367865	NTRK1
cg24262376	0.3179987	-110.2525	-0.2224953	0.540494	SCNM1

cg22496683	0.3713499	-110.9841	-0.2225321	0.593882	MGC27016
cg18344063	0.274103	-112.3613	-0.222533	0.496636	MGAT4C
cg23812886	0.3097421	-110.5036	-0.2225404	0.5322825	SSX5
cg04893119	0.213326	-119.9494	-0.2227789	0.436105	PI15
cg24252809	0.3750048	-111.5254	-0.2228511	0.5978559	MRGPRX1
cg13705284	0.2900616	-111.823	-0.2230152	0.5130768	ACOX2
cg16509045	0.2579085	-114.2942	-0.2230365	0.480945	TRPM6
cg27069753	0.3009391	-111.332	-0.2230514	0.5239905	ELA3B
cg25462291	0.1429741	-137.1779	-0.2230556	0.3660297	HEYL
cg00673191	0.2239732	-118.5535	-0.223068	0.4470413	DOPEY2
cg22396129	0.2605676	-114.1637	-0.2231603	0.4837278	ZCRB1
cg26511321	0.3369316	-110.8529	-0.2232245	0.560156	HOXA7
cg04515986	0.2452495	-115.9272	-0.223285	0.4685345	FTHL17
cg02723372	0.5697455	-156.0809	-0.2233765	0.793122	RUNX3
cg01375994	0.3314298	-111.0437	-0.223421	0.5548508	MXRA5
cg10414946	0.4074758	-114.6245	-0.2234836	0.6309593	MS4A2
cg22627427	0.6216372	-187.9505	-0.2234917	0.8451289	C11orf9
cg16907488	0.4333744	-117.7007	-0.2235114	0.6568857	CCDC17
cg03993463	0.6193956	-186.4161	-0.2235608	0.8429564	KCNJ15
cg26884581	0.1737842	-129.0191	-0.2235984	0.3973826	PYGM
cg14672680	0.6607402	-224.502	-0.2237027	0.8844429	TNNI2
cg27087809	0.5283981	-139.8231	-0.223753	0.7521511	ACSBG1
cg15518950	0.2485251	-116.0671	-0.2237896	0.4723147	PRP2

cg07664027	0.5567096	-150.8834	-0.2238456	0.7805552	RPL13A
cg02658251	0.424479	-116.998	-0.2238896	0.6483686	DEFB4
cg00364814	0.4329502	-118.1859	-0.2239744	0.6569245	R3HDM2
cg07173760	0.2874796	-112.9453	-0.2239871	0.5114667	CLC
cg06132342	0.3600129	-112.2092	-0.2240814	0.5840943	KRTHB5
cg07937272	0.2574245	-115.5969	-0.224254	0.4816785	PARP12
cg09748960	0.2712558	-114.3533	-0.2242625	0.4955182	BTNL2
cg13828758	0.273511	-114.2003	-0.2242858	0.4977968	NDN
cg14321743	0.42284	-117.3653	-0.2243665	0.6472065	PLA2G2D
cg11721194	0.3508886	-112.2682	-0.2243806	0.5752692	SLAMF7
cg00466436	0.2890034	-113.294	-0.2244083	0.5134117	DEFB126
cg23114594	0.4846773	-128.336	-0.2244261	0.7091034	C10orf30
cg13445249	0.3386483	-112.1692	-0.2244577	0.5631061	DSG4
cg00823148	0.5078383	-134.5338	-0.224533	0.7323713	CRYGB
cg00777121	0.2268729	-119.6932	-0.2246031	0.451476	RASSF1
cg17217677	0.4218591	-117.6313	-0.2246934	0.6465526	SMPD3
cg12682367	0.3883427	-114.4359	-0.2247436	0.6130863	FLJ46358
cg20011352	0.1296738	-143.5838	-0.2247488	0.3544226	GPR124
cg10051054	0.5175785	-137.8112	-0.224767	0.7423455	CCDC27
cg05135288	0.2503102	-117.0429	-0.2249336	0.4752438	RHOT2
cg17191715	0.4420363	-120.8655	-0.2250957	0.667132	CA1
cg16567044	0.3394385	-112.8535	-0.2251125	0.564551	MEG3
cg00406188	0.3969425	-115.6008	-0.2251582	0.6221007	LCE2C

cg20790540	0.4319594	-119.6643	-0.2252899	0.6572493	PTCRA
cg26218269	0.2634018	-116.1754	-0.2253715	0.4887733	MAB21L2
cg14178895	0.2279179	-120.3357	-0.2253852	0.4533031	C6orf105
cg03956628	0.1740755	-130.8432	-0.2253882	0.3994637	MLH1
cg01185080	0.3244218	-113.2157	-0.225447	0.5498688	ZNF710
cg25082710	0.219505	-121.7242	-0.2254889	0.4449939	IVL
cg09343150	0.549452	-150.5838	-0.2255042	0.7749562	MEN1
cg06577725	0.4441628	-121.7312	-0.2255097	0.6696725	SPATA16
cg10099900	0.4769881	-128.1315	-0.2255631	0.7025512	PSMF1
cg26227465	0.3323736	-113.3262	-0.2255849	0.5579584	IFNG
cg20401945	0.1518883	-137.2531	-0.2257033	0.3775916	ASPHD1
cg22424444	0.6536704	-222.4047	-0.2257578	0.8794282	TOLLIP
cg18994063	0.3182959	-113.705	-0.2258256	0.5441215	TIMD4
cg03312792	0.5686073	-159.9838	-0.2258748	0.7944821	SEMG2
cg14288464	0.5413928	-148.442	-0.226184	0.7675768	WFDC5
cg21846488	0.1796428	-130.4675	-0.2263098	0.4059526	LCE4A
cg07657776	0.4061101	-117.8765	-0.2263883	0.6324984	UBE3B
cg23047271	0.1111822	-152.8436	-0.2266049	0.3377871	PRICKLE2
cg04132607	0.6324897	-204.1801	-0.2266346	0.8591244	GATA5
cg04623837	0.07476372	-170.9396	-0.2266625	0.3014262	HCG9
cg21045917	0.3927364	-117.1192	-0.2267816	0.619518	ALAS2
cg17910564	0.3526103	-114.988	-0.2268496	0.57946	VDAC3
cg17786776	0.3580202	-115.2119	-0.2268927	0.584913	FKBP9

cg07924575	0.2919595	-115.7589	-0.2269194	0.5188788	HPS4
cg15234096	0.472664	-129.0412	-0.226961	0.699625	LIX1
cg12775613	0.40224	-118.1684	-0.2269681	0.6292081	HTR1F
cg25650811	0.4405864	-123.1045	-0.2270429	0.6676292	LOC223075
cg17706173	0.2113422	-124.7674	-0.2270778	0.43842	C16orf30
cg02397720	0.1190459	-150.1537	-0.2271084	0.3461542	RAB17
cg17501569	0.5844321	-170.8244	-0.2271537	0.8115858	MSLN
cg10490064	0.4884852	-133.0932	-0.2271955	0.7156807	CRYBB2
cg22381955	0.7304013	-348.7749	-0.2272445	0.9576458	CHFR
cg18884741	0.5919424	-175.8132	-0.2273896	0.819332	RABGEF1
cg07022477	0.3126059	-115.5575	-0.2274546	0.5400606	HIF3A
cg23213217	0.3304578	-115.4668	-0.2275667	0.5580245	DEGS1
cg17687962	0.5096968	-139.6139	-0.2275814	0.7372782	KLK3
cg07296772	0.5170985	-141.9682	-0.2275887	0.7446873	CHMP4A
cg21723486	0.3611399	-116.1412	-0.227624	0.5887639	TP73L
cg19875656	0.5641456	-160.9859	-0.227639	0.7917845	TSP50
cg01739167	0.3449625	-115.7972	-0.227751	0.5727136	CHRNE
cg25778479	0.1600235	-137.1139	-0.2277686	0.3877921	ANKMY2
cg00466249	0.3685535	-116.6604	-0.2277876	0.5963411	MGC15523
cg23683201	0.4338133	-123.0498	-0.227825	0.6616384	SLC22A9
cg27285056	0.467901	-129.1785	-0.2278255	0.6957265	NAPSA
cg01917648	0.2972334	-116.4882	-0.2278409	0.5250742	SPIC
cg16601385	0.3978977	-118.9715	-0.2279941	0.6258917	CFD

cg05275605	0.6695982	-247.6331	-0.2280443	0.8976426	C21orf123
cg23065097	0.211938	-125.7195	-0.2280629	0.4400008	FKBP1B
cg03379131	0.05784637	-183.1756	-0.2281241	0.2859704	ADAM15
cg07506795	0.6450031	-219.9616	-0.2281993	0.8732024	ZNF19
cg23444894	0.5145296	-142.164	-0.2282607	0.7427903	UNQ5810
cg23696886	0.1650369	-136.3306	-0.2283128	0.3933496	PDLIM2
cg05600717	0.07790525	-171.5166	-0.2286945	0.3065997	FLJ13639
cg18806980	0.4196835	-122.236	-0.2287543	0.6484378	KIAA0703
cg05446471	0.6106144	-191.4423	-0.2287717	0.8393861	HDAC11
cg25598083	0.3616797	-117.4581	-0.2287829	0.5904626	ACOT2
cg13407883	0.3613777	-117.473	-0.2288094	0.5901871	SIGLEC9
cg14404298	0.3387633	-116.9276	-0.2288656	0.5676289	CYP8B1
cg06233503	0.3351398	-116.8972	-0.2288657	0.5640055	KCNQ1
cg02202484	0.3864058	-119.0722	-0.2289189	0.6153247	SPRR4
cg03818682	0.4010755	-120.4363	-0.229004	0.6300795	MUC5AC
cg01987509	0.3562335	-117.6192	-0.2291169	0.5853504	PGR
cg07005767	0.4906001	-136.4593	-0.2291587	0.7197588	ABRA
cg23641267	0.4176008	-122.725	-0.2293546	0.6469554	LPXN
cg01587454	0.6215651	-201.4824	-0.229408	0.8509731	WDR21C
cg06793062	0.5333557	-150.8952	-0.2295982	0.7629538	CNTNAP4
cg09971646	0.1795304	-134.0368	-0.229605	0.4091354	DLK1
cg05341878	0.3109239	-117.8918	-0.2296068	0.5405307	RIMS2
cg22218909	0.6342463	-213.2303	-0.2296345	0.8638808	DEFA3

cg08711674	0.2721584	-119.9039	-0.229653	0.5018114	AKT1S1
cg13733733	0.3572831	-118.4152	-0.2298016	0.5870848	LILRA3
cg19906550	0.3855637	-120.099	-0.2298681	0.6154318	SLC22A18
cg04048249	0.4278722	-124.8311	-0.2299363	0.6578084	APOC3
cg07608333	0.278921	-119.8705	-0.2300811	0.5090021	CD209
cg12547930	0.3850092	-120.3322	-0.2301027	0.6151119	WFDC6
cg20951539	0.4017378	-121.8139	-0.2301043	0.6318421	PXT1
cg05942970	0.6636738	-246.3971	-0.2301271	0.8938009	C10orf39
cg22436411	0.3822428	-120.3989	-0.2303458	0.6125886	FCGR2B
cg19998328	0.3340025	-118.623	-0.2304541	0.5644566	LOC90580
cg14911395	0.2403131	-124.1313	-0.2305958	0.4709089	SEMA3B
cg22937804	0.3077793	-119.0505	-0.2305975	0.5383769	MGC44505
cg11297236	0.2785886	-120.4429	-0.2306221	0.5092107	PDILT
cg25553916	0.2920533	-119.7907	-0.230725	0.5227784	FLJ22318
cg03014957	0.3358972	-118.9542	-0.2307377	0.5666349	DEFB118
cg21094154	0.4874387	-137.9796	-0.2308084	0.7182471	TNFSF11
cg01367992	0.3588064	-119.6339	-0.2308194	0.5896258	LY9
cg00280814	0.6012024	-189.1597	-0.2308462	0.8320487	FLJ31568
cg11267879	0.3317075	-119.068	-0.2308736	0.5625811	CYP2F1
cg04557383	0.09437371	-165.3826	-0.2308772	0.3252509	MT1H
cg24691255	0.5921419	-183.164	-0.2309141	0.823056	SERPINB2
cg21541083	0.5755236	-173.33	-0.2310725	0.8065962	STXBP2
cg13877895	0.6063272	-193.5065	-0.2311292	0.8374563	GKN1

cg13410437	0.4857986	-138.0162	-0.2311365	0.716935	OR5P2
cg03458191	0.5184245	-147.9253	-0.2311428	0.7495673	SAA1
cg26661623	0.4026345	-123.3452	-0.2312751	0.6339096	ASGR2
cg14173523	0.4293409	-126.8619	-0.2313341	0.660675	FUT5
cg27214365	0.5255678	-151.1611	-0.2315726	0.7571404	GYPB
cg25677709	0.5097864	-145.8184	-0.2316507	0.7414371	NDST1
cg24076830	0.3390107	-120.0921	-0.2317421	0.5707527	OR5V1
cg02765820	0.3449202	-120.3246	-0.2318457	0.576766	C3orf22
cg22609784	0.2187094	-128.6588	-0.2318691	0.4505785	MSX1
cg19717326	0.2153663	-129.3206	-0.2319871	0.4473534	MYADM
cg13601079	0.5370651	-156.4181	-0.2319887	0.7690538	SSX3
cg19464944	0.313268	-120.5244	-0.2321003	0.5453683	FCGR1A
cg13694749	0.4523713	-131.9097	-0.232186	0.6845574	SCN4A
cg17820828	0.4922748	-141.459	-0.2322656	0.7245404	KCNQ1
cg05248470	0.2531202	-124.4531	-0.2322817	0.4854019	LILRB2
cg14440664	0.3288474	-120.6947	-0.2323755	0.5612229	PDCD1LG2
cg08816023	0.3036424	-121.116	-0.2324145	0.5360569	FGF1
cg00134539	0.4773838	-137.7897	-0.2324648	0.7098486	UBASH3A
cg03352153	0.4625567	-134.7238	-0.2327194	0.6952761	GLULD1
cg10885338	0.3785019	-123.0319	-0.2328066	0.6113085	ECRG4
cg24490338	0.4870744	-140.8363	-0.2328219	0.7198963	TPM3
cg13530039	0.3007274	-121.7627	-0.232906	0.5336334	CHRM1
cg23218877	0.4153374	-126.9244	-0.2329308	0.6482682	C10orf9

cg26771272	0.5683684	-172.9727	-0.2329493	0.8013176	SMCR7
cg24870273	0.3827364	-123.5313	-0.232963	0.6156994	STK19
cg23328404	0.3725308	-122.8993	-0.2330412	0.605572	ChGn
cg01053621	0.3346139	-121.8418	-0.2333677	0.5679817	APOA2
cg24816866	0.5141323	-149.9573	-0.2333781	0.7475104	PARK2
cg05569220	0.3470109	-122.1571	-0.2334278	0.5804387	FLJ44861
cg23753610	0.6966464	-307.5182	-0.233544	0.9301904	DNAHL1
cg00603172	0.512613	-149.8226	-0.2336194	0.7462324	BOK
cg10848367	0.4205082	-128.5853	-0.2336919	0.6542001	SCGB1D2
cg12687463	0.2874047	-123.2786	-0.2337179	0.5211226	CFHR1
cg24974599	0.4279639	-129.7647	-0.2337796	0.6617435	CFH
cg27159719	0.1936632	-135.3898	-0.2337855	0.4274487	TMEM71
cg27147004	0.5984142	-193.6247	-0.2337897	0.8322039	PIWIL1
cg18063149	0.4564127	-134.9758	-0.2338481	0.6902608	FMO3
cg10779183	0.5082952	-148.7899	-0.2338858	0.7421809	ELA3A
cg21960110	0.4418234	-132.2702	-0.2339314	0.6757548	HBZ
cg21402035	0.368232	-123.7243	-0.2339716	0.6022036	GALR3
cg17142134	0.6163093	-208.96	-0.234391	0.8507003	SLC2A2
cg17091851	0.2930851	-123.7651	-0.2344323	0.5275174	LOC348174
cg08695223	0.2971879	-123.7319	-0.2345613	0.5317491	SLC9A6
cg03916421	0.1914443	-136.7382	-0.2345812	0.4260255	LOC132321
cg14015044	0.05514169	-192.8586	-0.2345933	0.289735	TNFRSF10C
cg13482233	0.474011	-140.0317	-0.2346182	0.7086292	HEPH

cg16899306	0.387115	-125.9153	-0.2346197	0.6217347	HLA-DQB2
cg24621042	0.308225	-123.4939	-0.2346619	0.5428869	SERPINA1
cg24541550	0.3386225	-123.5447	-0.2348017	0.5734241	MRVI1
cg10503138	0.4868561	-143.7555	-0.234841	0.7216971	CNTN4
cg03782453	0.149296	-148.3445	-0.2350869	0.3843829	FLJ90575
cg18059223	0.3810874	-126.2843	-0.2353396	0.616427	NALP2
cg20516209	0.3977765	-127.8833	-0.2353551	0.6331316	EMILIN1
cg06277657	0.2440684	-129.002	-0.2354955	0.4795639	DGKI
cg26770281	0.6259612	-220.3191	-0.235511	0.8614722	FLJ14768
cg26918728	0.5980395	-197.3103	-0.2355756	0.8336151	SEMA3B
cg09584711	0.2179215	-133.0143	-0.235771	0.4536925	HPR
cg03860768	0.4081313	-129.5634	-0.2357751	0.6439065	BLK
cg06226384	0.1684461	-143.607	-0.2357816	0.4042277	CACNG5
cg14869028	0.1028471	-167.026	-0.2357884	0.3386355	TINAGL1
cg03782727	0.6192601	-215.3519	-0.2359555	0.8552155	FFAR1
cg05492113	0.4299538	-133.1035	-0.2361014	0.6660552	TUB
cg15830940	0.3297043	-124.9655	-0.2361341	0.5658384	SMARCAL1
cg09841009	0.5177139	-155.8913	-0.2362092	0.7539231	GYPA
cg20488657	0.4550422	-137.989	-0.2362553	0.6912975	TFF3
cg00344709	0.5390438	-164.8517	-0.2363951	0.7754389	ANKRD21
cg25020204	0.3417281	-125.4727	-0.2363987	0.5781268	DBH
cg08603768	0.4196449	-131.9695	-0.2364368	0.6560817	WNT8A
cg01407244	0.1679115	-144.494	-0.2364639	0.4043754	TNFRSF10C

cg16899036	0.3770697	-127.5884	-0.2366641	0.6137337	HOMER3
cg02988947	0.311791	-125.6922	-0.2366668	0.5484578	LIMD2
cg18509435	0.4815055	-144.9973	-0.2366763	0.7181818	CDH20
cg19138960	0.2576455	-128.8608	-0.2367084	0.4943539	C1QR1
cg03775123	0.4741894	-143.2147	-0.2367658	0.7109551	ANKDD1A
cg09948350	0.5582989	-174.9883	-0.2368093	0.7951082	FLJ25084
cg09418321	0.2423081	-130.8004	-0.236939	0.4792472	DYRK4
cg13530946	0.3596939	-126.8317	-0.2369568	0.5966507	IARS2
cg04117029	0.4676306	-141.9085	-0.2369876	0.7046182	UROS
cg10329418	0.2297478	-132.5983	-0.2370463	0.4667942	PON3
cg03574571	0.5224782	-159.1391	-0.2370874	0.7595656	CD22
cg06001166	0.5859318	-192.2447	-0.2371503	0.8230821	RPL3L
cg23282949	0.4185149	-132.724	-0.2371507	0.6556656	RENBP
cg26385286	0.2353177	-132.1433	-0.2373458	0.4726635	GCNT2
cg15880738	0.4455484	-137.6271	-0.2373841	0.6829324	CD3G
cg11512771	0.5881056	-194.2504	-0.237404	0.8255095	ECM1
cg04387658	0.4462341	-137.9075	-0.2374988	0.6837329	CD86
cg05019001	0.2229036	-134.148	-0.2375229	0.4604265	AR
cg16825643	0.699752	-329.0715	-0.2376157	0.9373677	FAM38A
cg24147596	0.4160553	-132.9838	-0.237619	0.6536743	ARL14
cg10498097	0.4835549	-146.9867	-0.2376318	0.7211867	MGC50811
cg19764418	0.3261438	-126.7173	-0.2376392	0.563783	RYR2
cg14481339	0.570859	-183.7696	-0.2376775	0.8085364	KCNJ1

cg02882813	0.5277486	-162.2778	-0.2376957	0.7654443	CST5
cg00690280	0.2693562	-128.9836	-0.2377626	0.5071188	WFDC10B
cg22510822	0.4064417	-131.9059	-0.237774	0.6442158	OR1E2
cg06066303	0.5691219	-182.9417	-0.2377752	0.8068971	MS4A5
cg19147390	0.150873	-151.284	-0.2381441	0.3890171	UHRF1
cg15626350	0.2293137	-133.8737	-0.2381483	0.467462	ESR1
cg15782391	0.330398	-127.3493	-0.2381606	0.5685586	ACPT
cg02504280	0.4490879	-139.7356	-0.2383831	0.6874709	MAGEA12
cg24546463	0.2240591	-135.0899	-0.238535	0.4625942	MGC39715
cg27337148	0.1979073	-139.8605	-0.2386472	0.4365545	CAMK1G
cg23152755	0.3202331	-128.2711	-0.2389782	0.5592114	DEFB105A
cg07922606	0.2559739	-131.545	-0.2389823	0.4949563	HIST1H3E
cg18530716	0.1016439	-171.714	-0.2393027	0.3409466	SLC16A11
cg15597540	0.6478311	-254.3708	-0.2393473	0.8871784	CYB561D2
cg21301440	0.102263	-171.5141	-0.2393711	0.3416341	CYGB
cg09548084	0.3385739	-128.8916	-0.2393774	0.5779514	SLC35B3
cg15480475	0.4822495	-149.4742	-0.239531	0.7217804	TUB
cg04520391	0.5076836	-157.7403	-0.2395849	0.7472685	PRB2
cg08124722	0.6013812	-209.1899	-0.2396583	0.8410395	CCL7
cg21434954	0.4062169	-134.3227	-0.2396808	0.6458977	LCE1B
cg24304714	0.197893	-141.0727	-0.239707	0.4376	LCE1C
cg24765079	0.2048484	-139.7493	-0.2397664	0.4446149	CDH1
cg23464269	0.3032053	-129.4813	-0.2398262	0.5430315	UGT1A3

cg05654163	0.3906471	-132.6999	-0.239834	0.6304811	SLC39A2
cg15303841	0.3612736	-130.3371	-0.2398358	0.6011094	RFPL1
cg00476577	0.07721754	-185.2461	-0.2398411	0.3170587	ZNF217
cg13112511	0.3977872	-133.6201	-0.2399403	0.6377275	PDE4D
cg22445920	0.4071589	-134.8285	-0.2399781	0.647137	SLC36A3
cg23704362	0.3079583	-129.5598	-0.2399957	0.547954	C8orf46
cg00620024	0.4671641	-146.2067	-0.2400431	0.7072072	PPP6C
cg04675937	0.3920677	-133.117	-0.2400558	0.6321235	CDKN2B
cg03545635	0.5249546	-165.2797	-0.2401134	0.765068	CHST12
cg17926869	0.5311932	-167.9664	-0.2401245	0.7713177	LOC115098
cg19592945	0.1819195	-145.1571	-0.2402226	0.4221421	P2RXL1
cg02992596	0.3195022	-130.1035	-0.2405865	0.5600887	MGC27016
cg13053396	0.3967267	-134.4087	-0.2406505	0.6373771	C1S
cg06022562	0.5718797	-190.593	-0.2406955	0.8125752	FLJ13841
cg13461622	0.4071532	-135.879	-0.2407807	0.6479338	RUNX3
cg09283007	0.6633656	-280.1976	-0.2410003	0.9043659	FAM47B
cg09691574	0.5600638	-184.1846	-0.2410247	0.8010886	MRGPRX4
cg18729973	0.5047862	-159.0988	-0.2410406	0.7458268	TFF1
cg02579133	0.4319502	-140.0711	-0.2410456	0.6729958	KRTAP10-8
cg27050763	0.5729588	-192.3416	-0.2412054	0.8141642	AHCTF1
cg25882366	0.1431598	-157.3698	-0.2412474	0.3844072	HOXB2
cg09837803	0.2851827	-131.9039	-0.2412878	0.5264704	IL16
cg22220722	0.5321267	-170.6396	-0.2413808	0.7735075	PLA2G2A

cg25391023	0.2853937	-132.041	-0.241427	0.5268207	BTNL2
cg00750606	0.2980546	-131.4954	-0.2414308	0.5394855	CDA
cg22294577	0.5628553	-186.7822	-0.2415203	0.8043756	SLC26A3
cg19841506	0.1060543	-172.3891	-0.2416064	0.3476608	ZMYND15
cg12858514	0.6423836	-254.6782	-0.2416702	0.8840538	PADI3
cg21643191	0.382506	-134.3108	-0.2417434	0.6242494	ABCB5
cg20261167	0.1709213	-149.7455	-0.2418589	0.4127803	SPP1
cg05826823	0.5794472	-198.0465	-0.2418872	0.8213344	CIZ1
cg26777475	0.3559102	-132.6026	-0.2419363	0.5978466	PCOLCE
cg16219122	0.2401309	-136.6767	-0.2419641	0.482095	ABCB1
cg09931793	0.4492289	-144.793	-0.2419701	0.6911989	OR2K2
cg08292050	0.5156402	-165.278	-0.2423093	0.7579495	SOCS4
cg22545356	0.492785	-157.4779	-0.2425773	0.7353623	MMRN2
cg05379350	0.1591785	-154.1971	-0.2428647	0.4020432	GIT1
cg09914304	0.5496357	-182.4267	-0.2430559	0.7926916	PRF1
cg27519140	0.3293664	-133.0932	-0.2430827	0.5724491	RPP21
cg26391080	0.4810144	-154.6124	-0.2430881	0.7241026	SH2D4B
cg13125510	0.2272897	-139.7403	-0.2431128	0.4704025	C11orf44
cg03224418	0.382649	-136.1097	-0.2431566	0.6258056	SAMD10
cg04273431	0.1457348	-158.9377	-0.2433335	0.3890684	PRR3
cg09222115	0.4273988	-142.681	-0.2435209	0.6709198	OTOS
cg15703357	0.3533623	-134.4201	-0.2435339	0.5968962	LINCR
cg24235633	0.5615892	-190.5104	-0.2437078	0.805297	CDIPT

cg08970694	0.3618883	-135.1786	-0.243748	0.6056363	HBE1
cg22951794	0.5180748	-168.8381	-0.243807	0.7618818	OR10A5
cg06415153	0.3080952	-134.0368	-0.2438429	0.5519381	PITPNM2
cg14902389	0.4781199	-155.0197	-0.2438934	0.7220133	MGAT4A
cg20802392	0.2738272	-135.5977	-0.2439033	0.5177305	CTSK
cg05788638	0.4561056	-149.2078	-0.2439483	0.7000539	SERPINA10
cg05800321	0.5869646	-208.5332	-0.2441731	0.8311377	LY6D
cg08124399	0.4166882	-141.7817	-0.2441787	0.6608669	DDX43
cg20713492	0.08631485	-185.5493	-0.2443014	0.3306163	AQP10
cg26894575	0.3332873	-134.6776	-0.2443415	0.5776288	S100A4
cg06836849	0.1410907	-161.8824	-0.2445324	0.3856231	SLC17A8
cg07294541	0.07289106	-193.6785	-0.2446344	0.3175254	DEF6
cg15602735	0.4429653	-147.4136	-0.2447546	0.6877199	MAGEC2
cg11885098	0.148361	-159.7339	-0.2447663	0.3931274	EFNA2
cg18192417	0.1372264	-163.5869	-0.2448264	0.3820527	NEBL
cg26415633	0.2226629	-142.5441	-0.244953	0.4676159	KLK1
cg14036856	0.3176066	-135.2825	-0.244977	0.5625836	MGC52423
cg21209356	0.4424043	-147.675	-0.2450225	0.6874267	CSF2RB
cg11237817	0.3597212	-136.6536	-0.2450467	0.6047679	KIR3DL3
cg13552869	0.1528126	-158.6886	-0.2450763	0.3978889	SEZ6L2
cg08859675	0.3853339	-139.2004	-0.245357	0.6306909	PDE4A
cg01305625	0.2832461	-136.7613	-0.2454171	0.5286632	PDLIM4
cg14264994	0.6263599	-247.8201	-0.245614	0.8719739	CTCFL

cg18239253	0.3133829	-136.2297	-0.2457492	0.559132	DEFB32
cg03900284	0.4768418	-157.7716	-0.2459109	0.7227527	P518
cg11921829	0.4204593	-144.7561	-0.2459179	0.6663772	FCRL2
cg04143809	0.3258609	-136.5431	-0.2459913	0.5718522	FLJ39822
cg24866437	0.4815278	-159.5102	-0.2460989	0.7276267	ALPK1
cg14088161	0.2846384	-137.5533	-0.246161	0.5307994	ACTL6B
cg08459368	0.5495923	-188.5321	-0.2461758	0.7957681	SCGB2A1
cg21519900	0.2833906	-137.633	-0.2461759	0.5295665	C20orf186
cg24625388	0.2664174	-138.92	-0.2462992	0.5127165	NEBL
cg02064402	0.4828488	-160.3575	-0.2463695	0.7292182	SLC6A18
cg15374234	0.3403395	-137.4352	-0.2464551	0.5867946	CD300LF
cg10125195	0.4439254	-150.1439	-0.2465144	0.6904398	LACRT
cg15670863	0.4189311	-145.3968	-0.2465678	0.6654989	SPACA4
cg23022999	0.3910207	-141.6309	-0.2467279	0.6377487	FLJ45909
cg23641264	0.4482726	-151.4765	-0.2467526	0.6950252	JRK
cg00415993	0.2049654	-147.6521	-0.2467642	0.4517296	F2RL2
cg01474260	0.3521394	-138.4444	-0.2468573	0.5989967	CESK1
cg16381688	0.3022677	-137.7227	-0.2468697	0.5491374	THEM2
cg05730092	0.4984969	-166.5998	-0.2469399	0.7454368	KIAA1446
cg09914773	0.4815105	-161.0144	-0.2470409	0.7285514	CYP3A4
cg06385087	0.04161858	-218.4979	-0.2471612	0.2887798	CTSZ
cg17386181	0.4856004	-162.8184	-0.2473538	0.7329543	MT1B
cg14338548	0.5972776	-224.6609	-0.2474889	0.8447666	LALBA

cg18473117	0.4504361	-153.1171	-0.2474991	0.6979352	CCDC22
cg15746187	0.4036401	-144.3927	-0.2475757	0.6512157	FBXO44
cg07947016	0.234233	-143.9661	-0.2476597	0.4818926	KLK2
cg22780475	0.333105	-138.7844	-0.2477307	0.5808358	CBLC
cg09551916	0.2814993	-139.8656	-0.2479966	0.529496	CFHR2
cg20692181	0.3672324	-141.1284	-0.2481628	0.6153951	RETN
cg13396068	0.4076138	-145.8086	-0.248189	0.6558028	DCD
cg15992730	0.288254	-139.7651	-0.2481918	0.5364458	GDF3
cg20795863	0.4466195	-153.2715	-0.2482128	0.6948323	NEU2
cg01361446	0.3757361	-141.9837	-0.2482457	0.6239817	IL2RG
cg21109025	0.4709354	-159.7505	-0.2482793	0.7192147	CCL2
cg27168844	0.3856453	-143.5306	-0.2486323	0.6342776	IL17
cg18807515	0.3732635	-142.297	-0.2486595	0.621923	PRAMEF2
cg15983538	0.4970742	-169.132	-0.2487548	0.745829	SEMA4A
cg26717133	0.242286	-144.2108	-0.2487572	0.4910431	PCDH21
cg00714377	0.1489242	-164.4015	-0.2489131	0.3978373	SLA2
cg05301852	0.3457112	-140.7872	-0.2489687	0.59468	FABP1
cg16673198	0.3885242	-144.3308	-0.2489964	0.6375206	CPNE4
cg19824441	0.3815079	-143.6812	-0.2491025	0.6306103	ADMR
cg08678755	0.4728641	-161.634	-0.2491088	0.7219729	ENPP3
cg25957124	0.2780446	-141.4807	-0.2491743	0.5272189	DNAH3
cg19971655	0.64571	-282.2487	-0.2492079	0.8949178	BSND
cg03548857	0.211842	-149.293	-0.2492765	0.4611185	FFAR2

cg06958211	0.3480201	-141.3655	-0.2493413	0.5973614	PAK6
cg14034870	0.3343896	-140.83	-0.2493495	0.5837391	SFTPG
cg24654350	0.4624793	-159.1151	-0.2494017	0.711881	KIR3DL1
cg12781568	0.3072443	-141.0727	-0.2497227	0.556967	WT1
cg27065979	0.5565285	-200.0897	-0.2498108	0.8063393	NEK3
cg04740359	0.2395044	-145.8182	-0.2498553	0.4893597	NTF3
cg17034109	0.2553468	-144.247	-0.2500062	0.505353	CYB561D1
cg15756091	0.3998707	-147.2093	-0.2500554	0.6499261	RGSL2
cg08471713	0.5851701	-220.9048	-0.2500855	0.8352556	MEOX1
cg21458041	0.3755651	-144.3805	-0.2501015	0.6256666	TNP2
cg09142399	0.405891	-148.165	-0.2501118	0.6560028	CRYZ
cg18085517	0.2933047	-141.9085	-0.2501376	0.5434423	TRPM1
cg18849169	0.4481159	-156.6142	-0.2501913	0.6983072	GPX3
cg02688643	0.4661749	-161.4134	-0.2502055	0.7163805	MGST2
cg06142324	0.3838381	-145.4952	-0.2502879	0.634126	FLJ25530
cg21296676	0.2802268	-142.7273	-0.2503306	0.5305574	EYA4
cg18023080	0.4062259	-148.5444	-0.2503515	0.6565773	MASP2
cg17192247	0.1068949	-182.7802	-0.2504865	0.3573814	MAPRE3
cg16739580	0.3823623	-145.622	-0.250506	0.6328683	POP2
cg03291145	0.3937111	-147.2896	-0.2507397	0.6444508	ARSF
cg18750960	0.245819	-146.1294	-0.2507626	0.4965817	HOXD4
cg19421752	0.1489412	-166.6313	-0.2508005	0.3997418	SLC6A18
cg06985415	0.5961958	-232.2014	-0.2508682	0.847064	C10orf39

cg03741352	0.4840969	-168.1162	-0.2508923	0.7349892	LCE3C
cg19635695	0.2778809	-143.6688	-0.2510033	0.5288842	PDE6C
cg09626634	0.2882023	-143.1572	-0.2510097	0.539212	EBI2
cg10787197	0.3205899	-142.7059	-0.2510828	0.5716727	C6orf105
cg21023001	0.2544853	-145.6347	-0.2511191	0.5056044	C1QR1
cg06825166	0.2834806	-143.6134	-0.2512116	0.5346922	TMEM10
cg07373172	0.3858117	-147.1981	-0.2513972	0.6372089	IFNA21
cg12619162	0.5169925	-182.4718	-0.2516088	0.7686013	FXYD4
cg06818777	0.1050799	-185.0173	-0.251609	0.3566888	CHAD
cg05696092	0.2331221	-148.7543	-0.2516573	0.4847794	NOSIP
cg21130374	0.3142623	-143.4709	-0.2517165	0.5659788	MX2
cg06277277	0.2337974	-148.769	-0.2517502	0.4855476	NR1I3
cg24697329	0.4878142	-170.8881	-0.2517656	0.7395797	ARHGEF4
cg10300154	0.3738896	-146.418	-0.2517831	0.6256728	MGC5297
cg06821120	0.2494536	-146.9798	-0.25182	0.5012736	RASSF1
cg14107638	0.4244716	-153.8756	-0.2518499	0.6763214	MAGEA5
cg12629515	0.2278369	-149.752	-0.2518739	0.4797108	HIST1H2BO
cg02280309	0.4064123	-150.7424	-0.2519127	0.658325	PKLR
cg10818781	0.3693992	-146.228	-0.2519622	0.6213614	PHB2
cg10432859	0.388385	-148.2948	-0.2519861	0.6403711	UGT1A7
cg20657421	0.4739642	-166.6301	-0.2520141	0.7259783	CCL4
cg01993576	0.1376941	-172.0078	-0.2520624	0.3897566	SLC29A1
cg00256074	0.5112333	-180.9784	-0.2521862	0.7634194	MYLPF

cg14544583	0.2970167	-144.3308	-0.2522421	0.5492588	HBB
cg26155152	0.446862	-159.539	-0.2523296	0.6991917	ZNF77
cg11765205	0.136088	-172.8933	-0.2523311	0.3884191	ARL11
cg19096475	0.3694352	-146.7324	-0.2523366	0.6217718	ASAM
cg21789545	0.2233734	-151.0431	-0.2524047	0.475778	COL9A1
cg02097420	0.4029606	-150.9409	-0.2524491	0.6554097	HRG
cg20850981	0.567439	-213.184	-0.2524726	0.8199116	ZNF323
cg16998872	0.503002	-178.0796	-0.2525446	0.7555467	GYPE
cg16192029	0.6730579	-337.0802	-0.2525812	0.925639	ANKRD7
cg10210238	0.2020104	-155.0739	-0.2525983	0.4546087	CDKN2B
cg18986165	0.5445288	-198.8182	-0.2526736	0.7972023	SIGLEC12
cg15627025	0.6032982	-243.5974	-0.2527229	0.8560211	KIR3DL1
cg00371195	0.5182887	-185.2461	-0.2527658	0.7710544	F2
cg07471052	0.6531493	-304.6597	-0.2527781	0.9059274	CDK3
cg08453096	0.5730367	-218.0541	-0.2528557	0.8258924	ABCG5
cg27566805	0.2372499	-149.7773	-0.2529885	0.4902384	USH2A
cg00756887	0.4323953	-157.306	-0.2530658	0.685461	PVRL4
cg23894003	0.4339709	-157.8132	-0.2531915	0.6871625	FBS1
cg22585988	0.4331074	-157.9962	-0.2534388	0.6865462	PVRL4
cg15531099	0.2827344	-146.418	-0.2535197	0.5362542	LCE1D
cg25547939	0.5038506	-180.3246	-0.2535762	0.7574269	VEGFB
cg00269932	0.6429129	-293.0196	-0.2537166	0.8966295	LAIR2
cg12970084	0.4238267	-156.9409	-0.2540293	0.677856	ELF3

cg03973663	0.1778009	-162.2247	-0.254053	0.4318539	LYN
cg11003133	0.3084437	-146.3555	-0.2540583	0.562502	AIM2
cg17170504	0.3431727	-147.2805	-0.254204	0.5973768	HSPC065
cg11061975	0.292535	-146.9032	-0.2542445	0.5467796	SIRPB2
cg22247240	0.3545766	-148.1197	-0.2543222	0.6088988	C14orf115
cg10756887	0.17586	-163.1331	-0.254397	0.4302571	C22orf15
cg06489008	0.298704	-147.1498	-0.254586	0.55329	CST11
cg01469547	0.5554714	-209.7718	-0.2546248	0.8100963	OR5P3
cg06946880	0.4598412	-166.6137	-0.2546489	0.7144901	ATP6V1B1
cg11052143	0.1526964	-170.0933	-0.2546712	0.4073675	ALS2CR11
cg05985767	0.415	-156.2583	-0.2547208	0.6697208	ANPEP
cg19890739	0.4554051	-165.4964	-0.254743	0.7101481	Pfs2
cg24628744	0.149321	-171.2776	-0.2547508	0.4040718	H2AFY
cg20176648	0.2182727	-154.7911	-0.2548285	0.4731012	AQP1
cg08394377	0.42807	-159.0038	-0.2548371	0.6829071	RIMS3
cg06259570	0.3687991	-149.988	-0.2548638	0.6236629	MMP27
cg22855405	0.1590247	-168.4159	-0.2549037	0.4139284	TINAGL1
cg12493906	0.3041862	-147.4454	-0.2549134	0.5590996	MMP26
cg24818418	0.603891	-250.1646	-0.2549471	0.8588381	EGF
cg24429836	0.2048941	-157.3401	-0.2549679	0.459862	LDHD
cg13439299	0.5697222	-220.6469	-0.2550292	0.8247514	DNAJC5G
cg10248727	0.5555401	-210.7799	-0.255069	0.810609	LCN1
cg15423764	0.4011753	-154.5832	-0.2552068	0.6563821	GLYAT

cg22083047	0.2286583	-153.6587	-0.2552297	0.483888	PRICKLE2
cg09207718	0.311445	-148.0943	-0.2554663	0.5669113	CYP1A2
cg08356693	0.2835695	-148.9575	-0.2556447	0.5392143	ITLN1
cg00033773	0.53451	-199.6853	-0.2558874	0.7903975	MORG1
cg04457051	0.3489223	-149.9082	-0.2559904	0.6049128	SCOC
cg06720660	0.3472812	-149.8761	-0.2560412	0.6033224	RNASE6
cg03874127	0.4872675	-178.2598	-0.2561595	0.743427	ARMC2
cg00795812	0.135962	-177.7542	-0.256233	0.392195	PDCD1
cg06785429	0.2220495	-155.9299	-0.2562642	0.4783137	DCUN1D1
cg18565355	0.2042307	-159.2096	-0.2564237	0.4606544	RBM35A
cg13180098	0.385524	-154.0818	-0.256455	0.641979	RHO
cg15060813	0.2529652	-152.1702	-0.2564621	0.5094273	LRFN3
cg23580945	0.4933964	-181.2465	-0.2564725	0.7498689	FLJ43826
cg12654845	0.3867095	-154.3438	-0.2565289	0.6432385	CLDN2
cg25982743	0.1804704	-164.547	-0.2565446	0.437015	TIMP4
cg22228134	0.3244248	-149.7009	-0.2566245	0.5810493	GZMH
cg18565510	0.6542677	-320.5371	-0.2568153	0.911083	CENTB5
cg06353345	0.2657905	-151.5303	-0.2568645	0.522655	OR51B4
cg02706881	0.611258	-263.5045	-0.256933	0.8681911	C21orf123
cg00152644	0.5642594	-221.0842	-0.2569669	0.8212262	SPRR2E
cg24352688	0.3607013	-152.2103	-0.2570629	0.6177641	OFD1
cg25659818	0.3183608	-150.1537	-0.2570816	0.5754424	CCL4
cg21808053	0.2684898	-151.6534	-0.2571287	0.5256186	DIRAS3

cg27378216	0.3950092	-156.442	-0.257183	0.6521923	SETBP1
cg22456522	0.1806704	-165.4804	-0.2573619	0.4380323	LILRB3
cg15916061	0.2922589	-150.8834	-0.2574666	0.5497255	SLC17A4
cg15481539	0.5517882	-213.6882	-0.2575035	0.8092917	DEFA5
cg23595927	0.2654163	-152.5922	-0.2576953	0.5231116	MYL5
cg22971191	0.2590091	-153.2219	-0.2577669	0.516776	SLC10A2
cg02968557	0.5264727	-199.325	-0.2578936	0.7843664	HSPB9
cg02966851	0.2296483	-156.774	-0.2579099	0.4875581	C6orf149
cg00622552	0.1214863	-185.5493	-0.2579297	0.379416	ODF3L1
cg22909609	0.2440322	-155.0217	-0.2579843	0.5020164	ITGBL1
cg12374577	0.4177622	-161.5847	-0.2580035	0.6757657	UGT2A3
cg06614002	0.5825242	-238.2831	-0.2580208	0.840545	SOX10
cg14587868	0.3882876	-156.6776	-0.2580377	0.6463253	TGM1
cg05556202	0.4999642	-186.807	-0.2580551	0.7580193	TM4SF19
cg02124291	0.4511847	-169.6582	-0.25808	0.7092648	OR7A5
cg13434852	0.6016939	-256.5412	-0.2580939	0.8597878	DOCK1
cg26980692	0.1935644	-163.4629	-0.2581301	0.4516945	SLC15A3
cg16964535	0.6513815	-320.5988	-0.2581326	0.9095141	DNAJC5G
cg09492887	0.09012207	-200.7354	-0.2581365	0.3482586	SLC26A5
cg16446783	0.3969857	-158.2406	-0.2582435	0.6552292	MRGPRX4
cg20649991	0.477111	-178.2	-0.2582665	0.7353776	LILRB5
cg21717724	0.3233055	-151.8034	-0.2582953	0.5816008	PSMD5
cg20573420	0.6033525	-258.9243	-0.2583285	0.861681	FLJ38451

cg01772980	0.3656408	-154.4096	-0.2583301	0.6239709	SCGB1D1
cg24812523	0.3756594	-155.5614	-0.2583795	0.6340389	AKAP6
cg16777782	0.6436204	-310.0086	-0.2585308	0.9021512	CDH13
cg04254119	0.5590276	-220.9576	-0.2585472	0.8175747	AKAP3
cg22264436	0.4881913	-183.141	-0.2587015	0.7468928	SOST
cg14345676	0.3367259	-153.2662	-0.2590377	0.5957636	HRH2
cg03044435	0.5063699	-191.5975	-0.2590663	0.7654362	FLJ35816
cg16540704	0.523401	-200.1323	-0.2591123	0.7825133	ASZ1
cg24623694	0.1983321	-163.7629	-0.2592101	0.4575422	PRX
cg16377880	0.3379869	-153.8532	-0.2594252	0.5974121	CYP4F3
cg10883352	0.4129232	-163.1245	-0.2596801	0.6726032	
cg11811840	0.3627725	-156.0568	-0.2597218	0.6224942	UGT1A1
cg15669228	0.3269805	-153.9901	-0.2598702	0.5868507	IFNA8
cg24884084	0.1706301	-171.1956	-0.2598982	0.4305283	SPRR1B
cg22341310	0.1693448	-171.6439	-0.2599888	0.4293335	ZNF541
cg11787522	0.2771168	-155.0676	-0.2602692	0.5373861	STRA6
cg14942312	0.422022	-165.8699	-0.2602745	0.6822965	GPR119
cg15430659	0.452902	-173.8247	-0.2603494	0.7132514	CNNM1
cg12616487	0.4237459	-166.6971	-0.2605781	0.684324	EML3
cg19903229	0.2747543	-155.6937	-0.2606489	0.5354032	C14orf105
cg03557698	0.608444	-271.4076	-0.2607685	0.8692125	C1orf177
cg06436504	0.4088246	-164.0249	-0.2608162	0.6696407	DOC1
cg21312148	0.2696252	-156.2913	-0.2608776	0.5305029	LCE2D

cg01120308	0.1164448	-191.6553	-0.2610619	0.3775067	PICALM
cg24237576	0.5282273	-206.9176	-0.2611052	0.7893326	DNASE1L2
cg08402568	0.3932329	-161.8064	-0.2611268	0.6543597	MGC34647
cg00311768	0.3580097	-157.5502	-0.2611521	0.6191617	TSTA3
cg14324675	0.348287	-157.0583	-0.261349	0.609636	LST1
cg10071275	0.3189432	-156.0668	-0.2615973	0.5805405	MYT1
cg25994725	0.3004022	-156.0392	-0.2616243	0.5620265	C6orf81
cg10159529	0.3996575	-163.623	-0.2616515	0.661309	IL5RA
cg07676849	0.3237547	-156.5785	-0.2619025	0.5856572	FOLR3
cg18129786	0.5479009	-220.9745	-0.2619662	0.8098671	ZNF445
cg17894008	0.5570477	-227.5238	-0.2619733	0.819021	NACAL
cg26353877	0.4914695	-190.6994	-0.2621084	0.7535778	APCS
cg12019109	0.3270956	-157.0418	-0.2621723	0.5892678	AZGP1
cg20322977	0.2431409	-160.3095	-0.2621808	0.5053217	CYP26C1
cg17405586	0.3774604	-161.1872	-0.2622471	0.6397075	KRT1
cg01031251	0.3499802	-158.497	-0.2623202	0.6123005	RPS6KA1
cg24840099	0.2982755	-157.3619	-0.2626455	0.5609211	MSX1
cg02989940	0.311698	-157.3619	-0.2626802	0.5743782	ERAF
cg17582777	0.3567047	-159.5515	-0.2626898	0.6193945	EFNA3
cg14162076	0.5668968	-237.2731	-0.2628566	0.8297534	CLEC4D
cg09425228	0.4075281	-166.8524	-0.2628839	0.6704121	CCL20
cg20399252	0.5988966	-267.2025	-0.2629012	0.8617978	EBPL
cg25214346	0.391118	-164.2482	-0.2630481	0.654166	NR1I3

cg12943082	0.4812468	-188.3428	-0.2631178	0.7443646	CCL26
cg25915982	0.5126151	-202.7966	-0.263251	0.7758661	GRB10
cg06811800	0.4120362	-168.3735	-0.2632884	0.6753246	ATP4B
cg08495878	0.6010603	-271.1706	-0.2634914	0.8645517	SERPINA4
cg03294491	0.1636743	-177.6901	-0.2635203	0.4271945	SMAD2
cg04587829	0.3043205	-158.5574	-0.2636078	0.5679283	FN3K
cg08474603	0.4026768	-167.0746	-0.2636428	0.6663196	CRP
cg09528351	0.4277885	-172.5147	-0.2637101	0.6914986	PIK3R5
cg24698533	0.4192035	-170.7447	-0.2638364	0.6830398	LYZL2
cg00644033	0.6535878	-345.9298	-0.2639786	0.9175664	MUC3B
cg06810461	0.416425	-170.4575	-0.2640451	0.6804701	AGRP
cg05755354	0.2947246	-159.3728	-0.2641511	0.5588757	FRMD4A
cg14366490	0.3732537	-163.5848	-0.2643045	0.6375582	TXNL6
cg14550066	0.2696154	-160.7108	-0.2643758	0.5339913	NCR1
cg03752087	0.4214114	-172.2636	-0.2645043	0.6859157	CASP14
cg24489015	0.3302807	-160.3095	-0.2645249	0.5948056	LPO
cg07039113	0.5608988	-236.7306	-0.2645844	0.8254831	S100A9
cg00518911	0.249284	-162.772	-0.2646827	0.5139667	HOXA10
cg01036012	0.6059142	-280.3795	-0.2647408	0.8706549	TUBB8
cg19226099	0.2884741	-160.4188	-0.2648408	0.5533149	MC3R
cg20352371	0.3509021	-162.1254	-0.2649154	0.6158174	PDZRN4
cg20357806	0.4391527	-177.5147	-0.2649513	0.7041041	PPBP
cg11161873	0.2704292	-161.5513	-0.2650728	0.535502	FLJ39575

cg07443748	0.3114133	-160.5329	-0.2650903	0.5765036	CESK1
cg01550148	0.2822922	-161.0296	-0.2651389	0.5474311	H2AFY
cg19111262	0.2588242	-162.493	-0.2651569	0.5239811	IGSF9
cg24309555	0.3606786	-164.049	-0.2656473	0.6263258	APOB
cg17095731	0.3772435	-166.049	-0.2656697	0.6429132	LRP8
cg14696870	0.3611942	-164.1582	-0.2656901	0.6268843	FCER1A
cg06851207	0.1817789	-175.657	-0.2657612	0.4475401	FLJ10781
cg24919884	0.3921971	-168.4451	-0.2657709	0.657968	ARHGEF16
cg15140807	0.4758484	-191.1178	-0.2657981	0.7416465	FLJ31222
cg04245402	0.2326841	-166.1211	-0.2658012	0.4984854	C19orf21
cg04968473	0.2919694	-161.6147	-0.2658303	0.5577998	CYP1A2
cg01072821	0.3624893	-164.5552	-0.2658773	0.6283666	UNQ9391
cg00071250	0.3403454	-162.8461	-0.2660013	0.6063467	FASLG
cg05955301	0.2446785	-165.0489	-0.2661064	0.5107849	PRELP
cg14620221	0.4798079	-193.3193	-0.2661356	0.7459434	OR8B8
cg07207789	0.328779	-162.5331	-0.2662339	0.5950129	CRISPLD2
cg09272256	0.3490272	-163.9771	-0.2663559	0.6153831	AKR1C4
cg10821722	0.6574079	-348.7749	-0.2663966	0.9238046	MAP4K2
cg18463686	0.2518802	-164.9586	-0.2666101	0.5184903	CLEC5A
cg19465374	0.5080917	-207.6229	-0.2667928	0.7748845	AZGP1
cg05190718	0.4295479	-177.9907	-0.2668292	0.696377	CASQ2
cg12552392	0.3544778	-165.3413	-0.2669983	0.6214761	NFS1
cg12732155	0.1942209	-174.3994	-0.2670596	0.4612805	LAPTM5

cg21754343	0.32732	-163.6284	-0.2670801	0.5944	LCE2B
cg15516226	0.5310134	-221.4043	-0.2671255	0.7981389	BTNL9
cg12113132	0.2803669	-163.798	-0.2672217	0.5475886	CCNDBP1
cg13883681	0.2399462	-167.0286	-0.267244	0.5071902	GOT2
cg27257987	0.3797505	-168.731	-0.2672704	0.6470209	PSG4
cg04962134	0.3327641	-164.1511	-0.267277	0.600041	TRIM51
cg18533225	0.4219054	-176.9467	-0.2673504	0.6892558	KLHDC7B
cg07072643	0.2930062	-163.7731	-0.2674938	0.5605	EMR3
cg01663968	0.07585751	-221.3183	-0.2676601	0.3435176	CTSZ
cg08555657	0.4771605	-195.2368	-0.2677554	0.7449159	SPRR2E
cg00436603	0.2807162	-164.6373	-0.2678827	0.5485989	CYP2E1
cg07531356	0.392355	-172.0219	-0.2681336	0.6604886	INSL6
cg09748975	0.4745896	-194.9207	-0.2681431	0.7427327	MSX1
cg14414534	0.6233838	-313.3767	-0.2681764	0.8915602	TMOD1
cg08887581	0.4280819	-179.8463	-0.2681815	0.6962634	C1orf64
cg24888049	0.189629	-177.0115	-0.2682895	0.4579185	FES
cg10837843	0.08302791	-217.9225	-0.2683011	0.351329	DUSP1
cg06906435	0.2675507	-166.2255	-0.2685626	0.5361133	FLJ25773
cg02717866	0.3828449	-171.1732	-0.2685947	0.6514395	FLJ32771
cg18056600	0.09626582	-211.1541	-0.2686744	0.3649402	ZMYND15
cg03712237	0.5152676	-215.4133	-0.2686816	0.7839491	SSX2
cg00350478	0.3358716	-166.4469	-0.268807	0.6046786	FRMD1
cg00623593	0.6152242	-304.6285	-0.2688596	0.8840837	DOCK1

cg24642820	0.4458232	-186.2722	-0.2690049	0.714828	NUP210
cg07977490	0.419591	-179.4391	-0.2692386	0.6888297	C16orf45
cg15560337	0.4825774	-200.3478	-0.2692816	0.7518591	HYAL4
cg16391792	0.4862956	-202.0144	-0.269289	0.7555845	LIPC
cg09458237	0.4247538	-181.2179	-0.2695282	0.694282	HSPA12B
cg18490846	0.2563291	-168.4573	-0.2696337	0.5259629	C17orf73
cg16242770	0.4925787	-205.6065	-0.2696422	0.7622209	KRTAP17-1
cg06531741	0.2660533	-167.9032	-0.2697713	0.5358245	HTR3B
cg01484156	0.2803335	-167.1496	-0.2697912	0.5501247	NCALD
cg05670596	0.2179748	-173.4391	-0.2698414	0.4878161	CCRL2
cg19000186	0.3986985	-176.0467	-0.2699675	0.668666	CNGA1
cg16016036	0.5619254	-251.0972	-0.270018	0.8319434	TPO
cg11070419	0.2778617	-167.75	-0.2701516	0.5480133	C4BPA
cg10052840	0.2249369	-172.8146	-0.2701882	0.4951251	SEMA6B
cg15447486	0.4318113	-184.222	-0.2702301	0.7020414	GPR109B
cg02130905	0.3364677	-168.5984	-0.2703236	0.6067913	STMN4
cg12188416	0.2293814	-172.548	-0.2704749	0.4998563	TP73L
cg09555879	0.510716	-216.6634	-0.2704886	0.7812046	GRIK5
cg11435943	0.2486187	-170.4448	-0.270596	0.5192146	SERPINB12
cg24387818	0.2096477	-175.9198	-0.2706122	0.48026	POF1B
cg19242268	0.2711027	-168.7517	-0.2706534	0.541756	TCEA2
cg04457794	0.5119018	-217.8939	-0.2707668	0.7826686	CTSE
cg09076584	0.3999365	-177.9558	-0.27104	0.6709765	FLJ25006

cg26504906	0.2113923	-176.61	-0.2713901	0.4827824	PRSS16
cg12639234	0.2864751	-169.1545	-0.2714374	0.5579125	NAT2
cg02601403	0.4978174	-212.038	-0.2715607	0.7693781	TBC1D3C
cg09467501	0.1005493	-212.8221	-0.2715639	0.3721131	PYY
cg10464775	0.3997396	-178.7914	-0.2715966	0.6713361	LAMP1
cg26822241	0.4263318	-185.0558	-0.2716095	0.6979414	CYP2C9
cg22970435	0.08716162	-220.0847	-0.2716898	0.3588514	SPATS1
cg21624282	0.5265132	-228.8622	-0.2717234	0.7982366	LOC122258
cg05564657	0.6084296	-305.0659	-0.2717645	0.880194	AADAC
cg17699374	0.1891924	-181.7114	-0.2718365	0.4610289	MGC35206
cg01785568	0.2787494	-170.0277	-0.2718783	0.5506277	MSX1
cg13474734	0.4721592	-200.8882	-0.2718818	0.7440411	FLJ43339
cg17500962	0.3914953	-177.6756	-0.2718844	0.6633798	C16orf47
cg05636175	0.1582367	-190.0912	-0.2719131	0.4301497	TNFRSF10C
cg18434152	0.4167968	-183.2372	-0.271999	0.6887958	PROK1
cg24489034	0.4189426	-183.9212	-0.2720881	0.6910307	LW-1
cg16268563	0.4318684	-187.3724	-0.2720946	0.703963	ATP6V1G2
cg01861509	0.4052117	-180.863	-0.2721553	0.6773669	SPOCK2
cg04189838	0.4480734	-192.6556	-0.2722377	0.7203111	CYP2C19
cg16792160	0.286668	-170.3067	-0.2722775	0.5589455	ASAH2
cg15741706	0.4226702	-185.3895	-0.2723919	0.6950621	CXorf48
cg21283680	0.3780369	-176.281	-0.2724587	0.6504956	SH3BP5
cg00601486	0.6200342	-323.9055	-0.2727015	0.8927357	H1T2

cg09715672	0.1938746	-181.8201	-0.2727247	0.4665993	C10orf116
cg00687674	0.2005213	-180.5307	-0.2728021	0.4733233	TMEM84
cg17741572	0.5241007	-230.0571	-0.2729389	0.7970396	CFB
cg27329371	0.2790008	-171.642	-0.2730939	0.5520948	ALDH3A1
cg19384697	0.227472	-176.2999	-0.2731118	0.5005838	UPK3B
cg18098286	0.3532396	-174.2865	-0.2732823	0.6265219	UGT1A10
cg21044104	0.3347231	-172.8777	-0.2734283	0.6081514	LYZL4
cg26922202	0.3205391	-172.1947	-0.2734801	0.5940192	OR2S2
cg25141995	0.246901	-174.4577	-0.2735152	0.5204162	VDAC1
cg15379858	0.6152232	-321.5026	-0.2740132	0.8892364	ChGn
cg13960126	0.3668105	-177.6384	-0.2744171	0.6412276	CRB3
cg24642468	0.243867	-176.0751	-0.2744782	0.5183452	MGC33367
cg18389810	0.5668723	-267.6636	-0.2745928	0.8414651	C14orf8
cg03854071	0.6465315	-348.7749	-0.2748145	0.9213461	NDUFA13
cg13281868	0.3865	-181.8766	-0.2751407	0.6616407	C6orf142
cg09995854	0.396209	-183.7418	-0.2751445	0.6713535	IL1F8
cg17204557	0.4483218	-198.0793	-0.2752447	0.7235665	FLJ14816
cg12811135	0.6041667	-311.1307	-0.2753757	0.8795425	FLJ25369
cg02813121	0.5510908	-255.8511	-0.2754324	0.8265232	S100A12
cg10677144	0.4356591	-194.5521	-0.2755885	0.7112477	MYOM1
cg00226923	0.4878011	-215.4533	-0.275713	0.7635141	FGD2
cg22478614	0.2827209	-175.1158	-0.275718	0.5584389	DEFB4
cg17720233	0.3869389	-183.0923	-0.2758657	0.6628047	TNFAIP8L2

cg23278885	0.4291568	-193.2157	-0.2759313	0.7050881	TGM6
cg24910675	0.1380422	-202.4209	-0.2760689	0.4141111	ENG
cg13521229	0.2769772	-175.9174	-0.2761424	0.5531197	JOSD2
cg21825364	0.6334008	-348.7749	-0.2765951	0.9099959	VCY
cg10334928	0.5543331	-261.8444	-0.2766593	0.8309923	STON2
cg22762309	0.4616982	-206.1234	-0.276868	0.7385662	OTUD6A
cg10368842	0.3391127	-178.5051	-0.2770415	0.6161542	C10orf81
cg16463460	0.2043725	-185.4626	-0.2771217	0.4814942	WT1
cg07193504	0.3392852	-178.649	-0.2771287	0.6164138	SRP9
cg04337944	0.2011278	-186.6851	-0.2775713	0.4786991	FBLN1
cg27160701	0.30349	-177.6126	-0.2776021	0.5810921	SBEM
cg05254747	0.4621343	-207.7363	-0.2776291	0.7397634	SLC39A14
cg24693053	0.192662	-188.6788	-0.2777409	0.4704029	MFSD7
cg23324787	0.3393727	-179.747	-0.2778433	0.617216	RAG2
cg15210427	0.2369719	-181.4283	-0.2778605	0.5148324	CST9L
cg18783781	0.4928145	-222.6393	-0.2779713	0.7707858	MGC4399
cg18223379	0.2411609	-181.3391	-0.2781435	0.5193044	BPIL3
cg26928682	0.5305565	-246.6617	-0.2781646	0.8087211	TREML2
cg26790059	0.4652608	-210.3069	-0.2782958	0.7435566	MGC27121
cg18204685	0.4075817	-191.8021	-0.2785072	0.686089	BTD
cg26264314	0.4707078	-213.52	-0.2787257	0.7494335	NALP5
cg16020904	0.4284619	-197.9989	-0.2788007	0.7072626	SPATA20
cg24825722	0.3168913	-179.747	-0.2788262	0.5957175	ACADVL

cg04655481	0.4230804	-196.6279	-0.2789128	0.7019932	GPR21
cg16358738	0.4454297	-203.9704	-0.2790205	0.7244502	AGXT
cg26628847	0.2255951	-184.539	-0.2790701	0.5046653	PIP
cg11346450	0.4475769	-205.0995	-0.2792021	0.726779	UGT1A3
cg14511156	0.2132646	-186.8547	-0.2793763	0.4926409	OSCAR
cg19201019	0.4817283	-219.9749	-0.2793966	0.7611248	B3GALT7
cg16626670	0.3663262	-185.9766	-0.2798799	0.6462061	CLEC4G
cg19481686	0.3499645	-183.8515	-0.279885	0.6298494	CDKN2B
cg16356516	0.5516476	-268.1291	-0.2799428	0.8315904	EXOSC6
cg01325515	0.5108501	-237.5609	-0.2799757	0.7908258	CTAG2
cg19241311	0.3127278	-181.4185	-0.2800826	0.5928104	DEFB123
cg04431776	0.5219554	-245.3951	-0.2801816	0.802137	GAGE2
cg07017706	0.4370141	-203.569	-0.2803862	0.7174003	K6IRS3
cg05810550	0.4301205	-201.3738	-0.2804131	0.7105336	DEFB106A
cg06145357	0.3998028	-193.2157	-0.2804367	0.6802394	MAGEA8
cg20622019	0.4627329	-213.7105	-0.280598	0.7433308	ADA
cg01443630	0.5292528	-251.6591	-0.2806314	0.8098843	CLCN7
cg20676303	0.5985171	-321.3517	-0.2807832	0.8793003	GAGE7B
cg14652095	0.2302759	-186.2722	-0.2808143	0.5110902	HIST1H1A
cg18809535	0.5640552	-282.3948	-0.2808743	0.8449295	LDHAL6B
cg17974185	0.2800422	-182.4239	-0.2808802	0.5609224	CTNNBL1
cg22772878	0.180457	-196.0449	-0.2810627	0.4615197	DIRAS1
cg13758677	0.4682764	-217.1134	-0.2811027	0.7493791	GAGE4

cg03517000	0.4679686	-218.0195	-0.2816354	0.749604	GSTA2
cg10938286	0.563175	-283.8617	-0.28171	0.844885	CST2
cg18396533	0.1371744	-210.4338	-0.2817587	0.4189331	DYDC1
cg23131950	0.4402527	-208.0591	-0.2822071	0.7224598	AP2S1
cg01970325	0.2555624	-185.8101	-0.2823845	0.5379469	NELF
cg21275690	0.2406928	-187.37	-0.2825241	0.5232169	WDR58
cg06827038	0.367606	-190.4317	-0.2825595	0.6501654	WFIKKN2
cg15996947	0.3350675	-186.4482	-0.2825771	0.6176445	L2HGDH
cg24027679	0.4933456	-232.9866	-0.2826183	0.7759639	SLC2A7
cg00896220	0.4218163	-202.9255	-0.2827085	0.7045248	CCL4L2
cg00520135	0.5727528	-296.9166	-0.2827659	0.8555187	TPM1
cg23829949	0.2983484	-185.4104	-0.2830752	0.5814235	ZNF238
cg19756068	0.3499673	-188.8913	-0.2831609	0.6331282	CYP2B6
cg08886154	0.2624986	-186.4456	-0.2831836	0.5456822	PAX4
cg00895324	0.4227516	-204.2795	-0.28331	0.7060616	PCP4
cg13019092	0.4493848	-213.7025	-0.2833937	0.7327785	PDZK1
cg24430616	0.3372833	-188.0298	-0.2834853	0.6207685	ENTPD4
cg08996986	0.3946352	-197.1626	-0.2834912	0.6781263	EPS8L1
cg07126559	0.4754681	-226.1104	-0.2838418	0.7593099	SGCG
cg08424423	0.5169744	-250.7868	-0.283903	0.8008775	CDSN
cg27090216	0.07403571	-245.0354	-0.2840547	0.3580904	TNFRSF10C
cg03544379	0.2082117	-194.2422	-0.2841081	0.4923198	OR7C2
cg05093686	0.19253	-197.3833	-0.2841108	0.4766409	MAB21L1

cg03169180	0.34089	-189.4122	-0.284142	0.625032	NLGN2
cg05559445	0.1945909	-197.4358	-0.2844748	0.4790657	CDKN1C
cg07595943	0.632269	-348.7749	-0.2845235	0.9167925	LOC161931
cg07339138	0.288756	-187.5802	-0.2845972	0.5733532	CCDC13
cg00501366	0.3043773	-187.7618	-0.2846103	0.5889876	ALOX12B
cg26111757	0.4034189	-201.363	-0.2847139	0.6881328	C20orf185
cg15005385	0.2844271	-188.0111	-0.284863	0.56929	CCL3L1
cg27513764	0.3786214	-196.1573	-0.2848996	0.6635211	EFCAB3
cg18780284	0.396364	-200.1506	-0.2850296	0.6813936	SPRR1B
cg21505334	0.4449533	-215.2579	-0.2850858	0.7300391	CEACAM5
cg18219418	0.4494605	-217.0393	-0.2851037	0.7345642	PARP6
cg22194129	0.3040097	-188.6096	-0.2851887	0.5891984	CLEC4C
cg22253945	0.4985378	-242.0048	-0.2852765	0.7838143	GPR45
cg02311163	0.3306396	-190.375	-0.2853699	0.6160095	SEMG2
cg12554476	0.4005704	-202.0862	-0.2855479	0.6861182	GP2
cg13259290	0.4400573	-214.4258	-0.2856266	0.7256839	CSF2
cg11984608	0.511614	-251.2101	-0.2856354	0.7972494	CLDN16
cg20047055	0.3809481	-198.1297	-0.2858366	0.6667847	KLRC1
cg08878744	0.2294291	-193.4751	-0.2859084	0.5153375	LCE1B
cg26523005	0.261979	-190.7536	-0.2861734	0.5481524	ZNF662
cg17240454	0.5137714	-254.0707	-0.2861901	0.7999614	SPDEF
cg25531166	0.4800046	-233.9593	-0.2864294	0.766434	CTAG1B
cg17051440	0.3726527	-197.8495	-0.2866014	0.6592541	CLDN2

cg01507173	0.3475056	-194.1785	-0.2867039	0.6342095	IL1F5
cg24477636	0.2754288	-190.982	-0.2867494	0.5621782	OR10H1
cg11750883	0.272415	-191.2468	-0.286862	0.5592771	C1orf42
cg07879977	0.2381216	-193.8477	-0.2869206	0.5250422	OR1F1
cg14117297	0.2381546	-193.9614	-0.2870043	0.5251589	MGC23244
cg13126790	0.3927585	-202.7717	-0.2870359	0.6797944	FLJ27255
cg05112299	0.2110282	-197.8973	-0.2870872	0.4981154	OR7A17
cg26063872	0.3553148	-195.8396	-0.2870894	0.6424041	DEFB123
cg07026910	0.4373119	-216.2631	-0.2871168	0.7244287	INPP5D
cg02192965	0.4033369	-205.6024	-0.2871362	0.690473	SLC3A1
cg23244913	0.08041057	-245.63	-0.2872761	0.3676866	HCG9
cg07525077	0.2865863	-191.6073	-0.2873208	0.5739071	RNASE3
cg01442426	0.4813456	-236.8736	-0.2874407	0.7687863	XCR1
cg06736444	0.2546618	-193.0956	-0.2874486	0.5421104	SRRM2
cg27020690	0.06568517	-255.3986	-0.2875087	0.3531938	TERC
cg13608094	0.5766066	-315.6033	-0.2875227	0.8641294	CCND1
cg09027725	0.3415252	-195.2816	-0.2878439	0.6293691	COX4I2
cg15422147	0.4002661	-206.6707	-0.2881874	0.6884536	SERPINB5
cg04739485	0.3302805	-195.07	-0.2884274	0.618708	MLXIP
cg22088368	0.245225	-195.3396	-0.2884772	0.5337022	MGC35206
cg27291231	0.4060988	-209.1233	-0.2886999	0.6947987	SSNA1
cg12391921	0.3254905	-195.4158	-0.2889148	0.6144053	ITGB1BP2
cg18766847	0.345991	-197.7215	-0.2890399	0.6350309	ACMSD

cg00718513	0.196113	-203.6907	-0.2891917	0.4853047	
cg21372914	0.424763	-215.9105	-0.2892652	0.7140282	CLEC4M
cg25552889	0.5020998	-254.1977	-0.2895663	0.7916662	C3orf40
cg06437862	0.4705487	-235.8024	-0.2895817	0.7601305	TUBA2
cg08314660	0.5579651	-301.8652	-0.2897904	0.8477555	PKP3
cg26705561	0.2526331	-196.6723	-0.2898253	0.5424584	SEC31L2
cg15903395	0.3649577	-202.1404	-0.2900175	0.6549752	FLJ25369
cg26189983	0.06063579	-262.6922	-0.2901096	0.3507454	TNFRSF1B
cg03104936	0.3481472	-199.7745	-0.2901464	0.6382935	GRB10
cg14297029	0.5513822	-296.7888	-0.2903346	0.8417169	SSTR3
cg06275635	0.2684315	-196.6169	-0.2904112	0.5588427	PGLYRP3
cg10213812	0.5723678	-320.4406	-0.2906434	0.8630112	FOXN1
cg09546307	0.3836251	-207.1632	-0.2907714	0.6743965	CLEC4D
cg12970081	0.2848725	-196.8374	-0.2908257	0.5756982	GPR32
cg18242139	0.4225373	-218.1394	-0.2908734	0.7134107	ELAVL4
cg07790638	0.3681438	-204.1952	-0.290905	0.6590487	LOC91431
cg24765446	0.3292072	-199.1071	-0.2910689	0.6202761	WFDC6
cg22937320	0.4164319	-216.6121	-0.2910962	0.7075281	C9orf138
cg03364781	0.3899066	-209.453	-0.2912555	0.6811622	ALPK1
cg09299388	0.4058265	-213.692	-0.2912609	0.6970874	PGK2
cg10746737	0.4138977	-216.1605	-0.2912852	0.7051829	HLA-DRB5
cg08815403	0.2833803	-197.5639	-0.2913038	0.5746841	HSD17B13
cg04705866	0.4023625	-213.1093	-0.2914674	0.6938299	GZMK

cg23889010	0.273544	-198.0577	-0.2915275	0.5650716	SLPI
cg02844051	0.444601	-228.0187	-0.2916336	0.7362346	ZD52F10
cg27132814	0.3040837	-198.6369	-0.2918154	0.5958991	C20orf79
cg14960043	0.1145439	-234.0244	-0.2918294	0.4063734	DEF6
cg00321478	0.3645331	-205.3564	-0.2919638	0.6564969	CRB1
cg21885995	0.3278359	-200.4066	-0.2919695	0.6198054	SRP68
cg08684473	0.456141	-234.4062	-0.2922423	0.7483833	LILRB5
cg24735937	0.3776745	-208.5378	-0.292318	0.6699926	CLTB
cg22340747	0.3972309	-213.8742	-0.2926865	0.6899173	GATM
cg02184413	0.3759092	-208.9536	-0.2927763	0.6686856	VNN1
cg25043279	0.392754	-213.0235	-0.2928424	0.6855964	C7orf33
cg24607398	0.2289655	-203.4643	-0.2928538	0.5218193	MLH1
cg24355048	0.3444321	-203.9586	-0.2930084	0.6374406	CTSG
cg26581729	0.1594657	-218.7068	-0.2931616	0.4526273	NPDC1
cg08157292	0.2875068	-201.4897	-0.2939066	0.5814134	PPP1R7
cg12351042	0.3540932	-206.9507	-0.293949	0.6480422	OR2B2
cg15542496	0.433926	-229.1028	-0.2943294	0.7282554	PIP
cg19876838	0.4309168	-228.0695	-0.2944055	0.7253224	KCNJ14
cg01515887	0.43412	-229.7781	-0.2946239	0.7287439	SAA2
cg25762706	0.2744652	-202.9089	-0.2947566	0.5692218	STMN4
cg25177139	0.4987257	-265.106	-0.2950055	0.7937313	SLC10A6
cg04953015	0.4183709	-225.2698	-0.2953083	0.7136792	CHRNA2
cg06270401	0.4692582	-248.2442	-0.2955773	0.7648356	DYRK4

cg16122592	0.3448111	-208.5577	-0.2957437	0.6405548	MAGEB6
cg10894512	0.3381223	-207.8588	-0.2958564	0.6339787	ACTA2
cg08728865	0.4955375	-265.5357	-0.2960736	0.7916111	NALP7
cg26309498	0.4998764	-268.6193	-0.296116	0.7959924	EDAR
cg20485165	0.351839	-210.5687	-0.2963197	0.6481587	WFDC12
cg14826683	0.2439201	-207.6514	-0.2967673	0.5406874	SPRR2D
cg11884243	0.4962993	-267.8994	-0.2968309	0.7931302	FCN2
cg00594952	0.2681084	-206.3009	-0.2968436	0.564952	RIMS3
cg12385643	0.5306451	-296.6125	-0.297254	0.8278991	UGT1A6
cg23807646	0.299449	-207.4817	-0.2975548	0.5970038	SLC26A8
cg04567009	0.2945365	-207.3489	-0.2975717	0.5921081	FCGR3B
cg24331162	0.4167991	-229.3218	-0.2976643	0.7144635	SYT8
cg20311730	0.3301173	-210.391	-0.297957	0.6280743	NALP10
cg24735489	0.3264592	-210.1205	-0.2980265	0.6244857	CDSN
cg10275770	0.1418907	-231.9794	-0.2981641	0.4400549	ICAM2
cg07597976	0.2868437	-208.5621	-0.2984506	0.5852943	CD19
cg09414535	0.3050535	-209.453	-0.2986448	0.6036983	GRIP1
cg19814116	0.3197477	-210.6377	-0.2987388	0.6184865	KCNAB2
cg25013053	0.3983355	-225.6137	-0.2988573	0.6971928	UNC45B
cg07484827	0.2010359	-217.9637	-0.2997178	0.5007538	CHRNA10
cg00689010	0.3544577	-217.1094	-0.299865	0.6543227	NCSTN
cg01568736	0.4397594	-243.0704	-0.2999135	0.7396729	SERPINB7
cg24857545	0.3778389	-222.0903	-0.2999452	0.6777841	GDI1

cg19787037	0.2486239	-212.3845	-0.3001315	0.5487555	SPAG11
cg01837574	0.2350105	-213.6321	-0.3001465	0.535157	TRAPPC1
cg12951282	0.3102588	-212.3048	-0.300186	0.6104448	ASGR2
cg01657380	0.3695983	-220.801	-0.3002739	0.6698722	NPFF
cg02595219	0.1629007	-228.2505	-0.3003815	0.4632823	KCNE3
cg24516901	0.1643419	-227.8892	-0.300416	0.4647579	FLJ22746
cg23881725	0.1290403	-240.6183	-0.3006434	0.4296838	DLEC1
cg23412777	0.3415281	-216.9855	-0.3009939	0.6425221	PYGO1
cg03087937	0.385405	-226.4005	-0.3012088	0.6866138	MUC15
cg01423840	0.4147469	-235.7854	-0.3012819	0.7160288	FATE1
cg02192520	0.3836895	-226.5977	-0.3015588	0.6852483	RDH5
cg02910574	0.5941569	-348.7749	-0.3016344	0.8957913	PCOLN3
cg15485859	0.4319783	-243.3994	-0.3016829	0.7336613	C1orf116
cg03609102	0.2694587	-213.8795	-0.3017732	0.5712318	MUC5B
cg04323365	0.4421142	-248.6726	-0.3020498	0.744164	GJB1
cg07711097	0.3607093	-222.5272	-0.3022776	0.6629869	GML
cg14120879	0.4525312	-254.4807	-0.3023432	0.7548744	DEFB105A
cg00367281	0.331255	-218.5727	-0.3027655	0.6340206	CHRNB3
cg14662756	0.3419178	-220.1774	-0.3028201	0.6447378	NPFF
cg12334759	0.3951156	-232.3406	-0.3028546	0.6979702	C19orf19
cg15589427	0.4314269	-246.165	-0.3031134	0.7345403	MUC4
cg22190114	0.4119118	-239.1468	-0.3034697	0.7153814	NALP8
cg06832950	0.4723859	-268.7228	-0.3036266	0.7760125	SPG3A

cg18797282	0.5953857	-348.7749	-0.3036309	0.8990166	CYB561D2
cg00819362	0.466608	-265.5596	-0.3037535	0.7703615	CLIPR-59
cg07115820	0.2731039	-217.1794	-0.3039018	0.5770056	EPX
cg16986846	0.2885061	-217.3856	-0.3039656	0.5924717	SCGB2A1
cg01530101	0.3236629	-220.347	-0.3043205	0.6279835	KCNQ1DN
cg05912121	0.3555444	-225.4672	-0.3044579	0.6600023	TH
cg22022041	0.4443298	-256.1099	-0.3049418	0.7492716	CCR9
cg10127415	0.2211309	-222.8673	-0.3051186	0.5262495	MAGEB6
cg23776892	0.4715109	-271.8214	-0.3051617	0.7766726	MAGEA1
cg21750887	0.3538496	-227.4035	-0.3057028	0.6595525	CPA6
cg14284171	0.5185962	-309.479	-0.3057945	0.8243908	SSX4
cg17568996	0.1752705	-233.1097	-0.3058789	0.4811494	NFAM1
cg15743985	0.3984907	-239.7922	-0.3061033	0.704594	CD22
cg07412254	0.3247169	-224.75	-0.3067812	0.631498	FLJ14816
cg06980053	0.1829755	-232.7939	-0.3069162	0.4898917	RASSF1
cg15417244	0.4081542	-245.0354	-0.3070277	0.7151818	PDZRN4
cg13745346	0.3691884	-233.7293	-0.3072953	0.6764838	CBFA2T3
cg14845091	0.3173621	-224.8275	-0.3073019	0.624664	ADPRHL1
cg04488758	0.3539637	-230.9232	-0.307604	0.6615677	USP44
cg24824840	0.4822708	-285.8018	-0.3078383	0.790109	SHANK1
cg01726767	0.3104739	-225.4662	-0.3080482	0.6185221	LALBA
cg26583078	0.3529527	-231.7403	-0.3081514	0.6611041	SORBS2
cg14722162	0.3435368	-230.1602	-0.3082675	0.6518043	C5orf20

cg03343942	0.2283098	-227.3196	-0.3084953	0.5368052	SLC39A5
cg04505023	0.391199	-242.3107	-0.3085439	0.6997429	SPRR1A
cg01774645	0.3735765	-237.9543	-0.3089375	0.682514	ARHGAP30
cg09300114	0.1577924	-242.8071	-0.3089397	0.466732	SLC16A5
cg03387723	0.4543735	-271.0217	-0.3090995	0.763473	SCMH1
cg01637734	0.3084218	-227.1574	-0.3091258	0.6175476	CD5L
cg21961766	0.3685766	-237.6838	-0.3094645	0.6780411	PRRG2
cg24852661	0.08128477	-278.1111	-0.3094985	0.3907833	GOLPH2
cg12061127	0.3592356	-235.6332	-0.3095291	0.6687647	WFDC9
cg23260026	0.1967403	-233.9593	-0.3095856	0.506326	FSTL3
cg01144251	0.4234826	-257.085	-0.309829	0.7333116	KLK9
cg05440289	0.4445443	-268.1076	-0.3101572	0.7547014	IVL
cg20189782	0.3994969	-248.4702	-0.3102429	0.7097399	MGC27121
cg18979223	0.3499402	-235.7845	-0.3106611	0.6606013	CDKN2B
cg11015241	0.5503455	-348.7749	-0.3106692	0.8610147	ATP10A
cg23713742	0.3318533	-232.6584	-0.3106843	0.6425375	SPAG4
cg11719283	0.3476937	-235.7845	-0.3109031	0.6585968	ZNF574
cg06236276	0.4471342	-271.3352	-0.3109686	0.7581028	SLC22A2
cg15842430	0.3167358	-231.4837	-0.3111222	0.627858	FAM12B
cg18138484	0.5136509	-323.2769	-0.3121738	0.8258247	CABP2
cg01074640	0.3346144	-236.1454	-0.3123727	0.6469871	IFNA17
cg03329572	0.3782331	-246.0053	-0.3123915	0.6906246	FCRL5
cg17398613	0.4418865	-272.6744	-0.3127682	0.7546546	SLC37A1

cg01718139	0.4526706	-279.7219	-0.3131233	0.7657939	UNQ3033
cg08093398	0.3388049	-238.2931	-0.3131634	0.6519684	PSF1
cg07792737	0.499006	-313.3028	-0.3133103	0.8123163	NPIP
cg00941549	0.3542565	-242.6567	-0.3138266	0.6680831	AKAP4
cg07441143	0.3756903	-248.3413	-0.3139452	0.6896355	SLURP1
cg21495715	0.4009342	-257.1218	-0.3141456	0.7150798	SLC5A10
cg19304352	0.4781792	-299.7588	-0.3143869	0.7925661	DEFA4
cg07339327	0.589286	-348.7749	-0.3146479	0.9039338	CCND1
cg24851490	0.1888121	-244.8876	-0.3154348	0.5042468	RNASE2
cg25778166	0.4093631	-263.4814	-0.3154951	0.7248582	FMO3
cg03752885	0.2096111	-241.1967	-0.315537	0.5251481	DAPK3
cg22549408	0.1818073	-246.801	-0.3156732	0.4974805	PMAIP1
cg02423618	0.2757107	-237.0193	-0.3158058	0.5915165	SPATA8
cg08970446	0.5067712	-327.5336	-0.3158175	0.8225887	SLC1A7
cg02324920	0.209111	-242.125	-0.3160629	0.5251739	NEURL
cg14256699	0.5442404	-348.7749	-0.3161483	0.8603887	SOST
cg10575735	0.284551	-237.9914	-0.3162329	0.6007839	SSX4
cg20416179	0.4743852	-301.8759	-0.3162399	0.7906252	C6orf71
cg18521925	0.5781611	-348.7749	-0.3162503	0.8944114	SLC22A16
cg22988566	0.4938543	-317.5233	-0.3163781	0.8102324	WFDC10B
cg01982597	0.3661152	-250.7563	-0.3164918	0.682607	PGBD3
cg08341924	0.4413522	-282.5049	-0.3169741	0.7583263	TGM1
cg18121684	0.3630699	-251.0052	-0.3170307	0.6801006	SERPINB13

cg01309153	0.5313424	-348.7749	-0.3171424	0.8484849	SURF1
cg05822532	0.2233445	-242.0048	-0.3171787	0.5405232	ELN
cg12593411	0.3117052	-241.9807	-0.3172739	0.6289791	ANGPTL6
cg03602500	0.4131526	-269.6009	-0.3175448	0.7306974	FLJ00060
cg02812142	0.3308622	-245.206	-0.3175869	0.6484491	ACMSD
cg19042947	0.3210836	-243.7479	-0.3176198	0.6387034	SERPINA4
cg12067287	0.4388438	-282.8668	-0.31771	0.7565538	MYOM1
cg25168545	0.1682519	-253.7777	-0.3178436	0.4860955	GIMAP1
cg05922591	0.4898552	-319.3784	-0.318222	0.8080772	LILRB4
cg21003606	0.5716891	-348.7749	-0.3183283	0.8900174	CALN1
cg20095587	0.3961494	-264.8537	-0.3185588	0.7147083	TREM2
cg25372195	0.3394875	-248.6429	-0.3185948	0.6580823	DCD
cg09701102	0.4342366	-283.1285	-0.3188852	0.7531219	NDUFV1
cg21450627	0.4967114	-327.4556	-0.3189473	0.8156587	PSD4
cg17264470	0.5351574	-348.7749	-0.3191949	0.8543522	FGF21
cg19233472	0.4023962	-270.363	-0.3199585	0.7223547	FOXI1
cg04816348	0.5461007	-348.7749	-0.3201669	0.8662676	CLEC4G
cg16772207	0.300029	-246.0688	-0.320203	0.620232	MYT1
cg25101056	0.3682984	-259.6443	-0.3205852	0.6888835	KCNG4
cg12582965	0.538265	-348.7749	-0.3206046	0.8588696	ATP10A
cg08244028	0.3322591	-251.5128	-0.3207971	0.6530562	MSH3
cg19954000	0.1807596	-256.083	-0.3212267	0.5019863	FGF1
cg13271951	0.2038947	-251.6207	-0.3213497	0.5252445	FAM57B

cg15780361	0.2299047	-248.4702	-0.321486	0.5513908	ALS2CR11
cg25400358	0.2410407	-248.0784	-0.3217643	0.5628049	GPR137
cg20932053	0.3926281	-270.7892	-0.3219326	0.7145607	CPM
cg04254916	0.539686	-348.7749	-0.3223692	0.8620552	KRT5
cg01883966	0.4561415	-305.0645	-0.3223773	0.7785187	STK19
cg08972170	0.2339301	-250.3322	-0.3227753	0.5567054	Ells1
cg11554507	0.3991411	-276.0389	-0.3231147	0.7222558	NEUROD6
cg02028524	0.5449733	-348.7749	-0.3231244	0.8680977	ATXN3
cg01182697	0.3052483	-252.9377	-0.3236393	0.6288877	TMEM59
cg13397379	0.3157371	-257.1284	-0.3251512	0.6408883	OR2C3
cg19982860	0.3042715	-255.7838	-0.3252042	0.6294757	IFNA21
cg19949550	0.4134928	-287.7309	-0.3253303	0.7388231	ASB2
cg13204181	0.3053282	-257.085	-0.3258382	0.6311664	GH1
cg11113534	0.5300537	-348.7749	-0.3259196	0.8559733	C20orf70
cg01894895	0.4521631	-312.1754	-0.3261403	0.7783033	ANXA1
cg16990174	0.2211758	-257.6286	-0.3263122	0.547488	RYBP
cg13656062	0.425539	-296.9852	-0.3265587	0.7520977	CYP4F2
cg07408456	0.2800498	-256.3827	-0.3266129	0.6066627	PGLYRP2
cg25033144	0.4058695	-287.4645	-0.3267667	0.7326362	FLJ00060
cg20543571	0.5135859	-348.7749	-0.3273217	0.8409076	C15orf43
cg17788013	0.3699794	-274.8345	-0.3275354	0.6975147	SPINK5
cg15905124	0.4435846	-310.2589	-0.3276067	0.7711913	MGC13034
cg20576002	0.2806484	-259.0337	-0.3280269	0.6086753	FAM112B

cg24670715	0.1713827	-270.2169	-0.3282604	0.4996431	ANGPT2
cg04574507	0.320428	-264.5321	-0.3284984	0.6489264	CD1B
cg02854090	0.4854319	-345.7707	-0.3285809	0.8140128	HIST1H2AA
cg18241647	0.4670315	-329.9	-0.3285989	0.7956304	WFDC12
cg02351381	0.1352054	-283.05	-0.3289891	0.4641945	C12orf34
cg19807685	0.3460565	-272.0647	-0.3295546	0.6756111	HSD17B2
cg12069042	0.3494155	-272.9709	-0.3295701	0.6789856	PLXNB1
cg03309967	0.2867194	-262.5089	-0.3296222	0.6163416	PSENEN
cg19368582	0.3172802	-266.4619	-0.3297521	0.6470323	MMRN2
cg25781162	0.4241525	-304.3334	-0.3298658	0.7540183	ABCG5
cg23514672	0.5426379	-348.7749	-0.3300395	0.8726774	FLJ32871
cg24949488	0.3425885	-273.2851	-0.3305674	0.6731559	DNTT
cg14696820	0.2702724	-263.5793	-0.3306769	0.6009493	LCE1A
cg22013966	0.5103047	-348.7749	-0.3306804	0.8409851	SERPINA13
cg16504670	0.3743675	-284.0467	-0.3309907	0.7053581	FLJ20186
cg27157038	0.4188251	-304.5585	-0.3311552	0.7499803	DNTT
cg19859270	0.3516883	-277.267	-0.3312573	0.6829456	GPR15
cg02187357	0.3034384	-267.8733	-0.331506	0.6349444	TBC1D22B
cg02442161	0.4215718	-307.0399	-0.3315553	0.7531271	PI3
cg07643942	0.2304846	-266.2505	-0.3316405	0.5621251	LACRT
cg18534730	0.3089684	-268.873	-0.3316447	0.6406131	CABP5
cg21065959	0.3270609	-272.1416	-0.3317196	0.6587805	LCE1E
cg13760253	0.3162237	-270.5275	-0.3318921	0.6481158	DNAJC5B

cg15928132	0.2756708	-266.1103	-0.3318962	0.607567	CCKAR
cg20891917	0.3902772	-293.15	-0.3322009	0.7224781	IFRD1
cg19561774	0.5268241	-348.7749	-0.3324218	0.8592458	SLC22A2
cg00075967	0.3824354	-291.3182	-0.3328268	0.7152622	STRA6
cg23617760	0.3733044	-288.7275	-0.3332415	0.7065459	C16orf44
cg10758292	0.4364644	-320.8498	-0.3333943	0.7698587	DEFA1
cg19717150	0.3335588	-277.8349	-0.3337365	0.6672953	HNF4A
cg10322876	0.2894093	-271.2307	-0.3340718	0.6234811	CYP2B6
cg16462075	0.3905206	-298.1159	-0.3343013	0.7248219	MUC3B
cg13928961	0.3672721	-289.3833	-0.3345127	0.7017848	K6IRS3
cg26946769	0.4461865	-331.5528	-0.3348459	0.7810324	MAPK4
cg07824742	0.4240645	-317.2795	-0.3350067	0.7590712	DBH
cg14894144	0.1440335	-290.8352	-0.3354205	0.479454	LAMA3
cg19587887	0.3591017	-289.2095	-0.3356785	0.6947802	PSKH2
cg07297178	0.4227123	-320.8498	-0.3367051	0.7594174	CEACAM7
cg10805676	0.3764992	-298.1159	-0.3368258	0.7133251	MRPL28
cg08475088	0.5112026	-348.7749	-0.3372961	0.8484987	NALP9
cg27377450	0.4690699	-348.7749	-0.337738	0.8068079	ARHGEF18
cg00689340	0.2298347	-278.4274	-0.3382654	0.5681001	RTKN
cg13311440	0.1763167	-286.8754	-0.3384621	0.5147789	CD48
cg16507522	0.4233927	-326.4729	-0.3386524	0.7620451	SERPINA3
cg14740251	0.3529815	-294.6292	-0.3389704	0.6919519	SIGLEC5
cg19047670	0.3294801	-288.479	-0.3391854	0.6686655	CCND1

cg26473272	0.5279998	-348.7749	-0.3393892	0.867389	SYT8
cg20544605	0.3180154	-286.9964	-0.3396858	0.6577012	SORBS2
cg11830061	0.5756878	-348.7749	-0.3400308	0.9157187	INSL6
cg06101324	0.1550567	-296.0378	-0.3403668	0.4954236	SPRR1A
cg11599505	0.4242913	-332.6265	-0.340737	0.7650282	C20orf102
cg16222568	0.3685597	-305.0645	-0.3411613	0.709721	APEG1
cg03221619	0.2118894	-286.0238	-0.3414024	0.5532917	FCER2
cg00209066	0.4603572	-348.7749	-0.3423614	0.8027186	BRD1
cg10990993	0.2558253	-286.3273	-0.3427078	0.5985331	MLH1
cg13053608	0.1682345	-296.6315	-0.3427556	0.5109901	LGP1
cg12815142	0.3333382	-297.2897	-0.3427876	0.6761258	SPAG7
cg08450982	0.2457947	-286.7545	-0.3429427	0.5887374	NUMBL
cg18881269	0.3009802	-291.7268	-0.3434087	0.6443889	LEPREL2
cg19728577	0.4293316	-344.0611	-0.3436246	0.7729563	GUCA2B
cg07651914	0.2293739	-289.0584	-0.343845	0.5732188	CLDN15
cg04727522	0.3212467	-297.3263	-0.3441927	0.6654394	C18orf22
cg11739626	0.3560668	-307.6586	-0.3442936	0.7003604	AKT1S1
cg14182690	0.4464585	-348.7749	-0.344438	0.7908964	RUNX3
cg08634024	0.47401	-348.7749	-0.3444574	0.8184674	OR2F1
cg06220755	0.2735677	-290.8352	-0.3445483	0.618116	RAI2
cg08448751	0.3164496	-297.9808	-0.3449927	0.6614423	SEMA3G
cg04711324	0.2199168	-292.3047	-0.3451746	0.5650914	RIT2
cg04810997	0.3262171	-300.7907	-0.3452186	0.6714357	TAS2R60

cg13320683	0.3166404	-299.2458	-0.3455568	0.6621972	RHOBTB1
cg25203980	0.4641289	-348.7749	-0.3457506	0.8098795	CENTB5
cg24750391	0.1432015	-309.479	-0.3458347	0.4890362	PON3
cg10853416	0.3606207	-313.3767	-0.3460103	0.7066309	MS4A7
cg19845843	0.3639342	-314.9962	-0.3461321	0.7100663	CXorf20
cg00463202	0.4237967	-347.2758	-0.3461489	0.7699456	ADPRHL1
cg17829936	0.3391233	-306.7665	-0.3463186	0.6854419	TAAR5
cg03468463	0.2994345	-297.6812	-0.3463667	0.6458012	SERPINB12
cg10569414	0.3428325	-308.6522	-0.3466473	0.6894798	C21orf121
cg15538820	0.5697677	-348.7749	-0.3467164	0.9164841	OBP2B
cg25072962	0.2782285	-296.554	-0.3471057	0.6253342	MGC35295
cg27418851	0.311491	-302.8915	-0.3477194	0.6592104	MBL2
cg01015871	0.2688373	-297.2913	-0.3479045	0.6167418	MT4
cg26701826	0.3084849	-302.9755	-0.3480487	0.6565337	MGC26963
cg00042156	0.2471239	-297.0272	-0.348168	0.5952918	MGC16291
cg23571857	0.198064	-301.507	-0.3486586	0.5467227	BIRC4BP
cg05547500	0.4804525	-348.7749	-0.3486833	0.8291358	TXNDC2
cg19728223	0.1896538	-302.9755	-0.3487248	0.5383787	KCNQ1
cg17778867	0.3272455	-309.6911	-0.3491242	0.6763697	KRTAP10-8
cg23815000	0.3778041	-329.1322	-0.3493912	0.7271953	LCN1
cg25004981	0.5378709	-348.7749	-0.3509209	0.8887918	LASS3
cg24901474	0.2006142	-305.5334	-0.3510062	0.5516204	RGS5
cg07997737	0.2210947	-303.8585	-0.3511978	0.5722926	NRTN

cg04891836	0.2934032	-307.0812	-0.3512458	0.644649	TNFSF14
cg14150666	0.4839177	-348.7749	-0.3515122	0.8354299	IL8RB
cg26292028	0.338005	-319.5547	-0.3519822	0.6899872	FLJ37587
cg00727947	0.5193245	-348.7749	-0.3537263	0.8730508	LILRA5
cg05215575	0.2110835	-310.0504	-0.3539603	0.5650438	FLJ25410
cg16545105	0.3026612	-315.692	-0.3544002	0.6570615	CRHBP
cg10370591	0.2640827	-312.5139	-0.355426	0.6195087	TPO
cg12022621	0.1952462	-315.0337	-0.355493	0.5507392	LAX1
cg26422060	0.4297962	-348.7749	-0.3562454	0.7860416	TBX10
cg14238120	0.3563842	-337.0802	-0.3563156	0.7126997	ELA3A
cg16794682	0.3965029	-348.7749	-0.3569428	0.7534457	CCND1
cg27341860	0.5343295	-348.7749	-0.3570616	0.891391	OR2L13
cg21686987	0.4186376	-348.7749	-0.3578405	0.7764781	CTRB1
cg03160508	0.1568123	-327.9585	-0.3579195	0.5147318	RHOD
cg15503752	0.3715441	-348.7749	-0.3581851	0.7297292	ST6GALNAC1
cg19292712	0.3712697	-348.7749	-0.3589695	0.7302393	SPAM1
cg04719766	0.4248973	-348.7749	-0.3590316	0.7839289	KCNQ1
cg20373326	0.3214322	-332.4868	-0.3595674	0.6809996	HSD17B2
cg21640749	0.2827542	-323.7346	-0.3595734	0.6423277	CD300LF
cg01103730	0.3375708	-338.4027	-0.3597994	0.6973703	IL20
cg12339029	0.3652011	-348.7749	-0.3612871	0.7264883	MYL1
cg15652212	0.3316394	-341.1264	-0.361736	0.6933754	FLJ90586
cg16175263	0.2003271	-328.7425	-0.3626026	0.5629296	TNFRSF10C

cg10604646	0.1745284	-332.9479	-0.3626198	0.5371482	RGS5
cg06244417	0.4120114	-348.7749	-0.3627746	0.774786	FCN1
cg26956535	0.3958749	-348.7749	-0.3627959	0.7586708	LAG3
cg10586756	0.3181477	-339.503	-0.3628575	0.6810052	NUP93
cg09037813	0.2953879	-335.3931	-0.3635995	0.6589874	LRRFIP1
cg11939496	0.2945712	-335.3694	-0.3636539	0.6582251	CD244
cg04450876	0.4208348	-348.7749	-0.3637578	0.7845926	FAM112B
cg11158374	0.3409301	-348.7749	-0.3641396	0.7050698	TFF2
cg27285599	0.3972953	-348.7749	-0.3642851	0.7615805	FLJ13841
cg06536578	0.2856412	-335.5433	-0.3645044	0.6501456	JPH4
cg26420196	0.107358	-348.7749	-0.3647684	0.4721264	GAS6
cg06351503	0.3214939	-346.1646	-0.3651345	0.6866283	RDBP
cg02067021	0.3214141	-348.7749	-0.3671657	0.6885798	DNAJC5B
cg23749046	0.4210391	-348.7749	-0.3672394	0.7882785	GPR61
cg24694549	0.2729596	-340.0665	-0.3673411	0.6403007	GRIP1
cg25934198	0.4200501	-348.7749	-0.3677717	0.7878218	FLJ36268
cg00334507	0.2554906	-341.3702	-0.3688123	0.6243029	MVP
cg01827098	0.4260628	-348.7749	-0.3690447	0.7951075	GIMAP7
cg09448880	0.2688533	-345.975	-0.3701201	0.6389734	PGLYRP3
cg05766474	0.4462655	-348.7749	-0.3722165	0.818482	CCL16
cg17907567	0.2495751	-348.7267	-0.3722184	0.6217936	HAMP
cg27442349	0.2608824	-348.7749	-0.3723222	0.6332046	NFKBIB
cg12456510	0.306675	-348.7749	-0.3728879	0.6795629	TFF2

cg20551517	0.3850877	-348.7749	-0.374342	0.7594296	GIP
cg26927807	0.3188835	-348.7749	-0.3747691	0.6936526	BTBD2
cg19917856	0.1371489	-348.7749	-0.3757069	0.5128558	LOC342897
cg00392257	0.225586	-348.7749	-0.3773492	0.6029352	ISG20L2
cg24407065	0.4256686	-348.7749	-0.3775972	0.8032658	BLZF1
cg09837648	0.4888737	-348.7749	-0.3777339	0.8666076	PLXNB1
cg22021786	0.3327555	-348.7749	-0.378675	0.7114305	WFDC8
cg22784047	0.3078903	-348.7749	-0.3787077	0.6865979	MVP
cg26149550	0.4098181	-348.7749	-0.3787282	0.7885463	KLK15
cg18920397	0.3233509	-348.7749	-0.3793584	0.7027093	LY9
cg12266049	0.4434841	-348.7749	-0.3803962	0.8238803	CCND1
cg08573687	0.2995992	-348.7749	-0.381118	0.6807172	TH
cg12513481	0.1789031	-348.7749	-0.3813594	0.5602626	SCAP1
cg05348870	0.3878016	-348.7749	-0.3815948	0.7693964	TNFSF14
cg06123346	0.2381001	-348.7749	-0.3818949	0.6199951	ATP4A
cg24024214	0.4389569	-348.7749	-0.3825926	0.8215494	BTNL8
cg13578652	0.2515547	-348.7749	-0.3835748	0.6351296	UBASH3A
cg21948655	0.4149814	-348.7749	-0.3838922	0.7988736	SMCP
cg20334738	0.2641549	-348.7749	-0.3847269	0.6488818	MAB21L2
cg14706739	0.3043368	-348.7749	-0.3882393	0.6925761	EPB49
cg14287742	0.3607836	-348.7749	-0.3890128	0.7497964	BLZF1
cg27619475	0.1665866	-348.7749	-0.3898995	0.5564861	SLC16A5
cg24363955	0.3955405	-348.7749	-0.3900249	0.7855654	FLJ14054

cg10062065	0.2680338	-348.7749	-0.3903173	0.6583511	APEG1
cg19712821	0.1760713	-348.7749	-0.3907272	0.5667985	KSP37
cg12489960	0.2773709	-348.7749	-0.3914766	0.6688474	SGCB
cg05444024	0.4591744	-348.7749	-0.3926745	0.8518489	FUT6
cg06539449	0.3446391	-348.7749	-0.3946602	0.7392992	CCND1
cg14333565	0.396083	-348.7749	-0.3953836	0.7914666	NRTN
cg02833725	0.2565947	-348.7749	-0.395802	0.6523967	ISG20L2
cg18508525	0.4157193	-348.7749	-0.3960332	0.8117525	CD36
cg01335367	0.1252518	-348.7749	-0.3980991	0.5233509	C12orf34
cg25221254	0.3252484	-348.7749	-0.4011958	0.7264441	ASAH3
cg09542291	0.3679734	-348.7749	-0.4014573	0.7694307	SMCP
cg14204735	0.06346902	-348.7749	-0.4026766	0.4661456	CYB561
cg26672426	0.2225113	-348.7749	-0.4037136	0.626225	PTGES
cg18149207	0.3283783	-348.7749	-0.4049728	0.7333511	RORC
cg22374142	0.09221421	-348.7749	-0.4054623	0.4976765	HSF4
cg08626653	0.3053736	-348.7749	-0.405626	0.7109996	FLJ37538
cg05671018	0.2701975	-348.7749	-0.4091846	0.679382	LYSMD2
cg21457804	0.3395817	-348.7749	-0.4109329	0.7505146	CT45-2
cg25813714	0.4318691	-348.7749	-0.4117246	0.8435937	CYP4F12
cg04144768	0.2673632	-348.7749	-0.4117397	0.6791029	DDC
cg25119415	0.2724371	-348.7749	-0.4155039	0.687941	MNDA
cg24992780	0.3828233	-348.7749	-0.415848	0.7986712	OR7C1
cg27235662	0.3405131	-348.7749	-0.4166173	0.7571304	CLDN16

cg20154346	0.2175191	-348.7749	-0.4204669	0.6379861	RAI2
cg14992108	0.2941869	-348.7749	-0.4260712	0.7202581	SNTB1
cg26164184	0.3855456	-348.7749	-0.4293696	0.8149152	FCN2
cg17357062	0.3870036	-348.7749	-0.4387513	0.8257549	FCN1
cg06639544	0.2950993	-348.7749	-0.4434159	0.7385153	OR7A5
cg15711744	0.3159782	-348.7749	-0.4465452	0.7625234	ANP32D
cg00698688	0.3535878	-348.7749	-0.4504796	0.8040674	SULT2B1
cg04138756	0.3275818	-348.7749	-0.4530488	0.7806306	SPRR3
cg16179125	0.1886545	-348.7749	-0.4545456	0.6432001	CTSZ
cg22575540	0.2930815	-348.7749	-0.4627779	0.7558594	TRIM54
cg25856811	0.250794	-348.7749	-0.4671628	0.7179568	SPRR3
cg13021192	0.1280138	-348.7749	-0.4725023	0.6005161	CTSZ
cg13726507	0.3544714	-348.7749	-0.4739468	0.8284182	CTAG2

Suppelementary table 12. Probes differentially methylated between tumours without known mutations and normal tissue.

TargetID	unknown.AVG_Beta	unknown.DiffScore	unknown.Delta Beta	normal.AVG_Beta	SYMBOL
cg01346152	0.6814715	350.469	0.4304945	0.2509769	DHRS3
cg13547644	0.5873128	350.469	0.4125772	0.1747356	ACTA1
cg16363586	0.7098469	350.469	0.3982374	0.3116095	BST2
cg24101578	0.7102449	350.469	0.3705311	0.3397138	CDH22
cg03852144	0.6351823	350.469	0.3689304	0.2662518	GLRX
cg03096975	0.5258527	350.469	0.3523498	0.1735029	EML2
cg17105014	0.5261912	350.469	0.3479071	0.1782841	GYPC
cg26509022	0.5475106	350.469	0.3441353	0.2033753	ALDH1A3
cg14409083	0.5781931	350.469	0.3396169	0.2385762	EMP1
cg15958424	0.6006592	350.469	0.3321485	0.2685107	ACPP

cg24122922	0.7519797	350.469	0.3292961	0.4226836	C20orf39
cg17518962	0.5537251	350.469	0.3288307	0.2248944	GAL3ST4
cg18702197	0.6944927	350.469	0.328288	0.3662047	HOXD3
cg22740835	0.6398525	350.469	0.325657	0.3141955	DDR2
cg13351161	0.5640377	350.469	0.3244539	0.2395838	SCARA3
cg10861599	0.600754	350.469	0.3187473	0.2820067	TNFSF4
cg04106785	0.7986509	350.469	0.3125568	0.4860941	CDK5R1
cg19224278	0.6235228	350.469	0.3086542	0.3148687	ALDH1A3
cg08624249	0.7847296	350.469	0.3056994	0.4790302	KIAA0889
cg11397854	0.4401974	350.469	0.3035792	0.1366182	IQSEC1
cg03642518	0.6914626	350.469	0.3025503	0.3889123	PNOC
cg06204948	0.5280117	350.469	0.3016967	0.226315	MARK2
cg17890764	0.7068838	350.469	0.3010291	0.4058547	ITIH4
cg18172186	0.8213209	350.469	0.3008283	0.5204926	KIAA1913
cg16517394	0.5006071	350.469	0.2958764	0.2047307	TNFSF4
cg09595479	0.4755045	350.469	0.2950402	0.1804643	PRPH
cg02490034	0.6915616	350.469	0.2943864	0.3971752	MEST
cg08831348	0.5661627	350.469	0.2891402	0.2770225	EML2
cg03605761	0.643705	350.469	0.2866039	0.357101	RNF126
cg25101936	0.7858939	350.469	0.2853156	0.5005782	ZBTB16
cg12564453	0.8170016	350.469	0.2833523	0.5336493	CETP
cg13906813	0.6822846	350.469	0.2830946	0.3991899	HLA-DPA1
cg01103836	0.9576275	350.469	0.2826693	0.6749582	MYO9B
cg04376312	0.5915592	350.469	0.2801917	0.3113675	MYLK
cg03562120	0.6342204	350.469	0.2798998	0.3543206	WISP2
cg18392482	0.8243915	350.469	0.2790235	0.545368	AMDHD1
cg02506908	0.6998755	350.469	0.2773866	0.4224889	HPD
cg07285276	0.7994277	350.469	0.2773489	0.5220789	RAPGEF1
cg01126560	0.6687445	350.469	0.2709431	0.3978014	C9orf142
cg24427660	0.7748529	350.469	0.2682846	0.5065683	PNPLA2
cg06507244	0.712719	350.469	0.2662384	0.4464805	DHX32

cg22601917	0.5191174	350.469	0.2647981	0.2543193	H6PD
cg23855989	0.4838436	350.469	0.2643647	0.2194789	AQP5
cg15926585	0.7788066	350.469	0.2639491	0.5148575	COMT
cg23283495	0.3758452	350.469	0.2636493	0.1121959	IRF6
cg18342279	0.3697937	350.469	0.2633064	0.1064874	ZAR1
cg13763232	0.7459232	350.469	0.2629043	0.4830189	SLC6A6
cg11554605	0.7776657	350.469	0.2598264	0.5178393	ASB4
cg24012708	0.6787763	350.469	0.2565539	0.4222224	HDHD3
cg20427879	0.4578368	350.469	0.2564145	0.2014222	EML2
cg04369341	0.4384564	350.469	0.2560803	0.182376	C20orf100
cg05342835	0.6274884	350.469	0.2550435	0.3724449	SYNC1
cg24459563	0.739025	350.469	0.2549827	0.4840423	CACNG1
cg14371590	0.5649461	350.469	0.2531691	0.3117769	SLC26A10
cg17791651	0.549388	350.469	0.2515884	0.2977996	POU3F1
cg08858521	0.4704008	350.469	0.2512837	0.219117	WFIKKN1
cg24739326	0.4359259	350.469	0.2510368	0.1848891	CHST8
cg00240880	0.8355954	350.469	0.2493613	0.5862341	WISP2
cg07360692	0.4713236	350.469	0.2475134	0.2238103	FLJ20032
cg26668713	0.3950138	350.469	0.2474786	0.1475352	SIPA1
cg07236190	0.7302842	350.469	0.2446781	0.4856061	AMDHD1
cg10705800	0.610572	350.469	0.2433327	0.3672392	CITED4
cg17686260	0.7416171	350.469	0.2426279	0.4989893	MGMT
cg12232463	0.6724831	350.469	0.2419253	0.4305579	LONRF2
cg17998964	0.4034719	350.469	0.2396806	0.1637913	MARK2
cg25277950	0.3862685	350.469	0.2393395	0.146929	EML2
cg27301343	0.3826765	350.469	0.2374098	0.1452667	EML2
cg24315815	0.7594456	350.469	0.2352092	0.5242364	PLSCR4
cg16077929	0.7387622	350.469	0.2348567	0.5039055	CDKL1
cg12177001	0.7549378	350.469	0.2341995	0.5207384	IFI27
cg02674804	0.8607553	350.469	0.2312064	0.6295489	REEP6
cg27114026	0.7605855	350.469	0.2297867	0.5307989	ELA1

cg05485060	0.6416215	142.6492	0.2297268	0.4118947	CTNNAL1
cg11318251	0.5723045	350.469	0.2287531	0.3435514	BAALC
cg02867079	0.4369153	350.469	0.2283422	0.2085731	KIAA1822
cg00431549	0.5604503	350.469	0.226958	0.3334922	MGP
cg02085507	0.4873027	350.469	0.2267435	0.2605592	TRIP10
cg27491887	0.4306612	350.469	0.2259769	0.2046843	KCNQ1
cg20748065	0.8443555	350.469	0.2254241	0.6189314	POR
cg22628873	0.6773793	136.8612	0.2245508	0.4528286	GGT6
cg16616769	0.7645056	142.6492	0.2245093	0.5399963	MGC35048
cg04640913	0.6180028	135.9285	0.2232044	0.3947985	CDH22
cg20777437	0.5372733	142.6492	0.2219902	0.3152832	CDCP2
cg01724150	0.5003433	350.469	0.2219546	0.2783887	NMNAT3
cg25683185	0.6858693	133.0886	0.2215774	0.4642918	ACRBP
cg04099420	0.5605203	135.9285	0.2186689	0.3418514	RIPK1
cg23418591	0.3284849	350.469	0.2153936	0.1130913	FLJ90166
cg05253327	0.7646453	132.6921	0.2136019	0.5510435	B3GNT1
cg22759185	0.7725071	133.5218	0.2132554	0.5592517	REEP6
cg01333011	0.5161049	135.1906	0.2128832	0.3032217	PTHLH
cg18109798	0.5877451	123.9389	0.2123849	0.3753601	SLCO1C1
cg17339202	0.490423	139.7417	0.2123547	0.2780684	SYNC1
cg17496788	0.5951198	122.5345	0.2117739	0.3833458	DDR2
cg03271907	0.7998694	138.0554	0.2112125	0.5886568	MGMT
cg04902405	0.6912694	119.1106	0.2093396	0.4819297	ZC3H11A
cg04498511	0.8106005	138.0554	0.209052	0.6015485	ZC3H11A
cg06834875	0.5246346	127.4415	0.2086236	0.316011	KIAA1822
cg16612562	0.6443003	116.7884	0.2085239	0.4357764	RRP22
cg13493001	0.7839817	127.7793	0.205815	0.5781667	LCN12
cg03630088	0.4568078	139.7417	0.2057614	0.2510464	CPXM2
cg02500392	0.8224658	136.8612	0.2048469	0.6176189	PBOV1
cg25936385	0.7644063	122.662	0.2046797	0.5597265	FAIM2
cg209598	0.3209203	350.469	0.2042592	0.1166611	AJAP1

66					
cg09547190	0.5508294	117.2171	0.2041111	0.3467183	C9orf89
cg04700814	0.4973715	125.1854	0.2032702	0.2941013	HEXIM1
cg21685427	0.7495278	117.868	0.202522	0.5470058	SGK2
cg20833786	0.6462013	109.6015	0.2022284	0.4439728	MRGPRX3
cg02000005	0.3410668	350.469	0.202108	0.1389588	CRIP1
cg02806777	0.5145999	119.0985	0.2013923	0.3132075	PGLYRP1
cg14444710	0.7877882	122.6297	0.2002894	0.5874987	PDPK1
cg09931793	0.4911548	-128.5634	-0.2000441	0.6911989	OR2K2
cg25781162	0.5537649	-152.4789	-0.2002534	0.7540183	ABCG5
cg05248781	0.6409426	-219.035	-0.2003386	0.8412812	LCE5A
cg26511321	0.3597817	-108.6272	-0.2003743	0.560156	HOXA7
cg22960952	0.2887161	-108.0575	-0.2004288	0.4891449	FLJ23657
cg05301852	0.3942087	-111.4083	-0.2004712	0.59468	FABP1
cg10190509	0.5737554	-163.862	-0.2005683	0.7743237	CCL16
cg26143719	0.1893438	-118.6379	-0.2006184	0.3899621	C1QTNF6
cg23240895	0.5921366	-175.9917	-0.2006547	0.7927914	PRR5
cg19546781	0.6315832	-209.94	-0.2006587	0.8322418	CCL17
cg16155702	0.5687166	-161.1652	-0.2006587	0.7693753	FGF21
cg10848367	0.4534241	-120.5707	-0.200776	0.6542001	SCGB1D2
cg07979357	0.09189799	-147.5306	-0.2007895	0.2926875	IL27RA
cg14345281	0.1704164	-122.6268	-0.2008008	0.3712171	NHS
cg26246138	0.1424249	-129.7667	-0.2009023	0.3433271	SCML2
cg26628847	0.3036134	-108.3471	-0.2010518	0.5046653	PIP
cg19798224	0.5663769	-160.8976	-0.2011512	0.7675281	C14orf68
cg16998872	0.5543851	-154.5475	-0.2011616	0.7555467	GYPE
cg04655481	0.5008295	-133.2088	-0.2011638	0.7019932	GPR21
cg24352688	0.4165213	-115.0526	-0.2012428	0.6177641	OFD1
cg24698533	0.4817888	-127.8783	-0.2012511	0.6830398	LYZL2
cg10968815	0.5051225	-134.879	-0.2013581	0.7064807	BPIL1
cg23661676	0.3408925	-109.0015	-0.2013856	0.5422781	GPR42

cg27291231	0.4933282	-131.4405	-0.2014705	0.6947987	SSNA1
cg12855851	0.606599	-189.2622	-0.2015212	0.8081203	PGC
cg15005385	0.3676634	-110.5808	-0.2016266	0.56929	CCL3L1
cg15096123	0.6527966	-237.8688	-0.2016768	0.8544734	KLK4
cg14287742	0.5481113	-152.5283	-0.2016851	0.7497964	BLZF1
cg03977657	0.5871169	-174.9308	-0.2016891	0.788806	LAMB3
cg26745032	0.252583	-111.5679	-0.2016976	0.4542806	REPS2
cg10878307	0.5519138	-154.5287	-0.2017702	0.753684	ATAD4
cg23812886	0.3305065	-109.1984	-0.201776	0.5322825	SSX5
cg07879977	0.3231188	-109.2624	-0.2019235	0.5250422	OR1F1
cg03264414	0.6387786	-221.6779	-0.2019572	0.8407358	PAEP
cg05446471	0.6374071	-220.1971	-0.201979	0.8393861	HDAC11
cg09033997	0.5264444	-143.6712	-0.202019	0.7284634	CCR9
cg03309967	0.4142775	-115.8041	-0.2020641	0.6163416	PSENEN
cg14519000	0.674649	-272.4478	-0.2020976	0.8767466	GATA5
cg08886154	0.3435394	-109.9685	-0.2021428	0.5456822	PAX4
cg01375871	0.292162	-109.9208	-0.202222	0.4943841	TPSD1
cg26457013	0.4282984	-118.0962	-0.2022548	0.6305531	TMEM86B
cg24459209	0.4341656	-119.0916	-0.2022845	0.6364501	PRG3
cg09528351	0.4890288	-131.7593	-0.2024699	0.6914986	PIK3R5
cg05997860	0.4198144	-117.1577	-0.2025199	0.6223344	ADAM21
cg15585987	0.2586266	-111.9817	-0.2025244	0.461151	SNTG1
cg08579995	0.566052	-163.5891	-0.2025282	0.7685802	FLJ46230
cg14532417	0.5560964	-158.2033	-0.2025285	0.758625	TBC1D3
cg27235662	0.5545719	-157.4603	-0.2025585	0.7571304	CLDN16
cg21624359	0.2754244	-111.0086	-0.2025919	0.4780163	FFAR3
cg22879289	0.3321253	-110.2431	-0.2026705	0.5347958	NID1
cg08742106	0.6817402	-287.805	-0.2027206	0.8844607	USP6
cg14662756	0.4419882	-121.1227	-0.2027497	0.6447378	NPFF
cg07728874	0.6182184	-203.3476	-0.2029507	0.8211691	CD3D
cg19589427	0.4562003	-124.3003	-0.2029857	0.659186	TNFSF18

cg05546044	0.3555813	-111.7557	-0.2032208	0.5588021	MAPK1
cg27562023	0.4343779	-120.4867	-0.2032826	0.6376605	RPH3AL
cg08130265	0.3575872	-112.0204	-0.2033535	0.5609407	C15orf5
cg19304352	0.5891515	-180.5075	-0.2034146	0.7925661	DEFA4
cg12351042	0.4446113	-122.5696	-0.2034309	0.6480422	OR2B2
cg16008138	0.2571468	-113.1126	-0.2034487	0.4605955	RNF190
cg23680518	0.6143672	-201.2478	-0.2034728	0.81784	SBSN
cg11825652	0.3779734	-113.7295	-0.2035752	0.5815486	CAV2
cg25545210	0.4834449	-131.9233	-0.2035863	0.6870312	KRTHA4
cg19206010	0.2167109	-117.6592	-0.2037381	0.420449	UXT
cg07974891	0.3247699	-111.4157	-0.2037973	0.5285671	ITGB1BP1
cg18374517	0.4943168	-135.6153	-0.2039071	0.6982238	ALPPL2
cg22377428	0.5773259	-173.7116	-0.203989	0.781315	GPR142
cg26331247	0.2360469	-115.6356	-0.2039969	0.4400437	FLJ33706
cg07026910	0.5204266	-144.8506	-0.2040021	0.7244287	INPP5D
cg26185508	0.4759432	-130.7388	-0.2041225	0.6800656	CDCP2
cg24613957	0.4229544	-119.7586	-0.2041295	0.6270838	PLA2G2E
cg21003606	0.6858142	-302.5053	-0.2042032	0.8900174	CALN1
cg05800321	0.6269315	-215.6367	-0.2042062	0.8311377	LY6D
cg04995095	0.3203951	-111.8823	-0.2042515	0.5246466	CD300E
cg20176989	0.3948682	-116.2972	-0.2043197	0.5991879	KIR3DL2
cg02124291	0.5049173	-139.7856	-0.2043475	0.7092648	OR7A5
cg06494770	0.3388134	-112.3758	-0.2043531	0.5431665	KLHL13
cg18466173	0.5667016	-168.2278	-0.2045015	0.771203	C21orf93
cg11113534	0.6514103	-246.1186	-0.204563	0.8559733	C20orf70
cg16381688	0.3445688	-112.8477	-0.2045686	0.5491374	THEM2
cg24309555	0.4217562	-120.1644	-0.2045696	0.6263258	APOB
cg12891678	0.204842	-120.3019	-0.2045918	0.4094338	SPRR2D
cg26523005	0.3435605	-112.8375	-0.204592	0.5481524	ZNF662
cg23207527	0.523984	-147.4141	-0.2046331	0.7286171	RBM24
cg07446572	0.5849887	-180.7757	-0.2047662	0.7897549	CDCA3

cg06882926	0.4085213	-118.6298	-0.204848	0.6133693	OR1G1
cg13696012	0.6711499	-278.7536	-0.2050812	0.8762311	BPIL1
cg13181284	0.6366572	-229.4772	-0.2051809	0.8418382	KRT6B
cg21312148	0.3252937	-113.0564	-0.2052091	0.5305029	LCE2D
cg20751395	0.6446698	-239.8413	-0.2053316	0.8500014	KCNQ1
cg16742703	0.425084	-121.7885	-0.2053753	0.6304594	KLK3
cg01899253	0.2462854	-116.1413	-0.2053864	0.4516717	FLT1
cg06325687	0.6970082	-333.3647	-0.2054625	0.9024706	OPN1MW
cg17910564	0.3739811	-115.7492	-0.2054789	0.57946	VDAC3
cg25677709	0.5359511	-154.1642	-0.205486	0.7414371	NDST1
cg11939496	0.4527369	-127.2231	-0.2054881	0.6582251	CD244
cg15590780	0.2918416	-113.5887	-0.2054986	0.4973402	USH2A
cg24908058	0.6352202	-229.134	-0.2055966	0.8408167	CGB5
cg03366382	0.5256021	-150.0212	-0.2056855	0.7312876	INS
cg11037787	0.6128796	-206.0804	-0.2057103	0.8185899	PLA2G2A
cg18133957	0.1486544	-133.3648	-0.2057327	0.3543871	APC2
cg00727947	0.6672321	-275.0727	-0.2058187	0.8730508	LILRA5
cg04705866	0.4880067	-136.7902	-0.2058232	0.6938299	GZMK
cg24433189	0.1695476	-128.4517	-0.2059251	0.3754727	SSTR5
cg10249734	0.3917103	-118.0702	-0.2059871	0.5976974	SECTM1
cg11154879	0.5891174	-187.0628	-0.2060717	0.7951891	C20orf151
cg27413508	0.2414072	-117.4223	-0.2061578	0.447565	COX4I2
cg14672680	0.6781704	-296.7086	-0.2062725	0.8844429	TNNI2
cg01367992	0.3833066	-117.6153	-0.2063192	0.5896258	LY9
cg20401945	0.1712275	-128.5593	-0.2063641	0.3775916	ASPHD1
cg24353217	0.5585726	-167.6138	-0.206405	0.7649776	MYL2
cg23110514	0.2498434	-117.0057	-0.2064611	0.4563045	LCE3E
cg22988566	0.6037399	-200.0179	-0.2064925	0.8102324	WFDC10B
cg20090497	0.355367	-115.8041	-0.2065937	0.5619607	TAS2R9
cg16607065	0.5632492	-170.9287	-0.2066696	0.7699188	TP73
cg19553721	0.5657281	-172.4762	-0.2066909	0.772419	FAM106A

cg07371530	0.6104724	-206.6959	-0.2067301	0.8172026	RPUSD1
cg08109646	0.419904	-122.8122	-0.2067445	0.6266485	ZNF683
cg18809535	0.6381605	-236.4525	-0.206769	0.8449295	LDHAL6B
cg18192417	0.1752136	-128.2289	-0.2068391	0.3820527	NEBL
cg03782727	0.6482784	-250.3012	-0.2069372	0.8552155	FFAR1
cg14722162	0.4448616	-127.6751	-0.2069427	0.6518043	C5orf20
cg00308665	0.2560108	-117.055	-0.206954	0.4629648	HTR2A
cg02671171	0.3370948	-115.4611	-0.2069998	0.5440947	RPH3AL
cg18881269	0.4373844	-126.2811	-0.2070045	0.6443889	LEPREL2
cg16514843	0.3912475	-119.3631	-0.2070512	0.5982988	PAX4
cg10414946	0.423804	-124.019	-0.2071553	0.6309593	MS4A2
cg03931808	0.6797356	-304.5097	-0.2072996	0.8870353	RLN3
cg24489015	0.3874988	-119.2819	-0.2073068	0.5948056	LPO
cg20649991	0.5280616	-154.1094	-0.207316	0.7353776	LILRB5
cg15113803	0.6696181	-285.5268	-0.2073402	0.8769584	RHO
cg20104776	0.3590471	-117.0897	-0.2074685	0.5665156	LDOC1
cg27160701	0.3735544	-118.2489	-0.2075376	0.5810921	SBEM
cg05559445	0.2714891	-116.754	-0.2075766	0.4790657	CDKN1C
cg05786601	0.3066075	-115.7234	-0.2075888	0.5141963	AR
cg05873268	0.5221703	-152.1778	-0.2076178	0.7297881	TPSAB1
cg01479232	0.3874846	-119.9164	-0.2077961	0.5952808	C20orf54
cg09001777	0.4382776	-127.6751	-0.2078625	0.6461401	FUT3
cg12435792	0.2970656	-116.2344	-0.2079349	0.5050005	PDE6B
cg06836849	0.1775533	-129.0856	-0.2080698	0.3856231	SLC17A8
cg11830061	0.707561	-350.469	-0.2081577	0.9157187	INSL6
cg05055150	0.5275187	-155.592	-0.2082248	0.7357436	MAG
cg17040807	0.1686754	-131.2018	-0.2082254	0.3769008	CYGB
cg20692181	0.4071675	-122.9144	-0.2082276	0.6153951	RETN
cg09995854	0.4630795	-133.8065	-0.208274	0.6713535	IL1F8
cg18221897	0.4858996	-140.3149	-0.2083762	0.6942758	KIR2DL1
cg18429742	0.3804748	-119.9631	-0.208396	0.5888708	ZDHHC11

cg21631409	0.2877513	-117.1091	-0.2085067	0.496258	ALDH3B2
cg21643361	0.35389	-118.0709	-0.2085446	0.5624346	ZNF135
cg04810997	0.4627643	-134.3313	-0.2086715	0.6714357	TAS2R60
cg09256683	0.3592908	-118.577	-0.2086778	0.5679686	CCL14
cg24030627	0.5762408	-184.0521	-0.2086979	0.7849387	FCGBP
cg24262376	0.331793	-117.2821	-0.208701	0.540494	SCNM1
cg27065979	0.5974585	-201.009	-0.2088808	0.8063393	NEK3
cg09626634	0.3303216	-117.4628	-0.2088904	0.539212	EBI2
cg06001166	0.6141227	-216.6177	-0.2089593	0.8230821	RPL3L
cg24552358	0.4815399	-140.1182	-0.2090669	0.6906068	ORM1
cg10710439	0.2354975	-121.3784	-0.2091994	0.4446968	FLJ37549
cg04511534	0.5438331	-165.4612	-0.2092237	0.7530569	GGT6
cg08495878	0.6550438	-269.9883	-0.2095079	0.8645517	SERPINA4
cg03986640	0.5459397	-167.1935	-0.2095205	0.7554603	MIP
cg06415153	0.3424119	-118.6504	-0.2095262	0.5519381	PITPNM2
cg20713492	0.1210853	-146.0864	-0.209531	0.3306163	AQP10
cg21652958	0.1007467	-153.807	-0.2095331	0.3102798	THBS2
cg05556717	0.3893179	-122.5068	-0.2096322	0.5989501	CCL26
cg13269964	0.5552626	-172.9604	-0.2097577	0.7650203	CD300LG
cg26391080	0.514299	-152.9197	-0.2098035	0.7241026	SH2D4B
cg07895149	0.05086954	-180.0742	-0.2098125	0.260682	FAM26B
cg03803009	0.3804965	-121.8517	-0.2098636	0.5903602	GPR142
cg24214470	0.4978617	-147.0399	-0.2100016	0.7078633	SERPINF1
cg14550066	0.3239555	-118.6595	-0.2100357	0.5339913	NCR1
cg05812599	0.4924637	-145.2927	-0.2100838	0.7025475	CLPB
cg21958034	0.5646359	-179.5538	-0.2100997	0.7747356	MST1
cg15670863	0.4553983	-134.707	-0.2101005	0.6654989	SPACA4
cg25203980	0.5997654	-206.3144	-0.2101141	0.8098795	CENTB5
cg20423977	0.5120962	-152.6589	-0.2101421	0.7222383	PLAC4
cg06259570	0.4133554	-126.7126	-0.2103075	0.6236629	MMP27
cg18356799	0.3144839	-118.9114	-0.2103544	0.5248383	DSC1

cg22165685	0.08140638	-163.9483	-0.2107675	0.2921739	VENTX
cg21529807	0.1185305	-148.6042	-0.210917	0.3294475	CEACAM4
cg02633817	0.4743912	-141.2127	-0.2110352	0.6854264	FXYD3
cg04872051	0.3346286	-120.2465	-0.2110969	0.5457255	CHST7
cg05379350	0.1909437	-129.8725	-0.2110995	0.4020432	GIT1
cg24693053	0.2592574	-121.4959	-0.2111455	0.4704029	MFSD7
cg14141399	0.29558	-119.9631	-0.2111692	0.5067493	HAS1
cg22022041	0.5380645	-166.5446	-0.2112071	0.7492716	CCR9
cg13882988	0.5547553	-175.8891	-0.2112178	0.7659731	MBL2
cg05113558	0.5737854	-188.48	-0.211227	0.7850124	FOXH1
cg26531804	0.2658991	-121.3215	-0.2113598	0.4772589	SPINT1
cg04557383	0.1136411	-151.1879	-0.2116098	0.3252509	MT1H
cg09237521	0.6563461	-280.5189	-0.2116351	0.8679812	IFITM2
cg16098726	0.6453733	-263.5039	-0.2116461	0.8570194	GP9
cg21570818	0.4982508	-150.2259	-0.2117392	0.70999	FUT5
cg06094150	0.5776005	-192.5678	-0.2117527	0.7893532	MT1B
cg22088368	0.3218666	-120.7832	-0.2118356	0.5337022	MGC35206
cg17894008	0.6071035	-218.2567	-0.2119175	0.819021	NACAL
cg17733331	0.08168285	-165.2658	-0.2119741	0.293657	CDH3
cg17446142	0.5433201	-171.0543	-0.2120245	0.7553446	GDF9
cg09971646	0.1970133	-129.9568	-0.2121221	0.4091354	DLK1
cg05341878	0.3282439	-121.4764	-0.2122868	0.5405307	RIMS2
cg27360282	0.6913626	-350.469	-0.2123	0.9036626	RUNX3
cg12610070	0.08018404	-166.4167	-0.2123196	0.2925036	TSPAN15
cg01474260	0.3865168	-125.9763	-0.2124799	0.5989967	CESK1
cg09863066	0.2173319	-127.375	-0.2125627	0.4298946	PVALB
cg11505080	0.1932025	-131.2818	-0.2127006	0.405903	GPR173
cg21365235	0.2296418	-126.0399	-0.2127274	0.4423692	OCRL
cg24484296	0.4046156	-128.8465	-0.212829	0.6174446	ZFPL
cg21745164	0.3223725	-122.0125	-0.212848	0.5352205	LOC63928
cg000575	0.3608706	-123.9805	-0.2128941	0.5737647	GML

93					
cg07864297	0.201047	-130.1692	-0.2129075	0.4139546	ESRRB
cg04740359	0.2762787	-122.769	-0.2130809	0.4893597	NTF3
cg26189983	0.1376283	-144.8355	-0.213117	0.3507454	TNFRSF1B
cg15055101	0.1544856	-140.1182	-0.2131253	0.3676109	SH2D3A
cg14015044	0.07658646	-169.2131	-0.2131485	0.289735	TNFRSF10C
cg22975712	0.4578727	-140.1198	-0.2132112	0.6710839	GALR3
cg18783781	0.5575531	-182.1955	-0.2132328	0.7707858	MGC4399
cg04893119	0.2228675	-127.4225	-0.2132375	0.436105	PI15
cg00795812	0.1787919	-134.8225	-0.213403	0.392195	PDCD1
cg22789545	0.5447636	-174.8768	-0.2134242	0.7581878	C20orf114
cg25182621	0.379626	-126.4817	-0.2134455	0.5930715	CHRD
cg16176600	0.5210272	-162.8111	-0.2134973	0.7345245	FRK
cg12639234	0.3444065	-123.6608	-0.213506	0.5579125	NAT2
cg15905124	0.5576105	-183.0171	-0.2135808	0.7711913	MGC13034
cg18849169	0.4847164	-148.7223	-0.2135909	0.6983072	GPX3
cg17542495	0.3655609	-125.299	-0.2136022	0.5791631	GJB1
cg03599338	0.3813483	-126.9122	-0.213632	0.5949804	SUSD2
cg25514503	0.6761049	-326.8951	-0.2136523	0.8897572	PER3
cg12089698	0.3073692	-122.9144	-0.2137485	0.5211176	SPATC1
cg21130124	0.6139977	-230.8676	-0.2137656	0.8277633	CALML5
cg17788013	0.483533	-148.9983	-0.2139817	0.6975147	SPINK5
cg23988567	0.5519831	-180.4614	-0.214003	0.7659861	
cg19465374	0.5608659	-186.2172	-0.2140186	0.7748845	AZGP1
cg10417559	0.5646461	-188.8263	-0.2140325	0.7786786	LMO6
cg16175792	0.3318051	-123.7801	-0.2140659	0.5458711	HSD3B1
cg23749046	0.5741333	-195.9214	-0.2141452	0.7882785	GPR61
cg03352153	0.4810252	-148.6304	-0.2142509	0.6952761	GLULD1
cg12029639	0.2259652	-128.2478	-0.2142918	0.440257	MAB21L1
cg25993152	0.4889802	-151.9273	-0.214574	0.7035542	XAGE5
cg099238	0.3238379	-124.2567	-0.2146568	0.5384948	HTATSF1

55					
cg10837843	0.1365406	-147.1101	-0.2147884	0.351329	DUSP1
cg01530101	0.4131739	-132.9903	-0.2148096	0.6279835	KCNQ1DN
cg10368842	0.4012713	-131.2053	-0.2148829	0.6161542	C10orf81
cg21132577	0.1932048	-134.0552	-0.2151511	0.4083559	MYOM2
cg12040555	0.6773289	-337.0627	-0.215176	0.8925049	MGMT
cg10612997	0.4580204	-143.4454	-0.2152686	0.673289	GREB1
cg13125510	0.2548641	-126.9122	-0.2155385	0.4704025	C11orf44
cg15484375	0.4945348	-155.6151	-0.2155408	0.7100756	SAA1
cg21458041	0.4101068	-133.5356	-0.2155598	0.6256666	TNP2
cg11599505	0.5494546	-182.4408	-0.2155737	0.7650282	C20orf102
cg14528319	0.2101205	-131.85	-0.2156103	0.4257309	GIPC1
cg18414950	0.2382861	-128.7965	-0.2159404	0.4542266	PDK3
cg12949760	0.4785755	-150.7576	-0.2159554	0.694531	KCNQ1
cg25101056	0.4729202	-148.9466	-0.2159633	0.6888835	KCNG4
cg24655310	0.3936172	-131.761	-0.2160881	0.6097053	CYP4F11
cg02280309	0.4422333	-140.7464	-0.2160916	0.658325	PKLR
cg19067730	0.07989821	-171.1551	-0.2161478	0.296046	PPGB
cg09492887	0.1320534	-150.1627	-0.2162052	0.3482586	SLC26A5
cg20342105	0.282965	-126.3425	-0.2162868	0.4992518	BSCL2
cg11328541	0.1979558	-134.707	-0.2164336	0.4143894	OR7C1
cg11061975	0.330334	-126.7172	-0.2164456	0.5467796	SIRPB2
cg13397379	0.4244277	-137.435	-0.2164606	0.6408883	OR2C3
cg11237817	0.3882686	-131.6173	-0.2164993	0.6047679	KIR3DL3
cg14511156	0.2760058	-126.9951	-0.216635	0.4926409	OSCAR
cg23413307	0.4274972	-138.3327	-0.2166485	0.6441457	LCE1F
cg09686308	0.2700568	-127.3234	-0.2166822	0.486739	CIB3
cg11052143	0.1906442	-136.2896	-0.2167233	0.4073675	ALS2CR11
cg06825166	0.3177403	-126.9705	-0.2169519	0.5346922	TMEM10
cg22456522	0.220946	-132.0624	-0.2170863	0.4380323	LILRB3
cg19642007	0.4540908	-145.3859	-0.2171468	0.6712376	TNNT3

cg19987219	0.4439421	-142.8197	-0.2171555	0.6610975	FLJ32011
cg27329371	0.3348638	-127.8965	-0.2172309	0.5520948	ALDH3A1
cg15648315	0.5004948	-161.3441	-0.2173742	0.717869	FLJ26443
cg26757722	0.2485564	-129.5863	-0.217415	0.4659714	CACNG2
cg27117399	0.3773159	-131.5943	-0.217464	0.5947799	CNDP1
cg09037813	0.4414841	-142.7724	-0.2175033	0.6589874	LRRFIP1
cg04037732	0.2034376	-135.0772	-0.217542	0.4209796	NLGN3
cg04488758	0.4438677	-143.6366	-0.2177001	0.6615677	USP44
cg27257987	0.4292253	-140.4255	-0.2177957	0.6470209	PSG4
cg13453139	0.5337037	-179.0318	-0.2182046	0.7519083	PIK3R5
cg24840099	0.3427053	-129.5722	-0.2182157	0.5609211	MSX1
cg15140807	0.5233172	-173.6963	-0.2183293	0.7416465	FLJ31222
cg02202484	0.396992	-135.3632	-0.2183327	0.6153247	SPRR4
cg06385087	0.07043407	-178.8738	-0.2183457	0.2887798	CTSZ
cg01193293	0.3001904	-128.5817	-0.218394	0.5185844	SIGLEC7
cg07022477	0.3216245	-128.9072	-0.2184361	0.5400606	HIF3A
cg19154438	0.5554065	-193.2907	-0.2185379	0.7739443	CKM
cg19841506	0.1287738	-154.3558	-0.218887	0.3476608	ZMYND15
cg09229960	0.3646733	-132.3376	-0.2190202	0.5836935	EMD
cg24898863	0.168626	-143.5893	-0.2191253	0.3877513	S100A8
cg19464944	0.3262077	-129.9685	-0.2191606	0.5453683	FCGR1A
cg06818777	0.137514	-151.9596	-0.2191748	0.3566888	CHAD
cg15746187	0.4319582	-143.2854	-0.2192576	0.6512157	FBXO44
cg08952029	0.2519566	-131.7268	-0.2194564	0.471413	CHRDL2
cg17699374	0.2414953	-132.6909	-0.2195336	0.4610289	MGC35206
cg17504145	0.3955919	-136.9029	-0.2195781	0.61517	CRYBB2
cg00463848	0.5026488	-167.0475	-0.2198917	0.7225405	KRT2A
cg18669381	0.2455633	-132.761	-0.2199	0.4654633	ARHGEF19
cg04431776	0.5819734	-218.113	-0.2201635	0.802137	GAGE2
cg04744379	0.5235782	-177.7557	-0.2201833	0.7437615	KLK15
cg229704	0.1386455	-152.7987	-0.220206	0.3588514	SPATS1

35					
cg27496506	0.5428812	-189.1424	-0.2202755	0.7631567	TGM5
cg09799714	0.4870093	-161.3977	-0.2202791	0.7072884	PDZD3
cg23887102	0.3151982	-131.1117	-0.2203088	0.5355069	TAAR6
cg21399079	0.6531585	-311.7395	-0.2203584	0.8735169	GPR45
cg16084788	0.6236766	-264.8333	-0.2203919	0.8440685	SLC2A7
cg04273431	0.1686375	-145.1097	-0.2204309	0.3890684	PRR3
cg04833845	0.3980954	-138.578	-0.2204728	0.6185682	KCNN4
cg26259865	0.5237428	-178.634	-0.2205465	0.7442893	LOC124220
cg10370591	0.3987846	-139.0545	-0.2207241	0.6195087	TPO
cg07297178	0.5386537	-187.6655	-0.2207637	0.7594174	CEACAM7
cg02882813	0.5446693	-191.4701	-0.2207749	0.7654443	CST5
cg01917648	0.3039936	-131.9014	-0.2210806	0.5250742	SPIC
cg05606799	0.4756788	-158.8863	-0.2211649	0.6968437	KISS1
cg24901474	0.330377	-132.8003	-0.2212434	0.5516204	RGS5
cg19353006	0.393215	-138.929	-0.2212466	0.6144616	TUSC3
cg11405695	0.3026225	-132.095	-0.2212477	0.5238702	ATAD3C
cg00895324	0.4847136	-162.4635	-0.221348	0.7060616	PCP4
cg20485165	0.4267237	-145.4461	-0.2214349	0.6481587	WFDC12
cg17412560	0.2654173	-133.3119	-0.2214994	0.4869167	CSEN
cg19006008	0.4156654	-143.2954	-0.2215274	0.6371928	F2RL3
cg15895197	0.178388	-144.3481	-0.221595	0.399983	EMILIN1
cg18390025	0.3738837	-136.9029	-0.2216735	0.5955572	ELOVL3
cg15075718	0.4087418	-142.2221	-0.2216809	0.6304227	MFRP
cg22077553	0.3868786	-138.8002	-0.221808	0.6086866	MYOC
cg12732155	0.2393463	-135.7098	-0.2219342	0.4612805	LAPTM5
cg15741706	0.4731185	-159.3699	-0.2219436	0.6950621	CXorf48
cg02786019	0.3606507	-135.894	-0.2219683	0.5826191	TRPV6
cg25691167	0.04070083	-202.0654	-0.2219864	0.2626873	FERD3L
cg10248727	0.5886211	-229.4772	-0.2219879	0.810609	LCN1
cg16739580	0.4106794	-143.3184	-0.2221889	0.6328683	POP2

cg00601486	0.6705068	-350.469	-0.2222289	0.8927357	H1T2
cg27244482	0.2905425	-133.3843	-0.2222358	0.5127783	CAMK2A
cg19382175	0.5050328	-172.9136	-0.2223135	0.7273464	PDE6A
cg14893161	0.2538249	-135.3	-0.2225766	0.4764015	FLJ32569
cg21621114	0.2638891	-134.8421	-0.2227072	0.4865962	CRX
cg02812142	0.425649	-147.3567	-0.2228001	0.6484491	ACMSD
cg17926869	0.5484484	-198.9128	-0.2228693	0.7713177	LOC115098
cg01255591	0.5950878	-238.8023	-0.2229213	0.8180091	R3HDML
cg27550918	0.4675472	-159.2038	-0.222922	0.6904692	SYNPO2L
cg08390209	0.3627245	-137.4501	-0.2229614	0.5856858	CDKN2B
cg07595943	0.6938281	-350.469	-0.2229644	0.9167925	LOC161931
cg03846767	0.6734681	-350.469	-0.2230432	0.8965113	TP73
cg18294257	0.1628645	-149.6021	-0.223061	0.3859255	SEC14L3
cg19998328	0.3412902	-135.9074	-0.2231663	0.5644566	LOC90580
cg21148892	0.4825572	-165.3569	-0.2233499	0.705907	CLEC4F
cg24054653	0.1542472	-152.2806	-0.223463	0.3777102	C1GALT1C1
cg19000186	0.4452021	-153.2951	-0.223464	0.668666	CNGA1
cg02192965	0.4669254	-160.0836	-0.2235477	0.690473	SLC3A1
cg01185080	0.326295	-135.6153	-0.2235739	0.5498688	ZNF710
cg14168975	0.3525207	-137.4733	-0.2236908	0.5762116	ALAS2
cg14102807	0.4722376	-162.1562	-0.2236947	0.6959323	CD19
cg19421044	0.2015512	-142.6539	-0.223737	0.4252881	DNASE1L1
cg25168545	0.262339	-136.1868	-0.2237565	0.4860955	GIMAP1
cg22202141	0.3774446	-140.4511	-0.2238827	0.6013273	FCGR3A
cg24621042	0.3188915	-135.8705	-0.2239954	0.5428869	SERPINA1
cg26796190	0.05051085	-197.9663	-0.2240167	0.2745275	PYY
cg08878744	0.2912505	-135.6637	-0.224087	0.5153375	LCE1B
cg03291145	0.4203442	-148.2392	-0.2241066	0.6444508	ARSF
cg22780475	0.3566891	-138.4712	-0.2241467	0.5808358	CBLC
cg26813458	0.6407934	-306.3069	-0.2242641	0.8650575	CEACAM6

cg01248426	0.2081108	-142.3241	-0.2242933	0.432404	ATP6V0D2
cg14107638	0.4519393	-156.7815	-0.2243821	0.6763214	MAGEA5
cg04761824	0.4716085	-163.2375	-0.2244179	0.6960264	30-nov
cg01375994	0.3303619	-136.9883	-0.2244889	0.5548508	MXRA5
cg03712237	0.5594022	-211.0533	-0.2245469	0.7839491	SSX2
cg15329483	0.3725325	-140.7861	-0.2245573	0.5970898	SSX7
cg14321743	0.4223771	-149.8163	-0.2248294	0.6472065	PLA2G2D
cg16792160	0.3340937	-137.6855	-0.2248518	0.5589455	ASAH2
cg01568736	0.5145674	-183.4541	-0.2251055	0.7396729	SERPINB7
cg27420123	0.4888001	-171.4801	-0.2252444	0.7140445	FSHB
cg23464269	0.3177448	-137.4953	-0.2252867	0.5430315	UGT1A3
cg05982504	0.4562955	-159.9022	-0.2254213	0.6817167	IGFALS
cg19787037	0.3232324	-138.0302	-0.2255231	0.5487555	SPAG11
cg12397274	0.2446659	-139.5977	-0.2255383	0.4702042	TINAG
cg17915429	0.4321337	-153.4325	-0.2256502	0.6577839	PGLYRP2
cg15206445	0.3990214	-146.4204	-0.2256929	0.6247143	SPG7
cg13859324	0.2747509	-138.0455	-0.225704	0.500455	UNC45B
cg27341860	0.665668	-350.469	-0.2257231	0.891391	OR2L13
cg04491443	0.644414	-319.8184	-0.2258722	0.8702862	PDILT
cg21066636	0.2480855	-139.8538	-0.2259824	0.474068	TM4SF5
cg20312687	0.3945848	-146.1126	-0.2260074	0.6205922	DEFB118
cg26062856	0.393006	-146.1908	-0.2262395	0.6192455	ATP10A
cg26644395	0.2356649	-141.4199	-0.226358	0.4620229	UCN3
cg01204985	0.5293819	-195.2106	-0.2265473	0.7559292	LILRA4
cg20856834	0.4060616	-149.2148	-0.226658	0.6327196	OR12D3
cg04891836	0.4178579	-151.9273	-0.2267911	0.644649	TNFSF14
cg18508525	0.5848864	-240.2222	-0.2268661	0.8117525	CD36
cg03608577	0.5489165	-209.2832	-0.2269486	0.7758651	OR12D3
cg08260891	0.06906583	-190.4687	-0.2270138	0.2960796	PPGB
cg12547930	0.3880421	-146.6199	-0.2270699	0.6151119	WFDC6
cg06731599	0.2264118	-143.2954	-0.2270923	0.4535041	ATP6AP2

cg14893129	0.6471527	-330.546	-0.2270989	0.8742516	CARD14
cg22909609	0.2748898	-139.8159	-0.2271266	0.5020164	ITGBL1
cg05955301	0.2836432	-139.6247	-0.2271416	0.5107849	PRELP
cg27337148	0.2093908	-145.5461	-0.2271637	0.4365545	CAMK1G
cg01353448	0.1494051	-158.0739	-0.2271987	0.3766038	C7orf16
cg14178895	0.2260703	-143.4921	-0.2272328	0.4533031	C6orf105
cg06323290	0.3913763	-147.499	-0.2272935	0.6186698	HK1
cg07531356	0.4331269	-156.5034	-0.2273617	0.6604886	INSL6
cg01072821	0.4005423	-149.9449	-0.2278243	0.6283666	UNQ9391
cg04245402	0.2704912	-141.0752	-0.2279941	0.4984854	C19orf21
cg08815403	0.3466347	-142.9428	-0.2280494	0.5746841	HSD17B13
cg22585988	0.4584843	-165.2658	-0.2280619	0.6865462	PVRL4
cg19055231	0.04946491	-203.7408	-0.2280768	0.2775417	STAC
cg15447486	0.4738173	-171.1187	-0.2282242	0.7020414	GPR109B
cg27285599	0.5332848	-201.7467	-0.2282956	0.7615805	FLJ13841
cg14587868	0.4178791	-154.5166	-0.2284462	0.6463253	TGM1
cg13552869	0.1691851	-154.8778	-0.2287038	0.3978889	SEZ6L2
cg07676849	0.3569158	-144.8804	-0.2287414	0.5856572	FOLR3
cg23216015	0.1517063	-159.3902	-0.2288068	0.3805131	C7orf16
cg18490846	0.2970306	-141.859	-0.2289323	0.5259629	C17orf73
cg18085517	0.3142168	-142.5998	-0.2292255	0.5434423	TRPM1
cg26504906	0.2535073	-143.5538	-0.2292751	0.4827824	PRSS16
cg05600717	0.07729743	-188.8728	-0.2293023	0.3065997	FLJ13639
cg23213217	0.3285882	-143.5173	-0.2294364	0.5580245	DEGS1
cg10300154	0.3962156	-151.6692	-0.2294572	0.6256728	MGC5297
cg14942312	0.4527502	-166.0073	-0.2295463	0.6822965	GPR119
cg02442161	0.5234693	-198.7226	-0.2296578	0.7531271	PI3
cg09142399	0.4262946	-158.6364	-0.2297082	0.6560028	CRYZ
cg10604646	0.3074246	-143.04	-0.2297236	0.5371482	RGS5
cg24824840	0.5601987	-225.9615	-0.2299104	0.790109	SHANK1
cg19881895	0.5335298	-205.9207	-0.2299796	0.7635094	SLC43A3

cg03169180	0.3950013	-152.3085	-0.2300307	0.625032	NLGN2
cg06980053	0.259859	-144.1233	-0.2300327	0.4898917	RASSF1
cg14186992	0.4283528	-159.7605	-0.2300752	0.6584281	HKR3
cg11750883	0.3291158	-144.5262	-0.2301613	0.5592771	C1orf42
cg16242770	0.5318554	-205.7489	-0.2303655	0.7622209	KRTAP17-1
cg00319692	0.2697401	-144.2093	-0.2304564	0.5001965	ATP6V0D2
cg17240454	0.5694994	-235.7678	-0.2304621	0.7999614	SPDEF
cg24959428	0.365558	-148.388	-0.2304627	0.5960206	GBP6
cg21274025	0.2675284	-144.5581	-0.2306631	0.4981915	PLA2G3
cg08157292	0.3506113	-147.1588	-0.2308022	0.5814134	PPP1R7
cg06946880	0.4836191	-180.3985	-0.230871	0.7144901	ATP6V1B1
cg03379131	0.05504336	-203.7453	-0.2309271	0.2859704	ADAM15
cg13916742	0.5541123	-224.6871	-0.2313242	0.7854365	SCGB1D1
cg03879730	0.3447264	-147.5306	-0.2314657	0.576192	PSMD4
cg01078434	0.5213423	-201.6425	-0.2314853	0.7528276	MAS1L
cg24407065	0.5717572	-240.962	-0.2315086	0.8032658	BLZF1
cg24697329	0.5079069	-194.2322	-0.2316728	0.7395797	ARHGEF4
cg09864990	0.563418	-233.6639	-0.2316956	0.7951136	GATA5
cg05254747	0.5080678	-194.3647	-0.2316956	0.7397634	SLC39A14
cg24546463	0.2308139	-148.6228	-0.2317802	0.4625942	MGC39715
cg06226384	0.1723251	-158.1263	-0.2319027	0.4042277	CACNG5
cg26895595	0.2727143	-146.0321	-0.231946	0.5046603	MAGEB3
cg18239253	0.3269853	-147.1349	-0.2321468	0.559132	DEFB32
cg27383362	0.4226432	-162.357	-0.2325335	0.6551768	ATAD3C
cg05131835	0.4209841	-161.9667	-0.2325535	0.6535376	GH2
cg09038914	0.5201944	-203.6998	-0.2326812	0.7528756	GFAP
cg03916421	0.193173	-155.1342	-0.2328525	0.4260255	LOC132321
cg01718139	0.5328691	-212.6636	-0.2329248	0.7657939	UNQ3033
cg10784090	0.5369083	-215.5983	-0.2329547	0.7698629	CLDN18
cg10746737	0.4721704	-179.7384	-0.2330126	0.7051829	HLA-DRB5

cg01993576	0.1566768	-163.2946	-0.2330798	0.3897566	SLC29A1
cg07115820	0.3438339	-149.8796	-0.2331717	0.5770056	EPX
cg04057858	0.3718692	-153.2178	-0.2331764	0.6050456	UNQ9391
cg01987509	0.3521137	-150.8182	-0.2332367	0.5853504	PGR
cg23444894	0.5094967	-198.7176	-0.2332936	0.7427903	UNQ5810
cg26884581	0.1640156	-161.8373	-0.233367	0.3973826	PYGM
cg21643191	0.3904382	-157.3048	-0.2338112	0.6242494	ABCB5
cg19971655	0.6606947	-350.469	-0.2342232	0.8949178	BSND
cg22082462	0.5194159	-207.3249	-0.2344349	0.7538508	CPNE6
cg15480475	0.4873033	-189.5432	-0.2344771	0.7217804	TUB
cg12332316	0.606167	-292.3145	-0.2345528	0.8407198	F12
cg00466436	0.2788324	-149.3391	-0.2345793	0.5134117	DEFB126
cg21723486	0.3540789	-153.1175	-0.234685	0.5887639	TP73L
cg26093687	0.5370523	-220.1971	-0.2347242	0.7717764	EIF3S2
cg18946226	0.1837406	-159.4097	-0.2348548	0.4185955	MYR8
cg24628744	0.169082	-162.6466	-0.2349898	0.4040718	H2AFY
cg07073964	0.4275088	-167.9727	-0.2350442	0.662553	PRSSL1
cg19421752	0.164652	-163.817	-0.2350898	0.3997418	SLC6A18
cg09207718	0.3315731	-151.9364	-0.2353383	0.5669113	CYP1A2
cg13320683	0.4267223	-168.4836	-0.2354748	0.6621972	RHOBTB1
cg09914304	0.5571808	-238.8554	-0.2355108	0.7926916	PRF1
cg08448751	0.425908	-168.3617	-0.2355343	0.6614423	SEMA3G
cg00367281	0.3983861	-161.7758	-0.2356345	0.6340206	CHRNB3
cg27513764	0.4278365	-169.1694	-0.2356845	0.6635211	EFCAB3
cg19257200	0.5113333	-205.3414	-0.2357237	0.7470571	SOX10
cg03956628	0.1636852	-164.9229	-0.2357785	0.3994637	MLH1
cg09923671	0.4466124	-175.4223	-0.2358457	0.682458	GATA5
cg18807515	0.3860365	-159.7345	-0.2358865	0.621923	PRAMEF2
cg26218269	0.2528329	-152.1447	-0.2359404	0.4887733	MAB21L2
cg09508556	0.6252576	-328.9825	-0.2359861	0.8612437	PSORS1C2
cg18129786	0.573794	-257.0817	-0.2360731	0.8098671	ZNF445

cg01731341	0.5571908	-240.5134	-0.2360815	0.7932723	FGF6
cg22021786	0.4747467	-188.2802	-0.2366839	0.7114305	WFDC8
cg00673191	0.2103111	-157.3381	-0.2367302	0.4470413	DOPEY2
cg23704362	0.31117	-152.7205	-0.2367839	0.547954	C8orf46
cg03343942	0.300002	-152.4041	-0.2368031	0.5368052	SLC39A5
cg02164442	0.3529468	-156.1535	-0.2368369	0.5897837	ITGAD
cg11819637	0.4118724	-166.8938	-0.2368511	0.6487235	THPO
cg06906435	0.2992482	-152.4665	-0.2368651	0.5361133	FLJ25773
cg20584011	0.5806308	-267.5251	-0.2369241	0.817555	ZDHHC11
cg18521925	0.657185	-350.469	-0.2372264	0.8944114	SLC22A16
cg07123548	0.5798149	-267.6395	-0.2372483	0.8170632	HIPK4
cg02157083	0.3934982	-163.4483	-0.2373166	0.6308148	APOA5
cg02187357	0.3975996	-164.3644	-0.2373447	0.6349444	TBC1D22B
cg25778166	0.4874338	-195.88	-0.2374244	0.7248582	FMO3
cg20993403	0.5481123	-236.3255	-0.2374579	0.7855701	EPB41L1
cg02723372	0.5555405	-243.326	-0.2375815	0.793122	RUNX3
cg01785568	0.3129162	-154.0842	-0.2377115	0.5506277	MSX1
cg12999109	0.551048	-239.6673	-0.2377301	0.7887781	ASAH3
cg22534509	0.2239673	-156.991	-0.2377955	0.4617628	GPR81
cg03918304	0.2091624	-158.9976	-0.2378957	0.4470581	HOXD10
cg05112299	0.2602146	-154.2795	-0.2379008	0.4981154	OR7A17
cg17091851	0.2894149	-153.9905	-0.2381026	0.5275174	LOC348174
cg20781967	0.1982175	-161.0235	-0.238184	0.4364015	NINJ2
cg23807646	0.3588181	-158.9404	-0.2381857	0.5970038	SLC26A8
cg09418321	0.2410604	-155.8481	-0.2381867	0.4792472	DYRK4
cg06885782	0.2498312	-155.4708	-0.2383767	0.4882079	KCNQ4
cg11584690	0.1227911	-180.5075	-0.2384175	0.3612086	ZNF574
cg22038738	0.3296824	-156.2153	-0.2384651	0.5681474	PLAT
cg23458892	0.4149408	-170.5291	-0.2385136	0.6534544	SIGLEC7
cg18462653	0.1660181	-168.079	-0.2387043	0.4047224	DEFB119
cg251934	0.3684269	-161.2496	-0.2387905	0.6072175	FLJ20186

94					
cg22341310	0.190056	-163.8006	-0.2392775	0.4293335	ZNF541
cg05330360	0.301807	-156.0494	-0.2394301	0.5412371	ZPBP2
cg25107791	0.3384545	-158.5418	-0.2395086	0.5779631	CLPS
cg26946769	0.541388	-236.626	-0.2396444	0.7810324	MAPK4
cg05135288	0.2355101	-158.368	-0.2397337	0.4752438	RHOT2
cg21505334	0.4901621	-202.623	-0.239877	0.7300391	CEACAM5
cg05440289	0.514272	-218.3749	-0.2404295	0.7547014	IVL
cg01837574	0.2947166	-157.2956	-0.2404404	0.535157	TRAPPC1
cg19903229	0.2949428	-157.3203	-0.2404604	0.5354032	C14orf105
cg02067021	0.4480022	-184.9252	-0.2405776	0.6885798	DNAJC5B
cg17298704	0.5588177	-255.5772	-0.2406176	0.7994353	CLDN18
cg15842430	0.3867767	-168.2115	-0.2410813	0.627858	FAM12B
cg08728865	0.5505217	-248.8612	-0.2410895	0.7916111	NALP7
cg03364504	0.6091297	-322.8816	-0.2411269	0.8502566	FLJ36116
cg07446846	0.5488917	-247.6201	-0.2411817	0.7900734	SLC6A8
cg02537838	0.5565517	-255.3952	-0.2413023	0.7978539	C20orf151
cg04713352	0.3348438	-160.9356	-0.2413767	0.5762205	ATP4A
cg07643942	0.3206114	-159.953	-0.2415137	0.5621251	LACRT
cg08626653	0.4694337	-195.9214	-0.2415659	0.7109996	FLJ37538
cg17803430	0.4244248	-178.9957	-0.2417209	0.6661457	C4BPA
cg09573795	0.3429434	-162.4536	-0.2418458	0.5847892	MSX1
cg18303397	0.2217949	-162.486	-0.2418576	0.4636526	MBD4
cg21495715	0.472999	-198.7034	-0.2420808	0.7150798	SLC5A10
cg12069042	0.4366885	-184.0883	-0.2422971	0.6789856	PLXNB1
cg14159672	0.1582636	-174.6849	-0.2423345	0.4005981	FLJ32569
cg05500074	0.464834	-195.4826	-0.2423487	0.7071827	TSKS
cg02658251	0.4059981	-174.8768	-0.2423705	0.6483686	DEFB4
cg15538820	0.6740784	-350.469	-0.2424057	0.9164841	OBP2B
cg10210238	0.2121375	-164.488	-0.2424712	0.4546087	CDKN2B
cg23065097	0.1974999	-166.7037	-0.242501	0.4400008	FKBP1B

cg04515986	0.2260182	-162.8931	-0.2425163	0.4685345	FTHL17
cg13705284	0.2704965	-160.249	-0.2425802	0.5130768	ACOX2
cg14652095	0.2684927	-160.3165	-0.2425975	0.5110902	HIST1H1A
cg27566805	0.2474818	-161.4414	-0.2427566	0.4902384	USH2A
cg11297236	0.2663648	-160.7157	-0.2428459	0.5092107	PDILT
cg08402568	0.4114048	-177.3787	-0.2429549	0.6543597	MGC34647
cg12954718	0.247919	-161.8373	-0.2430693	0.4909883	USP6
cg21453309	0.1904365	-168.7897	-0.2431801	0.4336165	FAM101A
cg09448880	0.3956542	-173.9296	-0.2433192	0.6389734	PGLYRP3
cg22983092	0.3522774	-165.9476	-0.2433562	0.5956336	KRT25A
cg17217677	0.4029818	-176.1888	-0.2435707	0.6465526	SMPD3
cg04567009	0.3484879	-165.8521	-0.2436203	0.5921081	FCGR3B
cg27020690	0.1095553	-192.3995	-0.2436385	0.3531938	TERC
cg23173910	0.5232614	-232.9153	-0.2436623	0.7669237	ACTG2
cg03544379	0.2485528	-162.7141	-0.243767	0.4923198	OR7C2
cg21519900	0.2856903	-161.9327	-0.2438761	0.5295665	C20orf186
cg27069753	0.2799405	-162.1543	-0.24405	0.5239905	ELA3B
cg09948350	0.5510276	-258.3488	-0.2440806	0.7951082	FLJ25084
cg16463460	0.2372132	-164.2157	-0.2442809	0.4814942	WT1
cg24816866	0.5032013	-220.5457	-0.2443091	0.7475104	PARK2
cg23765993	0.2361062	-164.5518	-0.2444631	0.4805694	SPINLW1
cg14934821	0.5747538	-286.7419	-0.2446855	0.8194393	GPSM1
cg24992780	0.5538051	-263.5718	-0.2448662	0.7986712	OR7C1
cg19096475	0.3768258	-172.5821	-0.244946	0.6217718	ASAM
cg15379858	0.6441248	-350.469	-0.2451116	0.8892364	ChGn
cg14902389	0.4768724	-207.2202	-0.2451409	0.7220133	MGAT4A
cg06531741	0.2904756	-164.0804	-0.2453489	0.5358245	HTR3B
cg01861509	0.4317928	-188.6806	-0.2455741	0.6773669	SPOCK2
cg11653864	0.2254426	-167.1457	-0.2456401	0.4710826	ELK1
cg19240938	0.5281997	-242.2229	-0.2456617	0.7738615	SLC22A18
cg041387	0.5349195	-248.0652	-0.2457111	0.7806306	SPRR3

56					
cg19481686	0.3840538	-175.5132	-0.2457956	0.6298494	CDKN2B
cg25087423	0.6636174	-350.469	-0.2459828	0.9096003	BLR1
cg06275635	0.3128447	-165.9847	-0.245998	0.5588427	PGLYRP3
cg25013053	0.4509612	-197.3979	-0.2462317	0.6971928	UNC45B
cg18530716	0.09467848	-202.202	-0.2462681	0.3409466	SLC16A11
cg25659818	0.3291696	-167.7186	-0.2462727	0.5754424	CCL4
cg16466334	0.4100572	-182.9996	-0.2462982	0.6563554	MMP3
cg03021690	0.593831	-319.8875	-0.2464244	0.8402554	TNFAIP2
cg06985415	0.6004899	-331.1423	-0.2465742	0.847064	C10orf39
cg00962459	0.3557386	-171.724	-0.2467032	0.6024418	PROKR1
cg04323365	0.4973292	-222.8962	-0.2468349	0.744164	GJB1
cg26385286	0.2258084	-168.7457	-0.246855	0.4726635	GCNT2
cg18988110	0.2134576	-170.2199	-0.2468849	0.4603425	ATAD4
cg11456838	0.2980641	-166.9359	-0.2471633	0.5452273	LOC202459
cg01053621	0.3207855	-168.3559	-0.2471961	0.5679817	APOA2
cg26661623	0.3866247	-178.6838	-0.2472849	0.6339096	ASGR2
cg12582965	0.6114747	-350.469	-0.2473949	0.8588696	ATP10A
cg16219122	0.2344775	-168.9822	-0.2476175	0.482095	ABCB1
cg01982597	0.4348948	-193.9898	-0.2477122	0.682607	PGBD3
cg00436603	0.3008222	-167.9553	-0.2477767	0.5485989	CYP2E1
cg06720660	0.3553964	-173.6592	-0.247926	0.6033224	RNASE6
cg00134539	0.4618022	-206.0231	-0.2480464	0.7098486	UBASH3A
cg27157038	0.5018852	-228.981	-0.2480952	0.7499803	DNTT
cg14297029	0.5935653	-326.4046	-0.2481515	0.8417169	SSTR3
cg01074640	0.3988083	-183.2555	-0.2481788	0.6469871	IFNA17
cg14034870	0.3353612	-171.6161	-0.2483779	0.5837391	SFTPG
cg00518911	0.2655599	-168.4444	-0.2484068	0.5139667	HOXA10
cg25903122	0.500747	-229.0698	-0.2484407	0.7491877	MGC2747
cg09546307	0.4259551	-192.1683	-0.2484415	0.6743965	CLEC4D
cg04719766	0.5353846	-256.7187	-0.2485443	0.7839289	KCNQ1

cg13928961	0.4532018	-203.1904	-0.248583	0.7017848	K6IRS3
cg21207418	0.4829552	-218.5247	-0.2486781	0.7316332	ACP5
cg12970081	0.3268791	-171.3891	-0.2488191	0.5756982	GPR32
cg24816455	0.229274	-171.1567	-0.2488639	0.4781379	SEMA3B
cg18982568	0.342907	-174.3924	-0.2495267	0.5924338	KRT1B
cg15060813	0.2596911	-170.505	-0.2497362	0.5094273	LRFN3
cg22784047	0.436831	-198.7726	-0.2497669	0.6865979	MVP
cg18396533	0.1689048	-182.3156	-0.2500283	0.4189331	DYDC1
cg26349773	0.4950125	-229.4939	-0.2501597	0.7451722	ATP6V0A4
cg10787197	0.3211367	-173.5085	-0.250536	0.5716727	C6orf105
cg01657380	0.419087	-194.3141	-0.2507852	0.6698722	NPFF
cg03752087	0.4350218	-200.3351	-0.250894	0.6859157	CASP14
cg08784110	0.2888319	-172.25	-0.2509851	0.5398171	MAS1
cg13126790	0.4287842	-198.1838	-0.2510101	0.6797944	FLJ27255
cg01693350	0.2415685	-173.1904	-0.2510163	0.4925848	WT1
cg02601403	0.5183059	-248.9881	-0.2510722	0.7693781	TBC1D3C
cg09283007	0.6532625	-350.469	-0.2511034	0.9043659	FAM47B
cg13608094	0.61296	-350.469	-0.2511694	0.8641294	CCND1
cg22083047	0.2326516	-174.1648	-0.2512364	0.483888	PRICKLE2
cg19561774	0.6079551	-350.469	-0.2512907	0.8592458	SLC22A2
cg00075967	0.4639589	-214.2221	-0.2513033	0.7152622	STRA6
cg23471482	0.5358982	-265.7744	-0.2514105	0.7873088	CCL22
cg16899306	0.370281	-182.2632	-0.2514538	0.6217347	HLA-DQB2
cg07173760	0.2599814	-172.9883	-0.2514854	0.5114667	CLC
cg15914863	0.5425782	-272.978	-0.2515869	0.7941651	CYP2W1
cg11346450	0.4750205	-221.1575	-0.2517585	0.726779	UGT1A3
cg08292050	0.5060157	-241.7079	-0.2519338	0.7579495	SOCS4
cg18056600	0.1128821	-202.202	-0.2520581	0.3649402	ZMYND15
cg06437862	0.5080656	-243.5998	-0.2520648	0.7601305	TUBA2
cg01144251	0.4812288	-225.5498	-0.2520828	0.7333116	KLK9
cg18920397	0.450542	-209.5667	-0.2521673	0.7027093	LY9

cg13271951	0.272992	-173.9083	-0.2522524	0.5252445	FAM57B
cg03860768	0.3915631	-188.8807	-0.2523434	0.6439065	BLK
cg07977490	0.4364668	-203.8423	-0.2523628	0.6888297	C16orf45
cg08766149	0.4336153	-202.8068	-0.2524091	0.6860244	GZMB
cg15589427	0.4820145	-227.0702	-0.2525258	0.7345403	MUC4
cg07426848	0.4598409	-214.8225	-0.2525346	0.7123755	S100A3
cg11472424	0.4133179	-195.8745	-0.2526162	0.6659341	PFKFB1
cg18533225	0.4365512	-204.5991	-0.2527046	0.6892558	KLHDC7B
cg12022621	0.2978632	-175.4437	-0.2528761	0.5507392	LAX1
cg19033555	0.3333549	-178.8177	-0.2530341	0.5863889	DEFB1
cg00689010	0.4006442	-193.9359	-0.2536785	0.6543227	NCSTN
cg23114594	0.455311	-215.3301	-0.2537924	0.7091034	C10orf30
cg13019092	0.478888	-228.4473	-0.2538905	0.7327785	PDZK1
cg14826683	0.2867484	-176.619	-0.253939	0.5406874	SPRR2D
cg05078019	0.2828536	-176.7846	-0.2541154	0.536969	PDE6B
cg23776892	0.522525	-261.439	-0.2541476	0.7766726	MAGEA1
cg21964481	0.4046157	-196.0096	-0.2541641	0.6587797	SLC34A3
cg24888049	0.2036866	-181.6637	-0.2542319	0.4579185	FES
cg24489034	0.4367154	-207.99	-0.2543154	0.6910307	LW-1
cg21755709	0.222721	-179.519	-0.25435	0.477071	C21orf124
cg16358738	0.4700245	-224.5353	-0.2544258	0.7244502	AGXT
cg08859675	0.3760654	-189.1106	-0.2546256	0.6306909	PDE4A
cg25645748	0.3890903	-192.7717	-0.254829	0.6439193	AKAP14
cg05696092	0.2299233	-179.5553	-0.2548561	0.4847794	NOSIP
cg25072962	0.3704724	-188.2802	-0.2548618	0.6253342	MGC35295
cg08834018	0.2631145	-177.9915	-0.2549482	0.5180627	PRODH
cg01726767	0.3634303	-187.1849	-0.2550918	0.6185221	LALBA
cg06625767	0.5171898	-259.8574	-0.2552256	0.7724154	F12
cg24477636	0.306934	-179.6842	-0.2552442	0.5621782	OR10H1
cg24027679	0.5204741	-263.527	-0.2554898	0.7759639	SLC2A7
cg211688	0.4956901	-243.6919	-0.2555763	0.7512664	C6orf122

84					
cg19372178	0.2150873	-182.7201	-0.2560424	0.4711297	TMEM16G
cg20822628	0.6734058	-350.469	-0.256205	0.9296108	GATA5
cg25477904	0.5516633	-298.4139	-0.2562312	0.8078945	PSG1
cg06489008	0.2970546	-180.5617	-0.2562355	0.55329	CST11
cg01550148	0.2911688	-180.3372	-0.2562623	0.5474311	H2AFY
cg00684178	0.471098	-229.6548	-0.2563681	0.7274661	NEU4
cg18473117	0.4413993	-214.7406	-0.2565359	0.6979352	CCDC22
cg22220722	0.516949	-263.5109	-0.2565585	0.7735075	PLA2G2A
cg17386181	0.4762729	-233.5023	-0.2566814	0.7329543	MT1B
cg25141490	0.2464841	-181.2068	-0.2567779	0.503262	IL17B
cg24743310	0.591197	-350.469	-0.2568502	0.8480471	FLJ31196
cg20576002	0.3515007	-188.4861	-0.2571746	0.6086753	FAM112B
cg24851490	0.247068	-181.7743	-0.2571788	0.5042468	RNASE2
cg23075286	0.3679128	-191.9128	-0.2572365	0.6251493	GALP
cg08420900	0.600529	-350.469	-0.2575591	0.858088	LW-1
cg04968473	0.300234	-182.8326	-0.2575658	0.5577998	CYP1A2
cg22971191	0.259192	-181.9662	-0.2575839	0.516776	SLC10A2
cg20095587	0.4570144	-224.9397	-0.2576939	0.7147083	TREM2
cg03055440	0.3891114	-198.6098	-0.2579857	0.6470971	MS4A6A
cg07220939	0.3666997	-193.0943	-0.2580534	0.6247531	SLC22A12
cg08996986	0.4200295	-209.0726	-0.2580968	0.6781263	EPS8L1
cg22189286	0.4325705	-214.7584	-0.2583822	0.6909527	HSPB8
cg06142324	0.3757302	-195.8142	-0.2583958	0.634126	FLJ25530
cg24429836	0.201429	-187.9692	-0.258433	0.459862	LDHD
cg00714377	0.1392918	-201.8851	-0.2585455	0.3978373	SLA2
cg08093398	0.3934182	-200.928	-0.2585501	0.6519684	PSF1
cg09447105	0.5798902	-347.2225	-0.2585836	0.8384738	PDE6H
cg15903395	0.3963731	-201.8981	-0.2586021	0.6549752	FLJ25369
cg22013966	0.5823739	-350.469	-0.2586112	0.8409851	SERPINA13
cg21122774	0.472607	-236.4069	-0.2588	0.7314069	SARDH

cg20189782	0.4508843	-224.3942	-0.2588555	0.7097399	MGC27121
cg09426307	0.3881746	-200.7607	-0.2592744	0.647449	SEC14L3
cg17095731	0.3836071	-199.5312	-0.2593061	0.6429132	LRP8
cg18231267	0.6246328	-350.469	-0.2593465	0.8839793	RUNX3
cg02694395	0.285348	-184.9554	-0.2594073	0.5447552	FMO4
cg00042156	0.3353192	-190.5684	-0.2599726	0.5952918	MGC16291
cg01970325	0.277803	-185.8924	-0.2601439	0.5379469	NELF
cg22247240	0.3487194	-193.177	-0.2601794	0.6088988	C14orf115
cg10125195	0.4302162	-217.6325	-0.2602237	0.6904398	LACRT
cg17264470	0.594126	-350.469	-0.2602262	0.8543522	FGF21
cg23753610	0.6697737	-350.469	-0.2604167	0.9301904	DNAHL1
cg06351503	0.4261608	-216.4185	-0.2604675	0.6866283	RDBP
cg13726463	0.4765437	-245.7687	-0.2615245	0.7380682	COX6A2
cg24363955	0.523945	-285.492	-0.2616205	0.7855654	FLJ14054
cg19728577	0.5110757	-273.868	-0.2618805	0.7729563	GUCA2B
cg02324920	0.2632822	-188.4749	-0.2618917	0.5251739	NEURL
cg26420196	0.2101865	-191.8644	-0.2619399	0.4721264	GAS6
cg26415633	0.2056006	-192.5482	-0.2620153	0.4676159	KLK1
cg07950803	0.2450307	-189.3452	-0.2621948	0.5072255	CD1A
cg24623694	0.1951702	-194.5628	-0.262372	0.4575422	PRX
cg05636175	0.1674595	-200.2601	-0.2626902	0.4301497	TNFRSF10C
cg27418851	0.3963546	-210.127	-0.2628559	0.6592104	MBL2
cg10490064	0.4525074	-235.1288	-0.2631733	0.7156807	CRYBB2
cg16272420	0.4574497	-238.1383	-0.2632618	0.7207115	PNLIPRP2
cg23873703	0.5164387	-283.136	-0.263265	0.7797037	KCNAB1
cg00392257	0.3396089	-196.9959	-0.2633263	0.6029352	ISG20L2
cg23580945	0.4864501	-257.8185	-0.2634188	0.7498689	FLJ43826
cg08341924	0.4947991	-264.6557	-0.2635272	0.7583263	TGM1
cg03872376	0.3723267	-204.7002	-0.2637119	0.6360386	ZP4
cg27377450	0.5430413	-314.2912	-0.2637666	0.8068079	ARHGEF18

cg21402035	0.3383627	-197.6902	-0.2638409	0.6022036	GALR3
cg27090216	0.09419132	-226.3992	-0.2638991	0.3580904	TNFRSF10C
cg17192247	0.09344193	-226.793	-0.2639394	0.3573814	MAPRE3
cg00941549	0.4039969	-215.2363	-0.2640862	0.6680831	AKAP4
cg17687962	0.4731542	-250.1533	-0.2641241	0.7372782	KLK3
cg04816348	0.6021244	-350.469	-0.2641432	0.8662676	CLEC4G
cg20011352	0.0900793	-228.9249	-0.2643433	0.3544226	GPR124
cg25915982	0.5115225	-281.7162	-0.2643436	0.7758661	GRB10
cg17001430	0.4744905	-252.2012	-0.2645606	0.7390511	KIF25
cg15711744	0.4976214	-270.9095	-0.2649019	0.7625234	ANP32D
cg08887581	0.4313168	-228.4076	-0.2649466	0.6962634	C1orf64
cg26473272	0.6019943	-350.469	-0.2653947	0.867389	SYT8
cg18138484	0.5603299	-344.6118	-0.2654948	0.8258247	CABP2
cg12061127	0.4032373	-217.8873	-0.2655274	0.6687647	WFDC9
cg20488657	0.4256143	-227.3873	-0.2656832	0.6912975	TFF3
cg21038703	0.3919985	-214.3934	-0.2657152	0.6577138	ASB16
cg11070419	0.2822882	-194.8604	-0.2657251	0.5480133	C4BPA
cg13530039	0.26776	-194.6775	-0.2658734	0.5336334	CHRM1
cg00415993	0.1858257	-201.2009	-0.2659039	0.4517296	F2RL2
cg10408410	0.445033	-237.814	-0.266052	0.711085	RLBP1
cg00350478	0.3383234	-202.0586	-0.2663552	0.6046786	FRMD1
cg00622552	0.1129912	-221.9673	-0.2664249	0.379416	ODF3L1
cg19384697	0.2339588	-196.6249	-0.2666249	0.5005838	UPK3B
cg08453096	0.5591764	-347.7715	-0.266716	0.8258924	ABCG5
cg02833725	0.3855561	-214.5343	-0.2668406	0.6523967	ISG20L2
cg25462291	0.09892453	-228.785	-0.2671052	0.3660297	HEYL
cg14150666	0.5678192	-350.469	-0.2676107	0.8354299	IL8RB
cg19226099	0.2856591	-198.1333	-0.2676557	0.5533149	MC3R
cg23881725	0.1617249	-209.1197	-0.2679588	0.4296838	DLEC1
cg01305625	0.2605624	-198.1258	-0.2681008	0.5286632	PDLIM4
cg221901	0.4472305	-244.034	-0.2681509	0.7153814	NALP8

14					
cg04048249	0.3896054	-218.5119	-0.268203	0.6578084	APOC3
cg21450627	0.5474451	-336.5656	-0.2682137	0.8156587	PSD4
cg05556202	0.4897697	-273.915	-0.2682496	0.7580193	TM4SF19
cg16673198	0.3691917	-212.596	-0.2683289	0.6375206	CPNE4
cg03752885	0.2565101	-198.9524	-0.268638	0.5251481	DAPK3
cg21407055	0.5179369	-302.5053	-0.268835	0.7867719	ART1
cg11884243	0.52402	-310.2112	-0.2691101	0.7931302	FCN2
cg17829936	0.4159941	-231.2552	-0.2694478	0.6854419	TAAR5
cg25033144	0.4631173	-257.4015	-0.2695189	0.7326362	FLJ00060
cg18121684	0.4103693	-229.4939	-0.2697313	0.6801006	SERPINB13
cg06277277	0.2157061	-202.9239	-0.2698414	0.4855476	NR1I3
cg11161873	0.2654936	-201.1889	-0.2700083	0.535502	FLJ39575
cg03468463	0.375702	-217.8812	-0.2700992	0.6458012	SERPINB12
cg08603768	0.3859621	-221.1645	-0.2701196	0.6560817	WNT8A
cg10986043	0.2568996	-201.3929	-0.2701824	0.527082	TCAP
cg18204685	0.4158792	-232.8926	-0.2702098	0.686089	BTD
cg05654163	0.3601753	-214.0182	-0.2703059	0.6304811	SLC39A2
cg00520135	0.5850471	-350.469	-0.2704716	0.8555187	TPM1
cg24919884	0.3874526	-222.461	-0.2705154	0.657968	ARHGEF16
cg12113132	0.2770408	-202.362	-0.2705479	0.5475886	CCNDBP1
cg16051685	0.4646289	-261.28	-0.2705956	0.7352245	TRIM63
cg24910675	0.1435033	-217.7036	-0.2706078	0.4141111	ENG
cg17582777	0.3487559	-211.8756	-0.2706386	0.6193945	EFNA3
cg03458191	0.4788628	-272.1026	-0.2707046	0.7495673	SAA1
cg14036856	0.2917149	-203.7798	-0.2708687	0.5625836	MGC52423
cg22228134	0.3100502	-205.8798	-0.2709991	0.5810493	GZMH
cg01459162	0.5187187	-310.6497	-0.271014	0.7897327	PADI3
cg14366490	0.3665084	-217.0895	-0.2710498	0.6375582	TXNL6
cg04457051	0.3335934	-210.0453	-0.2713194	0.6049128	SCOC
cg22294577	0.5330402	-328.9382	-0.2713354	0.8043756	SLC26A3

cg24506604	0.4350434	-245.1766	-0.2714458	0.7064892	LOC144501
cg05093686	0.2050186	-206.7971	-0.2716223	0.4766409	MAB21L1
cg26422060	0.5143065	-308.1748	-0.2717351	0.7860416	TBX10
cg13960126	0.3694419	-219.3238	-0.2717857	0.6412276	CRB3
cg03782453	0.1125455	-229.8505	-0.2718374	0.3843829	FLJ90575
cg04953015	0.4416723	-250.234	-0.2720069	0.7136792	CHRNA2
cg25982743	0.1646797	-214.8225	-0.2723353	0.437015	TIMP4
cg02351381	0.1916922	-209.9772	-0.2725022	0.4641945	C12orf34
cg15782391	0.2960484	-206.9271	-0.2725101	0.5685586	ACPT
cg11435943	0.2463514	-205.7734	-0.2728633	0.5192146	SERPINB12
cg19982860	0.3565557	-218.0323	-0.27292	0.6294757	IFNA21
cg12992720	0.1915257	-211.1852	-0.2733099	0.4648356	EDG4
cg14911395	0.1975895	-210.3238	-0.2733194	0.4709089	SEMA3B
cg23514672	0.5993263	-350.469	-0.2733511	0.8726774	FLJ32871
cg22805308	0.2504524	-206.4786	-0.2733615	0.5238139	PLEKHG5
cg07597976	0.3119079	-210.2454	-0.2733864	0.5852943	CD19
cg19717326	0.1739146	-214.5343	-0.2734389	0.4473534	MYADM
cg11739626	0.4268191	-245.7746	-0.2735413	0.7003604	AKT1S1
cg21985470	0.2608606	-207.9341	-0.2742492	0.5351098	PKLR
cg00174500	0.2099629	-210.5802	-0.2744811	0.4844441	CMTM5
cg18223379	0.2442207	-209.3558	-0.2750837	0.5193044	BPIL3
cg16545105	0.3818629	-230.0819	-0.2751986	0.6570615	CRHBP
cg19233472	0.4470837	-261.7956	-0.275271	0.7223547	FOXI1
cg15422147	0.4131543	-243.091	-0.2752992	0.6884536	SERPINB5
cg24825722	0.3202558	-215.1977	-0.2754617	0.5957175	ACADVL
cg24852661	0.1151843	-234.3016	-0.275599	0.3907833	GOLPH2
cg00644033	0.6414136	-350.469	-0.2761528	0.9175664	MUC3B
cg05671018	0.4032263	-240.5134	-0.2761557	0.679382	LYSMD2
cg25020204	0.3014529	-214.7228	-0.2766739	0.5781268	DBH
cg00321478	0.3797718	-232.5508	-0.2767251	0.6564969	CRB1
cg046373	0.326248	-218.6564	-0.2768114	0.6030594	FLJ32784

72					
cg02981703	0.5328804	-350.469	-0.2771741	0.8100545	CA6
cg08972170	0.2793976	-213.7938	-0.2773078	0.5567054	Ells1
cg02218324	0.3725599	-231.5379	-0.2774215	0.6499814	RSHL1
cg18967533	0.261448	-213.9401	-0.277874	0.539322	KLK6
cg04574507	0.370989	-232.091	-0.2779374	0.6489264	CD1B
cg20311730	0.3498144	-226.6553	-0.2782599	0.6280743	NALP10
cg12951282	0.3321485	-222.5885	-0.2782963	0.6104448	ASGR2
cg06958211	0.3189024	-220.3961	-0.278459	0.5973614	PAK6
cg05921699	0.3547309	-228.8716	-0.2787173	0.6334482	CD79A
cg07922606	0.2160703	-216.9141	-0.278886	0.4949563	HIST1H3E
cg00626119	0.2578932	-215.5106	-0.2788933	0.5367865	NTRK1
cg06303238	0.3733299	-234.8929	-0.2789036	0.6522335	SALL4
cg15303841	0.3221534	-221.8912	-0.2789561	0.6011094	RFPL1
cg15743985	0.4250975	-259.2743	-0.2794965	0.704594	CD22
cg21639401	0.3843788	-240.2838	-0.2795619	0.6639407	FLJ31222
cg10779183	0.4625717	-284.4727	-0.2796093	0.7421809	ELA3A
cg04337944	0.1989488	-220.0568	-0.2797503	0.4786991	FBLN1
cg24331162	0.4346772	-265.6363	-0.2797863	0.7144635	SYT8
cg06244417	0.4949653	-314.2912	-0.2798207	0.774786	FCN1
cg25372195	0.3782415	-238.5875	-0.2798408	0.6580823	DCD
cg24884084	0.150214	-230.1787	-0.2803144	0.4305283	SPRR1B
cg06233503	0.2835387	-219.4741	-0.2804668	0.5640055	KCNQ1
cg10213812	0.582496	-350.469	-0.2805152	0.8630112	FOXN1
cg03548857	0.180439	-224.3152	-0.2806796	0.4611185	FFAR2
cg03973663	0.1508814	-231.0237	-0.2809725	0.4318539	LYN
cg24541550	0.2919581	-222.0546	-0.281466	0.5734241	MRVI1
cg22627427	0.5636422	-350.469	-0.2814867	0.8451289	C11orf9
cg01663968	0.06200653	-268.9789	-0.2815111	0.3435176	CTSZ
cg26583078	0.3793765	-243.091	-0.2817276	0.6611041	SORBS2
cg09458237	0.4121009	-258.8009	-0.2821811	0.694282	HSPA12B

cg10071275	0.2983076	-224.233	-0.282233	0.5805405	MYT1
cg07484827	0.2184968	-222.0773	-0.282257	0.5007538	CHRNA10
cg22158923	0.2342943	-221.3058	-0.2822893	0.5165837	FLJ39237
cg19047670	0.3862455	-247.3097	-0.28242	0.6686655	CCND1
cg26581729	0.1695907	-229.9123	-0.2830366	0.4526273	NPDC1
cg25882366	0.1012537	-251.2368	-0.2831535	0.3844072	HOXB2
cg01772980	0.3407107	-234.0951	-0.2832602	0.6239709	SCGB1D1
cg12850636	0.4316413	-272.9081	-0.283335	0.7149763	TJP3
cg06236276	0.4745902	-306.2483	-0.2835126	0.7581028	SLC22A2
cg00594952	0.2814094	-224.7192	-0.2835425	0.564952	RIMS3
cg21129531	0.5376345	-350.469	-0.2837171	0.8213516	LRRC4
cg19859270	0.3990905	-256.3125	-0.2838551	0.6829456	GPR15
cg17778867	0.392306	-253.6482	-0.2840637	0.6763697	KRTAP10-8
cg19910382	0.311208	-230.0437	-0.2844008	0.5956088	FABP1
cg27622610	0.2240705	-225.3578	-0.2844642	0.5085348	OR1G1
cg09300114	0.1822392	-229.8939	-0.2844928	0.466732	SLC16A5
cg10322876	0.3389809	-236.16	-0.2845002	0.6234811	CYP2B6
cg01484156	0.2654594	-225.5889	-0.2846653	0.5501247	NCALD
cg25400358	0.2779229	-226.793	-0.284882	0.5628049	GPR137
cg23260026	0.2213237	-226.3992	-0.2850023	0.506326	FSTL3
cg19623751	0.3314493	-236.3255	-0.2855172	0.6169665	CEACAM7
cg04962134	0.3138452	-233.9226	-0.2861958	0.600041	TRIM51
cg23278885	0.4186796	-272.9428	-0.2864085	0.7050881	TGM6
cg05700681	0.5042711	-347.7397	-0.2864313	0.7907025	CCL22
cg00750606	0.2530096	-228.2885	-0.2864758	0.5394855	CDA
cg12781568	0.2704202	-229.1467	-0.2865468	0.556967	WT1
cg05569220	0.2938877	-231.4275	-0.286551	0.5804387	FLJ44861
cg13694749	0.3979414	-262.322	-0.286616	0.6845574	SCN4A
cg18242139	0.42658	-278.8625	-0.2868308	0.7134107	ELAVL4
cg08244028	0.3655253	-250.6076	-0.2875309	0.6530562	MSH3
cg13656062	0.4638909	-311.0428	-0.2882069	0.7520977	CYP4F2

cg00334507	0.336059	-242.9607	-0.2882439	0.6243029	MVP
cg09299388	0.4087616	-272.1511	-0.2883258	0.6970874	PGK2
cg19985911	0.4675846	-315.7811	-0.2886543	0.7562389	AVIL
cg09467501	0.08321916	-268.4623	-0.288894	0.3721131	PYY
cg03104936	0.349359	-248.1847	-0.2889345	0.6382935	GRB10
cg13053608	0.2217613	-233.3308	-0.2892288	0.5109901	LGP1
cg17820828	0.4348981	-292.0787	-0.2896423	0.7245404	KCNQ1
cg19368582	0.3569968	-253.0837	-0.2900355	0.6470323	MMRN2
cg19712821	0.2759593	-237.3123	-0.2908392	0.5667985	KSP37
cg02717866	0.3605849	-256.2002	-0.2908547	0.6514395	FLJ32771
cg05912121	0.3684546	-260.7738	-0.2915477	0.6600023	TH
cg22575540	0.4639342	-322.7798	-0.2919251	0.7558594	TRIM54
cg12334759	0.4059143	-279.9907	-0.2920558	0.6979702	C19orf19
cg21432842	0.2462023	-237.8602	-0.292085	0.5382873	CSF3
cg09701102	0.4607534	-321.2265	-0.2923684	0.7531219	NDUFV1
cg10677144	0.4188025	-288.8155	-0.2924452	0.7112477	MYOM1
cg07412254	0.3390306	-252.5694	-0.2924674	0.631498	FLJ14816
cg12775613	0.3366755	-252.0372	-0.2925326	0.6292081	HTR1F
cg02844051	0.4434413	-307.5853	-0.2927933	0.7362346	ZD52F10
cg18063149	0.3974466	-277.2419	-0.2928142	0.6902608	FMO3
cg07408456	0.3136784	-247.2138	-0.2929843	0.6066627	PGLYRP2
cg22194129	0.2960059	-244.3114	-0.2931924	0.5891984	CLEC4C
cg19949550	0.4452306	-311.3914	-0.2935925	0.7388231	ASB2
cg10052840	0.2014565	-242.3686	-0.2936686	0.4951251	SEMA6B
cg27394486	0.5062947	-350.469	-0.2945266	0.8008213	C15orf2
cg07792737	0.5176647	-350.469	-0.2946516	0.8123163	NPIP
cg04132607	0.5640612	-350.469	-0.2950631	0.8591244	GATA5
cg08475088	0.5533689	-350.469	-0.2951298	0.8484987	NALP9
cg16175725	0.5058921	-350.469	-0.2953535	0.8012456	TCF1
cg08424423	0.5054623	-350.469	-0.2954152	0.8008775	CDSN
cg04505023	0.4042006	-288.019	-0.2955423	0.6997429	SPRR1A

cg05215575	0.2692997	-245.8013	-0.2957442	0.5650438	FLJ25410
cg15996947	0.3217795	-255.0472	-0.295865	0.6176445	L2HGDH
cg18729973	0.4498937	-322.5797	-0.2959332	0.7458268	TFF1
cg00563932	0.410594	-294.1314	-0.2963617	0.7069557	PTGDS
cg19717150	0.3708904	-273.0597	-0.2964049	0.6672953	HNF4A
cg07651914	0.27646	-248.464	-0.2967588	0.5732188	CLDN15
cg09736922	0.4938422	-350.469	-0.2972735	0.7911158	THPO
cg11158374	0.4077763	-294.8553	-0.2972935	0.7050698	TFF2
cg17741572	0.4996379	-350.469	-0.2974017	0.7970396	CFB
cg06811800	0.3777986	-278.9435	-0.297526	0.6753246	ATP4B
cg15780361	0.2537917	-248.1199	-0.2975991	0.5513908	ALS2CR11
cg14333565	0.4937099	-350.469	-0.2977567	0.7914666	NRTN
cg15531099	0.2361316	-252.2012	-0.3001226	0.5362542	LCE1D
cg25710140	0.3890763	-295.0459	-0.3015749	0.6906511	MID1
cg18790143	0.3976119	-304.7707	-0.3032942	0.7009061	OTOS
cg12456510	0.3761174	-292.8861	-0.3034454	0.6795629	TFF2
cg19845843	0.4059637	-312.4424	-0.3041026	0.7100663	CXorf20
cg09414535	0.29913	-267.9784	-0.3045683	0.6036983	GRIP1
cg10575735	0.2959522	-267.864	-0.3048317	0.6007839	SSX4
cg03453449	0.3100609	-271.1938	-0.3048571	0.6149181	USP44
cg07339138	0.2683899	-263.5974	-0.3049633	0.5733532	CCDC13
cg17357062	0.5206921	-350.469	-0.3050629	0.8257549	FCN1
cg02423618	0.2862024	-267.0038	-0.3053141	0.5915165	SPATA8
cg06436504	0.3638363	-292.7789	-0.3058044	0.6696407	DOC1
cg08471713	0.5292574	-350.469	-0.3059983	0.8352556	MEOX1
cg18979223	0.3545418	-289.1455	-0.3060595	0.6606013	CDKN2B
cg10569414	0.3829961	-304.6054	-0.3064837	0.6894798	C21orf121
cg05670596	0.1807903	-268.0518	-0.3070258	0.4878161	CCRL2
cg11719283	0.3511111	-291.1681	-0.3074857	0.6585968	ZNF574
cg25957124	0.219695	-266.0245	-0.3075239	0.5272189	DNAH3
cg27442349	0.3252627	-282.461	-0.3079419	0.6332046	NFKBIB

cg24490338	0.4117915	-328.3008	-0.3081048	0.7198963	TPM3
cg14706739	0.3841461	-310.5495	-0.3084299	0.6925761	EPB49
cg14238120	0.4036578	-325.0606	-0.3090419	0.7126997	ELA3A
cg24355048	0.3283973	-286.0563	-0.3090433	0.6374406	CTSG
cg21065959	0.3495831	-294.7029	-0.3091974	0.6587805	LCE1E
cg03003745	0.2807887	-274.2547	-0.3092883	0.590077	UNQ473
cg15503752	0.4202399	-339.2592	-0.3094893	0.7297292	ST6GALNAC1
cg19917856	0.2033027	-270.4491	-0.3095531	0.5128558	LOC342897
cg10409560	0.4558077	-350.469	-0.3099591	0.7657669	FLJ23657
cg14845091	0.3132798	-286.5508	-0.3113842	0.624664	ADPRHL1
cg07525077	0.2624624	-275.9565	-0.3114448	0.5739071	RNASE3
cg26063872	0.3308553	-292.8418	-0.3115488	0.6424041	DEFB123
cg13204181	0.3189655	-290.2223	-0.3122009	0.6311664	GH1
cg01643624	0.4143671	-342.9843	-0.3122579	0.726625	C11orf16
cg21970438	0.445022	-350.469	-0.3126196	0.7576416	TTLL2
cg08314660	0.534674	-350.469	-0.3130814	0.8477555	PKP3
cg10127415	0.213104	-276.7932	-0.3131455	0.5262495	MAGEB6
cg03609102	0.2580696	-278.9435	-0.3131622	0.5712318	MUC5B
cg04711324	0.2517158	-278.721	-0.3133756	0.5650914	RIT2
cg24607398	0.2081466	-277.9554	-0.3136727	0.5218193	MLH1
cg06277657	0.1652984	-283.4637	-0.3142655	0.4795639	DGKI
cg25762706	0.254845	-281.0798	-0.3143768	0.5692218	STMN4
cg06832950	0.4612308	-350.469	-0.3147818	0.7760125	SPG3A
cg16462075	0.4091163	-349.3696	-0.3157056	0.7248219	MUC3B
cg12619162	0.4528832	-350.469	-0.3157181	0.7686013	FXYD4
cg13578652	0.3176605	-302.2734	-0.3174691	0.6351296	UBASH3A
cg01015871	0.2992542	-296.4294	-0.3174876	0.6167418	MT4
cg06220755	0.3001764	-297.7243	-0.3179397	0.618116	RAI2
cg22510822	0.3262143	-306.7373	-0.3180015	0.6442158	OR1E2
cg07126559	0.440896	-350.469	-0.3184139	0.7593099	SGCG

cg19111262	0.2050167	-288.2508	-0.3189644	0.5239811	IGSF9
cg07441143	0.3703793	-332.1194	-0.3192561	0.6896355	SLURP1
cg10062065	0.3389533	-315.6476	-0.3193978	0.6583511	APEG1
cg05348870	0.4496677	-350.469	-0.3197287	0.7693964	TNFSF14
cg13180098	0.3215347	-310.947	-0.3204444	0.641979	RHO
cg23829949	0.260803	-295.0406	-0.3206205	0.5814235	ZNF238
cg15626350	0.1461109	-300.2606	-0.3213511	0.467462	ESR1
cg26149550	0.4670851	-350.469	-0.3214612	0.7885463	KLK15
cg06639544	0.4168135	-350.469	-0.3217018	0.7385153	OR7A5
cg12489960	0.3468472	-326.3868	-0.3220002	0.6688474	SGCB
cg15210427	0.1923254	-295.7992	-0.322507	0.5148324	CST9L
cg16175263	0.2399207	-297.5203	-0.323009	0.5629296	TNFRSF10C
cg10805676	0.3902137	-350.469	-0.3231114	0.7133251	MRPL28
cg19807685	0.3516929	-334.1742	-0.3239182	0.6756111	HSD17B2
cg10758292	0.4455347	-350.469	-0.324324	0.7698587	DEFA1
cg19954000	0.1758692	-304.3867	-0.3261172	0.5019863	FGF1
cg20154346	0.3112076	-322.7963	-0.3267784	0.6379861	RAI2
cg10275770	0.1117552	-322.7963	-0.3282997	0.4400549	ICAM2
cg01774645	0.3540353	-348.3812	-0.3284787	0.682514	ARHGAP30
cg14894144	0.1509117	-312.7948	-0.3285423	0.479454	LAMA3
cg19042947	0.308572	-330.2887	-0.3301314	0.6387034	SERPINA4
cg00756887	0.3535585	-350.469	-0.3319025	0.685461	PVRL4
cg10883352	0.340476	-350.469	-0.3321272	0.6726032	
cg19728223	0.204212	-319.3528	-0.3341666	0.5383787	KCNQ1
cg16626670	0.3115368	-343.3619	-0.3346694	0.6462061	CLEC4G
cg15983538	0.4106378	-350.469	-0.3351912	0.745829	SEMA4A
cg21372914	0.3787588	-350.469	-0.3352694	0.7140282	CLEC4M
cg22264436	0.4114063	-350.469	-0.3354865	0.7468928	SOST
cg16122592	0.3045078	-343.9991	-0.336047	0.6405548	MAGEB6
cg07967308	0.2929082	-340.8091	-0.3365345	0.6294427	ACP5
cg054440	0.5148799	-350.469	-0.336969	0.8518489	FUT6

24					
cg09027725	0.2922337	-342.0867	-0.3371354	0.6293691	COX4I2
cg08403419	0.3097456	-349.7599	-0.3373724	0.647118	RLN3R2
cg00698688	0.4666557	-350.469	-0.3374117	0.8040674	SULT2B1
cg03534410	0.5241687	-350.469	-0.3374942	0.8616629	TMEM40
cg03364781	0.3435501	-350.469	-0.3376121	0.6811622	ALPK1
cg13521229	0.2154481	-327.3213	-0.3376715	0.5531197	JOSD2
cg02130905	0.2690745	-336.2427	-0.3377168	0.6067913	STMN4
cg16772207	0.2808773	-343.8202	-0.3393547	0.620232	MYT1
cg05822532	0.2010833	-330.6027	-0.3394399	0.5405232	ELN
cg12970084	0.338295	-350.469	-0.339561	0.677856	ELF3
cg15928132	0.2675095	-341.572	-0.3400575	0.607567	CCKAR
cg23412777	0.3017922	-350.469	-0.3407299	0.6425221	PYGO1
cg24735489	0.2835258	-348.8251	-0.3409599	0.6244857	CDSN
cg20544605	0.3165931	-350.469	-0.3411081	0.6577012	SORBS2
cg07997737	0.2310349	-336.9835	-0.3412576	0.5722926	NRTN
cg08970446	0.4802257	-350.469	-0.3423631	0.8225887	SLC1A7
cg24670715	0.156878	-340.3347	-0.3427651	0.4996431	ANGPT2
cg21686987	0.4327648	-350.469	-0.3437133	0.7764781	CTRB1
cg01515887	0.3849731	-350.469	-0.3437708	0.7287439	SAA2
cg05766474	0.4744237	-350.469	-0.3440583	0.818482	CCL16
cg14696820	0.256804	-348.8419	-0.3441452	0.6009493	LCE1A
cg14179628	0.2939194	-350.469	-0.3450334	0.6389529	TCEAL7
cg20373326	0.334526	-350.469	-0.3464736	0.6809996	HSD17B2
cg07824742	0.4125586	-350.469	-0.3465125	0.7590712	DBH
cg06101324	0.1475841	-350.469	-0.3478395	0.4954236	SPRR1A
cg25214346	0.3060293	-350.469	-0.3481367	0.654166	NR1I3
cg15516226	0.4489615	-350.469	-0.3491774	0.7981389	BTNL9
cg24024214	0.4715764	-350.469	-0.349973	0.8215494	BTNL8
cg08450982	0.2382757	-350.469	-0.3504617	0.5887374	NUMBL
cg16986846	0.2415437	-350.469	-0.350928	0.5924717	SCGB2A1

cg26292028	0.3369199	-350.469	-0.3530673	0.6899872	FLJ37587
cg16990174	0.1941861	-350.469	-0.353302	0.547488	RYBP
cg03221619	0.1977061	-350.469	-0.3555856	0.5532917	FCER2
cg25221254	0.3693327	-350.469	-0.3571114	0.7264441	ASAH3
cg14182690	0.4327426	-350.469	-0.3581538	0.7908964	RUNX3
cg05485062	0.4176066	-350.469	-0.3592479	0.7768545	SERPINA12
cg24750391	0.1296686	-350.469	-0.3593676	0.4890362	PON3
cg03602500	0.370848	-350.469	-0.3598494	0.7306974	FLJ00060
cg12815142	0.3144491	-350.469	-0.3616767	0.6761258	SPAG7
cg21948655	0.4369155	-350.469	-0.3619581	0.7988736	SMCP
cg15652212	0.3310183	-350.469	-0.3623571	0.6933754	FLJ90586
cg20334738	0.2836401	-350.469	-0.3652416	0.6488818	MAB21L2
cg08684473	0.3831029	-350.469	-0.3652804	0.7483833	LILRB5
cg24694549	0.2743033	-350.469	-0.3659974	0.6403007	GRIP1
cg10990993	0.2324561	-350.469	-0.3660769	0.5985331	MLH1
cg12513481	0.1929905	-350.469	-0.367272	0.5602626	SCAP1
cg14992108	0.3510814	-350.469	-0.3691767	0.7202581	SNTB1
cg01103730	0.3278863	-350.469	-0.369484	0.6973703	IL20
cg26672426	0.2563614	-350.469	-0.3698636	0.626225	PTGES
cg01335367	0.1497868	-350.469	-0.3735641	0.5233509	C12orf34
cg23815000	0.3526621	-350.469	-0.3745332	0.7271953	LCN1
cg08573687	0.3061677	-350.469	-0.3745495	0.6807172	TH
cg06123346	0.2424465	-350.469	-0.3775486	0.6199951	ATP4A
cg06501790	0.2863328	-350.469	-0.3817138	0.6680466	SLC34A1
cg18780284	0.2982884	-350.469	-0.3831052	0.6813936	SPRR1B
cg25119415	0.3001483	-350.469	-0.3877927	0.687941	MNDA
cg27619475	0.1684046	-350.469	-0.3880815	0.5564861	SLC16A5
cg17907567	0.2335434	-350.469	-0.3882501	0.6217936	HAMP
cg21457804	0.3606782	-350.469	-0.3898364	0.7505146	CT45-2
cg18149207	0.3345924	-350.469	-0.3987587	0.7333511	RORC
cg142047	0.06397704	-350.469	-0.4021686	0.4661456	CYB561

35					
cg06536578	0.2420131	-350.469	-0.4081324	0.6501456	JPH4
cg22374142	0.08937864	-350.469	-0.4082978	0.4976765	HSF4
cg25856811	0.3027658	-350.469	-0.4151909	0.7179568	SPRR3
cg04144768	0.2637959	-350.469	-0.415307	0.6791029	DDC
cg26927807	0.2687147	-350.469	-0.4249378	0.6936526	BTBD2
cg13726507	0.4021371	-350.469	-0.4262811	0.8284182	CTAG2
cg14256699	0.4281882	-350.469	-0.4322005	0.8603887	SOST
cg26164184	0.3667875	-350.469	-0.4481277	0.8149152	FCN2
cg13021192	0.09652124	-350.469	-0.5039948	0.6005161	CTSZ
cg16179125	0.1331095	-350.469	-0.5100906	0.6432001	CTSZ

Suppelementary table 13. Probes differentially methylated between *RET*-mutated tumours and normal tissue

TargetID	RET.AVG_Beta	RET.DiffScore	RET.Delta Beta	normal.AVG_Beta	SYMBOL
cg16363586	0.6141389	339.4807	0.3025294	0.3116095	BST2
cg06204948	0.5180554	339.4807	0.2917404	0.226315	MARK2
cg14409083	0.5276059	339.4807	0.2890297	0.2385762	EMP1
cg01126560	0.6742277	339.4807	0.2764263	0.3978014	C9orf142
cg03852144	0.5250785	339.4807	0.2588267	0.2662518	GLRX
cg01346152	0.5000116	339.4807	0.2490346	0.2509769	DHRS3
cg26394940	0.364035	339.4807	0.245714	0.118321	FLJ10945
cg16517394	0.4461616	339.4807	0.2414309	0.2047307	TNFSF4
cg09871315	0.5443512	126.9372	0.2389772	0.305374	HOXA2
cg24101578	0.5739437	118.2729	0.2342299	0.3397138	CDH22
cg10861599	0.5106688	119.1197	0.2286621	0.2820067	TNFSF4
cg08624249	0.7055397	112.1707	0.2265095	0.4790302	KIAA0889
cg17998964	0.3882163	339.4807	0.224425	0.1637913	MARK2
cg03605761	0.5792937	104.8027	0.2221926	0.357101	RNF126
cg22628873	0.6713313	101.805	0.2185028	0.4528286	GGT6
cg25101936	0.7188479	105.7451	0.2182697	0.5005782	ZBTB16
cg07251788	0.7014782	100.0918	0.214135	0.4873433	CLTCL1
cg22467567	0.4923321	104.6278	0.2139002	0.2784319	IGFBP5
cg06507244	0.6597449	96.0965	0.2132643	0.4464805	DHX32
cg06627364	0.3906854	121.2099	0.2076089	0.1830765	MGC4677
cg16616769	0.741579	93.63908	0.2015827	0.5399963	MGC35048
cg12564453	0.7337025	91.16164	0.2000533	0.5336493	CETP
cg01103836	0.874963	130.6221	0.2000048	0.6749582	MYO9B
cg23580945	0.5497846	-105.9985	-0.2000843	0.7498689	FLJ43826
cg03364781	0.4810647	-91.97941	-0.2000975	0.6811622	ALPK1

cg18538812	0.2416636	-88.80154	-0.2001664	0.44183	GIF
cg07471052	0.7057543	-201.2404	-0.2001731	0.9059274	CDK3
cg24919884	0.4577272	-89.05791	-0.2002409	0.657968	ARHGEF16
cg19717326	0.246828	-88.54828	-0.2005255	0.4473534	MYADM
cg25400358	0.3620302	-83.68384	-0.2007748	0.5628049	GPR137
cg23278885	0.5042692	-96.64542	-0.2008188	0.7050881	TGM6
cg18063149	0.4892721	-94.21997	-0.2009886	0.6902608	FMO3
cg07664027	0.5795134	-116.8238	-0.2010418	0.7805552	RPL13A
cg01663968	0.1423713	-108.4788	-0.2011463	0.3435176	CTSZ
cg21489722	0.5607121	-110.5895	-0.2011536	0.7618657	FAM9B
cg26473272	0.666001	-165.1459	-0.2013879	0.867389	SYT8
cg07792737	0.6109162	-130.2685	-0.2014001	0.8123163	NPIP
cg10052840	0.2936386	-85.70172	-0.2014864	0.4951251	SEMA6B
cg27418851	0.4576738	-90.37128	-0.2015366	0.6592104	MBL2
cg15590780	0.2954544	-85.92134	-0.2018858	0.4973402	USH2A
cg00689010	0.4524277	-90.15679	-0.201895	0.6543227	NCSTN
cg25072962	0.423374	-87.48565	-0.2019602	0.6253342	MGC35295
cg26583078	0.4591237	-91.0274	-0.2019804	0.6611041	SORBS2
cg18242139	0.5114066	-99.49696	-0.2020041	0.7134107	ELAVL4
cg04337944	0.2766576	-87.19349	-0.2020414	0.4786991	FBLN1
cg08684473	0.5462096	-107.7361	-0.2021737	0.7483833	LILRB5
cg13447818	0.5676587	-114.4472	-0.2022987	0.7699574	FLG
cg12949760	0.4922299	-96.17561	-0.202301	0.694531	KCNQ1
cg01568736	0.5373582	-105.629	-0.2023147	0.7396729	SERPINB7
cg02130905	0.4043903	-86.6141	-0.202401	0.6067913	STMN4
cg06244906	0.5652246	-113.9072	-0.2024825	0.7677072	ZIM2
cg16462075	0.5223384	-102.3908	-0.2024835	0.7248219	MUC3B
cg08495878	0.6619939	-164.5056	-0.2025579	0.8645517	SERPINA4
cg05985767	0.4669501	-92.83908	-0.2027707	0.6697208	ANPEP
cg25827666	0.533052	-105.2268	-0.2028347	0.7358867	NTRK1
cg06437862	0.5570782	-112.0406	-0.2030523	0.7601305	TUBA2
cg09715672	0.2635104	-89.12792	-0.2030889	0.4665993	C10orf116
cg06531741	0.3324736	-85.97534	-0.2033509	0.5358245	HTR3B
cg18979223	0.457029	-92.41476	-0.2035723	0.6606013	CDKN2B
cg18056600	0.1613224	-105.7451	-0.2036178	0.3649402	ZMYND15
cg11762346	0.4991357	-99.03928	-0.2036844	0.7028201	HKDC1
cg06275635	0.3549532	-86.38834	-0.2038894	0.5588427	PGLYRP3
cg19824441	0.4265645	-89.78389	-0.2040459	0.6306103	ADMR
cg15869022	0.3553292	-86.60481	-0.204138	0.5594673	GPR17
cg04488758	0.457402	-93.0736	-0.2041658	0.6615677	USP44
cg07525077	0.3697257	-86.87199	-0.2041815	0.5739071	RNASE3
cg19447966	0.5481178	-111.0378	-0.2042878	0.7524056	TEAD1
cg08158289	0.1715601	-104.125	-0.2042897	0.3758498	KIAA0141
cg04574507	0.4445444	-91.82198	-0.204382	0.6489264	CD1B
cg00152644	0.6168387	-138.4765	-0.2043875	0.8212262	SPRR2E

cg07484827	0.2962995	-88.10714	-0.2044543	0.5007538	CHRNA10
cg14911395	0.2664446	-90.10414	-0.2044643	0.4709089	SEMA3B
cg18988110	0.2558024	-91.13265	-0.2045401	0.4603425	ATAD4
cg20311730	0.423498	-90.04996	-0.2045763	0.6280743	NALP10
cg04567009	0.3872435	-88.10714	-0.2048647	0.5921081	FCGR3B
cg08555657	0.5400344	-109.6176	-0.2048815	0.7449159	SPRR2E
cg24607398	0.3169318	-87.64392	-0.2048875	0.5218193	MLH1
cg09343150	0.5697731	-119.2797	-0.2051831	0.7749562	MEN1
cg27020690	0.1478989	-110.7998	-0.205295	0.3531938	TERC
cg19000186	0.4633534	-95.06622	-0.2053126	0.668666	CNGA1
cg02813121	0.6211852	-142.5424	-0.2053381	0.8265232	S100A12
cg10677144	0.505901	-102.3446	-0.2053467	0.7112477	MYOM1
cg27394486	0.5954238	-129.7641	-0.2053975	0.8008213	C15orf2
cg07123548	0.6116178	-137.6178	-0.2054454	0.8170632	HIPK4
cg10127415	0.3207231	-88.1302	-0.2055264	0.5262495	MAGEB6
cg13439299	0.6191036	-142.0158	-0.2056478	0.8247514	DNAJC5G
cg01227741	0.5342569	-109.37	-0.2058856	0.7401425	GIMAP7
cg06325687	0.6964693	-206.3535	-0.2060013	0.9024706	OPN1MW
cg24660086	0.4630298	-95.81652	-0.2060328	0.6690627	RGR
cg10071275	0.3744169	-88.80154	-0.2061237	0.5805405	MYT1
cg08244028	0.4468985	-93.93452	-0.2061577	0.6530562	MSH3
cg15842430	0.421692	-91.46799	-0.2061661	0.627858	FAM12B
cg20903926	0.5451965	-112.7819	-0.2062033	0.7513998	C1orf177
cg07643942	0.3558691	-88.54828	-0.206256	0.5621251	LACRT
cg18780284	0.4749406	-98.00538	-0.206453	0.6813936	SPRR1B
cg02423618	0.3850442	-89.51582	-0.2064723	0.5915165	SPATA8
cg12619162	0.5621089	-118.5573	-0.2064924	0.7686013	FXYD4
cg16742703	0.4239115	-92.02892	-0.2065478	0.6304594	KLK3
cg21065959	0.4522318	-94.96701	-0.2065486	0.6587805	LCE1E
cg14186992	0.4517737	-95.02003	-0.2066544	0.6584281	HKR3
cg10321723	0.6625345	-174.0647	-0.2066876	0.8692221	PDZK1
cg24490338	0.5131286	-105.4175	-0.2067677	0.7198963	TPM3
cg04048249	0.4508872	-95.19501	-0.2069212	0.6578084	APOC3
cg01367992	0.3825469	-89.99275	-0.2070789	0.5896258	LY9
cg18533225	0.4821472	-100.0005	-0.2071086	0.6892558	KLHDC7B
cg04273431	0.1818621	-104.5292	-0.2072062	0.3890684	PRR3
cg17240454	0.5923019	-131.905	-0.2076595	0.7999614	SPDEF
cg25762706	0.3612584	-90.19344	-0.2079634	0.5692218	STMN4
cg13859324	0.2923816	-91.50857	-0.2080734	0.500455	UNC45B
cg03782453	0.176195	-106.5144	-0.2081879	0.3843829	FLJ90575
cg04727522	0.4571961	-97.42852	-0.2082433	0.6654394	C18orf22
cg03752885	0.3168474	-90.71026	-0.2083007	0.5251481	DAPK3
cg21808053	0.3171731	-90.83755	-0.2084454	0.5256186	DIRAS3
cg16242770	0.5534247	-118.9566	-0.2087961	0.7622209	KRTAP17-1
cg09414535	0.3948025	-92.31754	-0.2088957	0.6036983	GRIP1

cg22937320	0.4985354	-105.057	-0.2089927	0.7075281	C9orf138
cg07115820	0.3679951	-91.34138	-0.2090105	0.5770056	EPX
cg09299388	0.4880766	-103.2257	-0.2090108	0.6970874	PGK2
cg06066303	0.5977945	-137.0334	-0.2091026	0.8068971	MS4A5
cg09027725	0.4202377	-94.33099	-0.2091314	0.6293691	COX4I2
cg09931793	0.4820139	-102.3482	-0.2091851	0.6911989	OR2K2
cg04655481	0.4926478	-104.4146	-0.2093454	0.7019932	GPR21
cg24693053	0.261044	-94.94707	-0.2093588	0.4704029	MFSD7
cg22253945	0.5743541	-127.5209	-0.2094603	0.7838143	GPR45
cg06303238	0.442548	-97.18629	-0.2096855	0.6522335	SALL4
cg13259290	0.5159144	-109.7468	-0.2097695	0.7256839	CSF2
cg04132607	0.6492401	-170.5615	-0.2098842	0.8591244	GATA5
cg03931808	0.6770014	-195.7476	-0.2100338	0.8870353	RLN3
cg24352688	0.4077036	-94.28899	-0.2100605	0.6177641	OFD1
cg20416179	0.580469	-130.8422	-0.2101561	0.7906252	C6orf71
cg24429836	0.249203	-97.31154	-0.210659	0.459862	LDHD
cg23829949	0.3707085	-93.04058	-0.2107151	0.5814235	ZNF238
cg04450876	0.5737673	-129.2964	-0.2108253	0.7845926	FAM112B
cg00226923	0.552474	-121.8546	-0.2110401	0.7635141	FGD2
cg11739626	0.4893183	-105.6766	-0.2110421	0.7003604	AKT1S1
cg27117399	0.3835252	-94.06508	-0.2112547	0.5947799	CNDP1
cg10746737	0.4938364	-106.8577	-0.2113465	0.7051829	HLA-DRB5
cg18089000	0.4875986	-105.7451	-0.2113828	0.6989814	GBGT1
cg24355048	0.4259863	-97.29191	-0.2114542	0.6374406	CTSG
cg08815403	0.3631919	-93.58691	-0.2114922	0.5746841	HSD17B13
cg13271951	0.313558	-93.93452	-0.2116865	0.5252445	FAM57B
cg01869233	0.2010719	-104.9931	-0.2117176	0.4127895	C20orf75
cg19859270	0.4711509	-103.5207	-0.2117947	0.6829456	GPR15
cg05564657	0.6683458	-191.8242	-0.2118482	0.880194	AADAC
cg10853416	0.4947785	-107.6789	-0.2118525	0.7066309	MS4A7
cg18396533	0.2068432	-104.4146	-0.2120899	0.4189331	DYDC1
cg12069042	0.4667999	-103.2881	-0.2121857	0.6789856	PLXNB1
cg01726767	0.4062356	-96.45499	-0.2122865	0.6185221	LALBA
cg02989940	0.3620366	-94.36263	-0.2123416	0.5743782	ERAF
cg20104776	0.3538597	-94.51772	-0.212656	0.5665156	LDOC1
cg18239253	0.3462056	-94.70631	-0.2129264	0.559132	DEFB32
cg08314660	0.6346279	-166.4672	-0.2131276	0.8477555	PKP3
cg10322876	0.4102709	-97.70769	-0.2132102	0.6234811	CYP2B6
cg00718513	0.2719921	-97.66402	-0.2133126	0.4853047	
cg23382741	0.4557819	-103.0597	-0.2134281	0.6692101	PPP1R14D
cg26884581	0.183208	-110.6714	-0.2141746	0.3973826	PYGM
cg14127336	0.6558729	-185.94	-0.2141938	0.8700668	TCL1A
cg21624282	0.5838842	-139.2989	-0.2143524	0.7982366	LOC122258
cg09546307	0.4599354	-104.7249	-0.2144611	0.6743965	CLEC4D
cg01678091	0.5364901	-122.1554	-0.2148352	0.7513254	MAGEL2

cg20781967	0.2214122	-104.7893	-0.2149894	0.4364015	NINJ2
cg01053621	0.3529452	-96.83838	-0.2150365	0.5679817	APOA2
cg20856834	0.4175011	-100.5474	-0.2152185	0.6327196	OR12D3
cg19006008	0.4219073	-101.0481	-0.2152855	0.6371928	F2RL3
cg03044435	0.5498787	-127.629	-0.2155575	0.7654362	FLJ35816
cg12351042	0.4324412	-102.4639	-0.215601	0.6480422	OR2B2
cg02601403	0.5533972	-129.4429	-0.2159808	0.7693781	TBC1D3C
cg04806409	0.5176749	-119.0278	-0.2165038	0.7341787	TFF3
cg00698688	0.5872909	-145.0024	-0.2167765	0.8040674	SULT2B1
cg21639401	0.4470793	-105.5439	-0.2168614	0.6639407	FLJ31222
cg12593411	0.4120773	-101.8533	-0.2169017	0.6289791	ANGPTL6
cg08341924	0.5414116	-126.7753	-0.2169147	0.7583263	TGM1
cg10210238	0.2376852	-104.4592	-0.2169234	0.4546087	CDKN2B
cg07947016	0.2649397	-101.7714	-0.2169529	0.4818926	KLK2
cg18129786	0.5928427	-148.3588	-0.2170244	0.8098671	ZNF445
cg00644033	0.7005286	-244.9592	-0.2170378	0.9175664	MUC3B
cg14894144	0.2623193	-102.1422	-0.2171347	0.479454	LAMA3
cg15210427	0.2976436	-99.82443	-0.2171888	0.5148324	CST9L
cg22190114	0.4980483	-115.2747	-0.2173331	0.7153814	NALP8
cg24851490	0.2862798	-101.179	-0.217967	0.5042468	RNASE2
cg10575735	0.3827475	-100.9784	-0.2180364	0.6007839	SSX4
cg24619694	0.447648	-107.0537	-0.2181878	0.6658359	TAGLN
cg15518950	0.2540873	-103.9482	-0.2182274	0.4723147	PRP2
cg02218324	0.4316766	-105.2268	-0.2183048	0.6499814	RSHL1
cg01772980	0.405505	-102.9425	-0.2184658	0.6239709	SCGB1D1
cg03609102	0.3527656	-100.3049	-0.2184663	0.5712318	MUC5B
cg11158374	0.4864124	-114.4175	-0.2186574	0.7050698	TFF2
cg04962134	0.3813272	-101.6122	-0.2187138	0.600041	TRIM51
cg16899306	0.4029246	-103.0943	-0.2188101	0.6217347	HLA-DQB2
cg15075718	0.4114881	-103.9482	-0.2189346	0.6304227	MFRP
cg19910382	0.3766419	-101.6314	-0.2189669	0.5956088	FABP1
cg16222568	0.4907345	-115.7732	-0.2189865	0.709721	APEG1
cg06811800	0.4563305	-109.3234	-0.2189941	0.6753246	ATP4B
cg18971671	0.4751367	-112.5795	-0.2190324	0.6941691	TULP2
cg27090216	0.1387395	-127.5213	-0.2193509	0.3580904	TNFRSF10C
cg01074640	0.4275968	-105.8834	-0.2193903	0.6469871	IFNA17
cg23881725	0.2100529	-110.8664	-0.2196309	0.4296838	DLEC1
cg24824840	0.5703396	-142.2863	-0.2197694	0.790109	SHANK1
cg02324920	0.3052629	-102.1352	-0.219911	0.5251739	NEURL
cg24750391	0.268831	-104.4384	-0.2202052	0.4890362	PON3
cg00626119	0.3165711	-102.1118	-0.2202154	0.5367865	NTRK1
cg21432842	0.3177736	-102.3543	-0.2205137	0.5382873	CSF3
cg05248781	0.6203389	-172.7047	-0.2209423	0.8412812	LCE5A
cg02844051	0.5150685	-124.7876	-0.2211661	0.7362346	ZD52F10
cg08093398	0.4306025	-108.4788	-0.2213659	0.6519684	PSF1

cg27442349	0.411771	-106.468	-0.2214336	0.6332046	NFKBIB
cg15060813	0.2879737	-104.3831	-0.2214536	0.5094273	LRFN3
cg22194129	0.3675266	-103.9482	-0.2216718	0.5891984	CLEC4C
cg13694749	0.4627754	-113.7373	-0.2217819	0.6845574	SCN4A
cg19712821	0.3450021	-103.5207	-0.2217964	0.5667985	KSP37
cg14662756	0.4219618	-109.0515	-0.222776	0.6447378	NPFF
cg04457051	0.3820712	-105.7451	-0.2228415	0.6049128	SCOC
cg01837574	0.312162	-104.806	-0.222995	0.535157	TRAPPC1
cg08603768	0.4328246	-110.8664	-0.2232571	0.6560817	WNT8A
cg10990993	0.3747467	-106.3076	-0.2237864	0.5985331	MLH1
cg12943082	0.520501	-130.0436	-0.2238636	0.7443646	CCL26
cg25994725	0.3381523	-105.4156	-0.2238742	0.5620265	C6orf81
cg10569414	0.4655543	-116.9046	-0.2239256	0.6894798	C21orf121
cg17264470	0.6302181	-187.2439	-0.2241341	0.8543522	FGF21
cg24694549	0.4157196	-110.3805	-0.2245811	0.6403007	GRIP1
cg10213812	0.6384286	-195.2163	-0.2245826	0.8630112	FOXN1
cg03003745	0.3651375	-107.0537	-0.2249396	0.590077	UNQ473
cg15743985	0.4795564	-121.1006	-0.2250376	0.704594	CD22
cg08292050	0.5328642	-135.9419	-0.2250853	0.7579495	SOCS4
cg21686987	0.5510184	-143.3158	-0.2254597	0.7764781	CTRB1
cg24825722	0.370164	-107.9484	-0.2255535	0.5957175	ACADVL
cg08475088	0.6229246	-184.8049	-0.2255741	0.8484987	NALP9
cg17706173	0.2115145	-117.8896	-0.2269055	0.43842	C16orf30
cg01718139	0.5387414	-141.1574	-0.2270525	0.7657939	UNQ3033
cg12061127	0.4414831	-116.9312	-0.2272817	0.6687647	WFDC9
cg01657380	0.4423485	-117.3541	-0.2275237	0.6698722	NPFF
cg04353769	0.3860098	-111.2491	-0.2277761	0.6137858	MS4A6A
cg04810997	0.4436172	-117.9047	-0.2278185	0.6714357	TAS2R60
cg20516209	0.4050753	-113.231	-0.2280563	0.6331316	EMILIN1
cg11113534	0.6278597	-194.5688	-0.2281136	0.8559733	C20orf70
cg03552103	0.3424432	-110.0193	-0.2283302	0.5707734	09-sep
cg03343942	0.3084583	-110.2174	-0.2283468	0.5368052	SLC39A5
cg19561774	0.6307744	-197.6814	-0.2284714	0.8592458	SLC22A2
cg15741706	0.4665893	-122.8526	-0.2284728	0.6950621	CXorf48
cg15648315	0.4892918	-128.0956	-0.2285773	0.717869	FLJ26443
cg07595943	0.6881645	-264.5584	-0.228628	0.9167925	LOC161931
cg25033144	0.5031922	-132.8832	-0.229444	0.7326362	FLJ00060
cg11015241	0.6312377	-201.2694	-0.229777	0.8610147	ATP10A
cg25013053	0.4673476	-124.8361	-0.2298452	0.6971928	UNC45B
cg20512303	0.3909269	-114.5527	-0.2303395	0.6212664	PDLIM4
cg05755354	0.32849	-112.0398	-0.2303857	0.5588757	FRMD4A
cg23412777	0.4120054	-116.8761	-0.2305167	0.6425221	PYGO1
cg01668126	0.4563234	-123.7503	-0.2307048	0.6870282	MSR1
cg06539449	0.5082976	-136.785	-0.2310016	0.7392992	CCND1
cg22784047	0.4555395	-124.0675	-0.2310584	0.6865979	MVP

cg15542496	0.4970473	-133.6823	-0.2312081	0.7282554	PIP
cg27257987	0.4155802	-118.3483	-0.2314407	0.6470209	PSG4
cg27087809	0.5206755	-141.5645	-0.2314756	0.7521511	ACSBG1
cg26777475	0.366119	-114.4613	-0.2317276	0.5978466	PCOLCE
cg16626670	0.4142594	-118.7855	-0.2319468	0.6462061	CLEC4G
cg25957124	0.2950555	-114.6432	-0.2321633	0.5272189	DNAH3
cg12970081	0.3434562	-114.2474	-0.232242	0.5756982	GPR32
cg14238120	0.4804035	-130.8422	-0.2322963	0.7126997	ELA3A
cg05556717	0.3666343	-115.1599	-0.2323158	0.5989501	CCL26
cg10275770	0.2071534	-124.8069	-0.2329015	0.4400549	ICAM2
cg01515887	0.495783	-135.9419	-0.2329609	0.7287439	SAA2
cg14256699	0.6267931	-206.4446	-0.2335956	0.8603887	SOST
cg26705561	0.3087689	-115.8292	-0.2336895	0.5424584	SEC31L2
cg05671018	0.4453302	-126.0472	-0.2340518	0.679382	LYSMD2
cg23413307	0.410001	-120.9256	-0.2341447	0.6441457	LCE1F
cg16377880	0.3630417	-117.2756	-0.2343704	0.5974121	CYP4F3
cg26415633	0.2331165	-122.4237	-0.2344994	0.4676159	KLK1
cg12022621	0.3160466	-116.8238	-0.2346926	0.5507392	LAX1
cg07824742	0.5242762	-148.0895	-0.234795	0.7590712	DBH
cg00594952	0.3301486	-116.8704	-0.2348033	0.564952	RIMS3
cg26264314	0.5142482	-145.0014	-0.2351853	0.7494335	NALP5
cg06351503	0.4511709	-128.8737	-0.2354574	0.6866283	RDBP
cg10190509	0.5384123	-155.6749	-0.2359114	0.7743237	CCL16
cg01015871	0.3806767	-120.2864	-0.2360651	0.6167418	MT4
cg25119415	0.4515362	-130.0893	-0.2364048	0.687941	MNDA
cg05348870	0.5320689	-155.449	-0.2373275	0.7693964	TNFSF14
cg26353877	0.5160167	-149.4981	-0.2375612	0.7535778	APCS
cg06436504	0.4320759	-128.2192	-0.2375648	0.6696407	DOC1
cg00334507	0.3865867	-122.7392	-0.2377162	0.6243029	MVP
cg16175263	0.3250998	-120.0983	-0.2378298	0.5629296	TNFRSF10C
cg18508525	0.5737905	-177.3036	-0.237962	0.8117525	CD36
cg04143809	0.3333475	-120.9256	-0.2385047	0.5718522	FLJ39822
cg02351381	0.225666	-127.6959	-0.2385285	0.4641945	C12orf34
cg11825652	0.3430074	-121.1367	-0.2385412	0.5815486	CAV2
cg03557698	0.6306412	-222.819	-0.2385713	0.8692125	C1orf177
cg10062065	0.4197063	-127.6959	-0.2386449	0.6583511	APEG1
cg12334759	0.4592131	-134.9015	-0.238757	0.6979702	C19orf19
cg19047670	0.4298203	-129.458	-0.2388453	0.6686655	CCND1
cg24541550	0.3345642	-121.2937	-0.2388599	0.5734241	MRVI1
cg19304352	0.553628	-168.0886	-0.2389381	0.7925661	DEFA4
cg15422147	0.4489853	-133.6743	-0.2394682	0.6884536	SERPINB5
cg10414946	0.3912926	-125.5634	-0.2396667	0.6309593	MS4A2
cg12040555	0.6527676	-251.0325	-0.2397373	0.8925049	MGMT
cg16964535	0.6697609	-273.901	-0.2397531	0.9095141	DNAJC5G
cg12815142	0.4361942	-131.7939	-0.2399316	0.6761258	SPAG7

cg09467501	0.1313804	-152.9773	-0.2407327	0.3721131	PYY
cg23260026	0.2655411	-125.6736	-0.2407849	0.506326	FSTL3
cg25372195	0.4171788	-130.0893	-0.2409036	0.6580823	DCD
cg16772207	0.3790722	-126.2265	-0.2411598	0.620232	MYT1
cg27235662	0.5157973	-155.6576	-0.2413331	0.7571304	CLDN16
cg09448880	0.3975486	-128.2686	-0.2414248	0.6389734	PGLYRP3
cg04505023	0.4580215	-138.7678	-0.2417214	0.6997429	SPRR1A
cg21457804	0.5082275	-154.4301	-0.2422871	0.7505146	CT45-2
cg08634024	0.5760449	-187.8101	-0.2424225	0.8184674	OR2F1
cg17907567	0.3791381	-127.9771	-0.2426554	0.6217936	HAMP
cg20551517	0.5167314	-158.2428	-0.2426983	0.7594296	GIP
cg08448751	0.4183195	-133.0514	-0.2431228	0.6614423	SEMA3G
cg07126559	0.5157496	-159.3563	-0.2435603	0.7593099	SGCG
cg10805676	0.4696054	-144.4022	-0.2437196	0.7133251	MRPL28
cg15928132	0.3632918	-128.698	-0.2442752	0.607567	CCKAR
cg19917856	0.268276	-129.4705	-0.2445798	0.5128558	LOC342897
cg12582965	0.6133359	-223.8487	-0.2455336	0.8588696	ATP10A
cg11380128	0.4875372	-153.3238	-0.2460172	0.7335544	PRLH
cg06501790	0.4219672	-137.6178	-0.2460794	0.6680466	SLC34A1
cg01550148	0.3011	-129.777	-0.2463311	0.5474311	H2AFY
cg22901840	0.4934376	-156.0337	-0.2465378	0.7399755	DIRAS3
cg24901474	0.3049259	-130.0893	-0.2466944	0.5516204	RGS5
cg17788013	0.4506744	-144.2937	-0.2468404	0.6975147	SPINK5
cg23776892	0.5296398	-171.3389	-0.2470328	0.7766726	MAGEA1
cg03221619	0.305986	-130.718	-0.2473058	0.5532917	FCER2
cg05215575	0.3175384	-130.8422	-0.2475054	0.5650438	FLJ25410
cg09701102	0.5053974	-162.3105	-0.2477245	0.7531219	NDUFV1
cg24670715	0.251518	-134.8984	-0.248125	0.4996431	ANGPT2
cg13204181	0.3827667	-135.339	-0.2483997	0.6311664	GH1
cg05444024	0.6032271	-222.7355	-0.2486218	0.8518489	FUT6
cg15905124	0.5224634	-171.1919	-0.2487279	0.7711913	MGC13034
cg14179628	0.3898602	-137.0334	-0.2490926	0.6389529	TCEAL7
cg23444894	0.4934781	-160.5821	-0.2493123	0.7427903	UNQ5810
cg00344709	0.5260019	-173.9728	-0.249437	0.7754389	ANKRD21
cg17687962	0.4870034	-159.9724	-0.2502748	0.7372782	KLK3
cg16794682	0.5029283	-166.0949	-0.2505174	0.7534457	CCND1
cg19982860	0.3783564	-138.3831	-0.2511192	0.6294757	IFNA21
cg06101324	0.2442887	-139.0736	-0.2511348	0.4954236	SPRR1A
cg10894512	0.3823261	-139.4167	-0.2516526	0.6339787	ACTA2
cg19728223	0.2859054	-137.4076	-0.2524733	0.5383787	KCNQ1
cg06123346	0.3672938	-139.3087	-0.2527013	0.6199951	ATP4A
cg25214346	0.4013285	-143.3661	-0.2528375	0.654166	NR1I3
cg19368582	0.3940186	-142.6183	-0.2530137	0.6470323	MMRN2
cg05670596	0.2345363	-142.6471	-0.2532799	0.4878161	CCRL2
cg22374142	0.2442381	-141.7802	-0.2534384	0.4976765	HSF4

cg07997737	0.3185152	-138.3831	-0.2537774	0.5722926	NRTN
cg04744379	0.4895775	-167.2278	-0.254184	0.7437615	KLK15
cg26063872	0.387983	-143.6726	-0.2544212	0.6424041	DEFB123
cg19403023	0.2965997	-139.8691	-0.254903	0.5515027	TESSP1
cg14333565	0.536292	-190.1143	-0.2551746	0.7914666	NRTN
cg21948655	0.5430785	-195.2163	-0.2557951	0.7988736	SMCP
cg24992780	0.5419066	-196.4265	-0.2567647	0.7986712	OR7C1
cg20544605	0.4009073	-148.8268	-0.2567939	0.6577012	SORBS2
cg15780361	0.2945248	-142.3265	-0.2568659	0.5513908	ALS2CR11
cg16990174	0.2900146	-143.1223	-0.2574735	0.547488	RYBP
cg13578652	0.3769964	-147.4308	-0.2581331	0.6351296	UBASH3A
cg01643624	0.4684879	-166.5739	-0.2581371	0.726625	C11orf16
cg12513481	0.3006208	-145.5079	-0.2596418	0.5602626	SCAP1
cg19954000	0.2421568	-149.4981	-0.2598295	0.5019863	FGF1
cg18149207	0.4728476	-171.9121	-0.2605035	0.7333511	RORC
cg10758292	0.5091185	-187.1207	-0.2607402	0.7698587	DEFA1
cg14845091	0.3638223	-149.6352	-0.2608417	0.624664	ADPRHL1
cg04323365	0.4827223	-177.1717	-0.2614417	0.744164	GJB1
cg10604646	0.2750632	-149.4981	-0.2620851	0.5371482	RGS5
cg06536578	0.3878182	-154.6989	-0.2623273	0.6501456	JPH4
cg15652212	0.4309971	-162.9203	-0.2623783	0.6933754	FLJ90586
cg08573687	0.4181424	-160.3895	-0.2625748	0.6807172	TH
cg09426307	0.3848561	-154.6791	-0.2625929	0.647449	SEC14L3
cg14173523	0.3980212	-156.6852	-0.2626538	0.660675	FUT5
cg03602500	0.4679465	-174.0647	-0.2627509	0.7306974	FLJ00060
cg04816348	0.6031593	-261.4301	-0.2631083	0.8662676	CLEC4G
cg04144768	0.4159607	-160.7468	-0.2631422	0.6791029	DDC
cg21372914	0.4495877	-171.1919	-0.2644405	0.7140282	CLEC4M
cg22264436	0.4821696	-182.7407	-0.2647232	0.7468928	SOST
cg14366490	0.3721351	-156.8844	-0.2654231	0.6375582	TXNL6
cg17829936	0.4199609	-165.2	-0.2654809	0.6854419	TAAR5
cg14706739	0.4266823	-167.2555	-0.2658937	0.6925761	EPB49
cg01894895	0.5117868	-199.5271	-0.2665166	0.7783033	ANXA1
cg19728577	0.5061343	-197.227	-0.2668219	0.7729563	GUCA2B
cg23815000	0.4602005	-178.5933	-0.2669947	0.7271953	LCN1
cg19807685	0.4081664	-165.6518	-0.2674447	0.6756111	HSD17B2
cg16122592	0.3730304	-160.0336	-0.2675244	0.6405548	MAGEB6
cg07597976	0.317753	-155.8468	-0.2675413	0.5852943	CD19
cg05556202	0.489859	-192.2386	-0.2681602	0.7580193	TM4SF19
cg06233503	0.2957712	-156.6095	-0.2682343	0.5640055	KCNQ1
cg26292028	0.4205188	-171.465	-0.2694685	0.6899872	FLJ37587
cg00321478	0.3866285	-165.3831	-0.2698684	0.6564969	CRB1
cg02067021	0.4186682	-171.7041	-0.2699116	0.6885798	DNAJC5B
cg01827098	0.5243971	-215.362	-0.2707103	0.7951075	GIMAP7
cg08626653	0.4400897	-178.8424	-0.2709099	0.7109996	FLJ37538

cg01335367	0.2521847	-162.5771	-0.2711662	0.5233509	C12orf34
cg13521229	0.2817253	-161.0258	-0.2713943	0.5531197	JOSD2
cg26927807	0.4221363	-175.0013	-0.2715163	0.6936526	BTBD2
cg27619475	0.2848877	-161.17	-0.2715984	0.5564861	SLC16A5
cg08453096	0.5537253	-239.3138	-0.2721671	0.8258924	ABCG5
cg01119135	0.6062474	-292.7625	-0.2724242	0.8786716	C1orf116
cg11984608	0.5237565	-221.0501	-0.2734929	0.7972494	CLDN16
cg25813714	0.5683712	-259.9207	-0.2752225	0.8435937	CYP4F12
cg27285599	0.4862854	-204.3294	-0.275295	0.7615805	FLJ13841
cg08450982	0.3133986	-166.1987	-0.2753389	0.5887374	NUMBL
cg20334738	0.3731896	-171.8727	-0.2756922	0.6488818	MAB21L2
cg25781162	0.4778703	-202.0568	-0.2761481	0.7540183	ABCG5
cg17977362	0.4815962	-204.8697	-0.2767027	0.7582989	FLJ45964
cg06236276	0.4803484	-206.3068	-0.2777543	0.7581028	SLC22A2
cg26149550	0.5102534	-223.0106	-0.2782929	0.7885463	KLK15
cg11161417	0.384891	-178.4279	-0.2787412	0.6636322	SPACA3
cg16612699	0.5106326	-224.1558	-0.2787758	0.7894084	OR8B8
cg03993463	0.563938	-265.6174	-0.2790184	0.8429564	KCNJ15
cg20154346	0.3572274	-177.3036	-0.2807587	0.6379861	RAI2
cg16179125	0.3608437	-180.1405	-0.2823564	0.6432001	CTSZ
cg24027679	0.4932944	-222.819	-0.2826695	0.7759639	SLC2A7
cg19949550	0.4558808	-205.8167	-0.2829422	0.7388231	ASB2
cg14696820	0.3174019	-177.8609	-0.2835474	0.6009493	LCE1A
cg12456510	0.3957174	-188.8759	-0.2838454	0.6795629	TFF2
cg05766474	0.5344491	-253.3524	-0.2840329	0.818482	CCL16
cg26672426	0.3413076	-181.7499	-0.2849174	0.626225	PTGES
cg01103730	0.4123009	-195.1794	-0.2850694	0.6973703	IL20
cg06639544	0.4533954	-208.7921	-0.2851199	0.7385153	OR7A5
cg13726507	0.5417166	-265.8794	-0.2867016	0.8284182	CTAG2
cg25221254	0.4384227	-208.6	-0.2880214	0.7264441	ASAH3
cg14992108	0.4298312	-209.8724	-0.2904269	0.7202581	SNTB1
cg13019092	0.4393696	-219.5434	-0.2934089	0.7327785	PDZK1
cg04138756	0.4870442	-243.6324	-0.2935864	0.7806306	SPRR3
cg14204735	0.1703869	-209.3682	-0.2957587	0.4661456	CYB561
cg14162076	0.532065	-287.8008	-0.2976884	0.8297534	CLEC4D
cg22021786	0.412874	-219.7224	-0.2985565	0.7114305	WFDC8
cg21277505	0.5283129	-292.7625	-0.3008177	0.8291306	LOC284361
cg15503752	0.420312	-244.2118	-0.3094171	0.7297292	ST6GALNAC1
cg20373326	0.370323	-230.0284	-0.3106766	0.6809996	HSD17B2
cg26164184	0.5022024	-301.7491	-0.3127128	0.8149152	FCN2
cg22575540	0.4419567	-264.4109	-0.3139027	0.7558594	TRIM54
cg24024214	0.5046818	-316.1339	-0.3168676	0.8215494	BTNL8
cg12489960	0.3481986	-244.0208	-0.3206489	0.6688474	SGCB
cg02442161	0.4235378	-291.9978	-0.3295893	0.7531271	PI3
cg15711744	0.4303117	-301.5606	-0.3322116	0.7625234	ANP32D

cg25856811	0.3743167	-301.3283	-0.3436401	0.7179568	SPRR3
cg13021192	0.2487398	-292.7625	-0.3517762	0.6005161	CTSZ

Suppelementary table 14. Probes differentially methylated between *VHL*-mutated tumours and normal tissue.

TargetID	VHL.AVG_Beta	VHL.DiffScore	VHL.Delta Beta	normal.AVG_Beta	SYMBOL
cg02000005	0.5467802	350.6571	0.4078214	0.1389588	CRIP1
cg06204948	0.6026237	350.6571	0.3763087	0.226315	MARK2
cg13547644	0.5360144	350.6571	0.3612787	0.1747356	ACTA1
cg26509022	0.5556064	350.6571	0.3522311	0.2033753	ALDH1A3
cg13099330	0.5126672	350.6571	0.3487003	0.163967	RBP1
cg20025656	0.4878511	350.6571	0.3476234	0.1402277	ACTA1
cg06507244	0.7854767	350.6571	0.3389962	0.4464805	DHX32
cg07360692	0.5589018	350.6571	0.3350915	0.2238103	FLJ20032
cg23418591	0.4475849	350.6571	0.3344936	0.1130913	FLJ90166
cg18818531	0.7160312	350.6571	0.3329449	0.3830863	FOSL1
cg09871315	0.6295313	350.6571	0.3241573	0.305374	HOXA2
cg22628873	0.7767305	350.6571	0.323902	0.4528286	GGT6
cg01532103	0.3955467	350.6571	0.3200299	0.07551683	BSG
cg23283495	0.4309835	350.6571	0.3187875	0.1121959	IRF6
cg22392666	0.4826947	350.6571	0.3160658	0.1666288	FXYD7
cg04411625	0.3726659	350.6571	0.308938	0.06372797	CRIP1
cg23555120	0.5206136	350.6571	0.3070252	0.2135884	NUAK1
cg14611112	0.6953543	350.6571	0.3066743	0.38868	LCN6
cg15958424	0.5739948	350.6571	0.305484	0.2685107	ACPP
cg05627103	0.3895306	350.6571	0.3039972	0.08553335	KIAA1984
cg09595479	0.4825397	350.6571	0.3020754	0.1804643	PRPH
cg25936385	0.8582985	350.6571	0.2985719	0.5597265	FAIM2
cg04220579	0.3743143	350.6571	0.2978928	0.07642148	C7orf20
cg21660130	0.8551595	350.6571	0.2976953	0.5574641	CLCNKB
cg23668631	0.659946	350.6571	0.2956642	0.3642818	CAMKK1
cg14371590	0.6067922	350.6571	0.2950153	0.3117769	SLC26A10
cg01346152	0.5454737	350.6571	0.2944967	0.2509769	DHRS3
cg23855989	0.5133439	350.6571	0.2938649	0.2194789	AQP5
cg13906813	0.6908485	350.6571	0.2916585	0.3991899	HLA-DPA1
cg21608489	0.5324537	350.6571	0.2916502	0.2408035	POU2F2
cg02490034	0.6869414	350.6571	0.2897662	0.3971752	MEST
cg19224278	0.60417	350.6571	0.2893014	0.3148687	ALDH1A3
cg16363586	0.5994339	350.6571	0.2878244	0.3116095	BST2
cg02676865	0.4124649	350.6571	0.2875949	0.12487	UBTD1
cg08624249	0.7640241	350.6571	0.2849939	0.4790302	KIAA0889
cg24928687	0.814961	350.6571	0.2837105	0.5312504	EPHX1
cg16046376	0.6383562	350.6571	0.282903	0.3554532	PC
cg17296078	0.4009024	350.6571	0.2823689	0.1185335	UBTD1

cg17998964	0.4446734	350.6571	0.2808821	0.1637913	MARK2
cg17067528	0.3608509	350.6571	0.2791182	0.08173271	IER3
cg06958537	0.4819647	350.6571	0.2786984	0.2032664	ETS2
cg12177001	0.7988682	350.6571	0.2781299	0.5207384	IFI27
cg25580076	0.7493984	350.6571	0.2769866	0.4724118	MRPL43
cg24030630	0.3469318	350.6571	0.2752779	0.07165394	TCIRG1
cg12125117	0.6620348	350.6571	0.2752418	0.386793	GPR97
cg18172186	0.7950819	350.6571	0.2745892	0.5204926	KIAA1913
cg03605761	0.6290042	350.6571	0.2719032	0.357101	RNF126
cg21685427	0.8173883	350.6571	0.2703825	0.5470058	SGK2
cg26668713	0.4164905	350.6571	0.2689553	0.1475352	SIPA1
cg07285276	0.7906836	350.6571	0.2686048	0.5220789	RAPGEF1
cg04802221	0.6843781	350.6571	0.2658811	0.418497	LOC283849
cg04369341	0.446823	350.6571	0.2644469	0.182376	C20orf100
cg27114026	0.794076	350.6571	0.2632771	0.5307989	ELA1
cg03271907	0.8518438	350.6571	0.263187	0.5886568	MGMT
cg07236190	0.7475592	350.6571	0.2619531	0.4856061	AMDHD1
cg24012708	0.6830876	350.6571	0.2608652	0.4222224	HDHD3
cg25101936	0.7602313	350.6571	0.2596531	0.5005782	ZBTB16
cg02276665	0.6437225	350.6571	0.2595856	0.384137	CTNNA1
cg13269407	0.373593	350.6571	0.2590289	0.1145642	FLJ10945
cg21604615	0.4158446	350.6571	0.2588251	0.1570196	SYTL1
cg00446235	0.3903458	350.6571	0.25733	0.1330158	F11R
cg15236866	0.5195864	350.6571	0.2555445	0.2640419	DLX1
cg21667836	0.4484897	350.6571	0.2551173	0.1933723	PRMT8
cg24385322	0.3231154	350.6571	0.255092	0.06802341	HDAC3
cg03562120	0.6092091	350.6571	0.2548885	0.3543206	WISP2
cg17901463	0.3818444	350.6571	0.2543964	0.127448	GSTM1
cg15233681	0.7265753	350.6571	0.2536069	0.4729684	IL1R1
cg04587910	0.4290841	350.6571	0.2532781	0.175806	XLF
cg05859264	0.6996641	350.6571	0.2526832	0.4469808	MAPK13
cg06974755	0.343507	350.6571	0.2517619	0.09174509	RALGDS
cg21472642	0.6619656	135.2897	0.2507078	0.4112578	CHN2
cg21716693	0.5313275	350.6571	0.2505149	0.2808126	CPNE5
cg26069745	0.5620016	135.2897	0.2491071	0.3128945	HOXA2
cg23696949	0.4881983	350.6571	0.2479552	0.2402431	LAMC2
cg17790333	0.73996	350.6571	0.2473316	0.4926285	CYP11A1
cg01103836	0.9220673	350.6571	0.2471091	0.6749582	MYO9B
cg22467567	0.5247633	350.6571	0.2463315	0.2784319	IGFBP5
cg18392482	0.7915623	350.6571	0.2461943	0.545368	AMDHD1
cg10861599	0.5275854	350.6571	0.2455787	0.2820067	TNFSF4
cg15310873	0.6281904	128.7816	0.2454124	0.382778	C20orf85
cg13901526	0.3882084	350.6571	0.2451569	0.1430515	GYPC
cg15544721	0.7227138	132.4441	0.2442687	0.4784451	PPP1R9A
cg16616769	0.78408	350.6571	0.2440838	0.5399963	MGC35048

cg27652350	0.3754234	350.6571	0.2438986	0.1315248	ALDH1A3
cg08290628	0.720786	130.8027	0.2428196	0.4779663	CORO2B
cg10559803	0.6416352	125.643	0.2426377	0.3989975	RALGPS2
cg08368934	0.6849035	127.4265	0.2426098	0.4422937	GPR97
cg06285340	0.8389695	350.6571	0.241966	0.5970035	CYP11A1
cg20833786	0.685462	126.0039	0.2414892	0.4439728	MRGPRX3
cg14897096	0.804754	350.6571	0.2389115	0.5658425	PC
cg24101578	0.5780081	122.6457	0.2382943	0.3397138	CDH22
cg13587552	0.7825474	135.2897	0.2378663	0.544681	SCNN1D
cg24459563	0.7211055	124.9699	0.2370632	0.4840423	CACNG1
cg16854606	0.7159671	124.1542	0.2369777	0.4789894	DAND5
cg07572435	0.8101368	350.6571	0.2361757	0.5739611	LY6D
cg14409083	0.4746226	135.2897	0.2360464	0.2385762	EMP1
cg16077929	0.7394336	126.0039	0.2355281	0.5039055	CDKL1
cg04956511	0.5097899	127.4265	0.2349548	0.2748351	PTPN6
cg03440267	0.3450326	350.6571	0.2342654	0.1107672	F13A1
cg12052765	0.3657922	350.6571	0.2339586	0.1318336	CHAT
cg12866859	0.5960355	116.253	0.2337032	0.3623323	HEXIM1
cg22759185	0.7927269	135.2897	0.2334752	0.5592517	REEP6
cg02168291	0.77415	129.6312	0.2331308	0.5410191	CDH13
cg20427879	0.4343927	350.6571	0.2329705	0.2014222	EML2
cg19257550	0.7571352	126.4077	0.2327841	0.5243511	CA9
cg01724150	0.5111367	124.1542	0.232748	0.2783887	NMNAT3
cg14324200	0.3354699	350.6571	0.2326778	0.1027922	SAMD11
cg13164537	0.8052599	135.2897	0.2318316	0.5734284	CD226
cg27234090	0.3835475	350.6571	0.2316051	0.1519425	USP2
cg17820591	0.305077	350.6571	0.2311586	0.07391844	ENO3
cg09191232	0.5594805	115.4733	0.2307949	0.3286856	PAPSS1
cg20541456	0.4488353	135.2897	0.2302135	0.2186218	CYFIP2
cg18230216	0.670675	112.9752	0.2301703	0.4405047	NYX
cg01683883	0.3311715	350.6571	0.2301329	0.1010387	CMTM2
cg18702197	0.5953789	111.2979	0.2291742	0.3662047	HOXD3
cg13351161	0.4682353	128.0591	0.2286515	0.2395838	SCARA3
cg07251788	0.7158988	115.3945	0.2285555	0.4873433	CLTCL1
cg07078114	0.5160837	116.4751	0.2272268	0.2888569	FAM69B
cg21237418	0.4138851	350.6571	0.226728	0.1871571	RAB34
cg26822175	0.839664	350.6571	0.2266902	0.6129738	CRYBA4
cg20648149	0.7195987	113.7113	0.2264574	0.4931413	SYNE2
cg02674804	0.8554491	350.6571	0.2259002	0.6295489	REEP6
cg01126560	0.6236387	107.167	0.2258373	0.3978014	C9orf142
cg26511075	0.8190647	135.2897	0.2256454	0.5934194	FLJ25422
cg16517394	0.4281176	132.4441	0.2233869	0.2047307	TNFSF4
cg14444710	0.8106821	129.6312	0.2231834	0.5874987	PDPK1
cg24739326	0.4073599	135.2897	0.2224709	0.1848891	CHST8
cg20439022	0.5907054	104.0072	0.2222416	0.3684638	SLC16A8

cg25447894	0.4253955	130.8027	0.2218281	0.2035673	CSDC2
cg16393207	0.7131063	107.5086	0.2210477	0.4920586	GDPD5
cg24076884	0.4334815	126.4077	0.2210091	0.2124723	PCDHAC2
cg11319389	0.3738325	350.6571	0.2209202	0.1529123	C20orf100
cg05253327	0.7719323	117.0541	0.2208889	0.5510435	B3GNT1
cg01643580	0.3544478	350.6571	0.2207475	0.1337004	KCNK3
cg10521852	0.461314	117.4876	0.219773	0.241541	EDG4
cg03380645	0.8140736	125.643	0.2186121	0.5954615	SFTPB
cg11344614	0.7134748	104.8051	0.2182628	0.495212	JAM2
cg07044282	0.5895357	99.43771	0.2177761	0.3717596	ANGPTL1
cg20289949	0.4563799	116.058	0.2177697	0.2386102	HAAO
cg22601917	0.4719	112.401	0.2175808	0.2543193	H6PD
cg03852144	0.4830629	109.3	0.216811	0.2662518	GLRX
cg11318251	0.5602217	99.79023	0.2166703	0.3435514	BAALC
cg02506908	0.6391053	98.071	0.2166163	0.4224889	HPD
cg10978355	0.345729	350.6571	0.2165465	0.1291824	CKMT2
cg11716026	0.6939545	101.0267	0.216518	0.4774365	H19
cg19728382	0.7946569	117.8819	0.216278	0.5783789	STC2
cg01420388	0.6649272	98.27791	0.2157911	0.4491361	FBXO2
cg22536398	0.2590805	350.6571	0.2154402	0.04364023	NTN2L
cg18174542	0.3574583	350.6571	0.2149105	0.1425478	CSDC2
cg06575572	0.422483	120.6297	0.214504	0.207979	C22orf8
cg03192551	0.3152255	350.6571	0.2143086	0.1009169	CGI-69
cg06392241	0.3546007	350.6571	0.2135415	0.1410591	NUDT4
cg09539538	0.6588784	93.03526	0.2105636	0.4483148	C20orf42
cg16967583	0.6358476	91.86777	0.2102222	0.4256254	AGXT
cg20582779	0.4898548	99.9783	0.2098526	0.2800022	KIAA1822L
cg25374854	0.7211089	96.85287	0.2088053	0.5123036	ABR
cg10705800	0.5758737	90.80969	0.2086345	0.3672392	CITED4
cg01026744	0.7540043	100.9583	0.2079632	0.5460411	NAP1L5
cg02008416	0.6937289	92.85449	0.2076769	0.486052	TMEM61
cg11397854	0.3438962	350.6571	0.2072781	0.1366182	IQSEC1
cg13959523	0.2958796	350.6571	0.2071389	0.0887407	CHST8
cg11492040	0.7024648	92.85449	0.2067472	0.4957176	H19
cg08077673	0.4145004	111.4276	0.2061107	0.2083898	MEST
cg11164400	0.4087333	111.9161	0.205231	0.2035022	PPP1R9A
cg07047653	0.7630774	99.83517	0.2048976	0.5581798	AQP2
cg14546153	0.2824153	350.6571	0.2047458	0.07766946	FLJ90166
cg23539753	0.3833206	119.3316	0.2046309	0.1786897	SP100
cg18055007	0.4147924	108.5021	0.2039827	0.2108097	DDAH2
cg25483003	0.4035435	111.4276	0.2037556	0.1997879	ENTPD2
cg24511869	0.265835	350.6571	0.2035269	0.06230804	RELB
cg06197492	0.786277	103.0036	0.2030685	0.5832085	H19
cg06750167	0.4933911	91.49889	0.2025723	0.2908188	TESC
cg03096975	0.3757874	118.6309	0.2022845	0.1735029	EML2

cg06791102	0.5858598	84.25738	0.2020429	0.3838169	ARHGAP4
cg12091331	0.3832113	115.4903	0.2019289	0.1812824	PLAT
cg13055001	0.384516	114.9578	0.2018466	0.1826694	PPP1CA
cg22487322	0.8040839	106.2032	0.2017063	0.6023775	IL20RA
cg27360098	0.4957058	89.84649	0.2012706	0.2944352	ELN
cg08831348	0.4782138	92.12016	0.2011912	0.2770225	EML2
cg08858521	0.4202241	103.3389	0.2011071	0.219117	WFIKKN1
cg11783497	0.8077596	106.3789	0.2008415	0.606918	IL1RN
cg18342279	0.3070742	350.6571	0.2005869	0.1064874	ZAR1
cg25514304	0.6641119	84.25738	0.2005305	0.4635814	PSEN2
cg13960126	0.4411923	-86.43739	-0.2000353	0.6412276	CRB3
cg21755709	0.2762899	-85.46435	-0.2007811	0.477071	C21orf124
cg04143809	0.3709899	-83.2093	-0.2008623	0.5718522	FLJ39822
cg09027725	0.4284902	-86.16811	-0.2008789	0.6293691	COX4I2
cg12022621	0.3494335	-83.35494	-0.2013057	0.5507392	LAX1
cg22575540	0.5545133	-108.8463	-0.2013461	0.7558594	TRIM54
cg09414535	0.4019194	-85.23681	-0.2017788	0.6036983	GRIP1
cg02067021	0.486684	-94.33327	-0.2018958	0.6885798	DNAJC5B
cg27090216	0.15599	-105.4432	-0.2021004	0.3580904	TNFRSF10C
cg25983380	0.3126112	-85.14682	-0.2026295	0.5152407	GNAS
cg17907567	0.4190069	-87.28329	-0.2027867	0.6217936	HAMP
cg25882366	0.1813372	-100.3461	-0.20307	0.3844072	HOXB2
cg25813714	0.6398758	-151.7256	-0.2037179	0.8435937	CYP4F12
cg16772207	0.4165134	-88.02733	-0.2037186	0.620232	MYT1
cg15928132	0.4038335	-87.18691	-0.2037334	0.607567	CCKAR
cg27257987	0.4430272	-90.7382	-0.2039937	0.6470209	PSG4
cg25839227	0.4052781	-88.2916	-0.2047779	0.610056	ABI3
cg12456510	0.474294	-96.14628	-0.2052689	0.6795629	TFF2
cg08878744	0.3094683	-88.13448	-0.2058692	0.5153375	LCE1B
cg10127415	0.3202752	-87.90508	-0.2059743	0.5262495	MAGEB6
cg04810997	0.4648467	-96.20786	-0.206589	0.6714357	TAS2R60
cg23131950	0.5153583	-106.0624	-0.2071015	0.7224598	AP2S1
cg13125510	0.2632776	-92.21207	-0.2071249	0.4704025	C11orf44
cg01015871	0.4093629	-91.18626	-0.2073789	0.6167418	MT4
cg16626670	0.4385087	-94.15893	-0.2076975	0.6462061	CLEC4G
cg18056600	0.1572234	-110.5022	-0.2077168	0.3649402	ZMYND15
cg13204181	0.4232654	-92.8588	-0.207901	0.6311664	GH1
cg01718139	0.557219	-119.8071	-0.2085749	0.7657939	UNQ3033
cg24506604	0.4963033	-105.7533	-0.2101859	0.7064892	LOC144501
cg25221254	0.5158291	-110.6826	-0.210615	0.7264441	ASAH3
cg00644033	0.706861	-239.7324	-0.2107055	0.9175664	MUC3B
cg10249734	0.3865685	-93.59346	-0.2111289	0.5976974	SECTM1
cg24619694	0.4545559	-99.9588	-0.2112799	0.6658359	TAGLN
cg24697329	0.5280487	-115.1029	-0.211531	0.7395797	ARHGEF4
cg14287742	0.5381047	-118.1454	-0.2116917	0.7497964	BLZF1

cg07824742	0.5473509	-121.1164	-0.2117202	0.7590712	DBH
cg01103730	0.4856273	-105.5567	-0.211743	0.6973703	IL20
cg05348870	0.5576527	-124.7002	-0.2117438	0.7693964	TNFSF14
cg22243733	0.5113198	-111.4193	-0.2120333	0.7233531	OLFML1
cg06277657	0.2671968	-96.7354	-0.2123671	0.4795639	DGKI
cg21948655	0.5864288	-137.426	-0.2124448	0.7988736	SMCP
cg26884581	0.1845808	-108.9183	-0.2128018	0.3973826	PYGM
cg23873703	0.5667789	-129.8072	-0.2129248	0.7797037	KCNAB1
cg27566805	0.2759641	-97.83491	-0.2142743	0.4902384	USH2A
cg24939733	0.3948938	-97.55991	-0.2145369	0.6094307	FLJ38159
cg21457804	0.5355688	-122.0496	-0.2149458	0.7505146	CT45-2
cg23047271	0.1223429	-129.0228	-0.2154442	0.3377871	PRICKLE2
cg17741572	0.5812785	-140.7538	-0.2157611	0.7970396	CFB
cg10894512	0.4177922	-101.0243	-0.2161866	0.6339787	ACTA2
cg18530716	0.1243417	-129.4841	-0.2166048	0.3409466	SLC16A11
cg24516901	0.2480568	-102.6197	-0.2167011	0.4647579	FLJ22746
cg24884084	0.2130574	-108.1505	-0.2174709	0.4305283	SPRR1B
cg19384697	0.2827333	-100.6838	-0.2178504	0.5005838	UPK3B
cg01309153	0.6300664	-176.4296	-0.2184185	0.8484849	SURF1
cg00594952	0.3462249	-99.94583	-0.218727	0.564952	RIMS3
cg13726507	0.6096038	-162.6268	-0.2188144	0.8284182	CTAG2
cg15780361	0.3325471	-99.98878	-0.2188436	0.5513908	ALS2CR11
cg14440664	0.3422706	-100.0966	-0.2189523	0.5612229	PDCD1LG2
cg04273431	0.1701062	-118.2531	-0.2189622	0.3890684	PRR3
cg26164184	0.5957833	-154.8452	-0.2191319	0.8149152	FCN2
cg16176600	0.5151863	-122.2168	-0.2193382	0.7345245	FRK
cg24694549	0.4205299	-105.319	-0.2197708	0.6403007	GRIP1
cg02833725	0.4316808	-107.6667	-0.2207159	0.6523967	ISG20L2
cg27337148	0.2156696	-111.0991	-0.2208849	0.4365545	CAMK1G
cg26927807	0.4724464	-114.7313	-0.2212062	0.6936526	BTBD2
cg06818777	0.1341331	-132.3157	-0.2225558	0.3566888	CHAD
cg05670596	0.2647535	-107.236	-0.2230626	0.4878161	CCRL2
cg20334738	0.425726	-109.7581	-0.2231558	0.6488818	MAB21L2
cg25203980	0.5852529	-159.4632	-0.2246267	0.8098795	CENTB5
cg21372914	0.4889256	-123.3136	-0.2251026	0.7140282	CLEC4M
cg02397720	0.1208694	-140.0461	-0.2252849	0.3461542	RAB17
cg25119415	0.4626245	-117.9672	-0.2253164	0.687941	MNDA
cg04391540	0.3612602	-107.4012	-0.2255519	0.5868121	ZNF541
cg24851490	0.2784675	-108.8746	-0.2257794	0.5042468	RNASE2
cg09837803	0.2973253	-111.3065	-0.2291451	0.5264704	IL16
cg03782453	0.1547847	-133.2667	-0.2295982	0.3843829	FLJ90575
cg24024214	0.5916532	-174.0463	-0.2298962	0.8215494	BTNL8
cg16051685	0.5046632	-135.2377	-0.2305613	0.7352245	TRIM63
cg23881725	0.1985403	-124.5514	-0.2311435	0.4296838	DLEC1
cg09467501	0.1400947	-140.5345	-0.2320184	0.3721131	PYY

cg00626119	0.303601	-115.3569	-0.2331855	0.5367865	NTRK1
cg16098726	0.6232243	-207.8797	-0.233795	0.8570194	GP9
cg04138756	0.5456438	-158.4216	-0.2349868	0.7806306	SPRR3
cg10414946	0.3940531	-122.4565	-0.2369063	0.6309593	MS4A2
cg24499411	0.422236	-128.4821	-0.2389346	0.6611706	TNS3
cg15590780	0.2580026	-124.7404	-0.2393376	0.4973402	USH2A
cg17910564	0.3399887	-122.1296	-0.2394713	0.57946	VDAC3
cg14992108	0.4806582	-141.731	-0.2395999	0.7202581	SNTB1
cg03251857	0.5682601	-180.0853	-0.239845	0.8081051	PPAPDC3
cg12489960	0.4289993	-130.7036	-0.2398481	0.6688474	SGCB
cg21065959	0.4188224	-129.2351	-0.2399581	0.6587805	LCE1E
cg00334507	0.3840448	-125.5719	-0.2402581	0.6243029	MVP
cg04505023	0.4582959	-138.6711	-0.241447	0.6997429	SPRR1A
cg06639544	0.4968438	-150.2192	-0.2416714	0.7385153	OR7A5
cg14696820	0.3584007	-126.4067	-0.2425486	0.6009493	LCE1A
cg27020690	0.1105422	-164.7522	-0.2426517	0.3531938	TERC
cg16990174	0.3034991	-127.1891	-0.2439889	0.547488	RYBP
cg19368582	0.4028572	-132.4441	-0.2441751	0.6470323	MMRN2
cg17820828	0.4796043	-149.9318	-0.2449361	0.7245404	KCNQ1
cg21717724	0.3362814	-128.7816	-0.2453195	0.5816008	PSMD5
cg01530101	0.3825043	-131.7719	-0.2454792	0.6279835	KCNQ1DN
cg03221619	0.3075009	-129.1396	-0.2457908	0.5532917	FCER2
cg06123346	0.3726311	-133.2126	-0.247364	0.6199951	ATP4A
cg22784047	0.4392051	-143.0564	-0.2473928	0.6865979	MVP
cg12513481	0.3127575	-131.0239	-0.2475051	0.5602626	SCAP1
cg08684473	0.5006433	-161.8444	-0.24774	0.7483833	LILRB5
cg19917856	0.265061	-133.4034	-0.2477948	0.5128558	LOC342897
cg00622552	0.1315199	-162.3794	-0.2478962	0.379416	ODF3L1
cg01993576	0.1414131	-159.3175	-0.2483435	0.3897566	SLC29A1
cg15626350	0.218858	-139.948	-0.248604	0.467462	ESR1
cg15503752	0.4791507	-159.0379	-0.2505784	0.7297292	ST6GALNAC1
cg19717326	0.1952128	-148.7432	-0.2521406	0.4473534	MYADM
cg06436504	0.4173086	-145.932	-0.2523321	0.6696407	DOC1
cg10275770	0.1867097	-152.0804	-0.2533452	0.4400549	ICAM2
cg06714705	0.4914002	-170.2989	-0.2545714	0.7459716	OLFML1
cg10052840	0.2405236	-143.9704	-0.2546015	0.4951251	SEMA6B
cg26185508	0.4214747	-156.2185	-0.2585909	0.6800656	CDCP2
cg04551925	0.6212732	-281.2505	-0.260051	0.8813242	AQP1
cg25833031	0.5265922	-202.2105	-0.2621065	0.7886987	PAPD1
cg19903229	0.2728539	-151.044	-0.2625493	0.5354032	C14orf105
cg24407065	0.5396824	-213.7277	-0.2635833	0.8032658	BLZF1
cg14706739	0.4280058	-167.1283	-0.2645702	0.6925761	EPB49
cg04144768	0.4122399	-167.1283	-0.266863	0.6791029	DDC
cg16179125	0.3759146	-161.3132	-0.2672855	0.6432001	CTSZ
cg20154346	0.3693581	-162.3921	-0.268628	0.6379861	RAI2

cg26264314	0.4806181	-192.5611	-0.2688154	0.7494335	NALP5
cg21541083	0.5375924	-224.8784	-0.2690037	0.8065962	STXBP2
cg14894144	0.2064777	-172.8985	-0.2729762	0.479454	LAMA3
cg24816455	0.2045622	-174.0463	-0.2735757	0.4781379	SEMA3B
cg07997737	0.2975732	-166.6886	-0.2747194	0.5722926	NRTN
cg22374142	0.222408	-173.2088	-0.2752685	0.4976765	HSF4
cg22341310	0.1533717	-190.8205	-0.2759618	0.4293335	ZNF541
cg13521229	0.2770494	-169.0038	-0.2760703	0.5531197	JOSD2
cg01565918	0.5720414	-287.8604	-0.2810268	0.8530682	GNAS
cg19728223	0.2517124	-185.8537	-0.2866663	0.5383787	KCNQ1
cg19954000	0.2137332	-192.9937	-0.2882531	0.5019863	FGF1
cg06101324	0.2050041	-197.7227	-0.2904195	0.4954236	SPRR1A
cg18780284	0.3905918	-202.9911	-0.2908018	0.6813936	SPRR1B
cg13021192	0.3060146	-196.9446	-0.2945015	0.6005161	CTSZ
cg01484156	0.2536793	-200.5183	-0.2964454	0.5501247	NCALD
cg15060813	0.2084816	-213.0313	-0.3009458	0.5094273	LRFN3
cg27619475	0.2515306	-214.2036	-0.3049555	0.5564861	SLC16A5
cg12391921	0.3087181	-215.8704	-0.3056872	0.6144053	ITGB1BP2
cg08450982	0.2792279	-221.1291	-0.3095095	0.5887374	NUMBL
cg24901474	0.2420335	-222.5022	-0.3095869	0.5516204	RGS5
cg02351381	0.1538868	-241.5301	-0.3103077	0.4641945	C12orf34
cg06536578	0.3374255	-232.333	-0.3127201	0.6501456	JPH4
cg10677144	0.3950145	-255.2249	-0.3162332	0.7112477	MYOM1
cg24670715	0.1770853	-255.0376	-0.3225577	0.4996431	ANGPT2
cg21277505	0.4919105	-347.7558	-0.3372201	0.8291306	LOC284361
cg25856811	0.3781078	-303.3381	-0.339849	0.7179568	SPRR3
cg10604646	0.1913526	-294.273	-0.3457956	0.5371482	RGS5
cg14204735	0.1071686	-347.5609	-0.358977	0.4661456	CYB561
cg01335367	0.1614577	-333.122	-0.3618931	0.5233509	C12orf34
cg21949305	0.3307221	-347.7558	-0.3945665	0.7252886	ADORA2A
cg22189286	0.2823619	-347.7558	-0.4085908	0.6909527	HSPB8

Supplementary Table 15 – 100 probes differentially methylated in *VHL*-mutated, *RET*-mutated, *NF1*-mutated tumours and tumours without known mutation.

Probe	Associated gene
cg15590780	USH2A
cg14706739	EPB49
cg19917856	LOC342897
cg03782453	FLJ90575
cg21065959	LCE1E
cg14696820	LCE1A
cg06123346	ATP4A
cg14992108	SNTB1

cg25119415	MNDA
cg08450982	NUMBL
cg27020690	TERC
cg07824742	DBH
cg18056600	ZMYND15
cg14204735	CYB561
cg10861599	TNFSF4
cg04144768	DDC
cg23881725	DLEC1
cg13021192	CTSZ
cg08624249	KIAA0889
cg03221619	FCER2
cg15503752	ST6GALNAC1
cg13521229	JOSD2
cg06436504	DOC1
cg06639544	OR7A5
cg06507244	DHX32
cg10052840	SEMA6B
cg14409083	EMP1
cg15060813	LRFN3
cg24694549	GRIP1
cg24024214	BTNL8
cg10604646	RGS5
cg25221254	ASAH3
cg00334507	MVP
cg01103730	IL20
cg07997737	NRTN
cg19954000	FGF1
cg21457804	CT45-2
cg13726507	CTAG2
cg12022621	LAX1
cg27619475	SLC16A5
cg24851490	RNASE2
cg02351381	C12orf34
cg04810997	TAS2R60
cg16517394	TNFSF4
cg19368582	MMRN2
cg10127415	MAGEB6
cg26927807	BTBD2
cg00626119	NTRK1
cg06536578	JPH4
cg00644033	MUC3B
cg10414946	MS4A2
cg04505023	SPRR1A
cg12489960	SGCB

cg00594952	RIMS3
cg21948655	SMCP
cg05670596	CCRL2
cg24901474	RGS5
cg13204181	GH1
cg10677144	MYOM1
cg21372914	CLEC4M
cg09414535	GRIP1
cg20334738	MAB21L2
cg18780284	SPRR1B
cg04138756	SPRR3
cg19717326	MYADM
cg22575540	TRIM54
cg24101578	CDH22
cg12456510	TFF2
cg22784047	MVP
cg26884581	PYGM
cg01718139	UNQ3033
cg08684473	LILRB5
cg12513481	SCAP1
cg16363586	BST2
cg24670715	ANGPT2
cg16990174	RYBP
cg09467501	PYY
cg06101324	SPRR1A
cg16179125	CTSZ
cg27257987	PSG4
cg27090216	TNFRSF10C
cg14894144	LAMA3
cg16772207	MYT1
cg19728223	KCNQ1
cg01015871	MT4
cg15780361	ALS2CR11
cg15928132	CCKAR
cg02067021	DNAJC5B
cg05348870	TNFSF14
cg22374142	HSF4
cg20154346	RAI2
cg26164184	FCN2
cg25856811	SPRR3
cg09027725	COX4I2
cg01335367	C12orf34
cg10275770	ICAM2
cg01103836	MYO9B
cg17907567	HAMP

cg04273431	PRR3
cg16626670	CLEC4G

Supplementary Table 16– Probes with AUROC > 0.7

Probe	AUROC	Average β Malignant tumours	Average β Benign tumours	Δβ
cg13608094	0.7414	0.5319676	0.702949741	-0.170982141
cg23412777	0.7517	0.24781474	0.409473493	-0.161658753
cg00626119	0.8103	0.186285111	0.3352027	-0.148917589
cg25781162	0.7241	0.40751045	0.560333634	-0.152823184
cg05140736	0.7034	0.33142072	0.442233848	-0.110813128
cg06303238	0.7172	0.33761704	0.468263393	-0.130646353
cg07426960	0.7345	0.220085059	0.339183866	-0.119098807
cg10710439	0.7241	0.20627716	0.29835841	-0.09208125
cg09276451	0.7	0.2537176	0.351672666	-0.097955066

Supplementary table 17 – Non-tumoural contamination estimated by ASCAT for cases with available SNP array data.

Sample	Contamination %	Methylation cluster	Mutated gene	Malignant
BP9_9028.2_8R03C01	15.0	A	NF1	X
BP11_5193.1_4R08C01	27.0	A		X
BP9_7356.1_9R02C01*	17.0	A		X
BP9_5629.1_6R06C01*	19.0	A		X
BP9_6457.1_6R07C01*	20.0	A		
BP9_7095.1_8R06C01	20.0	A		
BP7_6359.1_4R01C01	22.0	A		
BP9_5178.1_4R02C01	25.0	A		X
BP9_6937.1_9R01C01	25.0	A		X
BP9_7294.1_5R07C01	25.0	A	VHL	
BP7_9266.1_1R03C01#	29.0	A	NF1	X
BP9_8432.2_11R02C01	30.0	A	NF1	
BP9_9086.1_10R01C01	30.0	A		
BP9_7087.1_1R02C01	31.0	A		X
BP9_9424.1_2R06C01	33.0	A	RET	
BP9_8592.1_12R01C01	34.0	A	RET	
BP12_8016.1_12R04C01	36.0	A	NF1	
BP9_6259.1_7R07C01	36.0	A	VHL	
BP9_8120.1_7R08C01	43.0	A	HRAS	
BP12_9263.2_12R05C01 #	47.0	A	NF1	X
BP9_7880.1_1R05C01	48.0	A	RET	
BP9_10058.1_8R01C01	52.0	A	RET	X
BP12_9233.1_8R08C01	55.0	A	RET	

BP9_6511.1_5R02C01	56.0	A		
BP9_9045.1_9R08C01	89.0	A		
BP3_7307.1_1R04C01	NA	A	HRAS	
BP9_6601.1_6R08C01	18.0	B	SDHB	
BP9_7774.1_5R08C01	18.0	B	RET	
BP9_7160.1_5R05C01	21.0	B	RET	
BP7_7174.1_3R06C01	38.0	B		
BP7_7899.2_3R04C01	53.0	B	VHL	
BP7_8202.1_4R06C01	56.0	B	NF1	
BP12_6643.1_2R02C01	61.0	B	VHL	
BP12_7810.1_7R02C01	71.0	B	VHL	
BP9_8774.1_1R06C01	76.0	B	VHL	

Supplemental figure 1. Extended heatmap including normal medulla

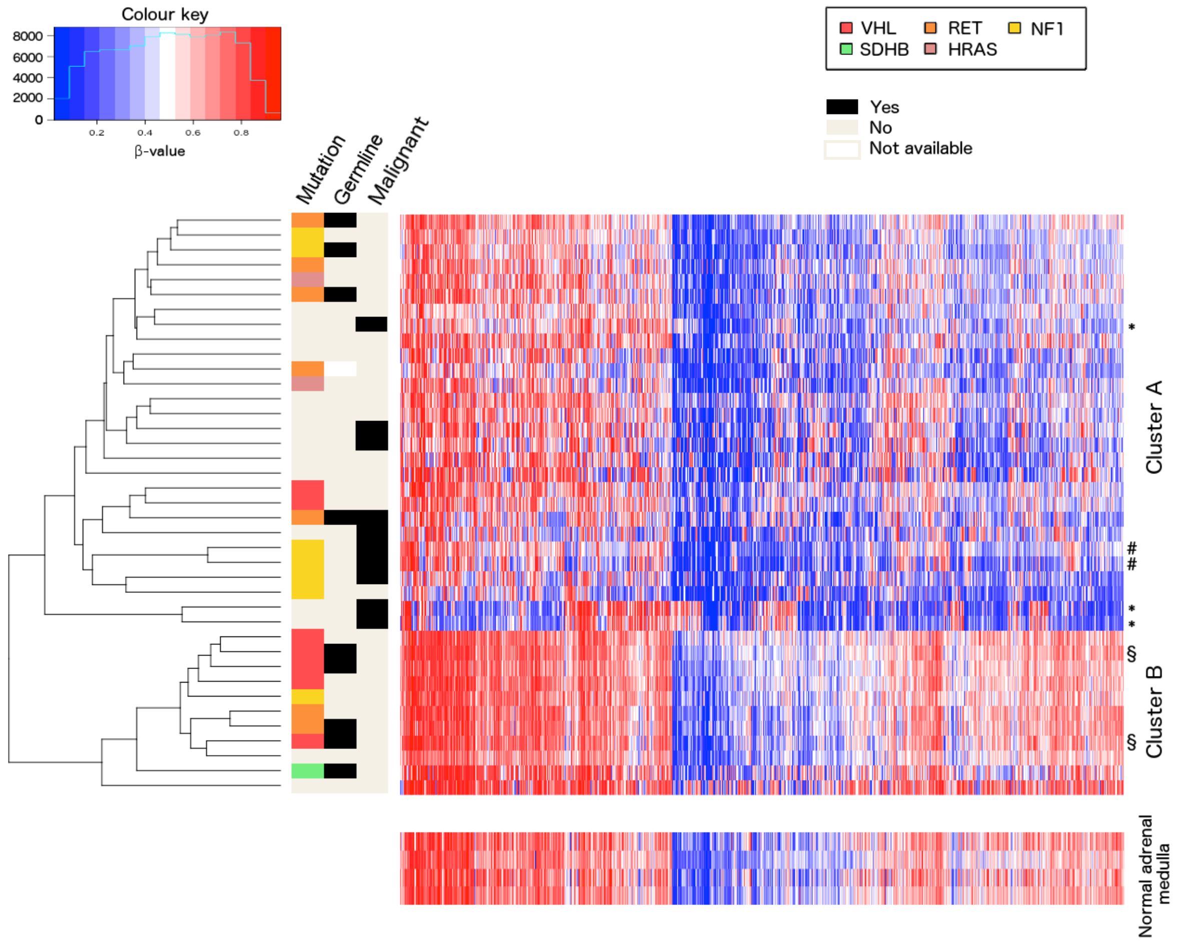

Supplemental figure 2. Kaplan-Meier survival analysis. No difference in survival is seen between the clusters.

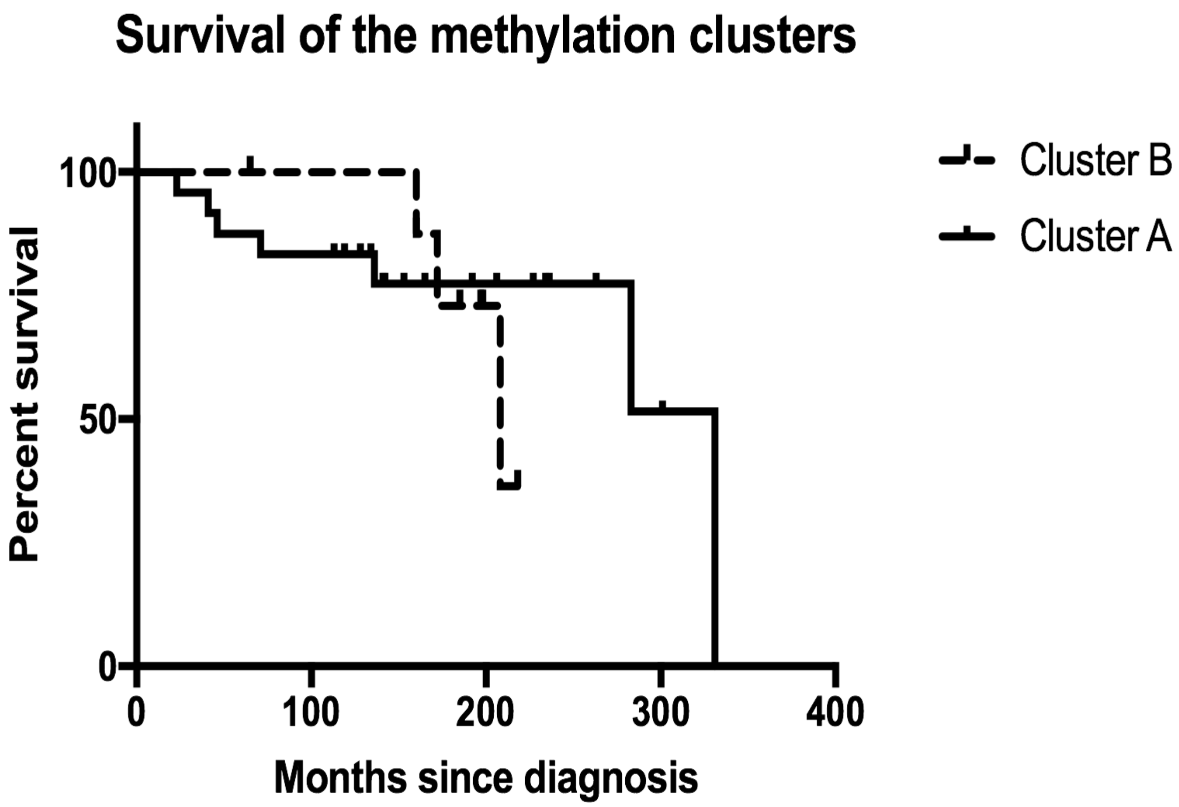

Supplemental figure 3. Venn diagram of probes differentially methylated between tumours with mutations in specific genes and normal medulla.

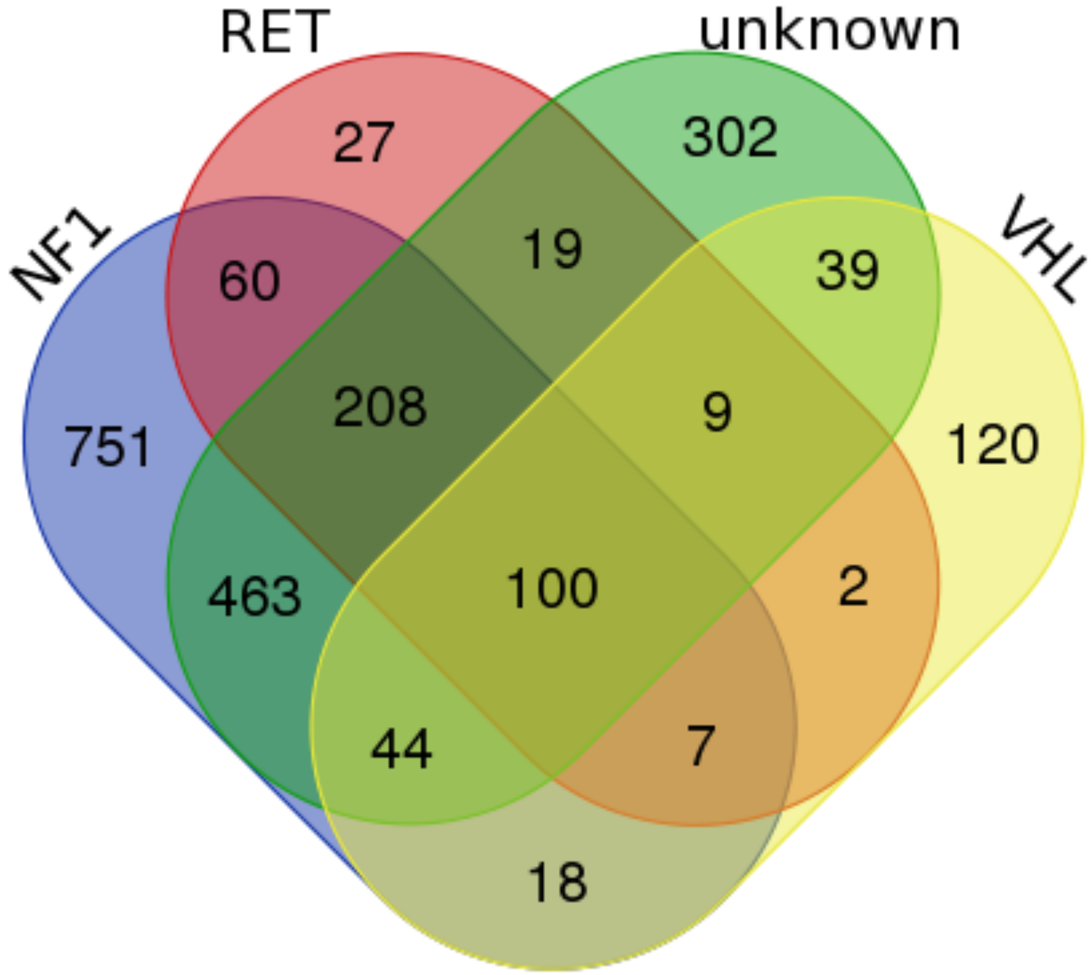

Supplemental figure 4. Somatic Copy Number Aberrations versus Methylation index

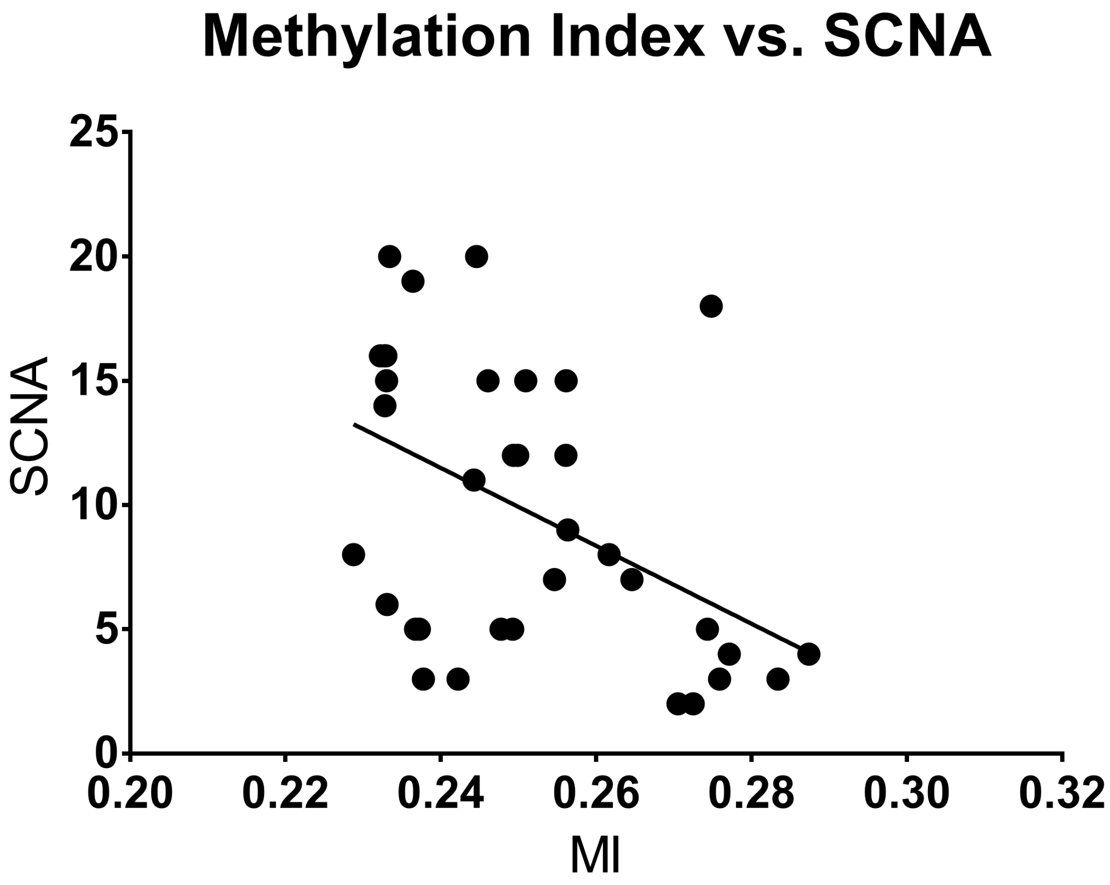

Supplemental figure 5. Heatmap for a case with multiple analysed tumours. P=Primary, M=Metastasis, R=Recurrence.

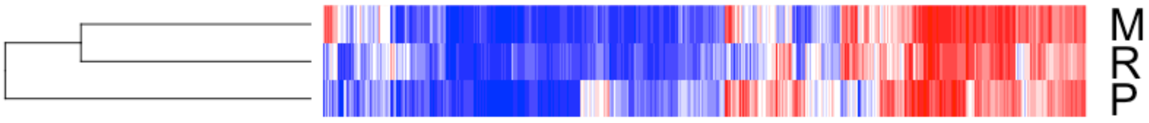

Supplemental figure 6. Heatmap for a case with multiple analysed tumours. P=Primary, M=Metastasis.

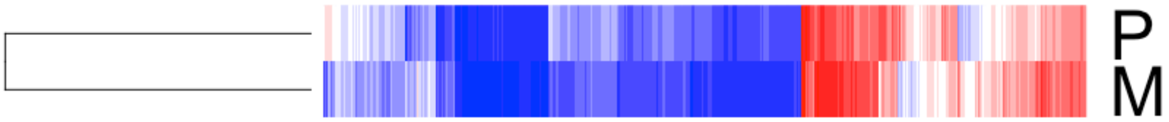

Supplemental figure 7. Heatmap for a case with multiple analysed tumours. P1 and P2 are two different primary tumours (bilateral disease).

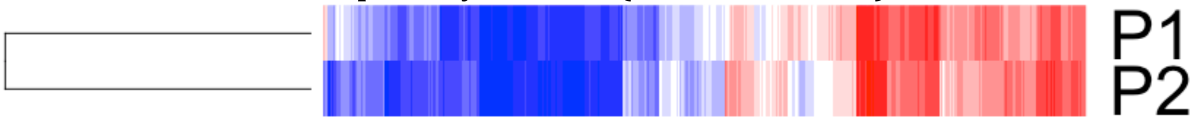

Supplemental figure 8. Relative *PNMT* expression in the two methylation clusters. No significant difference is detected.

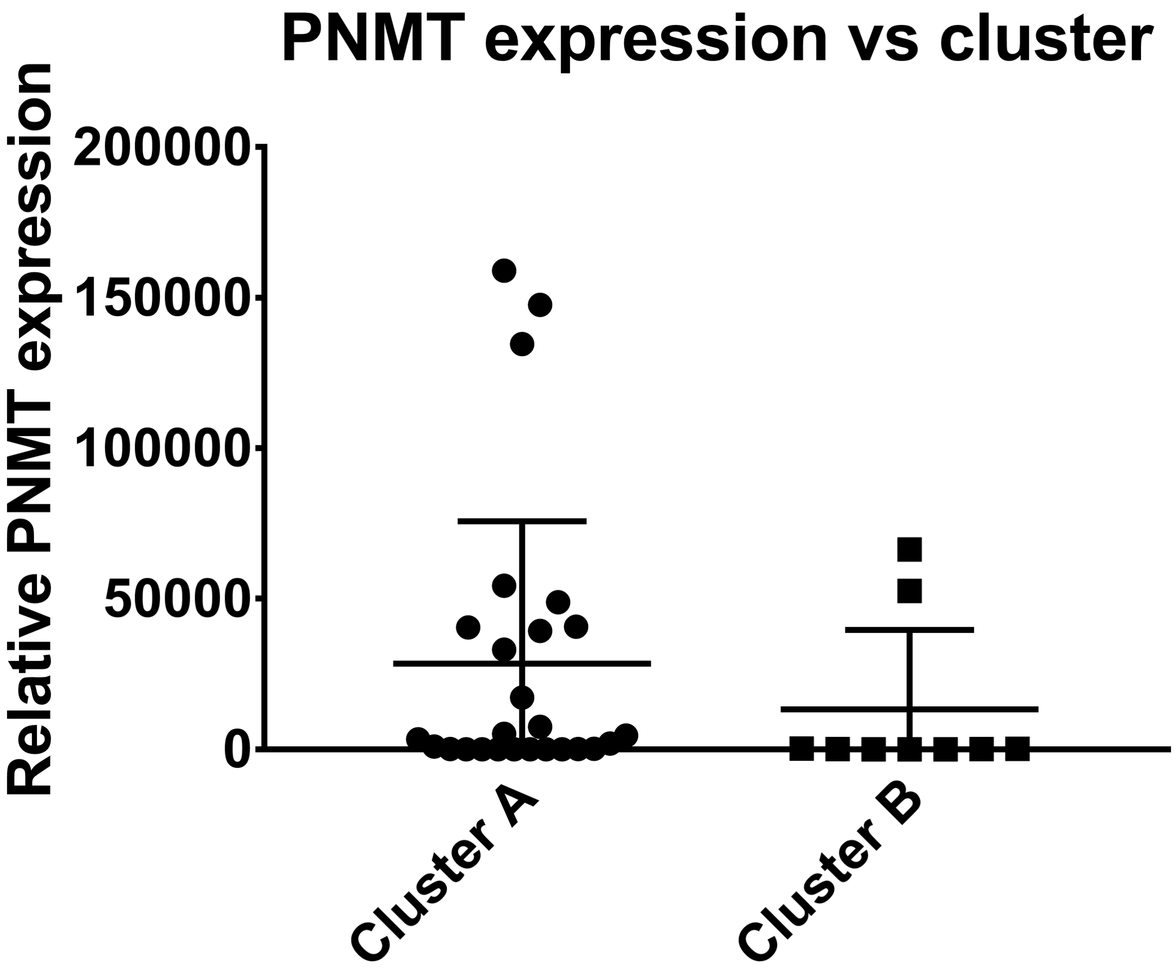

Supplemental figure 9. Relative *PNMT* expression in tumours with different mutations.

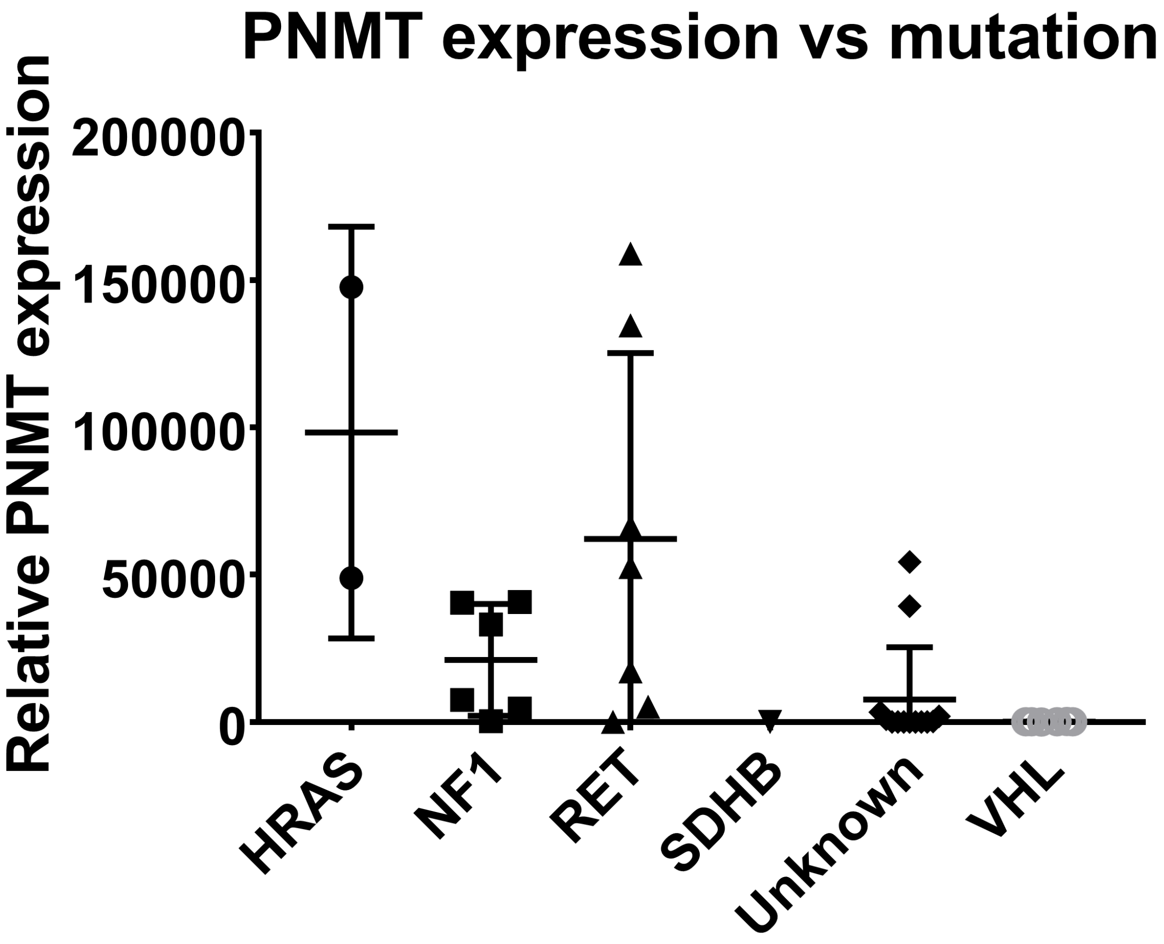

Supplement: Supplementary Information [file srep44943-s1.pdf]
